# Approach to Standardized Material Characterization of the Human Lumbopelvic System: Testing and Evaluation

**DOI:** 10.3390/bioengineering12080862

**Published:** 2025-08-11

**Authors:** Marc Gebhardt, Sascha Kurz, Fanny Grundmann, Thomas Klink, Volker Slowik, Christoph-Eckhard Heyde, Hanno Steinke

**Affiliations:** 1 Institute of Experimental Mechanics, Faculty of Civil Engineering, Leipzig University of Applied Sciences, 04277 Leipzig, Germany; marc.gebhardt@htwk-leipzig.de (M.G.);; 2 Institute of Anatomy, Faculty of Medicine, Leipzig University, 04103 Leipzig, Germany; 3 ZESBO (Zentrum zur Erforschung der Stütz- und Bewegungsorgane)—Center for Research on Musculoskeletal Systems, Faculty of Medicine, Leipzig University, 04103 Leipzig, Germany; 4 Clinic of Trauma, Orthopedic and Septic Surgery, Hospital St. Georg gGmbH, 04129 Leipzig, Germany; 5 Department of Orthopaedic, Trauma and Plastic Surgery, University of Leipzig Medical Center, 04103 Leipzig, Germany

**Keywords:** material characterization, standardization, human pelvis, cortical bone, trabecular bone, soft tissue, fascia, ligaments

## Abstract

The osseo-ligamentous lumbopelvic complex is essential for musculoskeletal load transfer, yet location-specific material data and standardized test protocols remain scarce, which is a hindrance for comparability. Based on 91 specimen locations per cadaver (five cadavers, average age: 77.3 years), we developed detailed methods for specimen preparation and mechanical testing (bending, tensile, and compression) with defined boundary conditions. Multiple measurements were taken to assess repeatability. The proposed methods allow us to identify location-specific properties of the lumbopelvic system for the first time. Cortical bone exhibited an elastic modulus of 1750 MPa and an ultimate strength of 28.2 MPa, while those of trabecular bone were 32.7 MPa and 1.26 MPa, and soft tissues revealed values of 148 MPa and 14.3 MPa for fascial tissue and 103 MPa with 10.7 MPa for ligamentous tissue, respectively. The quantified properties for cortical and trabecular bone and soft tissues not only enhance the comparability of material properties but also support more accurate numerical simulations and implant design. Furthermore, the ease of implementation and standardization of these methods enable their widespread application, as well as the accumulation of a broad database and the setting of benchmarks for future investigations.

## 1. Introduction

The lumbopelvic system is crucial for transferring loads between the upper and lower body, yet relevant standardized mechanical testing protocols and consistent material data remain limited. This limitation relates not only to osseous structures but also to strong ligaments, cartilaginous tissues, and muscle tissues, which all contribute to the complex functions of this system [1,2,3,4,5]. The biomechanical integrity of the lumbopelvic system is of great importance for maintaining quality of life, which is why it is frequently the subject of research in orthopedic and trauma surgery [6].

From a mechanical perspective, a model is only as reliable as the input data on which it is based. Numerical simulations are increasingly being used as model systems in anatomy and medicine [7,8], and the reliability of the material properties underlying these simulations is particularly important. Determining the material properties requires both imaging studies to determine geometry [9] and experimental investigations using load tests with cadavers. Undoubtedly, it is desirable and preferable to use in vivo data to determine the material properties of the lumbopelvic complex, but destructive testing on living tissue is not feasible. Consequently, cadaveric specimens remain the most reliable source of experimental data and provide critical material properties that are needed for numerical simulations and biomechanical models.

Indirect methods have been explored to derive material properties, such as computer tomography (CT) methods or other imaging-based approaches, but their accuracy is currently limited. Helgason et al. [10,11] and Taddei et al. [12] have shown that the errors and deviations associated with these methods are significantly larger than those involved in cadaveric testing. These approaches are undeniably innovative and represent a promising future direction, but they remain insufficient. Thus, there is a continued need for standardized experimental protocols using cadaveric specimens. These protocols form the foundation for reliable input data, which is essential for advancing numerical simulations, implant design, and biomechanical research.

The standardization of test conditions is an important prerequisite for deriving material properties. The methods used must be robust and specific to the type of tissue investigated. However, no standardized test procedures have been published for determining the material properties of the human pelvis. Furthermore, there is no reliable database of material properties for different areas of the bony pelvis. Dalstra et al. [13] conducted basic research on the material properties of trabecular bone in the pelvis, and Ebraheim et al. [14] radiologically investigated the structure of the sacrum.

Numerical simulations based on the finite element (FE) method are used not only for basic scientific investigations of biomechanical issues in medicine, but also for developing and optimizing implants. A well-founded material database is a crucial prerequisite for such simulations. Dalstra and Huiskes [15] and Linstrom et al. [16] have demonstrated that material properties can be site-dependent due to specific load-bearing areas relating to Wolff’s law [17]. Comparative studies [18,19] have shown that variability in material properties can be pronounced and is strongly dependent on the location and type of harvesting for both bony and soft tissue structures [6,20,21,22,23].

A previous study has addressed the relevance of standardization of the preparation and storage of specimens in regard to repeatability [24]. In the present study, we extend that investigation to the testing and evaluation of samples collected using the methods from that study. The axial tensile test for soft tissue specimens, the axial compression test for cancellous bone, and the three-point bending test for cortical bone are considered to be the most suitable methods in this context based on the natural load-bearing behavior of the tissue and the methods’ applicability. By proposing and applying suitable boundary conditions for biomechanical tests, the aim of this investigation is to contribute to the standardization of material testing and enhance the validity of the data obtained by reducing experimental uncertainties and optimizing data evaluation.

For the first time, location-specific material data for the human lumbopelvic complex were generated, and the repeatability and applicability of the standardized methods were demonstrated across multiple specimens.

**Main objectives:** The primary objectives of this study were to (1) standardize the testing methods used for biomechanical material characterization of the human lumbopelvic system, (2) standardize the material properties derived from these tests, and (3) demonstrate the applicability of these test methods for obtaining standardized material properties.

**Secondary objectives:** The secondary aim of the study was to create a data basis for the location-specific and tissue-specific material properties of the lumbopelvic complex. Such values have not been consistently reported in the literature thus far. In this context, we investigated the influence of the harvesting location as well as cadaver-specific influences, such as age, individual anatomical variations, bone density, and pre-existing medical conditions, on the measured material properties.

## 2. Materials and Methods

### 2.1. Specimen Acquisition

All of the donors of the specimens used in this study originated from the Institute of Anatomy of Leipzig University. During their lifetime, all of the individuals had provided written consent to donate their bodies to medical education and research. The body-donor program is regulated by the Saxonian Death and Funeral Act of 1994 (3rd section, paragraph 18, item 8). Accordingly, institutional approval for using post-mortem tissues was obtained from the Institute of Anatomy of Leipzig University. The authors declare that all of the experiments were performed according to the ethical principles of the Declaration of Helsinki. Cadaveric specimens were collected by following a previously established protocol for the standardization, preparation, and preservation of human tissue samples [24]. A total of seven human lumbopelvic systems with previously defined ligamentous apparatuses were tested. The first two pelvises were used in pre-tests for the development and validation of the preparation and testing procedures. The evaluation included lumbopelvic systems from three female and two male cadavers. The average age of the cadavers was 77.3 years (range: 53.2 to 89.2 years). We obtained 88 soft tissue, 146 trabecular, and 138 cortical specimens. No abnormalities were found in the deceased individuals’ medical histories according to the provided ICD-10 codes (10th revision of the International Statistical Classification of Diseases and Related Health Problems).

### 2.2. Testing Procedure

**Figure 1:**
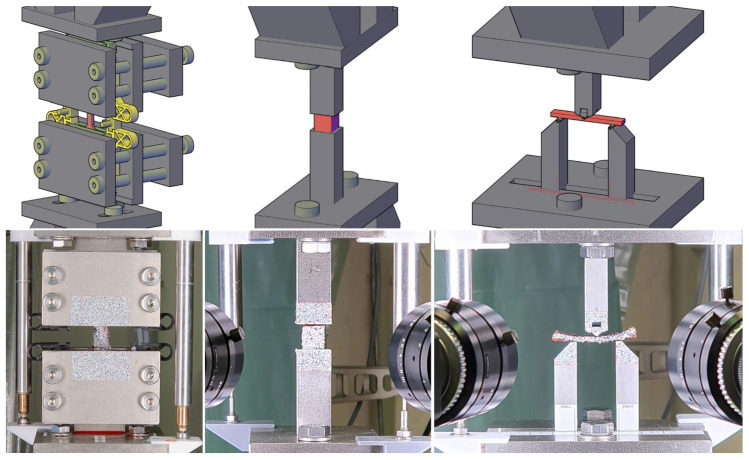
Experimental setup. Overview of the experimental setup modules. (**left**) Axial tension test. (**middle**) Axial compression test. (**right**) Three-point bending test. (**top**) Models. (**bottom**) Actual testing setup.

A specimen-clamping design based on a model from Scholze et al. [25] was employed in the axial tensile test. The design was manufactured by 3D-printing using fused deposition modeling, as shown on the left in Figure 1. With its pyramid-shaped profiling, this clamping design is optimized for static tests on soft tissue. Its compact design, complemented by spacers, allows for a straightforward and damage-free setup. To compensate for transverse creep deformation, which may result in slippage, an elastic energy accumulator was integrated into the pretensioning device and tightened. Supplementary Materials (File S1) provides more detail about the experimental setups (Figure 1) and materials used throughout the study. Blueprints of the test modules and geometrical models of all 3D-printed parts are provided in Supplementary Materials (File S2).

The dimensions of the individual specimens were measured using a digital caliper (150 mm|Preciva, Shenzhen, China), and masses were measured using a precision scale (AT400: Linearity ±0.5 mg |Mettler-Toledo, Gießen, Hesse, Germany). To increase the accuracy of the force measurement, an additional 200 N load cell (S40S-G3-0020: nominal load 20 kg, accuracy class 0.03|Bosche, Damme, Lower Saxony, Germany) was added to the test setup to augment the precision of the 10 kN range of the load cell in the electromechanical universal testing machine (LFEM 600/100/10|walter+bai, Löhnringen, Switzerland). Deformations were measured in the test using both conventional linear variable differential transformers (LVDTs) (WA20/W10K|Hottinger Brüel & Kjaer, Darmstadt, Hesse, Germany) and digital image correlation (DIC) (Q400 DCM 12.0|Limess, Krefeld, North Rhine-Westphalia, Germany; Istra4D|Dantec Dynamics, Skovlunde, Hovedstaden, Denmark). We used lenses (VS-5018H1|VS Technology, Tokyo, Japan) with a 50 mm focal length and 5 mm spacer rings for the tensile tests, while spacers with a height of 5 + 10 mm were used for the bending and compression tests. The setup consisted of two 12 MP sensors that had pixel densities of about 100 and 50 Px/mm. The sampling rate was 4 Hz. Two LED area lights (T120|Shenzhen Neewer Technology, Shenzhen, China) with a color temperature of 5600 K were used for illumination. A surface pattern was applied to the specimen and the experimental equipment. Preliminary tests revealed that applying opaque white primer (Eberhard Faber, Stein, Bavaria, Germany) followed by a sprinkle of black toner dust (2220D|Ricoh, Tokyo, Japan) resulted in effective speckle patterns on biological tissues that could be used for measurements with minimal impact on the results. Weatherproof labels (L4775|Avery Zweckform, Oberlaindern, Bavaria, Germany) with a speckle pattern printed on them were used on the experimental setup. The optical and conventional measurements were initially synchronized using a simultaneous start that was triggered by an electrical signal.

For biological tissue testing, it is important to establish the strain rate at a level that is physiologically reasonable to help eliminate most of the varying viscoelastic effects [26,27,28,29,30]. A constant strain rate of $\dot{\varepsilon }\;=\;0.005/s^{-1}$ was adopted for all of the material tests on bone tissue. When testing soft tissues, the speed was doubled ($\dot{\varepsilon }\;=\;0.010/s^{-1}$) because of the higher limiting strains compared with osseous material, which would result in long testing times. Long testing would negatively impact the condition of the specimens due to drying and decay-related processes. Given the strain rates $\dot{\varepsilon }$, it is recommended to calculate the displacement velocity $\dot{w}$ while considering the specimen’s geometry to ensure comparable results. For axial compression and tensile testing, the displacement velocity $\dot{w}$ can be estimated using the following equation:

(1) $$\dot{w}\;=\;\dot{\varepsilon }\;\cdot \;l$$

where *l* is the test length. For the three-point bending test, the displacement velocity can be calculated using Bernoulli’s flexural theory

(2) $$\dot{w}\;=\;\frac{\dot{\varepsilon }\;\cdot \;l^{2}}{6\;\cdot \;t}$$

where $\dot{\varepsilon }$ is the strain rate of the outer fiber, *l* is the span, and *t* is the thickness of the cortical beam in the loading direction.

During axial tensile tests of soft tissues, it is crucial to adhere to a rigorous loading regime. As indicated in prior studies [25,31,32,33], soft tissue should be cyclically preloaded to facilitate fiber alignment. Doing so mirrors the natural strain state and enables a more precise evaluation of the material properties. The goal was to apply 10 cycles of cyclic preloading with 10 to 30% of the estimated maximum load. However, we observed that control was not consistently achievable for estimated maximum loads under 100 N during preliminary tests. Therefore, no cyclic preload was applied in those instances. Section 3.3 presents values that could serve as references for estimated maximum loads in future investigations.

### 2.3. Data Evaluation

A highly automated evaluation scheme was implemented to ensure that every specimen was analyzed using the same standardized workflow to enhance comparability and reproducibility. Key properties that characterize the elastic and plastic material behavior for numerical simulations were determined. In particular, the elastic modulus *E* is regarded as a critical property. The strengths *f*, corresponding strains ε, and strain energy densities *U* were derived at defined evaluation points on the stress–strain curve: the point of the yield strength $X_{y}$, ultimate load $X_{u}$ and breaking load $X_{b}$. Yield strength was measured at 0.2% plastic strain or at a local maximum of the curve, and the displacement measurement method was specified as either conventional ($X_{con}$) or optical ($X_{opt}$).

To ensure a fully standardized evaluation for each specimen, the process was automated using custom Python routines (Python v3.7.10|https://www.python.org, accessed on 8 August 2025). These routines were designed to record all relevant information for every sample, including the cadaver specimen’s ID, the harvesting location, geometry data, and test conditions. The evaluation software makes extensive use of modules for data processing (Numpy v1.20.3|https://numpy.org, accessed on 8 August 2025, Scipy v1.6.2|https://scipy.org, accessed on 8 August 2025, Pandas v1.3.0.|https://pandas.pydata.org, accessed on 8 August 2025). Matplotlib (Matplotlib v3.3.4|https://matplotlib.org, accessed on 8 August 2025) and Seaborn (Seaborn v0.11.1|https://seaborn.pydata.org, accessed on 8 August 2025) were used for statistical data visualization.

The process begins with importing protocol data comprising the specimen key, geometry, and assessment codes, as well as establishing measurement-file paths and user options. Subsequently, relevant cross-sectional values are determined based on the test type, and the measurement data are read in. When optical data are available, they are synchronized with conventional measurements by identifying the onset of increased displacement. Conventional displacement data acquired at 100 Hz are downsampled and smoothed using a moving average to 4 Hz when optical data are available, and to 10 Hz otherwise. Notably, the measured force values remain unaltered except for a correction to compensate for the LVDT probe’s spring force (with a constant stiffness of 0.116 N mm^−1^ according to the manufacturer).

To identify the quasi-linear part of the stress–strain curve, a modification of Keuerleber’s approach [34] proved to be more effective than selecting a fixed range of stress or strain. This may be explained by the large variance of the curves measured in the biomechanical tests. For the experiments, the maximum difference quotient of the stress–strain curve was determined and the limits of the linear range were set to 75% of this value. The elastic modulus was then determined based on the data measured within the identified quasi-elastic range. The initial sections of the stress–strain curves were then linearized by extending the Keuerleber range [34] from its lower limit to zero stress, which eliminated falsifying influences from the initial range of the tests. This influence concerns the determined strain at maximum stress as well as the obtained strain energy density. The measured force and thus the stress value remained unchanged.

The investigated beam-like specimens of pelvic cortical bone are typically characterized by varying cross-sectional dimensions along the beam axis. Therefore, the evaluation of the three-point bending test is a special case that requires the acquisition of optically measured data. A detailed discussion of this issue and proposed methods for evaluating such data are available, see Gebhardt et al. [35]. The D2Mgwt evaluation method used to determine the elastic modulus is the preferred one according to Gebhardt et al. [35] (the elastic modulus is referred to as $E_{opt}$ in our method). The D2Mgwt method is based on the weighed fitting of an analytically determined elastic curve of a beam with non-constant cross-sectional dimensions to the deflection of the tested beam measured along its span. Distorting influences such as support indentation and shear deformation are eliminated.

The evaluation code and example datasets are provided in Supplementary Materials (File S3), while representative measured curves are shown in Supplementary Materials (File S4). The derived material properties and relevant specimen information are available in detail in Supplementary Materials (File S5), and the proposed structured database is illustrated in Supplementary Materials (File S1).

### 2.4. Statistical Methods

The requirements for parametric tests, such as normally distributed data or a dataset of sufficient size, are not necessarily satisfied. Accordingly, non-parametric methods were applied. Significance was determined at the conventional level of 5%, and 95% confidence intervals (CIs) were computed by bootstrapping to quantify the variance.

Comparisons among tissue types were evaluated using the Kruskal–Wallis H test in combination with Dunn’s post hoc test for pairwise comparisons. In this analysis, measured material properties were compared across tissue types. Repeatability was assessed through paired comparisons using the Wilcoxon signed-rank test, while comparisons among specimens from different donors or harvesting locations were conducted using the Mann–Whitney U test. Associations between continuous specimen characteristics and the measured material properties were examined using the Spearman rank correlation coefficient. Collectively, these non-parametric methods provide a reproducible and standardized framework for evaluating biomechanical test outcomes.

## 3. Results

A total of 372 specimens were harvested from human lumbopelvic systems according to the previously established standardized protocol [24]. Cortical bone specimens were obtained at seven distinct harvesting locations (Table 1 and Table 2): the ala ossis ilii inferior (AOIlI), ala ossis ilii superior (AOIlS), corpus ossis ilii (COIl), corpus ossis ischii (COIs), corpus vertebrae lumbales (CVLu), ramus ossis ischii (ROIs) and ramus superior ossis pubis (ROPu).

Trabecular bone specimens were collected at five locations and differentiated according to testing direction. These locations included the ala ossis ilii inferior (AOIlI), corpus ossis ilii (COIl), corpus ossis ischii (COIs), corpus vertebrae lumbales (CVLu) and corpus vertebrae sacrales (CVSa). For example, at the (AOIlI), trabecular bone was tested in the *x*-, *y*-, and *z*-directions, yielding 16, 16, and 14 specimens, respectively.

Soft tissue specimens were harvested from 14 distinct locations: The fascia crescent (FCre), fascia endopelvina (FEnP), fascia glutea (FGlu), fascia thoracolumbalis lamina profunda (FTLp), fascia thoracolumbalis lamina superficalis (FTLs), ligamenta sacroiliaca anteriora (SaIla), ligamenta sacroiliaca posteriora (SaIlp), ligamentum iliolumbale (IlLu), ligamentum inguinale (Ingu), ligamentum pectineum (Pect), ligamentum sacroiliacum posterior longum (SaIll), ligamentum sacrospinale (SaSp), ligamentum sacrotuberale (SaTu), and membrana obturatoria (MObt).

### 3.1. Cortical Bone

A total of 138 out of the 160 theoretically possible cortical specimens were successfully evaluated. There were 15 samples that were lost, which were primarily due to harvesting failures (assessment code B01 in Supplementary Materials (File S1)), damage during harvesting (B02), anatomical abnormalities (A00), geometric conditions (A07), or non-compliance with the harvesting protocol (B16). These issues have been documented in a previous publication [24] and in Supplementary Materials (File S4). Additionally, seven specimens were excluded due to unspecific failure during testing (D02) and dislocation (D03) or to a combination of strong individual influences (G02), such as significant geometric deviation.

According to the material properties determined for cortical bone (Table 3), the harvesting location had the most significant influence on the results. H-tests revealed significant differences, which indicate that the regional samples did not belong to a common population. However, it was not feasible to summarize the harvesting regions further in a meaningful way. There was no significant influence concerning proximal and distal sites based on the Wilcoxon signed-rank test (e.g., $E_{opt}$: N = 49, p = 0.337). This lack of influence was also observed for unrelated Mann-Whitney U tests (e.g., $E_{opt}$: N=67, p=0.189) except in the case of the energy density until fracture ($U_{b,opt}$: N = 67, p = 0.016).

**Figure 2:**
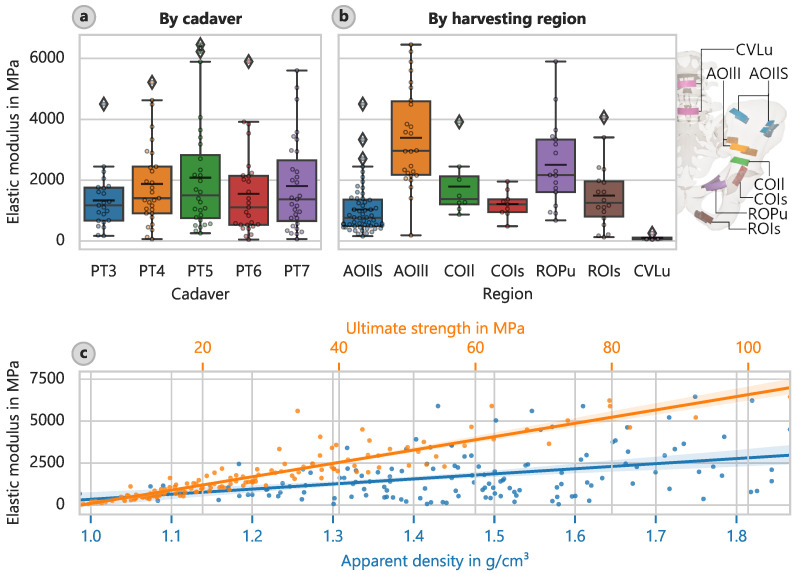
Cortical bone elastic modulus. (**a**) Elastic modulus for different cadavers. (**b**) Elastic modulus assigned to harvesting region, with locations indicated. (**c**) Elastic modulus versus ultimate strength and apparent density with overlaid linear regressions.

The lower part of the pelvic wing (AOIlI) showed the highest elastic modulus of approximately 3390 MPa (CI: 2810 MPa to 4020 MPa) (Figure 2b), while the lumbar vertebrae (CVLu) had the lowest at 109 MPa (CI: 53 MPa to 208 MPa). This corresponds well with the ratio derived from the 4890 MPa found by Kuhn et al. [36] for cortical bone of the iliac crest.

The strength determinations yielded similar results. The cortical bone of the AOIlI had a mean ultimate strength of 49.4 MPa (CI: 41.1 MPa to 59.1 MPa), and the cortical bone of the CVLu had a significantly lower average of only 3.61 MPa (CI: 2.23 MPa to 5.68 MPa).

A significant correlation between the strength and modulus of elasticity was found (Figure 2c). The corresponding equation for the optically determined elastic modulus is as follows:

(3) $$E_{opt}\;=\;67.28\;\cdot \;f_{u}-149.6$$

or

(4) $$E_{opt}\;=\;53.44\;\cdot \;{f_{u}}^{1.045}$$

Both of these equations have a coefficient of determination of about 84%.

We also observed a correlation between the apparent density and elastic modulus (Figure 2c). The corresponding equation for the optically determined elastic modulus is as follows:

(5) $$E_{opt}\;=\;3032\;\cdot \;\rho_{app}-2689$$

or

(6) $$E_{opt}\;=\;633.3\;\cdot \;{\rho_{app}}^{2.582}$$

where $\rho_{app}$ is in g cm^−3^ This equation is considered valid given its coefficient of determination of about 15%. The correlation between strength and apparent density can be described as follows:

(7) $$f_{u}\;=\;44.84\;\cdot \;\rho_{app}-37.42$$

or

(8) $$f_{u}\;=\;11.34\;\cdot \;{\rho_{app}}^{2.330}$$

for which $R^{2}\;=\;$ 18%.

When we examined the relationship between mechanical data and donor data, only age exhibited a significant influence. The samples’ apparent densities suggest that they did not derive from a common population. However, post-hoc multiple comparisons revealed that this relationship was not true for all of the cadavers individually. We cannot rule out that the apparent densities of three of the cadavers (ages: 85, 86, and 89) were derived from one common population and that the other two cadavers (ages: 53 and 71) were derived from another. Even so, a trend is perceptible:

(9) $$\rho_{app}\;=\;-0.003934\;\cdot \;\mathrm{age}+1.766$$

where age in years, and $R^{2}\;=\;$ 8.3%. There was a relatively high coefficient of determination for the regression line, considering the small group size of five donors.

### 3.2. Trabecular Bone

Out of the 165 theoretically possible trabecular bone specimens, 146 were included in the evaluation. The term trabecular bone is used synonymously with “spongy (cancellous) bone”. The excluded specimens primarily involved harvesting failures (B01), anatomical abnormalities (A00), or non-compliance with the cutting protocol (B16). Optical measurements were found to be unreliable for compression testing of trabecular bone, and the measurement pattern often failed early in the test due to fluid leakage. Supplementary Materials (File S4) provides an example of such a failure. We were able to determine the general failure mode of undefatted trabecular bone. The failure was due to the collapse of individual trabecular structures in an early stage of the testing sequence (i.e., at a lower load level). Hardening occurred after the stress built up to a maximum and then fell to a local minimum.

The most important results from Table 4 pertain to the elastic modulus $E_{con}$ (average: 32.7 MPa) and ultimate compressive strength $f_{u}$ (average: 1.26 MPa). Just as in the case of cortical bone, the harvesting location of the trabecular bone had the strongest effect on the material properties.

Apart from the strain at the ultimate strength and the fracture strength, the H-tests revealed significant differences, suggesting that the samples were unlikely to have been derived from a common population. As with the cortical bone samples, Dunn’s post-hoc tests did not allow us to reliably group the samples into subordinate regions.

**Figure 3:**
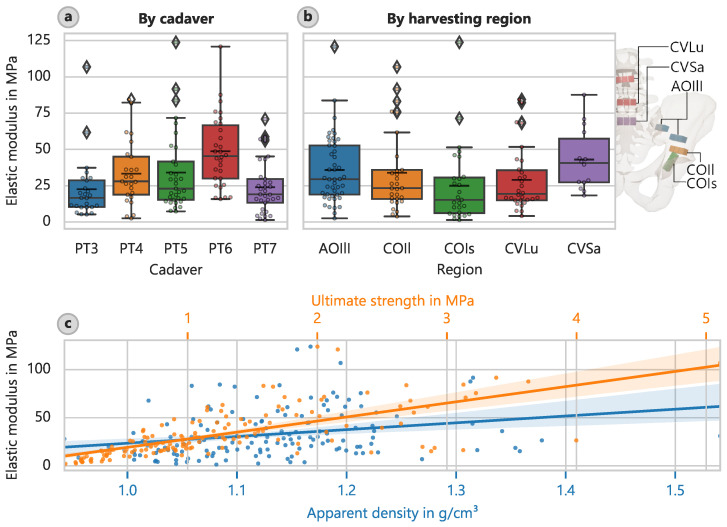
Trabecular bone elastic modulus. (**a**) Elastic modulus for different cadavers. (**b**) elastic modulus assigned to harvesting location, with locations indicated (**c**) elastic modulus versus ultimate strength and apparent density with overlaid linear regressions.

In relation to the different harvesting locations, the largest value of corresponding elastic moduli was approximately 43.0 MPa (CI: 32.5 MPa to 54.5 MPa) and was found in the sacrum region (CVSa) (Figure 3b). The lowest value of 24.9 MPa (CI: 16.5 MPa to 35.7 MPa) was observed in the acetabular region towards the ischium (COIs). The corresponding mean ultimate strengths were 2.048 MPa (CI: 1.610 MPa to 2.490 MPa) and 0.878 MPa (CI: 0.608 MPa to 1.234 MPa), respectively.

**Figure 4:**
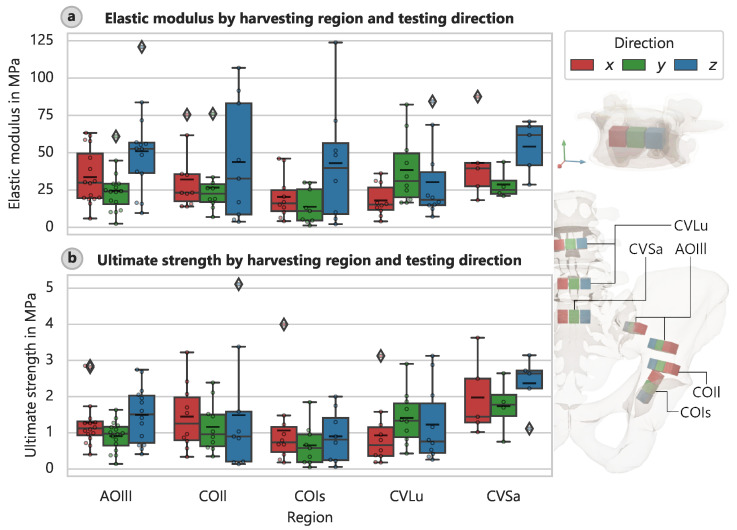
Trabecular bone direction. Elastic modulus and ultimate strength according to harvesting location and local testing direction. (**a**) Elastic modulus. (**b**) Ultimate strength.

There were also variations in stiffness and strength according to the specimen orientation during the harvesting process and thus the loading direction during testing. The elastic modulus varied significantly with the direction of the applied load (Figure 4a), and a similar pattern was observed for other specimens, for which the strengths depended on direction (Figure 4b).

The correlation analysis revealed the most robust link between strength and the elastic modulus (Figure 3c). The best-fit relationship describing the elastic modulus is as follows:

(10) $$E_{con}\;=\;18.65\;\cdot \;f_{u}\;+\;9.262$$

The coefficient of determination for this equation was 47.1%. Applying a power function leads to a slightly better fit:

(11) $$E_{con}\;=\;29.94\;\cdot \;{f_{u}}^{0.6819}$$

$R^{2}$ was 49.8% for this equation.

In addition, the apparent density strongly influenced the determined elastic modulus (Figure 3c), but not as strongly as for cortical bone. The linear relationship is as follows:

(12) $$E_{con}\;=\;70.71\;\cdot \;\rho_{app}-47.20$$

where $R^{2}\;=\;$ 6.9%. The power law equation is as follows:

(13) $$E_{con}\;=\;25.62\;\cdot \;{\rho_{app}}^{1.973}$$

for which $R^{2}$ was 6.1%. The correlations between strength and apparent density for cancellous bone are as follows:

(14) $$f_{u}\;=\;5.272\;\cdot \;\rho_{app}-4.701$$

for which $R^{2}=$ 28.4% and

(15) $$f_{u}\;=\;0.8057\;\cdot \;{\rho_{app}}^{3.503}$$

for which $R^{2}$ is 24.6%, respectively.

Further comparison of the cadaver data revealed that the age of the donor had a significant influence on the results. H-tests excluded the possibility of derivation from a common population with statistical significance, except in the case of the strain at the ultimate strength and fracture strength. However, the post-hoc multiple comparisons revealed that this situation did not apply to all of the cadavers individually. Like for cortical bone, a subordinate group assignment for the elastic modulus and strength did not yield representative results. In terms of the samples’ apparent density, we could not significantly rule out that two of the cadavers (donor ages: 85 and 86 years) belonged to one population, two others (donor ages: 53 and 89 years) belonged to another, and one (donor age: 71 years) belonged to its own unique population. Even so, we were able to extract trends. The coefficient of determination for the regression line was relatively high, given the small group size of five cadavers:

(16) $$\rho_{app}\;=\;-0.002151\;\cdot \;\mathrm{age}+1.297$$

for which $R^{2}\;=\;$ 9.1%.

### 3.3. Soft Tissue

Out of the 130 theoretically possible soft tissue specimens, 88 were included in our evaluation. The evaluation was challenging due to the issue of optimizing harvesting locations (this issue was not present for the bony samples). The primary reasons for excluding soft tissue specimens were: harvesting failures (B01), anatomical abnormalities (A00), and non-compliance with the harvesting protocol (B16). As specified previously in [24], and in accordance with the Terminologica Anatomica [37], classification was performed based on planar connective tissue structures called fascia and band-like structures called ligaments. This also corresponds more closely to the structural implementation differentiation in FE simulations.

The optical measurements did not yield reliable results for the soft tissue tests. This was partly due to the significant fluid leakage that occurred during the testing and disrupted the applied speckle pattern. Such leakage was particularly pronounced for thicker ligamentous test subjects. Additionally, we observed detachment of the outer layer of the samples, which primarily affected the fascial specimens. Since the measurement pattern was applied to the outer layer and thus the displacements measured at this layer, the inner fibers were not necessarily examined.

**Figure 5:**
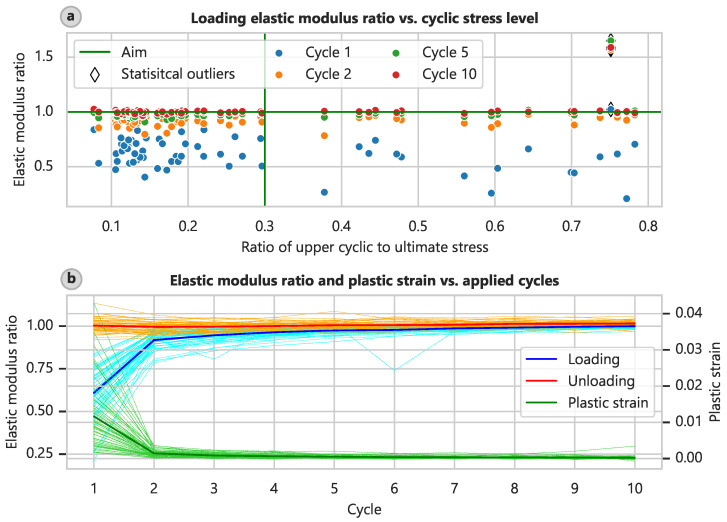
Soft tissue cyclic prestressing. Ratio of cyclic to final elastic modulus in relation to the pre-stress level and the number of applied cycles. (**a**) Upper cyclic stress level. (**b**) Elastic modulus and plastic strain in relation to the number of applied cycles with statistical outliers excluded (applying the 1.5-interquartile-range rule).

The standard test procedure for soft tissues involves cyclic preloading, as detailed in Section 2.2. We assessed the success of this procedure. Due to the aforementioned control issue, only 60 specimens could be cyclically preloaded. On average, a preload level of 8.61% (CI: 6.62% to 10.65%) and a cyclic level of 28.69% (CI: 23.38% to 34.01%) of the ultimate stress were achieved, and the target levels were 10 and 30%, respectively. The elastic modulus determined at the loading part of the test curve increased with the cycle number compared with the elastic modulus determined in the final part of the test (Figure 5a), and this trend was relatively independent of the actual loading level. This relationship is also depicted in plots of the ratios of the elastic modulus and plastic strain relative to the test cycle (Figure 5b).

By the fifth cycle, the elastic moduli of the loading branch had already attained 98.6% (CI: 97.1% to 101.1%) of the final elastic modulus, and the plastic strain was 5.52% (CI: 4.60% to 6.64%) of the value obtained after the first cycle. By the eighth cycle, these measurements had reached 100.2% (CI: 98.9% to 102.4%) and 3.52% (CI: 2.61% to 4.47%), respectively. Wilcoxon tests showed no significant difference between the elastic moduli in the ninth and tenth loading cycles in comparison to the final one (p = 0.158 and 0.556).

Interestingly, the elastic moduli determined at the unloading branches of the test curve remained relatively constant at 102.4% (CI: 101.7% to 103.3%) of the final elastic modulus. This finding might indicate that specimen slippage occurred during testing or a general difference in compression-tension behavior. However, slippage was not evident on the clamping surfaces after the removal of the samples, and the surfaces exhibited clear imprints of the clamping device’s pyramid shapes. Supplementary Materials (File S4) shows example images of the clamping surfaces.

A summary of the identified material properties and relevant geometric values of the soft tissues is only meaningful when we distinguish between planar (fascial) and band-like (ligamentous) connective tissue structures. Our evaluation also revealed that the determination of specimen size using calipers was prone to error.

The mean elastic modulus $E_{con}$ for the fascial specimens was determined to be 148 MPa, and the ultimate strength $f_{u}$ was determined to be 14.3 MPa (Table 5). For the ligamentous specimens, the mean elastic modulus of 103 MPa and the mean strength of 10.7 MPa were not markedly different from those of the fasciae (Table 6). Supplementary Materials (File S5) provides additional properties (e.g., spring stiffness) for the band-like ligaments.

Given the large variance in the material properties and the relatively small sample size of 88 specimens, the recovered statistical correlations are somewhat unreliable. However, for completeness, the results are included without separating fascia and ligament tissue. For the connective tissue specimens, the harvesting location had the strongest influence on the determined material properties. The H-tests indicated a very low likelihood that the specimens derived from a common base population. However, it was not feasible to consolidate these harvesting locations further in a meaningful way.

**Figure 6:**
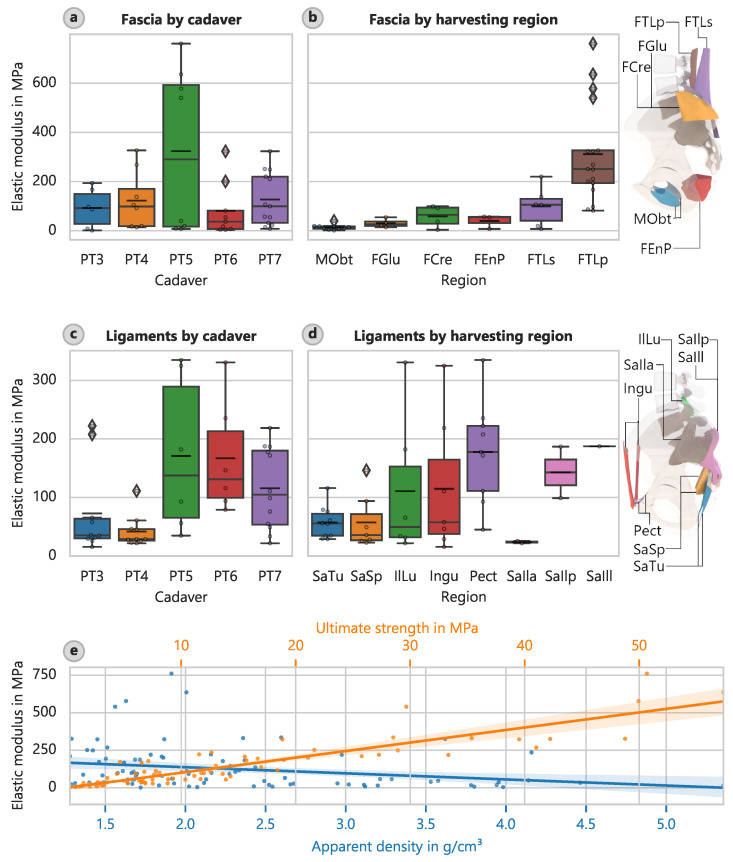
Soft tissue elastic modulus. (**a**) Elastic modulus for different cadavers for the fascial specimens. (**b**) Elastic modulus assigned to harvesting location for the fascial specimens, with locations indicated. (**c**) Elastic modulus for different cadavers for the ligamental specimens. (**d**) Elastic modulus assigned to harvesting location for the ligamental specimens, with locations indicated. (**e**) Elastic modulus versus ultimate strength and apparent density with overlaid linear regressions for fascial and ligamental specimens.

The largest elastic modulus of the fascia samples occurred in the deep layer of the dorsal fascia in the lumbar region (FTLp) with a value of 311.4 MPa (CI: 222.7 MPa to 404.3 MPa) (Figure 6). The lowest elastic modulus of 13.98 MPa (CI: 8.58 MPa to 21.42 MPa) occurred in the foramen obturatoria (MObt). Similar patterns occurred for the determined strengths, with corresponding mean ultimate strengths of 28.46 MPa (CI: 21.29 MPa to 35.95 MPa) and 2.23 MPa (CI: 1.31 MPa to 3.45 MPa).

Considering only the regions that yielded more than three samples, the largest average elastic modulus of the ligaments occurred for the pectineal ligament or Cooper’s ligament (Pect) at 177.5 MPa (CI: 127.2 MPa to 232.9 MPa) (Figure 6d). The least stiff region that also yielded more than three samples was the sacrotuberous ligament (SaTu) with an elastic modulus of 56.9 MPa (CI: 41.2 MPa to 74.2 MPa). However, the determined strengths did not follow the same pattern. The inguinal ligament (Ingu) had the highest strength of 16.43 MPa (CI: 6.61 MPa to 27.15 MPa), and the sacrospinous ligament (SaSp) had the lowest strength of 6.10 MPa (CI: 4.06 MPa to 8.69 MPa).

The strongest correlation occurred between the elastic modulus and ultimate strength (Figure 6e). The relationship can be described by the following:

(17) $$E_{con}\;=\;10.04\;\cdot \;f_{u}-0.3072$$

or

(18) $$E_{con}\;=\;9.143\;\cdot \;{f_{u}}^{1.027}$$

The coefficient of determination in this case was 81%. The elastic modulus correlated with apparent density (Figure 6e) and decreased as the density increased. The relationship can be described as follows:

(19) $$E_{con}\;=\;-40.68\;\cdot \;\rho_{app}\;+\;218.1$$

However, this relationship is not very meaningful given its coefficient of determination of 6.0%. We obtained a similar relationship between the strength and apparent density, with $R^{2}\;=\;$ 5.7%:

(20) $$f_{u}\;=\;-3.541\;\cdot \;\rho_{app}\;+\;20.59$$

## 4. Discussion

The standardization of the proposed testing methods is a key aspect of this study. We aimed to provide a robust framework that ensures consistent results across different laboratories by implementing standardized specimen preparation, testing protocols, and boundary conditions, as well as incorporating detailed documentation (main objective). While inter-laboratory validation is necessary to evaluate reproducibility, our methodology has demonstrated high repeatability within our own laboratory. The methodology’s simplicity allows for widespread adoption in biomechanical laboratories without requiring highly specialized equipment. Furthermore, the use of clearly defined material properties and the implementation of automated data evaluation routines minimize variability arising from subjective interpretation. The procedure design builds on previous work addressing the standardization of specimen preparation and harvesting [24] and allows all steps to be completed within five working days while preserving specimen integrity. This streamlined workflow can be carried out by a team of two individuals, which minimizes personnel requirements and ensures consistent results. Although the reproducibility cannot be demonstrated directly, due to the destructive nature of the testing, the methods developed and the quantified repeatability provide a robust framework that lends to inter-laboratory reproducibility.

In the following, the most important quantitative results of our investigations are discussed (secondary objectives) and compared with values found in the literature. The biomechanical testing revealed specific material properties for both bone and soft tissue. Cortical bone demonstrated an average elastic modulus of 1750 MPa and an ultimate strength of 28.2 MPa, while trabecular bone showed values of 32.7 MPa and 1.26 MPa, respectively. For fascial tissue, the average elastic modulus was 148 MPa, and the average ultimate strength was 14.3 MPa, which contrasts with values of 103 MPa and 10.7 MPa for ligamentous tissue. These results highlight the significant spatial variability and dependency of material properties on tissue type and harvesting location. They also provide a critical foundation for advancing mechanical simulations and improving implant development. Of particular note is the optically determined elastic modulus $E_{opt}$ for cortical bone, which was 1750 MPa on average. This value is nearly twice as high as the conventionally determined $E_{con}$ (method A0AL based on the midspan deflection measurement using LVDTs [35]). This discrepancy can be attributed to the elimination of falsifying influences (i.e., support indentation and shear deformation) and the global determination approach (outlined in Section 2.3). Comparable data for the pelvic cortical bone is sparse. Wirtz et al. [38] examined human long bones (cortical femoral bone with low apparent densities of 1.5 g cm^−3^) in the axial and transversal directions. The stiffness values obtained were 2.5 and 4.1 times higher than our results, and the strength values were 2.4 and 5.5 times higher. These differences can be explained by the distinct load-bearing behavior of the pelvis compared with long bones, such as the femur.

The results in Section 3.2 indicate that the general failure mode of undefatted trabecular bone involves the early collapse of individual trabecular structures, which occurs at relatively low loads. Given the persistence of this type of failure throughout the test, it might be reasonable to categorize this behavior as a quasi-elastic rather than linear-elastic. The post-hardening observed could potentially be due to the buildup of internal pressure in sealed chambers, as indicated by the observed leakage of fluid.

Wirtz et al. [38] also reported values for the elastic modulus $E_{con}$ and ultimate compressive strength $f_{u}$ for cancellous bone. However, they used different methods for measuring material density, so comparison with our values requires some assumptions about this property. Wirtz et al. [38] noted studies in which the apparent density was calculated as the mass of the mineralized bone divided by its total volume. However, we based the apparent density on the sample’s total mass, including the bone marrow. Using a density of 0.4 g cm^−3^ for trabecular bone, the elastic modulus of femoral bone is 7 to 13 times higher than our measured values and the compressive strength 5 to 6 times higher. These findings are consistent with those of Dalstra et al. [13], who found, that the mineralized content of trabecular pelvic bone accounts for less than 20% of its volume. They stated that this value corresponds to vertebral bone rather than femoral or tibial bone. The determined orthotropic elastic moduli of 59.8, 50.1, and 38.3 MPa with 95% CI for 33 specimens from the human pelvis in the three principal directions. These values are slightly higher than those in the present study. Van Ladesteijn [39] determined a peak modulus of 15.1 MPa and a yield strength of 0.426 MPa for cancellous bone from the acetabular region, whereas we determined a yield strength of 0.786 MPa (CI: 0.519 MPa to 1.130 MPa) for the COIs region. Our values are higher by about 65% for the elastic modulus and 85% for yield strength. The mean resilience (energy to yield) was 24.97 kJ m^−3^ (CI: 8.73 kJ m^−3^ to 51.22 kJ m^−3^), which is about four times larger than that of van Ladesteijn et al. [39] (5.71 kJ m^−3^) for the same region. Notably, Thompson et al. [40] reported a Young’s modulus of 116.4 MPa for the acetabular region and 47.4 MPa for the femoral head. The value for the acetabular region does not match our results or those of van Ladesteijn et al. [39]. It is challenging to interpret the results in terms of variations in stiffness and strength according to the orientation of the specimen without examining the principal stress directions in more detail. Additional investigations, such as FE simulations, are necessary. Given that each specimen’s position, direction, and geometry are well defined, such investigations should be feasible. Nevertheless, we can already draw some conservative conclusions. For instance, the average elastic modulus of the CVLu specimens in the y-direction (headward) was larger (38.41 MPa, CI: 25.90 MPa to 52.99 MPa) than in the x- and z-directions (lateral and ventral) (18.06 MPa, CI: 12.24 MPa to 24.51 MPa and 30.36 MPa, CI: 16.37 MPa to 46.96 MPa). This finding suggests that the stiffness of cancellous bone is larger in the primary direction of load transfer (cranial along the spine). Further supporting evidence of this assertion is the distribution of the elastic moduli of the CVSa, which peaks in the direction of the sacroiliac joint (the local z-direction).

Due to significant fluid leakage, which notably disrupted the applied speckle pattern in thicker ligamentous test objects, optical measurements in soft tissue tests did not yield reliable results. This issue might be mitigated by sealing the samples with a polymer such as polyethylene glycol, as suggested by Lozano et al. [41], or a more water-resistant primer. However, it is likely that this sealing affected the material properties of the samples, which applies, in particular, to thin specimens. A noteworthy observation was that the outer layer detached, particularly in fascial specimens, thereby confining the measurement pattern to that superficial region and potentially obscuring the behavior of the inner fibers. It might be beneficial for future investigations to remove this specific layer prior to testing.

The elastic modulus measured during loading increased with cycle number relative to that in the final phase, which was largely independent of the loading level. This observation aligns with our initial assumption that cyclic preloading leads to collagen fiber alignment and thus stiffening.

The values presented in Table 3 and Table 4 show that the fascial specimens had a mean elastic modulus $E_{con}$ of 148 MPa and an ultimate strength $f_{u}$ of 14.3 MPa, while the ligamentous specimens had a mean elastic modulus of 103 MPa and a mean strength of 10.7 MPa, which are markedly different from those of the fasciae. Supplementary Materials (File S5) provides properties (e.g., spring stiffness) for the band-like ligaments. These data could prove useful for numerical simulations. For example, the average spring stiffness of the ligamentous structures was 89.1 N mm^−1^ (CI: 75.2 N mm^−1^ to 105.1 N mm^−1^).

The data from this study are presented in detail in Supplementary Materials (File S5) to systematically distinguish between variations caused by differences in harvesting locations and inter-individual variability. These datasets provide transparency in regard to the contributions of anatomical harvesting regions versus biological factors to the variability in experimental results. The proposed methodology minimizes procedural variability and lays the groundwork for reproducible and comparable biomechanical measurements. However, the inherent differences in human anatomy must be acknowledged as these reflect natural variability that cannot be addressed by methodological approaches alone.

Instead, such variability requires large datasets that have been obtained under consistent protocols to better understand and quantify these differences. By providing a robust framework, this study could facilitate the future collection and interpretation of expanded datasets and improve the reliability of biomechanical research and its applications.

### 4.1. Clinical and Biomechanical Applications

Several potential applications emerge from the location-specific material properties identified in this study. When incorporated into FE models of the lumbopelvic complex, these data may enable more realistic simulations of stress and strain distributions under both physiological and pathological loading conditions, supporting the early identification of mechanically vulnerable areas under conditions such as osteoporosis or sacroiliac-joint insufficiency [42]. By considering anisotropic, region-dependent elastic moduli and strengths, the patient-specific treatment planning already in use could be refined: for example, surgeons might simulate different fixation strategies (plates, screws, sacroiliac stabilizers) more realistically to optimize implant geometry, stiffness grading, and anchorage, thereby minimizing complications and improving the outcome [43,44,45,46].

In rehabilitation settings, known soft-tissue properties can help to optimize the design of therapeutic exercise protocols. Musculoskeletal models could predict ligament and fascial strains during specific movements, allowing clinicians to prescribe exercises that maintain safe loading conditions, accelerate functional recovery, and reduce the risk of re-injury [47]. Finally, coupling a robust experimental material database with imaging-based inverse FEM—following approaches demonstrated by Helgason et al. [10,11] and Taddei et al. [12]—offers a non-invasive pathway to estimate patient-specific material properties, to guide diagnosis and personalized therapy without additional invasive procedures.

### 4.2. Technical Notes

In addition to the described axial test setups (Section 2.2), we also investigated modified versions with fewer constraints applied to the specimens in preliminary tests. The modifications included a ball-and-socket joint at the lower end of the specimens in the compression tests, a universal joint above the tensile test module, and a pivoting support in the bending tests. These modified test setups theoretically lessened the effect of irregular specimen geometries, but they also compromised the robustness of the testing procedures.

We also tested dog-bone shaped specimens for tensile tests of soft tissues using an adapted design of the Print-A-Punch device [48]. On one hand, this shape might have contributed to the prevention of slippage, but on the other hand, it shifted the location of the failure to the center of the specimen. However, the resulting deflection forces might have reduced the validity of the determined elastic material properties. In addition, creating a dog-bone-shaped object is not a viable option for all soft tissues. For example, the sacrotuberous ligament is both twisted and thickened to such an extent that it was not possible to trim it accordingly without removing the relevant main striation fibers. Therefore, the dog-bone shape appears to be useful only for soft tissues with parallel fibers, and in view of the intended standardization of the test procedure, this shape was not pursued further. Moreover, the main fiber direction was not controlled microscopically. Therefore, the mechanical tests were carried out primarily along the macroscopically determinable direction.

One of the goals was to minimize the hurdles of applicability as much as possible; therefore, some limitations have to be accepted. For example, this applies to mechanical testing in a temperature-controlled water bath that provides a constant temperature of 37 °C and prevents drying. In such a case, it can not be ruled out that the water bath would affect the optical measurement (e.g., due to refraction at phase transitions). In the present investigations, drying was counteracted by storing the specimens in special containers with 100% relative humidity and keeping the test duration as short as possible. These measures were backed by the investigations of Gebhardt et al. [49] into the influence of humidity on the elastic behavior of pelvic cortical bone.

Reproducing in vivo conditions for the mechanical tests would require very complex experimental setups. The triaxial testing chamber for cancellous bone used by Rincón-Kohli and Zysset [50] provides an impressive example of such an attempt.

Regarding the problematic determination of the geometry of the soft tissue, a method other than caliper measurement with undefined pressure could be used. Scholze et al. [25] determined the geometry of specimens using molding. Another viable method involves embedding the samples in plastic material, thinly sectioning the resulting body, and then scanning the slices to measure their cross-sectional dimensions. However, these methods are also prone to errors and were not considered to be suitable alternatives. A new method described by Schwarz et al. [51] could probably be adapted for reliable and fast determination of specimen thickness. The technique involves using transverse force loading to measure a pressure-thickness response. The unloaded thickness can be obtained by extrapolating the linear part of the resulting curve towards zero pressure.

In addition to the experimental setups used, we also advocate the use of non-contact optical measurements via DIC. This method enhances measurement precision but does not affect the sample during the mechanical test. Simple opaque white primer and toner dust were used to mark the samples for the optical measurements, but this approach was not sufficiently robust to reliably measure soft tissue and trabecular bone specimens. It mainly failed due to water leakage, which rendered the measurement pattern unsuitable due to reflections or disruption. A reliable evaluation of such specimens would have allowed us to determine Poisson’s ratio and define a three-dimensional elasticity matrix for each region. We selected our procedure to avoid imparting any undue influence on the biological material being examined. In particular, we were concerned about chemical attack, which can occur with paints that contain solvents. Such paints also exhibit increased water resistance as a result. On the other hand, we sought to avoid affecting the specimens’ behavior, such as drying or decay due to long drying times or stiffness increase due to stiff paint layers. However, after the present experiments were designed, Zwirner et al. [52] recently showed that even solvent-based color coatings had no effect on the stiffness of soft tissue.

Zhao et al. [53] reviewed the standardization of compression testing to measure the stiffness of human bone and recommended a similar procedure to the one that we used for specimen preparation and testing. However, their primary focus was the femur and other long bones, and they suggested sampling parallel to the trabecular alignment for both cortical and trabecular bone samples. This procedure is definitely reasonable for these types of bones, but it is not practical for bones with irregular geometries (such as those found on the pelvis). Furthermore, a compression test for cortical bone is not necessarily suitable in all cases [35]. There are special requirements associated with analyzing curved and irregularly shaped specimens, such as pelvic bones, and there are often unintended bending moments during axial testing. Multiaxial states of stress that are difficult to predict can occur due to friction at the end faces of the samples in axial compression tests. Multiaxial stress states can also occur in axial tension tests due to clamping forces.

Following the recommendation of Zhang et al. [54], we determined different yield strength values. This procedure was conducted in addition to the standard method using an offset of 0.2% plastic strain. For all of the materials tested, we adopted the upper limit of the Keuerleber approach [34] discussed in Section 2.3, as well as the intersections determined using 0%, 0.1% and 0.2% strain offset. In addition, we evaluated an offset of 0.007% for the cortical specimens, 0.05% for the trabecular specimens, and 0.5% for the soft tissue specimens. Specimen-specific FE optimization according to the approach by Zhang et al. [54] is still pending. The resulting deviations from the ultimate level are shown in Supplementary Materials (File S4).

### 4.3. Limitations

As mentioned in Section 3.2, we determined the apparent density for trabecular bone by relating the specimen’s mass to its volume. The mass was determined without changing the samples compared with the state of harvesting (i.e., without removing the bone marrow). This makes it difficult to compare our results with other studies, in which the mass of the calcified portion is normally determined [18]. This includes values such as dry, ash or bone mineral density. As described in our previous publication [24], we performed cadaver-specific computer tomography (CT) scans of the complete lumbopelvic complex. By adding a reference phantom, the density distribution of the material may be derived from the retained Houndsfield Units according to Anderson et al. [55]. This procedure allows us to bypass the determination at the sample level using methods such as ashing. The apparent densities of cancellous bone found and the derived correlations still have a lack of comparability to other studies. Yet, a question arises regarding how useful the bone mineral density is in biomechanical simulations, except for the identification of the degree of osteoporosis. In most cases, complex FE simulations are often based on patient-specific CT scans with regard to geometry and density distribution. However, due to their lower resolution, these scans only provide blurred gray values, in contrast to higher-resolution scientific imaging methods such as micro-computed tomography (µCT) scans. It is not possible to separate the materials of spongy bone (trabecular bone and marrow), so only a combined assignment of material properties can be performed directly.

Furthermore, cadaver-related factors may have an influence on location-specific material data. All specimens were obtained from five elderly cadavers (average age: 77.3 years, range: 53.2 to 89.2 years) with an unbalanced sex distribution (three females, two males). Age-related bone demineralization and subclinical osteoporosis or other metabolic bone diseases cannot be fully excluded and may have reduced apparent densities and mechanical strengths in both cortical and trabecular bone. Indeed, we observed a modest negative correlation between apparent density and age for cortical ($R^{2}\;=\;0.083$) and trabecular bone ($R^{2}\;=\;0.091$). The small cadaver cohort and skewed sex ratio preclude definitive conclusions on sex-specific differences. Future expansions of the database should include a broader age range, balanced sex representation and clinical assessments of bone quality to improve generalizability.

The determination of specimen size using calipers was also prone to errors, as uneven contraction force could have resulted in significant deviations, particularly when measuring thickness. This issue might have impacted the determination of material properties, which rely on having accurate cross-sectional dimensions.

## 5. Conclusions

Uncertainties inevitably arise from the natural variation and handling of donor material. This study could help to improve comparability and obtain more reliable data by making the underlying methods easily applicable, comparable, and repeatable. Our methods, code, results, and recommendations are fully accessible in the Supplementary Materials.

The material properties identified by the proposed methods for the lumbopelvic complex differ significantly from those of long bones. Our findings emphasize location and density dependencies and provide a solid basis for studying load transfer in the lumbopelvic system. They could also enhance the accuracy in treatment planning and implant development by better representing the tissue properties observed in cadaver studies. Notably, while a stiffness of 17 GPa was previously assumed for cortical bone [23,55,56,57], our results suggest a value that is approximately 10 times lower for the pelvis.

Future research could enhance material data for the lumbopelvic system by considering different loading directions and identifying orthotropic elasticity matrices. Structural tensors from µCT [45] or principal stress trajectories derived from FE simulations could guide these efforts. 

## Figures and Tables

**Table 1:** Bone specimen distribution according to harvesting location.

Location	Cortical Bone Count	Trabecular Bone Count		
		*x*	*y*	*z*
AOIII	26	16	16	14
AOIIS	56	—	—	—
COII	8	10	10	9
COIs	9	10	9	8
CVLu	4	10	10	10
ROIs	18	—	—	—
ROPu	17	—	—	—
CVSa	—	5	4	5

Abbreviations are explained at the end of the text in the Section Abbreviations.

**Table 2:** Soft tissue specimen distribution according to harvesting location.

Location	FCre	FEnP	FGlu	FTLp	FTLs	SaIla	SaIlp	IlLu	Ingu	Pect	SaIll	SaSp	SaTu	MObt
Count	4	3	4	17	6	2	2	6	7	9	1	7	10	10

Abbreviations are explained at the end of the text in the Section Abbreviations.

**Table 3:** Cortical bone properties (*N* = 138).

Variable	Unit	Mean	Median	Min	Max	CI_Min_	CI_Max_
*Structural properties*							
$t_{mean}$	mm	2.26	2.19	0.94	4.68	2.14	2.39
$w_{mean}$	mm	9.52	9.52	6.83	12.29	9.34	9.70
$A_{CS}$	mm^2^	21.62	20.55	7.84	43.41	20.34	22.86
*V*	mm^3^	76.36	72.92	26.78	165.48	71.70	80.98
$I_{mid}$	mm^4^	12.66	7.45	0.32	86.65	10.22	15.34
*Physical material properties*							
$\rho_{app}$	g cm^−3^	1.46	1.46	0.99	1.87	1.43	1.49
*Mechanical material properties*							
$E_{con}$	MPa	897.89	649.81	28.72	4402.50	766.69	1022.34
$E_{opt}$	MPa	1747.67	1317.88	48.45	6454.97	1510.86	1971.05
$f_{y}$	MPa	22.67	17.56	1.58	93.48	19.84	25.20
$\varepsilon_{y,opt}$	‰	15.51	14.97	4.15	34.39	14.77	16.22
$U_{y,opt}$	kJ m^−3^	167.33	131.86	10.61	682.05	144.98	187.81
$f_{u}$	MPa	28.20	23.00	1.95	106.15	24.84	31.21
$\varepsilon_{u,opt}$	‰	39.73	36.26	6.77	138.31	36.99	42.84
$U_{u,opt}$	kJ m^−3^	722.57	556.38	64.52	6059.98	618.49	838.09
$f_{b}$	MPa	22.95	18.63	1.45	94.72	20.01	25.57
$\varepsilon_{b,opt}$	‰	55.15	52.52	6.77	151.84	51.37	59.35
$U_{b,opt}$	kJ m^−3^	1099.07	819.15	64.52	6786.41	946.56	1260.89

Symbols are explained at the end of the text in the Section Symbols.

**Table 4:** Trabecular bone properties (*N* = 146).

Variable	Unit	Mean	Median	Min	Max	CI_Min_	CI_Max_
*Structural properties*							
$l_{test}$	mm	9.54	9.72	6.80	11.73	9.39	9.69
$A_{CS}$	mm^2^	91.50	93.28	42.10	124.96	89.03	93.66
*V*	mm^3^	87.58	88.42	32.29	123.36	84.85	90.22
*Physical material properties*							
$\rho_{app}$	g cm^−3^	1.13	1.13	0.94	1.54	1.12	1.15
*Mechanical material properties*							
$E_{con}$	MPa	32.69	25.85	1.34	123.83	29.28	36.61
$f_{y}$	MPa	1.10	0.89	0.02	5.11	0.98	1.25
$\varepsilon_{y,con}$	‰	40.02	32.90	17.03	190.73	35.78	44.97
$U_{y,con}$	kJ m^−3^	28.53	16.14	0.23	300.22	21.70	36.94
$f_{u}$	MPa	1.26	1.03	0.05	5.11	1.13	1.41
$\varepsilon_{u,con}$	‰	64.83	51.65	22.78	386.23	58.29	72.38
$U_{u,con}$	kJ m^−3^	55.58	31.03	1.31	687.40	44.05	69.42
$f_{b}$	MPa	1.12	0.91	0.05	3.95	0.99	1.26
$\varepsilon_{b,con}$	‰	83.38	71.37	31.12	386.96	76.29	90.96
$U_{b,con}$	kJ m^−3^	72.90	53.52	1.35	689.21	60.96	87.44

Symbols are explained at the end of the text in the Section Symbols.

**Table 5:** Fascia properties (*N* = 44).

Variable	Unit	Mean	Median	Min	Max	CIMin	CIMax
*Structural properties*							
$A_{CS}$	mm^2^	13.92	11.98	2.31	39.90	11.60	16.61
*V*	mm^3^	65.84	49.27	7.56	194.49	52.25	80.19
*Physical material properties*							
$\rho_{app}$	g cm^−3^	2.28	1.96	1.28	5.36	2.02	2.56
*Mechanical material properties*							
$E_{con}$	MPa	147.79	84.33	1.30	760.81	98.91	207.27
$f_{y}$	MPa	7.81	5.66	0.25	28.04	5.81	10.03
$\varepsilon_{y,con}$	‰	91.02	82.48	29.00	191.36	78.15	104.33
$U_{y,con}$	kJ m^−3^	282.23	201.68	23.94	1516.38	204.28	375.38
$f_{u}$	MPa	14.35	7.55	0.29	57.36	10.10	19.51
$\varepsilon_{u,con}$	‰	179.41	159.18	55.93	473.66	155.70	210.55
$U_{u,con}$	kJ m^−3^	1437.69	664.74	54.30	6440.13	998.46	1994.20
$f_{b}$	MPa	14.04	7.31	0.28	56.15	9.94	19.15
$\varepsilon_{b,con}$	‰	188.37	168.67	68.94	477.71	165.31	219.21
$U_{b,con}$	kJ m^−3^	1586.78	714.64	57.40	7073.87	1095.65	2199.46

Symbols are explained at the end of the text in the Section Symbols.

**Table 6:** Ligament properties (*N* = 44).

Variable	Unit	Mean	Median	Min	Max	CIMin	CIMax
*Structural properties*							
$A_{CS}$	mm^2^	28.26	18.01	3.71	106.84	21.73	36.33
*V*	mm^3^	184.38	73.41	14.33	970.60	128.98	249.08
*Physical material properties*							
$\rho_{app}$	g cm^−3^	2.26	2.07	1.29	4.46	2.04	2.50
*Mechanical material properties*							
$E_{con}$	MPa	103.48	62.96	15.37	334.84	77.00	131.33
$f_{y}$	MPa	7.58	4.69	1.03	31.13	5.59	9.69
$\varepsilon_{y,con}$	‰	82.16	78.23	26.74	251.73	70.21	94.58
$U_{y,con}$	kJ m^−3^	350.31	169.79	27.02	1574.58	246.67	473.93
$f_{u}$	MPa	10.74	7.65	1.48	42.23	8.19	13.62
$\varepsilon_{u,con}$	‰	176.47	153.04	29.15	405.31	150.89	204.91
$U_{u,con}$	kJ m^−3^	1055.30	858.73	72.14	3680.80	818.97	1331.45
$f_{b}$	MPa	10.54	7.27	1.37	42.06	8.00	13.39
$\varepsilon_{b,con}$	‰	182.92	160.41	30.34	413.28	157.50	212.07
$U_{b,con}$	kJ m^−3^	1114.52	927.39	77.50	3696.10	864.27	1404.61

Symbols are explained at the end of the text in the symbols section.

## Data Availability

The original data presented in the study are freely accessible via the Supplementary Materials and at https://github.com/MarcGebhardt/ExMechEva, accessed on 8 August 2025. Body donor-related data were anonymized to prevent inferences about their identity. Additional data are available from the corresponding author upon reasonable request.

## Supplementary Materials

The following supporting information can be downloaded at: https://www.mdpi.com/article/10.3390/bioengineering12080862/s1, File S1: Standard operation procedure–Testing; File S2: Designs and auxiliaries; File S3: Evaluation code; File S4: Supporting results; File S5: Experimental data.

## Abbreviations

The following abbreviations are used in this manuscript:

AOIlI	Ala ossis ilii inferior
AOIlS	Ala ossis ilii superior
CI	Confidence interval (confidence level of 95%)
COIl	Corpus ossis ilii
COIs	Corpus ossis ischii
CVLu	Corpus vertebrae lumbales
CVSa	Corpus vertebrae sacrales
CT	Computer tomography
DIC	Digital image correlation
FCre	Fascia crescent
FE	Finite element (as in finite element method)
FEnP	Fascia endopelvina
FGlu	Fascia glutea
FTLp	Fascia thoracolumbalis lamina profunda
FTLs	Fascia thoracolumbalis lamina superficalis
IlLu	Ligamentum iliolumbale
Ingu	Ligamentum inguinale
LVDTs	Linear variable differential transformers
MObt	Membrana obturatoria
Pect	Ligamentum pectineum
ROIs	Ramus ossis ischii
ROPu	Ramus superior ossis pubis
SaIla	Ligamenta sacroiliaca anteriora
SaIll	Ligamentum sacroiliacum posterior longum
SaIlp	Ligamenta sacroiliaca posteriora
SaSp	Ligamentum sacrospinale
SaTu	Ligamentum sacrotuberale

## Symbols

The following symbols are used in this manuscript:

*N*	Number of observations (or number of pairs for hypothesis tests)
$t_{\mathrm{mean}}$	Mean thickness of the beam in the direction of loading
$w_{\mathrm{mean}}$	Mean width of the beam
$l_{\mathrm{test}}$	Testing length
ACS	Cross-sectional area
*V*	Volume
$I_{\mathrm{mid}}$	Moment of inertia in mid-span
$\rho_{\mathrm{app}}$	Apparent density
*E*	Elastic modulus
*f*	Strength
ε	Strain
*U*	Strain energy density
_con_	Index for conventional determination of strain
_opt_	Index for optical determination of strain
_y_	Index for yield point (0.2% plastic strain)
_u_	Index for ultimate point
_b_	Index for fracture point
