# Supplementary material for: Approach to Standardized Material Characterization of the Human Lumbopelvic System: Testing and Evaluation
Source: Bioengineering (Basel). 2025 Aug 11;12(8):862. doi: 10.3390/bioengineering12080862 (PMC12383908; doi:10.3390/bioengineering12080862)
Supplement: Supplementary file 1 [file bioengineering-12-00862-s001.zip › File S2 Designs and auxiliaries/Additional_Material_test_Modules_PF010-001_220815.pdf]

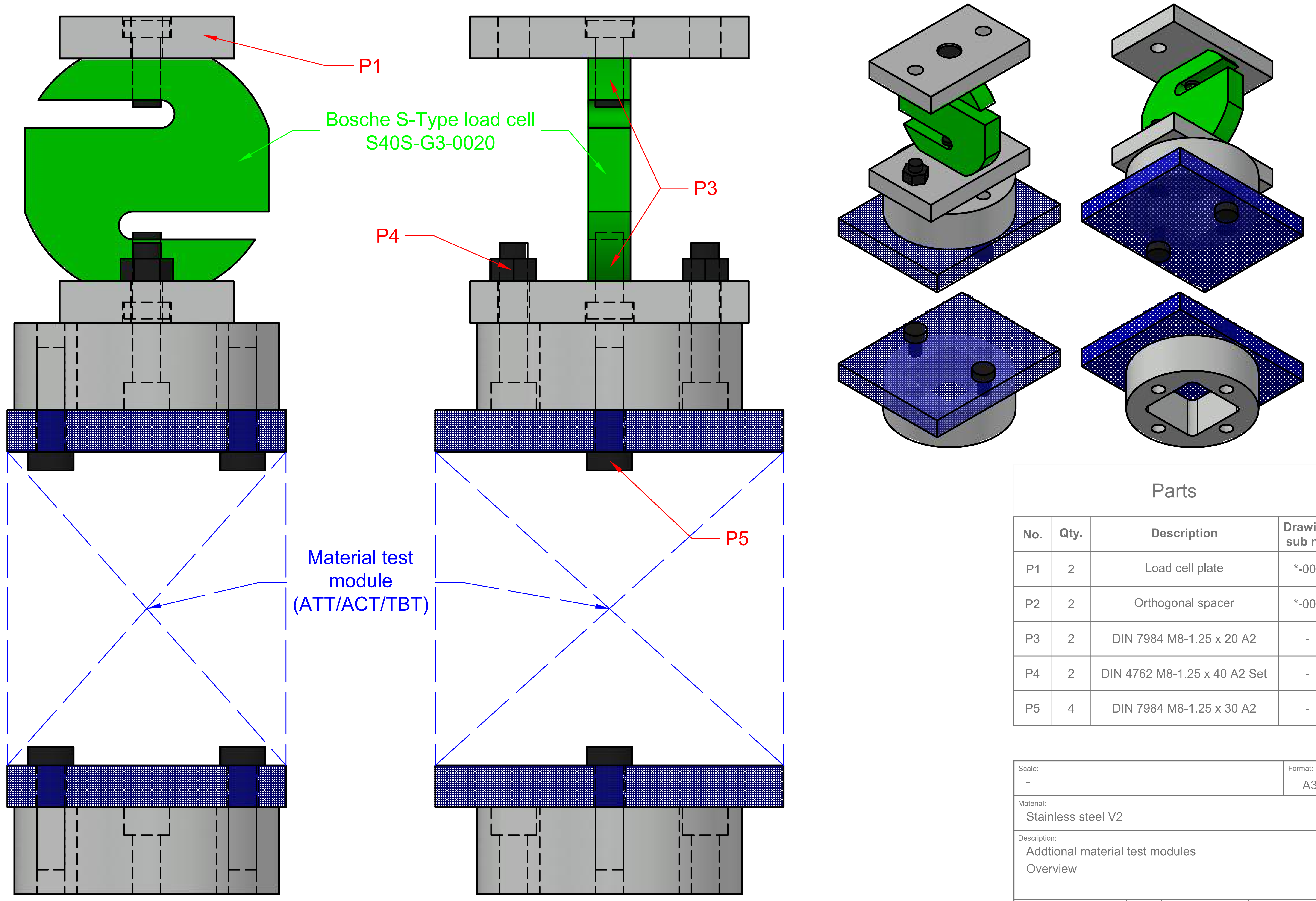

Parts

No.	Qty.	Description	Drawing sub no.
P1	2	Load cell plate	*-001
P2	2	Orthogonal spacer	*-001
P3	2	DIN 7984 M8-1.25 x 20 A2	-
P4	2	DIN 4762 M8-1.25 x 40 A2 Set	-
P5	4	DIN 7984 M8-1.25 x 30 A2	-

Scale: -	Format: A3
Material: Stainless steel V2	
Description: Addtional material test modules Overview	

Drawing number:	Revision:	Original date:	Revision date:
PF010-001-000	1	01.04.2019	15.08.2022

Drawer: Marc Gebhardt

Organisation: HTWK Leipzig University of Applied Sciences

Part 1 - Load cell plate

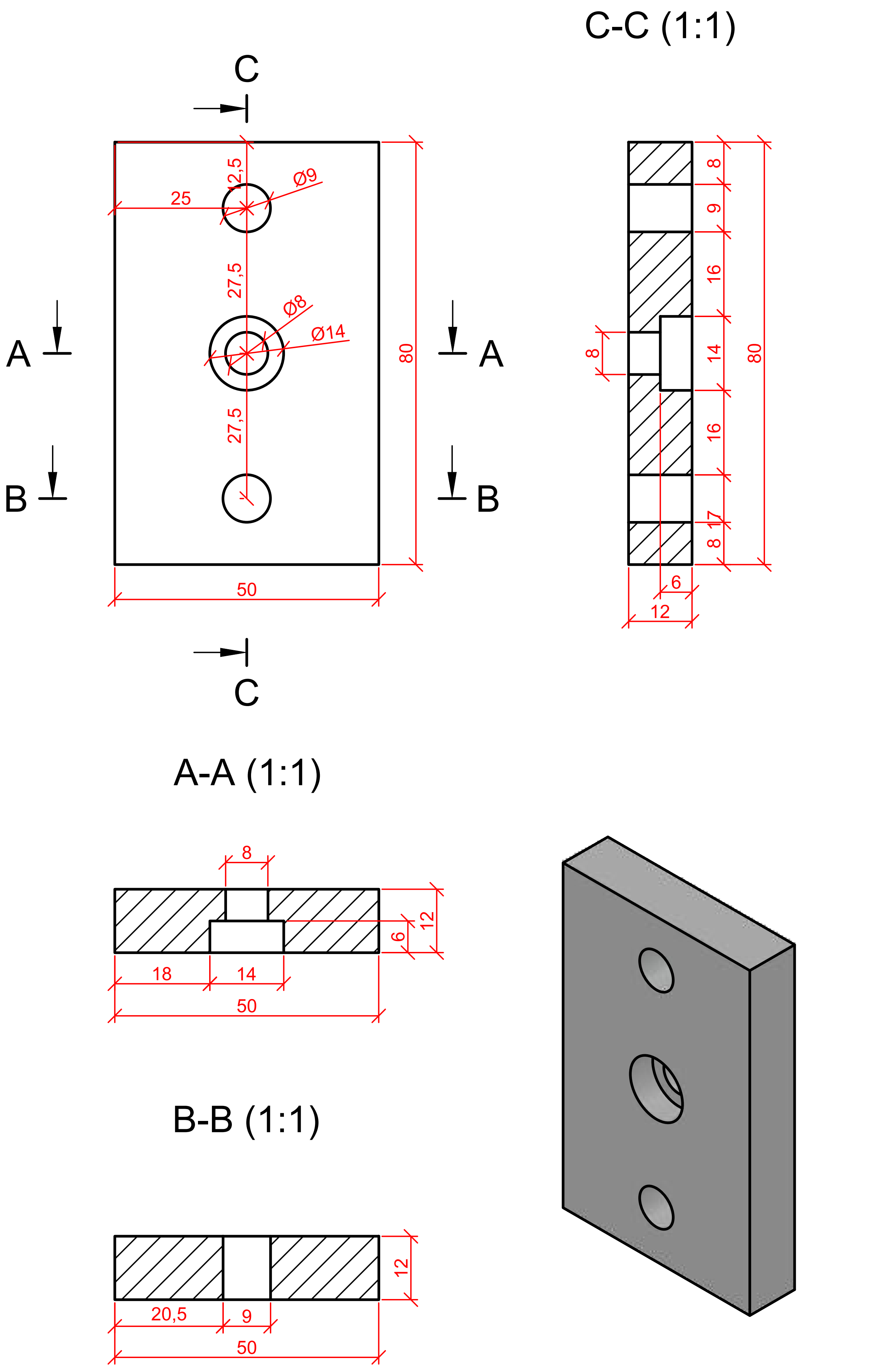

Part 2 - Orthogonal spacer

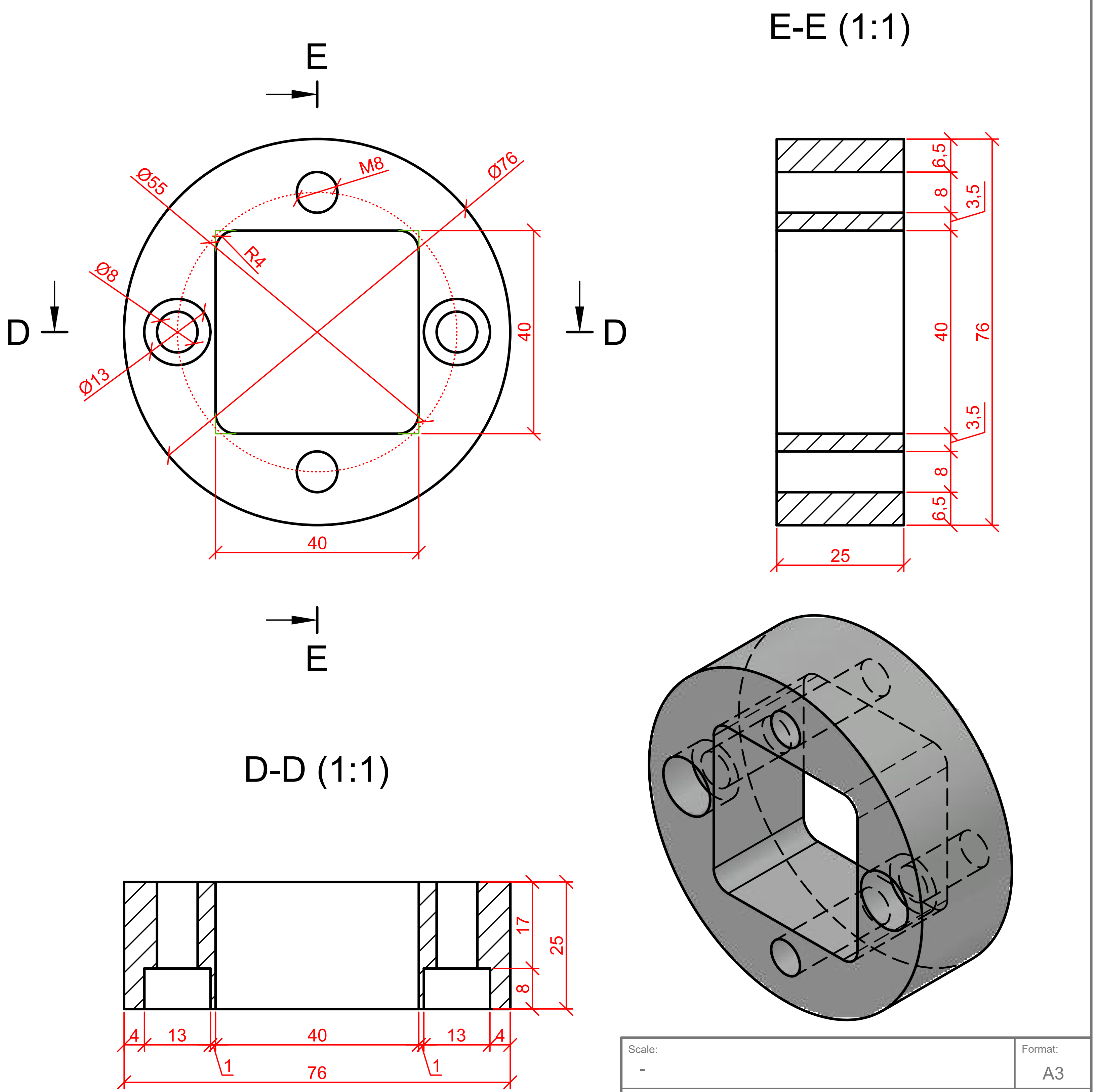

Scale:	Format:
-	A3

Material: Stainless steel V2			
Description: Addtional material test modules Part 1 - Load cell plate and Part 2 - Orthogonal spacer			
Drawing number: PF010-001-000	Revision: 1	Original date: 01.04.2019	Revision date: 15.08.2022
Drawer: Marc Gebhardt	Organisation: HTWK Leipzig University of Applied Sciences		

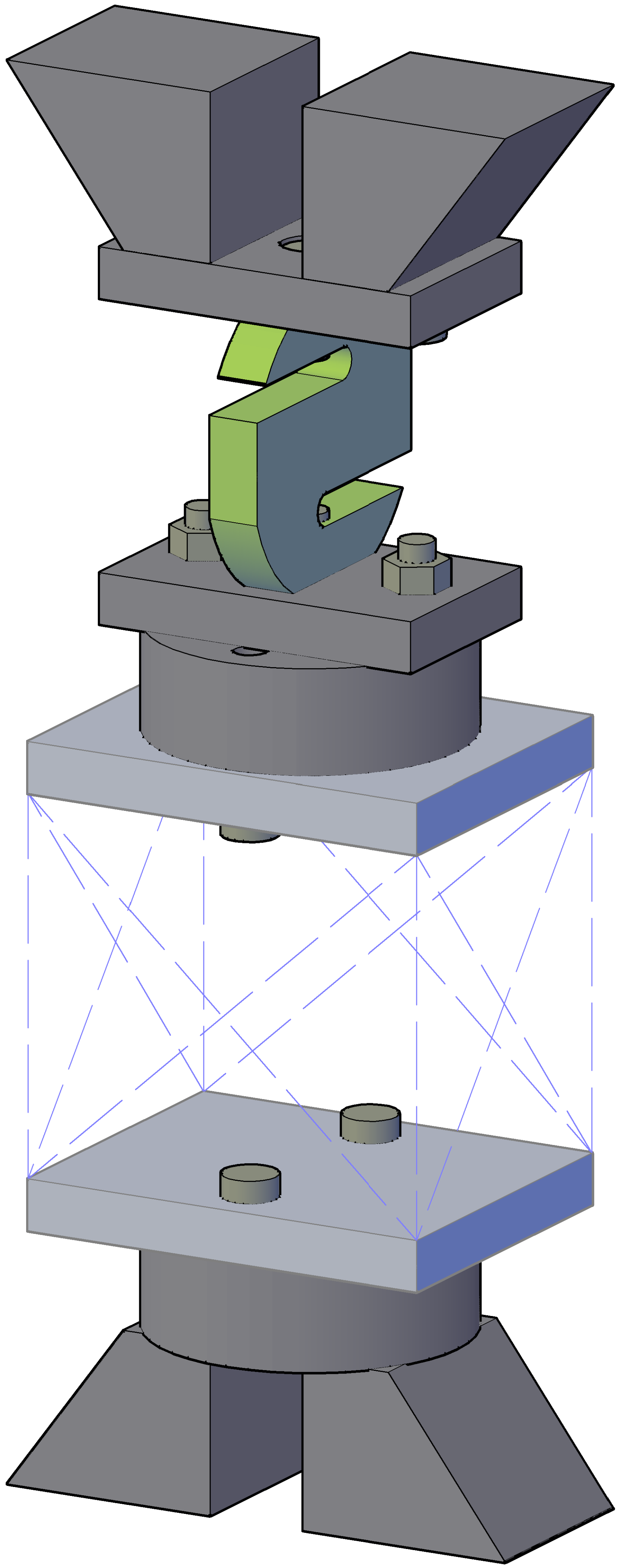

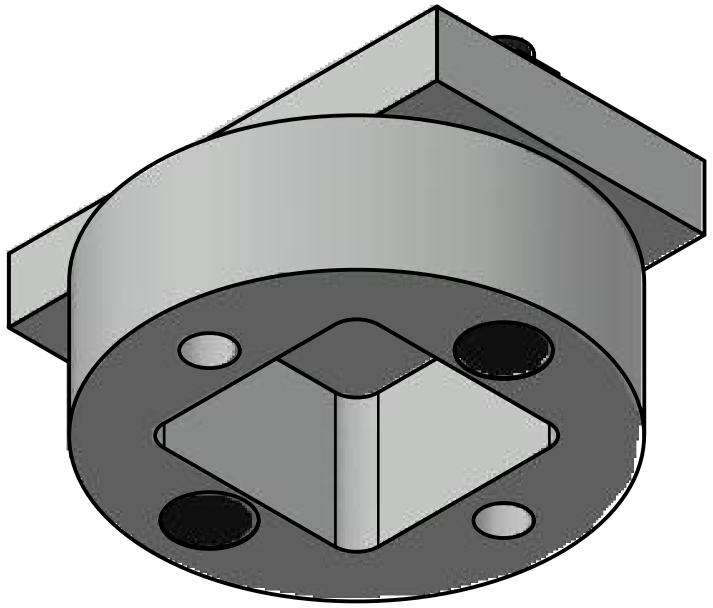

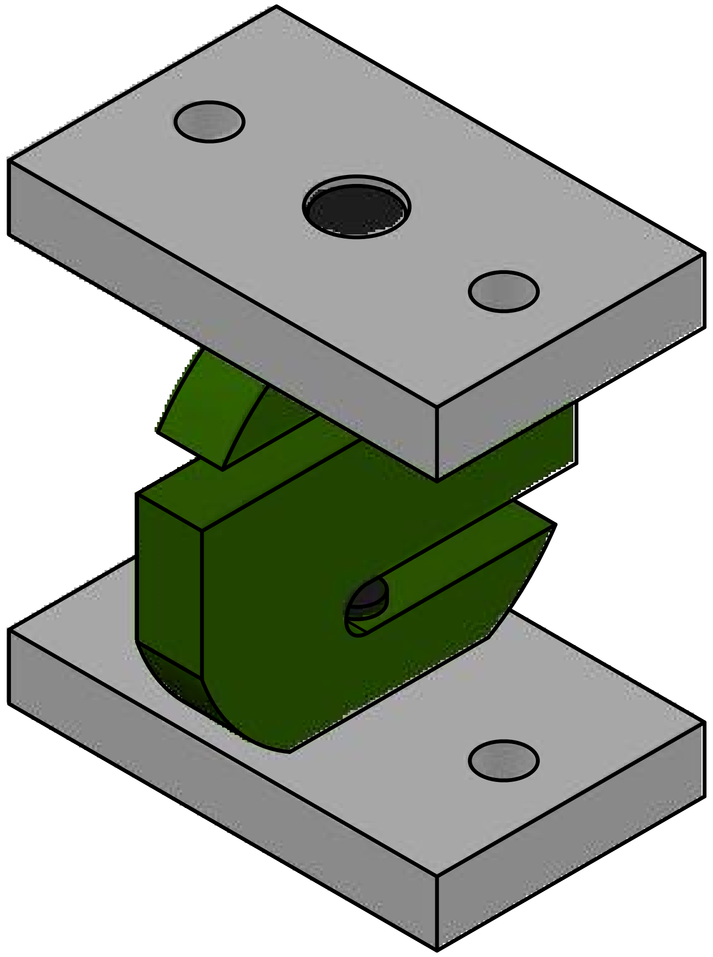

Scale: -				Format: A3
Material: Stainless steel V2				
Description: Addtional material test modules<br>3D rendered view				
Drawing number: PF010-001-010	Revision: 1	Original date: 01.04.2019	Revision date: 15.08.2022	
Drawer: Marc Gebhardt		Organisation: HTWK Leipzig University of Applied Sciences		

Axial Tension Test (ATT) Assembly

Axial Compression Test (ACT) Assembly

Three-Point Bending Test (TBT) Assembly

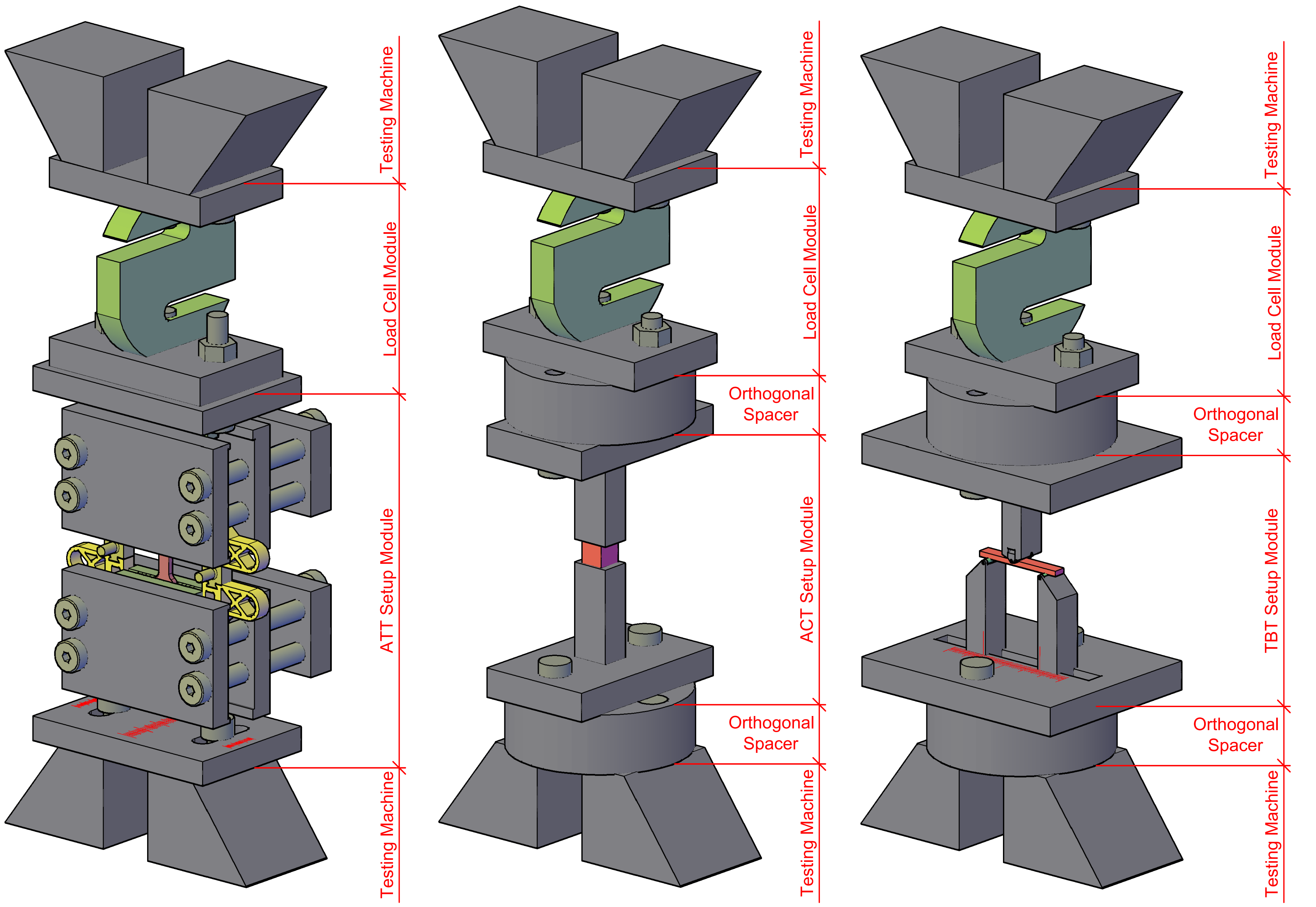

Scale: -				Format: A3
Material: Stainless steel V2				
Description: Addtional material test modules<br>3D rendered view				
Drawing number: PF010-001-009	Revision: 1	Original date: 01.04.2019	Revision date: 15.08.2022	
Drawer: Marc Gebhardt		Organisation: HTWK Leipzig University of Applied Sciences		
