# Supplementary material for: Approach to Standardized Material Characterization of the Human Lumbopelvic System: Testing and Evaluation
Source: Bioengineering (Basel). 2025 Aug 11;12(8):862. doi: 10.3390/bioengineering12080862 (PMC12383908; doi:10.3390/bioengineering12080862)
Supplement: Supplementary file 1 [file bioengineering-12-00862-s001.zip › File S2 Designs and auxiliaries/AST-Speckle-Pattern_Labels_V03-2.pdf]

Labels for mechanical test moduls

Gebhardt M, Kurz S, Grundmann F, Klink T, Slowik V, Heyde C-E, Steinke H.

Approach to standardized material characterization of the human lumbopelvic system – Testing and evaluation.

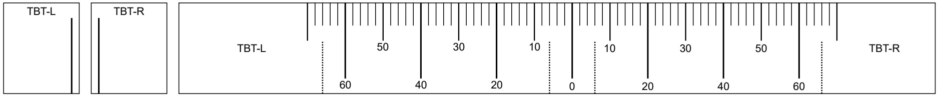

Vertical Print Calibration = 120 mm

Three-Point Bending Test

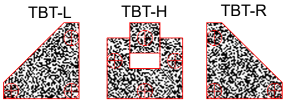

Text

Axial Tension Test

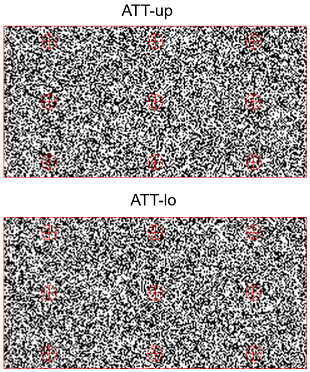

Axial Compression Test

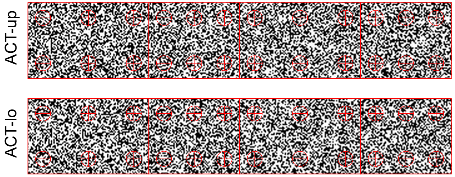

Horizontal Print Calibration = 180 mm
