# Supplementary material for: Approach to Standardized Material Characterization of the Human Lumbopelvic System: Testing and Evaluation
Source: Bioengineering (Basel). 2025 Aug 11;12(8):862. doi: 10.3390/bioengineering12080862 (PMC12383908; doi:10.3390/bioengineering12080862)
Supplement: Supplementary file 1 [file bioengineering-12-00862-s001.zip › File S2 Designs and auxiliaries/Axial_Compression_Test_Setup_PF003-002_220809.pdf]

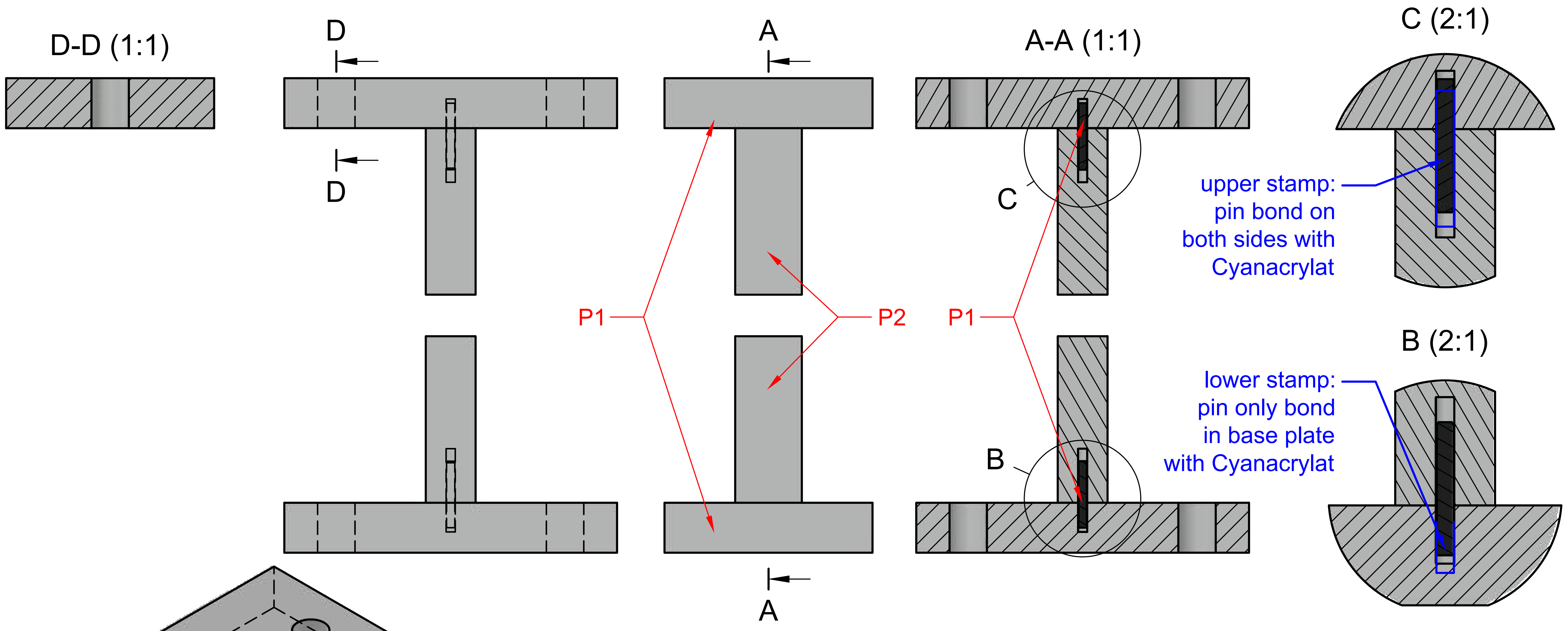

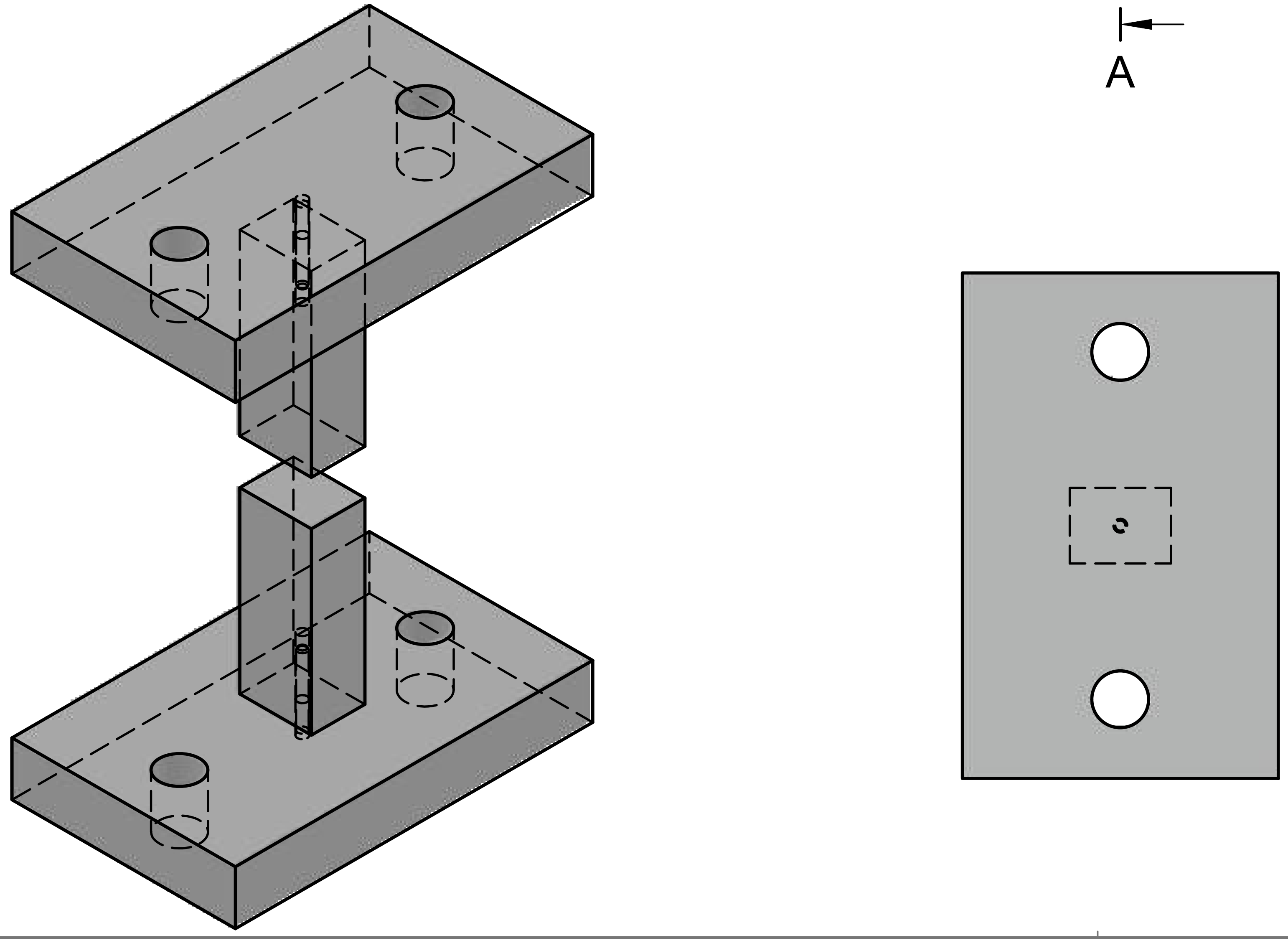

Parts

No.	Qty.	Description	Drawing sub no.
P1	2	Base plate	*-001
P2	2	Stamp	*-001
P3	2	Steel pin 2 x 16 mm	

Scale: -			Format: A3
Material: Stainless steel V2			
Description: Axial compression test setup for trabecular bone<br>Overview			
Drawing number: PF003-002-000	Revision: 2	Original date: 10.12.2018	Revision date: 09.08.2022
Drawer: Marc Gebhardt		Organisation: HTWK Leipzig University of Applied Sciences	

Part 1 - Base plate

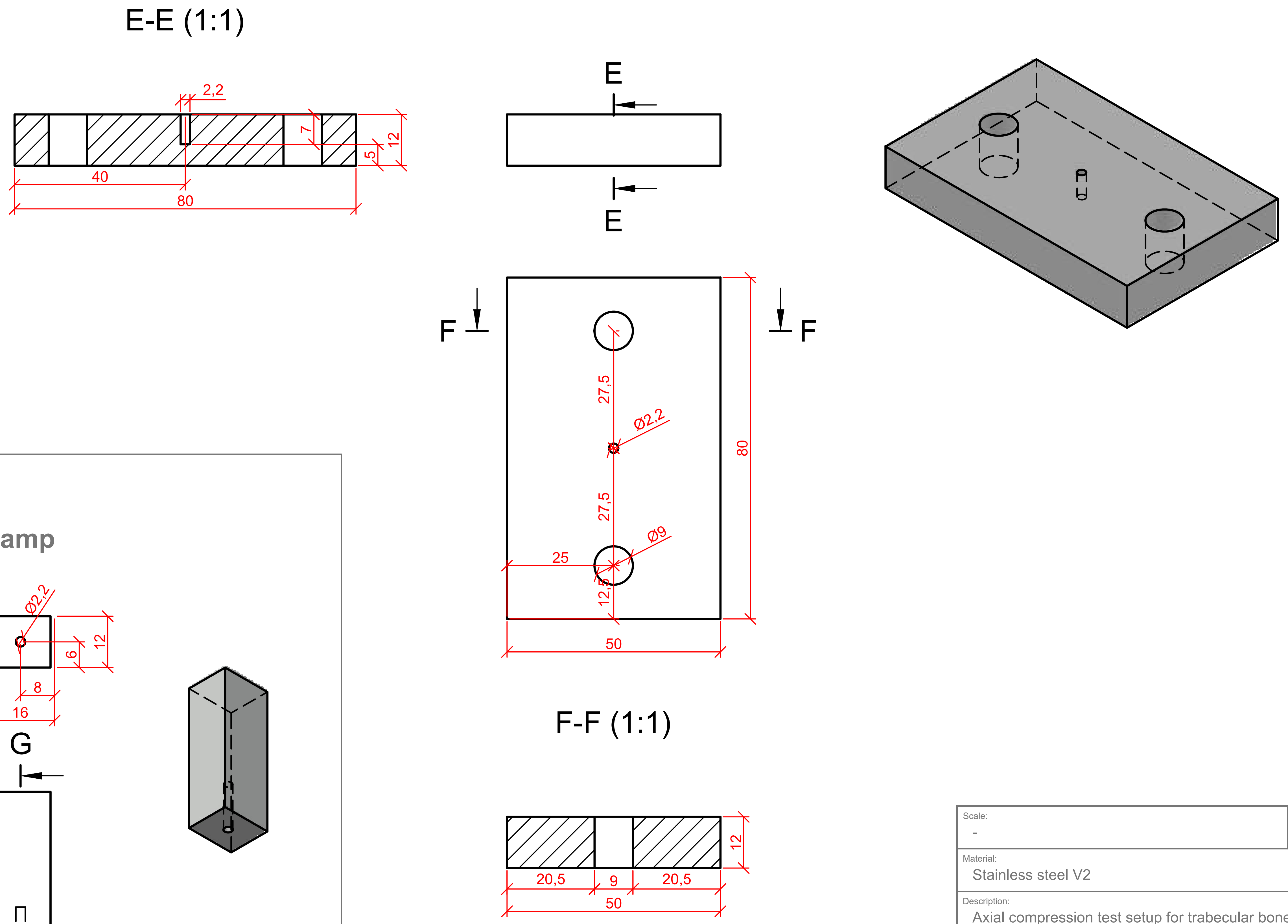

Part 2 - Stamp

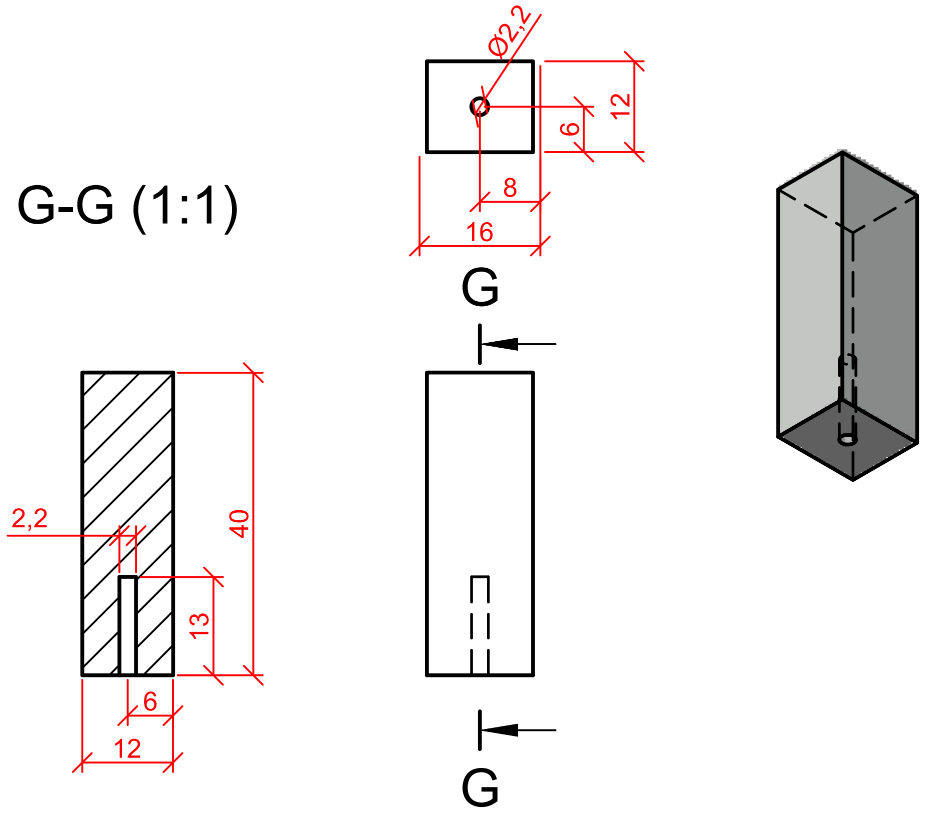

Scale: -	Format: A3
Material: Stainless steel V2	
Description: Axial compression test setup for trabecular bone<br>Part 1 - Base plate and<br>Part 2 - Stamp	

Drawing number:	Revision:	Original date:	Revision date:
PF003-002-001	2	10.12.2018	09.08.2022

Drawer:	Organisation:
Marc Gebhardt	HTWK Leipzig University of Applied Sciences

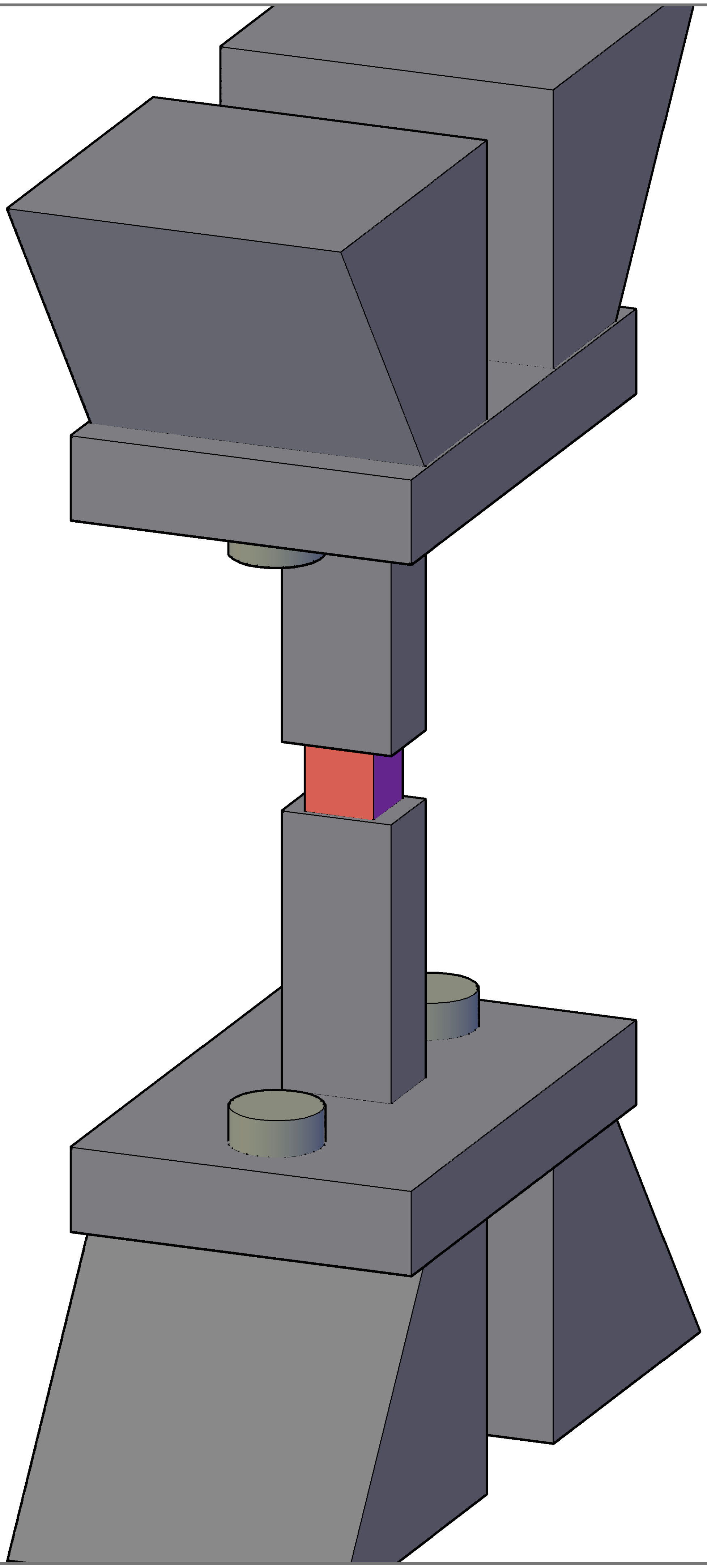

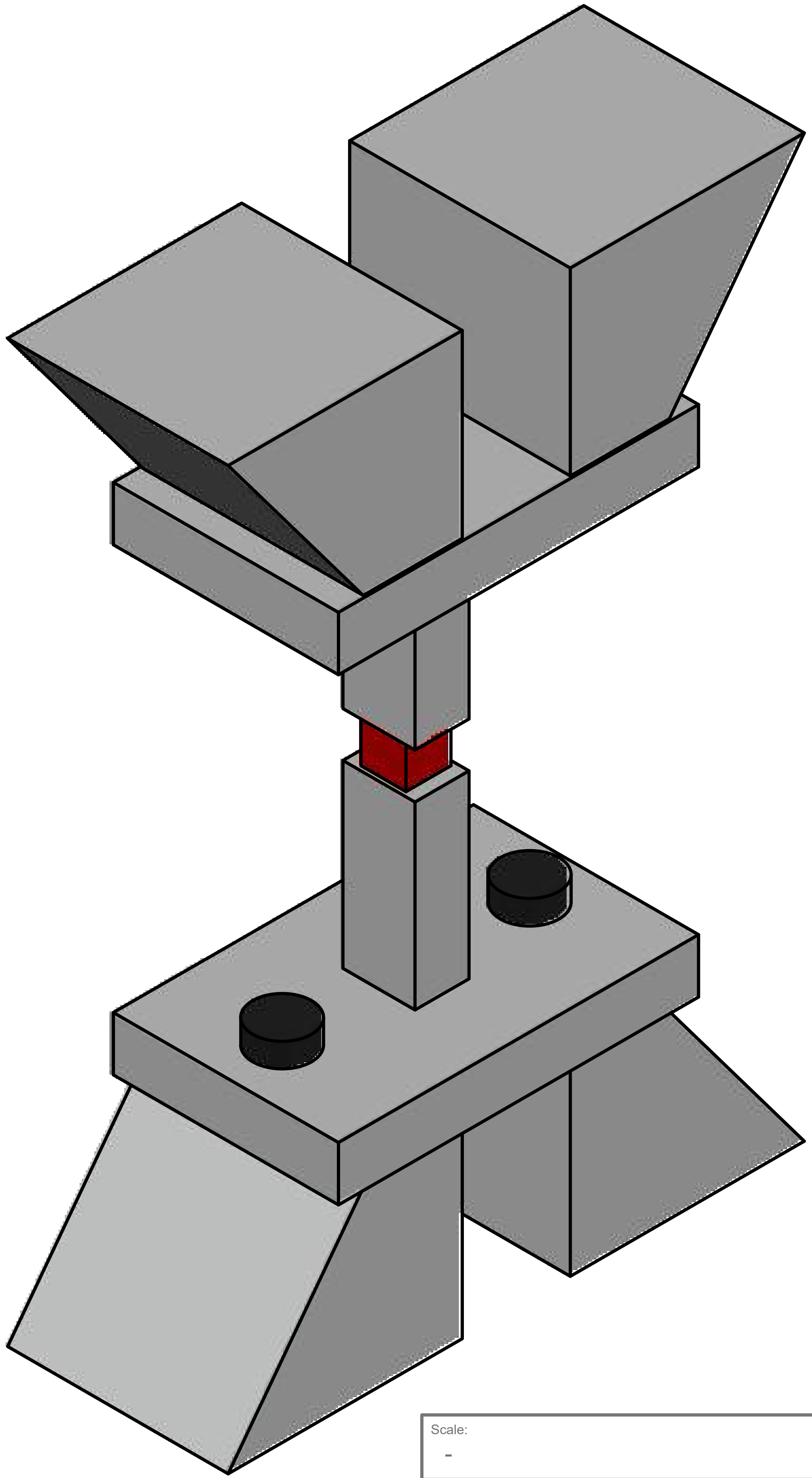

Scale: -			Format: A3
Material: Stainless steel V2			
Description: Axial compression test setup for trabecular bone<br>3D rendered view			
Drawing number: PF003-002-010	Revision: 2	Original date: 10.12.2018	Revision date: 09.08.2022
Drawer: Marc Gebhardt		Organisation: HTWK Leipzig University of Applied Sciences	
