# Supplementary material for: Approach to Standardized Material Characterization of the Human Lumbopelvic System: Testing and Evaluation
Source: Bioengineering (Basel). 2025 Aug 11;12(8):862. doi: 10.3390/bioengineering12080862 (PMC12383908; doi:10.3390/bioengineering12080862)
Supplement: Supplementary file 1 [file bioengineering-12-00862-s001.zip › File S2 Designs and auxiliaries/Axial_Tension_Test_Setup_PF002-004_220810.pdf]

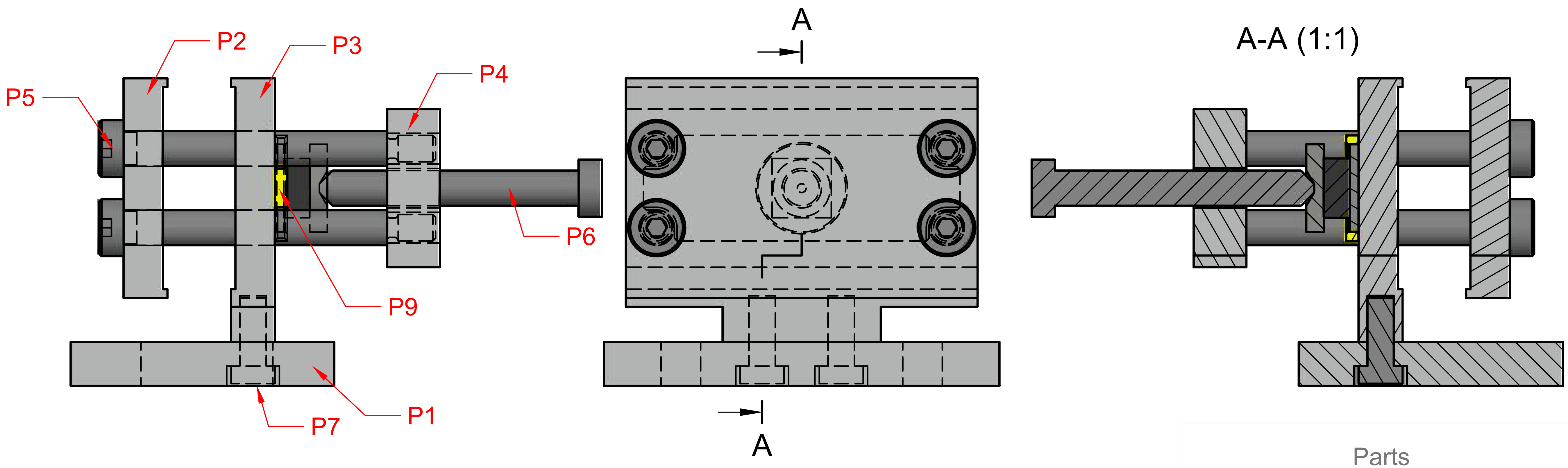

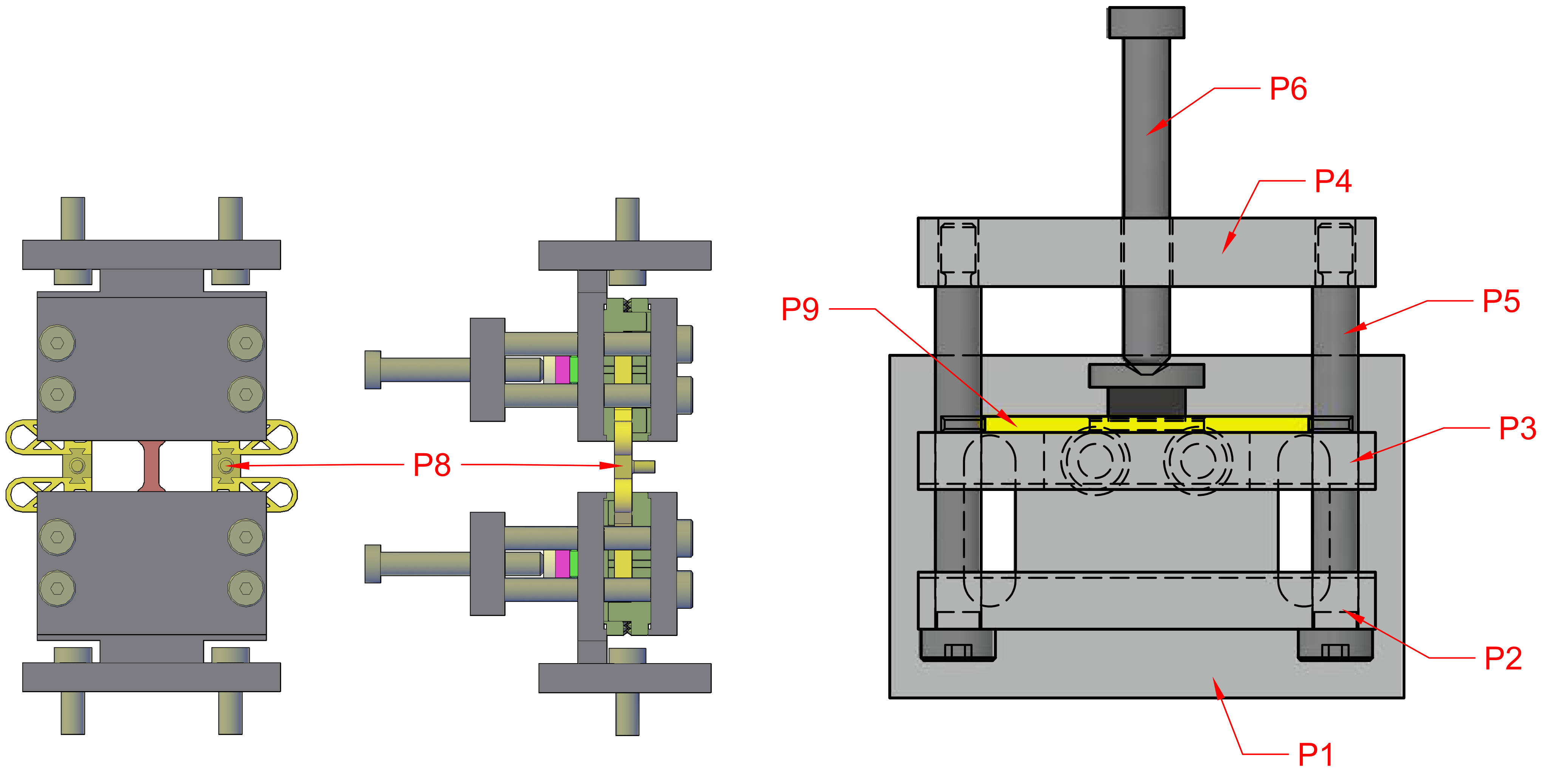

Parts

No.	Qty.	Description	Drawing sub no.
P1	2	Base plate	*-001
P2	2	Clamping plate - front	*-002
P3	2	Clamping plate - back	*-003
P4	2	Counter plate	*-004
P5	8	Fitting screw A2 12.8 8 x 60 (Norelem 07534-108x60)	-
P6	2	DIN EN 24017 M8-1.25 x 60	-
P7	4	DIN 7984 M6-1.0 x 16 A2	-
P8	1	Clamp mount set	*-005
P9	2	Elastic storage	*-005

Scale: -	Format: A3
Material: Stainless steel V2	
Description: Axial tension test setup for soft tissue<br>Overview	

Drawing number:	Revision:	Original date:	Revision date:
PF002-004-000	4	05.11.2018	10.08.2022

Drawer: Marc Gebhardt

Organisation: HTWK Leipzig University of Applied Sciences

B-B (1:1)

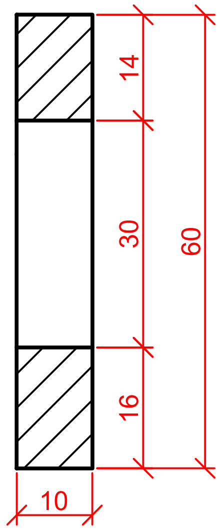

C-C (1:1)

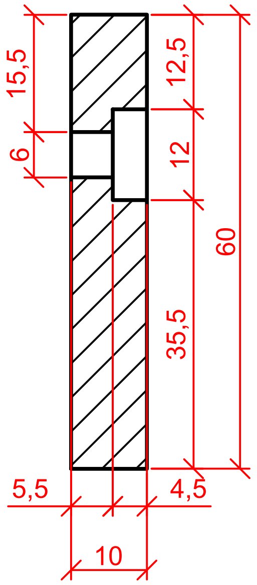

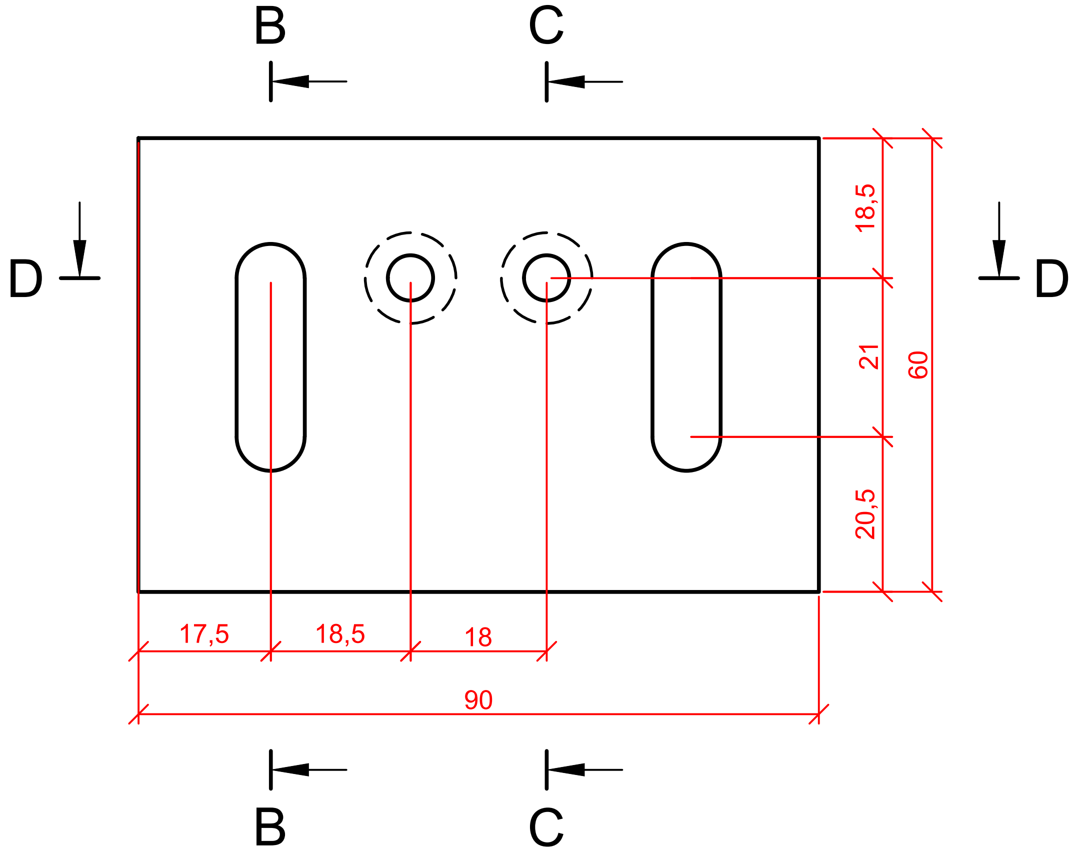

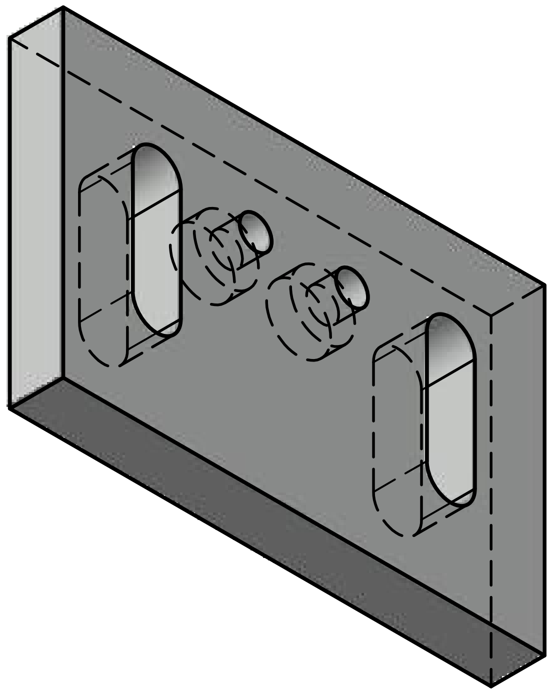

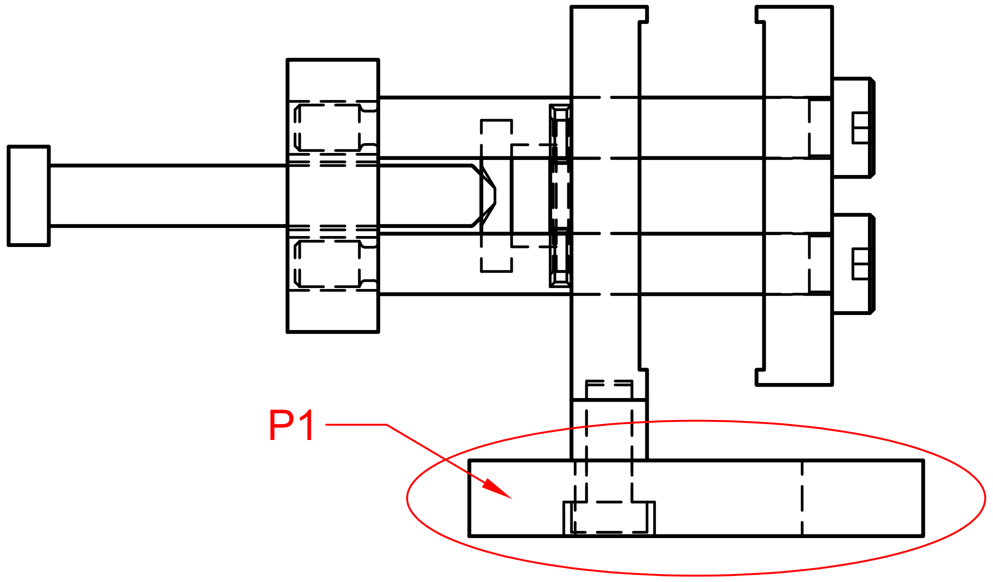

D-D (1:1)

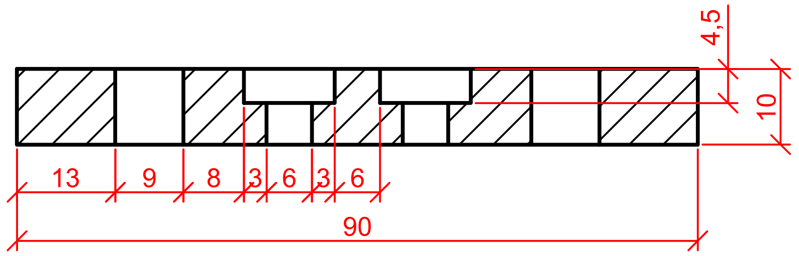

Scale: -				Format: A3
Material: Stainless steel V2				
Description: Axial tension test setup for soft tissue<br>Part 1 - Base plate				
Drawing number: PF002-004-001	Revision: 4	Original date: 05.11.2018	Revision date: 10.08.2022	
Drawer: Marc Gebhardt		Organisation: HTWK Leipzig University of Applied Sciences		

E-E (1:1)

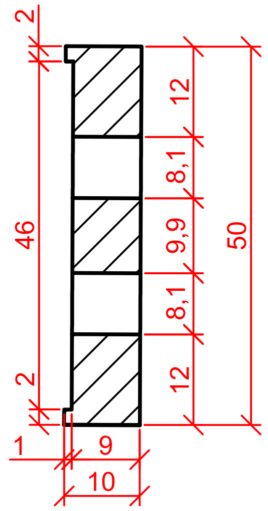

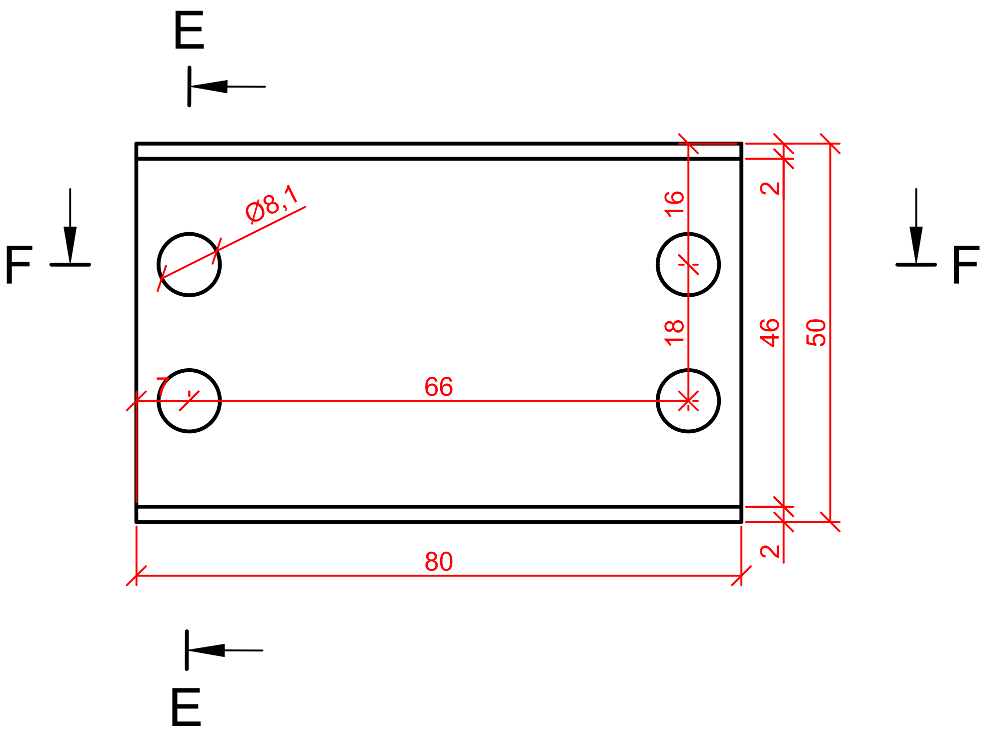

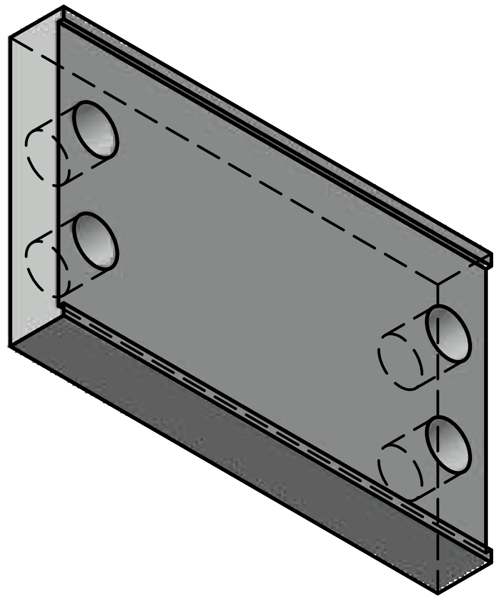

F-F (1:1)

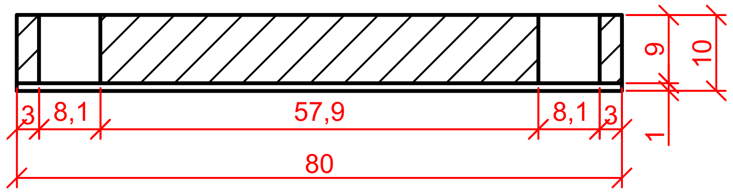

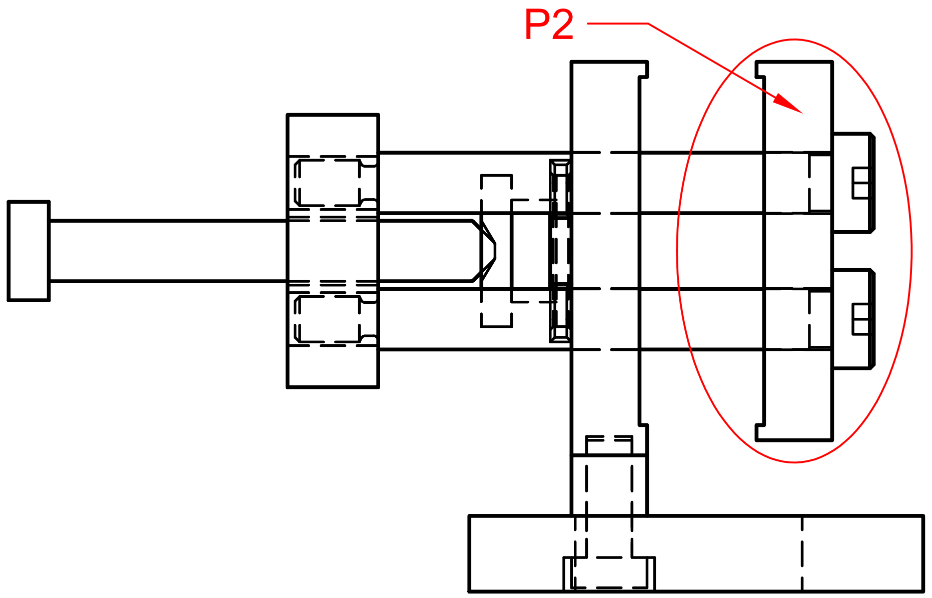

Scale: -			Format: A3
Material: Stainless steel V2			
Description: Axial tension test setup for soft tissue<br>Part 2 - Clamping plate - front			
Drawing number: PF002-004-002	Revision: 4	Original date: 05.11.2018	Revision date: 10.08.2022
Drawer: Marc Gebhardt	Organisation: HTWK Leipzig University of Applied Sciences		

G-G (1:1)

H-H (1:1)

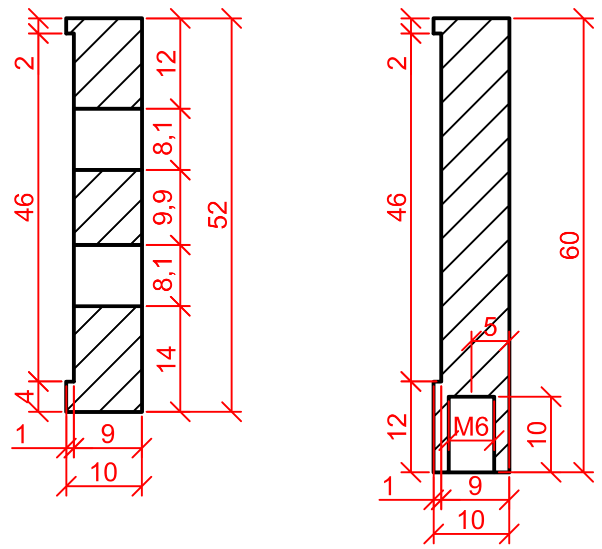

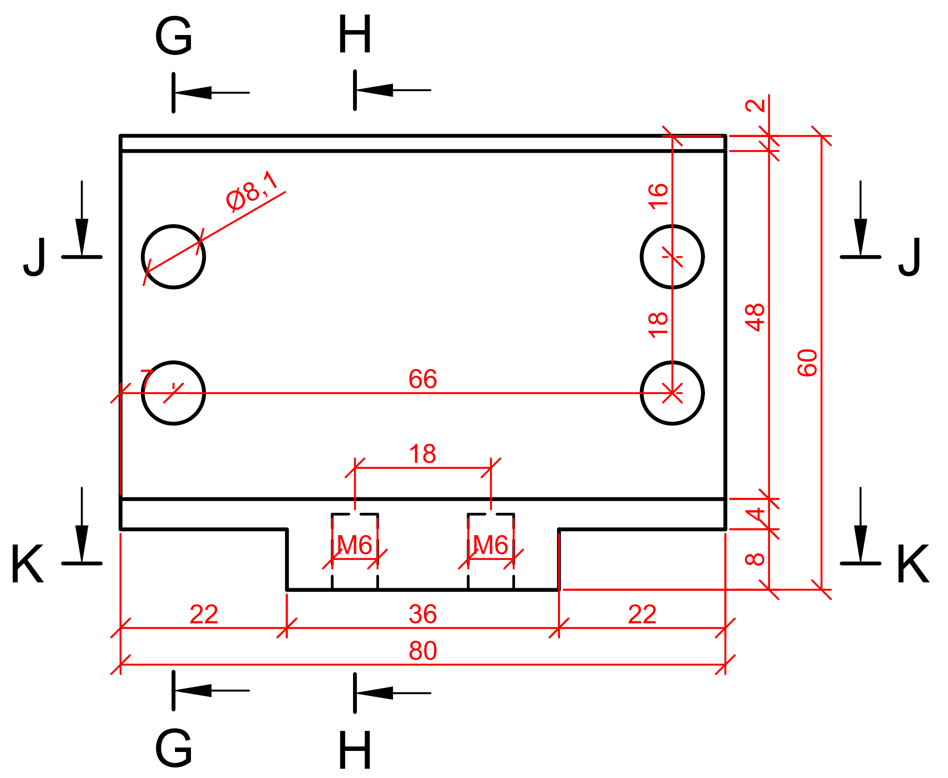

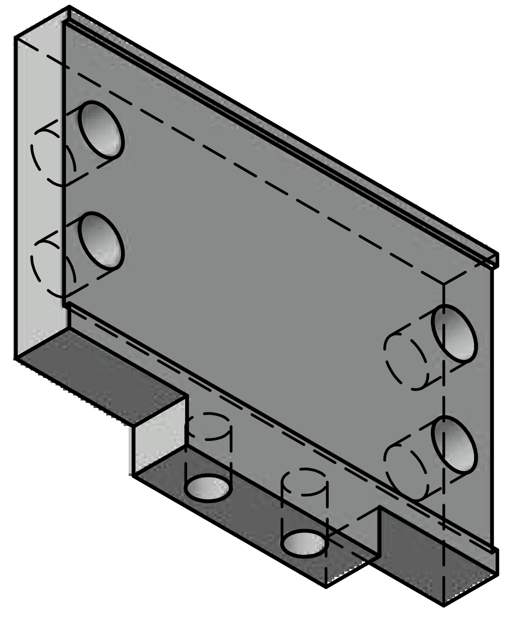

J-J (1:1)

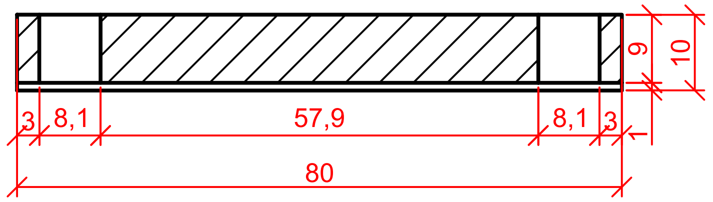

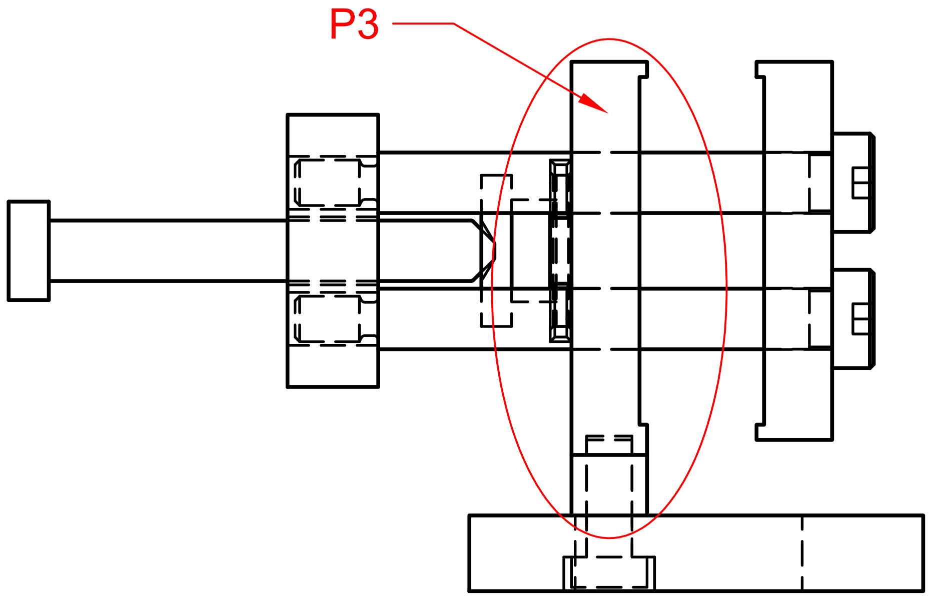

K-K (1:1)

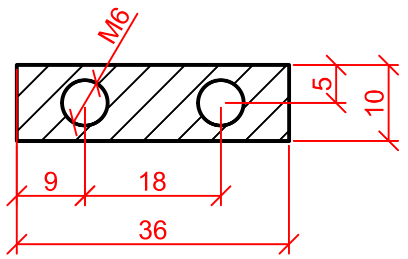

Scale:			Format:
-			A3
Material: Stainless steel V2			
Description: Axial tension test setup for soft tissue<br>Part 3 - Clamping plate - back			
Drawing number: PF002-004-003	Revision: 4	Original date: 05.11.2018	Revision date: 10.08.2022
Drawer: Marc Gebhardt		Organisation: HTWK Leipzig University of Applied Sciences	

L-L (1:1)

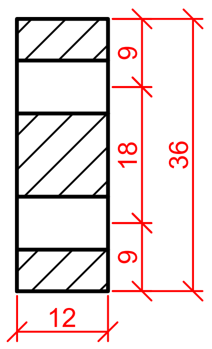

M-M (1:1)

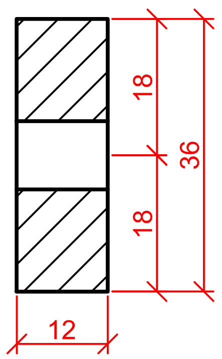

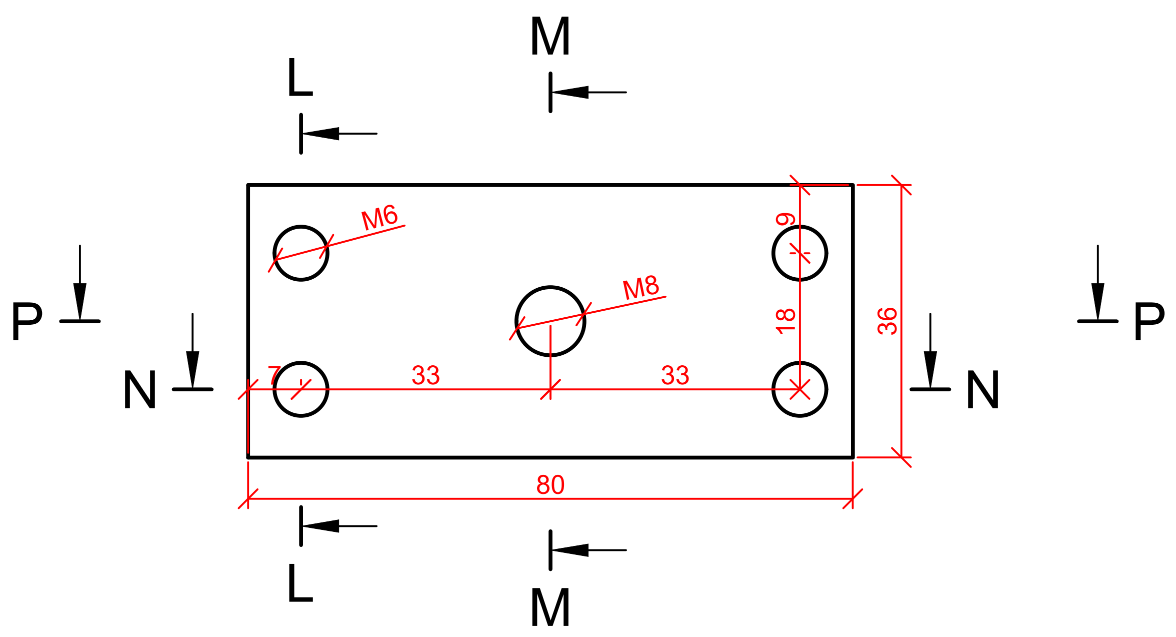

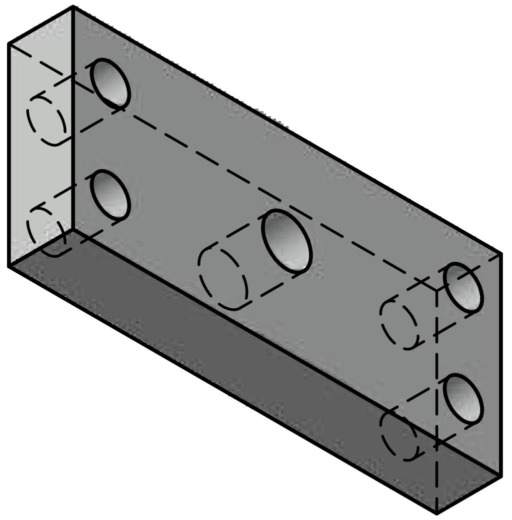

P-P (1:1)

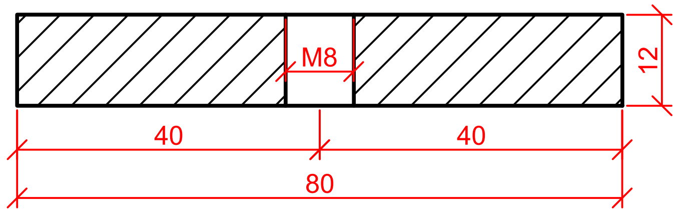

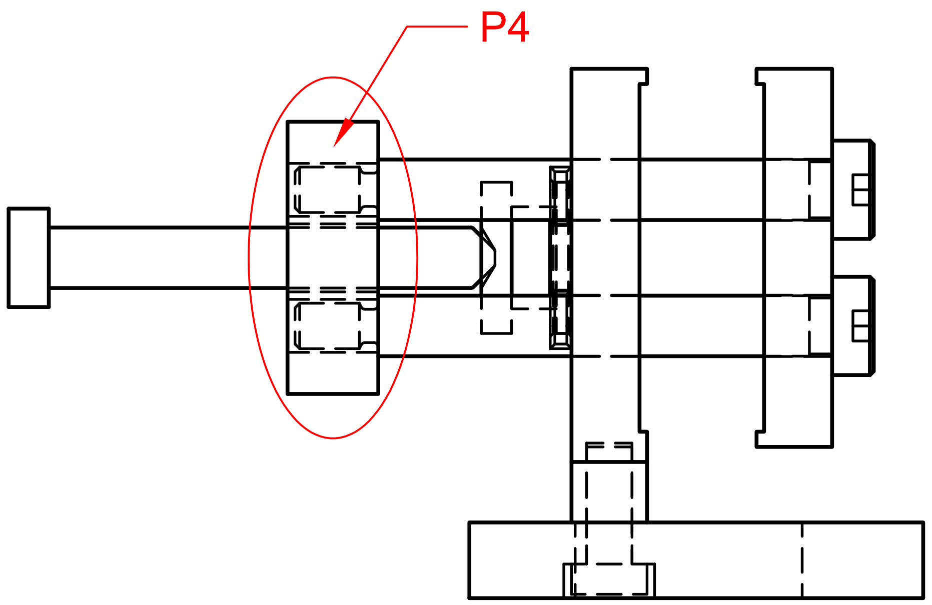

N-N (1:1)

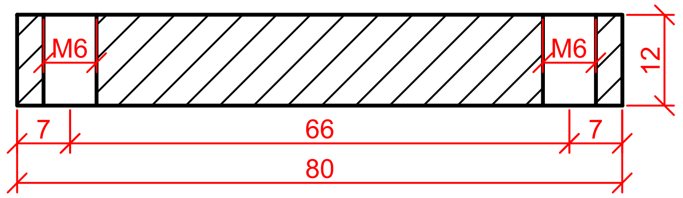

Scale: -				Format: A3
Material: Stainless steel V2				
Description: Axial tension test setup for soft tissue<br>Part 4 - Counter plate				
Drawing number: PF002-004-004	Revision: 4	Original date: 05.11.2018	Revision date: 10.08.2022	
Drawer: Marc Gebhardt		Organisation: HTWK Leipzig University of Applied Sciences		

Part 8 - Clamp mount set

For further information please see
"ATT_Clamp mount_V04.pdf" and
"ATT_Preparation_V02.pdf".

Modified on the basis of: Scholze M, Singh A, Lozano PF, Ondruschka B, Ramezani M, Werner M, et al. Utilization of 3D printing technology to facilitate and standardize soft tissue testing. Sci Rep. 2018; 8:11340. Epub 2018/07/27. doi: 10.1038/s41598-018-29583-4 PMID: 30054509.

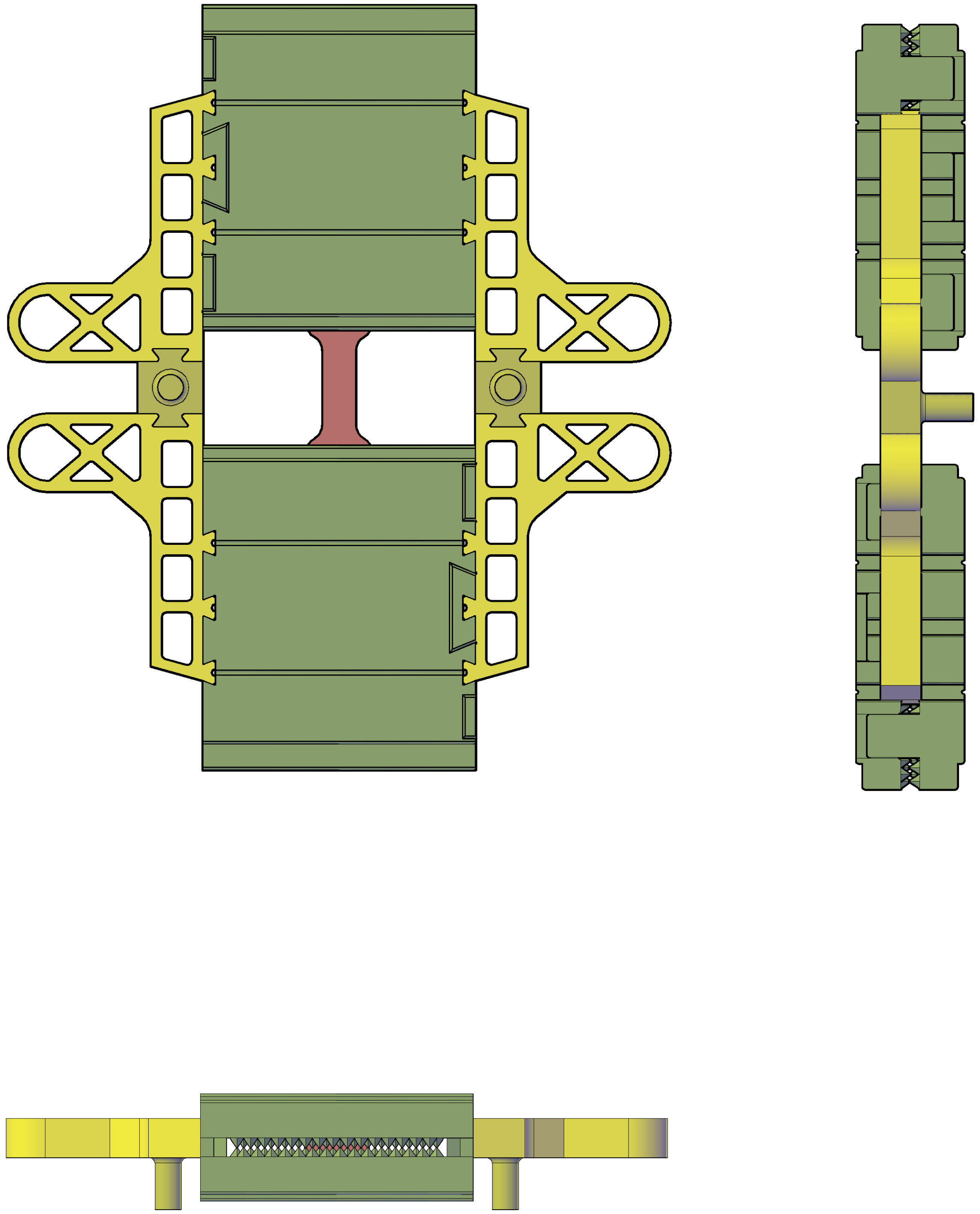

Part 9 - Elastic storage

For further information please see
"ATT_elastic_storage_holder_V01.pdf".

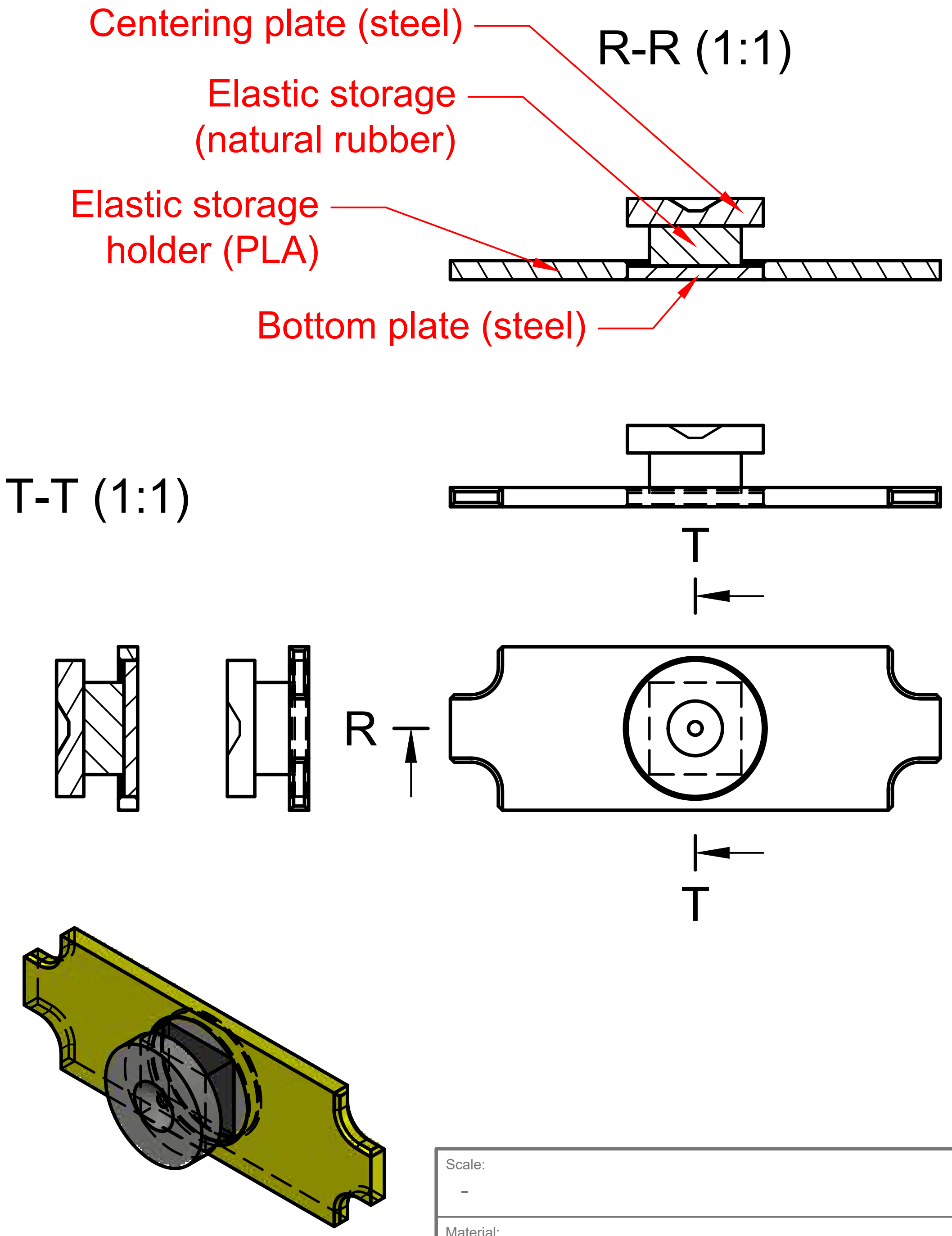

Scale: -			Format: A3
Material: -			
Description: Axial tension test setup for soft tissue Special			
Drawing number: PF002-004-005	Revision: 4	Original date: 05.11.2018	Revision date: 10.08.2022
Drawer: Marc Gebhardt		Organisation: HTWK Leipzig University of Applied Sciences	

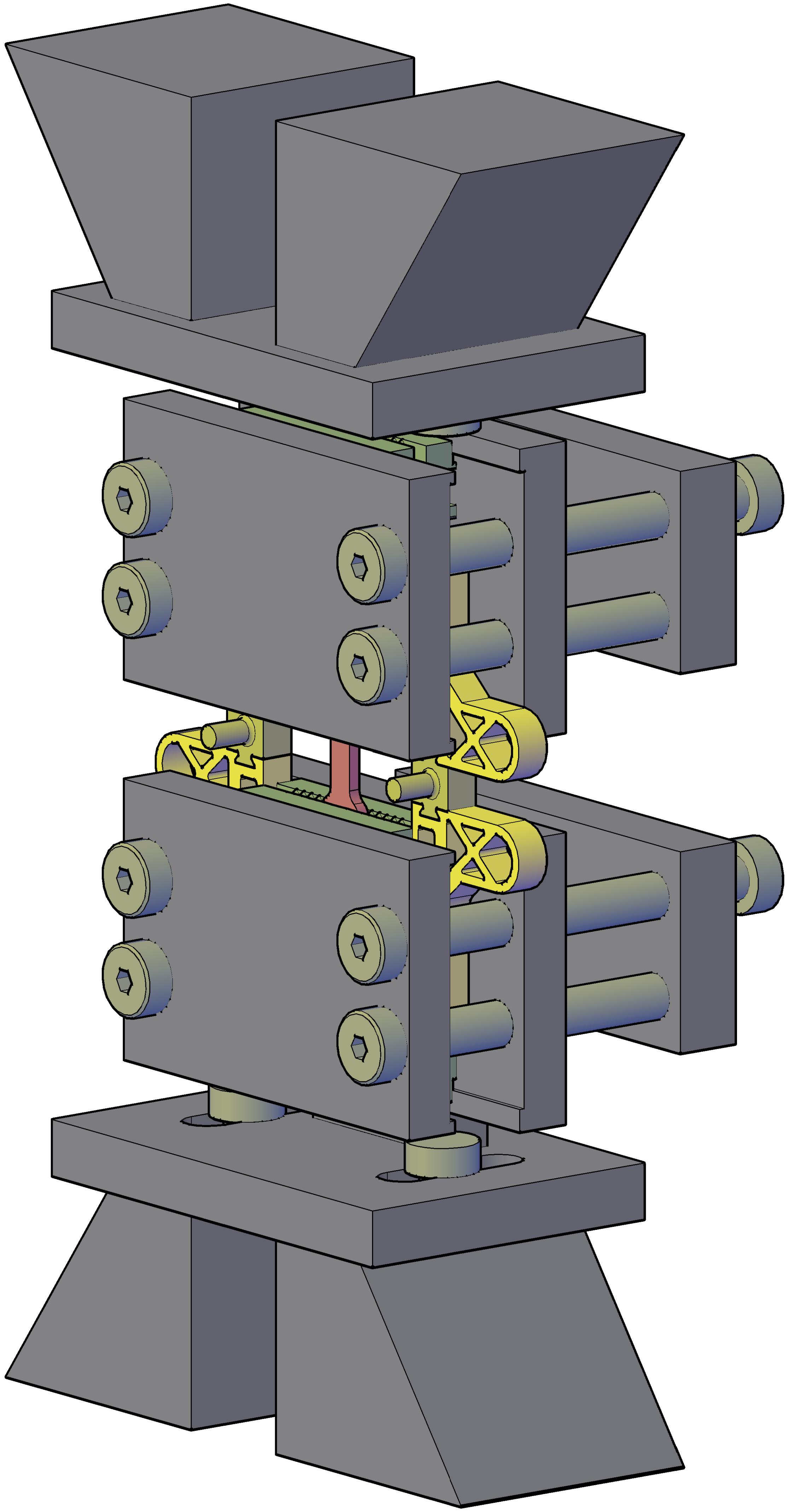

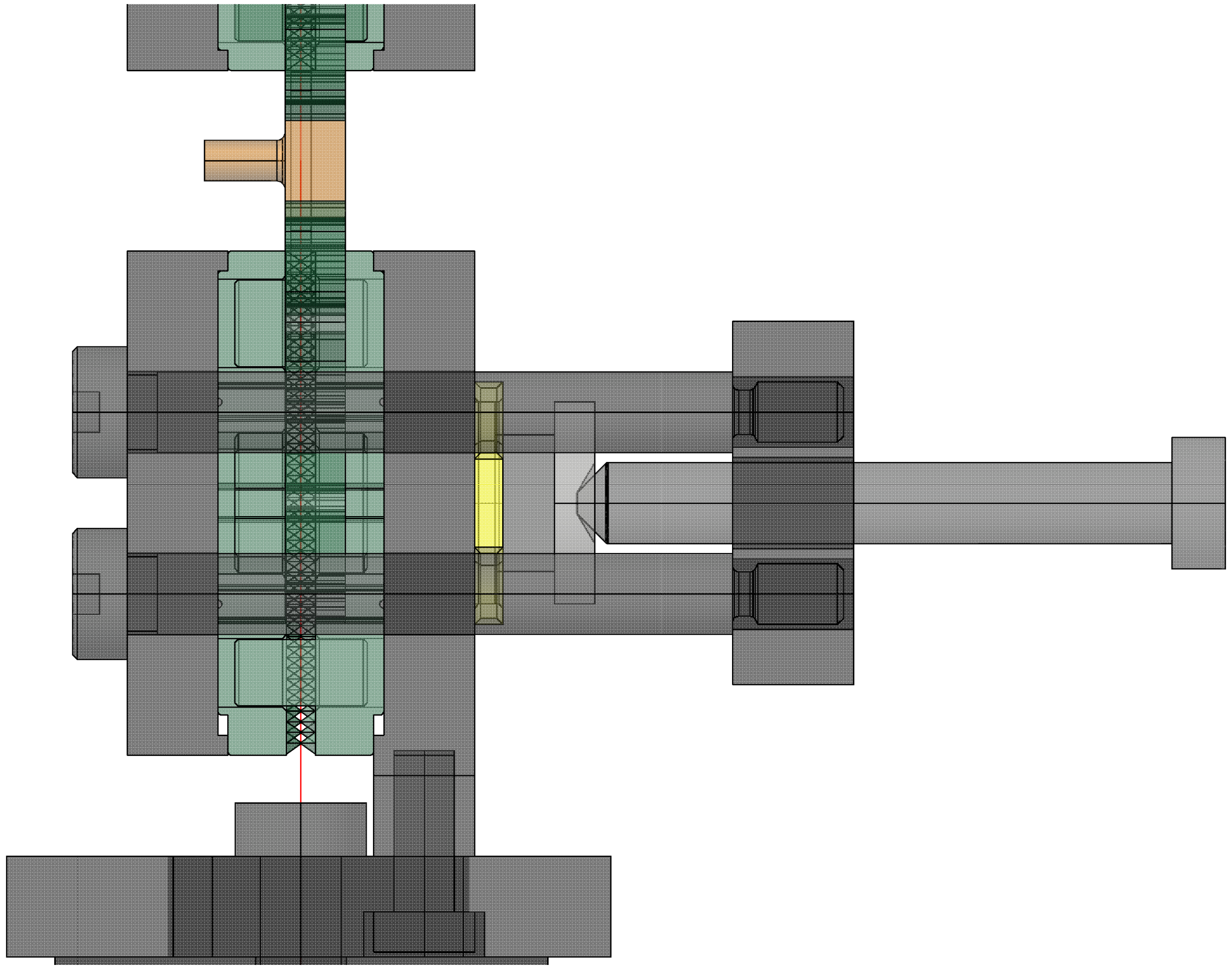

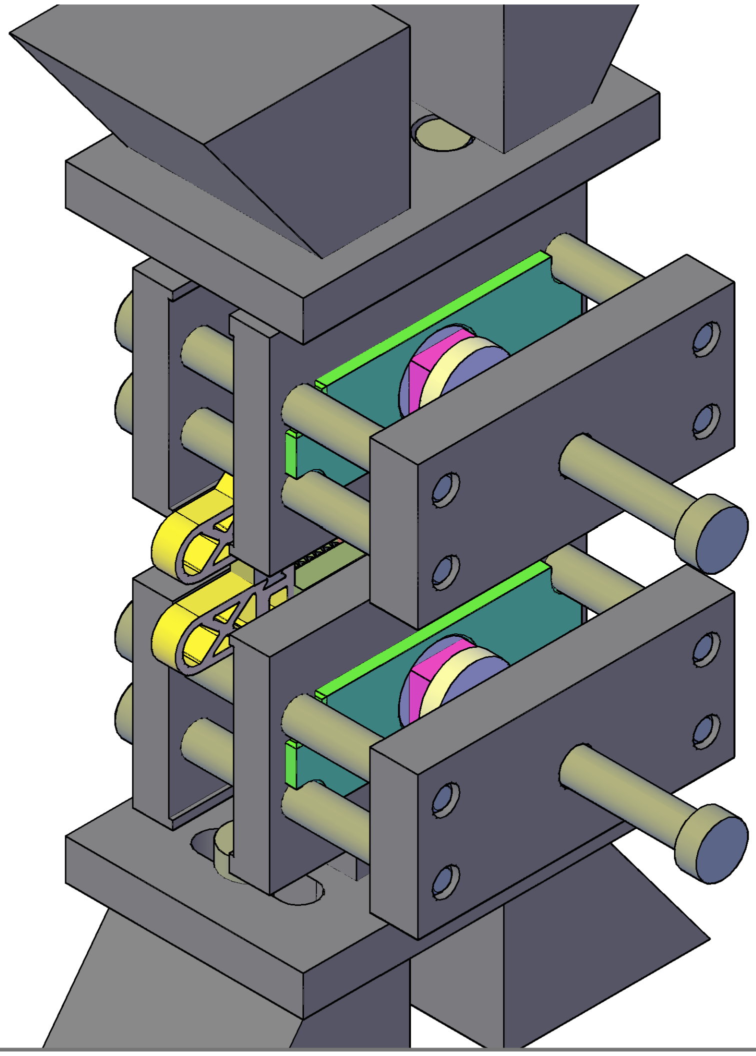

Scale:				Format:
-				A3
Material: Stainless steel V2				
Description: Axial tension test setup for soft tissue<br>3D rendered view				
Drawing number: PF002-004-010	Revision: 4	Original date: 05.11.2018	Revision date: 10.08.2022	
Drawer: Marc Gebhardt		Organisation: HTWK Leipzig University of Applied Sciences		
