# Supplementary material for: Approach to Standardized Material Characterization of the Human Lumbopelvic System: Testing and Evaluation
Source: Bioengineering (Basel). 2025 Aug 11;12(8):862. doi: 10.3390/bioengineering12080862 (PMC12383908; doi:10.3390/bioengineering12080862)
Supplement: Supplementary file 1 [file bioengineering-12-00862-s001.zip › File S2 Designs and auxiliaries/Models/ATT_Clamp mount_V04.pdf]

Clamp mount for axial tensile test of soft tissue

HTWK
Leipzig University
of Applied Sciences

Faculty of
Civil Engineering

Title	ATT-Soft tissue-Clamp mount V04
Subject	Biomechanics-Testing
Revision	2019-10-25-001
Author	Gebhardt, Marc

Notes

Clamp mount for axial tensile test of soft tissue specimen with 20 mm test length.

Manufacturing by Fused Deposition Modeling (FDM).
Tested with following settings:

- Nozzle = 0.4 mm
- Clamp, Support-Spacer:
  - Filament material = PLA
  - Resolution = 0.1 mm
  - Infill density = 50 %
  - Print speed = 40 mm/s
- Support:
  - Filament material = TPU-medium
  - Resolution = 0.2 mm
  - Infill density = 100 %
  - Print speed = 15 mm/s

Modified on the basis of "Scholze, M. et al. Utilization of 3D printing technology to facilitate and standardize soft tissue testing. Scientific reports 8, 11340; 10.1038/s41598-018-29583-4 (2018)".

Supported by:

Federal Ministry
for Economic Affairs
and Energy

on the basis of a decision
by the German Bundestag

ZIM
Zentrales
Innovationsprogramm
Mittelstand

CC BY
