# Supplementary material for: Approach to Standardized Material Characterization of the Human Lumbopelvic System: Testing and Evaluation
Source: Bioengineering (Basel). 2025 Aug 11;12(8):862. doi: 10.3390/bioengineering12080862 (PMC12383908; doi:10.3390/bioengineering12080862)
Supplement: Supplementary file 1 [file bioengineering-12-00862-s001.zip › File S2 Designs and auxiliaries/Models/ATT_elastic_storage_holder_V01.pdf]

Elastic storage for axial tensile test of soft tissue

HITWK B

Leipzig University
of Applied Sciences

Faculty of
Civil Engineering

Title	ATT-Soft tissue-Elastic storage
Subject	Biomechanics-Testing
Revision	2019-11-25-001
Author	Gebhardt, Marc

Notes

Elastic storage for axial tensile test of soft tissue specimen.

Manufacturing by Fused Deposition Modeling (FDM).
Tested with following settings:
- Nozzle = 0.4 mm
- Filament material = PLA
- Resolution = 0.2 mm
- Infill density = 50 %
- Print speed = 60 mm/s

Additional parts (bonding via Cyanacrylat-glue):
- Elastic storage:
  - Material = natural rubber
  - Geometry (widthxthickness) = 13.5x5.9 mm
- Centering plate:
  - Material = stainless steel
  - Geometry (diameterxthickness) = 20x4 mm
- Bottom platee:
  - Material = stainless steel
  - Geometry (diameterxthickness) = 20x2 mm

Supported by:

Federal Ministry
for Economic Affairs
and Energy

on the basis of a decision
by the German Bundestag

ZIM
Zentrales
Innovationsprogramm
Mittelstand

BY
