# Supplementary material for: Approach to Standardized Material Characterization of the Human Lumbopelvic System: Testing and Evaluation
Source: Bioengineering (Basel). 2025 Aug 11;12(8):862. doi: 10.3390/bioengineering12080862 (PMC12383908; doi:10.3390/bioengineering12080862)
Supplement: Supplementary file 1 [file bioengineering-12-00862-s001.zip › File S2 Designs and auxiliaries/Models/ATT_Preparation_V02.pdf]

Preparation table for axial tensile test clamp mount

HTWK
Leipzig University
of Applied Sciences
B
Faculty of
Civil Engineering

Title	ATT-Soft tissue-Preparation V02
Subject	Biomechanics-Testing
Revision	2019-10-23-001
Author	Gebhardt, Marc

Notes

Preparation table for clamp mount for axial tensile test of soft tissue specimen with 20 mm test length.

Manufacturing by Fused Deposition Modeling (FDM).
Tested with following settings:
- Nozzle = 0.4 mm
- Filament material = PLA
- Resolution = 0.2 mm
- Infill density = 50 %
- Print speed = 60 mm/s

Supported by:
Federal Ministry
for Economic Affairs
and Energy
on the basis of a decision
by the German Bundestag
ZIM
Zentrales
Innovationsprogramm
Mittelstand

cc BY
