# Supplementary material for: Approach to Standardized Material Characterization of the Human Lumbopelvic System: Testing and Evaluation
Source: Bioengineering (Basel). 2025 Aug 11;12(8):862. doi: 10.3390/bioengineering12080862 (PMC12383908; doi:10.3390/bioengineering12080862)
Supplement: Supplementary file 1 [file bioengineering-12-00862-s001.zip › File S2 Designs and auxiliaries/Three-point_bending_test_setup_004-004_220810.pdf]

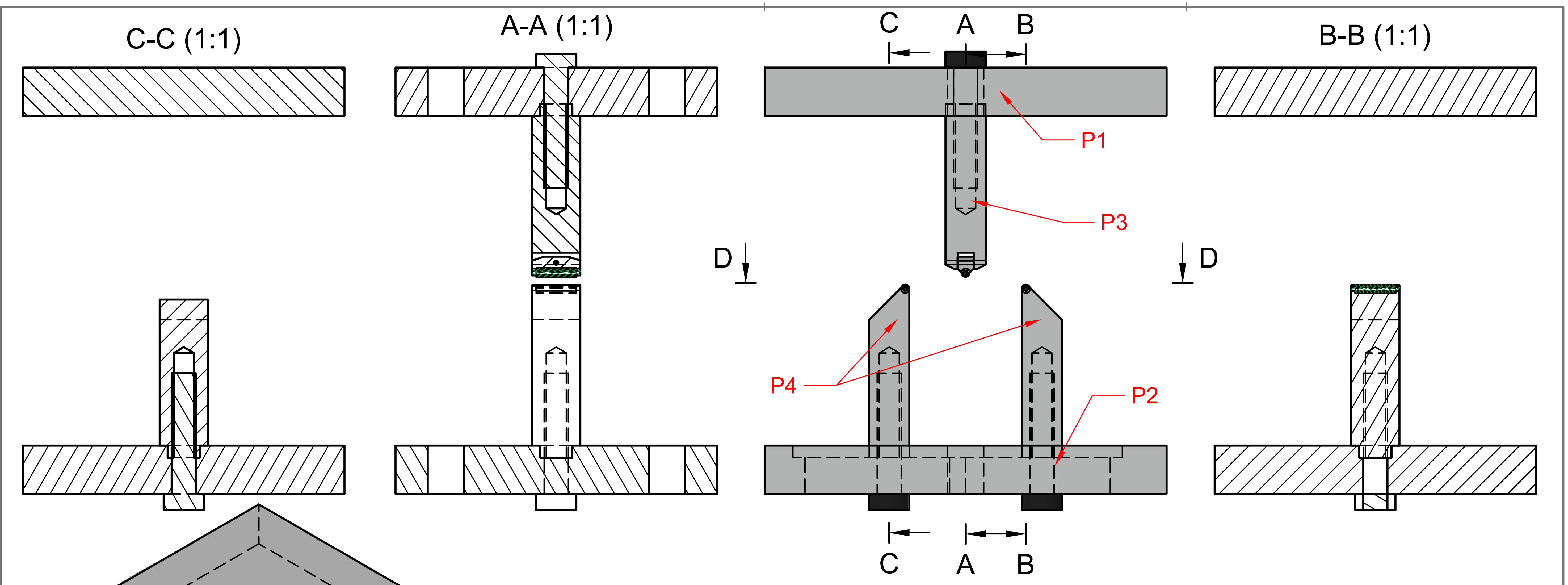

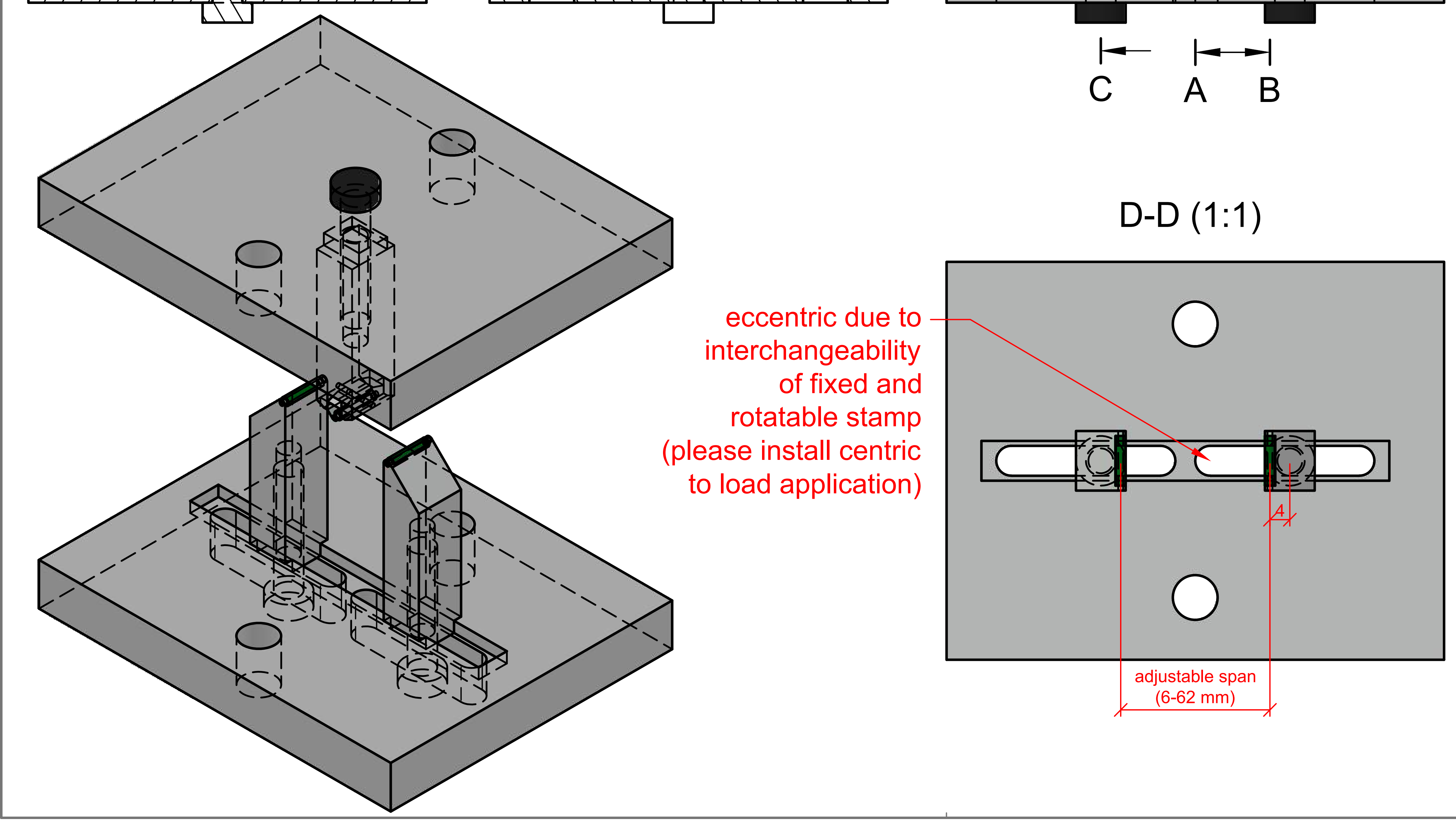

Parts

No.	Qty.	Description	Drawing sub no.
P1	1	Base plate top	*-001
P2	1	Base plate bottom	*-002
P3	1	Stamp rotatable	*-003
P4	2	Stamp fixed	*-004
P5	3	DIN 7984 M6-1.0 x 30 A2	-

Scale: -				Format: A3
Material: Stainless steel V2				
Description: Three-point bending test setup for cortical bone Overview				
Drawing number: PF004-004-000	Revision: 4	Original date: 27.02.2019	Revision date: 10.08.2022	
Drawer: Marc Gebhardt		Organisation: HTWK Leipzig University of Applied Sciences		

First published as supplementary information in: Gebhardt M, Steinke H, Slowik V. Determination of the Modulus of Elasticity by Bending Tests of Specimens with Nonuniform Cross Section. Exp Mech. 2023; 63:743–58. doi: 10.1007/s11340-023-00945-y.

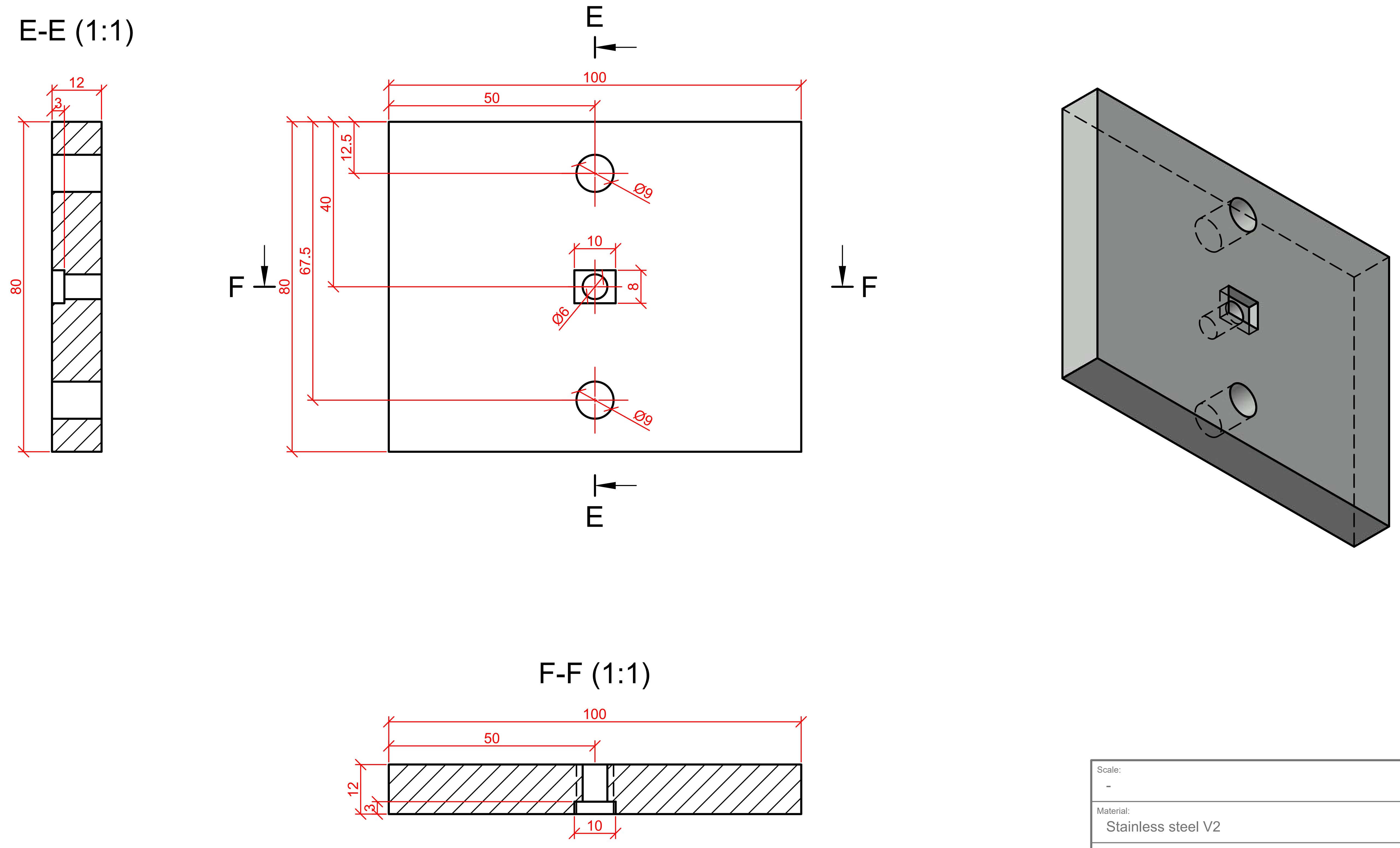

Scale:	Format:
-	A3

Material: Stainless steel V2			
Description: Three-point bending test setup for cortical bone<br>P1 - Base plate top			
Drawing number: PF004-004-001	Revision: 4	Original date: 27.02.2019	Revision date: 10.08.2022
Drawer: Marc Gebhardt		Organisation: HTWK Leipzig University of Applied Sciences	

First published as supplementary information in: Gebhardt M, Steinke H, Slowik V. Determination of the Modulus of Elasticity by Bending Tests of Specimens with Nonuniform Cross Section. Exp Mech. 2023; 63:743–58. doi: 10.1007/s11340-023-00945-y.

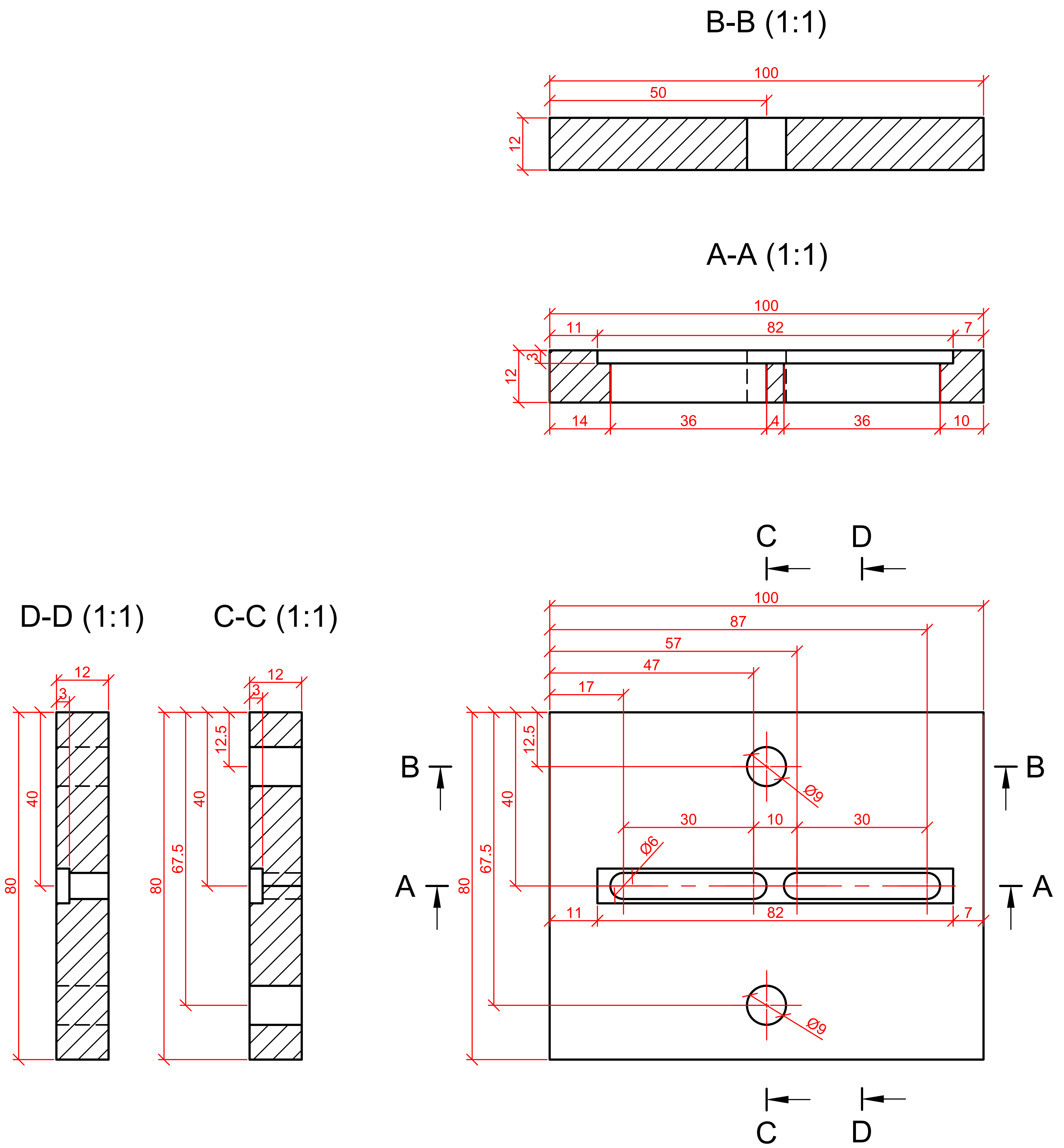

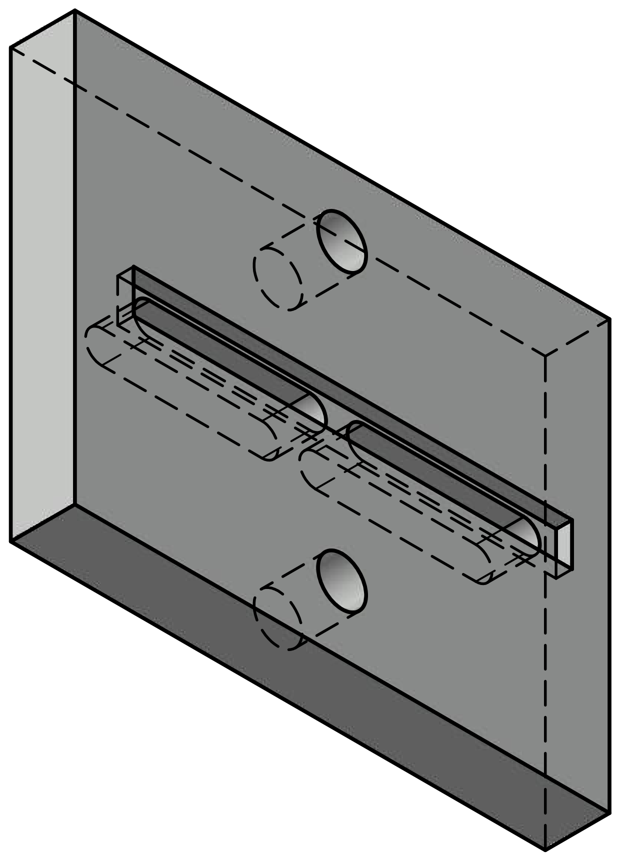

Scale:	Format:
-	A3

Material:	Stainless steel V2
Description:	Three-point bending test setup for cortical bone<br>P2 - Base plate bottom

Drawing number:	Revision:	Original date:	Revision date:
PF004-004-002	4	27.02.2019	10.08.2022

Drawer:	Organisation:
Marc Gebhardt	HTWK Leipzig University of Applied Sciences

First published as supplementary information in: Gebhardt M, Steinke H, Slowik V. Determination of the Modulus of Elasticity by Bending Tests of Specimens with Nonuniform Cross Section. Exp Mech. 2023; 63:743–58. doi: 10.1007/s11340-023-00945-y.

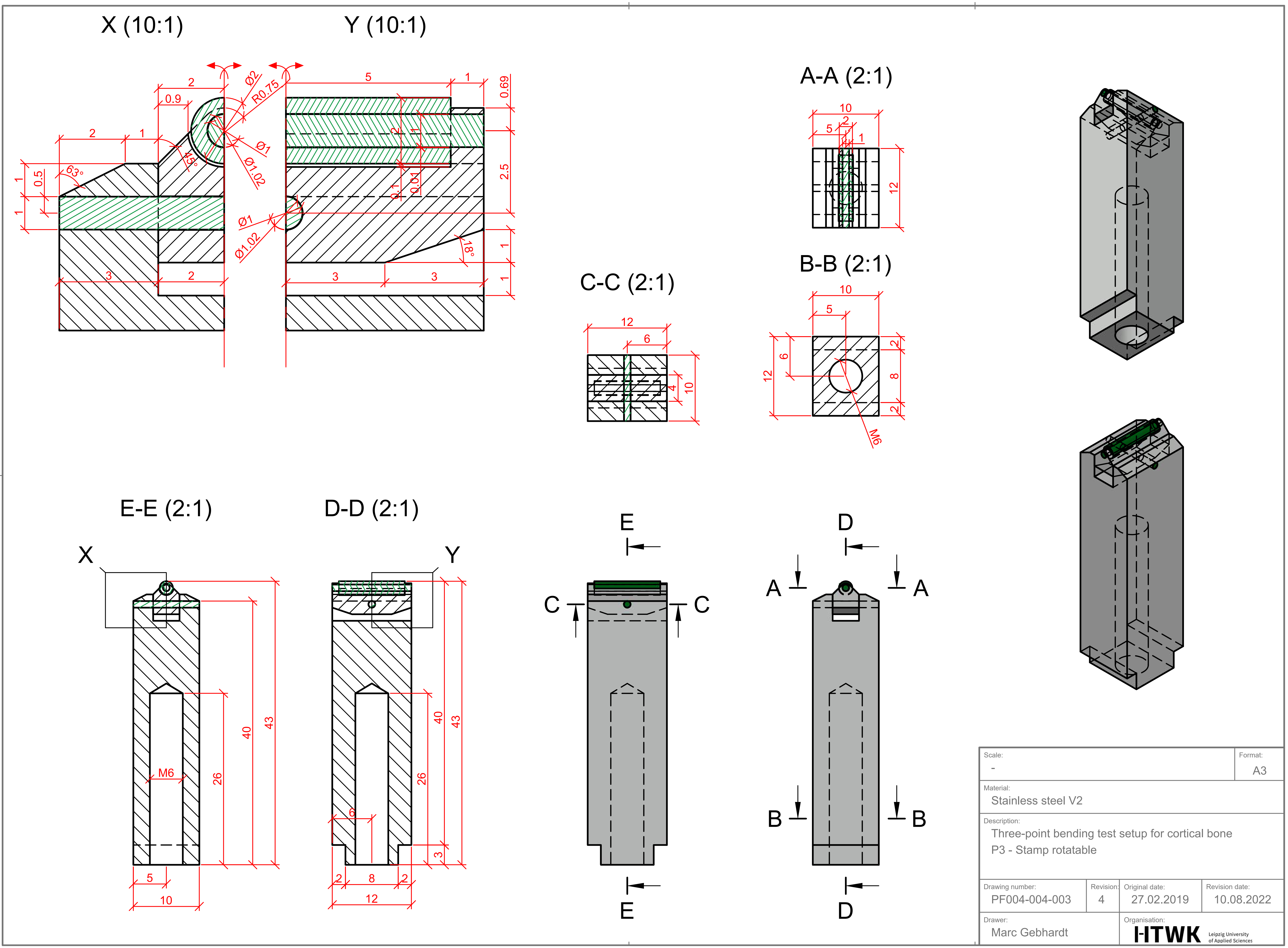

Scale: -	Format: A3
Material: Stainless steel V2	
Description: Three-point bending test setup for cortical bone<br>P3 - Stamp rotatable	

Drawing number:	Revision:	Original date:	Revision date:
PF004-004-003	4	27.02.2019	10.08.2022

Drawer: Marc Gebhardt

Organisation: HTWK Leipzig University of Applied Sciences

First published as supplementary information in: Gebhardt M, Steinke H, Slowik V. Determination of the Modulus of Elasticity by Bending Tests of Specimens with Nonuniform Cross Section. Exp Mech. 2023; 63:743–58. doi: 10.1007/s11340-023-00945-y.

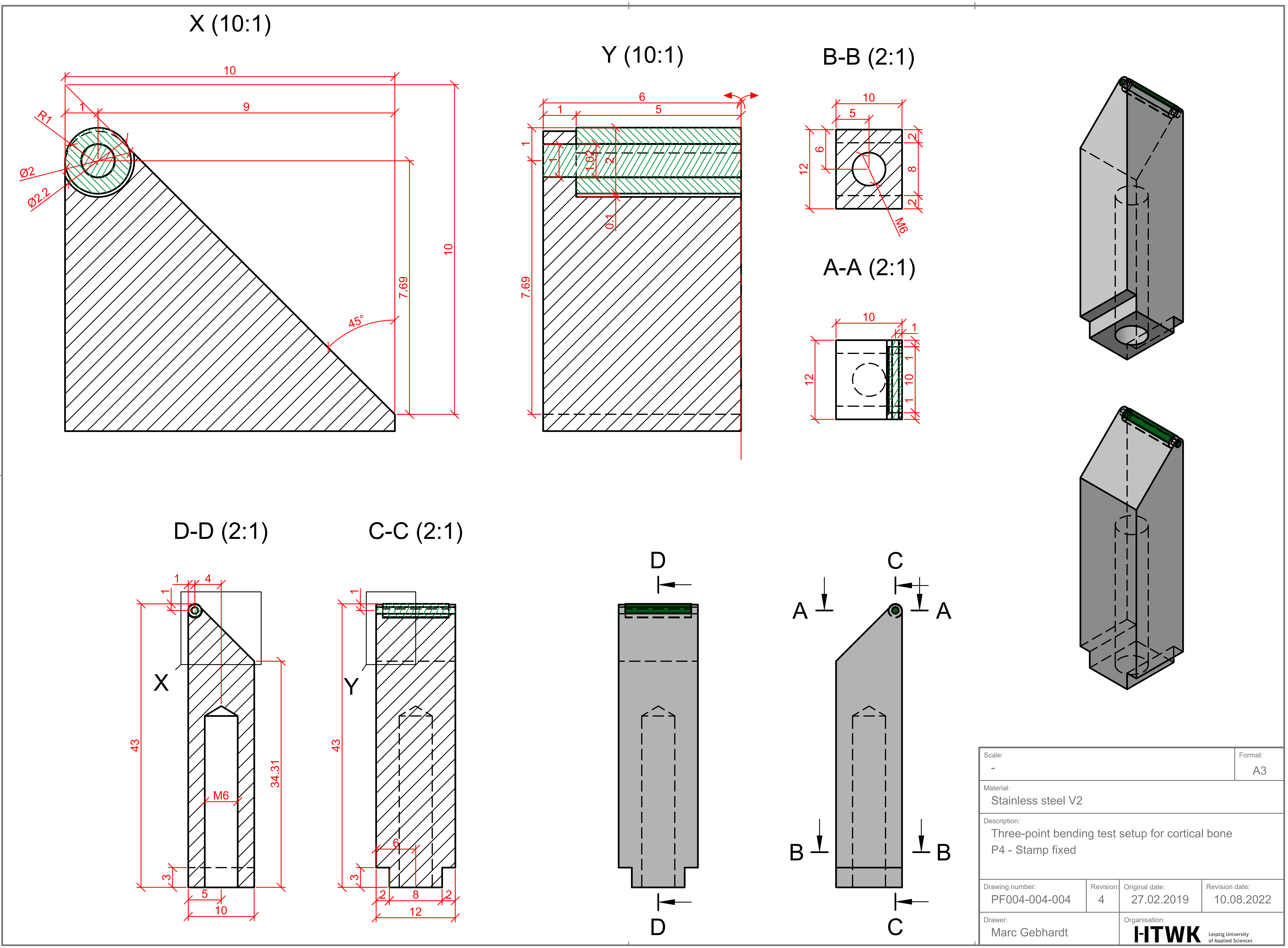

Scale:	Format:
-	A3

Material: Stainless steel V2

Description: Three-point bending test setup for cortical bone
P4 - Stamp fixed

Drawing number:	Revision:	Original date:	Revision date:
PF004-004-004	4	27.02.2019	10.08.2022

Drawer:	Organisation:
Marc Gebhardt	HTWK Leipzig University of Applied Sciences

First published as supplementary information in: Gebhardt M, Steinke H, Slowik V. Determination of the Modulus of Elasticity by Bending Tests of Specimens with Nonuniform Cross Section. Exp Mech. 2023; 63:743–58. doi: 10.1007/s11340-023-00945-y.

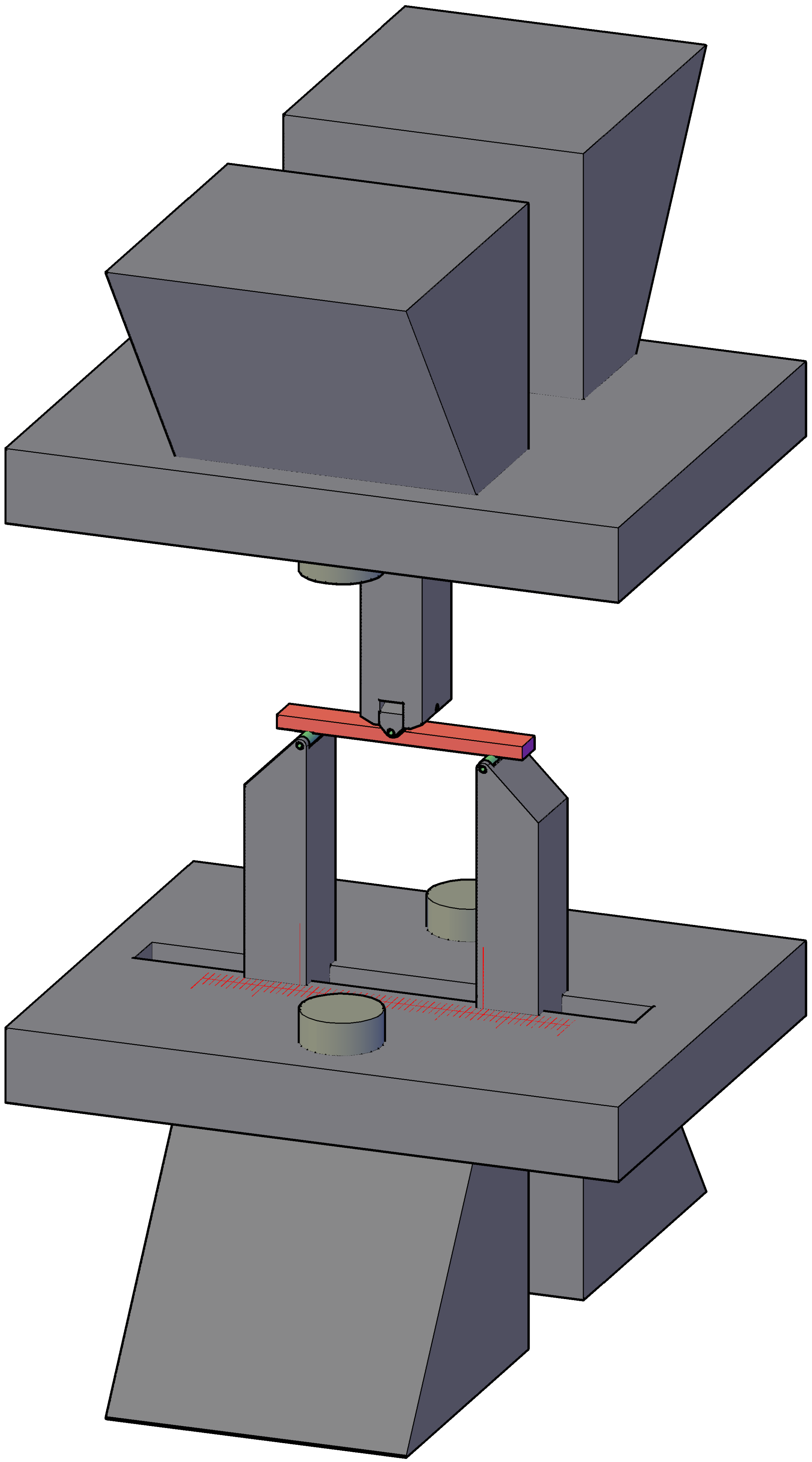

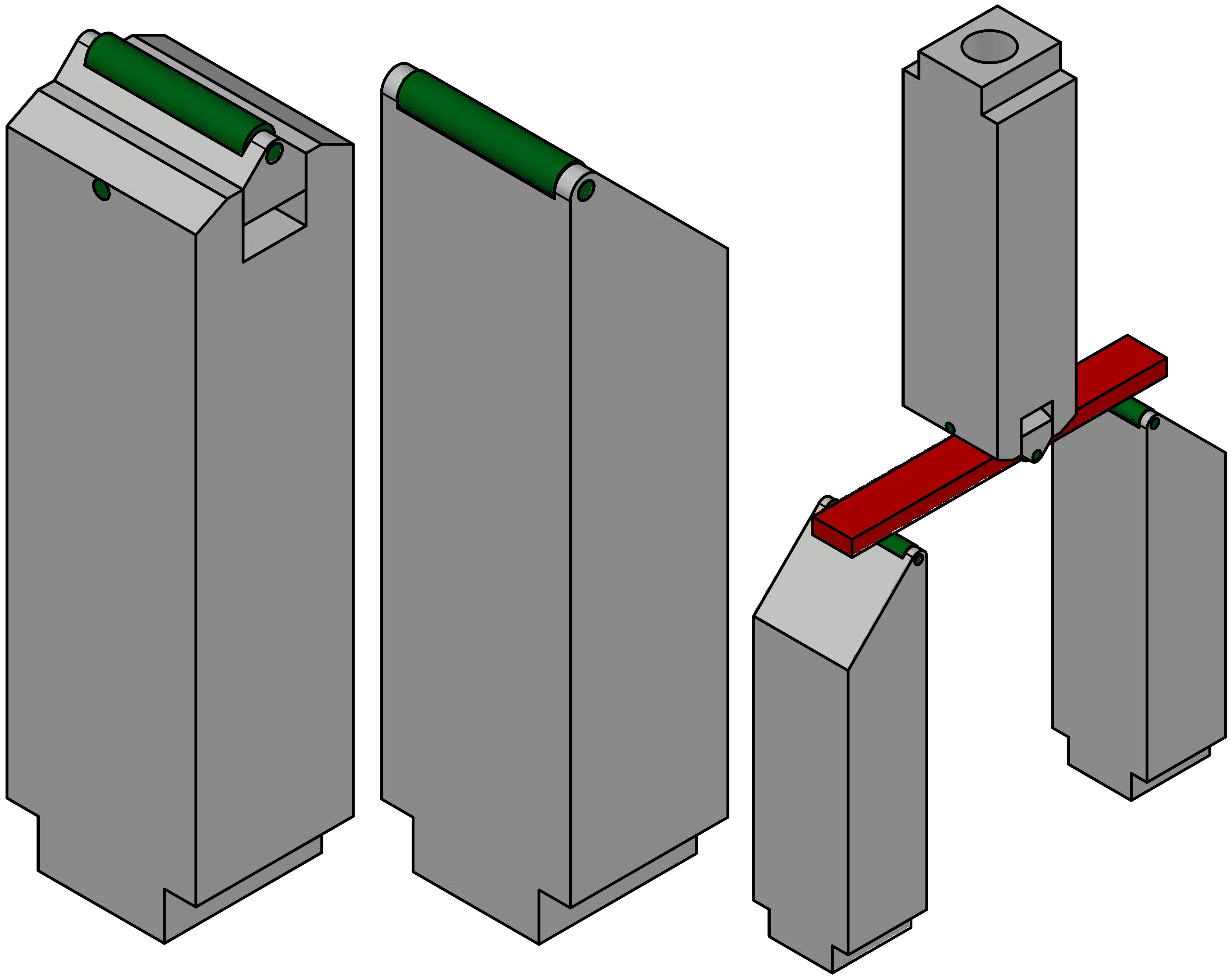

Scale: -	Format: A3
Material: Stainless steel V2	
Description: Three-point bending test setup for cortical bone<br>3D rendered view	

Drawing number:	Revision:	Original date:	Revision date:
PF004-004-010	4	27.02.2019	10.08.2022

Drawer: Marc Gebhardt

Organisation: HTWK Leipzig University of Applied Sciences

First published as supplementary information in: Gebhardt M, Steinke H, Slowik V. Determination of the Modulus of Elasticity by Bending Tests of Specimens with Nonuniform Cross Section. Exp Mech. 2023; 63:743–58. doi: 10.1007/s11340-023-00945-y.
