# Supplementary material for: Approach to Standardized Material Characterization of the Human Lumbopelvic System: Testing and Evaluation
Source: Bioengineering (Basel). 2025 Aug 11;12(8):862. doi: 10.3390/bioengineering12080862 (PMC12383908; doi:10.3390/bioengineering12080862)
Supplement: Supplementary file 1 [file bioengineering-12-00862-s001.zip › File S3 Evaluation code/ExMechEva-0.1.2/data/Test/ACT/Series_Test/eva/tl21x-YMRange_Imp.pdf]

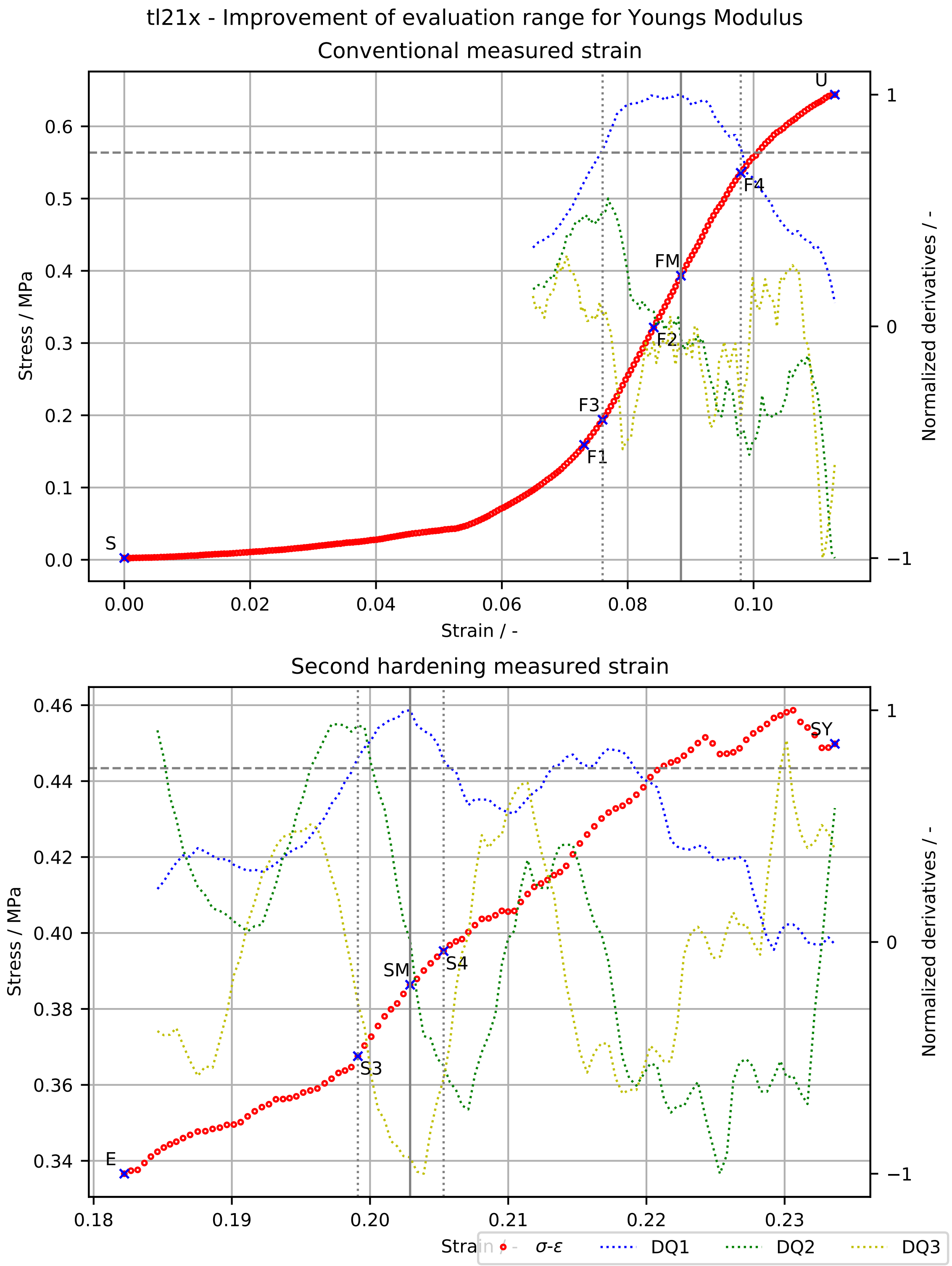

tl21x - Improvement of evaluation range for Youngs Modulus
Conventional measured strain
Stress / MPa
Normalized derivatives / -
Strain / -
S
F1
F3
F2
FM
F4
U
Second hardening measured strain
Stress / MPa
Normalized derivatives / -
Strain / -
E
S3
SM
S4
SY
σ-ε
DQ1
DQ2
DQ3
