# Supplementary material for: Approach to Standardized Material Characterization of the Human Lumbopelvic System: Testing and Evaluation
Source: Bioengineering (Basel). 2025 Aug 11;12(8):862. doi: 10.3390/bioengineering12080862 (PMC12383908; doi:10.3390/bioengineering12080862)
Supplement: Supplementary file 1 [file bioengineering-12-00862-s001.zip › File S3 Evaluation code/ExMechEva-0.1.2/docs/_build/html/exmecheva.bending.html]

exmecheva.bending package — ExMechEva v0.1.2 documentation


ExMechEva

Contents:

- ExMechEva
  - exmecheva package
    - Subpackages
      - exmecheva.bending package
        - Submodules
        - exmecheva.bending.attr\_bgl module
        - exmecheva.bending.bfunc\_class module
          - `Bend_func_cohort`
          - `Bend_func_legion`
          - `Bend_func_sub`
        - exmecheva.bending.bfunc\_com module
          - `Shear_area()`
          - `gamma_V_det()`
          - `triangle_func_d0()`
          - `triangle_func_d1()`
          - `triangle_func_d2()`
        - exmecheva.bending.bfunc\_fse module
          - `FSE_4sin_d0()`
          - `FSE_4sin_d1()`
          - `FSE_4sin_d2()`
          - `FSE_4sin_lin_func_d0()`
          - `FSE_4sin_lin_func_d1()`
          - `FSE_4sin_lin_func_d2()`
          - `FSE_4sin_wlin_d0()`
          - `FSE_4sin_wlin_d1()`
          - `FSE_4sin_wlin_d2()`
          - `FSE_SF_func_d0()`
          - `FSE_SF_func_d1()`
          - `FSE_SF_func_d2()`
        - exmecheva.bending.evaluation module
          - `Moment_perF_func()`
          - `Weight_func()`
          - `YM_check_many_with_method_D()`
          - `YM_check_with_method_D()`
          - `YM_eva_method_A()`
          - `YM_eva_method_B()`
          - `YM_eva_method_C()`
          - `YM_eva_method_D()`
          - `YM_eva_method_D_bend_df()`
          - `YM_eva_method_D_bend_df_add()`
          - `YM_eva_method_D_num()`
          - `YM_eva_method_D_res()`
          - `YM_eva_method_E()`
          - `YM_eva_method_F()`
          - `YM_eva_method_G()`
          - `coord_df_depo()`
          - `coord_df_mean()`
          - `straindf_from_curve()`
          - `stress_df_from_lin()`
          - `stress_perF()`
        - exmecheva.bending.fitting module
          - `Multi_minimize()`
          - `Perform_Fit()`
          - `lmfit_bound_checker()`
          - `lmfit_free_val_setter()`
          - `lmfit_modelize()`
          - `lmfit_param_adder()`
          - `lmfit_param_key_checker()`
          - `lmfit_param_prep()`
          - `res_multi_const_weighted()`
          - `shaped_array_fill_fandl()`
        - exmecheva.bending.opt\_mps module
          - `Point_df_combine()`
          - `Point_df_from_lin()`
          - `Point_df_idx()`
          - `Point_df_transform()`
          - `Points_add_step()`
          - `Points_dif_step()`
          - `Points_diff()`
          - `Points_eval_func()`
          - `v_Ctrans()`
          - `v_length()`
        - exmecheva.bending.plotting module
          - `colplt_common_ax()`
          - `colplt_df_ax()`
          - `colplt_funcs_all()`
          - `colplt_funcs_ax()`
          - `colplt_funcs_one()`
        - Module contents
      - exmecheva.common package
    - Submodules
    - exmecheva.Eva\_ACT module
    - exmecheva.Eva\_ATT module
    - exmecheva.Eva\_TBT module
    - exmecheva.eva module
    - Module contents

ExMechEva

- ExMechEva
- exmecheva package
- exmecheva.bending package
- View page source

---

# exmecheva.bending package

## Submodules

## exmecheva.bending.attr\_bgl module

Global attributes for bending modules.

@author: MarcGebhardt

## exmecheva.bending.bfunc\_class module

Classes for bending functions.

@author: MarcGebhardt

*class* exmecheva.bending.bfunc\_class.Bend\_func\_cohort(*d0=None*, *d1=None*, *d2=None*, *name=None*, *\*\*kws*)[source]
:   Bases: `object`

    Function collection of Subfunctions (Bend\_func\_sub)

    Init\_fandds(*expr\_d0*, *var\_names*, *var='x'*, *var\_types=None*, *option='d0\_str\_to\_all'*, *expr\_d1=None*, *expr\_d2=None*, *func\_d0=None*, *func\_d1=None*, *func\_d2=None*)[source]
    :   Return Sympy expressions and associated lambdified functions, depending on option-string.

        Parameters
        :   - **expr\_d0** (*string* *or* *sympy.expression*) – String or expression, which determine function.
            - **var\_names** (*TYPE*) – Variable names for parsing.
            - **var** (*TYPE**,* *optional*) – Variable name for derivation. The default is ‘x’.
            - **option** (*string**,* *optional*) – Option of function generation, in form of which(d0/each)\_type(str/expr)\_to\_what(d0/all). The default is ‘d0\_str\_to\_all’.
            - **expr\_d1** (*string* *or* *sympy.expression**,* *optional*) – String or expression, which determine 1st derivate of function. The default is None.
            - **expr\_d2** (*string* *or* *sympy.expression**,* *optional*) – String or expression, which determine 2nd derivate of function. The default is None.

        Raises
        :   **NotImplementedError** – Error for not implemented option.

        Returns
        :   **func\_package** – A function collection of all generated sympy expressions
            and associated lambdified functions and their 1st and 2nd derivates.

        Return type
        :   Bend\_func\_cohort

*class* exmecheva.bending.bfunc\_class.Bend\_func\_legion(*name=None*, *description=None*, *\*\*kws*)[source]
:   Bases: `object`

    Bend line describing functions and their 1st and 2nd derivates
    .. rubric:: Example

    bl=Bend\_func\_legion(name=’FSE fit’)
    bl.Builder(option=’FSE’)

    Builder(*option='FSE'*)[source]
    :   Build an instance of Bend per option to prepare fit of measured bend line.

        Parameters
        :   **option** (*string**,* *optional*) – Kind of . The default is ‘FSE’.

        Raises
        :   **NotImplementedError** – Building option not implemented.

        Return type
        :   None.

*class* exmecheva.bending.bfunc\_class.Bend\_func\_sub(*func*, *sy\_expr*, *sy\_string*, *independent\_vars=None*, *param\_names=None*, *param\_types=None*, *name=None*, *\*\*kws*)[source]
:   Bases: `object`

    Lowest element which provides necessary function informations.

    \_\_call\_\_(*x*, *kws*, *\*\*opts*)[source]
    :   Call and evaluate underlayed function.
        If x is an array of float and kws is a OrderedDict, result will be an array of float.
        If x is a Dataframe or float and kws is a Series, evaluates each index in kws.

        Parameters
        :   - **x** (*array* *of* *float* *|* *Dataframe* *of* *type Points*) – Independet variables to evaluate function.
            - **\*\*kws** (*OrderedDict* *|* *Series* *of* *OrderedDicts*) – Additional keyword arguments to pass to func
              (p.e. parameter value dictionary).

        Returns
        :   Evaluation values of function to x and kws.

        Return type
        :   array of float

## exmecheva.bending.bfunc\_com module

General functions for bending.

@author: MarcGebhardt

exmecheva.bending.bfunc\_com.Shear\_area(*Area*, *CS\_type='Rectangle'*, *kappa=None*)[source]
:   Returns the shear area of a cross section.

    Parameters
    :   - **Area** (*float* *or* *function* *or* *1*) – Cross section area.
        - **CS\_type** (*string**,* *optional*) – Cross section type. The default is ‘Rectangle’.
        - **kappa** (*float**,* *optional*) – Correction factor shear area. Depending to CS\_type. The default is None.

    Returns
    :   **AreaS** – Shear area.

    Return type
    :   same type as Area

exmecheva.bending.bfunc\_com.gamma\_V\_det(*poisson*, *t\_mean*, *Length*, *CS\_type='Rectangle'*, *kappa=None*)[source]
:   Returns the ratio between shear to entire deformation in mid of bending beam.

    Parameters
    :   - **poisson** (*float*) – Poisson’s ratio.
        - **t\_mean** (*float*) – Mean thickness.
        - **Length** (*float*) – Distance between load bearings.
        - **CS\_type** (*string**,* *optional*) – Cross section type. The default is ‘Rectangle’.
        - **kappa** (*float**,* *optional*) – Correction factor shear area. Depending to CS\_type. The default is None.

    Returns
    :   **gamma\_V** – Ratio between shear to entire deformation in mid of bending beam.

    Return type
    :   float

exmecheva.bending.bfunc\_com.triangle\_func\_d0(*x*, *xmin*, *xmax*, *f\_0*)[source]
:   Standard triangle function with maximum in middle between min. and max. x coordinate.

exmecheva.bending.bfunc\_com.triangle\_func\_d1(*x*, *xmin*, *xmax*, *f\_0*)[source]
:   First derivate of standard triangle function.

exmecheva.bending.bfunc\_com.triangle\_func\_d2(*x*, *xmin*, *xmax*, *f\_0*)[source]
:   Second derivate of standard triangle function (equal 0).

## exmecheva.bending.bfunc\_fse module

Specific fourier series expansion functions for bending.

@author: MarcGebhardt

exmecheva.bending.bfunc\_fse.FSE\_4sin\_d0(*x*, *xmin*, *xmax*, *FP*, *b1*, *b2*, *b3*, *b4*, *c=None*, *d=None*, *f\_V\_0=None*)[source]
:   Fourier series expansion with four sine elements without additional linear
    and constant element. For more information see FSE\_4sin\_wlin\_d0.

exmecheva.bending.bfunc\_fse.FSE\_4sin\_d1(*x*, *xmin*, *xmax*, *FP*, *b1*, *b2*, *b3*, *b4*, *c=None*, *d=None*, *f\_V\_0=None*)[source]
:   First deriavate of Fourier series expansion with four sine elements
    without additional linear and constant element. For more information see FSE\_4sin\_wlin\_d0.

exmecheva.bending.bfunc\_fse.FSE\_4sin\_d2(*x*, *xmin*, *xmax*, *FP*, *b1*, *b2*, *b3*, *b4*, *c=None*, *d=None*, *f\_V\_0=None*)[source]
:   Second deriavate of Fourier series expansion with four sine elements
    without additional linear and constant element. For more information see FSE\_4sin\_wlin\_d0.

exmecheva.bending.bfunc\_fse.FSE\_4sin\_lin\_func\_d0(*x*, *c*, *d*, *xmin=None*, *xmax=None*, *FP=None*, *b1=None*, *b2=None*, *b3=None*, *b4=None*, *f\_V\_0=None*)[source]
:   Linear part of fourier series expansion with four sine elements and
    additional linear and constant element. For more information see FSE\_4sin\_wlin\_d0.

exmecheva.bending.bfunc\_fse.FSE\_4sin\_lin\_func\_d1(*x*, *c*, *d=None*, *xmin=None*, *xmax=None*, *FP=None*, *b1=None*, *b2=None*, *b3=None*, *b4=None*, *f\_V\_0=None*)[source]
:   First deriavate of linear part of fourier series expansion with four sine elements and
    additional linear and constant element. For more information see FSE\_4sin\_wlin\_d0.

exmecheva.bending.bfunc\_fse.FSE\_4sin\_lin\_func\_d2(*x*, *c=None*, *d=None*, *xmin=None*, *xmax=None*, *FP=None*, *b1=None*, *b2=None*, *b3=None*, *b4=None*, *f\_V\_0=None*)[source]
:   Second deriavate of linear part of fourier series expansion with four sine elements and
    additional linear and constant element. For more information see FSE\_4sin\_wlin\_d0. (Equal 0)

exmecheva.bending.bfunc\_fse.FSE\_4sin\_wlin\_d0(*x*, *xmin*, *xmax*, *FP*, *b1*, *b2*, *b3*, *b4*, *c*, *d*, *f\_V\_0=None*)[source]
:   Fourier series expansion with four sine elements and additional linear and constant element.
    Defined as:

    \[f(x; b1,b2,b3,b4,b5,c,d) = b1 \sin (\pi / (xmax-xmin) (x-xmin)) + b2 \sin (2 \pi / (xmax-xmin) (x-xmin)) + b3 \sin (3 \pi / (xmax-xmin) (x-xmin)) + b4 \sin (4 \pi / (xmax-xmin) (x-xmin)) +c (x-xmin) + d\]

    with sine-parameters for \(b1,b2,b3,b4\), slope for \(c\) and intercept for \(d\).

    Parameters
    :   - **x** (*array* *of* *float*) – Array of cartesian coordinates in x-direction.
        - **xmin** (*float*) – Cartesian coordinate in x-direction of start of function (curvature=0, p.e. left bearing).
          Defines length of Fourier series expansion with xmax-xmin.
        - **xmax** (*float*) – Cartesian coordinate in x-direction of end of function (curvature=0, p.e. right bearing).
          Defines length of Fourier series expansion with xmax-xmin.
        - **b1** (*float*) – Parameter of first sine element.
        - **b2** (*float*) – Parameter of second sine element.
        - **b3** (*float*) – Parameter of third sine element.
        - **b4** (*float*) – Parameter of fourth sine element.
        - **c** (*float*) – Parameter of slope.
        - **d** (*float*) – Parameter of intercept.

    Returns
    :   **eq** – Fourier series expansion with four sine elements and additional linear and constant element.

    Return type
    :   Function

exmecheva.bending.bfunc\_fse.FSE\_4sin\_wlin\_d1(*x*, *xmin*, *xmax*, *FP*, *b1*, *b2*, *b3*, *b4*, *c*, *d=None*, *f\_V\_0=None*)[source]
:   First derivate of fourier series expansion with four sine elements and
    additional linear and constant element. For more information see FSE\_4sin\_wlin\_d0.

exmecheva.bending.bfunc\_fse.FSE\_4sin\_wlin\_d2(*x*, *xmin*, *xmax*, *FP*, *b1*, *b2*, *b3*, *b4*, *c=None*, *d=None*, *f\_V\_0=None*)[source]
:   Second derivate of fourier series expansion with four sine elements and
    additional linear and constant element. For more information see FSE\_4sin\_wlin\_d0.

exmecheva.bending.bfunc\_fse.FSE\_SF\_func\_d0(*x*, *xmin*, *xmax*, *f\_V\_0*, *FP=None*, *b1=None*, *b2=None*, *b3=None*, *b4=None*, *c=None*, *d=None*, *opt=None*)[source]
:   Shear deformation part of ourier series expansion with four sine elements
    and additional linear and constant element. Children of triangle\_func\_d0.
    For more information see FSE\_4sin\_wlin\_d0.

exmecheva.bending.bfunc\_fse.FSE\_SF\_func\_d1(*x*, *xmin*, *xmax*, *f\_V\_0*, *FP=None*, *b1=None*, *b2=None*, *b3=None*, *b4=None*, *c=None*, *d=None*, *opt=None*)[source]
:   First derivate of shear deformation part of ourier series expansion with
    four sine elements and additional linear and constant element.
    Children of triangle\_func\_d1. For more information see FSE\_4sin\_wlin\_d0.

exmecheva.bending.bfunc\_fse.FSE\_SF\_func\_d2(*x*, *xmin*, *xmax*, *f\_V\_0*, *FP=None*, *b1=None*, *b2=None*, *b3=None*, *b4=None*, *c=None*, *d=None*, *opt=None*)[source]
:   Second derivate of shear deformation part of ourier series expansion with
    four sine elements and additional linear and constant element.
    Children of triangle\_func\_d1. For more information see FSE\_4sin\_wlin\_d0.

## exmecheva.bending.evaluation module

Evaluation functionality for bending.

@author: MarcGebhardt

exmecheva.bending.evaluation.Moment\_perF\_func(*x*, *xmin*, *xmax*, *Test\_type='TPB'*)[source]
:   Returns a by force scaleable moment function.

exmecheva.bending.evaluation.Weight\_func(*x*, *option='Triangle'*, *c\_func=None*, *\*\*kwargs*)[source]
:   Returns a weighting function by given options and parameters.

    Parameters
    :   - **x** (*float*) – Coordinate in x direction.
        - **option** (*string**,* *optional*) –

          Choosen option.
          Possible are:

          > - ’Cut’: Excluding values outside range of xmin to xmax
          >   :   (weight equal 0).
          > - ’Triangle’: Weighing to triangle function with maximum in the
          >   :   middle between xmin and xmax.
          > - ’Triangle\_cut’: Mixture of ‘Triangle’ and ‘Cut’.
          > - ’Custom’: Weighing to custom function (p.e. displacement funtion).
          > - ’Custom\_cut’: Mixture of ‘Custom’ and ‘Cut’.

          The default is ‘Triangle’.
        - **c\_func** (*function**,* *optional*) – Custom function for weighing (p.e. displacement funtion).
          The default is None.
        - **\*\*kwargs** (*dict*) –

          Keyword arguments for custom function (p.e. displacement
          :   function parameters).

    Raises
    :   **NotImplementedError** – Option not implemented.

    Returns
    :   **eq** – Weights.

    Return type
    :   float

exmecheva.bending.evaluation.YM\_check\_many\_with\_method\_D(*E\_dict*, *F*, *Length*, *I\_func*, *w\_func*, *w\_params*, *rel\_steps=None*, *n=100*, *pb\_b=True*, *name='X'*)[source]
:   Compares the deformation of the analytical bending line (method D),
    scaled by elastic modulus determined by methods, with the measured bending
    line.
    Returns global mean, local (midspan) mean and complete (n-positions)
    deviation, as well as the scaled analytical bending line, for each
    specified step.

    Parameters
    :   - **E\_dict** (*dict*) – Dictionary of moduli of elasticity by determination method.
        - **F** (*pandas.Series*) – Series of force (or force increment).
        - **Length** (*float*) – Testing length.
        - **I\_func** (*TYPE*) – Function of Moment of Inertia.
        - **w\_func** (*Bend\_func\_sub*) – Bending function.
        - **w\_params** (*pd.Series* *of* *dict*) – Parameters per step for bending function (w\_func).
        - **rel\_steps** (*pandas.Index* *or* *numpy array**,* *optional*) – Relevant steps. The default is None.
        - **n** (*integer**,* *optional*) – Number of determination points. The default is 100.
        - **pb\_b** (*boolean**,* *optional*) – Switch progress bar output. The default is True.
        - **name** (*string**,* *optional*) – Name of executed check. The default is ‘X’.

    Returns
    :   - **check\_EtoD\_g** (*pandas.Dataframe*) – Global (total length) mean deviation of scaled analytical and measured deformation.
        - **check\_EtoD\_x** (*pandas.Dataframe*) – Local (midspan) mean deviation of scaled analytical and measured deformation.
        - **check\_E** (*dictionary of pandas.Dataframe*) – Deviation of scaled analytical and measured deformation.
        - **w\_D\_to\_E** (*dictionary of pandas.Dataframe*) – Scaled analytical defeormation by method.

exmecheva.bending.evaluation.YM\_check\_with\_method\_D(*E*, *F*, *Length*, *I\_func*, *w\_vgl\_df*, *pb\_b=True*, *name='X'*)[source]
:   Compares the deformation of the analytical bending line (method D),
    scaled by elastic modulus determined by methods, with the measured bending
    line.

    Parameters
    :   - **E** (*float*) – Elastic modulus.
        - **F** (*float*) – Force (or force increment).
        - **Length** (*float*) – Testing length.
        - **I\_func** (*TYPE*) – Function of Moment of Inertia.
        - **w\_func** (*Bend\_func\_sub*) – Bending function.
        - **w\_params** (*pd.Series* *of* *dict*) – Parameters per step for bending function (w\_func).
        - **rel\_steps** (*pandas.Index* *or* *numpy array**,* *optional*) – Relevant steps. The default is None.
        - **n** (*integer**,* *optional*) – Number of determination points. The default is 100.
        - **pb\_b** (*boolean**,* *optional*) – Switch progress bar output. The default is True.
        - **name** (*string**,* *optional*) – Name of executed check. The default is ‘X’.

    Returns
    :   - **check\_E** (*pandas.Dataframe*) – Deviation of scaled analytical and measured deformation.
        - **w\_D\_to\_E** (*pandas.Dataframe*) – Scaled analytical defeormation.

exmecheva.bending.evaluation.YM\_eva\_method\_A(*stress\_mid\_ser*, *strain\_mid\_ser*, *comp=True*, *name='A'*, *det\_opt='incremental'*, *\*\*kws*)[source]
:   Calculates Young’s Modulus by rise of stress to strain in midspan
    over defined range with definable method.
    Children of Evac.YM\_eva\_com\_sel.

    Parameters
    :   - **stress\_mid\_ser** (*pd.Series*) – Series with stress values in midspan corresponding strain\_ser.
        - **strain\_mid\_ser** (*pd.Series*) – Series with strain values in midspan corresponding stress\_ser.
        - **comp** (*boolean**,* *optional*) – Compression mode. The default is True.
        - **name** (*string**,* *optional*) – Name of operation. The default is ‘A’.
        - **det\_opt** (*TYPE**,* *optional*) – Definable method for determination.
          Ether incremental or leastsq. The default is ‘incremental’.
        - **\*\*kws** (*dict*) – Keyword dict for least-square determination.

    Returns
    :   - *det\_opt == “incremental”* –

          YM\_serpd.Series
          :   Series of Young’s Moduli.
        - *or*
        - *det\_opt == “leastsq”* –

          YMfloat
          :   Youngs Modulus (corresponds to slope of linear fit).

          YM\_absfloat
          :   Stress value on strain origin (corresponds to interception of linear fit).

          YM\_Rquadfloat
          :   Coefficient of determination.

          YM\_fitlmfit.model.ModelResult
          :   Fitting result from lmfit (use with fit.fit\_report() for report).

exmecheva.bending.evaluation.YM\_eva\_method\_B(*stress\_mid\_ser*, *thickness*, *Length*, *option='Points'*, *P\_df=None*, *P\_fork\_names=None*, *w\_func=None*, *w\_params=None*, *Length\_det=None*, *comp=True*, *name='B'*, *det\_opt='incremental'*, *\*\*kws*)[source]
:   Calculates Young’s Modulus by rise of stress to strain,
    calculated by optical counterpart to traditional fork transducer,
    over defined range with definable method.

    Parameters
    :   - **stress\_mid\_ser** (*pandas.Series*) – Series with stress values in midspan corresponding strain\_ser.
        - **thickness** (*np.poly1d function*) – Function of thickness to span.
        - **Length** (*float*) – Length of span.
        - **option** (*string**,* *optional*) – Input option, ether “Points”, for a Dataframe of three points, or
          “Fit”, for a fitted bend line.
          The default is “Points”.
        - **P\_df** (*pandas.DataFrame**,* *optional*) – Dataframe of Points. The default is None.
        - **P\_fork\_names** (*array* *of* *string**,* *optional*) – Names of points in P\_df in form of [left, mid, right].
          The default is None.
        - **w\_func** (*function**,* *optional*) – Function of bending line. The default is None.
        - **w\_params** (*array* *or* *pandas.Series**,* *optional*) – Parameters of function of bending line per step. The default is None.
        - **Length\_det** (*float**,* *optional*) – Determination length between left and right point to calculate.
          The default is None.
        - **comp** (*boolean**,* *optional*) – Compression mode.The default is True.
        - **name** (*string**,* *optional*) – Name of operation. The default is ‘B’.
        - **det\_opt** (*TYPE**,* *optional*) – Definable method for determination.
          Ether incremental or leastsq. The default is ‘incremental’.
        - **\*\*kws** (*dict*) – Keyword dict for least-square determination.

    Raises
    :   - **ValueError** – Error in combination of option and inputs.
        - **NotImplementedError** – Error if option not implemented.

    Returns
    :   - *det\_opt == “incremental”* –

          YM\_serpd.Series
          :   Series of Young’s Moduli.
        - *or*
        - *det\_opt == “leastsq”* –

          YMfloat
          :   Youngs Modulus (corresponds to slope of linear fit).

          YM\_absfloat
          :   Stress value on strain origin (corresponds to interception of linear fit).

          YM\_Rquadfloat
          :   Coefficient of determination.

          YM\_fitlmfit.model.ModelResult
          :   Fitting result from lmfit (use with fit.fit\_report() for report).

exmecheva.bending.evaluation.YM\_eva\_method\_C(*Force\_ser*, *w\_func*, *w\_params*, *length*, *I\_func*, *A\_func=None*, *CS\_type='Rectangle'*, *kappa=None*, *poisson=0.3*, *comp=True*, *option='M+V'*, *name='C'*)[source]
:   Calculates the Young’s modulus from the deformation energy as a
    function of the internal forces (pure geometric fitting function).

    Parameters
    :   - **Force\_ser** (*pandas.Series*) – Series of force increments.
        - **w\_func** (*function*) – Function of bending line.
        - **w\_params** (*pandas.Series* *of* *dictionionaries*) – Parameters of function of bending line per step.
        - **length** (*float*) – Length of span.
        - **I\_func** (*np.poly1d function*) – Function of Moment of inertia to position on span (x-direction).
        - **A\_func** (*np.poly1d function**,* *optional*) – Function of Area to position on span (x-direction).
        - **CS\_type** (*string**,* *optional*) – Cross-section type. The default is ‘Rectangle’.
        - **kappa** (*float**,* *optional*) – Correction factor shear area. Depending to CS\_type.
          The default is None.
        - **poisson** (*float**,* *optional*) – Poisson’s ratio. The default is 0.3.
        - **comp** (*boolean**,* *optional*) – Compression mode.The default is True.
        - **option** (*string**,* *optional*) – Determiantion option. Possible are ‘M’ and ‘M+V’. The default is “M+V”.
        - **name** (*string**,* *optional*) – Name of operation. The default is “F”.

    Returns
    :   **E\_ser** – Series of determined Young’s Moduli.

    Return type
    :   pandas.Series

exmecheva.bending.evaluation.YM\_eva\_method\_D(*P\_df*, *Force\_ser*, *Length*, *func\_I*, *n=100*, *rel\_steps=None*, *weighted=False*, *weight\_func=<function Weight\_func>*, *wkwargs={}*, *wargs=[]*, *pb\_b=True*, *name='D'*)[source]
:   Calculates the modulus of elasticity by matching the theoretical
    displacements from the determined integral of the bending line with
    the measured ones.

    Parameters
    :   - **P\_df** (*pandas.DataFrame*) – Dataframe of measured points to steps.
        - **Force\_ser** (*pandas.Series*) – Series of force increments.
        - **Length** (*float*) – Length of span.
        - **func\_I** (*np.poly1d function*) – Function of Moment of inertia to position on span (x-direction).
        - **n** (*integer**,* *optional*) – Division of the length for calculation. The default is 100.
        - **rel\_steps** (*index* *or* *array**,* *optional*) – Relevant steps for determination. The default is None.
        - **weighted** (*boolean**,* *optional*) – Switch for weighted averaging. The default is True.
        - **weight\_func** (*function**,* *optional*) – Weighting function for global averaging. The default is Weight\_func.
        - **wargs** (*array**,* *optional*) – Arguments for weighting function for global averaging.
          The default is [].
        - **wkwargs** (*dictionary**,* *optional*) – Keyword arguments for weighting function for global averaging.
          The default is {}.
        - **pb\_b** (*boolean**,* *optional*) – Switch of progressbar. The default is True.
        - **name** (*string**,* *optional*) – Name of operation. The default is ‘D’.

    Returns
    :   - **YM\_ser** (*pandas.Series*) – Series of determined Young’s Moduli.
        - **YM\_df** (*pandas.DataFrame*) – DataFrame of determined Young’s Moduli to x-position.

exmecheva.bending.evaluation.YM\_eva\_method\_D\_bend\_df(*Length*, *I\_func*, *n=100*, *E=1*, *F=1*)[source]
:   Calculates the deflection values via the determined integral of the
    bending line.

    Parameters
    :   - **Length** (*float*) – Length of span.
        - **I\_func** (*np.poly1d function*) – Function of Moment of inertia to position on span (x-direction).
        - **n** (*integer**,* *optional*) – Division of the length for calculation. The default is 100.
        - **E** (*float**,* *optional*) – Young’s Modulus. The default is 1.
        - **F** (*float**,* *optional*) – Force. The default is 1.

    Returns
    :   **m** – Dataframe of deflections and parts of partwise integration to
        x-positions.

    Return type
    :   pandas.DataFrame

exmecheva.bending.evaluation.YM\_eva\_method\_D\_bend\_df\_add(*points\_x*, *m\_df*, *Length*, *I\_func*, *E=1*, *F=1*)[source]
:   Adds additional points to the calculated deflection values via the
    determined integral of the bending line.

    Parameters
    :   - **points\_x** (*pd.Series*) – X-coordinates of additional points.
        - **m\_df** (*pandas.DataFrame*) – Dataframe of deflections and parts of partwise integration to x-positions.
        - **Length** (*float*) – Length of span.
        - **I\_func** (*np.poly1d function*) – Function of Moment of inertia to position on span (x-direction).
        - **E** (*float**,* *optional*) – Young’s Modulus. The default is 1.
        - **F** (*float**,* *optional*) – Force. The default is 1.

    Returns
    :   **p\_df** – Dataframe of deflections and parts of partwise integration to x-positions.

    Return type
    :   pandas.DataFrame

exmecheva.bending.evaluation.YM\_eva\_method\_D\_num(*P\_df*, *Force\_ser*, *step\_range*, *Length*, *func\_I*, *weighted=True*, *weight\_func=<function Weight\_func>*, *wargs=[]*, *wkwargs={}*, *max\_nfev=500*, *pb\_b=True*, *name='D'*)[source]
:   Evaluate elastic modulus by minimization to method D residuals.

    Parameters
    :   - **P\_df** (*pandas.DataFrame*) – Dataframe of measured points to steps.
        - **Force\_ser** (*pandas.Series*) – Series of force increments.
        - **step\_range** (*pd.Index* *or* *range* *or* *list*) – Evaluation range.
        - **Length** (*float*) – Length of span.
        - **func\_I** (*np.poly1d function*) – Function of Moment of inertia to position on span (x-direction).
        - **weighted** (*boolean**,* *optional*) – Switch for weighted averaging. The default is True.
        - **weight\_func** (*function**,* *optional*) – Weighting function for global averaging. The default is Weight\_func.
        - **wargs** (*array**,* *optional*) – Arguments for weighting function for global averaging.
          The default is [].
        - **wkwargs** (*dictionary**,* *optional*) – Keyword arguments for weighting function for global averaging.
          The default is {}.
        - **max\_nfev** (*int**,* *optional*) – Maximum number of iterations. The default is 500.
        - **pb\_b** (*boolean**,* *optional*) – Switch of progressbar. The default is True.
        - **name** (*string**,* *optional*) – Name of operation. The default is ‘D’.

    Returns
    :   **D\_df** – Evaluated elastic modulus and coefficent of determination per step.

    Return type
    :   pd.DataFrame

exmecheva.bending.evaluation.YM\_eva\_method\_D\_res(*E*, *x\_data*, *y\_data=None*, *weights=None*, *\*\*kws*)[source]
:   Calculate residues of method D.

exmecheva.bending.evaluation.YM\_eva\_method\_E(*Force\_ser*, *length*, *func\_curve*, *params\_curve*, *func\_MoI*, *func\_thick*, *evopt=0.5*, *opt\_det='all'*, *n=100*, *opt\_g='length'*, *opt\_g\_lim=0.5*, *weight\_func=<function Weight\_func>*, *wargs=[]*, *wkwargs={}*, *name='E'*)[source]
:   Calculates Young’s Modulus by rise of stress to strain,
    calculated by curvature (2nd derivate) of bending line,
    per step with definable method.

    Parameters
    :   - **Force\_ser** (*pandas.Series*) – Series of force increments.
        - **length** (*float*) – Length of span.
        - **func\_curve** (*function*) – Function of curvature (2nd derivate) of bending line. The default is None.
        - **params\_curve** (*array* *or* *pandas.Series* *of* *dictionionaries*) – Parameters of function of curvature (2nd derivate) of bending line per step..
        - **func\_MoI** (*np.poly1d function*) – Function of Moment of inertia to position on span (x-direction).
        - **func\_thick** (*np.poly1d function*) – Function of thickness to position on span (x-direction).
        - **evopt** (*float**,* *optional*) – Position according thickness for strain calculation. The default is 1/2.
        - **opt\_det** (*string**,* *optional*) –

          Determination option.
          possible are:

          > - ’all’: All options evaluated.
          > - ’stress’ : Local evaluation on maximum stress.
          > - ’strain’ : Local evaluation on maximum strain.
          > - ’range’ : Global evaluation over range defined by opt\_g.

          The default is ‘all’.
        - **n** (*integer**,* *optional*) – Division of the length for calculation. The default is 100.
        - **opt\_g** (*string**,* *optional*) –

          Option for global determination.
          possible are:

          > - ’length’: Averaging by range (opt\_g\_lim\*length) around midspan.
          > - ’strain’: Averaging by range (strain<=opt\_g\_lim\*strain\_max).
          > - ’Moment\_weighted’: Weighted averaging by moment function.
          > - ’Custom\_weighted’: Weighted averaging by custom function.

          The default is ‘length’.
        - **opt\_g\_lim** (*float**,* *optional*) – Limiter for opt\_g. The default is 0.5.
        - **weight\_func** (*function**,* *optional*) – Weighting function for global averaging. The default is Weight\_func.
        - **wargs** (*array**,* *optional*) – Arguments for weighting function for global averaging.
          The default is [].
        - **wkwargs** (*dictionary**,* *optional*) – Keyword arguments for weighting function for global averaging.
          The default is {}.
        - **name** (*string**,* *optional*) – Name of operation. The default is ‘E’.

    Raises
    :   **NotImplementedError** – Raise error if option not implemented.

    Returns
    :   - **E\_df** (*pandas.DataFrame*) – Dataframe of determined Young’s Moduli.
        - **sig\_eps\_df** (*pandas.DataFrame*) – Dataframe of stress and strain values used in determination.
        - **E\_to\_x** (*TYPE*) – Dataframe of determined Young’s Moduli to span position (x-direction).
        - **stress\_df** (*pandas.DataFrame*) – Dataframe of stress values.
        - **strain\_df** (*pandas.DataFrame*) – Dataframe of strain values.
        - **E\_to\_x\_g** (*pandas.DataFrame*) – Dataframe of determined Young’s Moduli to span position (x-direction)
          for global averaging.

exmecheva.bending.evaluation.YM\_eva\_method\_F(*c\_func*, *c\_params*, *Force\_ser*, *Length*, *func\_I*, *weighted=True*, *weight\_func=<function Weight\_func>*, *wargs=[]*, *wkwargs={}*, *xr\_dict={'fu': 1.0*, *'ha': 0.5*, *'th': 0.3333333333333333}*, *pb\_b=True*, *name='F'*, *n=100*)[source]
:   Calculates the Young’s modulus via the local application of
    the differential equation of the bending line.

    Parameters
    :   - **c\_func** (*function*) – Function of curvature (2nd derivate) of bending line.
        - **c\_params** (*pandas.Series* *of* *dictionionaries*) – Parameters of function of curvature (2nd derivate) of bending line
          per step.
        - **Force\_ser** (*pandas.Series*) – Series of force increments.
        - **Length** (*float*) – Length of span.
        - **func\_I** (*Tnp.poly1d function*) – Function of Moment of inertia to position on span (x-direction).
        - **weighted** (*boolean**,* *optional*) – Switch for weighted averaging. The default is True.
        - **weight\_func** (*function**,* *optional*) – Weighting function for global averaging. The default is Weight\_func.
        - **wargs** (*array**,* *optional*) – Arguments for weighting function for global averaging.
          The default is [].
        - **wkwargs** (*dictionary**,* *optional*) – Keyword arguments for weighting function for global averaging.
          The default is {}.
        - **xr\_dict** (*dictionary**,* *optional*) – Dictionary of name to range of length around midspan for determination.
          The default is {‘fu’:1/1, ‘ha’:1/2, ‘th’:1/3}.
        - **pb\_b** (*boolean**,* *optional*) – Switch of progressbar. The default is True.
        - **name** (*string**,* *optional*) – Name of operation. The default is ‘F’.
        - **n** (*integer**,* *optional*) – Division of the length for calculation. The default is 100.

    Returns
    :   **YM\_df** – Series of determined Young’s Moduli.

    Return type
    :   pandas.Series

exmecheva.bending.evaluation.YM\_eva\_method\_G(*Force\_ser*, *w\_func\_f\_0*, *w\_params*, *c\_func*, *r\_func*, *c\_params*, *r\_params*, *length*, *I\_func*, *A\_func=None*, *CS\_type='Rectangle'*, *kappa=None*, *poisson=0.3*, *comp=True*, *option='M+V'*, *name='G'*)[source]
:   Calculates Young’s Moduli via the approach of equality of external work
    and deformation energy.

    Parameters
    :   - **Force\_ser** (*pandas.Series*) – Series of force increments.
        - **w\_func\_f\_0** (*function*) – Function of bending line for external work calculation.
        - **w\_params** (*pandas.Series* *of* *dictionionaries*) – Parameters of function of bending line per step for external
          work calculation.
        - **c\_func** (*function*) – Function of curvature (2nd derivate) of bending line.
        - **r\_func** (*function*) – Function of rise (1st derivate) of bending line.
        - **c\_params** (*pandas.Series* *of* *dictionionaries*) – Parameters of function of curvature (2nd derivate) of bending
          line per step.
        - **r\_params** (*pandas.Series* *of* *dictionionaries*) – Parameters of function of rise (1st derivate) of bending line per step.
        - **length** (*float*) – Length of span.
        - **I\_func** (*np.poly1d function*) – Function of Moment of inertia to position on span (x-direction).
        - **A\_func** (*np.poly1d function**,* *optional*) – Function of Area to position on span (x-direction).
          The default is None.
        - **CS\_type** (*string**,* *optional*) – Cross-section type. The default is ‘Rectangle’.
        - **kappa** (*float**,* *optional*) – Correction factor shear area. Depending to CS\_type.
          The default is None.
        - **poisson** (*float**,* *optional*) – Poisson’s ratio. The default is 0.3.
        - **comp** (*boolean**,* *optional*) – Compression mode.The default is True.
        - **option** (*string**,* *optional*) – Determiantion option. Possible are ‘ignore\_V’ and ‘M+V’.
          The default is “M+V”.
        - **name** (*string**,* *optional*) – Name of operation. The default is “G”.

    Returns
    :   Series of determined Young’s Moduli.

    Return type
    :   pandas.Series

exmecheva.bending.evaluation.coord\_df\_depo(*df*, *name=''*, *pos=0.0*)[source]
:   Return values on postion (Default x=0.0)

exmecheva.bending.evaluation.coord\_df\_mean(*df*, *name=''*, *fex=1*, *lex=1*)[source]
:   Return mean values.

exmecheva.bending.evaluation.straindf\_from\_curve(*x*, *func\_curve*, *params\_curve*, *func\_thick*, *evopt=0.5*)[source]
:   Evaluates the combination of a curvature and thickness function to x
    on a thickness ratio. (Use only for bending)
    Returns a dataframe with steps as index and x as columns.

exmecheva.bending.evaluation.stress\_df\_from\_lin(*F*, *x*, *func\_MoI*, *func\_thick*, *xmin*, *xmax*, *evopt=0.5*, *Test\_type='TPB'*)[source]
:   Returns bending stress values according given x coordinates.

exmecheva.bending.evaluation.stress\_perF(*x*, *func\_MoI*, *func\_thick*, *xmin*, *xmax*, *evopt=0.5*, *Test\_type='TPB'*)[source]
:   Returns a by force scaleable bending stress function.

## exmecheva.bending.fitting module

Fitting functionality for bending.

@author: MarcGebhardt

exmecheva.bending.fitting.Multi\_minimize(*x*, *data*, *params*, *func\_d0*, *func\_d2*, *max\_nfev*, *nan\_policy*, *err\_weights*, *x\_lB*, *x\_rB*, *load\_dir='-y'*)[source]
:   Performs a weighted multiconstraint least-square-fit, based on lmfit.minimize.
    Returns lmfit-result, parameter-dict, Coefficient of determination for multi-errors and only displacement as well.

    Parameters
    :   - **x** (*array* *of* *float*) – Cartesian coordinate in x-direction.
        - **data** (*array* *of* *float**,* *optional*) – Cartesian coordinate in y-direction. The default is None.
        - **params** (*OrderedDict / dict / array*) – Parameters to be used in functions.
        - **func\_d0** (*lamopdified function*) – Input function.
        - **func\_d2** (*lamopdified function*) – Second derivate (curvature) of input function.
        - **max\_nfev** (*int*) – Maximum number of function evaluations.
        - **err\_weights** (*array* *of* *float**,* *optional*) – Error weights assigned to constraints.
          Enter [1,0,0,0] for standard residual fit on displacement.
          The default is [1,10,100,100].
        - **x\_lB** (*float*) – Cartesian coordinate in x-direction of start of function (curvature=0, p.e. left bearing).
        - **x\_rB** (*float*) – Cartesian coordinate in x-direction of end of function (curvature=0, p.e. right bearing).
        - **load\_dir** (*string**,* *optional*) –

          Direction of displacement application.
          Possible are:

          > - ”-y”: application in negative y-direction (standard, curvature positve(err2))
          > - ”+y”: application in negative y-direction (curvature negative(err2))

    Returns
    :   **MG\_multi\_minimize\_Dict** – Fit result dictionary (lmfit-result, parameter-dict, Coefficient of determination for multi-errors and only displacement as well.).

    Return type
    :   dict

exmecheva.bending.fitting.Perform\_Fit(*BFL*, *Fit\_func\_key*, *P\_df*, *lB*, *rB*, *s\_range*, *Shear\_func\_key=None*, *gamma\_V=None*, *err\_weights=[1, 10, 1000, 100]*, *max\_nfev=500*, *nan\_policy='raise'*, *option='Pre'*, *ldoption='fixed-y'*, *ldoptionadd=None*, *pb\_b=True*, *\*\*pwargs*)[source]
:   Performs a weighted multiconstraint least-square-fit, based on lmfit.minimize.

    Parameters
    :   - **BFL** (*Bend\_func\_legion*) – Class conttaining information about bending line and derivates.
          See Bend\_func\_legion in ./bfunc\_class.py for more information.
        - **Fit\_func\_key** (*string*) – Identifier for fit function, p.e. ‘w\_A’.
        - **P\_df** (*pd.DataFrame*) – Measured and 2D-transformed displacment data. Combination of
          x-coordinates and y-displacments.
        - **lB** (*float*) – Left support x-coordinate, p.e. -10.0 with 20 mm span.
        - **rB** (*float*) – Right support x-coordinate, p.e. 10.0 with 20 mm span.
        - **s\_range** (*list* *or* *index*) – Range of steps.
        - **Shear\_func\_key** (*string**,* *optional*) – Identifier for shear function, p.e. ‘w\_S’. The default is None.
        - **gamma\_V** (*float**,* *optional*) – Ratio between shear to entire deformation in mid of bending beam.
          See gamma\_V\_det in ./bfunc\_com.py
          The default is None.
        - **err\_weights** (*list* *of* *float**,* *optional*) – Weights for multi contraint fitting. See res\_multi\_const\_weighted.
          The default is [ 1, 10, 1000, 100].
        - **max\_nfev** (*int**,* *optional*) – Number of evaluations. The default is 500.
        - **nan\_policy** (*string**,* *optional*) – NaN handling. The default is ‘raise’.
        - **option** (*string**,* *optional*) –

          Option for fitting. Possible are:
          :   - ’Pre’: Pre fit with shear deformation
              - ’Bend’: Refit to adjusted bending deformation

              (without indentation and shear deformation)

          The default is ‘Pre’.
        - **ldoption** (*string**,* *optional*) –

          Load direction automatism.
          possible are:

          > - ’fixed-y’: Load application in -y-direction (default).
          > - ’fixed+y’: Load application in +y-direction.
          > - ’auto-dispser’: Load application direction automaticly determined
          >
          > by a series of displacements (applied as ldoptionadd,
          > index have to match with s\_range/P\_df).
          > - ‘auto-Pcoorddisp’: Load application direction automaticly
          > determined by a Points dataframe and specified point name and
          > coordinate (applied as ldoptionadd, index have to match with
          > s\_range/P\_df)

          The default is ‘fixed-y’.
        - **ldoptionadd** (*Series* *or* *array* *of* *[**Dataframe**,* *string**,* *string**]**,* *optional*) –

          Addendum for ldoption.
          Have to match to ldoption:

          > - ’auto-dispser’: Series of displacements (index have to match with
          >
          > s\_range/P\_df).
          > - ‘auto-Pcoorddisp’: Points dataframe and specified point name and
          > coordinate ([Points as Dataframe, point name as string, coordinate
          > as string],index have to match with s\_range/P\_df)

          The default is None.
        - **pb\_b** (*bool**,* *optional*) – Switch for showing progressbar. The default is True.
        - **\*\*pwargs** (*dict* *or* *pandas.Series*) – Parameter keyword arguments for function.

    Raises
    :   **NotImplementedError** – Option not implemented.

    Returns
    :   **Fit\_res\_df** – Data of fit results.

    Return type
    :   pandas.DatFrame

exmecheva.bending.fitting.lmfit\_bound\_checker(*Fit\_Result*, *BFs*, *param\_check\_types=['free']*, *level=1*)[source]
:   Check if lmfit Fit Result excides or hits bounds.

exmecheva.bending.fitting.lmfit\_free\_val\_setter(*bfs*, *param\_val={}*, *default\_val=-1.0*)[source]
:   Preset free values/parameters for lmfit.

exmecheva.bending.fitting.lmfit\_modelize(*bfs*, *option='init'*)[source]
:   Returns a lmfit.model defined by Bend function sub.

exmecheva.bending.fitting.lmfit\_param\_adder(*par\_df*)[source]
:   Returns lmfit Parameterset defined by pandas Dataframe.

exmecheva.bending.fitting.lmfit\_param\_key\_checker(*bfs*, *param\_dict*)[source]
:   Check parameters of lmfit with Bend\_func\_sub.

exmecheva.bending.fitting.lmfit\_param\_prep(*option*, *param\_name*, *param\_val=None*, *param\_type=None*, *param\_min=None*, *param\_max=None*)[source]
:   Prepare fit parameters for fitting.

exmecheva.bending.fitting.res\_multi\_const\_weighted(*params*, *x*, *func*, *func\_d2*, *x\_lB*, *x\_rB*, *func\_err\_weight=[1, 10, 100, 100]*, *load\_dir='-y'*, *data=None*)[source]
:   Returns weighted error sum according to different constraints between
    input values and function values.
    Constraints:

    > 0. error between data and function value
    > 1. error between function value of left and right bound to zero
    > 2. error between negative/positive value of second derivate of function and zero (depends on load direction)
    > 3. error between value of second derivate of function of left and right bound to zero

    Parameters
    :   - **params** (*OrderedDict / dict / array*) – Parameters to be used in functions.
        - **x** (*array* *of* *float*) – Cartesian coordinate in x-direction.
        - **func** (*lamopdified function*) – Input function.
        - **func\_d2** (*lamopdified function*) – Second derivate (curvature) of input function.
        - **x\_lB** (*float*) – Cartesian coordinate in x-direction of start of function (curvature=0, p.e. left bearing).
        - **x\_rB** (*float*) – Cartesian coordinate in x-direction of end of function (curvature=0, p.e. right bearing).
        - **func\_err\_weight** (*array* *of* *float**,* *optional*) – Error weights assigned to constraints.
          Enter [1,0,0,0] for standard residual fit on displacement.
          The default is [1,10,100,100].
        - **load\_dir** (*string**,* *optional*) –

          Direction of displacement application.
          Possible are:

          > - ”-y”: application in negative y-direction (standard, curvature positve(err2))
          > - ”+y”: application in negative y-direction (curvature negative(err2))
        - **data** (*array* *of* *float**,* *optional*) – Cartesian coordinate in y-direction. The default is None.

    Returns
    :   **err** – Weighted multi constraint error sum.

    Return type
    :   numpy array

exmecheva.bending.fitting.shaped\_array\_fill\_fandl(*ShapeAr*, *fElemV*, *lElemV*)[source]
:   Returns an 1D numpy array with shape of input array and filled with ones.
    First and last element replaced with input data.

    Parameters
    :   - **ShapeAr** (*numpy.array*) – Input array wich determine shape of output array.
        - **fElemV** (*float64*) – Value of first element passed to output array.
        - **lElemV** (*float64*) – Value of last element passed to output array.

    Returns
    :   **OutAr** – 1D numpy array with shape of input array and filled with ones.
        First and last element replaced with input data.

    Return type
    :   numpy array

## exmecheva.bending.opt\_mps module

Adds funcionality for optical measured cartesian points.
(Mostly vector geometry)

@author: MarcGebhardt

exmecheva.bending.opt\_mps.Point\_df\_combine(*df1*, *df2*, *coords\_set='y'*, *deepcopy=True*, *option=None*)[source]
:   Combines a deepcopy of one points dataframe to an other.

exmecheva.bending.opt\_mps.Point\_df\_from\_lin(*x*, *steps*, *coords=['x', 'y']*, *coords\_set='x'*, *col\_type='str'*, *Point\_prefix='L'*)[source]
:   Prepare a points dataframe with index of step and values of x.

exmecheva.bending.opt\_mps.Point\_df\_idx(*df*, *steps=None*, *points=None*, *coords=None*, *deepcopy=True*, *option=None*)[source]
:   Indexing a points dataframe by step (index), point-names and coordinates.

exmecheva.bending.opt\_mps.Point\_df\_transform(*Pmeas*, *Pspec*, *Pmeas\_Sdev*, *Pspec\_Sdev*, *dic\_P\_name\_org1*, *dic\_P\_name\_org2*, *output\_lvl=0*, *log\_mg=''*)[source]
:   Calculate in plane transformation of given measured 3D-points.

    Parameters
    :   - **Pmeas** (*pandas.Dataframe**(**[**]**,* *index=**[**'x'**,**'y'**,**'z'**]**,*) – columns=[‘P1’,’P2’,…], dtype=float64)
          Cartesian coordinates of measured points, which should be fitted.
        - **Pspec** (*pandas.Dataframe**(**[**]**,* *index=**[**'x'**,**'y'**,**'z'**]**,*) – columns=[‘S1’,’S2’,…], dtype=float64)
          Cartesian coordinates of special points which should used for
          coordinate transformation.
        - **Pmeas\_Sdev** (*pandas.Dataframe**(**[**]**,* *index=**[**'Sx'**,**'Sy'**,**'Sz'**]**,*) – columns=[‘P1’,’P2’,…], dtype=float64)
          Standard deviation of measured points, which should be fitted.
        - **Pspec\_Sdev** (*pandas.Dataframe**(**[**]**,* *index=**[**'Sx'**,**'Sy'**,**'Sz'**]**,*) – columns=[‘S1’,’S2’,…], dtype=float64)
          Standard deviation of special points which should used for coordinate
          transformation.
        - **dic\_P\_name\_org1** (*str*) – Name of point, which should be on the negativ x-axis.
          Origin is in the middle between dic\_P\_name\_org1 and dic\_P\_name\_org2.
        - **dic\_P\_name\_org2** (*str*) – Name of point, which should be on the positiv x-axis.
          Origin is in the middle between dic\_P\_name\_org1 and dic\_P\_name\_org2.

    Returns
    :   - *Points\_T* – Transformed coordinates and standard deviation of given points.
        - *Points\_L\_T* – In plane transformed coordinates and standard deviation of given points.

exmecheva.bending.opt\_mps.Points\_add\_step(*df*, *add\_step=0*, *add\_df=None*, *steps=None*, *points=None*, *coords=None*, *deepcopy=True*, *option=None*)[source]
:   Adding a Points df to an other.

exmecheva.bending.opt\_mps.Points\_dif\_step(*df*, *dif\_step=0*, *steps=None*, *points=None*, *coords=None*, *deepcopy=True*, *option=None*)[source]
:   Substracts a Points df from an other.

exmecheva.bending.opt\_mps.Points\_diff(*df*, *diff\_coord='y'*, *first\_val=0*, *steps=None*, *points=None*, *coords=None*, *deepcopy=True*, *option=None*, *nan\_policy='corr\_nan'*)[source]
:   Returns differential of specified coordinates of Points.

exmecheva.bending.opt\_mps.Points\_eval\_func(*func*, *fit\_params*, *pointdf*, *steps*, *in\_coord='x'*, *out\_coord='y'*, *points=None*, *deepcopy=True*, *option=None*)[source]
:   Evaluates a dataframe or series by a function.

exmecheva.bending.opt\_mps.v\_Ctrans(*v*, *TM*, *P0*)[source]
:   Transform a set of vectors(v=ixN3) with a translationmatrix (TM) and a new origin (P0)

exmecheva.bending.opt\_mps.v\_length(*v1*)[source]
:   Computes the length of an vector

## exmecheva.bending.plotting module

Plotting functionality for bending.

@author: MarcGebhardt

exmecheva.bending.plotting.colplt\_common\_ax(*xdata*, *ydata*, *step\_range=None*, *title=''*, *xlabel=''*, *ylabel=''*, *xstep=False*, *ystep=True*, *Point\_df=None*, *ax=None*, *cblabel='Step'*, *cbtick=None*)[source]
:   Returns a matpltlib axis plot of a pandas Dataframe of type points in a range.

exmecheva.bending.plotting.colplt\_df\_ax(*df*, *step\_range=None*, *title=''*, *xlabel=''*, *ylabel=''*, *Point\_df=None*, *ax=None*, *cblabel='Step'*, *cbtick=None*)[source]
:   Returns a matpltlib axis plot of a pandas Dataframe of type points in a range.

exmecheva.bending.plotting.colplt\_funcs\_all(*x*, *func\_cohort*, *params*, *step\_range=None*, *title=''*, *xlabel=''*, *Point\_df=None*, *cblabel='Step'*, *cbtick=None*, *path=None*, *plt\_scopt={'clear': True, 'close': True, 's\_types': ['pdf'], 'save': True, 'show': True, 'tight': True}*)[source]
:   Returns a matpltlib axis plot of a function cohort
    (function and theire first and second derivate)
    with defined parameters in a range.

exmecheva.bending.plotting.colplt\_funcs\_ax(*x*, *func*, *params*, *step\_range=None*, *title=''*, *xlabel=''*, *ylabel=''*, *Point\_df=None*, *ax=None*, *cblabel='Step'*, *cbtick=None*)[source]
:   Returns a matpltlib axis plot of a function with defined parameters in a range.

exmecheva.bending.plotting.colplt\_funcs\_one(*x*, *func*, *params*, *step\_range=None*, *title=''*, *xlabel=''*, *ylabel=''*, *Point\_df=None*, *cblabel='Step'*, *cbtick=None*, *path=None*, *plt\_scopt={'clear': True, 'close': True, 's\_types': ['pdf'], 'save': True, 'show': True, 'tight': True}*)[source]
:   Returns a matpltlib figure plot of a function with defined
    parameters in a range.

## Module contents

Functionality for exmecheva bending evaluation.

Previous
Next

---

© Copyright 2024, MarcGebhardt.

Built with Sphinx using a
theme
provided by Read the Docs.
