# Supplementary material for: Approach to Standardized Material Characterization of the Human Lumbopelvic System: Testing and Evaluation
Source: Bioengineering (Basel). 2025 Aug 11;12(8):862. doi: 10.3390/bioengineering12080862 (PMC12383908; doi:10.3390/bioengineering12080862)
Supplement: Supplementary file 1 [file bioengineering-12-00862-s001.zip › File S3 Evaluation code/ExMechEva-0.1.2/docs/_build/html/exmecheva.common.html]

exmecheva.common package — ExMechEva v0.1.2 documentation


ExMechEva

Contents:

- ExMechEva
  - exmecheva package
    - Subpackages
      - exmecheva.bending package
      - exmecheva.common package
        - Submodules
        - exmecheva.common.analyze module
          - `Geo_curve_TBC()`
          - `Inter_Lines()`
          - `Line_from2P()`
          - `TP_circle()`
          - `TP_radius()`
          - `normalize()`
          - `normalize_th()`
          - `sign_n_change()`
          - `sign_n_changeth()`
          - `threshhold_setter()`
        - exmecheva.common.eva\_opt\_hand module
          - `com_option_file_read()`
          - `com_option_file_write()`
          - `option_presetter()`
          - `option_reader()`
          - `option_reader_sel()`
          - `set_type_by_string()`
        - exmecheva.common.fitting module
          - `Refit_YM_vals()`
          - `Rquad()`
          - `YM_eva_com_sel()`
          - `YM_sigeps_lin()`
          - `fit_report_adder()`
          - `func_exp()`
          - `func_exp_str()`
          - `func_lin()`
          - `func_lin_str()`
          - `func_pow()`
          - `func_pow_str()`
          - `regfitret()`
          - `regfitret_restring_func()`
          - `strain_linfit()`
          - `stress_linfit()`
          - `stress_linfit_plt()`
        - exmecheva.common.helper module
          - `check_empty()`
          - `round_to_sigdig()`
          - `sigdig()`
          - `str_to_bool()`
          - `type_str_return()`
        - exmecheva.common.list\_ops module
          - `Failure_code_bool_df()`
          - `Failure_code_checker()`
          - `Failure_code_format()`
          - `Failure_code_lister()`
          - `ICD_bool_df()`
          - `ICD_lister()`
          - `list_boolean_df()`
          - `list_cell_compiler()`
          - `list_interpreter()`
          - `list_ser_to_1D()`
        - exmecheva.common.loadnsave module
          - `comb_logs()`
          - `file_namer()`
          - `file_namer_interpreter()`
          - `pack_hdf()`
          - `pack_hdf_mul()`
        - exmecheva.common.mc\_char module
          - `Diff_Quot()`
          - `Diff_Quot2()`
          - `Diff_Quot3()`
          - `YM_eva_range_refine()`
          - `curve_characterizer()`
          - `curve_merger()`
          - `curvecar_refine()`
          - `curvecar_section()`
          - `find_SandE()`
          - `peaky_finder()`
          - `peaky_finder_MM()`
          - `poi_det_plh()`
          - `poi_fixeva()`
          - `poi_refinement()`
          - `poi_rel_finder()`
          - `poi_vip_namer()`
          - `rise_curve()`
          - `test_pdmon()`
        - exmecheva.common.mc\_man module
          - `DetFinSSC()`
          - `Diff_ext()`
          - `Extend_Series_Poly()`
          - `Extend_Series_n_setter()`
          - `Predict_apply_retrim()`
          - `Retrim_Series()`
          - `Smoothsel()`
          - `Smoothsel_ext()`
          - `check_params()`
          - `mc_resampler()`
          - `smooth()`
        - exmecheva.common.mc\_yield module
          - `Find_intg2p()`
          - `YM_eva_range_refine()`
          - `Yield_redet()`
          - `Yield_redet2()`
          - `Yield_redet2_Multi()`
        - exmecheva.common.output module
          - `Otvalgetter_Multi()`
          - `Outvalgetter()`
          - `str_indent()`
          - `str_log()`
        - exmecheva.common.pd\_ext module
          - `Find_closest()`
          - `Find_closest_perc()`
          - `Find_closestv()`
          - `Find_first_sc()`
          - `deal_dupl_index()`
          - `pd_axischange()`
          - `pd_combine_index()`
          - `pd_exclnan()`
          - `pd_find_index()`
          - `pd_isDF()`
          - `pd_isSer()`
          - `pd_limit()`
          - `pd_nan_handler()`
          - `pd_outsort()`
          - `pd_slice_index()`
          - `pd_trapz()`
          - `pd_valid_index()`
          - `pd_vec_length()`
        - exmecheva.common.plotting module
          - `curve_char_plotter()`
          - `plt_add_DaAnno()`
          - `plt_ax_regfit()`
          - `plt_handle_suffix()`
          - `sns_pointplot_MMeb()`
          - `tick_label_inserter()`
          - `tick_label_renamer()`
          - `tick_legend_renamer()`
        - exmecheva.common.stat\_ext module
          - `CD_rep()`
          - `CD_test_multi()`
          - `CImax()`
          - `CImin()`
          - `Corr_ext()`
          - `Dist_test()`
          - `Dist_test_multi()`
          - `Hypo_test()`
          - `Hypo_test_multi()`
          - `MComp_interpreter()`
          - `Multi_conc()`
          - `NaN_stat_outliers()`
          - `agg_add_ci()`
          - `coefficient_of_variation()`
          - `coefficient_of_variation_woso()`
          - `confidence_interval()`
          - `cv()`
          - `cvwoso()`
          - `group_ANOVA_MComp()`
          - `group_ANOVA_MComp_multi()`
          - `group_Anova()`
          - `meanwoso()`
          - `pd_agg()`
          - `pd_agg_custom()`
          - `reg_stats_multi()`
          - `relative_deviation()`
          - `stat_box_vals()`
          - `stat_outliers()`
          - `stdwoso()`
        - Module contents
    - Submodules
    - exmecheva.Eva\_ACT module
    - exmecheva.Eva\_ATT module
    - exmecheva.Eva\_TBT module
    - exmecheva.eva module
    - Module contents

ExMechEva

- ExMechEva
- exmecheva package
- exmecheva.common package
- View page source

---

# exmecheva.common package

## Submodules

## exmecheva.common.analyze module

Analyzing functionality.

@author: MarcGebhardt

exmecheva.common.analyze.Geo\_curve\_TBC(*func*, *params*, *length*, *outopt='signed\_curvature'*)[source]
:   Determines the radius or curvature of a circle through three points.
    The sign indicates the direction (positve=left turn/curvature downwards).

    Parameters
    :   - **func** (*TYPE*) – DESCRIPTION.
        - **params** (*TYPE*) – DESCRIPTION.
        - **length** (*TYPE*) – DESCRIPTION.
        - **outopt** (*string**,* *optional*) –

          Option for output.
          Possible are:

          > - [‘signed\_radius’,’sr’]: signed radius
          > - [‘signed\_curvature’,’sc’]: signed curvature
          > - …: all values (Center of circle, radius, sign, quadrant to mid)

          The default is ‘signed\_curvature’.

    Returns
    :   **r** – Radius, curvature or all outputs.

    Return type
    :   float or tuple

exmecheva.common.analyze.Inter\_Lines(*r1*, *c1*, *r2*, *c2*, *out='x'*)[source]
:   Determine intersection point of two lines.

    Parameters
    :   - **r1** (*float*) – Rise of first line.
        - **c1** (*float*) – Constant part of first line.
        - **r2** (*float*) – Rise of second line.
        - **c2** (*float*) – Constant part of second line.
        - **out** (*string**,* *optional*) – Option for output (‘x’, ‘y’, or ‘xy’). The default is ‘x’.

    Returns
    :   Output values.

    Return type
    :   float or tuple of float

exmecheva.common.analyze.Line\_from2P(*P1*, *P2*)[source]
:   Determine line from two points (1st argument is x)

exmecheva.common.analyze.TP\_circle(*p1*, *p2*, *p3*)[source]
:   Returns the center and radius of the circle passing the given 3 points.
    In case the 3 points form a line, returns (None, infinity).

exmecheva.common.analyze.TP\_radius(*pts*, *outopt='signed\_curvature'*)[source]
:   Determines the radius or curvature of a circle through three points.
    The sign indicates the direction (positve=left turn/curvature downwards).

    Parameters
    :   - **pts** (*np.array* *or* *pd.DataFrame* *of* *shape* *(**2=**[**x**,**y**]**,**3=**[**left**,**mid**,**right**]**)*) – Array of point coordinates.
        - **outopt** (*string**,* *optional*) –

          Option for output.
          Possible are:

          > - [‘signed\_radius’,’sr’]: signed radius
          > - [‘signed\_curvature’,’sc’]: signed curvature
          > - …: all values (Center of circle, radius, sign, quadrant to mid)

          The default is ‘signed\_curvature’.

    Returns
    :   Radius, curvature or all outputs.

    Return type
    :   float or tuple

exmecheva.common.analyze.normalize(*pdo*, *axis=0*, *norm='absmax'*, *normadd=0.5*, *pdo\_n=None*, *warn\_excl=['x\_sc', 'dx\_sc']*)[source]
:   Normalize an array in respect to given option.

    Parameters
    :   - **pdo** (*pandas.Series* *or* *pandas.DataFrame*) – Input values.
        - **axis** (*{0* *or* *‘index’**,* *1* *or* *‘columns’}**,**,* *optional*) – Axis to apply normalizing. The default is 0.
        - **norm** (*string**,* *optional*) –

          Apllied option for normalizing.
          Possible are:

          > - ’absmax’: Normalize to maximum of absolut input values.
          > - ’absmin’: Normalize to minimum of absolut input values.
          > - ’absqua’: Normalize to Quantile of absolut input values (specified with normadd).
          > - ’max’: Normalize to maximum of input values.
          > - ’min’: Normalize to minimum of input values.
          > - ’qua’: Normalize to Quantile of input values (specified with normadd).
          > - ’val’: Normalize to given value (specified with normadd).
          > - None: No normalization (return input values).

          The default is ‘absmax’.
        - **normadd** (*int* *or* *float**,* *optional*) – Additional parameter for option ‘qua’(to set quantile value),
          or ‘val’(to set norm parameter directly).
          The default is 0.5.
        - **pdo\_n** (*pandas.Series* *or* *pandas.DataFrame* *or* *None**,* *optional*) – Additional values to get normalizing parameter.
          If None use input values. If type is Dataframe, same shape required.
          The default is None.
        - **warn\_excl** (*list*) – Defines exclusions of ZeroDivisionError-prevention-warnings.
          The default is [‘x\_sc’,’dx\_sc’].

    Raises
    :   **NotImplementedError** – Option not implemented.

    Returns
    :   **o** – Normalized output values.

    Return type
    :   pandas.Series or pandas.DataFrame

exmecheva.common.analyze.normalize\_th(*pdo*, *axis=0*, *pdo\_n=None*, *norm='absmax'*, *normadd=0.5*, *th=None*, *th\_option='abs'*, *th\_set\_val='th'*, *warn\_excl=['x\_sc', 'dx\_sc']*)[source]

exmecheva.common.analyze.sign\_n\_change(*y*)[source]
:   Computes sign and sign change of an array.

    Parameters
    :   **y** (*pandas.Series* *of* *float*) – Array which should analyzed.

    Returns
    :   - **y\_sign** (*pandas.Series of integer*) – Signs of y.
        - **y\_signchange** (*pandas.Series of boolean*) – Sign changes of y.

exmecheva.common.analyze.sign\_n\_changeth(*y*, *axis=0*, *norm=None*, *normadd=0.5*, *th=None*, *th\_option='abs'*, *th\_set\_val=0*, *rename=True*, *opt\_out='Tuple'*)[source]
:   Computes sign and sign change of an array.

    Parameters
    :   **y** (*pandas.Series* *of* *float*) – Array which should analyzed.

    Returns
    :   - **y\_sign** (*pandas.Series of integer*) – Signs of y.
        - **y\_signchange** (*pandas.Series of boolean*) – Sign changes of y.

exmecheva.common.analyze.threshhold\_setter(*pdo*, *th=None*, *option='abs'*, *set\_val='th'*)[source]

## exmecheva.common.eva\_opt\_hand module

Contains functionality for evaluation options.

@author: MarcGebhardt

exmecheva.common.eva\_opt\_hand.com\_option\_file\_read(*file*, *check\_OPT=True*)[source]
:   Reads common options file with json and return as pandas dataframe.

    Parameters
    :   - **file** (*path object* *or* *string*) – Common options file location.
        - **check\_OPT** (*bool**,* *optional*) – Check if variable name starts with ‘OPT\_’.
          If it is true, then use for options dataframe.
          The default is True.

    Returns
    :   **co** – Common options dataframe.

    Return type
    :   pd.DataFrame

exmecheva.common.eva\_opt\_hand.com\_option\_file\_write(*file*, *co*, *indent=1*)[source]
:   Writes coomon options dataframe to file with json.

    Parameters
    :   - **file** (*path object* *or* *string*) – Common options file location.
        - **co** (*pd.DataFrame*) – Common options dataframe.
        - **indent** (*int**,* *optional*) – Indent for json builder. The default is 1.

    Return type
    :   None.

exmecheva.common.eva\_opt\_hand.option\_presetter(*opt*, *mtype*, *preset*, *stype=None*)[source]
:   Presets option by value and types.

    Parameters
    :   - **opt** (*object*) – Option value.
        - **mtype** (*string*) – Main type (see set\_type\_by\_string for available types and additional “Array” with stype).
        - **preset** (*object*) – Preset value.
        - **stype** (*string* *or* *None**,* *optional*) – Sub types, used if mtype == “Array” (see set\_type\_by\_string for available types).
          The default is None.

    Returns
    :   **opt** – Presetted option value.

    Return type
    :   object

exmecheva.common.eva\_opt\_hand.option\_reader(*options*, *com\_opt\_df*, *com\_opts=None*)[source]

exmecheva.common.eva\_opt\_hand.option\_reader\_sel(*prot\_ser*, *paths*, *search\_inds=['Number', 'Designation', 'name']*, *variant=''*, *option='JFile+Prot'*, *sheet\_name='Eva\_Options'*, *re\_rkws={'header': 3, 'index\_col': 0, 'skiprows': range(4, 5)}*)[source]
:   Reads in evaluation options with different methods.

    Parameters
    :   - **prot\_ser** (*pd.Series*) – Protocoll series for measurement.
        - **paths** (*pd.Series*) –

          Series with paths (need to have indexes ‘opts’ for common options and
          :   ’prot’ if option contains ‘Sheet’).
        - **search\_inds** (*list**,* *optional*) – List of strings to build search strings in index of option sheet
          in protocoll excel table (excecuted in given order).
          Search strings have to be in prot\_ser as variable name or an
          attribute of prot\_ser (only ‘name’ implemented’).
          The default is [].
        - **variant** (*string* *or* *str**(**)* *callable**,* *optional*) – Additional addendum on search strings. Search will be exceuted after
          other search strings (see search\_inds).
          The default is [‘Number’,’Designation’,’name’].
        - **option** (*string**,* *optional*) –

          Implemented options for option\_reader\_sel.
          Implemented are:

          > - ’JFile+Prot’: Json file (loaded from paths[‘opts’] location) and
          >   :   direct load of evaluation options from protocoll
          >       series of measurement
          > - ’JFile+Sheet’: Json file (loaded from paths[‘opts’] location) and
          >   :   load of evaluation options from protocoll
          >       option sheet (see sheet\_name). Search order by
          >       search\_inds and combination of search\_inds and variant.

          The default is ‘JFile+Prot’.
        - **sheet\_name** (*string**,* *optional*) – Name of evalutaion options sheet in protocoll excel table.
          The default is “Eva\_Options”.
        - **re\_rkws** (*dict**,* *optional*) – Keyword arguments for loading evalutaion options from sheet in
          protocoll excel table (see paths and sheet\_name).
          The default is dict(header=3, skiprows=range(4,5), index\_col=0).

    Raises
    :   - **ValueError** – The search string is neither in the index nor an attribute.
        - **NotImplementedError** – Option for selecting options not implemented.

    Returns
    :   **opts\_out** – Evaluation options.

    Return type
    :   pd.Series

exmecheva.common.eva\_opt\_hand.set\_type\_by\_string(*o*, *t*)[source]
:   Create new object by given determiner.

    Parameters
    :   - **o** (*object*) – Object to convert.
        - **t** (*string*) –

          Determiner for conversion. Implemented are:
          :   - ”String”: String object
              - ”Int”: Integer object
              - ”Bool”: Boolean object
              - ”Float”: Float precision object
              - ”Json”: Json object (uses json.loads)
              - ”Free”: No conversion
              - Combination of formantioned, split by ‘\_’ in descending order

    Raises
    :   **NotImplementedError** – Given determiner/type is not implemented.

    Returns
    :   **o** – Converted object.

    Return type
    :   object

## exmecheva.common.fitting module

Created on Mon Dec 4 17:43:14 2023

@author: mgebhard

exmecheva.common.fitting.Refit\_YM\_vals(*m\_df*, *YM*, *VIP*, *n\_strain='Strain'*, *n\_stress='Stress'*, *n\_loBo=['F3']*, *n\_upBo=['F4']*, *option='range'*, *outopt='Series'*)[source]
:   Refits line values for given rising (i.e. absolute value and R² vor given elastic modulus)

    Parameters
    :   - **m\_df** (*pd.DataFrame*) – Measured data.
        - **YM** (*float*) – Youngs Modulus (fixed rising of line).
        - **VIP** (*pd.Series*) – Important points corresponding to measured data.
        - **n\_strain** (*string*) – Name of used strain (have to be in measured data).
        - **n\_stress** (*string*) – Name of used stress (have to be in measured data).
        - **n\_loBo** (*[**string**]*) – List of lower borders for determination (have to be in VIP). The default is [‘F3’].
        - **n\_upBo** (*[**string**]*) – List of upper borders for determination (have to be in VIP). The default is [‘F4’].
        - **option** (*string**,* *optional*) – Determination range. The default is ‘range’.
        - **outopt** (*string**,* *optional*) – Switch for outupt (Series or list). The default is ‘Series’.

    Returns
    :   **out** – Line values and firt parameters for fixed rising.

    Return type
    :   list or pd.Series

exmecheva.common.fitting.Rquad(*y\_true*, *y\_predicted*, *nan\_policy='omit'*)[source]
:   Returns coefficient of determination.

    Parameters
    :   - **y\_true** (*array* *of* *float*) – True values.
        - **y\_predicted** (*array* *of* *float*) – Predicted values.
        - **nan\_policy** (*bool**,* *optional*) – NaN policy (omit, raise or propagate). The default is ‘omit’.

    Raises
    :   - **ValueError** – NaN values during Rquad detected (nan\_policy is raise).
        - **NotImplementedError** – NaN policy type not implemented.

    Returns
    :   **r2\_score** – Coefficient of determination.

    Return type
    :   TYPE

exmecheva.common.fitting.YM\_eva\_com\_sel(*stress\_ser*, *strain\_ser*, *comp=True*, *name='A'*, *det\_opt='incremental'*, *\*\*kws*)[source]
:   Calculates Young’s Modulus over defined range with definable method.

    Parameters
    :   - **stress\_ser** (*pd.Series*) – Series with stress values corresponding strain\_ser.
        - **strain\_ser** (*pd.Series*) – Series with strain values corresponding stress\_ser.
        - **comp** (*boolean**,* *optional*) – Compression mode. The default is True.
        - **name** (*string**,* *optional*) – Name of operation. The default is ‘A’.
        - **det\_opt** (*TYPE**,* *optional*) – Definable method for determination.
          Ether incremental or leastsq. The default is ‘incremental’.
        - **\*\*kws** (*dict*) – Keyword dict for least-square determination.

    Returns
    :   - *det\_opt == “incremental”* –

          YM\_serpd.Series
          :   Series of Young’s Moduli.
        - *or*
        - *det\_opt == “leastsq”* –

          YMfloat
          :   Youngs Modulus (corresponds to slope of linear fit).

          YM\_absfloat
          :   Stress value on strain origin (corresponds to interception of linear fit).

          YM\_Rquadfloat
          :   Coefficient of determination.

          YM\_fitlmfit.model.ModelResult
          :   Fitting result from lmfit (use with fit.fit\_report() for report).

exmecheva.common.fitting.YM\_sigeps\_lin(*stress\_ser*, *strain\_ser*, *method='leastsq'*, *nan\_policy='omit'*, *ind\_S=None*, *ind\_E=None*)[source]
:   Calculates Youngs Modulus of a stress-strain-curve with linear approach
    using non linear least squares curve fitting.

    Parameters
    :   - **strain\_col** (*pd.Series*) – Strain curve.
        - **stress\_col** (*pd.Series*) – Stress\_curve.
        - **ind\_S** (*integer*) – First used index in df.
        - **ind\_E** (*integer*) – Last used index in df.

    Returns
    :   - **E** (*float*) – Youngs Modulus (corresponds to slope of linear fit).
        - **Eabs** (*float*) – Stress value on strain origin (corresponds to interception of linear fit).
        - **Rquad** (*float*) – Coefficient of determination.
        - **fit** (*lmfit.model.ModelResult*) – Fitting result from lmfit (use with fit.fit\_report() for report).

exmecheva.common.fitting.fit\_report\_adder(*fit*, *Var*, *Varname='R-square'*, *show\_correl=False*)[source]
:   Adds a statistical entry to the fit report of lmfit (p.e. coefficient of
    determination).

    Parameters
    :   - **fit** (*string* *or* *lmfit.model.ModelResult*) – Fit report.
        - **Var** (*float*) – Value of addional statistics entry.
        - **Varname** (*string**,* *optional*) – Name of addional statistics entry. The default is ‘R-square’.
        - **show\_correl** (*bool**,* *optional*) – Show or hide correlation. The default is False.

    Raises
    :   **TypeError** – Fit has wrong type.

    Returns
    :   **txt** – Fit report with additional statistics value.

    Return type
    :   string

exmecheva.common.fitting.func\_exp(*x*, *a*, *b*, *c*)[source]
:   Return values from a general exponential function.

exmecheva.common.fitting.func\_exp\_str(*xl*, *yl*, *a*, *b*, *c*, *t\_form='{a:.3e},{b:.3e},{c:.3e}'*)[source]
:   Return string from a general exponential function.

exmecheva.common.fitting.func\_lin(*x*, *a*, *b*)[source]
:   Return values from a general linear function.

exmecheva.common.fitting.func\_lin\_str(*xl*, *yl*, *a*, *b*, *t\_form='{a:.3e},{b:.3e}'*)[source]
:   Return string from a general linear function.

exmecheva.common.fitting.func\_pow(*x*, *a*, *b*, *c*)[source]
:   Return values from a general power function.

exmecheva.common.fitting.func\_pow\_str(*xl*, *yl*, *a*, *b*, *c*, *t\_form='{a:.3e},{b:.3e},{c:.3e}'*)[source]
:   Return string from a general power function.

exmecheva.common.fitting.regfitret(*pdo*, *x*, *y*, *name='linear'*, *guess={'a': 0.01, 'b': 0.1}*, *xl='$X$'*, *yl='$Y$'*, *t\_form='{a:.3e},{b:.3e}'*, *xt=None*, *yt=None*, *max\_nfev=1000*, *nan\_policy='omit'*, *outtype='Series'*)[source]
:   Performs a least square regression fit according given function type.

    Parameters
    :   - **pdo** (*pd.DataFrame*) – Input data.
        - **x** (*str*) – Column name for abscissa data.
        - **y** (*str*) – Column name for ordinate data.
        - **name** (*str**,* *optional*) –

          Type and name of function to adjust.
          Implemented are:

          > - ’linear’: linear and constant function (see func\_lin)
          > - ’power’: power and constant function (see func\_pow)
          > - ’exponential’: exponantial and constant function (see func\_exp)
          > - additional endings:
          >   :   - ’\_nc’: no constant value
          >       - ’\_x0’: fixed to zero

          The default is ‘linear’.
        - **guess** (*dictionary**,* *optional*) – First guess for fitting. The default is dict(a=0.01, b=0.1).
        - **xl** (*str**,* *optional*) – Variable name for abscissa. The default is r’$X$’.
        - **yl** (*str**,* *optional*) – Variable name for ordinate. The default is r’$Y$’.
        - **t\_form** (*str**,* *optional*) – String of dictionary with variables and format strings.
          The default is ‘{a:.3e},{b:.3e}’.
        - **xt** (*str**,* *optional*) – Variable string for description. The default is None.
        - **yt** (*str**,* *optional*) – Variable string for description. The default is None.
        - **max\_nfev** (*int**,* *optional*) – Maximum number of evaluations. The default is 1000.
        - **nan\_policy** (*string**,* *optional*) – NaN policy (omit, raise or propagate). The default is ‘omit’.
        - **outtype** (*string**,* *optional*) – Type for output (dictionary or pd.Series). The default is ‘Series’.

    Raises
    :   **NotImplementedError** – Function type not implemented.

    Returns
    :   **out** – Output data.

    Return type
    :   pd:series or dict

exmecheva.common.fitting.regfitret\_restring\_func(*reg\_res*, *xl='$X$'*, *yl='$Y$'*, *t\_form='{a:.3e},{b:.3e}'*, *rquad\_add=True*, *rq\_form='{:.3f}'*)[source]
:   Rebuild equation string for regfit result.

    Parameters
    :   - **reg\_res** (*pd.Series*) – Result of regfitret.
        - **xl** (*str**,* *optional*) – Variable name for abscissa. The default is r’$X$’.
        - **yl** (*str**,* *optional*) – Variable name for ordinate. The default is r’$Y$’.
        - **t\_form** (*str**,* *optional*) – String of dictionary with variables and format strings.
          The default is ‘{a:.3e},{b:.3e}’.
        - **rquad\_add** (*bool**,* *optional*) – Switch for adding coefficent of determination. The default is True.
        - **rq\_form** (*string**,* *optional*) – Format string for coefficent of determination. The default is ‘{:.3f}’.

    Raises
    :   **NotImplementedError** – Function type not implemented.

    Returns
    :   **out** – Equation string.

    Return type
    :   string

exmecheva.common.fitting.strain\_linfit(*stress\_ser*, *YM*, *YM\_abs*, *strain\_offset=0.002*)[source]
:   Linearised strain fit corresponding Youngs Modulus and stress

exmecheva.common.fitting.stress\_linfit(*strain\_ser*, *YM*, *YM\_abs*, *strain\_offset=0.002*)[source]
:   Linearised stress fit corresponding Youngs Modulus and strain

exmecheva.common.fitting.stress\_linfit\_plt(*strain\_ser*, *inds*, *YM*, *YM\_abs*, *strain\_offset=0*, *ext=0.1*)[source]
:   Linearised stress fit for plotting corresponding Youngs Modulus and strain

## exmecheva.common.helper module

Contains test and basic numeric functionality.

@author: MarcGebhardt

exmecheva.common.helper.check\_empty(*x*, *empty\_str=['', ' ', '  ', '   ', '#NVNaN', 'nan', 'NA']*)[source]
:   Tests if given variable (string or float) can interpreted as empty.

    Parameters
    :   - **x** (*None* *or* *string* *or* *float*) – Given variable to test.
        - **empty\_str** (*list* *of* *strings*) – Strings determining if variable can interpreted as empty.

    Returns
    :   **t** – Test result.

    Return type
    :   bool

exmecheva.common.helper.round\_to\_sigdig(*x*, *sd=3*)[source]
:   Round a floating number to a number of significant digits.

    Parameters
    :   - **x** (*float*) – Number.
        - **sd** (*int**,* *optional*) – Significant digits. The default is 3.

    Returns
    :   **xr** – Rounded number to significant digits.

    Return type
    :   float

exmecheva.common.helper.sigdig(*x*, *sd=3*)[source]
:   Returns the significant digits of a floating number.

    Parameters
    :   - **x** (*float*) – Number.
        - **sd** (*int**,* *optional*) – Significant digits. The default is 3.

    Returns
    :   **xr** – Rounded number to significant digits.

    Return type
    :   float

exmecheva.common.helper.str\_to\_bool(*x*, *str\_true=['True', 'true', 'Yes', '1', 'On']*)[source]
:   Interpretes a given string as boolean value

    Parameters
    :   - **x** (*string* *or* *bool*) – Value to be interpreted.
        - **str\_true** (*list* *of* *strings*) – List of strings interpreted as True.

    Returns
    :   **t** – Boolean value result.

    Return type
    :   bool

exmecheva.common.helper.type\_str\_return(*obj*)[source]
:   Tests type of given object and return an identifying string.

    Parameters
    :   **obj** (*object*) –

        Input object to check type.
        Implemented are:

        > - Standard: None, bool, str, int and float
        > - Numpy array of shape:
        >   :   - unknown shape -> npAu
        >       - single row -> npAr
        >       - single column -> npAc
        >       - frame -> npAF
        > - Pandas:
        >   :   - Index -> pdIN
        >       - Series -> pdSe
        >       - DataFrame-> pdDF

    Raises
    :   **NotImplementedError** – Type of object not implemented.

    Returns
    :   **t** – String of derived type.

    Return type
    :   str

## exmecheva.common.list\_ops module

List manipulation and interpretation functionality.

@author: MarcGebhardt

exmecheva.common.list\_ops.Failure\_code\_bool\_df(*list\_ser*, *sep=','*, *level=1*, *strength=[1, 2, 3]*, *drop\_duplicates=True*, *sort\_values=True*, *exclude=['nan']*, *replace\_whitespaces=True*, *as\_int=True*)[source]

exmecheva.common.list\_ops.Failure\_code\_checker(*fc\_ser*, *exclude=['nan']*, *pattern='[A-Z][0-9][0-9][.][0-3]'*)[source]

exmecheva.common.list\_ops.Failure\_code\_format(*fcstr*, *pattern='[A-Z][0-9][0-9][.][0-3]'*)[source]

exmecheva.common.list\_ops.Failure\_code\_lister(*\_list*, *level=1*, *strength=[1, 2, 3]*, *drop\_duplicates=True*, *replace\_whitespaces=True*)[source]
:   Shortens List of Failure-codes (Version 3.1) to levels and optional strength
    Level: 0=Procedure, 1=Procedure Failure\_Type, 2=Porcedure.Failure\_Type.Strength

    > ‘P’=Procedure, ‘F’=Failure\_Type

exmecheva.common.list\_ops.ICD\_bool\_df(*series*, *sep=','*, *level=1*, *drop\_duplicates=True*, *sort\_values=True*, *exclude=[]*, *replace\_whitespaces=True*, *as\_int=True*)[source]

exmecheva.common.list\_ops.ICD\_lister(*\_list*, *level=1*, *drop\_duplicates=True*, *replace\_whitespaces=True*)[source]
:   Shortens List of ICD-codes to levels

exmecheva.common.list\_ops.list\_boolean\_df(*series*, *unique\_items*, *as\_int=False*)[source]

exmecheva.common.list\_ops.list\_cell\_compiler(*ser*, *sep=','*, *replace\_whitespaces=True*, *replace\_nans=True*)[source]

exmecheva.common.list\_ops.list\_interpreter(*ser*, *inter\_list*, *option='exclude'*)[source]

exmecheva.common.list\_ops.list\_ser\_to\_1D(*series*, *drop\_duplicates=True*, *sort\_values=True*, *exclude=[]*)[source]

## exmecheva.common.loadnsave module

Contains functionality for loading and saving files.

@author: MarcGebhardt

exmecheva.common.loadnsave.comb\_logs(*in\_paths*, *out\_path*)[source]
:   Generates a combined log-file of a series of log-files.

    Parameters
    :   - **in\_paths** (*pd.DataFrame with columns* *[**'prot'**,**'out'**]*) – Path for finding protocol and evaluation data.
        - **out\_path** (*string*) – Output path of combined log-file (without extension).

    Return type
    :   None.

exmecheva.common.loadnsave.file\_namer(*fstr*, *svars='>'*, *svare='<'*, *sform='#'*)[source]
:   Names a file according given string (sub module of File\_namer\_interpreter).

    Parameters
    :   - **fstr** (*string*) – String with replaceable tokens.
        - **svars** (*string**,* *optional*) – Start character for token. The default is ‘>’.
        - **svare** (*string**,* *optional*) – End character for token. The default is ‘<’.
        - **sform** (*string**,* *optional*) – Form determinator at end of token. The default is ‘#’.

    Returns
    :   **vardf** – DESCRIPTION.

    Return type
    :   TYPE

    Examples

    - >Number#02<>Variant<.xlsx:
      :   with Number = 05 (from protocol) and Variant = C (from call)
          -> 05C.xlsx
    - >Designation<\_>Variant<\_foo.csv:
      :   with (from protocol) Designation = Test\_1 and Variant = B
          -> Test\_1\_B\_foo.csv

exmecheva.common.loadnsave.file\_namer\_interpreter(*fstr*, *prot\_ser*, *path*, *variant=''*, *expext='.xlsx'*, *svars='>'*, *svare='<'*, *sform='#'*)[source]
:   Builds a file location according given string (with repleacable tokens).

    Parameters
    :   - **fstr** (*string*) – String with replaceable tokens.
        - **prot\_ser** (*pd.Series*) – Series form protocoll dataframe with tokens as index.
        - **path** (*string*) – Path addendum (will be added before adjusted filename).
        - **variant** (*string**,* *optional*) – Variant (special string for token ‘Variant’). The default is ‘’.
        - **expext** (*string**,* *optional*) – Expected extension of file. The default is ‘.xlsx’.
        - **svars** (*string**,* *optional*) – Start character for token. The default is ‘>’.
        - **svare** (*string**,* *optional*) – End character for token. The default is ‘<’.
        - **sform** (*string**,* *optional*) – Form determinator at end of token. The default is ‘#’.

    Raises
    :   **ValueError** – Token not found in protocoll.

    Returns
    :   **fname** – Adjusted file name.

    Return type
    :   string

    Examples

    - >Number#02<>Variant<.xlsx:
      :   with Number = 05 (from protocol) and Variant = C (from call)
          -> 05C.xlsx
    - >Designation<\_>Variant<\_foo.csv:
      :   with (from protocol) Designation = Test\_1 and Variant = B
          -> Test\_1\_B\_foo.csv

exmecheva.common.loadnsave.pack\_hdf(*in\_paths*, *out\_path*, *hdf\_naming='Designation'*, *var\_suffix=['']*, *h5\_conc='Material\_Parameters'*, *h5\_data='Measurement'*, *prot\_rkws={'header': 11, 'index\_col': 0, 'skiprows': range(12, 13)}*, *opt\_pd\_out=True*, *opt\_hdf\_save=True*)[source]
:   Packs specimenwise evaluated measurements (HDF-files, \*.h5) to a
    HDF-database containing material parameters (key is ‘Summary’) and
    evaluated measurements (key is ‘Test\_Data’).

    Parameters
    :   - **in\_paths** (*pd.DataFrame with columns* *[**'prot'**,**'hdf'**]*) – Path for finding protocol and evaluation data.
        - **out\_path** (*string*) – Output path of created database.
        - **hdf\_naming** (*string**,* *optional*) – Naming rule of evaluated measurements (\*.h5).
          The default is ‘Designation’.
        - **var\_suffix** (*list* *of* *strings**,* *optional*) – Suffix of hdf naming rule. The default is [“”].
        - **h5\_conc** (*str**,* *optional*) – Identifier of material parameters conclusion in evaluated measurements
          (\*.h5). The default is ‘Material\_Parameters’.
        - **h5\_data** (*str**,* *optional*) – Identifier of measured and evaluated curves in evaluated measurements
          (\*.h5). The default is ‘Measurement’.
        - **prot\_rkws** (*dict**,* *optional*) – Dictionary for reading protocol. Must be keyword in pandas.read\_excel.
          The default is dict(header=11, skiprows=range(12,13), index\_col=0).
        - **opt\_pd\_out** (*bool**,* *optional*) – Option for pandas object output. The default is True.
        - **opt\_hdf\_save** (*bool**,* *optional*) – Option for hdf saving. The default is True.

    Returns
    :   - **dfc** (*pd.DataFrame*) – Summary of protocol and material data.
        - **dfd** (*pd.DataFrame*) – Summary of test data.

exmecheva.common.loadnsave.pack\_hdf\_mul(*in\_paths*, *out\_path*, *hdf\_naming='Designation'*, *var\_suffix=['']*, *h5\_conc='Material\_Parameters'*, *h5\_data='all'*, *prot\_rkws={'header': 11, 'index\_col': 0, 'skiprows': range(12, 13)}*, *opt\_pd\_out=True*, *opt\_hdf\_save=True*)[source]
:   Packs specimenwise evaluated measurements (HDF-files, \*.h5) to a
    HDF-database containing material parameters key is ‘Summary’ and other
    included data (key starting with ‘Add\_’).
    Only all keys in single hdf-files are implemented. (High storage
    requirements!)

    Parameters
    :   - **in\_paths** (*pd.DataFrame with columns* *[**'prot'**,**'hdf'**]*) – Path for finding protocol and evaluation data.
        - **out\_path** (*string*) – Output path of created database.
        - **hdf\_naming** (*string**,* *optional*) – Naming rule of evaluated measurements (\*.h5).
          The default is ‘Designation’.
        - **var\_suffix** (*list* *of* *strings**,* *optional*) – Suffix of hdf naming rule. The default is [“”].
        - **h5\_conc** (*str**,* *optional*) – Identifier of material parameters conclusion in evaluated measurements
          (\*.h5). The default is ‘Material\_Parameters’.
        - **h5\_data** (*str**,* *optional*) – Identifier for additional packaging. Only ‘all’ (packing all keys from
          input hfd’s)implemented. The default is ‘all’.
        - **prot\_rkws** (*dict**,* *optional*) – Dictionary for reading protocol. Must be keyword in pandas.read\_excel.
          The default is dict(header=11, skiprows=range(12,13), index\_col=0).
        - **opt\_pd\_out** (*bool**,* *optional*) – Option for pandas object output. The default is True.
        - **opt\_hdf\_save** (*bool**,* *optional*) – Option for hdf saving. The default is True.

    Returns
    :   - **dfc** (*pd.DataFrame*) – Summary of protocol and material data.
        - **dfd** (*pd.DataFrame*) – Summary of test data.

## exmecheva.common.mc\_char module

Created on Tue Dec 5 12:25:34 2023

@author: mgebhard

exmecheva.common.mc\_char.Diff\_Quot(*meas\_curve\_A*, *meas\_curve\_B*, *smoothbool*, *smooth\_lvl*, *opt\_shift=False*)[source]
:   Computes difference quotient (1st[rise] and 2nd-grade[curvature])
    to find points of inconstancy in measured curves.

    Parameters
    :   - **meas\_curve\_A** (*pd.Series* *of* *float*) – Abscissa of the curve for which the difference quotient is to be determined.
        - **meas\_curve\_B** (*pd.Series* *of* *float*) – Ordinate of the curve for which the difference quotient is to be determined.
        - **smoothbool** (*bool*) – Controles determination of rolling mean.
        - **smooth\_lvl** (*positv integer*) – Width of rolling mean determination.
        - **opt\_shift** (*bool*) – Shifting returned values to pointed input values.
          If False it points to the index before.
          Leading to a difficult to handle shift, ingreasing with difference quotient grade.
          Optional. The defalut is False.
        - **Test** –
        - **-------** –
        - **t=pd.DataFrame****(****[****[****0** – [5,2.5],[6,2],[7,2]], columns=[‘x’,’y’])
        - **0****]** – [5,2.5],[6,2],[7,2]], columns=[‘x’,’y’])
        - **[****1** – [5,2.5],[6,2],[7,2]], columns=[‘x’,’y’])
        - **0****]** – [5,2.5],[6,2],[7,2]], columns=[‘x’,’y’])
        - **[****1.5** – [5,2.5],[6,2],[7,2]], columns=[‘x’,’y’])
        - **1****]** – [5,2.5],[6,2],[7,2]], columns=[‘x’,’y’])
        - **[****2.5** – [5,2.5],[6,2],[7,2]], columns=[‘x’,’y’])
        - **2****]** – [5,2.5],[6,2],[7,2]], columns=[‘x’,’y’])
        - **[****3.5** – [5,2.5],[6,2],[7,2]], columns=[‘x’,’y’])
        - **3****]** – [5,2.5],[6,2],[7,2]], columns=[‘x’,’y’])
        - **[****4** – [5,2.5],[6,2],[7,2]], columns=[‘x’,’y’])
        - **3.5****]** – [5,2.5],[6,2],[7,2]], columns=[‘x’,’y’])

    :param : [5,2.5],[6,2],[7,2]], columns=[‘x’,’y’])
    :param DQ\_df = pd.concat(Diff\_Quot(t.x:

    > ” DQ1 DQ1\_signchange DQ2 DQ2\_signchange DQ3 DQ3\_signchange
    > :   0 NaN True NaN True NaN True
    >     1 0.0 True NaN True NaN True
    >     2 2.0 False 4.0 True NaN True
    >     3 1.0 False -1.0 True -5.0 True
    >     4 1.0 False 0.0 False 1.0 True
    >     5 1.0 True 0.0 True 0.0 True
    >     6 -1.0 False -2.0 True -2.0 True
    >     7 -0.5 True 0.5 False 2.5 True
    >     8 0.0 NaN 0.5 NaN 0.0 NaN”

    Parameters
    :   - **t.y** –

          ” DQ1 DQ1\_signchange DQ2 DQ2\_signchange DQ3 DQ3\_signchange
          :   0 NaN True NaN True NaN True
              1 0.0 True NaN True NaN True
              2 2.0 False 4.0 True NaN True
              3 1.0 False -1.0 True -5.0 True
              4 1.0 False 0.0 False 1.0 True
              5 1.0 True 0.0 True 0.0 True
              6 -1.0 False -2.0 True -2.0 True
              7 -0.5 True 0.5 False 2.5 True
              8 0.0 NaN 0.5 NaN 0.0 NaN”
        - **False** –

          ” DQ1 DQ1\_signchange DQ2 DQ2\_signchange DQ3 DQ3\_signchange
          :   0 NaN True NaN True NaN True
              1 0.0 True NaN True NaN True
              2 2.0 False 4.0 True NaN True
              3 1.0 False -1.0 True -5.0 True
              4 1.0 False 0.0 False 1.0 True
              5 1.0 True 0.0 True 0.0 True
              6 -1.0 False -2.0 True -2.0 True
              7 -0.5 True 0.5 False 2.5 True
              8 0.0 NaN 0.5 NaN 0.0 NaN”
        - **2** –

          ” DQ1 DQ1\_signchange DQ2 DQ2\_signchange DQ3 DQ3\_signchange
          :   0 NaN True NaN True NaN True
              1 0.0 True NaN True NaN True
              2 2.0 False 4.0 True NaN True
              3 1.0 False -1.0 True -5.0 True
              4 1.0 False 0.0 False 1.0 True
              5 1.0 True 0.0 True 0.0 True
              6 -1.0 False -2.0 True -2.0 True
              7 -0.5 True 0.5 False 2.5 True
              8 0.0 NaN 0.5 NaN 0.0 NaN”
        - **True****)** –

          ” DQ1 DQ1\_signchange DQ2 DQ2\_signchange DQ3 DQ3\_signchange
          :   0 NaN True NaN True NaN True
              1 0.0 True NaN True NaN True
              2 2.0 False 4.0 True NaN True
              3 1.0 False -1.0 True -5.0 True
              4 1.0 False 0.0 False 1.0 True
              5 1.0 True 0.0 True 0.0 True
              6 -1.0 False -2.0 True -2.0 True
              7 -0.5 True 0.5 False 2.5 True
              8 0.0 NaN 0.5 NaN 0.0 NaN”
        - **axis=1****)** –

          ” DQ1 DQ1\_signchange DQ2 DQ2\_signchange DQ3 DQ3\_signchange
          :   0 NaN True NaN True NaN True
              1 0.0 True NaN True NaN True
              2 2.0 False 4.0 True NaN True
              3 1.0 False -1.0 True -5.0 True
              4 1.0 False 0.0 False 1.0 True
              5 1.0 True 0.0 True 0.0 True
              6 -1.0 False -2.0 True -2.0 True
              7 -0.5 True 0.5 False 2.5 True
              8 0.0 NaN 0.5 NaN 0.0 NaN”

    Returns
    :   - **DQ1** (*pd.Series of float*) – Differential quotient 1st grade (rise) of meas\_curve\_B to meas\_curve\_A.
        - **DQ1\_signchange** (*pd.Series of bool*) – Indicates sign changes of DQ1.
        - **DQ2** (*pd.Series of float*) – Differential quotient 2nd grade (curvature) of meas\_curve\_B to meas\_curve\_A.
        - **DQ2\_signchange** (*pd.Series of bool*) – Indicates sign changes of DQ2.
        - **DQ3** (*pd.Series of float*) – Differential quotient 3rd grade of meas\_curve\_B to meas\_curve\_A.
        - **DQ3\_signchange** (*pd.Series of bool*) – Indicates sign changes of DQ3.

exmecheva.common.mc\_char.Diff\_Quot2(*x*, *y*, *smooth\_bool=False*, *smooth\_type='SMA'*, *smooth\_opts={'window\_length': 3}*, *smooth\_snip=False*, *sc\_kwargs={'norm': 'absmax', 'normadd': 0.5, 'th': 0.05, 'th\_option': 'abs', 'th\_set\_val': 0}*, *opt\_shift=False*, *opt\_out='Tuple'*)[source]
:   Computes difference quotient of input arrays (1st[rise], 2nd[curvature] and 3rd-grade)
    to find points of inconstancy in measured curves.

    Parameters
    :   - **x** (*pd.Series* *of* *float*) – Abscissa of the curve for which the difference quotient is to be determined.
        - **y** (*pd.Series* *of* *float*) – Ordinate of the curve for which the difference quotient is to be determined.
        - **smoothbool** (*bool* *or* *string*) – Controles determination of rolling mean.
        - **smooth\_type** (*string**,* *case-sensitive**,* *optional*) –

          Choosen smoothing type.
          Optional. The defalut is ‘SMA’.
          Possible are:

          > - ’SMA’: Moving average based on numpy.convolve.
          > - ’SMA\_f1d’: Moving average based scipy.ndimage.filters.uniform\_filter1d.
          > - ’SavGol’: Savitzky-Golay filter based on scipy.signal.savgol\_filter.
        - **smooth\_opts** (*dict**,* *optional*) – Keywords and values to pass to smoothing method.
          For further informations see smooth\_type and linked methods.
          Optional. The defalut is {‘window\_length’:3, ‘mode’:’same’}.
        - **smooth\_snip** (*bool* *or* *integer**,* *optional*) – Trimming of output. Either, if True with window\_length in smooth\_opts,
          none if False, or with inserted distance.
          The default is False.
        - **opt\_shift** (*bool*) – Shifting returned values to pointed input values.
          If False it points to the index before.
          Leading to a difficult to handle shift.
          Optional. The defalut is False.
        - **opt\_out** (*string*) – Type of Output. Either Tuple or pandas dataframe.
          Optional. The defalut is ‘Tuple’.
        - **Test** –
        - **-------** –
        - **t=pd.DataFrame****(****[****[****0** – [5,2.5],[6,2],[7,2]], columns=[‘x’,’y’])
        - **0****]** – [5,2.5],[6,2],[7,2]], columns=[‘x’,’y’])
        - **[****1** – [5,2.5],[6,2],[7,2]], columns=[‘x’,’y’])
        - **0****]** – [5,2.5],[6,2],[7,2]], columns=[‘x’,’y’])
        - **[****1.5** – [5,2.5],[6,2],[7,2]], columns=[‘x’,’y’])
        - **1****]** – [5,2.5],[6,2],[7,2]], columns=[‘x’,’y’])
        - **[****2.5** – [5,2.5],[6,2],[7,2]], columns=[‘x’,’y’])
        - **2****]** – [5,2.5],[6,2],[7,2]], columns=[‘x’,’y’])
        - **[****3.5** – [5,2.5],[6,2],[7,2]], columns=[‘x’,’y’])
        - **3****]** – [5,2.5],[6,2],[7,2]], columns=[‘x’,’y’])
        - **[****4** – [5,2.5],[6,2],[7,2]], columns=[‘x’,’y’])
        - **3.5****]** – [5,2.5],[6,2],[7,2]], columns=[‘x’,’y’])

    :param : [5,2.5],[6,2],[7,2]], columns=[‘x’,’y’])
    :param DQ\_df = pd.concat(Diff\_Quot(t.x:

    > ” DQ1 DQ1\_signchange DQ2 DQ2\_signchange DQ3 DQ3\_signchange
    > :   0 NaN True NaN True NaN True
    >     1 0.0 True NaN True NaN True
    >     2 2.0 False 4.0 True NaN True
    >     3 1.0 False -1.0 True -5.0 True
    >     4 1.0 False 0.0 False 1.0 True
    >     5 1.0 True 0.0 True 0.0 True
    >     6 -1.0 False -2.0 True -2.0 True
    >     7 -0.5 True 0.5 False 2.5 True
    >     8 0.0 False 0.5 False 0.0 False”

    Parameters
    :   - **t.y** –

          ” DQ1 DQ1\_signchange DQ2 DQ2\_signchange DQ3 DQ3\_signchange
          :   0 NaN True NaN True NaN True
              1 0.0 True NaN True NaN True
              2 2.0 False 4.0 True NaN True
              3 1.0 False -1.0 True -5.0 True
              4 1.0 False 0.0 False 1.0 True
              5 1.0 True 0.0 True 0.0 True
              6 -1.0 False -2.0 True -2.0 True
              7 -0.5 True 0.5 False 2.5 True
              8 0.0 False 0.5 False 0.0 False”
        - **False** –

          ” DQ1 DQ1\_signchange DQ2 DQ2\_signchange DQ3 DQ3\_signchange
          :   0 NaN True NaN True NaN True
              1 0.0 True NaN True NaN True
              2 2.0 False 4.0 True NaN True
              3 1.0 False -1.0 True -5.0 True
              4 1.0 False 0.0 False 1.0 True
              5 1.0 True 0.0 True 0.0 True
              6 -1.0 False -2.0 True -2.0 True
              7 -0.5 True 0.5 False 2.5 True
              8 0.0 False 0.5 False 0.0 False”
        - **2** –

          ” DQ1 DQ1\_signchange DQ2 DQ2\_signchange DQ3 DQ3\_signchange
          :   0 NaN True NaN True NaN True
              1 0.0 True NaN True NaN True
              2 2.0 False 4.0 True NaN True
              3 1.0 False -1.0 True -5.0 True
              4 1.0 False 0.0 False 1.0 True
              5 1.0 True 0.0 True 0.0 True
              6 -1.0 False -2.0 True -2.0 True
              7 -0.5 True 0.5 False 2.5 True
              8 0.0 False 0.5 False 0.0 False”
        - **True****)** –

          ” DQ1 DQ1\_signchange DQ2 DQ2\_signchange DQ3 DQ3\_signchange
          :   0 NaN True NaN True NaN True
              1 0.0 True NaN True NaN True
              2 2.0 False 4.0 True NaN True
              3 1.0 False -1.0 True -5.0 True
              4 1.0 False 0.0 False 1.0 True
              5 1.0 True 0.0 True 0.0 True
              6 -1.0 False -2.0 True -2.0 True
              7 -0.5 True 0.5 False 2.5 True
              8 0.0 False 0.5 False 0.0 False”
        - **axis=1****)** –

          ” DQ1 DQ1\_signchange DQ2 DQ2\_signchange DQ3 DQ3\_signchange
          :   0 NaN True NaN True NaN True
              1 0.0 True NaN True NaN True
              2 2.0 False 4.0 True NaN True
              3 1.0 False -1.0 True -5.0 True
              4 1.0 False 0.0 False 1.0 True
              5 1.0 True 0.0 True 0.0 True
              6 -1.0 False -2.0 True -2.0 True
              7 -0.5 True 0.5 False 2.5 True
              8 0.0 False 0.5 False 0.0 False”

    Returns
    :   - **DQ1** (*pd.Series of float*) – Differential quotient 1st grade (rise) of y to x.
        - **DQ1\_si** (*pd.Series of bool*) – Signs of DQ1.
        - **DQ1\_sc** (*pd.Series of bool*) – Indicates sign changes of DQ1.
        - **DQ2** (*pd.Series of float*) – Differential quotient 2nd grade (curvature) of y to x.
        - **DQ2\_si** (*pd.Series of bool*) – Signs of DQ2.
        - **DQ2\_sc** (*pd.Series of bool*) – Indicates sign changes of DQ2.
        - **DQ3** (*pd.Series of float*) – Differential quotient 3rd grade (curvature change) of y to x.
        - **DQ3\_si** (*pd.Series of bool*) – Signs of DQ3.
        - **DQ3\_sc** (*pd.Series of bool*) – Indicates sign changes of DQ3.

exmecheva.common.mc\_char.Diff\_Quot3(*y*, *x=None*, *deep=3*, *ex\_bool=True*, *ex\_kwargs={'polydeg': 1}*, *smooth\_bool=False*, *smooth\_type='SMA'*, *smooth\_opts={'window\_length': 3}*, *sc\_kwargs={'norm': 'absmax', 'normadd': 0.5, 'th': 0.05, 'th\_option': 'abs', 'th\_set\_val': 0}*, *shift\_value=False*, *opt\_out='DataFrame'*)[source]
:   Computes difference quotient of input arrays (nth-grade) and their signs,
    as well as sign changes to find points of inconstancy in measured curves.

    Parameters
    :   - **y** (*pd.Series* *of* *float*) – Ordinate of the curve for which the difference quotient is to be
          determined.
        - **x** (*pd.Series* *of* *float* *or* *None**,* *optional*) – Abscissa of the curve for which the difference quotient is to be
          determined. The default is None.
        - **deep** (*int**,* *optional*) – Number of differential quotients to determine. The default is 3.
        - **ex\_bool** (*bool**,* *optional*) – Switch for extending data with Extend\_Series\_n\_setter and afterwards
          retrim them with Retrim\_Series. The default is True.
        - **ex\_kwargs** (*dict**,* *optional*) – Keyword arguments for Extend\_Series\_n\_setter.
          The default is {‘polydeg’:1}.
        - **smooth\_bool** (*bool* *or* *string**,* *optional*) – Switch for smoothing of data. Must ether be True or False or in
          [‘Input’,’x-only’,’y-only’,’yandDQ’,’DQ’]. The default is False.
        - **smooth\_type** (*string**,* *optional*) – Type of smoothing methode (see Smoothsel for further information).
          The default is ‘SMA’.
        - **smooth\_opts** (*dict**,* *optional*) – Smoothing option for choosen method (see Smoothsel for further
          information). The default is {‘window\_length’:3}.
        - **sc\_kwargs** (*dict**,* *optional*) –

          Keyword arguments for sign and sign change determination
          (normalization and thresholding, for further information see
          ./analyze.sign\_n\_changeth).
          The default is {‘norm’:’absmax’, ‘normadd’:0.5,

          > ’th’:0.05, ‘th\_option’:’abs’, ‘th\_set\_val’:0}.
        - **shift\_value** (*bool* *or* *int**,* *optional*) – Shifting of values. The default is False.
        - **opt\_out** (*string**,* *optional*) – Output option (only DataFrame or DF implemented yet).
          The default is ‘DataFrame’.

    Raises
    :   - **TypeError** – Determination length of smooth\_opts seems to be wrong.
        - **NotImplementedError** – Output option not implemented.

    Returns
    :   **out** – Differnece quotients, signs and sign changes of given input data.

    Return type
    :   pd.DataFrame

exmecheva.common.mc\_char.YM\_eva\_range\_refine(*m\_df*, *VIP*, *n\_strain*, *n\_stress*, *n\_loBo='S'*, *n\_upBo='U'*, *d\_loBo=0.05*, *d\_max=0.75*, *rise\_det=[True, 4]*, *n\_Outlo='F3'*, *n\_Outmi='FM'*, *n\_Outhi='F4'*)[source]
:   Refines the Youngs Modulus determinition range according to
    “Keuerleber, M. (2006) - Bestimmung des Elastizitätsmoduls von Kunststoffen
    bei hohen Dehnraten am Beispiel von PP. Von der Fakultät Maschinenbau der
    Universität Stuttgart zur Erlangung der Würde eines Doktor-Ingenieurs (Dr.-Ing.)
    genehmigte Abhandlung. Doktorarbeit. Universität Stuttgart, Stuttgart”

    Parameters
    :   - **m\_df** (*pd.DataFrame*) – Measured data.
        - **VIP** (*pd.Series*) – Important points corresponding to measured data.
        - **n\_strain** (*string*) – Name of used strain (have to be in measured data).
        - **n\_stress** (*string*) – Name of used stress (have to be in measured data).
        - **n\_loBo** (*string*) – Lower border for determination (have to be in VIP). The default is ‘S’.
        - **n\_upBo** (*string*) – Upper border for determination (have to be in VIP). The default is ‘U’.
        - **d\_loBo** (*float/str**,* *optional*) – When float: Percentage of range between n\_upBo and n\_loBo as start distance to n\_loBo.
          When str starting with ‘S’, followed by integer: Distance in steps to n\_loBo.
          The default is 0.05.
        - **d\_max** (*float**,* *optional*) – Percentage of . The default is 0.75.
        - **rise\_det** (*[**bool**,* *int**]**,* *optional*) – Determination options for stress rising ([smoothing, smoothing factor]).
        - **n\_Outlo** (*string**,* *optional*) – Name of new lower border. The default is ‘F3’.
        - **n\_Outmi** (*string**,* *optional*) – Name of maximum differential quotient. The default is ‘FM’.
        - **n\_Outhi** (*string**,* *optional*) – Name of new upper border. The default is ‘F4’.

    Yields
    :   - **VIP\_new** (*pd.Series*) – Important points corresponding to measured data.
        - **txt** (*string*) – Documantation string.

exmecheva.common.mc\_char.curve\_characterizer(*x*, *y*, *ex\_bool=True*, *ex\_kwargs={'polydeg': 1}*, *smooth\_bool=True*, *smooth\_type='SMA'*, *smooth\_opts={'window\_length': 3}*, *shift\_value=-1*, *sc\_kwargs={'norm': 'absmax', 'normadd': 0.5, 'th': 0.05, 'th\_option': 'abs', 'th\_set\_val': 0}*, *peak\_norm='absmax'*, *peak\_kwargs={'height': 0.1, 'prominence': 0.1}*, *cc\_snip=3*, *cc\_threshold=0.01*, *cc\_norm='abs\_max\_ref'*, *cc\_refine=True*, *ccr\_threshold=0.75*, *ccr\_norm='abs\_mid'*, *nan\_policy='omit'*)[source]
:   Characterize a measured curve. To plot results please see
    curve\_char\_plotter in ./plotting.py.

    Parameters
    :   - **x** (*pandas Series* *of* *float**, or* *pandas.DataFrame*) – Abscissa or difference quotient dataframe of input.
        - **y** (*pandas Series* *of* *float*) – Ordinate of input.
        - **smooth\_bool** (*bool* *or* *string**,* *optional*) – Smoothing behavior. The default is True.
        - **smooth\_type** (*string**,* *case-sensitive**,* *optional*) –

          Choosen smoothing type.
          Optional. The defalut is ‘SMA’.
          Possible are:

          > - ’SMA’: Moving average based on numpy.convolve.
          > - ’SMA\_f1d’: Moving average based scipy.ndimage.filters.uniform\_filter1d.
          > - ’SavGol’: Savitzky-Golay filter based on scipy.signal.savgol\_filter.
        - **smooth\_opts** (*dict**,* *optional*) – Keywords and values to pass to smoothing method.
          For further informations see smooth\_type and linked methods.
          Optional. The defalut is {‘window\_length’:3, ‘mode’:’same’}.
        - **smooth\_snip** (*bool* *or* *integer**,* *optional*) – Trimming of output. Either, if True with window\_length in smooth\_opts,
          none if False, or with inserted distance.
          The default is False.
        - **opt\_shift** (*bool*) – Shifting returned values to pointed input values.
          If False it points to the index before.
          Leading to a difficult to handle shift.
          Optional. The defalut is False.
        - **peak\_norm** (*string**,* *optional*) – Normalization method. The default is ‘absmax’.
        - **peak\_kwargs** (*dict**,* *optional*) – Keyword arguments to pass to scipy.signal.find\_peaks.
          The default is {‘prominence’:0.1, ‘height’:0.1}.
        - **cc\_snip** (*corresponding type* *of* *index**,* *optional*) – Determination distance to start and end for characterization of
          section. The default is 3.
        - **cc\_threshold** (*float**,* *optional*) – Determination threshold for characterization of section.
          The default is 0.01.
        - **cc\_norm** (*string**,* *optional*) – Nomralization method for characterization of section.
          The default is ‘abs\_max\_ref’.
        - **cc\_refine** (*bool**,* *optional*) – Turn refinement by curvature of curve section on. The default is True.
        - **ccr\_threshold** (*float**,* *optional*) – Determination threshold of refinement. The default is 0.75.
        - **ccr\_norm** (*string**,* *optional*) – Nomralization method of refinement. The default is ‘abs\_mid’.

    Returns
    :   - *pandas Dataframe* –

          Characterization of input (for linear parts: linearisation and
          :   intersection to previous included).
        - *pandas Series* – Points of first characteriation(S=Start,E=End, I=Increase, D=Decrease).
        - *pandas Dataframe* – Maxima and minima of input, as well as differential quotients.
        - *pandas Dataframe* – Input, as well as differential quotients.

exmecheva.common.mc\_char.curve\_merger(*cps1*, *cps2*, *how='1stMax'*, *option='nearest'*)[source]
:   Finds time offset between two measured curves.

    Parameters
    :   - **cps1** (*pandas Dataframe* *(**result* *of* *curve\_characterizer**)*) – First curve characterization.
        - **cps2** (*pandas Dataframe* *(**result* *of* *curve\_characterizer**)*) – Second curve characterization.
        - **how** (*string**,* *optional*) –

          Switch for how to determine time offset. Possible are:
          :   - ’1stMax’: first maximum
              - ’wo\_1st+last’: without first and last relevant positions
              - ’complete’: all relevant positions
              - ’list;[x,x,…]’: list of relevant positions
              - ’int;x’: one relevant position
              - ’fix;x’: fixed time offset

          The default is ‘1stMax’.
        - **option** (*string**,* *optional*) – Switch for offset determination. The default is ‘nearest’
          (merging nearest positions found in both frames).

    Raises
    :   **NotImplementedError** – Method not implemented.

    Returns
    :   - **t\_off** (*float*) – Offset to apply on first to merge second.
        - **t1** (*float or pd.Series*) – Timestamp/-s of first curve characterization.
        - **t2** (*float or pd.Series*) – Timestamp/-s of second curve characterization.

exmecheva.common.mc\_char.curvecar\_refine(*i\_start*, *i\_mid*, *i\_end*, *ref\_ser*, *threshold=0.5*, *norm='abs\_mid'*, *out\_vals=['', 'Pos', 'Neg']*)[source]
:   Determine type, as well as new start and end points,
    of a refined section by comparing a reference series to a given threshold.

    Parameters
    :   - **i\_start** (*corresponding type* *of* *index*) – Start index of first section.
        - **i\_mid** (*corresponding type* *of* *index*) – Index between sections.
        - **i\_end** (*corresponding type* *of* *index*) – End index of second section.
        - **ref\_ser** (*pands Series*) – Reference series.
        - **threshold** (*float**,* *optional*) – Determination threshold. The default is 0.5.
        - **norm** (*string**,* *optional*) – Nomralization method. The default is ‘abs\_mid’.
        - **out\_vals** (*array* *of* *strings**,* *optional*) – Strings for determination type. The default is [‘’,’Pos’,’Neg’].

    Returns
    :   - *string* – Determined type of curve section.
        - *corresponding type of index* – New start point of refined curve section.
        - *corresponding type of index* – New end point of refined curve section.
        - *float* – Mean value over range of reference series.

exmecheva.common.mc\_char.curvecar\_section(*i\_start*, *i\_end*, *ref\_ser*, *sm\_w\_length=0*, *threshold=0.01*, *norm='abs\_max\_ref'*, *out\_vals=['', 'Const', 'Rise', 'Fall']*, *kind='median'*)[source]
:   Determine type of section by comparing a reference series to a given threshold.

    Parameters
    :   - **i\_start** (*corresponding type* *of* *index*) – Start index of section.
        - **i\_end** (*corresponding type* *of* *index*) – End index of section.
        - **ref\_ser** (*pands Series*) – Reference series.
        - **sm\_w\_length** (*corresponding type* *of* *index**,* *optional*) – Determination distance to start and end. The default is 0.
        - **threshold** (*float**,* *optional*) – Determination threshold. The default is 0.01.
        - **norm** (*string**,* *optional*) – Nomralization method. The default is ‘abs\_max\_ref’.
        - **out\_vals** (*array* *of* *strings**,* *optional*) – Strings for determination type. The default is [‘’,’Const’,’Rise’,’Fall’].

    Returns
    :   - *string* – Determined type of curve section.
        - *float* – Mean value over range of reference series.

exmecheva.common.mc\_char.find\_SandE(*Val*, *Val2*, *drop\_op*, *drop\_val*)[source]
:   Computes first and last indices which fullfill the choosen condition.

    Parameters
    :   - **Val** (*pd.Series**(**[**]**,**dtype='float64'**)*) – Input value.
        - **Val2** (*pd.Series**(**[**]**,**dtype='float64'**)*) – Testing value.
        - **drop\_op** (*string**,* *case-sensitive*) – Condition name (abV\_self,pgm\_self,pgm\_other,qua\_self),
        - **drop\_val** (*float64*) – Value for condition.

    Returns
    :   - **iS** (*TYPE*) – First index which fullfill the choosen condition.
        - **iE** (*TYPE*) – Last index which fullfill the choosen condition.

exmecheva.common.mc\_char.peaky\_finder(*df*, *cols='all'*, *norm='absmax'*, *fp\_kwargs={'height': 0.1, 'prominence': 0.1}*, *out\_opt='valser-loc'*)[source]
:   Find peaks in passed input data.

    Parameters
    :   - **df** (*pandas DataFrame*) – Input data.
        - **cols** (*string* *or* *array* *of* *strings* *or* *boolean**,* *optional*) – Columns (axis=1) of data to use. The default is ‘all’.
        - **norm** (*string**,* *optional*) – Normalization method. The default is ‘absmax’.
        - **fp\_kwargs** (*dict**,* *optional*) – Keyword arguments to pass to scipy.signal.find\_peaks.
          The default is {‘prominence’:0.1, ‘height’:0.1}.
        - **out\_opt** (*string**,* *optional*) – Output options. The default is ‘valser-loc’.

    Raises
    :   **NotImplementedError** – Option not implemented.

    Returns
    :   **o** – Series with input-columns as index and array of input index as values.

    Return type
    :   Series of arrays

exmecheva.common.mc\_char.peaky\_finder\_MM(*df*, *cols='all'*, *norm='absmax'*, *fp\_kwargs={'height': 0.1, 'prominence': 0.1}*, *out\_opt='valser-loc'*)[source]
:   Pacckage method of peaky\_finder to find maxima and minima.

exmecheva.common.mc\_char.poi\_det\_plh(*x*, *y*, *cps*, *z=None*, *dft=None*, *det\_dist=2*, *det\_r='RF'*, *refineP=True*)[source]
:   Automatically determines high and low points, as well of points with with
    changing rise based on a given curve.

    Parameters
    :   - **x** (*pd.Series*) – Input data on abcissa, p.e. time.
        - **y** (*pd.Series*) – Input data on ordinate, p.e. force.
        - **cps** (*pandas Dataframe* *(**first result* *of* *curve\_characterizer**)*) – Measurement curve characterization.
        - **z** (*pd.Series**,* *optional*) – Additional input data on ordinate, p.e. displacement.
          The default is None.
        - **dft** (*pandas Dataframe* *(**last result* *of* *curve\_characterizer**)**,* *optional*) – Input, as well as differential quotients. Used if refineP is True.
          The default is None.
        - **det\_dist** (*int**,* *optional*) – Determination distance. The default is 2.
        - **det\_r** (*string**,* *optional*) – Determination range selection keyword to search in cps.
          The default is ‘RF’.
        - **refineP** (*bool**,* *optional*) – Switch for refinement. The default is True.

    Returns
    :   - **cip** (*pd.Series*) – Points of interest, with index by names and value by index of input.
          data. Labels are ‘H’-high point, ‘L’-low point, ‘Pl’-rise change on
          loading and ‘Pu’-rise change on unloading.
        - **c** (*int*) – Number of cyclic loadings.

    Example

    if ((‘preload’ in \_opts[‘OPT\_Testtype’])
    :   or (‘cyclic’ in \_opts[‘OPT\_Testtype’])):

    Vt, cycles = poi\_det\_plh(x=messu.Time, y=messu.Force,
    :   cps=cps\_t, z=messu.Way, dft=df\_t,
        det\_dist=\_opts[‘OPT\_Determination\_Distance’],
        det\_r=’RF’, refineP=True)

    VIP\_messu = Vt.combine\_first(VIP\_messu).sort\_values().astype(int)

exmecheva.common.mc\_char.poi\_fixeva(*pds*, *p*, *iS=None*, *iE=None*, *range\_sub='min'*, *norm='max'*, *option='abs'*, *check\_irr=False*, *irr=None*, *irr\_opt='nearest'*)[source]
:   Determine points for start and end of fixed range determination
    (by ratios to given value).

    Parameters
    :   - **pds** (*pd.Series*) – Input values, p.e. measured stress data.
        - **p** (*list* *of* *two floats*) – Lower (first) and upper (second) border of determination range.
        - **iS** (*TYPE**,* *optional*) – DESCRIPTION. The default is None.
        - **iE** (*TYPE**,* *optional*) – DESCRIPTION. The default is None.
        - **range\_sub** (*TYPE**,* *optional*) – DESCRIPTION. The default is ‘min’.
        - **norm** (*TYPE**,* *optional*) – DESCRIPTION. The default is ‘max’.
        - **option** (*TYPE**,* *optional*) – DESCRIPTION. The default is ‘abs’.
        - **check\_irr** (*TYPE**,* *optional*) – DESCRIPTION. The default is False.
        - **irr** (*TYPE**,* *optional*) – DESCRIPTION. The default is None.
        - **irr\_opt** (*TYPE**,* *optional*) – DESCRIPTION. The default is ‘nearest’.

    Raises
    :   **NotImplementedError** – DESCRIPTION.

    Returns
    :   - *TYPE* – DESCRIPTION.
        - *TYPE* – DESCRIPTION.
        - *TYPE* – DESCRIPTION.

    Example

    for cc in np.arange(1,cycles+1):
    :   tmp\_iS=poi\_vip\_namer(VIP=VIP\_messu,ttype=\_opts[‘OPT\_Testtype’],
        :   key=\_opts[‘OPT\_YM\_Determination\_range’][-2],
            cc=cc,mc=cycles,lu=’l’)

        tmp\_iE=poi\_vip\_namer(VIP=VIP\_messu,ttype=\_opts[‘OPT\_Testtype’],
        :   key=\_opts[‘OPT\_YM\_Determination\_range’][-1],
            cc=cc,mc=cycles,lu=’l’)

        tmp,\_,\_ = poi\_fixeva(pds=messu.Stress,
        :   p=\_opts[‘OPT\_YM\_Determination\_range’][0:2],
            iS=VIP\_messu[tmp\_iS], iE=VIP\_messu[tmp\_iE],
            range\_sub=’min’, norm=’max’, option=’abs’,
            check\_irr=True, irr=messu[messu.driF\_schg].index.values,
            irr\_opt=’nearest’)

        VIP\_messu[‘FlA’+str(cc)],VIP\_messu[‘FlB’+str(cc)]=tmp
        if not ((‘destructive’ in \_opts[‘OPT\_Testtype’]) and cc==cycles):

        > tmp\_iS=poi\_vip\_namer(VIP=VIP\_messu,ttype=\_opts[‘OPT\_Testtype’],
        > :   key=\_opts[‘OPT\_YM\_Determination\_range’][-1],
        >     cc=cc,mc=cycles,lu=’u’)
        >
        > tmp\_iE=poi\_vip\_namer(VIP=VIP\_messu,ttype=\_opts[‘OPT\_Testtype’],
        > :   key=\_opts[‘OPT\_YM\_Determination\_range’][-2],
        >     cc=cc,mc=cycles,lu=’u’)
        >
        > tmp,\_,\_ = poi\_fixeva(pds=messu.Stress,
        > :   p=\_opts[‘OPT\_YM\_Determination\_range’][0:2],
        >     iS=VIP\_messu[tmp\_iS], iE=VIP\_messu[tmp\_iE],
        >     range\_sub=’min’, norm=’max’, option=’abs’,
        >     check\_irr=True, irr=messu[messu.driF\_schg].index.values,
        >     irr\_opt=’nearest’)
        >
        > VIP\_messu[‘FuB’+str(cc)],VIP\_messu[‘FuA’+str(cc)]=tmp

exmecheva.common.mc\_char.poi\_refinement(*DQ*, *B\_ser*, *s\_range*, *B\_ind=None*, *names=['F3', 'M', 'F4']*, *Blp=0.15*, *Bup=0.95*, *Maxp=0.75*, *trimsch=True*)[source]
:   Refine elastic modulus determination range according to a method described
    by Keuerleber [1].
    ([1]: Keuerleber, M. (2006). Bestimmung des Elastizitätsmoduls von
    Kunststoffen bei hohen Dehnraten am Beispiel von PP. Von der Fakultät
    Maschinenbau der Universität Stuttgart zur Erlangung der Würde eines
    Doktor-Ingenieurs (Dr.-Ing.) genehmigte Abhandlung. Doktorarbeit.
    Universität Stuttgart, Stuttgart.)

    Parameters
    :   - **DQ** (*pd.DataFrame*) – Stress-strain-curve differential quotients and sign changes.
          Generated by Diff\_Quot.
        - **B\_ser** (*pd.Series*) – Input data, p.e. stress, to search for given lower limit ratio
          (Blp) und upper limit ratio (Bup) for refined range.
        - **s\_range** (*list*) – Start and end of step range to search for refined range.
        - **B\_ind** (*int**,* *optional*) – Index for B\_ser. The default is None. Not implemented yet
          (using absolute maximum of given B\_ser values instead).
        - **names** (*list* *of* *string**,* *optional*) – Output names for lower limit, maximum of differental quotient
          and upper limit. The default is [‘F3’,’M’,’F4’].
        - **Blp** (*float**,* *optional*) – Lower limit ratio to identified B\_ser value (p.e. maximum stress).
          The default is 0.15.
        - **Bup** (*float**,* *optional*) – Upper limit ratio to identified B\_ser value (p.e. maximum stress).
          The default is 0.95.
        - **Maxp** (*float**,* *optional*) – Lowest value to include in refined range. The default is 0.75.
          (i.e. 75 % of the maximum differntail quotient)
        - **trimsch** (*bool**,* *optional*) – Switch for trimming step range of differential quotients.
          The default is True.

    Returns
    :   - **VIP** (*pd.Series*) – Determined points of interest as series of steps indexed by given names.
        - **DQs** (*pd.DataFrame*) – Differential quotients limitet to range.

    Example

    DQcons=None
    DQopts=None
    for cc in np.arange(1,cycles+1):

    > tmp\_iS=poi\_vip\_namer(VIP=VIP\_messu,ttype=\_opts[‘OPT\_Testtype’],
    > :   key=\_opts[‘OPT\_YM\_Determination\_refinement’][2],
    >     cc=cc,mc=cycles,lu=’l’)
    >
    > tmp\_iE=poi\_vip\_namer(VIP=VIP\_messu,ttype=\_opts[‘OPT\_Testtype’],
    > :   key=\_opts[‘OPT\_YM\_Determination\_refinement’][3],
    >     cc=cc,mc=cycles,lu=’l’)
    >
    > tmp,DQcont = poi\_refinement(DQ=DQcon, B\_ser=messu.loc(axis=1)[‘Stress’],
    > :   s\_range=[VIP\_messu[tmp\_iS],VIP\_messu[tmp\_iE]],
    >     names=[‘RlA’+str(cc),’RlM’+str(cc),’RlB’+str(cc)],
    >     Blp=\_opts[‘OPT\_YM\_Determination\_refinement’][0],
    >     Bup=1.0-\_opts[‘OPT\_YM\_Determination\_refinement’][0],
    >     Maxp=\_opts[‘OPT\_YM\_Determination\_refinement’][1])
    >
    > VIP\_messu=tmp.combine\_first(VIP\_messu).sort\_values().astype(int)
    > if DQcons is None:
    >
    > > DQcons = DQcont
    >
    > else:
    > :   DQcons=pd.concat([DQcons,DQcont],axis=0)
    >
    > tmp,DQoptt = poi\_refinement(DQ=DQopt, B\_ser=messu.loc(axis=1)[‘Stress’],
    > :   s\_range=[VIP\_messu[tmp\_iS],VIP\_messu[tmp\_iE]],
    >     names=[‘RlA’+str(cc),’RlM’+str(cc),’RlB’+str(cc)],
    >     Blp=\_opts[‘OPT\_YM\_Determination\_refinement’][0],
    >     Bup=1.0-\_opts[‘OPT\_YM\_Determination\_refinement’][0],
    >     Maxp=\_opts[‘OPT\_YM\_Determination\_refinement’][1])
    >
    > VIP\_dicu=tmp.combine\_first(VIP\_dicu).sort\_values().astype(int)
    > if DQopts is None:
    >
    > > DQopts = DQoptt
    >
    > else:
    > :   DQopts=pd.concat([DQopts,DQoptt],axis=0)
    >
    > if not ((‘destructive’ in \_opts[‘OPT\_Testtype’]) and cc==cycles):
    > :   tmp\_iS=poi\_vip\_namer(VIP=VIP\_messu,ttype=\_opts[‘OPT\_Testtype’],
    >     :   key=\_opts[‘OPT\_YM\_Determination\_refinement’][3],
    >         cc=cc,mc=cycles,lu=’u’)
    >
    >     tmp\_iE=poi\_vip\_namer(VIP=VIP\_messu,ttype=\_opts[‘OPT\_Testtype’],
    >     :   key=\_opts[‘OPT\_YM\_Determination\_refinement’][2],
    >         cc=cc,mc=cycles,lu=’u’)
    >
    >     tmp,DQcont = poi\_refinement(DQ=DQcon, B\_ser=messu.loc(axis=1)[‘Stress’],
    >     :   s\_range=[VIP\_messu[tmp\_iS],VIP\_messu[tmp\_iE]],
    >         names=[‘RuA’+str(cc),’RuM’+str(cc),’RuB’+str(cc)],
    >         Blp=\_opts[‘OPT\_YM\_Determination\_refinement’][0],
    >         Bup=1.0-\_opts[‘OPT\_YM\_Determination\_refinement’][0],
    >         Maxp=\_opts[‘OPT\_YM\_Determination\_refinement’][1])
    >
    >     VIP\_messu=tmp.combine\_first(VIP\_messu).sort\_values().astype(int)
    >     if DQcons is None:
    >
    >     > DQcons = DQcont
    >
    >     else:
    >     :   DQcons=pd.concat([DQcons,DQcont],axis=0)
    >
    >     tmp,DQoptt = poi\_refinement(DQ=DQopt, B\_ser=messu.loc(axis=1)[‘Stress’],
    >     :   s\_range=[VIP\_messu[tmp\_iS],VIP\_messu[tmp\_iE]],
    >         names=[‘RuA’+str(cc),’RuM’+str(cc),’RuB’+str(cc)],
    >         Blp=\_opts[‘OPT\_YM\_Determination\_refinement’][0],
    >         Bup=1.0-\_opts[‘OPT\_YM\_Determination\_refinement’][0],
    >         Maxp=\_opts[‘OPT\_YM\_Determination\_refinement’][1])
    >
    >     VIP\_dicu=tmp.combine\_first(VIP\_dicu).sort\_values().astype(int)
    >     if DQopts is None:
    >
    >     > DQopts = DQoptt
    >
    >     else:
    >     :   DQopts=pd.concat([DQopts,DQoptt],axis=0)

exmecheva.common.mc\_char.poi\_rel\_finder(*cip*, *cycles*, *xser*, *df*, *dx=None*, *POI\_start='P'*, *POI\_sec=['l', 'u']*, *comp\_dfx='x'*, *rel\_df='DQ2'*, *ascending=True*)[source]
:   Finds the relevant point from several given points. For example, if there
    are several rise changes (e.g. due to measurement deviation), an attempt
    is made to find the actual one.

    Parameters
    :   - **cip** (*pd.Series* *(**(**interim-**)**result* *of* *poi\_det\_plh**)*) – Points of interest, with index by names and value by index of input.
          data. Labels are ‘H’-high point, ‘L’-low point, ‘Pl’-rise change on
          loading and ‘Pu’-rise change on unloading.
        - **cycles** (*int*) – Number of cyclic loadings.
        - **xser** (*pd.Series*) – Input data on abcissa, p.e. time.
        - **df** (*pandas Dataframe* *(**last result* *of* *curve\_characterizer**)**,* *optional*) – Input, as well as differential quotients.
        - **dx** (*float* *or* *None**,* *optional*) – Test range. The default is None.
        - **POI\_start** (*str**,* *optional*) – Start of point of interest name. The default is ‘P’.
        - **POI\_sec** (*list* *of* *strings**,* *optional*) – Second part of point of interest name. The default is [‘l’,’u’].
        - **comp\_dfx** (*string**,* *optional*) – Comparision data on df. The default is ‘x’.
        - **rel\_df** (*string**,* *optional*) – Relevant data on df for testing (DQ1=ris and DQ2=curve).
          The default is ‘DQ2’.
        - **ascending** (*bool**,* *optional*) – Switch for ascending or descending. The default is True.

    Returns
    :   **Vren** – Renamer for relevant points.

    Return type
    :   pd.Series

    Example

    Vren=poi\_rel\_finder(cip=cip, cycles=cycles, xser=x,
    :   df=dft, dx=x.diff().mean()\*(det\_dist),
        POI\_start=’P’, POI\_sec=[‘l’,’u’],
        comp\_dfx=’x’, rel\_df=’DQ2’, ascending=True)

    cip.index=cip.index.to\_series().replace(Vren).values

exmecheva.common.mc\_char.poi\_vip\_namer(*VIP*, *ttype*, *key*, *cc=1*, *mc=1*, *lu='l'*)[source]
:   Automatic naming of points of interest to use before poi\_fixeva and
    poi\_refinement. Replaces given string by its equivalent for current cycle.
    For example if key is ‘P’ (may be lower edge of elastic modulus
    determination range) and lu=’l’ it will find ‘Pl1’ for the first cycle and
    ‘L3’ for the third cycle.

    Parameters
    :   - **VIP** (*pd.Series*) – Identified points of interest. Index by name and value by index of
          measurement dataframe.
        - **ttype** (*string*) – Test type, i.e. type of applied loading. Searches only for ‘cyclic’
          and ‘destructive’.
        - **key** (*string*) – Label for naming, possible are ‘H’-high point, ‘L’-low point,
          ‘P’-rise change and ‘S’-start.
        - **cc** (*int**,* *optional*) – Current cycle. The default is 1.
        - **mc** (*int**,* *optional*) – maximum applied cycles. The default is 1.
        - **lu** (*string**,* *optional*) – Loading (‘l’) or unloading (‘u’). The default is ‘l’.

    Returns
    :   Found name of point of interest.

    Return type
    :   string

    Example

    for cc in np.arange(1,cycles+1):
    :   tmp\_iS=poi\_vip\_namer(VIP=VIP\_messu,ttype=\_opts[‘OPT\_Testtype’],
        :   key=\_opts[‘OPT\_YM\_Determination\_range’][-2],
            cc=cc,mc=cycles,lu=’l’)

        tmp\_iE=poi\_vip\_namer(VIP=VIP\_messu,ttype=\_opts[‘OPT\_Testtype’],
        :   key=\_opts[‘OPT\_YM\_Determination\_range’][-1],
            cc=cc,mc=cycles,lu=’l’)

        tmp,\_,\_ = poi\_fixeva(pds=messu.Stress,
        :   p=\_opts[‘OPT\_YM\_Determination\_range’][0:2],
            iS=VIP\_messu[tmp\_iS], iE=VIP\_messu[tmp\_iE],
            range\_sub=’min’, norm=’max’, option=’abs’,
            check\_irr=True, irr=messu[messu.driF\_schg].index.values,
            irr\_opt=’nearest’)

        VIP\_messu[‘FlA’+str(cc)],VIP\_messu[‘FlB’+str(cc)]=tmp
        if not ((‘destructive’ in \_opts[‘OPT\_Testtype’]) and cc==cycles):

        > tmp\_iS=poi\_vip\_namer(VIP=VIP\_messu,ttype=\_opts[‘OPT\_Testtype’],
        > :   key=\_opts[‘OPT\_YM\_Determination\_range’][-1],
        >     cc=cc,mc=cycles,lu=’u’)
        >
        > tmp\_iE=poi\_vip\_namer(VIP=VIP\_messu,ttype=\_opts[‘OPT\_Testtype’],
        > :   key=\_opts[‘OPT\_YM\_Determination\_range’][-2],
        >     cc=cc,mc=cycles,lu=’u’)
        >
        > tmp,\_,\_ = poi\_fixeva(pds=messu.Stress,
        > :   p=\_opts[‘OPT\_YM\_Determination\_range’][0:2],
        >     iS=VIP\_messu[tmp\_iS], iE=VIP\_messu[tmp\_iE],
        >     range\_sub=’min’, norm=’max’, option=’abs’,
        >     check\_irr=True, irr=messu[messu.driF\_schg].index.values,
        >     irr\_opt=’nearest’)
        >
        > VIP\_messu[‘FuB’+str(cc)],VIP\_messu[‘FuA’+str(cc)]=tmp

exmecheva.common.mc\_char.rise\_curve(*meas\_curve*, *smoothbool*, *smooth\_lvl*)[source]
:   Computes partial integration (1st[rise] and 2nd-grade[curvature])
    to find points of inconstancy in measured curves

exmecheva.common.mc\_char.test\_pdmon(*df*, *cols*, *m*, *dist*)[source]
:   Test of monotonic de-/increasing in dataframe columns.

    Parameters
    :   - **df** (*pd.DataFrame*) – Dataframe which includes cols as Column-names.
        - **cols** (*string* *or* *array* *of* *strings*) – Column-names for monotonic test.
        - **m** (*np.int*) – Kind of monotoni (1=increasing, -1=decreasing).
        - **dist** (*np.int*) – test distance of monotonic creasing.

    Returns
    :   **d** – Series with index ‘cols’ of last ‘df.index’ with monotonic de-/increasing of ‘dist’-length.

    Return type
    :   pd.Series([],dtype=’float64’)

## exmecheva.common.mc\_man module

Adds functionality to temporary manipulate measurement curves.

@author: MarcGebhardt

exmecheva.common.mc\_man.DetFinSSC(*mdf*, *YM*, *iS*, *iLE=None*, *StressN='Stress'*, *StrainN='Strain'*, *addzero=True*, *izero=None*, *option='YM'*)[source]
:   Determine final stress-strain curve (moved to strain offset from elastic modulus).

    Parameters
    :   - **mdf** (*pd.DataFrame*) – Original stress-strain curve data.
        - **YM** (*dict* *or* *pd.Series* *or* *float*) – Elastic modulus (float) or
          elastic modulus (key=E) and intersection on strain=0 (key=Eabs) or
          direct input of strain offset (if option is in [‘strain\_offset’,’SO’,’so’]).
        - **iS** (*index* *of* *mdf*) – Start of linear behavior (all values befor will dropped).
        - **iLE** (*TYPE**,* *optional*) – End of linear behavior (if None, strain offset will determined only with iS).
          The default is None.
        - **StressN** (*string**,* *optional*) – Column name for stress variable. The default is ‘Stress’.
        - **StrainN** (*TYPE**,* *optional*) – Column name for strain variable. The default is ‘Strain’.
        - **addzero** (*bool**,* *optional*) – Switch to adding Zero value (index,StressN,StrainN=0).
          The default is True.
        - **izero** (*index* *of* *mdf**,* *optional*) – Index for zero value line (p.e. to match old start index)
        - **option** (*string**,* *optional*) –

          Option for strain offset. Possible:
          :   - ’YM’: Strain offset determined by elastic modulus.
              - [‘strain\_offset’,’SO’,’so’]: Direct input of strain offset.

          The default is ‘YM’.

    Returns
    :   - **out** (*pd.DataFrame*) – Moved stress-strain curve data.
        - **so** (*float*) – Strain offset.

exmecheva.common.mc\_man.Diff\_ext(*x*, *periods=1*, *axis=0*, *pfunc=<function Extend\_Series\_Poly>*, *pkwargs={}*, *shift\_value=0*, *fill\_value=None*)[source]
:   Calculates the difference of input values after extension and retrims.

    Parameters
    :   - **x** (*pd.Series* *or* *pd. DataFrame*) – Input values.
        - **periods** (*int**,* *optional*) – Periods to shift (see pandas diff). The default is 1.
        - **axis** (*integer**,* *optional*) – Axis to apply. The default is 0.
        - **pfunc** (*function**,* *optional*) – Extension function. The default is Extend\_Series\_Poly.
        - **pkwargs** (*dict**,* *optional*) –

          Keyword arguments passed to extening funtion (pfunc).
          :   The default is {}.
        - **shift\_value** (*int**,* *optional*) – Value for shifting. The default is 0.
        - **fill\_value** (*int* *or* *None**,* *optional*) –

          Value for filling free values after shifting.
          :   The default is None, which apply self.dtype.na\_value.

    Raises
    :   **TypeError** – DESCRIPTION.

    Returns
    :   **xout** – Output values.

    Return type
    :   pd.Series or pd. DataFrame

exmecheva.common.mc\_man.Extend\_Series\_Poly(*y*, *n=3*, *polydeg=1*, *kind='fb'*)[source]
:   Extend an Array or series with polynomial values.

    Parameters
    :   - **y** (*numpy.array* *or* *pandas.Series*) – Input values.
        - **n** (*int* *or* *list* *of* *or dict* *of* *kind{'f':int**,**'b':int}*) – Number of points to extend.
          Must be at least equal to the order of the polynomial.
          Could be 0 (skipping extension).
        - **polydeg** (*int* *or* *list* *of* *or dict* *of* *kind{'f':int**,**'b':int}**,* *optional*) – Order of polynom. The default is 1.
        - **kind** (*str**,* *optional*) – Kind of extension (‘f’-forward, ‘b’-backward and ‘fb’-both).
          The default is ‘fb’.

    Raises
    :   **TypeError** – DESCRIPTION.

    Returns
    :   **yout** – Extended input values.

    Return type
    :   numpy.array or pandas.Series

exmecheva.common.mc\_man.Extend\_Series\_n\_setter(*ffunctype='Smooth'*, *ffuncargs=()*, *ffunckwargs={}*)[source]
:   Sets window length for extending of series by given aspiired function.

    Parameters
    :   - **ffunctype** (*function**,* *optional*) – Function to use after extension. The default is ‘Smooth’.
        - **ffuncargs** (*list**,* *optional*) – Arguments for function. The default is ().
        - **ffunckwargs** (*dict**,* *optional*) – Keyword arguments for function. The default is {}.

    Raises
    :   **ValueError** – No valid value found for extension length.

    Returns
    :   **ntmp** – Extension length.

    Return type
    :   integer

exmecheva.common.mc\_man.Predict\_apply\_retrim(*x*, *afunc=None*, *pfunc=<function Extend\_Series\_Poly>*, *aargs=[]*, *akwargs={}*, *pargs=[]*, *pkwargs={'kind': 'fb'*, *'n': 3*, *'polydeg': 1}*, *shift\_value=0*, *fill\_value=None*)[source]
:   Predict input over their boundary values, apply a function and
    retrim to original size.

    Parameters
    :   - **x** (*numpy.array* *or* *pandas.Series*) – Input values.
        - **afunc** (*function**,* *optional*) –

          Function for manipulation to apply after extending the input values.
          :   The default is None.
        - **pfunc** (*function**,* *optional*) –

          Function for extending the input values.
          :   The default is Extend\_Series\_Poly.
        - **aargs** (*list**,* *optional*) –

          Arguments passed to manipulation funtion (afunc).
          :   The default is [].
        - **akwargs** (*dict**,* *optional*) –

          Keyword arguments passed to manipulation funtion (afunc).
          :   The default is {}.
        - **pargs** (*list**,* *optional*) –

          Arguments passed to extening funtion (pfunc).
          :   The default is [].
        - **pkwargs** (*dict**,* *optional*) –

          Keyword arguments passed to extening funtion (pfunc).
          :   The default is {‘n’:1, ‘polydeg’:1, ‘kind’:’fb’}.
        - **shift\_value** (*int**,* *optional*) – Value for shifting. The default is 0.
        - **fill\_value** (*int* *or* *None**,* *optional*) –

          Value for filling free values after shifting.
          :   The default is None, which apply self.dtype.na\_value.

    Raises
    :   **TypeError** – Not expected data type.

    Returns
    :   **xout** – Manipualted output values.

    Return type
    :   numpy.array or pandas.Series

exmecheva.common.mc\_man.Retrim\_Series(*y*, *axis=0*, *n=0*, *kind='fb'*)[source]
:   Retrim an Array or series after extension.

    Parameters
    :   - **y** (*numpy.array* *or* *pandas.Series* *or* *pandas.DataFrame*) – Input values.
        - **n** (*int* *or* *list* *of* *or dict* *of* *kind{'f':int**,**'b':int}*) – Number of points which are extend.
          Could be 0 (skip retrimming).
        - **kind** (*str**,* *optional*) – Kind of extension (‘f’-front, ‘b’-back and ‘fb’-both).
          The default is ‘fb’.

    Raises
    :   **TypeError** – DESCRIPTION.

    Returns
    :   **yout** – Extended input values.

    Return type
    :   numpy.array or pandas.Series

exmecheva.common.mc\_man.Smoothsel(*x*, *smooth\_type='SMA'*, *smooth\_opts={'mode': 'nearest', 'window\_length': 3}*, *snip=False*, *conv\_method='scipy'*)[source]
:   Computes a smoothed version of an input array, according to different
    smoothing types.

    Parameters
    :   - **x** (*array* *or* *pandas.Series* *of* *float*) – Input values.
        - **smooth\_type** (*string**,* *case-sensitive**,* *optional*) –

          Choosen smoothing type.
          Optional. The defalut is ‘SMA’.
          Possible are:

          > - ’SMA’: Simple moving average based on numpy.convolve.
          > - ’BMA’: Moving average with binomial coefficents based on numpy.convolve.
          > - ’SMA\_f1d’: Simple moving average based scipy.ndimage.filters.uniform\_filter1d.
          > - ’SavGol’: Savitzky-Golay filter based on scipy.signal.savgol\_filter.
        - **smooth\_opts** (*dict**,* *optional*) – Keywords and values to pass to smoothing method.
          For further informations see smooth\_type and linked methods.
          Optional. The defalut is {‘window\_length’:3, ‘mode’:’nearest’}.
        - **snip** (*bool* *or* *integer**,* *optional*) – Trimming of output. Either, if True with (window\_length-1)//2 in smooth\_opts,
          none if False, or with inserted distance.
          The default is False.
        - **conv\_method** (*string**,* *optional*) –

          Convolve method to use (‘numpy’ for numpy.convolve,
          :   ’scipy’ for scipy.ndimage.convolve1d)

          The default is ‘scipy’.

    Raises
    :   - **TypeError** – Type not expected.
        - **NotImplementedError** – Not implemented.

    Returns
    :   **out** – Output values.

    Return type
    :   array or pandas.Series of float

exmecheva.common.mc\_man.Smoothsel\_ext(*x, axis=0, smooth\_type='SMA', smooth\_opts={'mode': 'nearest', 'window\_length': 3}, snip=False, conv\_method='scipy', pfunc=<function Extend\_Series\_Poly>, pkwargs={}, shift\_value=0, fill\_value=None, so\_idks=['window\_length', 'size', 'box', 'box\_pts']*)[source]
:   Smoothes extended data and retrimes afterwards.

    Parameters
    :   - **x** (*pd.Series* *or* *pd.DataFrame*) – Input data.
        - **axis** (*int**,* *optional*) – 2D axis to apply. The default is 0.
        - **smooth\_type** (*string**,* *case-sensitive**,* *optional*) –

          Choosen smoothing type.
          Optional. The defalut is ‘SMA’.
          Possible are:

          > - ’SMA’: Simple moving average based on numpy.convolve.
          > - ’BMA’: Moving average with binomial coefficents based on numpy.convolve.
          > - ’SMA\_f1d’: Simple moving average based scipy.ndimage.filters.uniform\_filter1d.
          > - ’SavGol’: Savitzky-Golay filter based on scipy.signal.savgol\_filter.
        - **smooth\_opts** (*dict**,* *optional*) – Keywords and values to pass to smoothing method.
          For further informations see smooth\_type and linked methods.
          Optional. The defalut is {‘window\_length’:3, ‘mode’:’nearest’}.
        - **snip** (*bool* *or* *integer**,* *optional*) – Trimming of output. Either, if True with (window\_length-1)//2 in smooth\_opts,
          none if False, or with inserted distance.
          The default is False.
        - **conv\_method** (*string**,* *optional*) –

          Convolve method to use (‘numpy’ for numpy.convolve,
          :   ’scipy’ for scipy.ndimage.convolve1d)

          The default is ‘scipy’.
        - **pfunc** (*function**,* *optional*) – Function for extension. The default is Extend\_Series\_Poly.
        - **pkwargs** (*dict**,* *optional*) – Keywords for extension function (pfunc). The default is {}.
        - **shift\_value** (*int**,* *optional*) – Value for shifting. The default is 0.
        - **fill\_value** (*int* *or* *None**,* *optional*) –

          Value for filling free values after shifting.
          :   The default is None, which apply self.dtype.na\_value.
        - **so\_idks** (*list* *of* *string**,* *optional*) – Smoothing options Keywords to search for extension length.
          The default is [‘window\_length’,’size’,’box’,’box\_pts’].

    Raises
    :   - **TypeError** – Input type error.
        - **ValueError** – No extension length found.

    Returns
    :   **xout** – Output data.

    Return type
    :   pd.Series or pd.DataFrame

exmecheva.common.mc\_man.check\_params(*test\_val*, *test\_var='n'*, *func=<function Extend\_Series\_Poly>*, *args=()*, *kwargs={'polydeg': 1}*)[source]
:   Checkes if tested values merge with given function.

    Parameters
    :   - **test\_val** (*TYPE*) – Variable value to test.
        - **test\_var** (*TYPE**,* *optional*) – Variable name. The default is ‘n’.
        - **func** (*function**,* *optional*) –

          Function to test. Implemented are:
          :   - Extend\_Series\_Poly

          The default is Extend\_Series\_Poly.
        - **args** (*list**,* *optional*) – Arguments for function. The default is ().
        - **kwargs** (*dict**,* *optional*) – Keyword arguments for function. The default is {‘polydeg’:1}.

    Raises
    :   **NotImplementedError** – Function not implemented.

    Returns
    :   - **leg\_val** (*bool*) – Indicates if the input value is legitimate.
        - **adj\_val** (*TYPE*) – Adjusted value.
        - **leg\_str** (*TYPE*) – Output string.

exmecheva.common.mc\_man.mc\_resampler(*mdf*, *t\_col='Time'*, *resample=True*, *res\_frequ=4*, *move\_ave=True*, *ma\_sampler='data\_rf'*, *rel\_time\_digs=2*)[source]
:   Resample measured data to aspired frequancy, with or without moving
    average.
    Only tested with downsampling and integer frequency;
    fixed time format ‘s’ and 3 digits for resampling.
    TODO: Implement mc\_smoothing.Smoothsel\_ext

    > (extending, smoothing and retrimming).

    Parameters
    :   - **mdf** (*pd.DataFrame*) – Time depend measured data.
        - **t\_col** (*string**,* *optional*) – Name of time column. The default is ‘Time’.
        - **resample** (*bool**,* *optional*) – Resampling switch. The default is True.
        - **res\_frequ** (*int**,* *optional*) – Aspired output frequency. The default is 4.
        - **move\_ave** (*bool**,* *optional*) – Switch for moving average. The default is True.
        - **ma\_sampler** (*string* *or* *int**,* *optional*) – Sampler for moving average. Can ether be option ‘data\_rf’
          (sampler derived from quotient of present to aspired frequency) or
          integer number. The default is ‘data\_rf’.
        - **rel\_time\_digs** (*int**,* *optional*) – Relevant digits for time variable. The default is 2.

    Returns
    :   **mdf** – Resampled measured data.

    Return type
    :   pd.DataFrame

exmecheva.common.mc\_man.smooth(*y*, *box\_pts*)[source]
:   Computes moving average of y about distance of box\_pts.

    Parameters
    :   - **y** (*array* *of* *float*) – Input data.
        - **box\_pts** (*integer*) – Window length.

    Returns
    :   **y\_smooth** – Output data.

    Return type
    :   array of float

## exmecheva.common.mc\_yield module

Created on Tue Dec 5 12:36:27 2023

@author: mgebhard

exmecheva.common.mc\_yield.Find\_intg2p(*gsl*, *gin*, *pdo*, *i1=None*, *i2=None*, *x='Strain'*, *y='Stress'*, *so=0*, *n\_yield='Y'*)[source]
:   Determine intersection of line and point (usefull to find yield point)

exmecheva.common.mc\_yield.YM\_eva\_range\_refine(*m\_df*, *VIP*, *n\_strain*, *n\_stress*, *n\_loBo='S'*, *n\_upBo='U'*, *d\_loBo=0.05*, *d\_max=0.75*, *rise\_det=[True, 4]*, *n\_Outlo='F3'*, *n\_Outmi='FM'*, *n\_Outhi='F4'*)[source]
:   Refines the Youngs Modulus determinition range according to
    “Keuerleber, M. (2006) - Bestimmung des Elastizitätsmoduls von Kunststoffen
    bei hohen Dehnraten am Beispiel von PP. Von der Fakultät Maschinenbau der
    Universität Stuttgart zur Erlangung der Würde eines Doktor-Ingenieurs (Dr.-Ing.)
    genehmigte Abhandlung. Doktorarbeit. Universität Stuttgart, Stuttgart”

    Parameters
    :   - **m\_df** (*pd.DataFrame*) – Measured data.
        - **VIP** (*pd.Series*) – Important points corresponding to measured data.
        - **n\_strain** (*string*) – Name of used strain (have to be in measured data).
        - **n\_stress** (*string*) – Name of used stress (have to be in measured data).
        - **n\_loBo** (*string*) – Lower border for determination (have to be in VIP). The default is ‘S’.
        - **n\_upBo** (*string*) – Upper border for determination (have to be in VIP). The default is ‘U’.
        - **d\_loBo** (*float/str**,* *optional*) – When float: Percentage of range between n\_upBo and n\_loBo as start distance to n\_loBo.
          When str starting with ‘S’, followed by integer: Distance in steps to n\_loBo.
          The default is 0.05.
        - **d\_max** (*float**,* *optional*) – Percentage of . The default is 0.75.
        - **rise\_det** (*[**bool**,* *int**]**,* *optional*) – Determination options for stress rising ([smoothing, smoothing factor]).
        - **n\_Outlo** (*string**,* *optional*) – Name of new lower border. The default is ‘F3’.
        - **n\_Outmi** (*string**,* *optional*) – Name of maximum differential quotient. The default is ‘FM’.
        - **n\_Outhi** (*string**,* *optional*) – Name of new upper border. The default is ‘F4’.

    Yields
    :   - **VIP\_new** (*pd.Series*) – Important points corresponding to measured data.
        - **txt** (*string*) – Documantation string.

exmecheva.common.mc\_yield.Yield\_redet(*m\_df*, *VIP*, *n\_strain*, *n\_stress*, *n\_loBo*, *n\_upBo*, *n\_loBo\_int*, *YM*, *YM\_abs*, *strain\_offset=0.002*, *rise\_det=[True, 4]*, *n\_yield='Y'*)[source]
:   Redetermine yield point to different conditions
    (intersection with linearised strain offset (ones after), zero rising, fixed endpoint).

    Parameters
    :   - **m\_df** (*pd.DataFrame*) – Measured data.
        - **VIP** (*pd.Series*) – Important points corresponding to measured data.
        - **n\_strain** (*string*) – Name of used strain (have to be in measured data).
        - **n\_stress** (*string*) – Name of used stress (have to be in measured data).
        - **n\_loBo** (*[**string**]*) – List of lower borders for determination (have to be in VIP).
        - **n\_upBo** (*[**string**]*) – List of upper borders for determination (have to be in VIP).
        - **n\_loBo\_int** (*[**string**]*) – List of lower borders for interseption (have to be in VIP).
        - **YM** (*float*) – Youngs Modulus.
        - **YM\_abs** (*float*) – Absolut value of Youngs Modulus.
        - **strain\_offset** (*float**,* *optional*) – Strain offset (eq. plastic strain). The default is -0.002.
        - **rise\_det** (*[**bool**,* *int**]**,* *optional*) – Determination options for stress rising ([smoothing, smoothing factor]).
          The default is [True,4].
        - **n\_yield** (*string**,* *optional*) – Name of yield point (have to be in VIP). The default is ‘Y’.

    Returns
    :   - **VIP\_new** (*pd.Series*) – Important points corresponding to measured data.
        - **txt** (*string*) – Documantation string.

exmecheva.common.mc\_yield.Yield\_redet2(*m\_df*, *VIP*, *n\_strain*, *n\_stress*, *n\_loBo*, *n\_upBo*, *n\_loBo\_int*, *YM*, *YM\_abs*, *strain\_offset=0.002*, *use\_rd=True*, *rise\_det=[True, 4]*, *n\_yield='Y'*, *ywhere='n'*)[source]
:   Redetermine yield point to different conditions
    (intersection with linearised strain offset (ones after), zero rising, fixed endpoint).

    Parameters
    :   - **m\_df** (*pd.DataFrame*) – Measured data.
        - **VIP** (*pd.Series*) – Important points corresponding to measured data.
        - **n\_strain** (*string*) – Name of used strain (have to be in measured data).
        - **n\_stress** (*string*) – Name of used stress (have to be in measured data).
        - **n\_loBo** (*[**string**]*) – List of lower borders for determination (have to be in VIP).
        - **n\_upBo** (*[**string**]*) – List of upper borders for determination (have to be in VIP).
        - **n\_loBo\_int** (*[**string**]*) – List of lower borders for interseption (have to be in VIP).
        - **YM** (*float*) – Youngs Modulus.
        - **YM\_abs** (*float*) – Absolut value of Youngs Modulus.
        - **strain\_offset** (*float**,* *optional*) – Strain offset (eq. plastic strain). The default is -0.002.
        - **use\_rd** (*bool**,* *optional*) – Switch for using change in stress rising. The default is True.
        - **rise\_det** (*[**bool**,* *int**]**,* *optional*) – Determination options for stress rising ([smoothing, smoothing factor]).
          The default is [True,4].
        - **n\_yield** (*string**,* *optional*) – Name of yield point (have to be in VIP). The default is ‘Y’.
        - **ywhere** (*string**,* *optional*) – Which point should be choosen (n-next, a-after, b-before). The default is ‘n’.

    Returns
    :   - **VIP\_new** (*pd.Series*) – Important points corresponding to measured data.
        - **txt** (*string*) – Documantation string.

exmecheva.common.mc\_yield.Yield\_redet2\_Multi(*m\_df*, *VIP*, *YM*, *YM\_abs*, *strain\_osd={'Y': 0.002, 'Y0': 0.0, 'Y1': 7.000000000000001e-05, 'YK': 0.0}*, *strain\_osdf={'YK': 'F4'}*, *n\_strain='Strain'*, *n\_stress='Stress'*, *n\_loBo=['F3']*, *n\_upBo=['U']*, *n\_loBo\_int=['F3']*, *use\_rd=True*, *rise\_det=[True, 2]*, *ywhere='n'*)[source]

## exmecheva.common.output module

Functions for output data generation.

@author: MarcGebhardt

exmecheva.common.output.Otvalgetter\_Multi(*mdf*, *Vs=['Y', 'YK', 'Y0', 'Y1', 'U', 'B']*, *datasep=['con', 'opt']*, *VIPs={'con': None, 'opt': None}*, *exacts={'con': None, 'opt': None}*, *orders={'con': ['f', 'e', 'U'], 'opt': ['e', 'U']}*, *add\_esufs={'con': '\_con', 'opt': '\_opt'}*, *n\_strains={'con': 'Strain', 'opt': 'DStrain'}*, *n\_stresss={'con': 'Stress', 'opt': 'Stress'}*, *use\_exacts=True*)[source]
:   Wrapper for Outvalgetter.

    Parameters
    :   - **mdf** (*TYPE*) – DESCRIPTION.
        - **Vs** (*list* *of* *strings**,* *optional*) – Relevant VIPs (have to be in VIP).
          The default is [‘Y’,’YK’,’Y0’,’Y1’,’U’,’B’].
        - **datasep** (*list* *of* *strings**,* *optional*) – Data seperation by strain/displacement relation.
          The default is [‘con’,’opt’].
        - **VIPs** (*dict**,* *optional*) – ‘Very important points’. The default is {‘con’:None,’opt’:None}.
        - **exacts** (*dict* *of* *pd.DataFrame**,* *optional*) –

          Exact values (p.e. for yield point) seperated by strain/displacement relation.
          Need to have special format (see common.analyze.Yield\_redet2\_Multi):

          > - index: VIP
          > - columns: [‘strain\_os’,’ind’,’ind\_ex’,n\_strain,n\_stress]

          The default is None.
        - **orders** (*dict* *of* *lists* *of* *strings**,* *optional*) –

          Output order seperated to datasep. Implemented are:
          :   - ’f’: stress
              - ’F’: force
              - ’e’: strain
              - ’s’: displacement
              - ’U’: strain energy / -density
              - ’W’: deformation energy

          The default is {‘con’:[‘f’,’e’,’U’],’opt’:[‘e’,’U’]}.
        - **add\_esufs** (*dict* *of* *strings**,* *optional*) – Suffixes for strain/displacement relation acc. to measurement method.
          The default is {‘con’:’\_con’,’opt’:’\_opt’}.
        - **n\_strains** (*dict* *of* *strings**,* *optional*) – Column name in mdf for strain values.
          The default is {‘con’:’Strain’,’opt’:’DStrain’}.
        - **n\_stresss** (*TYPE**,* *optional*) – Column name in mdf for stress values.
          The default is {‘con’:’Stress’,’opt’:’Stress’}.
        - **use\_exacts** (*bool**,* *optional*) – Switch to use exact values if applicable. The default is True.

    Returns
    :   **out** – Evaluated values.

    Return type
    :   pd.Series

    Examples

    Otvalgetter\_Multi(messu\_FP, Vs=relVS,
    :   datasep=[‘con’,’opt’],
        VIPs={‘con’:VIP\_messu,’opt’:VIP\_dicu},
        exacts={‘con’:yield\_df\_con,’opt’:yield\_df\_opt},
        orders={‘con’:[‘f’,’e’,’U’],’opt’:[‘e’,’U’]},
        add\_esufs={‘con’:’\_con’,’opt’:’\_opt’},
        n\_strains={‘con’:’Strain’,’opt’:dic\_used\_Strain},
        n\_stresss={‘con’:’Stress’,’opt’:’Stress’},
        use\_exacts=True)

exmecheva.common.output.Outvalgetter(*mdf*, *V*, *VIP*, *exacts=None*, *order=['f', 'e', 'U']*, *add\_esuf='\_con'*, *n\_strain='Strain'*, *n\_stress='Stress'*, *use\_exacts=True*)[source]
:   Get output values from given data and options.

    Parameters
    :   - **mdf** (*pd.DataFrame*) – DESCRIPTION.
        - **V** (*string*) – Relevant VIP (have to be in VIP).
        - **VIP** (*list* *of* *string*) – ‘Very important point’.
        - **exacts** (*pd.DataFrame**,* *optional*) –

          Exact values (p.e. for yield point).
          Need to have special format (see common.analyze.Yield\_redet2\_Multi):

          > - index: VIP
          > - columns: [‘strain\_os’,’ind’,’ind\_ex’,n\_strain,n\_stress]

          The default is None.
        - **order** (*list* *of* *string**,* *optional*) –

          Output order. Implemented are:
          :   - ’f’: stress (no suffix)
              - ’F’: force (no suffix)
              - ’e’: strain
              - ’s’: displacement
              - ’U’: strain energy / -density
              - ’W’: deformation energy

          The default is [‘f’,’e’,’U’].
        - **add\_esuf** (*string**,* *optional*) – Suffix for strain/displacement relation acc. to measurement method.
          The default is ‘\_con’.
        - **n\_strain** (*string**,* *optional*) – Column name in mdf for strain values. The default is ‘Strain’.
        - **n\_stress** (*string**,* *optional*) – Column name in mdf for stress values. The default is ‘Stress’.
        - **use\_exacts** (*bool**,* *optional*) – Switch to use exact values if applicable. The default is True.

    Returns
    :   **out** – Evaluated values.

    Return type
    :   pd.Series

exmecheva.common.output.str\_indent(*po*, *indent=3*)[source]
:   Adds indentation to str at each new line.

    Parameters
    :   - **po** (*string* *or* *str callable*) – String to manipulate.
        - **indent** (*int**,* *optional*) – Number of spaces for indentation. The default is 3.

    Returns
    :   **poo** – Output string with indentation.

    Return type
    :   string

exmecheva.common.output.str\_log(*s*, *logfp*, *output\_lvl=1*, *logopt=True*, *printopt=True*)[source]
:   Write lines to log and/or console.
    TODO: add functionality for different backends! (Console/window object)

    Parameters
    :   - **s** (*string*) – String to write or print.
        - **logfp** (*string* *or* *path*) – Open log file.
        - **output\_lvl** (*positiv integer*) – Output level (0=none, 1=only text, 2=additional diagramms).
          The default is 1.
        - **logopt** (*bool*) – Wriet in log file (logfp). The default is True.
        - **printopt** (*bool*) – Display on terminal. The default is True.

## exmecheva.common.pd\_ext module

Contains extensions to pandas functionality (mostly indexing and NaN-handling).

@author: MarcGebhardt

exmecheva.common.pd\_ext.Find\_closest(*pds*, *val*, *iS=None*, *iE=None*, *option='abs'*)[source]
:   Returns the index of the closest value of a Series to a value

exmecheva.common.pd\_ext.Find\_closest\_perc(*pds*, *p*, *iS=None*, *iE=None*, *range\_sub='min'*, *norm='max'*, *option='abs'*)[source]
:   Returns the index of the closest value of a Series
    to a percentage value in a range of this series

exmecheva.common.pd\_ext.Find\_closestv(*pds1*, *pds2*, *val1*, *val2*, *iS=None*, *iE=None*, *option='quad'*)[source]
:   Returns the index of the closest value of two corresponding Series
    to a pair of values

exmecheva.common.pd\_ext.Find\_first\_sc(*pds*, *val*, *iS=None*, *iE=None*, *direction='normal'*, *option='after'*, *exclude\_1st=True*, *nan\_policy='omit'*)[source]
:   Returns the index of the first value of a Series with change in sign to a value

exmecheva.common.pd\_ext.deal\_dupl\_index(*df*, *deal\_dupl\_ind='raise'*)[source]
:   Deal with dupliceted index values according option.

    Parameters
    :   - **df** (*pandas.Series* *or* *pandas.DataFrame*) – Data input.
        - **deal\_dupl\_ind** (*string**,* *optional*) –

          Option to deal with duplicated index entries.
          Possible are:

          > - ’raise’: raises IndexError when finding duplicates.
          > - ’keep’: keep unchanged and give no error.
          > - ’keep-first’: drop all duplicated entries except first one.
          > - ’keep-last’: drop all duplicated entries except first one.
          > - Aggratable function or string accapted by pd.agg: Aggregate duplicated index entries by function.

          The default is ‘raise’.

    Raises
    :   - **IndexError** – Index has duplicates and option set to ‘raise’.
        - **ValueError** – option (deal\_dupl\_ind) is not aggregatable.

    Returns
    :   **dft** – Data output.

    Return type
    :   pandas.Series or pandas.DataFrame

exmecheva.common.pd\_ext.pd\_axischange(*axis*)[source]
:   Change axis (use for pandas object). Index will return columns,
    0 will return 1 and in visa versa.

    Parameters
    :   **axis** (*int in* *[**0**,**1**] or* *string in* *[**index**,**columns**]*) – Axis determiner.

    Returns
    :   **a** – Oposite axis of axis.

    Return type
    :   int or string

exmecheva.common.pd\_ext.pd\_combine\_index(*pd1*, *pd2*, *option='dropna'*)[source]
:   Returns a combined index of two pandas objects (series or dataframe)
    with valide values in both.

exmecheva.common.pd\_ext.pd\_exclnan(*pdo*, *axis=1*)[source]

exmecheva.common.pd\_ext.pd\_find\_index(*ser*, *s*)[source]

exmecheva.common.pd\_ext.pd\_isDF(*pdo*)[source]
:   Tests if object is instance of pandas dataframe

exmecheva.common.pd\_ext.pd\_isSer(*pdo*)[source]
:   Tests if object is instance of pandas series

exmecheva.common.pd\_ext.pd\_limit(*self*, *iS=None*, *iE=None*)[source]
:   Limit a pandas Series or Dataframe to given indexes

    Parameters
    :   - **iS** (*matching type* *of* *self.index**,* *optional*) – Start index. The default is None.
        - **iE** (*matching type* *of* *self.index**,* *optional*) – End index. The default is None.

    Returns
    :   Limited pandas object.

    Return type
    :   pandas.Series or pandas.DataFrame

exmecheva.common.pd\_ext.pd\_nan\_handler(*pdo*, *ind=None*, *axis=0*, *nan\_policy='omit'*)[source]
:   Handles NaN in pandas object according to NaN-policy.

    Parameters
    :   - **pdo** (*pandas.Series* *or* *pandas.DataFrame*) – Input Value.
        - **ind** (*(**same as corresponding axis**)**,* *optional*) – Index limitation on not scanned axis. The default is None.
        - **axis** (*[**0**,**1**]**/**[**"index"**,**"columns"**]**,* *optional*) – Scanned axis. The default is 0.
        - **nan\_policy** (*string**,* *optional*) – Policy to handle NaN. Implemented: omit, raise, interpolate.
          The default is ‘omit’.

    Raises
    :   **ValueError** – Raise error if nan\_policy is raise and NaN’s detected.

    Returns
    :   **pds** – Output value with NaN handled acc. NaN-policy.

    Return type
    :   pandas.Series or pandas.DataFrame

exmecheva.common.pd\_ext.pd\_outsort(*data*, *outsort='ascending'*)[source]
:   Sorts pandas object by given string.

    Parameters
    :   - **data** (*pd.Series* *or* *pd.DataFrame*) – Data to be sorted.
        - **outsort** (*str**,* *optional*) – Option for sorting. Implemented:
          - ascending: [‘A’,’a’,’ascending’,True,’r’,’rising’,’rise’]
          - descending: [‘D’,’d’,’descending’,’f’,’falling’,’fall’]
          The default is ‘ascending’.

    Returns
    :   **dout** – Sortted data.

    Return type
    :   pd.Series or pd.DataFrame

exmecheva.common.pd\_ext.pd\_slice\_index(*index*, *vals*, *option='range'*)[source]
:   Slice an index by given values and option.

    Parameters
    :   - **index** (*pd.Index*) – Index.
        - **vals** (*range* *or* *list*) – Slicing values (list or, lower and upper limits).
        - **option** (*string**,* *optional*) – Option for slicing (range or list). The default is ‘range’.

    Raises
    :   **NotImplementedError** – Option not implemented.

    Returns
    :   **ind\_new** – New index.

    Return type
    :   pd.Index

exmecheva.common.pd\_ext.pd\_trapz(*pdo*, *y=None*, *x=None*, *axis=0*, *nan\_policy='omit'*)[source]
:   Extends usage of np.trapz for pandas with nan policy.

    Parameters
    :   - **pdo** (*pd.DataFrame* *or* *pd.Series*) – Pandas object containing data.
        - **y** (*string**,* *optional*) – Determiner for y-values. Have to be in index or columns (see axis).
          The default is None.
        - **x** (*string**,* *optional*) – Determiner for x-values. Have to be in index or columns (see axis).
          The default is None.
        - **axis** (*int in* *[**0**,**1**] or* *string in* *[**index**,**columns**]**,* *optional*) – Axis determiner to perform action. The default is 0.
        - **nan\_policy** (*string**,* *optional*) – Option for handling NaN-values. The default is ‘omit’.

    Raises
    :   - **ValueError** – NaN handling.
        - **NotImplementedError** – Option for nan\_policy not implemented.

    Returns
    :   **out** – DESCRIPTION.

    Return type
    :   float or series of float

exmecheva.common.pd\_ext.pd\_valid\_index(*val*, *pdo*, *opt='ba'*, *na\_hand='Value'*, *na\_out=None*, *add\_ind=None*)[source]
:   Returning valid index of pandas object in respect to defined option.
    (b=before,a=after,n=nearest,ba=before and after,bna=before, nearest and after)
    Filling not available values according a handler (na\_hand) with
    standard value (na\_out) or a part of an additional index (add\_ind).

exmecheva.common.pd\_ext.pd\_vec\_length(*pdo*, *norm=False*, *norm\_kws={}*, *out='Series'*)[source]
:   Calculate (normalized) vector length and return ether whole series or index

## exmecheva.common.plotting module

Plotting functionality.

@author: MarcGebhardt

exmecheva.common.plotting.curve\_char\_plotter(*cps*, *cip*, *dfp*, *df*, *head=None*, *xlabel=None*, *ylabel\_l=None*, *ylabel\_r=None*, *cco={'y': 'm'}*, *cco\_sc={}*, *ccd={'DQ1': 'b', 'DQ2': 'g', 'DQ3': 'y'}*, *ccd\_sc={'DQ1\_sc': 'b', 'DQ2\_sc': 'g', 'DQ3\_sc': 'y'}*, *cc={'Const': 'b', 'Fall': 'r', 'Neg': 'y', 'Pos': 'm', 'Rise': 'g'}*, *disp\_opt\_DQ='Normalized'*, *do\_kwargs={'norm': 'absmax', 'normadd': 0.5, 'th': 0.05, 'th\_option': 'abs', 'th\_set\_val': 0}*, *limDQ=False*, *limDQvals=[-1.1, 1.1]*)[source]
:   Plotting method for mc\_char.curve\_characterizer results.

    Parameters
    :   - **cps** (*pd.Dataframe* *(**mc\_char.curve\_characterizer output**)*) –

          Characterization of input (for linear parts: linearisation and
          :   intersection to previous included).
        - **cip** (*pd.Series* *(**mc\_char.curve\_characterizer output**)*) – Points of first characteriation (S=Start, E=End, I=Increase, D=Decrease).
        - **dfp** (*pd.Dataframe* *(**mc\_char.curve\_characterizer output**)*) – Maxima and minima of input, as well as differential quotients.
        - **df** (*pd.Dataframe* *(**mc\_char.curve\_characterizer output**)*) – Input, as well as differential quotients.
        - **head** (*string* *or* *None**,* *optional*) – Figure title. The default is None.
        - **xlabel** (*string* *or* *None**,* *optional*) – Label on x-axis. The default is None.
        - **ylabel\_l** (*string* *or* *None**,* *optional*) – Label on left y-axis. The default is None.
        - **ylabel\_r** (*string* *or* *None**,* *optional*) – Label on right y-axis. The default is None.
        - **cco** (*dict**,* *optional*) – Color codes for Max and Min markers in accordance with dfp column name.
          The default is {‘y’:’m’}.
        - **cco\_sc** (*dict**,* *optional*) – Color codes for changes in sign markers in accordance with dfp column
          name. The default is {}.
        - **ccd** (*dict**,* *optional*) – Color codes for differential quotient plotting.
          The default is {‘DQ1’:’b’,’DQ2’:’g’,’DQ3’:’y’}.
        - **ccd\_sc** (*dict**,* *optional*) – Color codes for plotting of differential quotient sign changes.
          The default is {‘DQ1\_sc’:’b’,’DQ2\_sc’:’g’,’DQ3\_sc’:’y’}.
        - **cc** (*ict**,* *optional*) – Color codes for plotting of curve description in cps.
          The default is {‘Const’:’b’,’Rise’:’g’,’Fall’:’r’,’Pos’:’m’,’Neg’:’y’}.
        - **disp\_opt\_DQ** (*string**,* *optional*) – Normalization of differential quotients. The default is ‘Normalized’.
        - **do\_kwargs** (*dict**,* *optional*) –

          Keyword arguments for normalization of differential quotients.
          The default is {‘norm’:’absmax’, ‘normadd’:0.5,

          > ’th’:0.05, ‘th\_option’:’abs’, ‘th\_set\_val’:0}.
        - **limDQ** (*bool**,* *optional*) – Limit plot on y-axis to limDQvals. The default is False.
        - **limDQvals** (*list* *of* *float**,* *optional*) – Lower and upper limits on y-axis for limDQ. The default is [-1.1,1.1].

    Raises
    :   **NotImplementedError** – DESCRIPTION.

    Return type
    :   None.

exmecheva.common.plotting.plt\_add\_DaAnno(*mdf*, *x\_n*, *y\_n*, *VIP*, *ax=None*, *xy\_standard=(-6, 6)*, *xy\_ded={1: (1, 1), 2: (-1, 1), 3: (-1, -1), 4: (1, -1)}*, *pkwargs={'color': 'red', 'label': 'Points of interest', 'linestyle': '', 'marker': 'x'}*, *akwargs={'ha': 'center', 'textcoords': 'offset points', 'va': 'center', 'xycoords': 'data'}*)[source]
:   Adds data annotations to curve.

    Parameters
    :   - **mdf** (*pd.DataFrame*) – DataFrame of measured data.
        - **x\_n** (*string*) – Column name of data to plot on x-axis.
        - **y\_n** (*string*) – Column name of data to plot on y-axis.
        - **VIP** (*pd.Series*) – Points of interest (name as index and index of mdf as values).
        - **ax** (*plt.subplot axis**,* *optional*) – Matplotlib axis object to plot. The default is None.
        - **xy\_standard** (*tuple* *of* *int**,* *optional*) – Standard position of text, coordinates related to data.
          The default is (-6,6).
        - **xy\_ded** (*dict**,* *optional*) – Dictionary with positiioning multipliers to apply to xy\_standard.
          The default is {1:(1,1),2:(-1,1),3:(-1,-1),4:(1,-1)}.
        - **pkwargs** (*dict**,* *optional*) –

          Keyword arguments for plt.plot.
          The default is {‘marker’:’x’, ‘linestyle’:’’,’color’:’red’,

          > ’label’:’Points of interest’}.
        - **akwargs** (*TYPE**,* *optional*) –

          Keyword arguments for plt.annotate.
          The default is {‘xycoords’:’data’, ‘textcoords’:’offset points’,

          > ’ha’:”center”, ‘va’:”center”}.

    Returns
    :   Combined postion and text (for control purposes).

    Return type
    :   pd.DataFrame

exmecheva.common.plotting.plt\_ax\_regfit(*pdo*, *x*, *y=None*, *fit={}*, *ax=None*, *plt\_d=False*, *label\_d='Data'*, *label\_f=False*, *xlabel=None*, *ylabel=None*, *title=None*, *legend=True*, *skws={}*, *lkws={}*, *t\_form={}*)[source]
:   Plots a regression (done by fitting.regfitret) and data (optional).
    Optional legend entry with fitting function.

    Parameters
    :   - **pdo** (*pd.DataFrame*) – Input data.
        - **x** (*str*) – Column name for abscissa data.
        - **y** (*str* *or* *None*) – Column name for ordinate data or none, if plt\_d is False.
          The default is None.
        - **fit** (*pd.DataFrame* *(**result* *of* *fitting.regfitret**)**,* *optional*) – Least square regression fit results. The default is {}.
        - **ax** (*plt.axis* *or* *None**,* *optional*) – Axis to plote result. The default is None.
        - **plt\_d** (*bool**,* *optional*) – Switch for plotting data. The default is False.
        - **label\_d** (*string**,* *optional*) – Label for data plot. The default is ‘Data’.
        - **label\_f** (*bool**,* *optional*) – Label for function plot. The default is False.
        - **xlabel** (*string**,* *optional*) – label for x-axis. The default is None.
        - **ylabel** (*string**,* *optional*) – Label for y-axis. The default is None.
        - **title** (*string**,* *optional*) – Axis title. The default is None.
        - **skws** (*dict**,* *optional*) – Dictionary for scatter plot (data). The default is {}.
        - **lkws** (*dict**,* *optional*) – Dictionary for lineplot (function). The default is {}.
        - **legend** (*bool**,* *optional*) – Switch for showing legend. The default is True.

    Return type
    :   None.

exmecheva.common.plotting.plt\_handle\_suffix(*fig*, *path='foo'*, *tight=True*, *show=True*, *save=True*, *s\_types=['pdf', 'png']*, *clear=True*, *close=True*)[source]
:   Handler for end of plotting procedure.

    Parameters
    :   - **fig** (*matplotlib.pyplot.figure*) – Figure instance.
        - **path** (*str**,* *optional*) – Save path. The default is ‘foo’.
        - **tight** (*bool**,* *optional*) – Tight the layout. The default is True.
        - **show** (*bool**,* *optional*) – Show figure. The default is True.
        - **save** (*bool**,* *optional*) – Save figure. The default is True.
        - **s\_types** (*list* *of* *str**,* *optional*) – Types in which the figure is saved. The default is [“pdf”,”png”].
        - **clear** (*bool**,* *optional*) – Clear figure instance. The default is True.
        - **close** (*bool**,* *optional*) – Close figure window. The default is True.

    Return type
    :   None.

exmecheva.common.plotting.sns\_pointplot\_MMeb(*ax*, *data*, *x*, *y*, *hue=None*, *dodge=0.2*, *join=False*, *palette=None*, *markers=['o', 'P']*, *scale=1*, *barsabove=True*, *capsize=4*, *controlout=False*)[source]
:   Generate Pointplot with errorbars marking minimum and maximum instead of CI.

exmecheva.common.plotting.tick\_label\_inserter(*ax*, *pos=0*, *ins=''*, *axis='both'*)[source]
:   Inserts a string to tick labels on axis (can be switched [‘both’,’x’,’y’]).

exmecheva.common.plotting.tick\_label\_renamer(*ax*, *renamer={}*, *axis='both'*)[source]
:   Renames labels on axis (can be switched [‘both’,’x’,’y’]).

exmecheva.common.plotting.tick\_legend\_renamer(*ax*, *renamer={}*, *title=''*)[source]
:   Renames legend entries on axis and optionally insert a title

## exmecheva.common.stat\_ext module

Created on Mon Dec 4 17:54:05 2023

@author: mgebhard

exmecheva.common.stat\_ext.CD\_rep(*pdo*, *groupby='Series'*, *var='DEFlutoB'*, *det\_met='SM-RRT'*, *outtype='txt'*, *tnform='{:.3e}'*)[source]
:   Calculate critical differences and compare to given mean difference.
    Can provide information on the comparability and/or repeatability of two
    test series.

    Parameters
    :   - **pdo** (*pd.DataFrame*) – Dataframe with groups and values as columns for test.
        - **groupby** (*str*) – Name of groups for statistical test.
        - **var** (*str*) – Name of variable for statistical test.
        - **det\_met** (*string**,* *optional*) –

          Determination method for deriving critical differences (CD).
          The default is ‘SM-RRT’.
          Implemented are (all only implemented for 95 % confidence):

          > - ’SM-RRT’: CD by standard deviation and size
          >
          > (see https://www.methodensammlung-bvl.de/resource/blob/208066/e536126ed1723145e51fc90b12736f5e/planung-und-statistische-auswertung-data.pdf)
          > - ‘CV-DC’: CD by coefficient of variation and maximum
          > (see https://flexikon.doccheck.com/de/Kritische\_Differenz)
          > - ‘CV’: CD by coefficient of variation
          > (see https://link.springer.com/chapter/10.1007/978-3-662-48986-4\_887)
          > - ‘SD’: CD by standard deviation
          > (see https://edoc.hu-berlin.de/bitstream/handle/18452/11713/cclm.1982.20.11.817.pdf?sequence=1
        - **outtype** (*string**,* *optional*) –

          Switch for output. The default is ‘txt’.
          Implemented are:

          > - ’Ser\_all’: pd.Series with all derived results
          > - ’Tuple\_all’: tuple of all derived results
          > - ’txt’: single line text output showing results acc. repeatability
          > - ’Series’: pd.Series with important resulzs
        - **tnform** (*formatcode**,* *optional*) – Format for float in sttestual output. The default is ‘{:.3e}’.

    Raises
    :   **NotImplementedError** – Method not implemented.

    Returns
    :   **out** – Output according to switch (see outtype).

    Return type
    :   pd.Series or tuple or string

exmecheva.common.stat\_ext.CD\_test\_multi(*df*, *group\_main='Series'*, *group\_sub=['A']*, *ano\_Var=['WC\_vol']*, *det\_met='SM-RRT'*, *Transpose=True*)[source]
:   Calculate critical differences and compare to given mean difference for
    groups and subgroups.
    Can provide information on the comparability and/or repeatability of two
    test series.

    Parameters
    :   - **df** (*pd.DataFrame*) – Dataframe with groups, subgroups and values as columns for test.
        - **group\_main** (*str**,* *optional*) – Main group for statistical test. The default is ‘Series’.
        - **group\_sub** (*list* *of* *str**,* *optional*) – Sub-groups for statistical test (p.e. variants). The default is [‘A’].
        - **ano\_Var** (*list* *of* *str**,* *optional*) – Names of variables for statistical test.The default is [‘WC\_vol’].
        - **det\_met** (*string**,* *optional*) –

          Determination method for deriving critical differences (CD).
          The default is ‘SM-RRT’.
          Implemented are (all only implemented for 95 % confidence):

          > - ’SM-RRT’: CD by standard deviation and size
          >
          > (see https://www.methodensammlung-bvl.de/resource/blob/208066/e536126ed1723145e51fc90b12736f5e/planung-und-statistische-auswertung-data.pdf)
          > - ‘CV-DC’: CD by coefficient of variation and maximum
          > (see https://flexikon.doccheck.com/de/Kritische\_Differenz)
          > - ‘CV’: CD by coefficient of variation
          > (see https://link.springer.com/chapter/10.1007/978-3-662-48986-4\_887)
          > - ‘SD’: CD by standard deviation
          > (see https://edoc.hu-berlin.de/bitstream/handle/18452/11713/cclm.1982.20.11.817.pdf?sequence=1
        - **Transpose** (*bool**,* *optional*) – Switch for transposing result. The default is False.

    Returns
    :   **df\_out** – Derived test results (pd.Series({‘MD’:grs\_MD,’CD’:CD, ‘eta’:eta, ‘H0’: MD\_l\_CD})).

    Return type
    :   pd.DataFrame

exmecheva.common.stat\_ext.CImax(*data*, *confidence=0.95*, *method='Seaborn\_Bootstrap'*, *func=<function nanmean>*, *n\_boot=1000*, *axis=None*, *units=None*, *seed=0*)[source]
:   Return only maximum of confidence interval. (Use same seed and n\_boot!)

exmecheva.common.stat\_ext.CImin(*data*, *confidence=0.95*, *method='Seaborn\_Bootstrap'*, *func=<function nanmean>*, *n\_boot=1000*, *axis=None*, *units=None*, *seed=0*)[source]
:   Return only minimum of confidence interval. (Use same seed and n\_boot!)

exmecheva.common.stat\_ext.Corr\_ext(*df*, *method='spearman'*, *sig\_level={0.001: '$^a$', 0.01: '$^b$', 0.05: '$^c$', 0.1: '$^d$'}*, *corr\_round=2*)[source]
:   Performs correlation according to method and significance levels.

    Parameters
    :   - **df** (*pd.DataFrame*) – Input data.
        - **method** (*string**,* *optional*) –

          Correlation method, implemented are:
          :   - Pearson: [‘pearson’,’Pearson’,’P’]
              - Spearman: [‘spearman’, ‘Spearman’,’S’]
              - Kendalltau: [‘kendall’, ‘kendalltau’, ‘Kendall’, ‘Kendalltau’, ‘K’]

          The default is ‘spearman’.
        - **sig\_level** (*list* *or* *dict**,* *optional*) – Significance level for anotation, if list: ascending number of \*´s,
          if dict: dictionary values.
          The default is {0.001:’$^a$’,0.01:’$^b$’,0.05:’$^c$’,0.10:’$^d$’}.
        - **corr\_round** (*int* *or* *None**,* *optional*) – Number of digits for rounding of annotation of correlation.
          The default is 2.

    Raises
    :   **NotImplementedError** – Method fpr correlation not implemented.

    Returns
    :   Output dataframes (Correlation, Annotation strings with significance levels).

    Return type
    :   pd.DataFrame, pd.DataFrame

exmecheva.common.stat\_ext.Dist\_test(*pds*, *alpha=0.05*, *mcomp='Shapiro'*, *mkws={}*, *skipna=True*, *add\_out=False*)[source]
:   Distribution test of data to Hypothesis sample looks Gaussian (reject, if
    p<=alpha).

    Parameters
    :   - **pds** (*pd.Series*) – Series of data.
        - **alpha** (*float**,* *optional*) – Test criterion to reject zero hypothesis (normal distribution).
          The default is 0.05.
        - **mcomp** (*str**,* *optional*) – Test method. The default is ‘Shapiro’.
        - **mkws** (*dict**,* *optional*) – Keyword arguments for used test. The default is {}.
        - **skipna** (*bool**,* *optional*) – Switch for skipping NaNs in data. The default is True.
        - **add\_out** (*bool* *or* *str**,* *optional*) – Switch for output. The default is False.

    Raises
    :   **NotImplementedError** – Method not implemented.

    Returns
    :   Output of test results, depending on add\_out.

    Return type
    :   str, Dict, Series, [Series, Series, str]

exmecheva.common.stat\_ext.Dist\_test\_multi(*pdo*, *axis=0*, *alpha=0.05*, *mcomps=['Shapiro', 'DAgostino']*, *mkws={}*, *skipna=True*, *add\_out='DF'*)[source]
:   Performs multiple distribution tests.

exmecheva.common.stat\_ext.Hypo\_test(*df*, *groupby*, *ano\_Var*, *group\_str=None*, *ano\_str=None*, *alpha=0.05*, *group\_ren={}*, *mcomp='TukeyHSD'*, *mkws={}*, *rel=False*, *rel\_keys=[]*, *deal\_dupl\_ind='raise'*, *group\_ord=None*, *add\_T\_ind=3*, *add\_out=False*)[source]
:   Performs hypothesis test of given data according to selected method.
    Returns results acc. to selected output switch.

    Parameters
    :   - **df** (*pd.DataFrame*) – Dataframe with groups and values as columns for statistical test.
        - **groupby** (*str*) – Name of groups for statistical test.
        - **ano\_Var** (*str*) – Name of variable for statistical test.
        - **group\_str** (*str**,* *optional*) – String for output implementation of group names. The default is None.
        - **ano\_str** (*str**,* *optional*) – String for output implementation of value names. The default is None.
        - **alpha** (*float**,* *optional*) – Test criterion to reject zero hypothesis (all means are equal).
          The default is 0.05.
        - **group\_ren** (*dict**,* *optional*) – Renaming of groups in df. The default is {}.
        - **mcomp** (*TYPE**,* *optional*) –

          Method for hypothesis test. The default is ‘TukeyHSD’.
          Implemented are:

          > - ’TukeyHSD’: Tukey HSD test (see scipy.stats.tukey\_hsd)
          > - ’ttest\_ind’: Independend t-test (see scipy.stats.ttest\_ind)
          > - ’ttest\_rel’: Related t-test (see scipy.stats.ttest\_rel)
          > - ’mannwhitneyu’: Mann-Whittney-U-test (see scipy.stats.mannwhitneyu)
          > - ’wilcoxon’: Wilcoxon signed rank test (see scipy.stats.wilcoxon)
        - **mkws** (*dict**,* *optional*) – Keyword arguments for used hypothesis test.
          p.e.: {‘equal\_var’: False, ‘nan\_policy’: ‘omit’}
          The default is {}.
        - **rel** (*bool**,* *optional*) – Switch for relation of samples (True= related). The default is False.
        - **rel\_keys** (*list**,* *optional*) – Variabel/column names for determining relation. The default is [].
        - **deal\_dupl\_ind** (*string**,* *optional*) – Behavior with duplicated indices (see deal\_dupl\_index).
          The default is ‘raise’.
        - **group\_ord** (*None* *or* *list**,* *optional*) – Selected groups. None will use all columns. The default is None.
        - **add\_T\_ind** (*int**,* *optional*) – Additional indentation of level in textual output. The default is 3.
        - **add\_out** (*TYPE**,* *optional*) –

          Switch for output. The default is False.
          Implemented are:

          > - False: only text output
          > - True: text output (str) and test result (statistic, pvalue as float)
          > - ’Series’: pd.Series of degrees of freedom, statistics, p-value
          >
          > and zero hypothesis test result to alpha
          > - ‘Test’: complete output

    Raises
    :   - **ValueError** – More than two groups identified.
        - **NotImplementedError** – Method not implemented.

    Returns
    :   Output of hypothesis test acc. to selected switch(see add\_out).

    Return type
    :   str or (str, float) or pd.Series or (pd.DataFrame, pd.Series, string)

exmecheva.common.stat\_ext.Hypo\_test\_multi(*df*, *group\_main='Series'*, *group\_sub=['A']*, *ano\_Var=['WC\_vol']*, *mcomp='mannwhitneyu'*, *alpha=0.05*, *mkws={}*, *rel=False*, *rel\_keys=[]*, *deal\_dupl\_ind='raise'*, *group\_ord=None*, *Transpose=True*)[source]
:   Performs hypothesis tests for given variable, in respect to given groups
    according to selected method.
    Returns results acc. to selected output switch.

    Parameters
    :   - **df** (*pd.DataFrame*) – Dataframe with groups and values as columns for statistical test.
        - **group\_main** (*str*) – Main group for statistical test. The default is ‘Series’.
        - **group\_sub** (*list* *of* *str*) – Sub-groups for statistical test (p.e. variants). The default is [‘A’].
        - **ano\_Var** (*list* *of* *str*) – Names of variables for statistical test. The default is [‘WC\_vol’].
        - **mcomp** (*TYPE**,* *optional*) –

          Method for hypothesis test. The default is ‘TukeyHSD’.
          Implemented are:

          > - ’TukeyHSD’: Tukey HSD test (see scipy.stats.tukey\_hsd)
          > - ’ttest\_ind’: Independend t-test (see scipy.stats.ttest\_ind)
          > - ’ttest\_rel’: Related t-test (see scipy.stats.ttest\_rel)
          > - ’mannwhitneyu’: Mann-Whittney-U-test (see scipy.stats.mannwhitneyu)
          > - ’wilcoxon’: Wilcoxon signed rank test (see scipy.stats.wilcoxon)
        - **alpha** (*float**,* *optional*) – Test criterion to reject zero hypothesis (all means are equal).
          The default is 0.05.
        - **mkws** (*dict**,* *optional*) – Keyword arguments for used hypothesis test.
          p.e.: {‘equal\_var’: False, ‘nan\_policy’: ‘omit’}
          The default is {}.
        - **rel** (*bool**,* *optional*) – Switch for relation of samples (True= related). The default is False.
        - **rel\_keys** (*list**,* *optional*) – Variabel/column names for determining relation. The default is [].
        - **deal\_dupl\_ind** (*string**,* *optional*) – Behavior with duplicated indices (see deal\_dupl\_index).
          The default is ‘raise’.
        - **group\_ord** (*None* *or* *list**,* *optional*) – Changing of order of groups not implemented. The default is None.
        - **Transpose** (*bool**,* *optional*) – Switch for transposing result. The default is False.

    Returns
    :   **df\_out** – Dataframe of test results indexed variable name and group.

    Return type
    :   pd.DataFrame

exmecheva.common.stat\_ext.MComp\_interpreter(*T\_Result*)[source]
:   Interprets a test result from statsmodels multicomparision (Zero hypothesis
    reject). Returns identified higher order groups in relation to original
    groups.

    Parameters
    :   **T\_Result** (*statsmodels.iolib.table.SimpleTable*) – Simple table result from statsmodels.

    Returns
    :   - **dict2** (*dict*) – Dictionary of results.
        - **txt** (*str*) – Textual output.

exmecheva.common.stat\_ext.Multi\_conc(*df*, *group\_main='Donor'*, *anat='VA'*, *met='Kruskal'*, *alpha=0.05*, *stdict={'DEFlutoB': ['C', 'G', 'L'], 'DHAntoB': ['C', 'G', 'L'], 'Hyst\_An': ['B'], 'WC\_vol': ['A', 'B', 'L'], 'WC\_vol\_rDA': ['B', 'C', 'L'], 'lu\_F\_mean': ['B']}*, *rel=False*, *rel\_keys=[]*, *kws={}*)[source]
:   Multiple conclusion of statistical tests (variance analysis, hyphotesis test or critical differences)

    Parameters
    :   - **df** (*pd.DataFrame*) – Dataframe.
        - **group\_main** (*str**,* *optional*) – Main group name. The default is ‘Donor’.
        - **anat** (*str**,* *optional*) –

          Type of test.
          Implemented are:

          > - VA: variance analysis
          > - VAwoSg: variance analysis without subgrouping
          > - HT: hyphotesis test
          > - CD: critical differences

          The default is ‘VA’.
        - **met** (*str**,* *optional*) – Methode of test. The default is ‘Kruskal’.
        - **alpha** (*float**,* *optional*) – Test criterion to reject zero hypothesis.
          The default is 0.05.
        - **stdict** (*dict**,* *optional*) –

          Dictionary of value and list of variants to evaluate.
          The default is {‘WC\_vol’:[‘A’,’B’,’L’],’WC\_vol\_rDA’:[‘B’,’C’,’L’],

          > ’lu\_F\_mean’:[‘B’],’DEFlutoB’:[‘C’,’G’,’L’],
          > ‘Hyst\_An’:[‘B’],’DHAntoB’:[‘C’,’G’,’L’]}.
        - **rel** (*bool**,* *optional*) – Switch for relation of samples (True= related). The default is False.
        - **rel\_keys** (*list**,* *optional*) – Variabel/column names for determining relation. The default is [].
        - **kws** (*dict**,* *optional*) – Dictionarie of additional Keyword arguments for selected test.
          The default is {}.

    Returns
    :   **out** – Dataframe of results.

    Return type
    :   pd.DataFrame

exmecheva.common.stat\_ext.NaN\_stat\_outliers(*df*, *numeric\_only=True*, *option='IQR'*, *span=1.5*, *out='all'*, *outsort=None*)[source]
:   Determine statistical outliers with respect to NaN values. Optional selects
    numerical data only.

    Parameters
    :   - **df** (*pd.DataFrame*) – Input data.
        - **numeric\_only** (*bool**,* *optional*) – Numerical columns only. The default is True.
        - **option** (*str**,* *optional*) – Determination option. Only ‘IQR’: interquartile-range-rule implemented.
          The default is ‘IQR’.
        - **span** (*float**,* *optional*) – Span for inclusion (p.e.: ‘IQr’ and 1.5 leads to standard
          1.5-interquartile-range-rule). The default is 1.5.
        - **out** (*str**,* *optional*) –

          Output option. Implemented are:
          :   - ’all’: all data points outside range (all statistical outliers)
              - ’lower’: data points lower then range
              - ’higher’: data points higher then range
              - ’inner’: data points inside range (exclusion of statistical outliers)

          The default is ‘all’.
        - **outsort** (*str**,* *optional*) – Sorting of output. Implemented are ‘ascending’ and ‘descending’.
          All other values will return data unsorted. The default is None.

    Returns
    :   **dfout** – Statistical outlier data acc. to options.

    Return type
    :   pd.DataFrame

exmecheva.common.stat\_ext.agg\_add\_ci(*pdo*, *agg\_funcs=['mean', 'std', 'min', 'max']*)[source]
:   Adds confidence interval to pandas aggregate function

exmecheva.common.stat\_ext.coefficient\_of\_variation(*data*, *outsort=None*, *optmeanabs=True*)[source]
:   Determine coefficient of determination (standard deviation to mean value).

    Parameters
    :   - **data** (*pd.Series* *or* *pd.DataFrame*) – Input data.
        - **outsort** (*str**,* *optional*) – Sorting of output. Implemented are None ‘ascending’ and ‘descending’.
          The default is None.
        - **optmeanabs** (*bool**,* *optional*) – Option for using absolute mean value (if False and mean is negative,
          coefficient of variation will be negative).
          The default is True.

    Returns
    :   **dout** – Coefficient of variation value/-s of input data.

    Return type
    :   float or pd.Series

exmecheva.common.stat\_ext.coefficient\_of\_variation\_woso(*data*, *option='IQR'*, *span=1.5*, *out='inner'*, *outsort=None*, *optmeanabs=True*)[source]
:   Determine coefficient of variation of input data without statistical
    outliers.(see stat\_outliers for more information)

    Parameters
    :   - **data** (*pd.Series* *or* *pd.DataFrame*) – Input data.
        - **option** (*str**,* *optional*) – Determination option. Only ‘IQR’: interquartile-range-rule implemented.
          The default is ‘IQR’.
        - **span** (*float**,* *optional*) – Span for inclusion (p.e.: ‘IQr’ and 1.5 leads to standard
          1.5-interquartile-range-rule). The default is 1.5.
        - **out** (*str**,* *optional*) –

          Output option. Only option inner makes sense.
          Implemented are:

          > - ’all’: all data points outside range (all statistical outliers)
          > - ’lower’: data points lower then range
          > - ’higher’: data points higher then range
          > - ’inner’: data points inside range (exclusion of statistical outliers)

          The default is ‘inner’.
        - **outsort** (*str**,* *optional*) – Sorting of output. Implemented are ‘ascending’ and ‘descending’.
          All other values will return data unsorted. The default is None.
        - **optmeanabs** (*bool**,* *optional*) – Option for using absolute mean value (if False and mean is negative,
          coefficient of variation will be negative).
          The default is True.

    Returns
    :   **dout** – Coefficient of variation value/-s of input data without statistical
        outliers.

    Return type
    :   float or pd.Series

exmecheva.common.stat\_ext.confidence\_interval(*data*, *confidence=0.95*, *method='Seaborn\_Bootstrap'*, *func=<function nanmean>*, *n\_boot=1000*, *axis=None*, *units=None*, *seed=0*, *outtype='List'*)[source]
:   Calculates the confidence interval of given data.

    Parameters
    :   - **data** (*pandas.Series* *of* *float*) – Series of values to be analysed.
        - **confidence** (*float**,* *optional*) – Confidence value. The default is 0.95.
        - **method** (*string**,* *optional*) –

          Method to use.
          Implemented are:

          > - Seaborn\_Bootstrap: Seaborn Bootstrapping (Compare Seaborn barplot)
          > - Wald: Wald-Confidence-Interval

          The default is “Seaborn\_Bootstrap”.
        - **func** (*string* *or* *callable**,* *optional*) – DESCRIPTION. The default is “nanmean”.
        - **n\_boot** (*int**,* *optional*) – Number of iterations. The default is 1000.
        - **axis** (*int**,* *optional*) – Applied axis. The default is None.
        - **units** (*array**,* *optional*) – Sampling units (see seaborn.algorithms). The default is None.
        - **seed** (*Generator* *|* *SeedSequence* *|* *RandomState* *|* *int* *|* *None**,* *optional*) – Seed for rondom number generator. The default is 0.
        - **outtype** (*str**,* *optional*) – Switch for output (List, Dict, Min, Max). The default is List.

    Raises
    :   **NotImplementedError** – Error to raise if option not implemented.

    Returns
    :   **CI** – Confidence interval values [lower, higher].

    Return type
    :   list or or dict or value

exmecheva.common.stat\_ext.cv(*data*, *outsort=None*, *optmeanabs=True*)[source]
:   Short naming of funciton coefficient\_of\_variation.
    Readability improved by usage of pandas aggregate function (pd.agg).

exmecheva.common.stat\_ext.cvwoso(*data*, *option='IQR'*, *span=1.5*, *out='inner'*, *outsort=None*, *optmeanabs=True*)[source]
:   Short naming of funciton coefficient\_of\_variation\_woso.
    Readability improved by usage of pandas aggregate function (pd.agg).

exmecheva.common.stat\_ext.group\_ANOVA\_MComp(*df*, *groupby*, *ano\_Var*, *group\_str=None*, *ano\_str=None*, *mpop='ANOVA'*, *alpha=0.05*, *group\_ren={}*, *do\_mcomp\_a=1*, *mcomp='TukeyHSD'*, *mpadj='bonf'*, *Ffwalpha=2*, *mkws={}*, *nan\_policy='omit'*, *check\_resnorm=False*, *add\_T\_ind=3*, *add\_out=False*)[source]
:   Performs an one way variance analysis and multi comparision test for given
    variable, in respect to given groups.
    Returns an output string with summary and optional additional test outputs.

    Parameters
    :   - **df** (*pd.DataFrame*) – Dataframe with groups and values as columns for statistical test.
        - **groupby** (*str*) – Name of groups for statistical test.
        - **ano\_Var** (*str*) – Name of variable for statistical test.
        - **group\_str** (*str**,* *optional*) – String for output implementation of group names. The default is None.
        - **ano\_str** (*str**,* *optional*) – String for output implementation of value names. The default is None.
        - **mpop** (*str**,* *optional*) – Method for population test.
          Implemented are ANOVA and Kruskal-Wallis H-test.
          The default is ANOVA.
        - **alpha** (*float**,* *optional*) – Test criterion to reject zero hypothesis (all means are equal).
          The default is 0.05.
        - **group\_ren** (*dict**,* *optional*) – Renaming of groups in df. The default is {}.
        - **do\_mcomp\_a** (*int**,* *optional*) – Performance level of tukey test
          (0 - never, 1 - only if p of ANOVA lower alpha, 2 - allways).
          The default is 1.
        - **mcomp** (*str**,* *optional*) – Method for multi comparison.
          Implemented are TukeyHSD, ttest\_ind, ttest\_rel, mannwhitneyu.
          The default is TukeyHSD.
        - **mpadj** (*str**,* *optional*) – Method for testing and adjustment of pvalues.
          For further information see: statsmodels.stats.multitest.multipletests
          The default is bonf.
        - **Ffwalpha** (*float**,* *optional*) – Factor for family-wise error in comparision to alpha.
          The default is 2.
        - **mkws** (*dict**,* *optional*) – Keyword arguments for used multi comparision test.
          p.e.: {‘equal\_var’: False, ‘nan\_policy’: ‘omit’}
          The default is {}.
        - **nan\_policy** (*str**,* *optional*) – Handling of NaN values. The default is ‘omit’.
        - **add\_T\_ind** (*int**,* *optional*) – Additional indentation of lines in txt for Tukey HSD. The default is 3.
        - **add\_out** (*bool**,* *optional*) – Additional output request. The default is False.

    Returns
    :   - **txt** (*str*) – Text output with ANOVA and optional Tukey-HSD results.
        - **[F,p]** (*[float,float], optional*) – ANOVA results
        - **t** (*statsmodels.iolib.table.SimpleTable, optional*) – Results of multi comaprison test.

exmecheva.common.stat\_ext.group\_ANOVA\_MComp\_multi(*df*, *group\_main='Series'*, *group\_sub=['A']*, *ano\_Var=['WC\_vol']*, *mpop='ANOVA'*, *alpha=0.05*, *group\_ren={}*, *do\_mcomp\_a=0*, *mcomp='TukeyHSD'*, *mpadj='bonf'*, *Ffwalpha=1*, *mkws={}*, *check\_resnorm=False*, *Transpose=True*)[source]
:   Performs an one way ANOVA and multi comparision test for given variable,
    in respect to given groups and sub-groups.
    Returns an output string with summary and optional additional test outputs.

    Parameters
    :   - **df** (*pd.DataFrame*) – Dataframe with groups and values as columns for statistical test.
        - **group\_main** (*str*) – Main group for statistical test.
        - **group\_sub** (*list* *of* *str*) – Sub-groups for statistical test (p.e. variants).
        - **ano\_Var** (*list* *of* *str*) – Names of variables for statistical test.
        - **group\_str** (*str**,* *optional*) – String for output implementation of group names. The default is None.
        - **mpop** (*str**,* *optional*) – Method for population test.
          Implemented are ANOVA and Kruskal-Wallis H-test.
          The default is ANOVA.
        - **alpha** (*float**,* *optional*) – Test criterion to reject zero hypothesis (all means are equal). The default is 0.05.
        - **group\_ren** (*dict**,* *optional*) – Renaming of groups in df. The default is {}.
        - **do\_mcomp\_a** (*int**,* *optional*) – Performance level of tukey test
          (0 - never, 1 - only if p of ANOVA lower alpha, 2 - allways).
          The default is 1.
        - **mcomp** (*str**,* *optional*) – Method for multi comparison.
          Implemented are TukeyHSD, ttest\_ind, ttest\_rel, mannwhitneyu.
          The default is TukeyHSD.
        - **mpadj** (*str**,* *optional*) – Method for testing and adjustment of pvalues.
          For further information see: statsmodels.stats.multitest.multipletests
          The default is bonf.
        - **Ffwalpha** (*float**,* *optional*) – Factor for family-wise error in comparision to alpha.
          The default is 2.
        - **mkws** (*dict**,* *optional*) – Keyword arguments for used multi comparision test.
          p.e.: {‘equal\_var’: False, ‘nan\_policy’: ‘omit’}
          The default is {}.
        - **Transpose** (*bool**,* *optional*) – Switch for transposing result. The default is False.

    Returns
    :   **df\_out** – DataFrame output with variance analyses and additional results.

    Return type
    :   pd.DataFrame

exmecheva.common.stat\_ext.group\_Anova(*df*, *groupby*, *ano\_Var*, *group\_str=None*, *ano\_str=None*, *alpha=0.05*)[source]
:   Performs an one way ANOVA -> depricated (use group\_ANOVA\_MComp)

exmecheva.common.stat\_ext.meanwoso(*data*, *option='IQR'*, *span=1.5*, *out='inner'*, *outsort=None*)[source]
:   Determine mean value of input data without statistical outliers.
    (see stat\_outliers for more information)

    Parameters
    :   - **data** (*pd.Series* *or* *pd.DataFrame*) – Input data.
        - **option** (*str**,* *optional*) – Determination option. Only ‘IQR’: interquartile-range-rule implemented.
          The default is ‘IQR’.
        - **span** (*float**,* *optional*) – Span for inclusion (p.e.: ‘IQr’ and 1.5 leads to standard
          1.5-interquartile-range-rule). The default is 1.5.
        - **out** (*str**,* *optional*) –

          Output option. Only option inner makes sense.
          Implemented are:

          > - ’all’: all data points outside range (all statistical outliers)
          > - ’lower’: data points lower then range
          > - ’higher’: data points higher then range
          > - ’inner’: data points inside range (exclusion of statistical outliers)

          The default is ‘inner’.
        - **outsort** (*str**,* *optional*) – Sorting of output. Implemented are ‘ascending’ and ‘descending’.
          All other values will return data unsorted. The default is None.

    Returns
    :   **dout** – Mean value/-s of input data without statistical outliers.

    Return type
    :   float or pd.Series

exmecheva.common.stat\_ext.pd\_agg(*pd\_o*, *agg\_funcs=['mean', 'median', 'std', 'max', 'min']*, *numeric\_only=False*)[source]
:   Aggregate pandas object with defined functions.

exmecheva.common.stat\_ext.pd\_agg\_custom(*pdo, agg\_funcs=['mean', <function meanwoso>, 'median', 'std', <function coefficient\_of\_variation>, <function stdwoso>, <function coefficient\_of\_variation\_woso>, 'min', 'max', <function confidence\_interval>], numeric\_only=False, af\_ren={'coefficient\_of\_variation': 'CV', 'coefficient\_of\_variation\_woso': 'CVwoso'}, af\_unp={'confidence\_interval': ['CImin', 'CImax']}*)[source]
:   Aggregate pandas object with defined functions, including unpacked
    multi-value functions.

    Parameters
    :   - **pdo** (*pd.DataFrame* *or* *pd.Series*) – Pandas object (DataFrame or Series).
        - **agg\_funcs** (*list* *of* *functions**,* *optional*) –

          List of aggregatable functions (pandas aggregate accepted).
          The default is [‘mean’,meanwoso,’median’,

          > ’std’,coefficient\_of\_variation,stdwoso,
          > coefficient\_of\_variation\_woso,
          > ‘min’,’max’,confidence\_interval].
        - **numeric\_only** (*bool**,* *optional*) – Switch for consideration of numerical values only (int and float). The default is False.
        - **af\_ren** (*dict**,* *optional*) –

          Dictionary for renaming of aggregate function names.
          The default is {‘coefficient\_of\_variation\_woso’:’CVwoso’,

          > ’coefficient\_of\_variation’:’CV’}.
        - **af\_unp** (*dict**,* *optional*) – Dictionary for unpacking of aggregate function.
          The default is {‘confidence\_interval’: [‘CImin’,’CImax’]}.

    Returns
    :   Aggregation values depending on given functions.

    Return type
    :   pd.DataFrame or pd.Series

exmecheva.common.stat\_ext.reg\_stats\_multi(*df*, *lRd*, *var\_ren={}*, *var\_sym={}*, *ftype='linear'*, *guess={'a': 0.01, 'b': 0.1}*, *t\_form='{a:.3e},{b:.3e}'*, *max\_nfev=1000*, *nan\_policy='omit'*, *ind=3*, *addind=3*)[source]
:   Performs multiple regression fits (according fitting.regfitret) on given
    data. Returns a DataFrame and textual output.

    Parameters
    :   - **df** (*pd.DataFrame*) – Input data.
        - **lRd** (*list*) – List of compared variables (must be column names in df).
          Example [[‘Density\_app’,’Age’]]
        - **var\_ren** (*dict**,* *optional*) – Renamer for given variables (description).
          Example dict(‘Density\_app’=’apparent density’,’Age’=’donor age’)
          The default is {}.
        - **var\_sym** (*dict**,* *optional*) – Renamer for given variables (symbols).
          Example dict(‘Density\_app’=’rho\_{app}’,’Age’=’age’)
          The default is {}.
        - **ftype** (*str**,* *optional*) –

          Type and name of function to adjust.
          Implemented are:

          > - ’linear’: linear and constant function (see func\_lin)
          > - ’power’: power and constant function (see func\_pow)
          > - ’exponential’: exponantial and constant function (see func\_exp)
          > - additional endings:
          >   :   - ’\_nc’: no constant value
          >       - ’\_x0’: fixed to zero

          The default is ‘linear’.
        - **guess** (*dictionary**,* *optional*) – Dictionary for first guess. The default is dict(a=0.01, b=0.1).
        - **t\_form** (*str**,* *optional*) – String of dictionary with variables and format strings.
          The default is ‘{a:.3e},{b:.3e}’.
        - **max\_nfev** (*int**,* *optional*) – Maximum number of evaluations. The default is 1000.
        - **nan\_policy** (*string**,* *optional*) – NaN policy (omit, raise or propagate). The default is ‘omit’.
        - **ind** (*TYPE**,* *optional*) – Indentation for text output. The default is 3.
        - **addind** (*int**,* *optional*) – Additional indentation for text output. The default is 3.

    Returns
    :   - **reg\_df** (*pd.Dataframe*) – Results of regressions.
        - **txt** (*str*) – Textual output.

exmecheva.common.stat\_ext.relative\_deviation(*a*, *b*, *axis=0*)[source]
:   Calculates the relative deviation from second argument to first.

    \[RD\_{b-a} = (b-a)/a\]

    Parameters
    :   - **a** (*int* *or* *float* *or* *pd.Series* *or* *pd.DataFrame*) – Base value.
        - **b** (*pd.Series* *or* *pd.DataFrame*) – Calculation value.
        - **axis** (*int**,* *optional*) – Identifyier for performing calculation. The default is 0.

    Raises
    :   **NotImplementedError** – Combination of types not implemented.

    Returns
    :   **out** – Relative deviation result (type depends on input types).

    Return type
    :   int or float or pd.Series or pd.DataFrame

exmecheva.common.stat\_ext.stat\_box\_vals(*data*, *option='IQR'*, *span=1.5*)[source]
:   Determine values of typical boxplot values (1st quantile, 3rd qhuantile)

    datapd.Series or pd.DataFrame
    :   Input data.

    optionstr, optional
    :   Determination option. Only ‘IQR’: interquartile-range-rule implemented.
        The default is ‘IQR’.

    spanfloat, optional
    :   Span for inclusion (p.e.: ‘IQr’ and 1.5 leads to standard
        1.5-interquartile-range-rule). The default is 1.5.

    Raises
    :   **NotImplementedError** – Option not implemented.

    Returns
    :   - **box\_vals** (*dict*) – DESCRIPTION.
          - ‘lBo’: lower boarder of 1.5-IQR
          - ‘minin’: minimal value inside range
          - ‘dQ1’: first quartile
          - ‘med’: Median
          - ‘dQ3’: third quartile
          - ‘maxin’: maximal value inside range
          - ‘uBo’: upper boarder of 1.5-IQR
        - **inner\_vals** (*pd.Series or pd.DataFrame (like input data)*) – Index of values inside range (not statistical outlier).
        - **outer\_vals** (*pd.Series or pd.DataFrame (like input data)*) – Index of values outside range (statistical outlier).

exmecheva.common.stat\_ext.stat\_outliers(*data*, *option='IQR'*, *span=1.5*, *out='all'*, *outsort='ascending'*)[source]
:   Determine statistical outliers.

    Parameters
    :   - **data** (*pd.Series* *or* *pd.DataFrame*) – Input data.
        - **option** (*str**,* *optional*) – Determination option. Only ‘IQR’: interquartile-range-rule implemented.
          The default is ‘IQR’.
        - **span** (*float**,* *optional*) – Span for inclusion (p.e.: ‘IQr’ and 1.5 leads to standard
          1.5-interquartile-range-rule). The default is 1.5.
        - **out** (*str**,* *optional*) –

          Output option. Implemented are:
          :   - ’all’: all data points outside range (all statistical outliers)
              - ’lower’: data points lower then range
              - ’higher’: data points higher then range
              - ’inner’: data points inside range (exclusion of statistical outliers)

          The default is ‘all’.
        - **outsort** (*str**,* *optional*) – Sorting of output. Implemented are ‘ascending’ and ‘descending’.
          All other values will return data unsorted. The default is ‘ascending’.

    Raises
    :   **NotImplementedError** – Option not implemented.

    Returns
    :   **stol** – Statistical outlier data acc. to options.

    Return type
    :   pd.Series or pd.DataFrame (like input data)

exmecheva.common.stat\_ext.stdwoso(*data*, *option='IQR'*, *span=1.5*, *out='inner'*, *outsort=None*)[source]
:   Determine standard deviation of input data without statistical outliers.
    (see stat\_outliers for more information)

    Parameters
    :   - **data** (*pd.Series* *or* *pd.DataFrame*) – Input data.
        - **option** (*str**,* *optional*) – Determination option. Only ‘IQR’: interquartile-range-rule implemented.
          The default is ‘IQR’.
        - **span** (*float**,* *optional*) – Span for inclusion (p.e.: ‘IQr’ and 1.5 leads to standard
          1.5-interquartile-range-rule). The default is 1.5.
        - **out** (*str**,* *optional*) –

          Output option. Only option inner makes sense.
          Implemented are:

          > - ’all’: all data points outside range (all statistical outliers)
          > - ’lower’: data points lower then range
          > - ’higher’: data points higher then range
          > - ’inner’: data points inside range (exclusion of statistical outliers)

          The default is ‘inner’.
        - **outsort** (*str**,* *optional*) – Sorting of output. Implemented are ‘ascending’ and ‘descending’.
          All other values will return data unsorted. The default is None.

    Returns
    :   **dout** – Standard deviation value/-s of input data without statistical outliers.

    Return type
    :   float or pd.Series

## Module contents

Common functionality for exmecheva.

Previous

---

© Copyright 2024, MarcGebhardt.

Built with Sphinx using a
theme
provided by Read the Docs.
