# Supplementary material for: Approach to Standardized Material Characterization of the Human Lumbopelvic System: Testing and Evaluation
Source: Bioengineering (Basel). 2025 Aug 11;12(8):862. doi: 10.3390/bioengineering12080862 (PMC12383908; doi:10.3390/bioengineering12080862)
Supplement: Supplementary file 1 [file bioengineering-12-00862-s001.zip › File S3 Evaluation code/ExMechEva-0.1.2/docs/_build/html/exmecheva.html]

exmecheva package — ExMechEva v0.1.2 documentation


ExMechEva

Contents:

- ExMechEva
  - exmecheva package
    - Subpackages
      - exmecheva.bending package
      - exmecheva.common package
    - Submodules
    - exmecheva.Eva\_ACT module
      - `ACT_single()`
    - exmecheva.Eva\_ATT module
      - `ATT_single()`
    - exmecheva.Eva\_TBT module
      - `TBT_single()`
    - exmecheva.eva module
      - `selector()`
      - `series()`
    - Module contents

ExMechEva

- ExMechEva
- exmecheva package
- View page source

---

# exmecheva package

## Subpackages

- exmecheva.bending package
  - Submodules
  - exmecheva.bending.attr\_bgl module
  - exmecheva.bending.bfunc\_class module
    - `Bend_func_cohort`
      - `Bend_func_cohort.Init_fandds()`
    - `Bend_func_legion`
      - `Bend_func_legion.Builder()`
    - `Bend_func_sub`
      - `Bend_func_sub.__call__()`
  - exmecheva.bending.bfunc\_com module
    - `Shear_area()`
    - `gamma_V_det()`
    - `triangle_func_d0()`
    - `triangle_func_d1()`
    - `triangle_func_d2()`
  - exmecheva.bending.bfunc\_fse module
    - `FSE_4sin_d0()`
    - `FSE_4sin_d1()`
    - `FSE_4sin_d2()`
    - `FSE_4sin_lin_func_d0()`
    - `FSE_4sin_lin_func_d1()`
    - `FSE_4sin_lin_func_d2()`
    - `FSE_4sin_wlin_d0()`
    - `FSE_4sin_wlin_d1()`
    - `FSE_4sin_wlin_d2()`
    - `FSE_SF_func_d0()`
    - `FSE_SF_func_d1()`
    - `FSE_SF_func_d2()`
  - exmecheva.bending.evaluation module
    - `Moment_perF_func()`
    - `Weight_func()`
    - `YM_check_many_with_method_D()`
    - `YM_check_with_method_D()`
    - `YM_eva_method_A()`
    - `YM_eva_method_B()`
    - `YM_eva_method_C()`
    - `YM_eva_method_D()`
    - `YM_eva_method_D_bend_df()`
    - `YM_eva_method_D_bend_df_add()`
    - `YM_eva_method_D_num()`
    - `YM_eva_method_D_res()`
    - `YM_eva_method_E()`
    - `YM_eva_method_F()`
    - `YM_eva_method_G()`
    - `coord_df_depo()`
    - `coord_df_mean()`
    - `straindf_from_curve()`
    - `stress_df_from_lin()`
    - `stress_perF()`
  - exmecheva.bending.fitting module
    - `Multi_minimize()`
    - `Perform_Fit()`
    - `lmfit_bound_checker()`
    - `lmfit_free_val_setter()`
    - `lmfit_modelize()`
    - `lmfit_param_adder()`
    - `lmfit_param_key_checker()`
    - `lmfit_param_prep()`
    - `res_multi_const_weighted()`
    - `shaped_array_fill_fandl()`
  - exmecheva.bending.opt\_mps module
    - `Point_df_combine()`
    - `Point_df_from_lin()`
    - `Point_df_idx()`
    - `Point_df_transform()`
    - `Points_add_step()`
    - `Points_dif_step()`
    - `Points_diff()`
    - `Points_eval_func()`
    - `v_Ctrans()`
    - `v_length()`
  - exmecheva.bending.plotting module
    - `colplt_common_ax()`
    - `colplt_df_ax()`
    - `colplt_funcs_all()`
    - `colplt_funcs_ax()`
    - `colplt_funcs_one()`
  - Module contents
- exmecheva.common package
  - Submodules
  - exmecheva.common.analyze module
    - `Geo_curve_TBC()`
    - `Inter_Lines()`
    - `Line_from2P()`
    - `TP_circle()`
    - `TP_radius()`
    - `normalize()`
    - `normalize_th()`
    - `sign_n_change()`
    - `sign_n_changeth()`
    - `threshhold_setter()`
  - exmecheva.common.eva\_opt\_hand module
    - `com_option_file_read()`
    - `com_option_file_write()`
    - `option_presetter()`
    - `option_reader()`
    - `option_reader_sel()`
    - `set_type_by_string()`
  - exmecheva.common.fitting module
    - `Refit_YM_vals()`
    - `Rquad()`
    - `YM_eva_com_sel()`
    - `YM_sigeps_lin()`
    - `fit_report_adder()`
    - `func_exp()`
    - `func_exp_str()`
    - `func_lin()`
    - `func_lin_str()`
    - `func_pow()`
    - `func_pow_str()`
    - `regfitret()`
    - `regfitret_restring_func()`
    - `strain_linfit()`
    - `stress_linfit()`
    - `stress_linfit_plt()`
  - exmecheva.common.helper module
    - `check_empty()`
    - `round_to_sigdig()`
    - `sigdig()`
    - `str_to_bool()`
    - `type_str_return()`
  - exmecheva.common.list\_ops module
    - `Failure_code_bool_df()`
    - `Failure_code_checker()`
    - `Failure_code_format()`
    - `Failure_code_lister()`
    - `ICD_bool_df()`
    - `ICD_lister()`
    - `list_boolean_df()`
    - `list_cell_compiler()`
    - `list_interpreter()`
    - `list_ser_to_1D()`
  - exmecheva.common.loadnsave module
    - `comb_logs()`
    - `file_namer()`
    - `file_namer_interpreter()`
    - `pack_hdf()`
    - `pack_hdf_mul()`
  - exmecheva.common.mc\_char module
    - `Diff_Quot()`
    - `Diff_Quot2()`
    - `Diff_Quot3()`
    - `YM_eva_range_refine()`
    - `curve_characterizer()`
    - `curve_merger()`
    - `curvecar_refine()`
    - `curvecar_section()`
    - `find_SandE()`
    - `peaky_finder()`
    - `peaky_finder_MM()`
    - `poi_det_plh()`
    - `poi_fixeva()`
    - `poi_refinement()`
    - `poi_rel_finder()`
    - `poi_vip_namer()`
    - `rise_curve()`
    - `test_pdmon()`
  - exmecheva.common.mc\_man module
    - `DetFinSSC()`
    - `Diff_ext()`
    - `Extend_Series_Poly()`
    - `Extend_Series_n_setter()`
    - `Predict_apply_retrim()`
    - `Retrim_Series()`
    - `Smoothsel()`
    - `Smoothsel_ext()`
    - `check_params()`
    - `mc_resampler()`
    - `smooth()`
  - exmecheva.common.mc\_yield module
    - `Find_intg2p()`
    - `YM_eva_range_refine()`
    - `Yield_redet()`
    - `Yield_redet2()`
    - `Yield_redet2_Multi()`
  - exmecheva.common.output module
    - `Otvalgetter_Multi()`
    - `Outvalgetter()`
    - `str_indent()`
    - `str_log()`
  - exmecheva.common.pd\_ext module
    - `Find_closest()`
    - `Find_closest_perc()`
    - `Find_closestv()`
    - `Find_first_sc()`
    - `deal_dupl_index()`
    - `pd_axischange()`
    - `pd_combine_index()`
    - `pd_exclnan()`
    - `pd_find_index()`
    - `pd_isDF()`
    - `pd_isSer()`
    - `pd_limit()`
    - `pd_nan_handler()`
    - `pd_outsort()`
    - `pd_slice_index()`
    - `pd_trapz()`
    - `pd_valid_index()`
    - `pd_vec_length()`
  - exmecheva.common.plotting module
    - `curve_char_plotter()`
    - `plt_add_DaAnno()`
    - `plt_ax_regfit()`
    - `plt_handle_suffix()`
    - `sns_pointplot_MMeb()`
    - `tick_label_inserter()`
    - `tick_label_renamer()`
    - `tick_legend_renamer()`
  - exmecheva.common.stat\_ext module
    - `CD_rep()`
    - `CD_test_multi()`
    - `CImax()`
    - `CImin()`
    - `Corr_ext()`
    - `Dist_test()`
    - `Dist_test_multi()`
    - `Hypo_test()`
    - `Hypo_test_multi()`
    - `MComp_interpreter()`
    - `Multi_conc()`
    - `NaN_stat_outliers()`
    - `agg_add_ci()`
    - `coefficient_of_variation()`
    - `coefficient_of_variation_woso()`
    - `confidence_interval()`
    - `cv()`
    - `cvwoso()`
    - `group_ANOVA_MComp()`
    - `group_ANOVA_MComp_multi()`
    - `group_Anova()`
    - `meanwoso()`
    - `pd_agg()`
    - `pd_agg_custom()`
    - `reg_stats_multi()`
    - `relative_deviation()`
    - `stat_box_vals()`
    - `stat_outliers()`
    - `stdwoso()`
  - Module contents

## Submodules

## exmecheva.Eva\_ACT module

Axial compression test evaluation.

@author: MarcGebhardt

exmecheva.Eva\_ACT.ACT\_single(*prot\_ser*, *paths*, *mfile\_add=''*, *log\_scopt={'logfp': None, 'logopt': True, 'output\_lvl': 1, 'printopt': False}*, *plt\_scopt={'clear': True, 'close': True, 's\_types': ['pdf'], 'save': True, 'show': True, 'tight': True}*)[source]
:   Evaluate single axial compression test measurement form protocol table and
    path collection. Using common options wich are overwritten by protocol
    variables starting with ‘OPT\_’. Produce evaluated measurements,
    material parameters and plots and safe them as table (.csv), unstructured
    database (.h5) and document (.pdf), using the designation of the specimen
    for distinction.
    Procedure:

    > - 1: Read in options and presetting
    > - 2: Determining geometrical values
    > - 3: Read in measurements (conventional and optical (optinal))
    > - 4: Merging measurements (time offset of conventional to optical
    >   :   (if available), downsampling and merging)
    > - 5: Determine evaluation space (start and end)
    > - 6: Evaluation (curves (stress/strain), importent points on curves,
    >   :   elastic moduli (different types implemented))
    > - 7: Generating output (tables, database and plots)

    Parameters
    :   - **prot\_ser** (*pd.Series*) –

          Input data as pandas series with specimen information
          (identifier, origin, geometrical data,

          > assessment codes, evaluation options).
        - **paths** (*pd.DataFrame*) –

          Path collection for in- and output paths.
          Needs indexes:

          > - ”opts”: Common evaluation options
          > - ”prot”: Protocol
          > - ”meas”: Conventional measured data
          > - ”dic”: Optical measured data
          > - ”out”: Output
        - **mfile\_add** (*string**,* *optional*) – Suffix of variants of measurements
          (p.E. diffferent moistures [“A”,”B”,…]).
          The default is ‘’.
        - **log\_scopt** (*dict**,* *optional*) –

          Options for custom logging. Determining file path,
          output level (0=none, 1=normal, 2=special), logging enabled and
          printing enabled.
          The default is {‘logfp’:None, ‘output\_lvl’: 1,

          > ’logopt’:True, ‘printopt’:False}.
        - **plt\_scopt** (*dict**,* *optional*) –

          Options for plotting.
          The default is {‘tight’:True, ‘show’:True,

          > ’save’:True, ‘s\_types’:[“pdf”],
          > ‘clear’:True, ‘close’:True}.

    Raises
    :   - **ValueError** – Input value not correct.
        - **NotImplementedError** – Method not implemented.

    Yields
    :   - **timings** (*pd.Series*) – Timings of procedure steps.
        - **cout** (*string*) – Special text output for control purposes.

## exmecheva.Eva\_ATT module

Axial tensile test evaluation.

@author: MarcGebhardt

exmecheva.Eva\_ATT.ATT\_single(*prot\_ser*, *paths*, *mfile\_add=''*, *log\_scopt={'logfp': None, 'logopt': True, 'output\_lvl': 1, 'printopt': False}*, *plt\_scopt={'clear': True, 'close': True, 's\_types': ['pdf'], 'save': True, 'show': True, 'tight': True}*)[source]
:   Evaluate single axial tensile test measurement form protocol table and
    path collection. Using common options wich are overwritten by protocol
    variables starting with ‘OPT\_’. Produce evaluated measurements,
    material parameters and plots and safe them as table (.csv), unstructured
    database (.h5) and document (.pdf), using the designation of the specimen
    for distinction.
    Procedure:

    > - 1: Read in options and presetting
    > - 2: Determining geometrical values
    > - 3: Read in measurements (conventional and optical (optinal))
    > - 4: Merging measurements (time offset of conventional to optical
    >   :   (if available), downsampling and merging)
    > - 5: Determine evaluation space (start and end)
    > - 6: Evaluation (curves (stress/strain), importent points on curves,
    >   :   elastic moduli (different types implemented))
    > - 7: Generating output (tables, database and plots)

    Parameters
    :   - **prot\_ser** (*pd.Series*) –

          Input data as pandas series with specimen information
          (identifier, origin, geometrical data,

          > assessment codes, evaluation options).
        - **paths** (*pd.DataFrame*) –

          Path collection for in- and output paths.
          Needs indexes:

          > - ”opts”: Common evaluation options
          > - ”prot”: Protocol
          > - ”meas”: Conventional measured data
          > - ”dic”: Optical measured data
          > - ”out”: Output
        - **mfile\_add** (*string**,* *optional*) – Suffix of variants of measurements
          (p.E. diffferent moistures [“A”,”B”,…]).
          The default is ‘’.
        - **log\_scopt** (*dict**,* *optional*) –

          Options for custom logging. Determining file path,
          output level (0=none, 1=normal, 2=special), logging enabled and
          printing enabled.
          The default is {‘logfp’:None, ‘output\_lvl’: 1,

          > ’logopt’:True, ‘printopt’:False}.
        - **plt\_scopt** (*dict**,* *optional*) –

          Options for plotting.
          The default is {‘tight’:True, ‘show’:True,

          > ’save’:True, ‘s\_types’:[“pdf”],
          > ‘clear’:True, ‘close’:True}.

    Raises
    :   - **ValueError** – Input value not correct.
        - **NotImplementedError** – Method not implemented.

    Yields
    :   - **timings** (*pd.Series*) – Timings of procedure steps.
        - **cout** (*string*) – Special text output for control purposes.

## exmecheva.Eva\_TBT module

Three point bending test evaluation.

@author: MarcGebhardt

exmecheva.Eva\_TBT.TBT\_single(*prot\_ser*, *paths*, *mfile\_add=''*, *log\_scopt={'logfp': None, 'logopt': True, 'output\_lvl': 1, 'printopt': False}*, *plt\_scopt={'clear': True, 'close': True, 's\_types': ['pdf'], 'save': True, 'show': True, 'tight': True}*)[source]
:   Evaluate single three point bending measurement form protocol table and
    path collection. Using common options wich are overwritten by protocol
    variables starting with ‘OPT\_’. Produce evaluated measurements,
    material parameters and plots and safe them as table (.csv), unstructured
    database (.h5) and document (.pdf), using the designation of the specimen
    for distinction.
    Procedure:

    > - 1: Read in options and presetting
    > - 2: Determining geometrical values
    > - 3: Read in measurements (conventional and optical (optinal))
    > - 4: Merging measurements (time offset of conventional to optical
    >   :   (if available), downsampling and merging)
    > - 5: Determine evaluation space (start and end)
    > - 6: Evaluation (curves (stress/strain), importent points on curves,
    >   :   elastic moduli (different types implemented))
    > - 7: Generating output (tables, database and plots)

    Parameters
    :   - **prot\_ser** (*pd.Series*) –

          Input data as pandas series with specimen information
          (identifier, origin, geometrical data,

          > assessment codes, evaluation options).
        - **paths** (*pd.DataFrame*) –

          Path collection for in- and output paths.
          Needs indexes:

          > - ”opts”: Common evaluation options
          > - ”prot”: Protocol
          > - ”meas”: Conventional measured data
          > - ”dic”: Optical measured data
          > - ”out”: Output
        - **mfile\_add** (*string**,* *optional*) – Suffix of variants of measurements
          (p.E. diffferent moistures [“A”,”B”,…]).
          The default is ‘’.
        - **log\_scopt** (*dict**,* *optional*) –

          Options for custom logging. Determining file path,
          output level (0=none, 1=normal, 2=special), logging enabled and
          printing enabled.
          The default is {‘logfp’:None, ‘output\_lvl’: 1,

          > ’logopt’:True, ‘printopt’:False}.
        - **plt\_scopt** (*dict**,* *optional*) –

          Options for plotting.
          The default is {‘tight’:True, ‘show’:True,

          > ’save’:True, ‘s\_types’:[“pdf”],
          > ‘clear’:True, ‘close’:True}.

    Raises
    :   - **ValueError** – Input value not correct.
        - **NotImplementedError** – Method not implemented.

    Yields
    :   - **timings** (*pd.Series*) – Timings of procedure steps.
        - **cout** (*string*) – Special text output for control purposes.

## exmecheva.eva module

Functionality for selection of evaluation (single, series or complete) and
packing of HDF-file.

@author: MarcGebhardt
.. todo:: - series only work with output\_lvl>=1

exmecheva.eva.selector(*eva\_single\_func*, *option*, *combpaths*, *no\_stats\_fc*, *var\_suffix=['']*, *ser=''*, *des=''*, *out\_path=''*, *prot\_rkws={'header': 11, 'index\_col': 0, 'skiprows': range(12, 13)}*, *log\_scopt={'logfp': None, 'logopt': True, 'output\_lvl': 1, 'printopt': False}*, *plt\_scopt={'clear': True, 'close': True, 's\_types': ['pdf'], 'save': True, 'show': True, 'tight': True}*)[source]
:   Selects suitable evaluation method acc. to choosen option.

    Parameters
    :   - **eva\_single\_func** (*function*) – Function to perform single evaluation.
        - **option** (*str*) –

          Evaluation option. Possible are:
          :   - ’single’: Evaluate single measurement
              - ’series’: Evaluate series of measurements (see protocol table)
              - ’complete’: Evaluate series of series
              - ’pack’: Pack all evaluations into single hdf-file (only results and evaluated measurement)
              - ’pack-all’: Pack all evaluations into single hdf-file with (all results, Warning: high memory requirements!)
        - **combpaths** (*pandas.DataFrame*) – Combined paths for in- and output of evaluations.
        - **no\_stats\_fc** (*list* *of* *str*) – Assessment codes for excluding from evaluation (searched in protocol variable “Failure\_code”).
        - **var\_suffix** (*list* *of* *str**,* *optional*) – Suffix of variants of measurements (p.E. different moistures [“A”,”B”,…]).
          The default is [“”].
        - **ser** (*str**,* *optional*) – Accessor for series. Must be as index in combpaths. The default is ‘’.
        - **des** (*str**,* *optional*) – Accessor for/Designation of measurement/specimen.
          Must be as index in combpaths. The default is ‘’.
        - **out\_path** (*str* *or* *Path**,* *optional*) – Additional outputpath for packed evaluation (hdf file). The default is ‘’.
        - **prot\_rkws** (*dict**,* *optional*) – Dictionary for reading protocol. Must be keyword ind pandas.read\_excel.
          The default is dict(header=11, skiprows=range(12,13),index\_col=0).

    Raises
    :   **NotImplementedError** – Option not implemented.

    Return type
    :   None.

exmecheva.eva.series(*eva\_single\_func*, *paths*, *no\_stats\_fc*, *var\_suffix*, *prot\_rkws*, *output\_lvl=1*, *log\_scopt={'logfp': None, 'logopt': True, 'output\_lvl': 1, 'printopt': False}*, *plt\_scopt={'clear': True, 'close': True, 's\_types': ['pdf'], 'save': True, 'show': True, 'tight': True}*)[source]
:   Evaluates a series of single evaluations (one after an other).

    Parameters
    :   - **eva\_single\_func** (*function*) – Function to perform single evaluation.
        - **paths** (*pandas.Series*) –

          Paths for evaluation:
          :   - ’prot’: path to protocoll excel table
              - ’opts’: path to common options as json file
              - ’meas’: path to measurement (conventional) file location
              - ’dic’: path to measurement (optical) file location
              - ’out’: path for output files
        - **no\_stats\_fc** (*list* *of* *strings*) –

          No evaluation if in protocoll.Failure\_code.
          Example: no\_stats\_fc = [‘A01.1’,’A01.2’,’A01.3’, ‘A02.3’,

          > ’B01.1’,’B01.2’,’B01.3’, ‘B02.3’,
          > ‘C01.1’,’C01.2’,’C01.3’, ‘C02.3’,
          > ‘D01.1’,’D01.2’,’D01.3’, ‘D02.3’,
          > ‘F01.1’,’F01.2’,’F01.3’, ‘F02.3’]
        - **var\_suffix** (*string*) – Suffix for variant of measurement (p.E. different moistures [“A”,”B”,…]).
        - **prot\_rkws** (*dict*) – Dictionary for reading protocol. Must be keyword ind pandas.read\_excel.
        - **output\_lvl** (*int**,* *optional*) – Output level (0: no output, 1: only necessary, 2: all).
          The default is 1.

    Return type
    :   None.

## Module contents

Previous
Next

---

© Copyright 2024, MarcGebhardt.

Built with Sphinx using a
theme
provided by Read the Docs.
