# Supplementary material for: Approach to Standardized Material Characterization of the Human Lumbopelvic System: Testing and Evaluation
Source: Bioengineering (Basel). 2025 Aug 11;12(8):862. doi: 10.3390/bioengineering12080862 (PMC12383908; doi:10.3390/bioengineering12080862)
Supplement: Supplementary file 1 [file bioengineering-12-00862-s001.zip › File S3 Evaluation code/ExMechEva-0.1.2/docs/_build/html/genindex.html]

Index — ExMechEva v0.1.2 documentation


ExMechEva

Contents:

- ExMechEva

ExMechEva

- Index

---

# Index

**\_**
| **A**
| **B**
| **C**
| **D**
| **E**
| **F**
| **G**
| **H**
| **I**
| **L**
| **M**
| **N**
| **O**
| **P**
| **R**
| **S**
| **T**
| **V**
| **W**
| **Y**

## \_

|  |
| --- |
| - \_\_call\_\_() (exmecheva.bending.bfunc\_class.Bend\_func\_sub method) |

## A

|  |  |
| --- | --- |
| - ACT\_single() (in module exmecheva.Eva\_ACT) | - agg\_add\_ci() (in module exmecheva.common.stat\_ext) - ATT\_single() (in module exmecheva.Eva\_ATT) |

## B

|  |  |
| --- | --- |
| - Bend\_func\_cohort (class in exmecheva.bending.bfunc\_class) - Bend\_func\_legion (class in exmecheva.bending.bfunc\_class) | - Bend\_func\_sub (class in exmecheva.bending.bfunc\_class) - Builder() (exmecheva.bending.bfunc\_class.Bend\_func\_legion method) |

## C

|  |  |
| --- | --- |
| - CD\_rep() (in module exmecheva.common.stat\_ext) - CD\_test\_multi() (in module exmecheva.common.stat\_ext) - check\_empty() (in module exmecheva.common.helper) - check\_params() (in module exmecheva.common.mc\_man) - CImax() (in module exmecheva.common.stat\_ext) - CImin() (in module exmecheva.common.stat\_ext) - coefficient\_of\_variation() (in module exmecheva.common.stat\_ext) - coefficient\_of\_variation\_woso() (in module exmecheva.common.stat\_ext) - colplt\_common\_ax() (in module exmecheva.bending.plotting) - colplt\_df\_ax() (in module exmecheva.bending.plotting) - colplt\_funcs\_all() (in module exmecheva.bending.plotting) - colplt\_funcs\_ax() (in module exmecheva.bending.plotting) - colplt\_funcs\_one() (in module exmecheva.bending.plotting) | - com\_option\_file\_read() (in module exmecheva.common.eva\_opt\_hand) - com\_option\_file\_write() (in module exmecheva.common.eva\_opt\_hand) - comb\_logs() (in module exmecheva.common.loadnsave) - confidence\_interval() (in module exmecheva.common.stat\_ext) - coord\_df\_depo() (in module exmecheva.bending.evaluation) - coord\_df\_mean() (in module exmecheva.bending.evaluation) - Corr\_ext() (in module exmecheva.common.stat\_ext) - curve\_char\_plotter() (in module exmecheva.common.plotting) - curve\_characterizer() (in module exmecheva.common.mc\_char) - curve\_merger() (in module exmecheva.common.mc\_char) - curvecar\_refine() (in module exmecheva.common.mc\_char) - curvecar\_section() (in module exmecheva.common.mc\_char) - cv() (in module exmecheva.common.stat\_ext) - cvwoso() (in module exmecheva.common.stat\_ext) |

## D

|  |  |
| --- | --- |
| - deal\_dupl\_index() (in module exmecheva.common.pd\_ext) - DetFinSSC() (in module exmecheva.common.mc\_man) - Diff\_ext() (in module exmecheva.common.mc\_man) - Diff\_Quot() (in module exmecheva.common.mc\_char) | - Diff\_Quot2() (in module exmecheva.common.mc\_char) - Diff\_Quot3() (in module exmecheva.common.mc\_char) - Dist\_test() (in module exmecheva.common.stat\_ext) - Dist\_test\_multi() (in module exmecheva.common.stat\_ext) |

## E

|  |  |
| --- | --- |
| - exmecheva   - module - exmecheva.bending   - module - exmecheva.bending.attr\_bgl   - module - exmecheva.bending.bfunc\_class   - module - exmecheva.bending.bfunc\_com   - module - exmecheva.bending.bfunc\_fse   - module - exmecheva.bending.evaluation   - module - exmecheva.bending.fitting   - module - exmecheva.bending.opt\_mps   - module - exmecheva.bending.plotting   - module - exmecheva.common   - module - exmecheva.common.analyze   - module - exmecheva.common.eva\_opt\_hand   - module - exmecheva.common.fitting   - module - exmecheva.common.helper   - module | - exmecheva.common.list\_ops   - module - exmecheva.common.loadnsave   - module - exmecheva.common.mc\_char   - module - exmecheva.common.mc\_man   - module - exmecheva.common.mc\_yield   - module - exmecheva.common.output   - module - exmecheva.common.pd\_ext   - module - exmecheva.common.plotting   - module - exmecheva.common.stat\_ext   - module - exmecheva.eva   - module - exmecheva.Eva\_ACT   - module - exmecheva.Eva\_ATT   - module - exmecheva.Eva\_TBT   - module - Extend\_Series\_n\_setter() (in module exmecheva.common.mc\_man) - Extend\_Series\_Poly() (in module exmecheva.common.mc\_man) |

## F

|  |  |
| --- | --- |
| - Failure\_code\_bool\_df() (in module exmecheva.common.list\_ops) - Failure\_code\_checker() (in module exmecheva.common.list\_ops) - Failure\_code\_format() (in module exmecheva.common.list\_ops) - Failure\_code\_lister() (in module exmecheva.common.list\_ops) - file\_namer() (in module exmecheva.common.loadnsave) - file\_namer\_interpreter() (in module exmecheva.common.loadnsave) - Find\_closest() (in module exmecheva.common.pd\_ext) - Find\_closest\_perc() (in module exmecheva.common.pd\_ext) - Find\_closestv() (in module exmecheva.common.pd\_ext) - Find\_first\_sc() (in module exmecheva.common.pd\_ext) - Find\_intg2p() (in module exmecheva.common.mc\_yield) - find\_SandE() (in module exmecheva.common.mc\_char) - fit\_report\_adder() (in module exmecheva.common.fitting) - FSE\_4sin\_d0() (in module exmecheva.bending.bfunc\_fse) - FSE\_4sin\_d1() (in module exmecheva.bending.bfunc\_fse) | - FSE\_4sin\_d2() (in module exmecheva.bending.bfunc\_fse) - FSE\_4sin\_lin\_func\_d0() (in module exmecheva.bending.bfunc\_fse) - FSE\_4sin\_lin\_func\_d1() (in module exmecheva.bending.bfunc\_fse) - FSE\_4sin\_lin\_func\_d2() (in module exmecheva.bending.bfunc\_fse) - FSE\_4sin\_wlin\_d0() (in module exmecheva.bending.bfunc\_fse) - FSE\_4sin\_wlin\_d1() (in module exmecheva.bending.bfunc\_fse) - FSE\_4sin\_wlin\_d2() (in module exmecheva.bending.bfunc\_fse) - FSE\_SF\_func\_d0() (in module exmecheva.bending.bfunc\_fse) - FSE\_SF\_func\_d1() (in module exmecheva.bending.bfunc\_fse) - FSE\_SF\_func\_d2() (in module exmecheva.bending.bfunc\_fse) - func\_exp() (in module exmecheva.common.fitting) - func\_exp\_str() (in module exmecheva.common.fitting) - func\_lin() (in module exmecheva.common.fitting) - func\_lin\_str() (in module exmecheva.common.fitting) - func\_pow() (in module exmecheva.common.fitting) - func\_pow\_str() (in module exmecheva.common.fitting) |

## G

|  |  |
| --- | --- |
| - gamma\_V\_det() (in module exmecheva.bending.bfunc\_com) - Geo\_curve\_TBC() (in module exmecheva.common.analyze) | - group\_Anova() (in module exmecheva.common.stat\_ext) - group\_ANOVA\_MComp() (in module exmecheva.common.stat\_ext) - group\_ANOVA\_MComp\_multi() (in module exmecheva.common.stat\_ext) |

## H

|  |  |
| --- | --- |
| - Hypo\_test() (in module exmecheva.common.stat\_ext) | - Hypo\_test\_multi() (in module exmecheva.common.stat\_ext) |

## I

|  |  |
| --- | --- |
| - ICD\_bool\_df() (in module exmecheva.common.list\_ops) - ICD\_lister() (in module exmecheva.common.list\_ops) | - Init\_fandds() (exmecheva.bending.bfunc\_class.Bend\_func\_cohort method) - Inter\_Lines() (in module exmecheva.common.analyze) |

## L

|  |  |
| --- | --- |
| - Line\_from2P() (in module exmecheva.common.analyze) - list\_boolean\_df() (in module exmecheva.common.list\_ops) - list\_cell\_compiler() (in module exmecheva.common.list\_ops) - list\_interpreter() (in module exmecheva.common.list\_ops) - list\_ser\_to\_1D() (in module exmecheva.common.list\_ops) | - lmfit\_bound\_checker() (in module exmecheva.bending.fitting) - lmfit\_free\_val\_setter() (in module exmecheva.bending.fitting) - lmfit\_modelize() (in module exmecheva.bending.fitting) - lmfit\_param\_adder() (in module exmecheva.bending.fitting) - lmfit\_param\_key\_checker() (in module exmecheva.bending.fitting) - lmfit\_param\_prep() (in module exmecheva.bending.fitting) |

## M

|  |  |
| --- | --- |
| - mc\_resampler() (in module exmecheva.common.mc\_man) - MComp\_interpreter() (in module exmecheva.common.stat\_ext) - meanwoso() (in module exmecheva.common.stat\_ext) - module   - exmecheva   - exmecheva.bending   - exmecheva.bending.attr\_bgl   - exmecheva.bending.bfunc\_class   - exmecheva.bending.bfunc\_com   - exmecheva.bending.bfunc\_fse   - exmecheva.bending.evaluation   - exmecheva.bending.fitting   - exmecheva.bending.opt\_mps   - exmecheva.bending.plotting   - exmecheva.common   - exmecheva.common.analyze   - exmecheva.common.eva\_opt\_hand   - exmecheva.common.fitting   - exmecheva.common.helper   - exmecheva.common.list\_ops   - exmecheva.common.loadnsave   - exmecheva.common.mc\_char   - exmecheva.common.mc\_man   - exmecheva.common.mc\_yield   - exmecheva.common.output   - exmecheva.common.pd\_ext   - exmecheva.common.plotting   - exmecheva.common.stat\_ext   - exmecheva.eva   - exmecheva.Eva\_ACT   - exmecheva.Eva\_ATT   - exmecheva.Eva\_TBT | - Moment\_perF\_func() (in module exmecheva.bending.evaluation) - Multi\_conc() (in module exmecheva.common.stat\_ext) - Multi\_minimize() (in module exmecheva.bending.fitting) |

## N

|  |  |
| --- | --- |
| - NaN\_stat\_outliers() (in module exmecheva.common.stat\_ext) | - normalize() (in module exmecheva.common.analyze) - normalize\_th() (in module exmecheva.common.analyze) |

## O

|  |  |
| --- | --- |
| - option\_presetter() (in module exmecheva.common.eva\_opt\_hand) - option\_reader() (in module exmecheva.common.eva\_opt\_hand) | - option\_reader\_sel() (in module exmecheva.common.eva\_opt\_hand) - Otvalgetter\_Multi() (in module exmecheva.common.output) - Outvalgetter() (in module exmecheva.common.output) |

## P

|  |  |
| --- | --- |
| - pack\_hdf() (in module exmecheva.common.loadnsave) - pack\_hdf\_mul() (in module exmecheva.common.loadnsave) - pd\_agg() (in module exmecheva.common.stat\_ext) - pd\_agg\_custom() (in module exmecheva.common.stat\_ext) - pd\_axischange() (in module exmecheva.common.pd\_ext) - pd\_combine\_index() (in module exmecheva.common.pd\_ext) - pd\_exclnan() (in module exmecheva.common.pd\_ext) - pd\_find\_index() (in module exmecheva.common.pd\_ext) - pd\_isDF() (in module exmecheva.common.pd\_ext) - pd\_isSer() (in module exmecheva.common.pd\_ext) - pd\_limit() (in module exmecheva.common.pd\_ext) - pd\_nan\_handler() (in module exmecheva.common.pd\_ext) - pd\_outsort() (in module exmecheva.common.pd\_ext) - pd\_slice\_index() (in module exmecheva.common.pd\_ext) - pd\_trapz() (in module exmecheva.common.pd\_ext) - pd\_valid\_index() (in module exmecheva.common.pd\_ext) - pd\_vec\_length() (in module exmecheva.common.pd\_ext) - peaky\_finder() (in module exmecheva.common.mc\_char) | - peaky\_finder\_MM() (in module exmecheva.common.mc\_char) - Perform\_Fit() (in module exmecheva.bending.fitting) - plt\_add\_DaAnno() (in module exmecheva.common.plotting) - plt\_ax\_regfit() (in module exmecheva.common.plotting) - plt\_handle\_suffix() (in module exmecheva.common.plotting) - poi\_det\_plh() (in module exmecheva.common.mc\_char) - poi\_fixeva() (in module exmecheva.common.mc\_char) - poi\_refinement() (in module exmecheva.common.mc\_char) - poi\_rel\_finder() (in module exmecheva.common.mc\_char) - poi\_vip\_namer() (in module exmecheva.common.mc\_char) - Point\_df\_combine() (in module exmecheva.bending.opt\_mps) - Point\_df\_from\_lin() (in module exmecheva.bending.opt\_mps) - Point\_df\_idx() (in module exmecheva.bending.opt\_mps) - Point\_df\_transform() (in module exmecheva.bending.opt\_mps) - Points\_add\_step() (in module exmecheva.bending.opt\_mps) - Points\_dif\_step() (in module exmecheva.bending.opt\_mps) - Points\_diff() (in module exmecheva.bending.opt\_mps) - Points\_eval\_func() (in module exmecheva.bending.opt\_mps) - Predict\_apply\_retrim() (in module exmecheva.common.mc\_man) |

## R

|  |  |
| --- | --- |
| - Refit\_YM\_vals() (in module exmecheva.common.fitting) - reg\_stats\_multi() (in module exmecheva.common.stat\_ext) - regfitret() (in module exmecheva.common.fitting) - regfitret\_restring\_func() (in module exmecheva.common.fitting) - relative\_deviation() (in module exmecheva.common.stat\_ext) | - res\_multi\_const\_weighted() (in module exmecheva.bending.fitting) - Retrim\_Series() (in module exmecheva.common.mc\_man) - rise\_curve() (in module exmecheva.common.mc\_char) - round\_to\_sigdig() (in module exmecheva.common.helper) - Rquad() (in module exmecheva.common.fitting) |

## S

|  |  |
| --- | --- |
| - selector() (in module exmecheva.eva) - series() (in module exmecheva.eva) - set\_type\_by\_string() (in module exmecheva.common.eva\_opt\_hand) - shaped\_array\_fill\_fandl() (in module exmecheva.bending.fitting) - Shear\_area() (in module exmecheva.bending.bfunc\_com) - sigdig() (in module exmecheva.common.helper) - sign\_n\_change() (in module exmecheva.common.analyze) - sign\_n\_changeth() (in module exmecheva.common.analyze) - smooth() (in module exmecheva.common.mc\_man) - Smoothsel() (in module exmecheva.common.mc\_man) - Smoothsel\_ext() (in module exmecheva.common.mc\_man) - sns\_pointplot\_MMeb() (in module exmecheva.common.plotting) | - stat\_box\_vals() (in module exmecheva.common.stat\_ext) - stat\_outliers() (in module exmecheva.common.stat\_ext) - stdwoso() (in module exmecheva.common.stat\_ext) - str\_indent() (in module exmecheva.common.output) - str\_log() (in module exmecheva.common.output) - str\_to\_bool() (in module exmecheva.common.helper) - strain\_linfit() (in module exmecheva.common.fitting) - straindf\_from\_curve() (in module exmecheva.bending.evaluation) - stress\_df\_from\_lin() (in module exmecheva.bending.evaluation) - stress\_linfit() (in module exmecheva.common.fitting) - stress\_linfit\_plt() (in module exmecheva.common.fitting) - stress\_perF() (in module exmecheva.bending.evaluation) |

## T

|  |  |
| --- | --- |
| - TBT\_single() (in module exmecheva.Eva\_TBT) - test\_pdmon() (in module exmecheva.common.mc\_char) - threshhold\_setter() (in module exmecheva.common.analyze) - tick\_label\_inserter() (in module exmecheva.common.plotting) - tick\_label\_renamer() (in module exmecheva.common.plotting) - tick\_legend\_renamer() (in module exmecheva.common.plotting) | - TP\_circle() (in module exmecheva.common.analyze) - TP\_radius() (in module exmecheva.common.analyze) - triangle\_func\_d0() (in module exmecheva.bending.bfunc\_com) - triangle\_func\_d1() (in module exmecheva.bending.bfunc\_com) - triangle\_func\_d2() (in module exmecheva.bending.bfunc\_com) - type\_str\_return() (in module exmecheva.common.helper) |

## V

|  |  |
| --- | --- |
| - v\_Ctrans() (in module exmecheva.bending.opt\_mps) | - v\_length() (in module exmecheva.bending.opt\_mps) |

## W

|  |
| --- |
| - Weight\_func() (in module exmecheva.bending.evaluation) |

## Y

|  |  |
| --- | --- |
| - Yield\_redet() (in module exmecheva.common.mc\_yield) - Yield\_redet2() (in module exmecheva.common.mc\_yield) - Yield\_redet2\_Multi() (in module exmecheva.common.mc\_yield) - YM\_check\_many\_with\_method\_D() (in module exmecheva.bending.evaluation) - YM\_check\_with\_method\_D() (in module exmecheva.bending.evaluation) - YM\_eva\_com\_sel() (in module exmecheva.common.fitting) - YM\_eva\_method\_A() (in module exmecheva.bending.evaluation) - YM\_eva\_method\_B() (in module exmecheva.bending.evaluation) - YM\_eva\_method\_C() (in module exmecheva.bending.evaluation) - YM\_eva\_method\_D() (in module exmecheva.bending.evaluation) | - YM\_eva\_method\_D\_bend\_df() (in module exmecheva.bending.evaluation) - YM\_eva\_method\_D\_bend\_df\_add() (in module exmecheva.bending.evaluation) - YM\_eva\_method\_D\_num() (in module exmecheva.bending.evaluation) - YM\_eva\_method\_D\_res() (in module exmecheva.bending.evaluation) - YM\_eva\_method\_E() (in module exmecheva.bending.evaluation) - YM\_eva\_method\_F() (in module exmecheva.bending.evaluation) - YM\_eva\_method\_G() (in module exmecheva.bending.evaluation) - YM\_eva\_range\_refine() (in module exmecheva.common.mc\_char)   - (in module exmecheva.common.mc\_yield) - YM\_sigeps\_lin() (in module exmecheva.common.fitting) |

---

© Copyright 2024, MarcGebhardt.

Built with Sphinx using a
theme
provided by Read the Docs.
