# Supplementary material for: Approach to Standardized Material Characterization of the Human Lumbopelvic System: Testing and Evaluation
Source: Bioengineering (Basel). 2025 Aug 11;12(8):862. doi: 10.3390/bioengineering12080862 (PMC12383908; doi:10.3390/bioengineering12080862)
Supplement: Supplementary file 1 [file bioengineering-12-00862-s001.zip › File S3 Evaluation code/ExMechEva-0.1.2/docs/_build/html/index.html]

Welcome to ExMechEva’s documentation! — ExMechEva v0.1.2 documentation


ExMechEva

Contents:

- ExMechEva

ExMechEva

- Welcome to ExMechEva’s documentation!
- View page source

---

# Welcome to ExMechEva’s documentation!

Contents:

- ExMechEva
  - exmecheva package

# Indices and tables

- Index
- Module Index
- Search Page

Next

---

© Copyright 2024, MarcGebhardt.

Built with Sphinx using a
theme
provided by Read the Docs.
