# Supplementary material for: Approach to Standardized Material Characterization of the Human Lumbopelvic System: Testing and Evaluation
Source: Bioengineering (Basel). 2025 Aug 11;12(8):862. doi: 10.3390/bioengineering12080862 (PMC12383908; doi:10.3390/bioengineering12080862)
Supplement: Supplementary file 1 [file bioengineering-12-00862-s001.zip › File S3 Evaluation code/ExMechEva-0.1.2/docs/_build/html/modules.html]

ExMechEva — ExMechEva v0.1.2 documentation


ExMechEva

Contents:

- ExMechEva
  - exmecheva package

ExMechEva

- ExMechEva
- View page source

---

# ExMechEva

- exmecheva package
  - Subpackages
    - exmecheva.bending package
      - Submodules
      - exmecheva.bending.attr\_bgl module
      - exmecheva.bending.bfunc\_class module
      - exmecheva.bending.bfunc\_com module
      - exmecheva.bending.bfunc\_fse module
      - exmecheva.bending.evaluation module
      - exmecheva.bending.fitting module
      - exmecheva.bending.opt\_mps module
      - exmecheva.bending.plotting module
      - Module contents
    - exmecheva.common package
      - Submodules
      - exmecheva.common.analyze module
      - exmecheva.common.eva\_opt\_hand module
      - exmecheva.common.fitting module
      - exmecheva.common.helper module
      - exmecheva.common.list\_ops module
      - exmecheva.common.loadnsave module
      - exmecheva.common.mc\_char module
      - exmecheva.common.mc\_man module
      - exmecheva.common.mc\_yield module
      - exmecheva.common.output module
      - exmecheva.common.pd\_ext module
      - exmecheva.common.plotting module
      - exmecheva.common.stat\_ext module
      - Module contents
  - Submodules
  - exmecheva.Eva\_ACT module
    - `ACT_single()`
  - exmecheva.Eva\_ATT module
    - `ATT_single()`
  - exmecheva.Eva\_TBT module
    - `TBT_single()`
  - exmecheva.eva module
    - `selector()`
    - `series()`
  - Module contents

Previous
Next

---

© Copyright 2024, MarcGebhardt.

Built with Sphinx using a
theme
provided by Read the Docs.
