# Supplementary material for: Approach to Standardized Material Characterization of the Human Lumbopelvic System: Testing and Evaluation
Source: Bioengineering (Basel). 2025 Aug 11;12(8):862. doi: 10.3390/bioengineering12080862 (PMC12383908; doi:10.3390/bioengineering12080862)
Supplement: Supplementary file 1 [file bioengineering-12-00862-s001.zip › File S3 Evaluation code/ExMechEva-0.1.2/docs/_build/html/py-modindex.html]

Python Module Index — ExMechEva v0.1.2 documentation


ExMechEva

Contents:

- ExMechEva

ExMechEva

- Python Module Index

---

# Python Module Index

**e**

|  |  |  |
| --- | --- | --- |
|  |  |  |
|  | **e** |  |
|  | `exmecheva` |  |
|  | `exmecheva.bending` |  |
|  | `exmecheva.bending.attr_bgl` |  |
|  | `exmecheva.bending.bfunc_class` |  |
|  | `exmecheva.bending.bfunc_com` |  |
|  | `exmecheva.bending.bfunc_fse` |  |
|  | `exmecheva.bending.evaluation` |  |
|  | `exmecheva.bending.fitting` |  |
|  | `exmecheva.bending.opt_mps` |  |
|  | `exmecheva.bending.plotting` |  |
|  | `exmecheva.common` |  |
|  | `exmecheva.common.analyze` |  |
|  | `exmecheva.common.eva_opt_hand` |  |
|  | `exmecheva.common.fitting` |  |
|  | `exmecheva.common.helper` |  |
|  | `exmecheva.common.list_ops` |  |
|  | `exmecheva.common.loadnsave` |  |
|  | `exmecheva.common.mc_char` |  |
|  | `exmecheva.common.mc_man` |  |
|  | `exmecheva.common.mc_yield` |  |
|  | `exmecheva.common.output` |  |
|  | `exmecheva.common.pd_ext` |  |
|  | `exmecheva.common.plotting` |  |
|  | `exmecheva.common.stat_ext` |  |
|  | `exmecheva.eva` |  |
|  | `exmecheva.Eva_ACT` |  |
|  | `exmecheva.Eva_ATT` |  |
|  | `exmecheva.Eva_TBT` |  |

---

© Copyright 2024, MarcGebhardt.

Built with Sphinx using a
theme
provided by Read the Docs.
