# Supplementary material for: Approach to Standardized Material Characterization of the Human Lumbopelvic System: Testing and Evaluation
Source: Bioengineering (Basel). 2025 Aug 11;12(8):862. doi: 10.3390/bioengineering12080862 (PMC12383908; doi:10.3390/bioengineering12080862)
Supplement: Supplementary file 1 [file bioengineering-12-00862-s001.zip › File S3 Evaluation code/ExMechEva-0.1.2/docs/_build/html/_modules/exmecheva/bending/bfunc_class.html]

exmecheva.bending.bfunc\_class — ExMechEva 0.1.0 documentation


ExMechEva

Contents:

- ExMechEva

ExMechEva

- Module code
- exmecheva.bending.bfunc\_class

---

# Source code for exmecheva.bending.bfunc\_class

```
# -*- coding: utf-8 -*-
"""
Classes for bending functions.

@author: MarcGebhardt
"""
import numpy as np
import pandas as pd
import sympy as sy
# import dill # saving banding func legion does not work

from .opt_mps import (Point_df_from_lin, Points_eval_func)
from .bfunc_fse import (
    FSE_4sin_wlin_d0, FSE_4sin_wlin_d1, FSE_4sin_wlin_d2,
    FSE_4sin_lin_func_d0, FSE_4sin_lin_func_d1, FSE_4sin_lin_func_d2,
    FSE_4sin_d0, FSE_4sin_d1, FSE_4sin_d2,
    FSE_SF_func_d0, FSE_SF_func_d1, FSE_SF_func_d2
    )

[docs]class Bend_func_sub:
    """Lowest element which provides necessary function informations."""
    def __init__(self, func, sy_expr, sy_string,
                 independent_vars=None, param_names=None, param_types=None, name=None, **kws):
        """
        Subfunction takes callable function and additional informations.

        Parameters
        ----------
        func : callable
            Function.
        sy_expr : sympy expression
            Sympy expression.
        sy_string : string expression
            Expression string.
        independent_vars : array of string | string, optional
            Arguments to `func` that are independent variables. The default is None.
        param_names : array of string, optional
            Names of arguments to `func` that are to be made into
            parameters. The default is None.
        name : string, optional
            Name of function. The default is None.
        **kws : TYPE
            Additional keyword arguments to pass.

        Returns
        -------
        None.

        """
        self.func=func
        self.sy_expr=sy_expr
        self.sy_string=sy_string
        self.independent_vars=independent_vars
        self.param_names=param_names
        # self.param_types=param_types
        # self.param_types=Bend_func_sub._set_free_type(self)
        _param_types=Bend_func_sub._set_free_type(param_names,param_types)
        self.param_types=_param_types
        self.name=name
        self.opts = kws

[docs]    def __call__(self, x, kws, **opts):
        """
        Call and evaluate underlayed function.
        If x is an array of float and kws is a OrderedDict, result will be an array of float.
        If x is a Dataframe or float and kws is a Series, evaluates each index in kws.

        Parameters
        ----------
        x : array of float | Dataframe of type Points
            Independet variables to evaluate function.
        **kws : OrderedDict | Series of OrderedDicts
            Additional keyword arguments to pass to func
            (p.e. parameter value dictionary).

        Returns
        -------
        array of float
            Evaluation values of function to x and kws.

        """
        if (type(x) is pd.core.frame.DataFrame) and (type(kws) is pd.core.series.Series):
            return Points_eval_func(self.func, fit_params=kws, pointdf=x, steps=kws.index)
        if (type(x) is np.ndarray) and (type(kws) is pd.core.series.Series):
            # xt='L'+pd.Series(x).index.map(str)
            # mit=pd.MultiIndex.from_product([xt,['y']],names=['Points','Vars'])
            # # dft=pd.DataFrame([],index=kws.index,columns=mit)
            # # dft.loc[:]=x
            # # return Points_eval_func(self.func, fit_params=kws, pointdf=dft, steps=kws.index)
            dfs=kws.apply(lambda b: self.func(x,**b))
            dft=pd.DataFrame(item for item in dfs)
            dft.index=kws.index
            if not 'coords'       in opts: opts.update({'coords':      ['y']})
            if not 'coords_set'   in opts: opts.update({'coords_set':   None})
            if not 'col_type'     in opts: opts.update({'col_type':    'str'})
            if not 'Point_prefix' in opts: opts.update({'Point_prefix':'L'  })
            mit=Point_df_from_lin(x=x, steps=[0], **opts).columns
            dft.columns=mit
            return dft
        if (type(x) is pd.core.indexes.numeric.Float64Index) and (type(kws) is pd.core.series.Series):
            dfs=kws.apply(lambda b: self.func(x.to_numpy(),**b))
            dft=pd.DataFrame(item for item in dfs)
            dft.index=kws.index
            dft.columns=x
            return dft
        if type(x) is int: x=float(x)
        if (type(x) is float) and (type(kws) is pd.core.series.Series):
            dfs=kws.apply(lambda b: self.func(x,**b))
            dfs.name=self.name+'>'+str(x)
            return dfs
        return self.func(x,**kws)

def __getitem__(self,obj):
        return getattr(self, obj)
    
    def __str__(self):
        return self.sy_string
    
    # def _set_free_type(self):
    #     """Set all remaining parameter types, not specified in types to 'free'."""
    #     self.param_types = {i: (self.param_types[i] if i in self.param_types.keys() else 'free') 
    #                         for i in self.param_names}
    def _set_free_type(pnames,ptypes):
        """Set all remaining parameter types, not specified in types to 'free'."""
        param_types = {i: (ptypes[i] if i in ptypes.keys() else 'free') 
                            for i in pnames}
        return param_types

# # Test für bessere zuordnung    
# class Bend_func_sub_FSE_4sin_wlin_d0(Bend_func_sub):
#     def __init__(self, name=None, **kws):
#         def FSE_4sin_wlin_d0(x, xmin,xmax,FP, b1,b2,b3,b4,c,d, f_V_0=None):
#             eq=b1*np.sin(FP*(x-xmin))+b2*np.sin(2*FP*(x-xmin))+b3*np.sin(3*FP*(x-xmin))+b4*np.sin(4*FP*(x-xmin))+c*(x-xmin)+d
#             return eq
#         f  = FSE_4sin_wlin_d0
#         ss = 'b1*sin(FP*(x-xmin))+b2*sin(2*FP*(x-xmin))+b3*sin(3*FP*(x-xmin))+b4*sin(4*FP*(x-xmin))+c*(x-xmin)+d'
#         se = sy.parse_expr(ss)
#         param_names = np.array(['x','xmin','xmax','FP','b1','b2','b3','b4','c','d','f_V_0'])
#         super().__init__(func=f, sy_expr=se, sy_string=ss,
#                          independent_vars='x', param_names=param_names,
#                          name=name, **kws)

[docs]class Bend_func_cohort(object):
    """Function collection of Subfunctions (Bend_func_sub)"""
    
    # @property
    # def _constructor(self) -> type[Bend_func_cohort]:
    #     return Bend_func_cohort
    
    def __init__(self, d0=None, d1=None, d2=None,
                 name=None, **kws):
        # self.d0=Bend_func_sub.d0
        # self.d1=Bend_func_sub.d1
        # self.d2=Bend_func_sub.d2
        self.name=name
        self.opts=kws
            
    def __getitem__(self,obj):
        return getattr(self, obj)

[docs]    def Init_fandds(self, expr_d0, var_names, var='x', var_types=None,
                    option='d0_str_to_all',
                    expr_d1=None, expr_d2=None,
                    func_d0=None, func_d1=None, func_d2=None):
        """
        Return Sympy expressions and associated lambdified functions, depending on option-string.
    
        Parameters
        ----------
        expr_d0 : string or sympy.expression
            String or expression, which determine function.
        var_names : TYPE
            Variable names for parsing.
        var : TYPE, optional
            Variable name for derivation. The default is 'x'.
        option : string, optional
            Option of function generation, in form of which(d0/each)_type(str/expr)_to_what(d0/all). The default is 'd0_str_to_all'.
        expr_d1 : string or sympy.expression, optional
            String or expression, which determine 1st derivate of function. The default is None.
        expr_d2 : string or sympy.expression, optional
            String or expression, which determine 2nd derivate of function. The default is None.
    
        Raises
        ------
        NotImplementedError
            Error for not implemented option.
    
        Returns
        -------
        func_package : Bend_func_cohort
            A function collection of all generated sympy expressions
            and associated lambdified functions and their 1st and 2nd derivates.
    
        """
        if option == 'func_to_all':
            self.d0=Bend_func_sub(func=func_d0, sy_expr=sy.parse_expr(expr_d0), sy_string=expr_d0,
                                  name=self.name+'_d0', independent_vars=var,
                                  param_names=var_names, param_types=var_types)
            self.d1=Bend_func_sub(func=func_d1, sy_expr=sy.parse_expr(expr_d1), sy_string=expr_d1,
                                  name=self.name+'_d1', independent_vars=var,
                                  param_names=var_names, param_types=var_types)
            self.d2=Bend_func_sub(func=func_d2, sy_expr=sy.parse_expr(expr_d2), sy_string=expr_d2,
                                  name=self.name+'_d2', independent_vars=var,
                                  param_names=var_names, param_types=var_types)
        else:            
            if option=='d0_str_to_all':
                    d0 = sy.parse_expr(expr_d0)
                    d1 = sy.simplify(d0.diff(var))
                    d2 = sy.simplify(d1.diff(var))
            elif option=='d0_expr_to_all':
                    d0 = expr_d0
                    d1 = sy.simplify(d0.diff(var))
                    d2 = sy.simplify(d1.diff(var))
            elif option== 'each_str_to_all':
                    d0 = sy.parse_expr(expr_d0)
                    d1 = sy.parse_expr(expr_d1)
                    d2 = sy.parse_expr(expr_d2)
            elif option== 'each_expr_to_all':
                    d0 = expr_d0
                    d1 = expr_d1
                    d2 = expr_d2
            elif option== 'd0_str_to_d0':
                    d0 = sy.parse_expr(expr_d0)
            elif option== 'd0_expr_to_d0':
                    d0 = expr_d0
            else:
                raise NotImplementedError("Option not implemented!")
                
            
            if ('to_all') in option:
                d0_func = sy.lambdify(var_names, d0)
                d1_func = sy.lambdify(var_names, d1)
                d2_func = sy.lambdify(var_names, d2)
                self.d0=Bend_func_sub(func=d0_func, sy_expr=d0, sy_string=expr_d0.__str__(),
                                             name=self.name+'_d0', independent_vars=var,
                                             param_names=var_names, param_types=var_types)
                self.d1=Bend_func_sub(func=d1_func, sy_expr=d1, sy_string=d1.__str__(),
                                             name=self.name+'_d1', independent_vars=var,
                                             param_names=var_names, param_types=var_types)
                self.d2=Bend_func_sub(func=d2_func, sy_expr=d2, sy_string=d2.__str__(),
                                             name=self.name+'_d2', independent_vars=var,
                                             param_names=var_names, param_types=var_types)
    
            elif ('to_d0') in option:
                d0_func = sy.lambdify(var_names, d0)
                self.d0=Bend_func_sub(func=d0_func, sy_expr=d0, sy_string=expr_d0,
                                             name='_d0', independent_vars=var,
                                             param_names=var_names, param_types=var_types)
                
            else:
                raise NotImplementedError("Option not implemented!")


[docs]class Bend_func_legion(object):
    """Bend line describing functions and their 1st and 2nd derivates
       Example:
        bl=Bend_func_legion(name='FSE fit')
        bl.Builder(option='FSE')"""
    # @property
    # def _constructor(self) -> type[Bend_func_cohort]:
    #     return Bend_func_cohort
    
    def __init__(self, name=None, description=None, **kws):
        """
        Inital properties setter.

        Parameters
        ----------
        name : string, optional
            Name of bend func legion. The default is None.
        description : TYPE, optional
            Description of bend func legion. The default is None.
        **kws : dict
            Dictionary of keyword arguments.

        Returns
        -------
        None.

        """
        # self.d0=Bend_func_sub.d0
        # self.d1=Bend_func_sub.d1
        # self.d2=Bend_func_sub.d2
        self.name=name
        self.description=description
        self.opts=kws
        
    def __getitem__(self,obj):
        return getattr(self, obj)

[docs]    def Builder(self, option='FSE'):
        """
        Build an instance of Bend per option to prepare fit of measured bend line.

        Parameters
        ----------
        option : string, optional
            Kind of . The default is 'FSE'.

        Raises
        ------
        NotImplementedError
            Building option not implemented.

        Returns
        -------
        None.

        """
        if option=='FSE':
            self.description='Bend line as lambdified Fourier series expansion'
    
            w_A_d0_str='b1*sin(FP*(x-xmin))+b2*sin(2*FP*(x-xmin))+b3*sin(3*FP*(x-xmin))+b4*sin(4*FP*(x-xmin))+c*(x-xmin)+d'
            w_variables_names=np.array(['x','xmin','xmax','FP','b1','b2','b3','b4','c','d','f_V_0'])
            self.w_A=Bend_func_cohort(name='A')
            Bend_func_cohort.Init_fandds(self.w_A, expr_d0=w_A_d0_str,
                                         var_names=w_variables_names,
                                         option='d0_str_to_all')
            
            w_I_d0_str = 'c*(x-xmin)+d'
            self.w_I=Bend_func_cohort(name='I')
            Bend_func_cohort.Init_fandds(self.w_I, expr_d0=w_I_d0_str,
                                     var_names=w_variables_names,
                                     option='d0_str_to_all')
            
            w_S_d0_exp = self.w_A.d0.sy_expr - self.w_I.d0.sy_expr
            self.w_S=Bend_func_cohort(name='S')
            Bend_func_cohort.Init_fandds(self.w_S, expr_d0=w_S_d0_exp,
                                     var_names=w_variables_names,
                                     option='d0_expr_to_all')
            
            # w_variables_names_V=np.append(w_variables_names,'f_V_0')
            w_V_d0_str = 'f_V_0*(1-2*abs(x)/(xmax-xmin))'
            w_V_d1_str = 'f_V_0*(-2*sign(x)/(xmax-xmin))'
            w_V_d2_str = '0'
            self.w_V=Bend_func_cohort(name='V')
            Bend_func_cohort.Init_fandds(self.w_V, expr_d0=w_V_d0_str,
                                     expr_d1=w_V_d1_str, expr_d2=w_V_d2_str,
                                     # var_names=w_variables_names_V,
                                     var_names=w_variables_names,
                                     option='each_str_to_all')
    
            w_Mima_d0_exp = self.w_S.d0.sy_expr - self.w_V.d0.sy_expr
            w_Mima_d1_exp = self.w_S.d1.sy_expr - self.w_V.d1.sy_expr
            w_Mima_d2_exp = self.w_S.d2.sy_expr - self.w_V.d2.sy_expr
            self.w_M_ima=Bend_func_cohort(name='M imaginary')
            Bend_func_cohort.Init_fandds(self.w_M_ima, expr_d0=w_Mima_d0_exp,
                                     expr_d1=w_Mima_d1_exp, expr_d2=w_Mima_d2_exp,
                                     # var_names=w_variables_names_V,
                                     var_names=w_variables_names,
                                     option='each_expr_to_all')
            
            w_M_d0_str='b1*sin(FP*(x-xmin))+b2*sin(2*FP*(x-xmin))+b3*sin(3*FP*(x-xmin))+b4*sin(4*FP*(x-xmin))'
            w_variables_names_M=np.array(['x','xmin','xmax','FP','b1','b2','b3','b4']) 
            self.w_M=Bend_func_cohort(name='M')
            Bend_func_cohort.Init_fandds(self.w_M, expr_d0=w_M_d0_str,
                                         var_names=w_variables_names_M,
                                         # var_names=w_variables_names,
                                         option='d0_str_to_all')
        elif option=='FSE_fixed':
            self.description='Bend line as Fourier series expansion'
    
            w_A_d0_str='b1*sin(FP*(x-xmin))+b2*sin(2*FP*(x-xmin))+b3*sin(3*FP*(x-xmin))+b4*sin(4*FP*(x-xmin))+c*(x-xmin)+d'
            w_A_d1_str='FP*b1*cos(FP*(x - xmin)) + 2*FP*b2*cos(2*FP*(x - xmin)) + 3*FP*b3*cos(3*FP*(x - xmin)) + 4*FP*b4*cos(4*FP*(x - xmin)) + c'
            w_A_d2_str='-FP**2*(b1*sin(FP*(x - xmin)) + 4*b2*sin(2*FP*(x - xmin)) + 9*b3*sin(3*FP*(x - xmin)) + 16*b4*sin(4*FP*(x - xmin)))'
            w_A_d0_func=FSE_4sin_wlin_d0
            w_A_d1_func=FSE_4sin_wlin_d1
            w_A_d2_func=FSE_4sin_wlin_d2
            w_variables_names=np.array(['x','xmin','xmax','FP',
                                        'b1','b2','b3','b4','c','d',
                                        'f_V_0'])
            w_variables_types=dict({'x':'independent',
                                    'xmin':'fixed','xmax':'fixed',
                                    'FP':'expr','f_V_0':'post'})
            
            self.w_A=Bend_func_cohort(name='A')
            Bend_func_cohort.Init_fandds(self.w_A, 
                                         expr_d0=w_A_d0_str, func_d0=w_A_d0_func,
                                         expr_d1=w_A_d1_str, func_d1=w_A_d1_func,
                                         expr_d2=w_A_d2_str, func_d2=w_A_d2_func,
                                         var_names=w_variables_names,
                                         var_types=w_variables_types,
                                         option='func_to_all')
            
            w_I_d0_str = 'c*(x-xmin)+d'
            w_I_d1_str = 'c'
            w_I_d2_str = '0'
            w_I_d0_func=FSE_4sin_lin_func_d0
            w_I_d1_func=FSE_4sin_lin_func_d1
            w_I_d2_func=FSE_4sin_lin_func_d2
            self.w_I=Bend_func_cohort(name='I')
            Bend_func_cohort.Init_fandds(self.w_I,
                                         expr_d0=w_I_d0_str, func_d0=w_I_d0_func,
                                         expr_d1=w_I_d1_str, func_d1=w_I_d1_func,
                                         expr_d2=w_I_d2_str, func_d2=w_I_d2_func,
                                         var_names=w_variables_names,
                                         var_types=w_variables_types,
                                         option='func_to_all')
            
            w_S_d0_str = 'b1*sin(FP*(x - xmin)) + b2*sin(2*FP*(x - xmin)) + b3*sin(3*FP*(x - xmin)) + b4*sin(4*FP*(x - xmin))'
            w_S_d1_str = 'FP*(b1*cos(FP*(x - xmin)) + 2*b2*cos(2*FP*(x - xmin)) + 3*b3*cos(3*FP*(x - xmin)) + 4*b4*cos(4*FP*(x - xmin)))'
            w_S_d2_str = '-FP**2*(b1*sin(FP*(x - xmin)) + 4*b2*sin(2*FP*(x - xmin)) + 9*b3*sin(3*FP*(x - xmin)) + 16*b4*sin(4*FP*(x - xmin)))'
            w_S_d0_func=FSE_4sin_d0
            w_S_d1_func=FSE_4sin_d1
            w_S_d2_func=FSE_4sin_d2
            self.w_S=Bend_func_cohort(name='S')
            Bend_func_cohort.Init_fandds(self.w_S,
                                         expr_d0=w_S_d0_str, func_d0=w_S_d0_func,
                                         expr_d1=w_S_d1_str, func_d1=w_S_d1_func,
                                         expr_d2=w_S_d2_str, func_d2=w_S_d2_func,
                                         var_names=w_variables_names,
                                         var_types=w_variables_types,
                                         option='func_to_all')
            
            w_V_d0_str = 'f_V_0*(1-2*abs(x)/(xmax-xmin))'
            w_V_d1_str = 'f_V_0*(-2*sign(x)/(xmax-xmin))'
            w_V_d2_str = '0'
            w_V_d0_func=FSE_SF_func_d0
            w_V_d1_func=FSE_SF_func_d1
            w_V_d2_func=FSE_SF_func_d2
            self.w_V=Bend_func_cohort(name='V')
            Bend_func_cohort.Init_fandds(self.w_V,
                                         expr_d0=w_V_d0_str, func_d0=w_V_d0_func,
                                         expr_d1=w_V_d1_str, func_d1=w_V_d1_func,
                                         expr_d2=w_V_d2_str, func_d2=w_V_d2_func,
                                         var_names=w_variables_names,
                                         var_types=w_variables_types,
                                         option='func_to_all')
    
            w_Mima_d0_exp = self.w_S.d0.sy_expr - self.w_V.d0.sy_expr
            w_Mima_d1_exp = self.w_S.d1.sy_expr - self.w_V.d1.sy_expr
            w_Mima_d2_exp = self.w_S.d2.sy_expr - self.w_V.d2.sy_expr
            self.w_M_ima=Bend_func_cohort(name='M imaginary')
            Bend_func_cohort.Init_fandds(self.w_M_ima, expr_d0=w_Mima_d0_exp,
                                     expr_d1=w_Mima_d1_exp, expr_d2=w_Mima_d2_exp,
                                     var_names=w_variables_names,
                                     var_types=w_variables_types,
                                     option='each_expr_to_all')
            
            w_M_d0_str = 'b1*sin(FP*(x-xmin))+b2*sin(2*FP*(x-xmin))+b3*sin(3*FP*(x-xmin))+b4*sin(4*FP*(x-xmin))'
            w_M_d1_str = 'FP*(b1*cos(FP*(x - xmin)) + 2*b2*cos(2*FP*(x - xmin)) + 3*b3*cos(3*FP*(x - xmin)) + 4*b4*cos(4*FP*(x - xmin)))'
            w_M_d2_str = '-FP**2*(b1*sin(FP*(x - xmin)) + 4*b2*sin(2*FP*(x - xmin)) + 9*b3*sin(3*FP*(x - xmin)) + 16*b4*sin(4*FP*(x - xmin)))'
            w_M_d0_func=FSE_4sin_d0
            w_M_d1_func=FSE_4sin_d1
            w_M_d2_func=FSE_4sin_d2
            w_variables_names_M=np.array(['x','xmin','xmax','FP','b1','b2','b3','b4'])
            w_variables_types_M=dict({'x':'independent',
                                      'xmin':'fixed','xmax':'fixed',
                                      'FP':'expr'})
            self.w_M=Bend_func_cohort(name='M')
            Bend_func_cohort.Init_fandds(self.w_M,
                                         expr_d0=w_M_d0_str, func_d0=w_M_d0_func,
                                         expr_d1=w_M_d1_str, func_d1=w_M_d1_func,
                                         expr_d2=w_M_d2_str, func_d2=w_M_d2_func,
                                         var_names=w_variables_names_M,
                                         var_types=w_variables_types_M,
                                         option='func_to_all')
        elif option=='P4O':
            self.description='Bend line as 4th order polynom'
            
            w_A_d0_str='a*x**4+b*x**3+c*x**2+d*x+e'
            w_variables_names=np.array(['x','xmin','xmax','a','b','c','d','e','f_V_0'])
            self.w_A=Bend_func_cohort(name='A')
            Bend_func_cohort.Init_fandds(self.w_A, expr_d0=w_A_d0_str,
                                     var_names=w_variables_names,
                                     option='d0_str_to_all')
            
            w_I_d0_str = '((a*(xmax)**4+b*(xmax)**3+c*(xmax)**2+d*(xmax)+e)-(a*(xmin)**4+b*(xmin)**3+c*(xmin)**2+d*(xmin)+e))/(xmax-xmin)*x+((a*(xmax)**4+b*(xmax)**3+c*(xmax)**2+d*(xmax)+e)+(a*(xmin)**4+b*(xmin)**3+c*(xmin)**2+d*(xmin)+e))/2'
            self.w_I=Bend_func_cohort(name='I')
            Bend_func_cohort.Init_fandds(self.w_I, expr_d0=w_I_d0_str,
                                     var_names=w_variables_names,
                                     option='d0_str_to_all')
            
            w_S_d0_exp = self.w_A.d0.sy_expr - self.w_I.d0.sy_expr
            self.w_S=Bend_func_cohort(name='S')
            Bend_func_cohort.Init_fandds(self.w_S, expr_d0=w_S_d0_exp,
                                     var_names=w_variables_names,
                                     option='d0_expr_to_all')
            
            # w_variables_names_V=np.append(w_variables_names,'f_V_0')
            w_V_d0_str = 'f_V_0*(1-2*abs(x)/(xmax-xmin))'
            w_V_d1_str = 'f_V_0*(-2*sign(x)/(xmax-xmin))'
            w_V_d2_str = '0'
            self.w_V=Bend_func_cohort(name='V')
            Bend_func_cohort.Init_fandds(self.w_V, expr_d0=w_V_d0_str,
                                     expr_d1=w_V_d1_str, expr_d2=w_V_d2_str,
                                     var_names=w_variables_names,
                                     option='each_str_to_all')
    
            w_Mima_d0_exp = self.w_S.d0.sy_expr - self.w_V.d0.sy_expr
            w_Mima_d1_exp = self.w_S.d1.sy_expr - self.w_V.d1.sy_expr
            w_Mima_d2_exp = self.w_S.d2.sy_expr - self.w_V.d2.sy_expr
            self.w_M_ima=Bend_func_cohort(name='M imaginary')
            Bend_func_cohort.Init_fandds(self.w_M_ima, expr_d0=w_Mima_d0_exp,
                                     expr_d1=w_Mima_d1_exp, expr_d2=w_Mima_d2_exp,
                                     var_names=w_variables_names,
                                     option='each_expr_to_all')
            
            w_M_d0_str = 'a*x**4+b*x**3+c*x**2+d*x+e'
            w_variables_names_M = np.array(['x','xmin','xmax','a','b','c','d','e'])
            self.w_M=Bend_func_cohort(name='M')
            Bend_func_cohort.Init_fandds(self.w_M, expr_d0=w_M_d0_str,
                                     var_names=w_variables_names_M,
                                     option='d0_str_to_all')
        
        else:
            raise NotImplementedError("Option %s not implemented!"%option)

# def save_dill(self,filename):
    #     """
    #     Save builded Bend_func legion
    #     Does not work!!!
        
    #     """
    #     dill.dump(self, open(filename, "wb"))
    #     raise NotImplementedError("Not implemented successfully!")
        
    # def load_dill(self,filename):
    #     """
    #     Load builded Bend_func legion
    #     Does not work!!!
        
    #     """
    #     dill.load(self, open(filename, "rb"))
    #     raise NotImplementedError("Not implemented successfully!")
```

---

© Copyright 2024, MarcGebhardt.

Built with Sphinx using a
theme
provided by Read the Docs.
