# Supplementary material for: Approach to Standardized Material Characterization of the Human Lumbopelvic System: Testing and Evaluation
Source: Bioengineering (Basel). 2025 Aug 11;12(8):862. doi: 10.3390/bioengineering12080862 (PMC12383908; doi:10.3390/bioengineering12080862)
Supplement: Supplementary file 1 [file bioengineering-12-00862-s001.zip › File S3 Evaluation code/ExMechEva-0.1.2/docs/_build/html/_modules/exmecheva/bending/bfunc_com.html]

exmecheva.bending.bfunc\_com — ExMechEva 0.1.0 documentation


ExMechEva

Contents:

- ExMechEva

ExMechEva

- Module code
- exmecheva.bending.bfunc\_com

---

# Source code for exmecheva.bending.bfunc\_com

```
# -*- coding: utf-8 -*-
"""
General functions for bending.

@author: MarcGebhardt
"""
import numpy as np

#%% triangle function

[docs]def triangle_func_d0(x, xmin,xmax, f_0):
    """Standard triangle function with maximum in middle between min. and max. x coordinate."""
    eq=f_0*(1-2*abs(x)/(xmax-xmin))
    return eq


[docs]def triangle_func_d1(x, xmin,xmax, f_0):
    """First derivate of standard triangle function."""
    eq=f_0*(-2*np.sign(x)/(xmax-xmin))
    return eq


[docs]def triangle_func_d2(x, xmin,xmax, f_0):
    """Second derivate of standard triangle function (equal 0)."""
    eq=0
    return eq

#%% shear deformation

[docs]def Shear_area(Area, CS_type='Rectangle', kappa=None):
    """
    Returns the shear area of a cross section.

    Parameters
    ----------
    Area : float or function or 1
        Cross section area.
    CS_type : string, optional
        Cross section type. The default is 'Rectangle'.
    kappa : float, optional
         Correction factor shear area. Depending to CS_type. The default is None.

    Returns
    -------
    AreaS : same type as Area
        Shear area.

    """
    if  CS_type=='Rectangle': kappa = 5/6
    AreaS = Area * kappa
    return AreaS


[docs]def gamma_V_det(poisson, t_mean, Length, CS_type='Rectangle', kappa=None):
    """
    Returns the ratio between shear to entire deformation in mid of bending beam.

    Parameters
    ----------
    poisson : float
        Poisson's ratio.
    t_mean : float
        Mean thickness.
    Length : float
        Distance between load bearings.
    CS_type : string, optional
        Cross section type. The default is 'Rectangle'.
    kappa : float, optional
        Correction factor shear area. Depending to CS_type. The default is None.

    Returns
    -------
    gamma_V : float
        Ratio between shear to entire deformation in mid of bending beam.

    """
    kappa = Shear_area(1, CS_type, kappa)
    
    gamma_V = 2 / kappa * (1 + poisson) * t_mean**2 / Length**2
    return gamma_V
```

---

© Copyright 2024, MarcGebhardt.

Built with Sphinx using a
theme
provided by Read the Docs.
