# Supplementary material for: Approach to Standardized Material Characterization of the Human Lumbopelvic System: Testing and Evaluation
Source: Bioengineering (Basel). 2025 Aug 11;12(8):862. doi: 10.3390/bioengineering12080862 (PMC12383908; doi:10.3390/bioengineering12080862)
Supplement: Supplementary file 1 [file bioengineering-12-00862-s001.zip › File S3 Evaluation code/ExMechEva-0.1.2/docs/_build/html/_modules/exmecheva/bending/bfunc_fse.html]

exmecheva.bending.bfunc\_fse — ExMechEva 0.1.0 documentation


ExMechEva

Contents:

- ExMechEva

ExMechEva

- Module code
- exmecheva.bending.bfunc\_fse

---

# Source code for exmecheva.bending.bfunc\_fse

```
# -*- coding: utf-8 -*-
"""
Specific fourier series expansion functions for bending.

@author: MarcGebhardt
"""
import numpy as np

from .bfunc_com import (triangle_func_d0, triangle_func_d1, triangle_func_d2)

#%% Fourier series expansion with linear and constant element

[docs]def FSE_4sin_wlin_d0(x, xmin,xmax,FP, b1,b2,b3,b4,c,d, f_V_0=None):
    """
    Fourier series expansion with four sine elements and additional linear and constant element.
    Defined as:
 
    .. math::
     
        f(x; b1,b2,b3,b4,b5,c,d) = b1 \sin (\pi / (xmax-xmin) (x-xmin)) + b2 \sin (2 \pi / (xmax-xmin) (x-xmin)) + b3 \sin (3 \pi / (xmax-xmin) (x-xmin)) + b4 \sin (4 \pi / (xmax-xmin) (x-xmin)) +c (x-xmin) + d

    with `sine-parameters` for :math:`b1,b2,b3,b4`, `slope` for :math:`c` and `intercept` for :math:`d`.

    Parameters
    ----------
    x : array of float
        Array of cartesian coordinates in x-direction.
    xmin : float
        Cartesian coordinate in x-direction of start of function (curvature=0, p.e. left bearing).
        Defines length of Fourier series expansion with xmax-xmin.
    xmax : float
        Cartesian coordinate in x-direction of end of function (curvature=0, p.e. right bearing).
        Defines length of Fourier series expansion with xmax-xmin.
    b1 : float
        Parameter of first sine element.
    b2 : float
        Parameter of second sine element.
    b3 : float
        Parameter of third sine element.
    b4 : float
        Parameter of fourth sine element.
    c : float
        Parameter of slope.
    d : float
        Parameter of intercept.

    Returns
    -------
    eq : Function
        Fourier series expansion with four sine elements and additional linear and constant element.

    """
    eq=b1*np.sin(FP*(x-xmin))+b2*np.sin(2*FP*(x-xmin))+b3*np.sin(3*FP*(x-xmin))+b4*np.sin(4*FP*(x-xmin))+c*(x-xmin)+d
    return eq


[docs]def FSE_4sin_wlin_d1(x, xmin,xmax,FP, b1,b2,b3,b4,c,d=None, f_V_0=None):
    """First derivate of fourier series expansion with four sine elements and 
    additional linear and constant element. For more information see FSE_4sin_wlin_d0."""
    eq=FP*(b1*np.cos(FP*(x-xmin))+2*b2*np.cos(2*FP*(x-xmin))+3*b3*np.cos(3*FP*(x-xmin))+4*b4*np.cos(4*FP*(x-xmin)))+c
    return eq


[docs]def FSE_4sin_wlin_d2(x, xmin,xmax,FP, b1,b2,b3,b4,c=None,d=None, f_V_0=None):
    """Second derivate of fourier series expansion with four sine elements and 
    additional linear and constant element. For more information see FSE_4sin_wlin_d0."""
    eq=(-FP**2)*(b1*np.sin(FP*(x-xmin))+4*b2*np.sin(2*FP*(x-xmin))+9*b3*np.sin(3*FP*(x-xmin))+16*b4*np.sin(4*FP*(x-xmin)))
    return eq

#%% Linear and constant part only

[docs]def FSE_4sin_lin_func_d0(x, c,d, 
                         xmin=None,xmax=None,FP=None, b1=None,b2=None,b3=None,b4=None,
                         f_V_0=None):
    """Linear part of fourier series expansion with four sine elements and 
    additional linear and constant element. For more information see FSE_4sin_wlin_d0."""
    eq=c*(x-xmin)+d
    return eq


[docs]def FSE_4sin_lin_func_d1(x, c,d=None, 
                         xmin=None,xmax=None,FP=None, b1=None,b2=None,b3=None,b4=None,
                         f_V_0=None):
    """First deriavate of linear part of fourier series expansion with four sine elements and 
    additional linear and constant element. For more information see FSE_4sin_wlin_d0."""
    eq=c
    return eq


[docs]def FSE_4sin_lin_func_d2(x, c=None,d=None, 
                         xmin=None,xmax=None,FP=None, b1=None,b2=None,b3=None,b4=None,
                         f_V_0=None):
    """Second deriavate of linear part of fourier series expansion with four sine elements and 
    additional linear and constant element. For more information see FSE_4sin_wlin_d0. (Equal 0)"""
    eq=0
    return eq

#%% Fourier series expansion (without linear and constant element)

[docs]def FSE_4sin_d0(x, xmin,xmax,FP, b1,b2,b3,b4,c=None,d=None, f_V_0=None):
    """Fourier series expansion with four sine elements without additional linear 
    and constant element. For more information see FSE_4sin_wlin_d0."""
    eq=b1*np.sin(FP*(x-xmin))+b2*np.sin(2*FP*(x-xmin))+b3*np.sin(3*FP*(x-xmin))+b4*np.sin(4*FP*(x-xmin))
    return eq


[docs]def FSE_4sin_d1(x, xmin,xmax,FP, b1,b2,b3,b4,c=None,d=None, f_V_0=None):
    """First deriavate of Fourier series expansion with four sine elements 
    without additional linear and constant element. For more information see FSE_4sin_wlin_d0."""
    eq=(FP)*(b1*np.cos(FP*(x-xmin))+2*b2*np.cos(2*FP*(x-xmin))+3*b3*np.cos(3*FP*(x-xmin))+4*b4*np.cos(4*FP*(x-xmin)))
    return eq


[docs]def FSE_4sin_d2(x, xmin,xmax,FP, b1,b2,b3,b4,c=None,d=None, f_V_0=None):
    """Second deriavate of Fourier series expansion with four sine elements 
    without additional linear and constant element. For more information see FSE_4sin_wlin_d0."""
    eq=(-FP**2)*(b1*np.sin(FP*(x-xmin))+4*b2*np.sin(2*FP*(x-xmin))+9*b3*np.sin(3*FP*(x-xmin))+16*b4*np.sin(4*FP*(x-xmin)))
    return eq

#%% Shear function (triangle)

[docs]def FSE_SF_func_d0(x, xmin,xmax, f_V_0,
                   FP=None, b1=None,b2=None,b3=None,b4=None,c=None,d=None, opt=None):
    """Shear deformation part of ourier series expansion with four sine elements 
    and additional linear and constant element. Children of triangle_func_d0.
    For more information see FSE_4sin_wlin_d0."""
    eq=triangle_func_d0(x=x, xmin=xmin,xmax=xmax, f_0=f_V_0)
    return eq


[docs]def FSE_SF_func_d1(x, xmin,xmax, f_V_0,
                   FP=None, b1=None,b2=None,b3=None,b4=None,c=None,d=None, opt=None):
    """First derivate of shear deformation part of ourier series expansion with
    four sine elements and additional linear and constant element. 
    Children of triangle_func_d1. For more information see FSE_4sin_wlin_d0."""
    eq=triangle_func_d1(x=x, xmin=xmin,xmax=xmax, f_0=f_V_0)
    return eq


[docs]def FSE_SF_func_d2(x, xmin,xmax, f_V_0,
                   FP=None, b1=None,b2=None,b3=None,b4=None,c=None,d=None, opt=None):
    """Second derivate of shear deformation part of ourier series expansion with
    four sine elements and additional linear and constant element. 
    Children of triangle_func_d1. For more information see FSE_4sin_wlin_d0."""
    eq=triangle_func_d2(x=x, xmin=xmin,xmax=xmax, f_0=f_V_0)
    return eq
```

---

© Copyright 2024, MarcGebhardt.

Built with Sphinx using a
theme
provided by Read the Docs.
