# Supplementary material for: Approach to Standardized Material Characterization of the Human Lumbopelvic System: Testing and Evaluation
Source: Bioengineering (Basel). 2025 Aug 11;12(8):862. doi: 10.3390/bioengineering12080862 (PMC12383908; doi:10.3390/bioengineering12080862)
Supplement: Supplementary file 1 [file bioengineering-12-00862-s001.zip › File S3 Evaluation code/ExMechEva-0.1.2/docs/_build/html/_modules/exmecheva/bending/evaluation.html]

exmecheva.bending.evaluation — ExMechEva 0.1.0 documentation


ExMechEva

Contents:

- ExMechEva

ExMechEva

- Module code
- exmecheva.bending.evaluation

---

# Source code for exmecheva.bending.evaluation

```
# -*- coding: utf-8 -*-
"""
Evaluation functionality for bending.

@author: MarcGebhardt
"""
import numpy as np
import pandas as pd
import scipy.integrate as scint
import lmfit
from tqdm import tqdm

import exmecheva.common as emec
from .opt_mps import (Point_df_idx)
from .bfunc_com import (triangle_func_d0, Shear_area)

from .attr_bgl import (_significant_digits_fpd)

#%% Stress and strain

[docs]def straindf_from_curve(x, func_curve, params_curve, func_thick, evopt=1/2):
    """Evaluates the combination of a curvature and thickness function to x
    on a thickness ratio. (Use only for bending)
    Returns a dataframe with steps as index and x as columns."""
    df_strain = func_curve(
        x, params_curve,
        coords=None, coords_set=None, 
        col_type='val'
        )*func_thick(x)*evopt
    return df_strain


[docs]def Moment_perF_func(x, xmin,xmax, Test_type='TPB'):
    """Returns a by force scaleable moment function."""
    if Test_type == 'TPB':
        MpF_0=(xmax-xmin)/4
        eq=triangle_func_d0(x=x, xmin=xmin,xmax=xmax, f_0=MpF_0)
    else:
        raise NotImplementedError("Test-type %s not implemented!"%Test_type)
    return eq


[docs]def stress_perF(x, func_MoI, func_thick, xmin,xmax, evopt=1/2, Test_type='TPB'):
    """Returns a by force scaleable bending stress function."""
    stress_pF_data = (lambda b: Moment_perF_func(
        b, xmin,xmax, Test_type
        )*func_thick(b)*evopt/func_MoI(b))(x)
    if isinstance(x,float):
        return stress_pF_data
    else:
        # round float point division
        ind=pd.Index(x).map(lambda x: emec.helper.round_to_sigdig(
            x, _significant_digits_fpd
            ))
        stress_pF = pd.Series(stress_pF_data,index=ind)
        return stress_pF


[docs]def stress_df_from_lin(F, x, func_MoI, func_thick, xmin,xmax, evopt=1/2, 
                       Test_type='TPB'):
    """Returns bending stress values according given x coordinates."""
    stress_pF = stress_perF(x, func_MoI, func_thick, 
                            xmin, xmax, evopt, Test_type)
    df_stress = pd.DataFrame(stress_pF.values * F.values[:, None],
                             index=F.index, columns=stress_pF.index)
    return df_stress

#%% Weighting

[docs]def Weight_func(x, option='Triangle', c_func=None, **kwargs):
    """
    Returns a weighting function by given options and parameters.

    Parameters
    ----------
    x : float
        Coordinate in x direction.
    option : string, optional
        Choosen option. 
        Possible are:
            - 'Cut': Excluding values outside range of xmin to xmax 
                    (weight equal 0).
            - 'Triangle': Weighing to triangle function with maximum in the 
                    middle between xmin and xmax.
            - 'Triangle_cut': Mixture of 'Triangle' and 'Cut'.
            - 'Custom': Weighing to custom function (p.e. displacement funtion). 
            - 'Custom_cut': Mixture of 'Custom' and 'Cut'.
        The default is 'Triangle'.
    c_func : function, optional
        Custom function for weighing (p.e. displacement funtion). 
        The default is None.
    **kwargs : dict
        Keyword arguments for custom function (p.e. displacement 
                                               function parameters).

    Raises
    ------
    NotImplementedError
        Option not implemented.

    Returns
    -------
    eq : float
        Weights.

    """
    # if option == 'Triangle':
    #     f = 2/(kwargs['xmax']-kwargs['xmin'])
    #     f = 1
    #     eq = triangle_func_d0(x=x, xmin=kwargs['xmin'], xmax=kwargs['xmax'], f_0=f)
    if option == 'Cut':
        eq = np.where((x>=kwargs['xmin'])&(x<=kwargs['xmax']),1.0,0.0)
    elif option in ['Triangle','Triangle_cut']:
        # f = 2/(kwargs['xmax']-kwargs['xmin'])
        f = 1
        eq = triangle_func_d0(x=x, xmin=kwargs['xmin'], xmax=kwargs['xmax'], f_0=f)
        if option == 'Triangle_cut':
            eq = np.where((x>=kwargs['xmin'])&(x<=kwargs['xmax']),eq,0.0)
    elif option in ['Custom','Custom_cut']:
        eq = c_func(x, kwargs)
        if option == 'Custom_cut':
            eq = np.where((x>=kwargs['xmin'])&(x<=kwargs['xmax']),eq,0.0)
    else:
        raise NotImplementedError("Option %s not implemented!"%option)
    return eq

#%% Elastic modulus evaluation functions

[docs]def YM_eva_method_A(stress_mid_ser,
                    strain_mid_ser,
                    comp=True, name='A', 
                    det_opt='incremental',**kws):
    """
    Calculates Young's Modulus by rise of stress to strain in midspan 
    over defined range with definable method.
    Children of Evac.YM_eva_com_sel.

    Parameters
    ----------
    stress_mid_ser : pd.Series
        Series with stress values in midspan corresponding strain_ser.
    strain_mid_ser : pd.Series
        Series with strain values in midspan corresponding stress_ser.
    comp : boolean, optional
        Compression mode. The default is True.
    name : string, optional
        Name of operation. The default is 'A'.
    det_opt : TYPE, optional
        Definable method for determination.
        Ether incremental or leastsq. The default is 'incremental'.
    **kws : dict
        Keyword dict for least-square determination.

    Returns
    -------
    det_opt == "incremental":
        YM_ser : pd.Series
            Series of Young's Moduli.
    or
    det_opt == "leastsq":
        YM : float
            Youngs Modulus (corresponds to slope of linear fit).
        YM_abs : float
            Stress value on strain origin (corresponds to interception of linear fit).
        YM_Rquad : float
            Coefficient of determination.
        YM_fit : lmfit.model.ModelResult
            Fitting result from lmfit (use with fit.fit_report() for report).
    """
    YM = emec.fitting.YM_eva_com_sel(stress_ser=stress_mid_ser,
                                   strain_ser=strain_mid_ser,
                                   comp=comp, name=name,
                                   det_opt=det_opt, **kws)
    return YM


[docs]def YM_eva_method_B(stress_mid_ser,
                    thickness, Length, option="Points", 
                    P_df=None, P_fork_names=None,
                    w_func=None, w_params=None, Length_det=None,
                    comp=True, name='B', det_opt='incremental',**kws):
    """
    Calculates Young's Modulus by rise of stress to strain, 
    calculated by optical counterpart to traditional fork transducer,
    over defined range with definable method.

    Parameters
    ----------
    stress_mid_ser : pandas.Series
        Series with stress values in midspan corresponding strain_ser.
    thickness : np.poly1d function
        Function of thickness to span.
    Length : float
        Length of span.
    option : string, optional
        Input option, ether "Points", for a Dataframe of three points, or
        "Fit", for a fitted bend line.
        The default is "Points".
    P_df : pandas.DataFrame, optional
        Dataframe of Points. The default is None.
    P_fork_names : array of string, optional
        Names of points in P_df in form of [left, mid, right]. 
        The default is None.
    w_func : function, optional
        Function of bending line. The default is None.
    w_params : array or pandas.Series, optional
        Parameters of function of bending line per step. The default is None.
    Length_det : float, optional
        Determination length between left and right point to calculate. 
        The default is None.
    comp : boolean, optional
        Compression mode.The default is True.
    name : string, optional
        Name of operation. The default is 'B'.
    det_opt : TYPE, optional
        Definable method for determination.
        Ether incremental or leastsq. The default is 'incremental'.
    **kws : dict
        Keyword dict for least-square determination.

    Raises
    ------
    ValueError
        Error in combination of option and inputs.
    NotImplementedError
        Error if option not implemented.
        
    Returns
    -------
    det_opt == "incremental":
        YM_ser : pd.Series
            Series of Young's Moduli.
    or
    det_opt == "leastsq":
        YM : float
            Youngs Modulus (corresponds to slope of linear fit).
        YM_abs : float
            Stress value on strain origin (corresponds to interception of linear fit).
        YM_Rquad : float
            Coefficient of determination.
        YM_fit : lmfit.model.ModelResult
            Fitting result from lmfit (use with fit.fit_report() for report).
    """
    if option == "Points":
        if (P_df is None) or (P_fork_names is None) or len(P_fork_names)!=3:
            raise ValueError("Please insert correct values for P_df and P_fork_names!")
        # step_range=emec.pd_ext.pd_combine_index(stress_mid_ser, P_df)
        step_range=emec.pd_ext.pd_combine_index(stress_mid_ser, P_df.index)
        f_lr = Point_df_idx(P_df, steps=step_range,
                            points=[P_fork_names[0],P_fork_names[-1]],
                               coords=['y']).mean(axis=1)
        f_c  = Point_df_idx(P_df, steps=step_range,
                            points=[P_fork_names[1]],
                            coords=['y']).mean(axis=1)
        x_lr = Point_df_idx(P_df, steps=step_range,
                            points=[P_fork_names[0],P_fork_names[-1]],
                            coords=['x'])
        Length_det = (x_lr[P_fork_names[-1]]-x_lr[P_fork_names[0]])['x']
    elif option == "Fit":
        if (w_func is None) or (w_params is None) or (Length_det is None):
            raise ValueError("Please insert correct values w_func, w_params and Length_det!")
        step_range=emec.pd_ext.pd_combine_index(stress_mid_ser, w_params)
        f_lr = w_func(np.array([-Length_det/2, Length_det/2]),
                      w_params.loc[step_range]).mean(axis=1)
        f_c = w_func(0.0, w_params.loc[step_range])
    else:
        raise NotImplementedError("Option %s not implemented!"%option)
        
    f = (f_c - f_lr) * (-1 if comp else 1)
    stress_mid_ser = stress_mid_ser.loc[step_range]
    strain_ser = (12 * f * thickness * Length) / (3 * Length * Length_det**2 - Length_det**3)
    strain_ser = pd.Series(strain_ser, name=name)
    if det_opt=="incremental":
        YM_ser = stress_mid_ser / strain_ser
        YM_ser = pd.Series(YM_ser, name=name)
        return YM_ser, strain_ser
    elif det_opt=="leastsq":
        if stress_mid_ser.isna().all() or strain_ser.isna().all():
            YM, YM_abs, YM_Rquad, YM_fit = np.nan,np.nan,np.nan,np.nan
        else:
            YM, YM_abs, YM_Rquad, YM_fit = emec.fitting.YM_sigeps_lin(
                stress_mid_ser, strain_ser, **kws
                )
        return YM, YM_abs, YM_Rquad, YM_fit, strain_ser


[docs]def YM_eva_method_E(Force_ser, length,
                    func_curve, params_curve,
                    func_MoI, func_thick, evopt=1/2,
                    opt_det='all', n=100,
                    opt_g='length', opt_g_lim=0.5,
                    weight_func=Weight_func,
                    wargs=[], wkwargs={}, name='E'):
    """
    Calculates Young's Modulus by rise of stress to strain, 
    calculated by curvature (2nd derivate) of bending line,
    per step with definable method.

    Parameters
    ----------
    Force_ser : pandas.Series
        Series of force increments.
    length : float
        Length of span.
    func_curve :  function
        Function of curvature (2nd derivate) of bending line. The default is None.
    params_curve : array or pandas.Series of dictionionaries
        Parameters of function of curvature (2nd derivate) of bending line per step..
    func_MoI : np.poly1d function
        Function of Moment of inertia to position on span (x-direction).
    func_thick : np.poly1d function
        Function of thickness to position on span (x-direction).
    evopt : float, optional
        Position according thickness for strain calculation. The default is 1/2.
    opt_det : string, optional
        Determination option.
        possible are:
            - 'all': All options evaluated.
            - 'stress' : Local evaluation on maximum stress.
            - 'strain' : Local evaluation on maximum strain.
            - 'range' : Global evaluation over range defined by opt_g.
        The default is 'all'.
    n : integer, optional
        Division of the length for calculation. The default is 100.
    opt_g : string, optional
        Option for global determination.
        possible are:
            - 'length': Averaging by range (opt_g_lim*length) around midspan.
            - 'strain': Averaging by range (strain<=opt_g_lim*strain_max).
            - 'Moment_weighted': Weighted averaging by moment function.
            - 'Custom_weighted': Weighted averaging by custom function.
        The default is 'length'.
    opt_g_lim : float, optional
        Limiter for opt_g. The default is 0.5.
    weight_func : function, optional
        Weighting function for global averaging. The default is Weight_func.
    wargs : array, optional
        Arguments for weighting function for global averaging. 
        The default is [].
    wkwargs : dictionary, optional
        Keyword arguments for weighting function for global averaging. 
        The default is {}.
    name : string, optional
        Name of operation. The default is 'E'.

    Raises
    ------
    NotImplementedError
        Raise error if option not implemented.

    Returns
    -------
    E_df : pandas.DataFrame
        Dataframe of determined Young's Moduli.
    sig_eps_df : pandas.DataFrame
        Dataframe of stress and strain values used in determination.
    E_to_x : TYPE
        Dataframe of determined Young's Moduli to span position (x-direction).
    stress_df : pandas.DataFrame
        Dataframe of stress values.
    strain_df : pandas.DataFrame
        Dataframe of strain values.
    E_to_x_g : pandas.DataFrame
        Dataframe of determined Young's Moduli to span position (x-direction) 
        for global averaging.

    """
            
    # 26.10.22 - unload (negative force increment) added
    def MaxMinboSer(po,oths,k='Max',axis=1):
        gax = emec.pd_ext.pd_axischange(axis)
        # po_sign   = po.apply(np.sign)
        oths_sign = oths.apply(np.sign)
        po_osign = po.mul(oths_sign,axis=gax)
        if k == 'max':
            out=po_osign.max(axis=axis).mul(oths_sign,axis=gax)
        elif k == 'idxmax':
            out=po_osign.idxmax(axis=axis)
        elif k == 'min':
            out=po_osign.min(axis=axis).mul(oths_sign,axis=gax)
        elif k == 'idxmin':
            out=po_osign.idxmin(axis=axis)
        else:
            raise NotImplementedError("Option %s not implemented!"%k)
        return out
    
    if not opt_det in ['all', 'stress', 'strain', 'range']:
        raise NotImplementedError("Option %s type not implemented!"%opt_det)
        
    step_range=emec.pd_ext.pd_combine_index(Force_ser, params_curve)
    xlin = np.linspace(-length/2,length/2,n+1)
    
    stress_df = stress_df_from_lin(F=Force_ser, x=xlin,
                                   func_MoI=func_MoI, func_thick=func_thick,
                                   xmin=-length/2, xmax=length/2)
    stress_df = stress_df.loc[step_range]
    strain_df = straindf_from_curve(xlin, func_curve, params_curve, func_thick, evopt)
    strain_df = strain_df.loc[step_range]
    
    E_to_x = stress_df / strain_df
    
    if opt_det in ['all', 'stress', 'strain']:
                  
        # stress_max   = pd.Series(stress_df.max(axis=1), name='stress_max')
        # stress_max_x = pd.Series(stress_df.idxmax(axis=1), name='stress_max_x')
        # 26.10.22 - unload (negative force increment) added
        stress_max   = pd.Series(MaxMinboSer(stress_df,Force_ser,'max',1),
                                 name='stress_max')
        stress_max_x = pd.Series(MaxMinboSer(stress_df,Force_ser,'idxmax',1),
                                 name='stress_max_x')
        
        # Krücke, muss besser über Direktindizierung gehen
        E_stress_max = pd.Series([],dtype='float64', name='E_stress_max')
        stress_max_strain = pd.Series([],dtype='float64', name='stress_max_strain')
        for step in step_range: #Krücke da Force ab 'S' 
            E_stress_max.loc[step]      = E_to_x.loc[step,stress_max_x.loc[step]]
            stress_max_strain.loc[step] = strain_df.loc[step,stress_max_x.loc[step]]
        # E_stress_max = pd.Series(E_stress_max, name='E_stress_max')
        E_stress_max = pd.Series(E_stress_max, name=name+'l')
            
    if opt_det in ['all', 'strain']:
    
        # strain_max   = pd.Series(strain_df.max(axis=1), name='strain_max')
        # strain_max_x = pd.Series(strain_df.idxmax(axis=1), name='strain_max_x')
        # 26.10.22 - unload (negative force increment) added
        strain_max   = pd.Series(MaxMinboSer(strain_df,Force_ser,'max',1),
                                 name='strain_max')
        strain_max_x = pd.Series(MaxMinboSer(strain_df,Force_ser,'idxmax',1),
                                 name='strain_max_x')
        
        # Krücke, muss besser über Direktindizierung gehen
        E_strain_max = pd.Series([],dtype='float64', name='E_strain_max')
        strain_max_stress = pd.Series([],dtype='float64', name='strain_max_stress')
        for step in step_range: #Krücke da Force ab 'S' 
            E_strain_max.loc[step]      = E_to_x.loc[step,strain_max_x.loc[step]]
            strain_max_stress.loc[step] = stress_df.loc[step,strain_max_x.loc[step]]
        # E_strain_max = pd.Series(E_strain_max, name='E_strain_max')
        E_strain_max = pd.Series(E_strain_max, name=name+'e')

    if opt_det in ['all', 'range']:
        if opt_g == 'length':
            cols = emec.pd_ext.pd_slice_index(
                E_to_x.columns,
                [xlin.min()*opt_g_lim, xlin.max()*opt_g_lim]
                )
            E_to_x_g = E_to_x.loc(axis=1)[cols]
            E_to_x_g_mean = E_to_x.loc[step_range,cols].mean(axis=1)
        elif opt_g == 'strain':
            test=pd.DataFrame([],columns=strain_df.columns)
            for step in strain_df.index:
                # test.loc[step]=strain_df.loc[step] >= opt_g_lim*strain_df.max(axis=1).loc[step]
                # 26.10.22 - unload (negative force increment) added
                if Force_ser[step]>=0:
                    test.loc[step]=strain_df.loc[step] >= opt_g_lim*strain_df.max(axis=1).loc[step]
                else:
                    test.loc[step]=strain_df.loc[step] <= opt_g_lim*strain_df.min(axis=1).loc[step]
            E_to_x_g = E_to_x.copy()[test]
            E_to_x_g_mean = E_to_x_g.mean(axis=1)
        elif opt_g == 'Moment_weighted':
            cols = emec.pd_ext.pd_slice_index(
                E_to_x.columns,
                [xlin.min()*opt_g_lim, xlin.max()*opt_g_lim]
                )
            E_to_x_g = E_to_x.loc(axis=1)[cols]
            kws={'xmin':cols.min(),'xmax':cols.max()}
            mask=np.isnan(E_to_x_g)
            weights=np.array([Weight_func(x=cols,option='Triangle',**kws), ] * E_to_x_g.shape[0], dtype=np.float64)
            mw = np.ma.MaskedArray(weights, mask=mask)
            ma = np.ma.MaskedArray(E_to_x_g, mask=mask)
            E_to_x_g_mean = np.ma.average(a=ma,axis=1,weights=mw).filled(np.nan)
            # E_to_x_g_mean = np.average(a=E_to_x_g, axis=1,
            #                            weights=Weight_func(x=cols,option='Triangle',**kws))
            E_to_x_g_mean = pd.Series(E_to_x_g_mean, index=E_to_x.index)
        elif opt_g == 'Custom_weighted':
            cols = emec.pd_ext.pd_slice_index(
                E_to_x.columns,
                [xlin.min()*opt_g_lim, xlin.max()*opt_g_lim]
                )
            E_to_x_g = E_to_x.loc(axis=1)[cols]
            mask=np.isnan(E_to_x_g)
            if (type(wkwargs) is pd.core.series.Series):
                weights=weight_func(E_to_x_g.columns,wkwargs.loc[step_range])
            elif (type(wkwargs) is dict):
                weights=np.array([weight_func(E_to_x_g.columns,**wkwargs), ] * E_to_x_g.shape[0], dtype=np.float64)
            else:
                raise NotImplementedError("Type %s of wkwargs not implemented!"%type(wkwargs))
            mw = np.ma.MaskedArray(weights, mask=mask)
            ma = np.ma.MaskedArray(E_to_x_g, mask=mask)
            E_to_x_g_mean = np.ma.average(a=ma,axis=1,weights=mw).filled(np.nan)
            E_to_x_g_mean = pd.Series(E_to_x_g_mean, index=E_to_x.index)
        else:
            raise NotImplementedError("Option-global %s type not implemented!"%opt_g)
        # E_to_x_g_mean = pd.Series(E_to_x_g_mean, name='E_global_' + opt_g)
        # E_to_x_g_mean = pd.Series(E_to_x_g_mean, name='E_global')
        E_to_x_g_mean = pd.Series(E_to_x_g_mean, name=name+'g')
    else:
        E_to_x_g=[]    
        
    if opt_det == 'all':   
        E_df = pd.DataFrame([E_stress_max,E_strain_max,E_to_x_g_mean]).T
        sig_eps_df = pd.DataFrame([stress_max,stress_max_x,stress_max_strain,
                                   strain_max,strain_max_x,strain_max_stress]).T
    elif opt_det == 'stress':   
        E_df = pd.DataFrame([E_stress_max]).T
        sig_eps_df = pd.DataFrame([stress_max,stress_max_x,stress_max_strain]).T 
    elif opt_det == 'strain':  
        E_df = pd.DataFrame([E_strain_max]).T
        sig_eps_df = pd.DataFrame([strain_max,strain_max_x,strain_max_stress]).T
    elif opt_det == 'range':   
        E_df = pd.DataFrame([E_to_x_g_mean]).T
        sig_eps_df = pd.DataFrame([]).T
        
    return E_df, sig_eps_df, E_to_x, stress_df, strain_df, E_to_x_g


[docs]def YM_eva_method_G(Force_ser,
                     w_func_f_0, w_params,
                     c_func, r_func,
                     c_params, r_params,
                     length, I_func, A_func=None,
                     CS_type='Rectangle', kappa=None, poisson=0.3,
                     comp=True, option="M+V", name="G"):
    """
    Calculates Young's Moduli via the approach of equality of external work 
    and deformation energy.

    Parameters
    ----------
    Force_ser : pandas.Series
        Series of force increments.
    w_func_f_0 : function
        Function of bending line for external work calculation.
    w_params : pandas.Series of dictionionaries
        Parameters of function of bending line per step for external 
        work calculation.
    c_func : function
        Function of curvature (2nd derivate) of bending line.
    r_func : function
        Function of rise (1st derivate) of bending line.
    c_params : pandas.Series of dictionionaries
        Parameters of function of curvature (2nd derivate) of bending 
        line per step.
    r_params : pandas.Series of dictionionaries
        Parameters of function of rise (1st derivate) of bending line per step.
    length : float
        Length of span.
    I_func : np.poly1d function
        Function of Moment of inertia to position on span (x-direction).
    A_func : np.poly1d function, optional
        Function of Area to position on span (x-direction). 
        The default is None.
    CS_type : string, optional
        Cross-section type. The default is 'Rectangle'.
    kappa : float, optional
         Correction factor shear area. Depending to CS_type. 
         The default is None.
    poisson : float, optional
        Poisson's ratio. The default is 0.3.
    comp : boolean, optional
        Compression mode.The default is True.
    option : string, optional
        Determiantion option. Possible are 'ignore_V' and 'M+V'. 
        The default is "M+V".
    name : string, optional
        Name of operation. The default is "G".

    Returns
    -------
    pandas.Series
        Series of determined Young's Moduli.

    """
    step_range = emec.pd_ext.pd_combine_index(Force_ser, w_params)
    
    def W_ext(Force_ser, f_0, comp=True):
        W_ext = (-1 if comp else 1) * 1/2 * Force_ser * f_0
        return W_ext
    f_0 = w_func_f_0(0.0, w_params.loc[step_range])
    Wext = W_ext(Force_ser.loc[step_range], f_0, comp)

    def W_int_integrant(w_func, w_params, CS_func):
        Wint_I_func = lambda s,x: w_func(x,w_params.loc[s])**2*CS_func(x)
        return Wint_I_func
    
    if option == "ignore_V":
        Wint_I = W_int_integrant(c_func, c_params, I_func)
        Wint = pd.Series([],dtype='float64',name=name)
        for step in step_range:
            Wint.loc[step]=1/2 * scint.quad(
                lambda x: Wint_I(x=x,s=step),-length/2,length/2)[0]
    elif option == "M+V":
        Wint_I_M = W_int_integrant(c_func, c_params, I_func)
        Wint_M = pd.Series([],dtype='float64',name=name)
        func_AS = Shear_area(A_func, CS_type, kappa)
        Wint_I_V = W_int_integrant(r_func, r_params, func_AS)
        Wint_V = pd.Series([],dtype='float64',name=name)
        for step in step_range:
            Wint_M.loc[step]=1/2 * scint.quad(
                lambda x: Wint_I_M(x=x,s=step),-length/2,length/2
                )[0]
            Wint_V.loc[step]=1/2 * 1/(2*(1+poisson))*scint.quad(
                lambda x: Wint_I_V(x=x,s=step),-length/2,length/2
                )[0]
        Wint=Wint_M + Wint_V
    E_ser = pd.Series(Wext/Wint,name=name)
    return E_ser


[docs]def YM_eva_method_F(c_func, c_params,
                    Force_ser,
                    Length, func_I,
                    weighted=True, weight_func=Weight_func,
                    wargs=[], wkwargs={},
                    xr_dict = {'fu':1/1, 'ha':1/2, 'th':1/3},
                    pb_b=True, name='F', n=100):
    """
    Calculates the Young's modulus via the local application of 
    the differential equation of the bending line.

    Parameters
    ----------
    c_func : function
        Function of curvature (2nd derivate) of bending line.
    c_params : pandas.Series of dictionionaries
        Parameters of function of curvature (2nd derivate) of bending line 
        per step.
    Force_ser : pandas.Series
        Series of force increments.
    Length : float
        Length of span.
    func_I : Tnp.poly1d function
        Function of Moment of inertia to position on span (x-direction).
    weighted : boolean, optional
        Switch for weighted averaging. The default is True.
    weight_func : function, optional
        Weighting function for global averaging. The default is Weight_func.
    wargs : array, optional
        Arguments for weighting function for global averaging. 
        The default is [].
    wkwargs : dictionary, optional
        Keyword arguments for weighting function for global averaging. 
        The default is {}.
    xr_dict : dictionary, optional
        Dictionary of name to range of length around midspan for determination.
        The default is {'fu':1/1, 'ha':1/2, 'th':1/3}.
    pb_b : boolean, optional
        Switch of progressbar. The default is True.
    name : string, optional
        Name of operation. The default is 'F'.
    n : integer, optional
        Division of the length for calculation. The default is 100.

    Returns
    -------
    YM_df : pandas.Series
        Series of determined Young's Moduli.

    """
    def E_YM(c_func, c_params, I_func, Length):
        YM = lambda s,x: Moment_perF_func(x, -Length/2, Length/2)*Force_ser.loc[s]/(c_func(x,c_params.loc[s])*I_func(x))
        return YM 
    
    step_range = emec.pd_ext.pd_combine_index(Force_ser, c_params)    
    if pb_b: pb = tqdm(step_range, desc =name+": ", unit=' steps', ncols=100)
    E_YM_func = E_YM(c_func, c_params, func_I, Length)
    
    YM_df = pd.DataFrame([],columns=xr_dict.keys(),dtype='float64')
    xlin  = pd.DataFrame([],columns=xr_dict.keys(),dtype='float64')
    for k in xr_dict:
        xlin[k] = np.linspace(-Length*xr_dict[k]/2,Length*xr_dict[k]/2,n+1)
    for step in step_range:
        YM_wei = pd.DataFrame([],columns=xr_dict.keys(),dtype='float64')
        YM_tmp = pd.DataFrame([],columns=xr_dict.keys(),dtype='float64')
        for k in xr_dict: 
            if weighted:
                if (type(wkwargs) is pd.core.series.Series):
                    YM_wei[k] = weight_func(xlin[k],*wargs,**wkwargs.loc[step])
                else:
                    YM_wei[k] = weight_func(xlin[k],*wargs,**wkwargs)
            else:
                # YM_wei[k] = None
                YM_wei[k] = np.array([1,]*xlin[k].shape[0])
            YM_tmp[k] = E_YM_func(s=step, x=xlin[k])
            ind_E       = np.where(np.logical_not(np.isnan(YM_tmp[k])))[0]
            YM_df.at[step,k] = np.average(YM_tmp.loc[ind_E,k],
                                          weights=YM_wei.loc[ind_E,k])
        if pb_b: pb.update()
    if pb_b: pb.close()
    # YM_df.columns=np.array([name+'_'+k for k in xr_dict.keys()])
    YM_df.columns=np.array([name+k for k in xr_dict.keys()])
    return YM_df


[docs]def YM_eva_method_C(Force_ser, w_func, w_params,
                     length, I_func, A_func=None,
                     CS_type='Rectangle', kappa=None, poisson=0.3,
                     comp=True, option="M+V", name="C"):
    """
    Calculates the Young's modulus from the deformation energy as a
    function of the internal forces (pure geometric fitting function).

    Parameters
    ----------
    Force_ser : pandas.Series
        Series of force increments.
    w_func : function
        Function of bending line.
    w_params : pandas.Series of dictionionaries
        Parameters of function  of bending line per step.
    length : float
        Length of span.
    I_func : np.poly1d function
        Function of Moment of inertia to position on span (x-direction).
    A_func : np.poly1d function, optional
        Function of Area to position on span (x-direction).
    CS_type : string, optional
        Cross-section type. The default is 'Rectangle'.
    kappa : float, optional
         Correction factor shear area. Depending to CS_type. 
         The default is None.
    poisson : float, optional
        Poisson's ratio. The default is 0.3.
    comp : boolean, optional
        Compression mode.The default is True.
    option : string, optional
        Determiantion option. Possible are 'M' and 'M+V'. The default is "M+V".
    name : string, optional
        Name of operation. The default is "F".

    Returns
    -------
    E_ser : pandas.Series
        Series of determined Young's Moduli.

    """
    step_range = emec.pd_ext.pd_combine_index(Force_ser, w_params)
    func_AS = Shear_area(A_func, CS_type, kappa)
    F_Alpha = length**2/8*scint.quad(lambda x: (1-abs(x*2/length))**2/I_func(x),-length/2,length/2)[0]
    if option == "M+V":
        F_Beta  = (1+poisson)*scint.quad(lambda x: 1/func_AS(x),-length/2,length/2)[0]
    else:
        F_Beta=0
    f_0   = (-1 if comp else 1)*(w_func(0.0,w_params.loc[step_range]))
    F_gamma = Force_ser.loc[step_range] / (2 * f_0)
    E_ser = pd.Series(F_gamma * (F_Alpha + F_Beta), name=name)
    return E_ser


[docs]def YM_eva_method_D_bend_df(Length, I_func, n=100, E=1, F=1):
    """
    Calculates the deflection values via the determined integral of the 
    bending line.

    Parameters
    ----------
    Length :  float
        Length of span.
    I_func : np.poly1d function
        Function of Moment of inertia to position on span (x-direction).
    n : integer, optional
        Division of the length for calculation. The default is 100.
    E : float, optional
        Young's Modulus. The default is 1.
    F : float, optional
        Force. The default is 1.

    Returns
    -------
    m : pandas.DataFrame
        Dataframe of deflections and parts of partwise integration to 
        x-positions.

    """
    def sidewise_df(x, Length, F, E , I_func, side='l'):
        if side == 'l':
            m_df=pd.DataFrame(data=None,index=-x)
        elif side == 'r':
            m_df=pd.DataFrame(data=None,index= x)
        else:
            raise NotImplementedError("Side %s not implemented, only l and r allowed!"%side)
        m_df['M'] = -F * Moment_perF_func(
            x=m_df.index, 
            xmin=-Length/2, xmax=Length/2, 
            Test_type='TPB'
            )
        m_df['I'] = I_func(m_df.index)
        m_df['Quotient'] = m_df.loc[:,'M'] / (E * m_df.loc[:,'I'])
        j = 0
        for i in m_df.index:
            if i==0:
                m_df.loc[i,'Integral_1'] = 0
                m_df.loc[i,'Integral_2'] = 0
                m_df.loc[i,'Omega']      = 0
                j = i
            else:
                # m_df.loc[i,'Integral_1'] = m_df.loc[j,'Integral_1'] + m_df.loc[[i,j],'Quotient'].mean() * Length/2/(n/2)
                # m_df.loc[i,'Integral_2'] = m_df.loc[j,'Integral_2'] + m_df.loc[[i,j],'Integral_1'].mean() * Length/2/(n/2)
                m_df.loc[i,'Integral_1'] = m_df.loc[j,'Integral_1'] + m_df.loc[[i,j],'Quotient'].mean() * abs((i-j))
                m_df.loc[i,'Integral_2'] = m_df.loc[j,'Integral_2'] + m_df.loc[[i,j],'Integral_1'].mean() * abs((i-j))
                m_df.loc[i,'Omega']      = -m_df.loc[i,'Integral_2']
                j = i
        return m_df
    
    x = pd.Float64Index(np.linspace(0,Length/2,int(n/2)+1),name='x')
    _significant_digits_fpd=12
    x=x.map(lambda x: emec.helper.round_to_sigdig(x, _significant_digits_fpd))
    m_l = sidewise_df(x, Length, F, E, I_func, side='l')
    m_r = sidewise_df(x, Length, F, E, I_func, side='r')
    
    RHS     = -np.array([m_l.iloc[-1].loc['Omega'], m_r.iloc[-1].loc['Omega']])        
    Kmat    = np.array([[1,-Length/2],[1,Length/2]])
    Kmatinv = np.linalg.inv(Kmat)
    mid     = Kmatinv.dot(RHS)
    
    m = m_l.iloc[1:].append(m_r).sort_index()
    m['w']   = mid[0] + mid[1] * m.index + m.loc[:,'Omega']
    m['wi']  = mid[1] - m.loc[:,'Integral_1']*np.sign(m.index)
    m['wii'] = -m.loc[:,'Quotient']
    return m


[docs]def YM_eva_method_D_bend_df_add(points_x, m_df, Length, I_func, E=1, F=1):
    """
    Adds additional points to the calculated deflection values via the
    determined integral of the bending line.

    Parameters
    ----------
    points_x : pd.Series
        X-coordinates of additional points.
    m_df : pandas.DataFrame
        Dataframe of deflections and parts of partwise integration to x-positions.
    Length : float
        Length of span.
    I_func : np.poly1d function
        Function of Moment of inertia to position on span (x-direction).
    E : float, optional
        Young's Modulus. The default is 1.
    F : float, optional
        Force. The default is 1.

    Returns
    -------
    p_df : pandas.DataFrame
        Dataframe of deflections and parts of partwise integration to x-positions.

    """
    x = pd.Float64Index(points_x,name='x')
    p_df=pd.DataFrame(data=None,index=x)
    
    p_df['M'] = -F * Moment_perF_func(
        x=x, xmin=-Length/2, xmax=Length/2, Test_type='TPB'
        )
    p_df['I'] = I_func(x)
    p_df['Quotient'] = p_df.loc[:,'M'] / (E * p_df.loc[:,'I'])
    for i in points_x:
        if i<0.0:
            method='bfill'
        else:
            method='ffill'
        j=m_df.index[m_df.index.get_loc(i,method=method)]
        if i==0.0:
            p_df.loc[i,'Integral_1'] = 0
            p_df.loc[i,'Integral_2'] = 0
            p_df.loc[i,'Omega']      = 0
        else:
            p_df.loc[i,'Integral_1'] = m_df.loc[j,'Integral_1'] + np.mean([m_df.loc[j,'Quotient'],p_df.loc[i,'Quotient']]) * abs(i-j)
            p_df.loc[i,'Integral_2'] = m_df.loc[j,'Integral_2'] + np.mean([m_df.loc[j,'Integral_1'],p_df.loc[i,'Integral_1']]) * abs(i-j)
            p_df.loc[i,'Omega']      = -p_df.loc[i,'Integral_2']
    
    p_df['w']   = m_df.loc[0.0,'w'] + m_df.loc[0.0,'wi'] * p_df.index + p_df.loc[:,'Omega']
    p_df['wi']  = m_df.loc[0.0,'wi'] - p_df.loc[:,'Integral_1']*np.sign(p_df.index)
    p_df['wii'] = -p_df.loc[:,'Quotient']
    return p_df


[docs]def YM_eva_method_D_res(E, x_data, y_data=None, weights=None, **kws):
    """Calculate residues of method D."""
    m_df=YM_eva_method_D_bend_df(
        kws['Length'], kws['I_func'], kws['n'], E, kws['F']
        )
    p_df=YM_eva_method_D_bend_df_add(
        x_data, m_df,kws['Length'], kws['I_func'], E, kws['F']
        )
    if y_data is None:
        err=p_df['w']
    elif weights is None:
        err=p_df['w']-y_data
    else:
        err=(p_df['w']-y_data)*weights
    return err


[docs]def YM_eva_method_D_num(P_df, Force_ser, step_range,
                        Length, func_I,
                        weighted=True, weight_func=Weight_func,
                        wargs=[], wkwargs={}, max_nfev=500,
                        pb_b=True, name='D'):
    """
    Evaluate elastic modulus by minimization to method D residuals.

    Parameters
    ----------
    P_df : pandas.DataFrame
        Dataframe of measured points to steps.
    Force_ser : pandas.Series
        Series of force increments.
    step_range : pd.Index or range or list
        Evaluation range.
    Length : float
        Length of span.
    func_I : np.poly1d function
        Function of Moment of inertia to position on span (x-direction).
    weighted : boolean, optional
        Switch for weighted averaging. The default is True.
    weight_func : function, optional
        Weighting function for global averaging. The default is Weight_func.
    wargs : array, optional
        Arguments for weighting function for global averaging. 
        The default is [].
    wkwargs : dictionary, optional
        Keyword arguments for weighting function for global averaging. 
        The default is {}.
    max_nfev : int, optional
        Maximum number of iterations. The default is 500.
    pb_b : boolean, optional
        Switch of progressbar. The default is True.
    name : string, optional
        Name of operation. The default is 'D'.

    Returns
    -------
    D_df : pd.DataFrame
        Evaluated elastic modulus and coefficent of determination per step.

    """
    D_df = pd.DataFrame([],columns=['E','Rquad'],dtype='float64')
    if pb_b: pb = tqdm(step_range, desc =name+": ", unit=' steps', ncols=100)
    for step in step_range:
        mo=lmfit.Model(YM_eva_method_D_res, independent_vars=['x_data'])
        mo.name='YM_eva_method_D'
        par = mo.make_params()
        par.add('E', value=500.0, min=0.0, max=np.inf)
        
        F=Force_ser.loc[step]
        x_data = P_df.loc[step].loc[:,'x'].values
        y_data = P_df.loc[step].loc[:,'y'].values
        if weighted:
            if (type(wkwargs) is pd.core.series.Series):
                weights = weight_func(x_data,*wargs,**wkwargs.loc[step])
            else:
                weights = weight_func(x_data,*wargs,**wkwargs)
        else:
            weights = None
        fit_Result = lmfit.minimize(
            YM_eva_method_D_res, par, args=(x_data,),
            kws={'I_func': func_I, 'n':100, 'Length': Length, 'F': F, 
                 'y_data': y_data, 'weights': weights},
            scale_covar=True, max_nfev=max_nfev
            )
        D_df.loc[step,'E'] = fit_Result.params['E'].value
        D_df.loc[step,'Rquad'] = 1 - fit_Result.residual.var() / np.var(y_data)
        if pb_b: pb.update()
    if pb_b: pb.close()
    return D_df


[docs]def YM_eva_method_D(P_df, Force_ser,
                    Length, func_I, n=100, rel_steps = None,
                    weighted=False, weight_func=Weight_func,
                    wkwargs={}, wargs=[], pb_b=True, name='D'):
    """
    Calculates the modulus of elasticity by matching the theoretical 
    displacements from the determined integral of the bending line with 
    the measured ones.

    Parameters
    ----------
    P_df : pandas.DataFrame
        Dataframe of measured points to steps.
    Force_ser : pandas.Series
        Series of force increments.
    Length : float
        Length of span.
    func_I : np.poly1d function
        Function of Moment of inertia to position on span (x-direction).
    n : integer, optional
        Division of the length for calculation. The default is 100.
    rel_steps : index or array, optional
        Relevant steps for determination. The default is None.
    weighted : boolean, optional
        Switch for weighted averaging. The default is True.
    weight_func : function, optional
        Weighting function for global averaging. The default is Weight_func.
    wargs : array, optional
        Arguments for weighting function for global averaging. 
        The default is [].
    wkwargs : dictionary, optional
        Keyword arguments for weighting function for global averaging. 
        The default is {}.
    pb_b : boolean, optional
        Switch of progressbar. The default is True.
    name : string, optional
        Name of operation. The default is 'D'.

    Returns
    -------
    YM_ser : pandas.Series
        Series of determined Young's Moduli.
    YM_df : pandas.DataFrame
        DataFrame of determined Young's Moduli to x-position.

    """
    m = YM_eva_method_D_bend_df(Length=Length, I_func=func_I, n=n, E=1, F=1)
    import warnings
    # ignore NaN-multiply
    warnings.filterwarnings('ignore',category=RuntimeWarning)
    
    step_range = emec.pd_ext.pd_combine_index(
        Force_ser, P_df.loc[np.invert(P_df.isna().all(axis=1))].index
        )
    
    if not rel_steps is None: 
        step_range = emec.pd_ext.pd_combine_index(rel_steps, step_range)
    
    if weighted:
        if (type(wkwargs) is pd.core.series.Series):
             step_range = emec.pd_ext.pd_combine_index(step_range,
                                                      wkwargs)
        
    YM_ser = pd.Series([],dtype='float64')
    YM_df = pd.DataFrame([],columns=P_df.columns.droplevel(1).drop_duplicates(),
                         dtype='float64')
    if pb_b: pb = tqdm(step_range, desc =name+": ", unit=' steps', ncols=100)
    for step in step_range:
        F = Force_ser.loc[step]
        x_data = P_df.loc[step].loc[:,'x']
        y_data = P_df.loc[step].loc[:,'y']
        p = YM_eva_method_D_bend_df_add(points_x=x_data.dropna(), m_df=m,
                                         Length=Length, I_func=func_I,
                                         E=1, F=1)
        D_gamma = F/y_data
        D_alpha = (x_data/Length-0.5)*m['Omega'].iloc[0]
        D_beta  = (x_data/Length+0.5)*m['Omega'].iloc[-1]
        D_omega = pd.Series([], dtype='float64')
        for i in x_data.index:
            if np.isnan(x_data[i]):
                D_omega[i] = np.nan
            else:
                D_omega[i] = p.loc[x_data[i],'Omega']
        YM_df.loc[step] = D_gamma*(D_alpha-D_beta+D_omega)
        if weighted:
            if (type(wkwargs) is pd.core.series.Series):
                weights = weight_func(x_data,*wargs,**wkwargs.loc[step])
            else:
                weights = weight_func(x_data,*wargs,**wkwargs)
            ind_E = np.invert((YM_df.loc[step].isna()) | (YM_df.loc[step] == 0))
            YM_ser.at[step] = np.average(YM_df.loc[step][ind_E],
                                         weights=weights[ind_E])
        else:
            weights = None
            ind_E = np.invert((YM_df.loc[step].isna()) | (YM_df.loc[step] == 0))
            YM_ser.at[step] = YM_df.loc[step][ind_E].mean()
        YM_ser =pd.Series(YM_ser,name=name)
        if pb_b: pb.update()
    if pb_b: pb.close()
    return YM_ser, YM_df

#%% Checking and documentation

[docs]def YM_check_with_method_D(E, F, Length, I_func, w_vgl_df, 
                           pb_b=True, name='X'):
    """
    Compares the deformation of the analytical bending line (method D), 
    scaled by elastic modulus determined by methods, with the measured bending 
    line.
    
    Parameters
    ----------
    E : float
        Elastic modulus.
    F : float
        Force (or force increment).
    Length : float
        Testing length.
    I_func : TYPE
        Function of Moment of Inertia.
    w_func : Bend_func_sub
        Bending function.
    w_params : pd.Series of dict
        Parameters per step for bending function (w_func).
    rel_steps : pandas.Index or numpy array, optional
        Relevant steps. The default is None.
    n : integer, optional
        Number of determination points. The default is 100.
    pb_b : boolean, optional
        Switch progress bar output. The default is True.
    name : string, optional
        Name of executed check. The default is 'X'.

    Returns
    -------
    check_E : pandas.Dataframe
        Deviation of scaled analytical and measured deformation.
    w_D_to_E : pandas.Dataframe
        Scaled analytical defeormation.

    """
    step_range = emec.pd_ext.pd_combine_index(E, w_vgl_df)
    m = YM_eva_method_D_bend_df(
        Length=w_vgl_df.columns.max()-w_vgl_df.columns.min(),
        I_func=I_func, n=len(w_vgl_df.columns)-1, E=1, F=1
        )
    D_gamma = F.loc[step_range]/E.loc[step_range]
    D_alpha = (m.index/Length-0.5)*m['Omega'].iloc[0]
    D_beta  = (m.index/Length+0.5)*m['Omega'].iloc[-1]
    D_sigma = (D_alpha-D_beta+m['Omega'])
    # w_D_to_E = D_gamma*(D_alpha-D_beta+m['Omega'])
    # w_D_to_E = pd.DataFrame([],index=D_gamma.index,columns=m.index,dtype='float64')
    # for step in D_gamma.index:
    #     w_D_to_E.loc[step] = D_gamma.loc[step]*D_sigma
    tmp = np.array([D_gamma.values,]*D_sigma.shape[0]).transpose()
    w_D_to_E = pd.DataFrame(tmp*D_sigma.values,
                            index=D_gamma.index,
                            columns=D_sigma.index)
    check_E = (w_D_to_E.loc[step_range]-w_vgl_df.loc[step_range])/w_vgl_df.loc[step_range]
    return check_E, w_D_to_E


[docs]def coord_df_mean(df, name='', fex=1,lex=1):
    """Return mean values."""
    ser_m=pd.Series(df.iloc(axis=1)[fex:-lex].mean(axis=1), name=name)
    return ser_m


[docs]def coord_df_depo(df, name='', pos=0.0):
    """Return values on postion (Default x=0.0)"""
    ser_m=pd.Series(df[pos], name=name)
    return ser_m


[docs]def YM_check_many_with_method_D(E_dict, F, Length, I_func,
                                 w_func, w_params, rel_steps=None, n=100,
                                 pb_b=True, name='X'):
    """
    Compares the deformation of the analytical bending line (method D), 
    scaled by elastic modulus determined by methods, with the measured bending 
    line.
    Returns global mean, local (midspan) mean and complete (n-positions) 
    deviation, as well as the scaled analytical bending line, for each 
    specified step.

    Parameters
    ----------
    E_dict : dict
        Dictionary of moduli of elasticity by determination method.
    F : pandas.Series
        Series of force (or force increment).
    Length : float
        Testing length.
    I_func : TYPE
        Function of Moment of Inertia.
    w_func : Bend_func_sub
        Bending function.
    w_params : pd.Series of dict
        Parameters per step for bending function (w_func).
    rel_steps : pandas.Index or numpy array, optional
        Relevant steps. The default is None.
    n : integer, optional
        Number of determination points. The default is 100.
    pb_b : boolean, optional
        Switch progress bar output. The default is True.
    name : string, optional
        Name of executed check. The default is 'X'.

    Returns
    -------
    check_EtoD_g : pandas.Dataframe
        Global (total length) mean deviation of scaled analytical and measured deformation.
    check_EtoD_x : pandas.Dataframe
        Local (midspan) mean deviation of scaled analytical and measured deformation.
    check_E : dictionary of pandas.Dataframe
        Deviation of scaled analytical and measured deformation.
    w_D_to_E : dictionary of pandas.Dataframe
        Scaled analytical defeormation by method.

    """
    if pb_b: pb = tqdm(range(len(E_dict.keys())), desc ="check "+name+": ",
                       unit=' method', ncols=100)
    step_range = emec.pd_ext.pd_combine_index(F, w_params)
    if not rel_steps is None: 
        step_range = emec.pd_ext.pd_combine_index(rel_steps, step_range)
    xlin=np.linspace(-Length/2,Length/2,n+1)
    w_vgl_df = w_func(xlin, w_params.loc[step_range],
                      coords=None,coords_set=None,col_type='val')
    m = YM_eva_method_D_bend_df(Length=Length, I_func=I_func, n=n, E=1, F=1)
    
    D_alpha = (m.index/Length-0.5)*m['Omega'].iloc[0]
    D_beta  = (m.index/Length+0.5)*m['Omega'].iloc[-1]
    D_sigma = (D_alpha-D_beta+m['Omega'])
    w_D_to_E={}
    check_E={}
    check_EtoD_g = {}
    check_EtoD_x = {}
    for E in E_dict:
        D_gamma = F.loc[step_range]/E_dict[E].loc[step_range]
        tmp = np.array([D_gamma.values,]*D_sigma.shape[0]).transpose()
        w_D_to_E[E] = pd.DataFrame(tmp*D_sigma.values,
                                   index=D_gamma.index,
                                   columns=D_sigma.index)
        check_E[E] = (w_D_to_E[E].loc[step_range]-w_vgl_df.loc[step_range])/w_vgl_df.loc[step_range]
        check_EtoD_g[E] = coord_df_mean(check_E[E], E)
        check_EtoD_x[E] = coord_df_depo(check_E[E], E)
        if pb_b: pb.update()
    if pb_b: pb.close()
    check_EtoD_g = pd.DataFrame.from_dict(check_EtoD_g)
    check_EtoD_x = pd.DataFrame.from_dict(check_EtoD_x)
    return check_EtoD_g, check_EtoD_x, check_E, w_D_to_E
```

---

© Copyright 2024, MarcGebhardt.

Built with Sphinx using a
theme
provided by Read the Docs.
