# Supplementary material for: Approach to Standardized Material Characterization of the Human Lumbopelvic System: Testing and Evaluation
Source: Bioengineering (Basel). 2025 Aug 11;12(8):862. doi: 10.3390/bioengineering12080862 (PMC12383908; doi:10.3390/bioengineering12080862)
Supplement: Supplementary file 1 [file bioengineering-12-00862-s001.zip › File S3 Evaluation code/ExMechEva-0.1.2/docs/_build/html/_modules/exmecheva/bending/fitting.html]

exmecheva.bending.fitting — ExMechEva 0.1.0 documentation


ExMechEva

Contents:

- ExMechEva

ExMechEva

- Module code
- exmecheva.bending.fitting

---

# Source code for exmecheva.bending.fitting

```
# -*- coding: utf-8 -*-
"""
Fitting functionality for bending.

@author: MarcGebhardt
"""
import copy
import warnings

import numpy as np
import pandas as pd
import lmfit
from tqdm import tqdm

# from .bfunc_class import (Bend_func_sub) # Bend_func_sub changed to bfs (redefinition)
from .attr_bgl import (_param_types_not_free, _param_types_fit_or_set)

#%% lmfit personalization

[docs]def lmfit_modelize(bfs, option='init'):
    """Returns a lmfit.model defined by Bend function sub."""
    mo=lmfit.Model(bfs.func, independent_vars=bfs.independent_vars)
    mo.name=bfs.name+'_fit'
    if option=='init':
        par = mo.make_params()
    else:
        raise NotImplementedError("Option %s not implemented!"%option)
    return mo,par


[docs]def lmfit_param_adder(par_df):
    """Returns lmfit Parameterset defined by pandas Dataframe."""
    params = lmfit.Parameters()
    for i in par_df.index:
        if par_df.loc[i,'typ'] == 'independent':
            pass
            # params.add(i, value = None, vary = False)
        elif par_df.loc[i,'typ'] == 'fixed':
            params.add(i, value = par_df.loc[i,'val'], vary = False)
        elif par_df.loc[i,'typ'] == 'expr':
            params.add(i, expr = par_df.loc[i,'val'], vary = False)
        elif par_df.loc[i,'typ'] == 'post':
            params.add(i, value = None, vary = False)
        elif par_df.loc[i,'typ'] == 'free':
            params.add(i, value = par_df.loc[i,'val'], vary = True,
                       min = par_df.loc[i,'min'], max = par_df.loc[i,'max'])
        else:
            raise NotImplementedError("Variable type not implemented!")
    return params


[docs]def lmfit_free_val_setter(bfs, param_val={}, default_val=-1.0):
    """Preset free values/parameters for lmfit."""
    pts=pd.Series(bfs.param_types)
    pval_guess={i: (param_val[i] if i in param_val.keys() else default_val)
                for i in pts.loc[pts=='free'].index}
    return pval_guess


[docs]def lmfit_param_key_checker(bfs, param_dict):
    """Check parameters of lmfit with Bend_func_sub."""
    param_dict=copy.deepcopy(param_dict)
    t=[]
    for i in param_dict.keys():
        if not i in list(bfs.param_types.keys()):
            t.append(i)
    for i in t:        
        param_dict.pop(i,None)
    return param_dict


[docs]def lmfit_param_prep(option,
                     param_name, param_val=None, param_type=None, 
                     param_min=None, param_max=None):
    """Prepare fit parameters for fitting."""
    if option=='1st_build':
        # w_variables_df=pd.DataFrame(np.full(len(w_variables_names),4,None),
        #                             index=w_variables_names,
        #                             columns=['type','val','min','max'])
        par_typ = pd.Series(param_type,index=param_name,dtype='string',name='typ')
        par_val = pd.Series(param_val,index=param_name,dtype='O',name='val')
        par_min = pd.Series(param_min,index=param_name,dtype='O',name='min')
        par_max = pd.Series(param_max,index=param_name,dtype='O',name='max')
        par_df = pd.DataFrame([par_typ,par_val,par_min,par_max]).T
        params = lmfit_param_adder(par_df)
        
    elif option=='1st_build_Bfs':
        par_df = pd.DataFrame(np.full((len(param_name.param_names),4),None),
                              index=param_name.param_names,
                              columns=['typ','val','min','max'])
        if param_type is None:
            par_typ = param_name.param_types
        else:
            par_typ = param_type
        if param_val is None:
            # par_val = param_name.param_vals
            raise ValueError("Set at least fixed and expression parameter values!")
        else:
            par_val = param_val
            par_val = lmfit_param_key_checker(param_name, par_val)
            par_val.update(lmfit_free_val_setter(param_name,par_val))
        if param_min is None:
            # par_min = param_name.param_mins
            par_min = {i: -np.inf for i in param_name.param_names}
        else:
            par_min = param_min
            par_min = lmfit_param_key_checker(param_name, par_min)
        if param_max is None:
            # par_max = param_name.param_maxs
            par_max = {i: np.inf for i in param_name.param_names}
        else:
            par_max = param_max
            par_max = lmfit_param_key_checker(param_name, par_max)
            
        par_df.loc[par_typ.keys(),'typ']=par_typ
        par_df.typ.loc[np.invert(par_df.typ.isin(_param_types_not_free))]='free'    
        par_df.loc[par_val.keys(),'val']=par_val
        par_df.loc[par_min.keys(),'min']=par_min
        par_df.loc[par_max.keys(),'max']=par_max
        params = lmfit_param_adder(par_df)
        
    elif option == 'old_result':
        params = param_name
    
    elif option == 'old_dict':
        params = lmfit.Parameters(param_name)
    else:
        raise NotImplementedError("Option not implemented!")
        
    return params

#%% Array shape helper 
# TODO: move to common

[docs]def shaped_array_fill_fandl(ShapeAr, fElemV, lElemV):
    """
    Returns an 1D numpy array with shape of input array and filled with ones.
    First and last element replaced with input data.

    Parameters
    ----------
    ShapeAr : numpy.array
        Input array wich determine shape of output array.
    fElemV : float64
        Value of first element passed to output array.
    lElemV : float64
        Value of last element passed to output array.

    Returns
    -------
    OutAr : numpy array
        1D numpy array with shape of input array and filled with ones.
        First and last element replaced with input data.

    """
    OutAr     = 0 * np.ones(shape=(len(ShapeAr),))
    OutAr[0]  = fElemV
    OutAr[-1] = lElemV
    return OutAr

#%% Fitting

[docs]def res_multi_const_weighted(params, x, func, func_d2,
                             x_lB, x_rB, func_err_weight=[1,10,100,100], 
                             load_dir='-y', data=None):
    """
    Returns weighted error sum according to different constraints between 
    input values and function values.
    Constraints:
        0. error between data and function value
        1. error between function value of left and right bound to zero
        2. error between negative/positive value of second derivate of function and zero (depends on load direction)
        3. error between value of second derivate of function of left and right bound to zero
    

    Parameters
    ----------
    params : OrderedDict / dict / array
        Parameters to be used in functions.
    x : array of float
        Cartesian coordinate in x-direction.
    func : lamopdified function
        Input function.
    func_d2 : lamopdified function
        Second derivate (curvature) of input function.
    x_lB : float
        Cartesian coordinate in x-direction of start of function (curvature=0, p.e. left bearing).
    x_rB : float
        Cartesian coordinate in x-direction of end of function (curvature=0, p.e. right bearing).
    func_err_weight : array of float, optional
        Error weights assigned to constraints. 
        Enter [1,0,0,0] for standard residual fit on displacement.
        The default is [1,10,100,100].
    load_dir : string, optional
        Direction of displacement application.
        Possible are:
            - "-y": application in negative y-direction (standard, curvature positve(err2))
            - "+y": application in negative y-direction (curvature negative(err2))
    data : array of float, optional
        Cartesian coordinate in y-direction. The default is None.

    Returns
    -------
    err: numpy array
        Weighted multi constraint error sum.

    """
    if data is None:
        return func(x,**params)
    if func_err_weight is None:
        func_err_weight=[1,1,1,1]
        
    ew=func_err_weight/np.sum(func_err_weight)
    
    # Error on displacement
    err0=(func(x,**params) - data)
    if func_err_weight == [1,0,0,0]:
        return err0
    # Error on displacement on left end right border in aspect to 0
    err1=(func(shaped_array_fill_fandl(x,x_lB,x_rB),**params)-0)*shaped_array_fill_fandl(x,1,1)
    # Error on curvature (have to be positive/negative for loading in -y/+y)
    if load_dir == '-y':
        err2=np.where(func_d2(x,**params)<0.0, -func_d2(x,**params), 0)
    elif load_dir == '+y':
        err2=np.where(func_d2(x,**params)>0.0, -func_d2(x,**params), 0)
    else:
        raise NotImplementedError("Loading direction %s not implemented!"%load_dir)
    # Error on curvature on left end right border in aspect to 0
    err3=(func_d2(shaped_array_fill_fandl(x,x_lB,x_rB),**params)-0)*shaped_array_fill_fandl(x,1,1)
    
    err=((err0*ew[0])**2+(err1*ew[1])**2+(err2*ew[2])**2+(err3*ew[3])**2)**0.5
    return err


[docs]def lmfit_bound_checker(Fit_Result, BFs, param_check_types = ['free'],level=1):
    """Check if lmfit Fit Result excides or hits bounds."""
    msg=''
    ub_bool = False
    lb_bool = False
    for p in Fit_Result.params:
        if BFs.param_types[p] in param_check_types:
            tv  = Fit_Result.params[p].value
            tub = Fit_Result.params[p].max
            tlb = Fit_Result.params[p].min
            if (tv >= tub) or (tv <= tlb):
                if tv >= tub:
                    ub_bool=True
                    msg+=('\nParameter %s with value %e excides maximum of %e'%(p,tv,tub))
                if tv <= tlb:
                    lb_bool=True
                    msg+=('\nParameter %s with value %e excides minimum of %e'%(p,tv,tlb))
            else:
                msg+=('\nParameter %s with value %e is inside bounds [%e,%e]'%(p,tv,tlb,tub))
    bound_bool=ub_bool|lb_bool
    return bound_bool, ub_bool, lb_bool, msg


[docs]def Multi_minimize(x, data, params, func_d0, func_d2, 
                   max_nfev, nan_policy, err_weights, x_lB, x_rB, load_dir='-y'):
    """
    Performs a weighted multiconstraint least-square-fit, based on lmfit.minimize.
    Returns lmfit-result, parameter-dict, Coefficient of determination for multi-errors and only displacement as well.

    Parameters
    ----------
    x : array of float
        Cartesian coordinate in x-direction.
    data : array of float, optional
        Cartesian coordinate in y-direction. The default is None.
    params : OrderedDict / dict / array
        Parameters to be used in functions.
    func_d0 : lamopdified function
        Input function.
    func_d2 : lamopdified function
        Second derivate (curvature) of input function.
    max_nfev : int
        Maximum number of function evaluations.
    err_weights :  array of float, optional
        Error weights assigned to constraints. 
        Enter [1,0,0,0] for standard residual fit on displacement.
        The default is [1,10,100,100].
    x_lB : float
        Cartesian coordinate in x-direction of start of function (curvature=0, p.e. left bearing).
    x_rB : float
        Cartesian coordinate in x-direction of end of function (curvature=0, p.e. right bearing).
    load_dir : string, optional
        Direction of displacement application.
        Possible are:
            - "-y": application in negative y-direction (standard, curvature positve(err2))
            - "+y": application in negative y-direction (curvature negative(err2))

    Returns
    -------
    MG_multi_minimize_Dict : dict
        Fit result dictionary (lmfit-result, parameter-dict, Coefficient of determination for multi-errors and only displacement as well.).

    """
    fit_Result = lmfit.minimize(res_multi_const_weighted, params,
                                args=(x,), kws={'func':func_d0,'func_d2':func_d2,
                                                'x_lB':x_lB, 'x_rB':x_rB,
                                                'func_err_weight': err_weights, 
                                                'load_dir': load_dir,
                                                'data': data},
                                scale_covar=True, max_nfev=max_nfev,
                                nan_policy=nan_policy)
    fit_params_dict = fit_Result.params.valuesdict()
    # Rquad_multi = 1 - fit_Result.residual.var() / np.var(data)
    Rquad_multi = 1 - fit_Result.residual.var() / np.nanvar(data)
    res_disp = res_multi_const_weighted(params=fit_params_dict, x=x,
                                        func=func_d0, func_d2=func_d2,
                                        x_lB=None, x_rB=None,
                                        func_err_weight=[1,0,0,0], 
                                        load_dir=load_dir, data=data)
    # Rquad_disp = 1 - res_disp.var() / np.var(data)
    Rquad_disp = 1 - np.nanvar(res_disp) / np.nanvar(data)
    Multi_minimize_Dict = dict({'Fit_Result': fit_Result, 'Fit_params_dict': fit_params_dict,
                                'Rquad_multi': Rquad_multi, 'Rquad_disp': Rquad_disp})
    return Multi_minimize_Dict


[docs]def Perform_Fit(BFL, Fit_func_key, P_df,
                lB, rB, s_range, 
                # Shear_func_key=None, t_mean=None, poisson=0.3, 
                Shear_func_key=None, gamma_V=None,
                err_weights=[ 1, 10, 1000, 100], max_nfev=500, nan_policy='raise',
                option='Pre', ldoption='fixed-y', ldoptionadd=None,
                pb_b=True,**pwargs):
    """
    Performs a weighted multiconstraint least-square-fit, based on lmfit.minimize.

    Parameters
    ----------
    BFL : Bend_func_legion
        Class conttaining information about bending line and derivates.
        See Bend_func_legion in ./bfunc_class.py for more information.
    Fit_func_key : string
        Identifier for fit function, p.e. 'w_A'.
    P_df : pd.DataFrame
        Measured and 2D-transformed displacment data. Combination of 
        x-coordinates and y-displacments.
    lB : float
        Left support x-coordinate, p.e. -10.0 with 20 mm span.
    rB : float
        Right support x-coordinate, p.e. 10.0 with 20 mm span.
    s_range : list or index
        Range of steps.
    Shear_func_key : string, optional
        Identifier for shear function, p.e. 'w_S'. The default is None.
    gamma_V : float, optional
        Ratio between shear to entire deformation in mid of bending beam.
        See gamma_V_det in ./bfunc_com.py
        The default is None.
    err_weights : list of float, optional
        Weights for multi contraint fitting. See res_multi_const_weighted.
        The default is [ 1, 10, 1000, 100].
    max_nfev : int, optional
        Number of evaluations. The default is 500.
    nan_policy : string, optional
        NaN handling. The default is 'raise'.
    option : string, optional
        Option for fitting. Possible are:
            - 'Pre': Pre fit with shear deformation
            - 'Bend': Refit to adjusted bending deformation
            (without indentation and shear deformation)
        The default is 'Pre'.
    ldoption : string, optional
        Load direction automatism.
        possible are:
            - 'fixed-y': Load application in -y-direction (default).
            - 'fixed+y': Load application in +y-direction.
            - 'auto-dispser': Load application direction automaticly determined 
            by a series of displacements (applied as ldoptionadd, 
            index have to match with s_range/P_df).
            - 'auto-Pcoorddisp': Load application direction automaticly 
            determined by a Points dataframe and specified point name and 
            coordinate (applied as ldoptionadd, index have to match with
            s_range/P_df)
        The default is 'fixed-y'.
    ldoptionadd : Series or array of [Dataframe, string, string], optional
        Addendum for ldoption.
        Have to match to ldoption:
            - 'auto-dispser': Series of displacements (index have to match with
            s_range/P_df).
            - 'auto-Pcoorddisp': Points dataframe and specified point name and 
            coordinate ([Points as Dataframe, point name as string, coordinate
            as string],index have to match with s_range/P_df)            
        The default is None.
    pb_b : bool, optional
        Switch for showing progressbar. The default is True.
    **pwargs : dict or pandas.Series
        Parameter keyword arguments for function.

    Raises
    ------
    NotImplementedError
        Option not implemented.

    Returns
    -------
    Fit_res_df : pandas.DatFrame
        Data of fit results.

    """
    
    if not option in ['Pre','Bend']:
                raise NotImplementedError("Option %s not implemented!"%option)
    if not ldoption in ['fixed-y','fixed+y','auto-dispser','auto-Pcoorddisp']:
                raise NotImplementedError("Option for load direction %s not implemented!"%ldoption)
        
    if pb_b: pb = tqdm(s_range, desc =option+" fit: ", unit=' steps', ncols=100)

    Fit_model, Fit_params = lmfit_modelize(BFL[Fit_func_key]['d0'],
                                           option='init')
    Fit_res_df = pd.DataFrame([],index=s_range,columns=['Fit_Result','Fit_params_dict',
                                                        'Rquad_multi','Rquad_disp'],
                              dtype='float64')
    
    for step in s_range:
        if step == s_range[0]:
            # Fit_params.add('xmin', value=lB, vary = False)
            # Fit_params.add('xmax', value=rB, vary = False)
            # if BFL.name=='FSE fit':
            #     Fit_params.add('FP', expr='pi/(xmax-xmin)', vary = False)
            #     Fit_params.add('b1', value=1.0)
            #     Fit_params.add('b2', value=1.0)
            #     Fit_params.add('b3', value=1.0)
            #     Fit_params.add('b4', value=1.0)
            #     if option == 'Pre':
            #         Fit_params.add('c',  value=1.0)
            #         Fit_params.add('d',  value=1.0)
            # elif BFL.name=='P4O fit':
            #     Fit_params.add('a', value=1.0)
            #     Fit_params.add('b', value=1.0)
            #     Fit_params.add('c', value=1.0)
            #     Fit_params.add('d', value=1.0)
            #     Fit_params.add('e', value=1.0)
            # else:
            #     raise NotImplementedError("Fit type not implemented!")
            # if option == 'Pre': Fit_params.add('f_V_0',  value=None, vary = False)
            if BFL.name=='FSE fit':
                # if 'pval' in pwargs.keys():
                #     pval=pwargs['pval']
                # else:
                #     pval = {'xmin':lB,'xmax':rB,'FP':'pi/(xmax-xmin)'}
                # Fit_params=lmfit_param_prep('1st_build_Bfs',
                #                             BFL[Fit_func_key]['d0'], pval)
                if 'param_val' not in pwargs.keys():
                    pwargs.update({'param_val':{'xmin':lB,'xmax':rB,'FP':'pi/(xmax-xmin)'}})
                Fit_params=lmfit_param_prep('1st_build_Bfs',
                                            BFL[Fit_func_key]['d0'], **pwargs)
            else:
                if BFL.name=='P4O fit':
                    Fit_params.add('xmin', value=lB, vary = False)
                    Fit_params.add('xmax', value=rB, vary = False)
                    Fit_params.add('a', value=1.0)
                    Fit_params.add('b', value=1.0)
                    Fit_params.add('c', value=1.0)
                    Fit_params.add('d', value=1.0)
                    Fit_params.add('e', value=1.0)
                else:
                    raise NotImplementedError("Fit type not implemented!")
                if option == 'Pre': Fit_params.add('f_V_0',  value=None, vary = False)
        else:
            # changed 21-09-07 (doesn't work with gaps in s_range)
            step_bf = s_range[s_range.get_indexer_for([step])[0]-1]
            # step_bf = Evac.pd_slice_index(s_range,step-1,option='list')
            # Fit_params = Fit_res_df.loc[step_bf,'Fit_Result'].params
            Fit_params=lmfit_param_prep('old_result',
                                        Fit_res_df.loc[step_bf,'Fit_Result'].params)
        
        if ldoption == 'fixed-y':
            load_dir = '-y'
        elif ldoption == 'fixed+y':
            load_dir = '+y'
        else:
            if ldoption == 'auto-dispser':
                load_dir_set = ldoptionadd.loc[step]
            elif ldoption == 'auto-Pcoorddisp':
                load_dir_set = ldoptionadd[0].loc[step].loc[ldoptionadd[1],ldoptionadd[2]]
            load_dir = '-y' if load_dir_set<=0 else '+y'
        
        x_data = P_df.loc[step].loc[:,'x'].values
        y_data = P_df.loc[step].loc[:,'y'].values
        f_tmp = Multi_minimize(x=x_data, data=y_data, params=Fit_params,
                               func_d0=BFL[Fit_func_key]['d0'].func,
                               func_d2=BFL[Fit_func_key]['d2'].func,
                               max_nfev=max_nfev,  nan_policy=nan_policy,
                               err_weights=err_weights,
                               x_lB=lB, x_rB=rB, load_dir=load_dir)
        Fit_res_df.loc[step] = f_tmp
        # Check boundaries for free paramters
        bccheck = lmfit_bound_checker(f_tmp['Fit_Result'],BFL[Fit_func_key]['d0'])
        if bccheck[0]: warnings.warn(UserWarning(bccheck[-1]))
        if pb_b: pb.update()
    if pb_b: pb.close()
    
    if option =='Pre':    
    # Shear-force-deformation ratio
        f_S_0 = BFL['w_S']['d0'](0.0,Fit_res_df.loc[:,'Fit_params_dict'])
        # Length = lB-rB
        # gamma_V = 2.4*(1+poisson)*t_mean**2/Length**2
        f_V_0 = f_S_0*gamma_V/(1+gamma_V)
        for step in s_range:
            Fit_res_df.loc[step,'Fit_params_dict'].update({'f_V_0':f_V_0.loc[step]})
            
    return Fit_res_df

# #%% Fitting parameters
# def params_dict_sub(params_dict, step, dif_step, BFs,
#                     param_types_dif=_param_types_fit_or_set):
#     """Performs a substraction of parameter values
#     with specified type in a parameter dictionary.
#     Warning: Seems to be not accurate!"""
#     a = params_dict.loc[dif_step]
#     b = params_dict.loc[step]
#     params_dict_res = copy.deepcopy(b)
#     for key in b:
#         # if key in ['b1','b2','b3','b4','c','d','f_V_0']:
#         if BFs.param_types[key] in param_types_dif:
#             params_dict_res[key]=b[key]-a[key]
#     return params_dict_res

# def params_dict_diff(params_dict_series, step_range, BFs,
#                     param_types_dif=_param_types_fit_or_set,
#                     option='Fill_NaN'):
#     """Performs a differencation of a series of parameter values
#     with specified type in a parameter dictionary.
#     Warning: Seems to be not accurate!"""
#     params_dict_res=pd.Series([],dtype='O')
#     for step in step_range:        
#         step_bf = step_range[step_range.get_indexer_for([step])[0]-1]
#         if step_bf == (step-1):
#             params_dict_res.loc[step] = params_dict_sub(params_dict_series, step, step_bf,
#                                                         BFs, param_types_dif)
#         elif option == 'Next' and step_bf < step:
#             params_dict_res.loc[step] = params_dict_sub(params_dict_series, step, step_bf,
#                                                         BFs, param_types_dif)
#         else:
#             params_dict_res.loc[step] = np.nan
#     return params_dict_res
```

---

© Copyright 2024, MarcGebhardt.

Built with Sphinx using a
theme
provided by Read the Docs.
