# Supplementary material for: Approach to Standardized Material Characterization of the Human Lumbopelvic System: Testing and Evaluation
Source: Bioengineering (Basel). 2025 Aug 11;12(8):862. doi: 10.3390/bioengineering12080862 (PMC12383908; doi:10.3390/bioengineering12080862)
Supplement: Supplementary file 1 [file bioengineering-12-00862-s001.zip › File S3 Evaluation code/ExMechEva-0.1.2/docs/_build/html/_modules/exmecheva/bending/opt_mps.html]

exmecheva.bending.opt\_mps — ExMechEva 0.1.0 documentation


ExMechEva

Contents:

- ExMechEva

ExMechEva

- Module code
- exmecheva.bending.opt\_mps

---

# Source code for exmecheva.bending.opt\_mps

```
# -*- coding: utf-8 -*-
"""
Adds funcionality for optical measured cartesian points.
(Mostly vector geometry)

@author: MarcGebhardt
"""
import numpy as np
import pandas as pd
import matplotlib.pyplot as plt

import vg

import exmecheva.common as emec

from .attr_bgl import (_significant_digits_fpd)

[docs]def v_length(v1):
    """
    Computes the length of an vector
    """
    length=np.sqrt(np.sum(v1**2))
    return length


[docs]def v_Ctrans (v,TM,P0):
    """
    Transform a set of vectors(v=ixN3) with a translationmatrix (TM) and a new origin (P0)
    """
    if type(v).__name__ == 'ndarray':
        vtrans=np.ndarray(v.shape)
        for i in range(v.shape[1]):
            vtrans[:,i]=np.dot(TM,(v[:,i]-P0))
    elif type(v).__name__ == 'DataFrame':
        vtrans=pd.DataFrame(index=v.index,columns=v.columns)
        for i in v.columns:
            vtrans.loc[:,i]=np.dot(TM,(v.loc[:,i]-P0))
    return vtrans


[docs]def Point_df_transform(Pmeas, Pspec, Pmeas_Sdev, Pspec_Sdev, 
                       dic_P_name_org1, dic_P_name_org2,
                       output_lvl=0, log_mg=''):
    """
    Calculate in plane transformation of given measured 3D-points.

    Parameters
    ----------
    Pmeas : pandas.Dataframe([], index=['x','y','z'], 
                             columns=['P1','P2',...], dtype=float64)
        Cartesian coordinates of measured points, which should be fitted.
    Pspec : pandas.Dataframe([], index=['x','y','z'], 
                             columns=['S1','S2',...], dtype=float64)
        Cartesian coordinates of special points which should used for 
        coordinate transformation.
    Pmeas_Sdev : pandas.Dataframe([], index=['Sx','Sy','Sz'],
                                  columns=['P1','P2',...], dtype=float64)
        Standard deviation of measured points, which should be fitted.
    Pspec_Sdev : pandas.Dataframe([], index=['Sx','Sy','Sz'],
                                  columns=['S1','S2',...], dtype=float64)
        Standard deviation of special points which should used for coordinate 
        transformation.
    dic_P_name_org1 : str
        Name of point, which should be on the negativ x-axis. 
        Origin is in the middle between dic_P_name_org1 and dic_P_name_org2.
    dic_P_name_org2 : str
        Name of point, which should be on the positiv x-axis. 
        Origin is in the middle between dic_P_name_org1 and dic_P_name_org2.


    Returns
    -------
     Points_T
         Transformed coordinates and standard deviation of given points.
     Points_L_T
         In plane transformed coordinates and standard deviation of given points.

    """
    # Plane-Fitting
    A = np.column_stack([Pspec.loc['x'], Pspec.loc['y'], np.ones(len(Pspec.loc['x']))]) # Ebene auf Punkte Versuchseinrichtung gelegt
    b = Pspec.loc['z']
    P_fit, res, _, _ = np.linalg.lstsq(A, b, rcond=None)
    fitnorm=vg.normalize(np.array([P_fit[0],P_fit[1],-1]))
#    rquad=1 - res / (b.size * b.var()) # nur bei Pmeas als Eingang
    
    Pmeas_dist=(P_fit[0] * Pmeas.loc['x'] + P_fit[1] * Pmeas.loc['y'] - Pmeas.loc['z'] + P_fit[2])/v_length(np.array([P_fit[0],P_fit[1],-1]))
    Pspec_dist=(P_fit[0] * Pspec.loc['x'] + P_fit[1] * Pspec.loc['y'] - Pspec.loc['z'] + P_fit[2])/v_length(np.array([P_fit[0],P_fit[1],-1]))
    
    if output_lvl>=1:
        log_mg.write("\n    Fitting solution: %f x + %f y + %f = z" % (P_fit[0], P_fit[1], P_fit[2]))
#        print("R² = %.3f" %rquad) # nur bei Pmeas als Eingang
        log_mg.write("\n    Mean distance from meas. to spec. points = %.3e" %Pmeas_dist.mean())
    
    #Lotfußpunkte
    Pmeas_L=pd.DataFrame(data=np.zeros(Pmeas.shape),index=Pmeas.index,columns=Pmeas.columns)
    for i in range(Pmeas.shape[1]):
        Pmeas_L.iloc[:,i]=Pmeas.iloc[:,i]-Pmeas_dist[i]*fitnorm
    Pspec_L=pd.DataFrame(data=np.zeros(Pspec.shape),index=Pspec.index,columns=Pspec.columns)
    for i in range(Pspec.shape[1]):
        Pspec_L.iloc[:,i]=Pspec.iloc[:,i]-Pspec_dist[i]*fitnorm
    
    if output_lvl>=2:    
        # plot raw data
        plt.figure()
        ax = plt.subplot(111, projection='3d')
        ax.scatter(Pmeas.loc['x'], Pmeas.loc['y'],Pmeas.loc['z'], color='b',label='meas. points')
        ax.scatter(Pspec.loc['x'], Pspec.loc['y'],Pspec.loc['z'], color='m',label='spec. points')
        #plot Lotfußpunkte
        ax.scatter(Pmeas_L.loc['x'], Pmeas_L.loc['y'],Pmeas_L.loc['z'], color='c', marker="+",label='meas. lot points')
        ax.scatter(Pspec_L.loc['x'], Pspec_L.loc['y'],Pspec_L.loc['z'], color='r', marker="+",label='spec. lot points')
        # Create cubic bounding box to simulate equal aspect ratio
        max_range = np.array([Pmeas_L.loc['x'].max()-Pmeas_L.loc['x'].min(), Pmeas.loc['y'].max()-Pmeas.loc['y'].min(), Pmeas.loc['z'].max()-Pmeas.loc['z'].min()]).max()
        Xb = 0.5*max_range*np.mgrid[-1:2:2,-1:2:2,-1:2:2][0].flatten() + 0.5*(Pmeas_L.loc['x'].max()+Pmeas_L.loc['x'].min())
        Yb = 0.5*max_range*np.mgrid[-1:2:2,-1:2:2,-1:2:2][1].flatten() + 0.5*(Pmeas.loc['y'].max()+Pmeas.loc['y'].min())
        Zb = 0.5*max_range*np.mgrid[-1:2:2,-1:2:2,-1:2:2][2].flatten() + 0.5*(Pmeas.loc['z'].max()+Pmeas.loc['z'].min())
        # Comment or uncomment following both lines to test the fake bounding box:
        for xb, yb, zb in zip(Xb, Yb, Zb):
            ax.plot([xb], [yb], [zb], 'w')
        # plot plane
        xlim = ax.get_xlim()
        ylim = ax.get_ylim()
        X,Y = np.meshgrid(np.arange(xlim[0], xlim[1]),
          np.arange(ylim[0], ylim[1]))
        Z = np.zeros(X.shape)
        for r in range(X.shape[0]):
           for c in range(X.shape[1]):
               Z[r,c] = P_fit[0] * X[r,c] + P_fit[1] * Y[r,c] + P_fit[2]
        ax.plot_wireframe(X,Y,Z, color='g',label='fit plane')
        ax.set_xlabel('x')
        ax.set_ylabel('y')
        ax.set_zlabel('z')
        plt.show()
        
    # Koordinatentransformation
    KT_Pzero=(Pspec_L.loc[:,dic_P_name_org1]+Pspec_L.loc[:,dic_P_name_org2])/2 # Koordinatenursprung auf Mitte org1 und org2 (Standard S1 und S2)
    KT_Pxdir=Pspec_L.loc[:,dic_P_name_org2] # Richtung x-Achse in org2 (Standard S2)
    KT_Vxdir=vg.normalize(KT_Pxdir-KT_Pzero)
    KT_Vzdir=fitnorm # z-Achse in Normalen-Richtung des Ebenen-fits 
    KT_Vydir=vg.perpendicular(KT_Vxdir,KT_Vzdir, normalized=True)
    KT_MTrans=np.array([KT_Vxdir,KT_Vydir,KT_Vzdir]) # Transformationsmatrix
        
    Pmeas_T     =v_Ctrans(Pmeas,    KT_MTrans,KT_Pzero)
    Pmeas_L_T   =v_Ctrans(Pmeas_L,  KT_MTrans,KT_Pzero)
    Pspec_T     =v_Ctrans(Pspec,    KT_MTrans,KT_Pzero)
    Pspec_L_T   =v_Ctrans(Pspec_L,  KT_MTrans,KT_Pzero)
    
    Pmeas_Sdev_T     =v_Ctrans(Pmeas_Sdev,    KT_MTrans,np.zeros(3))
    Pspec_Sdev_T     =v_Ctrans(Pspec_Sdev,    KT_MTrans,np.zeros(3))
    
    Points_T=pd.concat([pd.concat([Pmeas_T,Pmeas_Sdev_T],axis=0,sort=True),pd.concat([Pspec_T,Pspec_Sdev_T],axis=0,sort=True)],axis=1,sort=True)
    Points_L_T=pd.concat([pd.concat([Pmeas_L_T,Pmeas_Sdev_T],axis=0,sort=True),pd.concat([Pspec_L_T,Pspec_Sdev_T],axis=0,sort=True)],axis=1,sort=True)
    
    if output_lvl>=2:    
        # plot transformed data
        plt.figure()
        ax = plt.subplot(111, projection='3d')
        ax.scatter(Pmeas_T.loc['x'], Pmeas_T.loc['y'], Pmeas_T.loc['z'], color='b',label='meas. points')
        ax.scatter(Pspec_T.loc['x'], Pspec_T.loc['y'], Pspec_T.loc['z'], color='r',label='spec. points')
        # plot Lotfußpunkte
        ax.scatter(Pmeas_L_T.loc['x'], Pmeas_L_T.loc['y'], Pmeas_L_T.loc['z'], color='c', marker="+",label='meas. lot points')
        ax.scatter(Pspec_L_T.loc['x'], Pspec_L_T.loc['y'], Pspec_L_T.loc['z'], color='y', marker="+",label='spec. lot points')
        ax.set_xlabel('x')
        ax.set_ylabel('y')
        ax.set_zlabel('z')
        plt.show()

    if output_lvl>=2:    
        fig, ax1 = plt.subplots()
        ax1.set_xlabel('x / mm')
        ax1.set_ylabel('y / mm')
        plt.axis('equal')
        ax1.plot(Pmeas_L_T.loc['x'], Pmeas_L_T.loc['y'], 'r+',label='meas. points')
        ax1.grid()
        fig.legend(loc='upper left', bbox_to_anchor=(0.12, 0.88))
        fig.tight_layout()  # otherwise the right y-label is slightly clipped
        plt.show()
        fig.clf()
        plt.close(fig)
        
    return (Points_T, Points_L_T)


[docs]def Point_df_idx(df, steps=None, points=None, coords=None,
                 deepcopy=True, option=None):
    """Indexing a points dataframe by step (index), point-names and coordinates."""
    if deepcopy:
        dfs=df.copy(deep=True)
    else:
        dfs=df

    if option=='Regex':
        # if (type(points) is not str) or (type(coords) is not str):
        if not (isinstance(points,str) or isinstance(coords,str)):
            raise ValueError("Input is not a regex-string!")
            
    def nonchecker(vcheck):
        if vcheck is None:
            vcbool = False
        #elif (len(vcheck)>1) and not (type(vcheck) is str):
        #    vcbool = (vcheck!=None).all()
        else:
        #    vcbool = vcheck!=None
            vcbool = True
        return vcbool

    stepsbool=nonchecker(steps)
    pointsbool=nonchecker(points)
    coordsbool=nonchecker(coords)

    if stepsbool:
        dfs=dfs.loc(axis=0)[steps]

    if pointsbool:
        if type(dfs) is pd.core.series.Series:
            if option=='Regex':
                #points=dfs.droplevel(level=1).str.contains(pat=points,na=False,regex=True)
                points=dfs.index.droplevel(level=1).str.contains(
                    pat=points,na=False,regex=True
                    )
            dfs=dfs.loc[points,:]
        else:
            if option=='Regex':
                points=dfs.columns.droplevel(level=1).str.contains(
                    pat=points,na=False,regex=True
                    )
            dfs=dfs.loc(axis=1)[points,:]

    if coordsbool:
        if type(dfs) is pd.core.series.Series:
            if option=='Regex':
                coords=dfs.index.droplevel(level=0).str.contains(
                    pat=coords,na=False,regex=True
                    )
            #     dfs=dfs.loc[:,coords]
            # else:
            #     dfs=dfs.loc[:,[coords]]
            # if type(coords) is list:
            if isinstance(coords,(list,np.ndarray)):
                dfs=dfs.loc[:,coords]
            else:
                dfs=dfs.loc[:,[coords]]
        else:
            if option=='Regex':
                coords=dfs.columns.droplevel(level=0).str.contains(
                    pat=coords,na=False,regex=True
                    )
            #     dfs=dfs.loc(axis=1)[:,coords]
            # else:
            #     dfs=dfs.loc(axis=1)[:,[coords]]
            # if type(coords) is list:
            if isinstance(coords,(list,np.ndarray)):
                dfs=dfs.loc(axis=1)[:,coords]
            else:
                dfs=dfs.loc(axis=1)[:,[coords]]

    return dfs


[docs]def Points_dif_step(df, dif_step=0,
                    steps=None, points=None, coords=None,
                    deepcopy=True, option=None):
    """Substracts a Points df from an other."""
    dfs    = Point_df_idx(df,steps,points,coords,deepcopy,option)
    # Fehler bei Points_df_idx (Ausgabe Serie, muss var enthalten)
    df_dif = Point_df_idx(df,dif_step,points,coords,deepcopy,option)
    # df_dif = df.loc(axis=1)[:,coords].loc[dif_step]
    dfs    = dfs - df_dif
    return dfs


[docs]def Points_add_step(df, add_step=0, add_df=None,
                    steps=None, points=None, coords=None,
                    deepcopy=True, option=None):
    """Adding a Points df to an other."""
    dfs    = Point_df_idx(df,steps,points,coords,deepcopy,option)
    if add_df is None:
        df_add = Point_df_idx(df,add_step,points,coords,deepcopy,option)
    else:
        df_add = Point_df_idx(add_df,add_step,points,coords,deepcopy,option)
    dfs    = dfs + df_add
    return dfs


[docs]def Points_diff(df, diff_coord='y', first_val=0,
                steps=None, points=None, coords=None,
                deepcopy=True, option=None, 
                nan_policy = 'corr_nan'):
    """Returns differential of specified coordinates of Points."""
    dfs    = Point_df_idx(df,steps,points,coords,deepcopy,option)
    df_diff = Point_df_idx(df,steps,points,diff_coord,deepcopy,option).diff()
    if not np.isnan(first_val):
        df_diff.iloc[0]=0
    dfs.loc(axis=1)[:,diff_coord] = df_diff
    if nan_policy == 'corr_nan':
        sdf = dfs.loc(axis=1)[:,diff_coord].isna().droplevel(1,axis=1)
        dfs[sdf] = np.nan
    return dfs


[docs]def Points_eval_func(func, fit_params, pointdf, steps,
                     in_coord='x', out_coord='y', points=None,
                     deepcopy=True, option=None):
    """Evaluates a dataframe or series by a function."""
    if type(fit_params) is not pd.core.series.Series:
        raise ValueError('Fit Parameters are not pandas series!')
    
    dfs = Point_df_idx(df=pointdf, steps=steps,
                       points=points, coords=in_coord,
                       deepcopy=deepcopy, option=option)
    if type(dfs) is pd.core.frame.DataFrame:
        dfs = dfs.apply(lambda b: func(b,**fit_params.loc[b.name]),axis=1)
    else:
        dfs = dfs.apply(lambda b: func(b,**fit_params.loc[b.name]))
    dfs = dfs.rename(columns={in_coord: out_coord})

    return dfs


[docs]def Point_df_from_lin(x, steps, coords=['x','y'], coords_set='x',
                      col_type='str', Point_prefix='L'):
    """Prepare a points dataframe with index of step and values of x."""
    if col_type == 'str':
        xt  = Point_prefix + pd.Series(x).index.map(str)
    elif col_type == 'val':
        xt  = x
    else:
        raise NotImplementedError("Column index type %s not implemented!"%col_type)
    if coords is None:
        # round float point division
        mit = pd.Index(xt).map(lambda x: emec.helper.round_to_sigdig(
            x, _significant_digits_fpd
            ))
        dft = pd.DataFrame([],index=steps,columns=mit)
        if not coords_set is None: dft.loc(axis=1)[:]=x
    else:        
        mit = pd.MultiIndex.from_product([xt,coords], names=['Points','Vars'])
        dft = pd.DataFrame([],index=steps,columns=mit)
        if not coords_set is None: dft.loc(axis=1)[:,coords_set]=x
    return dft


[docs]def Point_df_combine(df1, df2, coords_set='y',
                    deepcopy=True, option=None):
    """Combines a deepcopy of one points dataframe to an other."""
    dfs=df1.copy(deep=deepcopy)
    dfs.loc(axis=1)[:,coords_set]=df2.loc(axis=1)[:,coords_set]
    return dfs
```

---

© Copyright 2024, MarcGebhardt.

Built with Sphinx using a
theme
provided by Read the Docs.
