# Supplementary material for: Approach to Standardized Material Characterization of the Human Lumbopelvic System: Testing and Evaluation
Source: Bioengineering (Basel). 2025 Aug 11;12(8):862. doi: 10.3390/bioengineering12080862 (PMC12383908; doi:10.3390/bioengineering12080862)
Supplement: Supplementary file 1 [file bioengineering-12-00862-s001.zip › File S3 Evaluation code/ExMechEva-0.1.2/docs/_build/html/_modules/exmecheva/bending/plotting.html]

exmecheva.bending.plotting — ExMechEva 0.1.0 documentation


ExMechEva

Contents:

- ExMechEva

ExMechEva

- Module code
- exmecheva.bending.plotting

---

# Source code for exmecheva.bending.plotting

```
# -*- coding: utf-8 -*-
"""
Plotting functionality for bending.

@author: MarcGebhardt
"""
import matplotlib.pyplot as plt
import matplotlib.cm as cm
import matplotlib.colors as colors

import exmecheva.common as emec

from .opt_mps import (Point_df_idx, Point_df_from_lin, Point_df_combine)

[docs]def colplt_df_ax(df, step_range=None,
                 title='', xlabel='', ylabel='',
                 Point_df=None, ax=None,
                 cblabel='Step', cbtick=None):
    """Returns a matpltlib axis plot of a pandas Dataframe of type points in a range."""
    if step_range is None: step_range=df.index
    if ax is None: ax = plt.gca()
    cb_map=cm.ScalarMappable(
        norm=colors.Normalize(
            vmin=step_range.min(), vmax=step_range.max()), cmap=cm.rainbow
        )
    color=cb_map
    # color=cm.rainbow(np.linspace(0,1,step_range.max()-step_range.min()+1))
    
    ax.set_title(title)
    ax.set_xlabel(xlabel)
    ax.set_ylabel(ylabel)
    for step in step_range:
        ax.plot(Point_df_idx(df,steps=step,coords='x'),
                Point_df_idx(df,steps=step,coords='y'),
                '-',c=color.to_rgba(step))
        if Point_df is not None: 
            ax.plot(Point_df_idx(Point_df,steps=step,coords='x'),
                    Point_df_idx(Point_df,steps=step,coords='y'),
                    'k+', markersize=.8)
    if cbtick is None:
        cb1=plt.colorbar(cb_map, extend='max', label=cblabel, ax=ax)
    else:
        cb1=plt.colorbar(cb_map, extend='max', label=cblabel, ax=ax,
                         ticks=list(cbtick.values()))
        cb1.ax.set_yticklabels(list(cbtick.keys()))
    cb1.ax.invert_yaxis()
    ax.grid()
    return ax


[docs]def colplt_common_ax(xdata, ydata, step_range=None,
                     title='', xlabel='', ylabel='',
                     xstep=False, ystep=True,
                     Point_df=None, ax=None,
                     cblabel='Step', cbtick=None):
    """Returns a matpltlib axis plot of a pandas Dataframe of type points in a range."""
    if step_range is None:
        if ystep: step_range=ydata.index
        elif xstep: step_range=xdata.index
        else: step_range=[0]
    if ax is None: ax = plt.gca()
    cb_map=cm.ScalarMappable(norm=colors.Normalize(
        vmin=step_range.min(), vmax=step_range.max()),cmap=cm.rainbow)
    color=cb_map
    
    ax.set_title(title)
    ax.set_xlabel(xlabel)
    ax.set_ylabel(ylabel)
    for step in step_range:
        ax.plot(xdata.loc[step] if xstep else xdata,
                ydata.loc[step] if ystep else ydata,
                '-',c=color.to_rgba(step))
    if cbtick is None:
        cb1=plt.colorbar(cb_map, extend='max', label=cblabel, ax=ax)
    else:
        cb1=plt.colorbar(cb_map, extend='max', label=cblabel, ax=ax,
                         ticks=list(cbtick.values()))
        cb1.ax.set_yticklabels(list(cbtick.keys()))
    cb1.ax.invert_yaxis()
    ax.grid()
    return ax


[docs]def colplt_funcs_ax(x, func, params, step_range=None,
                    title='', xlabel='', ylabel='',
                    Point_df=None, ax=None,
                    cblabel='Step', cbtick=None):
    """Returns a matpltlib axis plot of a function with defined parameters in a range."""
    if step_range is None: step_range=params.index
    df1 = Point_df_from_lin(x, step_range)
    try: #wenn nicht alle enthalten sind (bei RangeIndex)
        df2 = func(x, params.loc[step_range])
    except KeyError:
        df2 = func(x, params.indlim(step_range.min(),step_range.max()))
    df  = Point_df_combine(df1, df2)
    ax  = colplt_df_ax(df, step_range,
                       title, xlabel, ylabel,
                       Point_df, ax,
                       cblabel, cbtick)
    return ax


[docs]def colplt_funcs_one(x, func, params, step_range=None,
                     title='', xlabel='', ylabel='',
                     Point_df=None,
                     cblabel='Step', cbtick=None,
                     path=None,
                     plt_scopt={'tight':True, 'show':True, 
                                'save':True, 's_types':["pdf"], 
                                'clear':True, 'close':True}):
                     # savefig=False, savefigname=None):
    """Returns a matpltlib figure plot of a function with defined 
    parameters in a range."""
     
    fig, ax1 = plt.subplots()
    colplt_funcs_ax(x, func, params, step_range,
                    title, xlabel, ylabel,
                    Point_df, ax1,
                    cblabel, cbtick)
    emec.plotting.plt_handle_suffix(fig, path=path, **plt_scopt)


[docs]def colplt_funcs_all(x, func_cohort, params, step_range=None,
                     title='', xlabel='',
                     Point_df=None,
                     cblabel='Step', cbtick=None,
                     path=None,
                     plt_scopt={'tight':True, 'show':True, 
                                'save':True, 's_types':["pdf"], 
                                'clear':True, 'close':True}):
    """Returns a matpltlib axis plot of a function cohort 
    (function and theire first and second derivate)
    with defined parameters in a range."""
    fig, (ax1,ax2,ax3) = plt.subplots(nrows=3, ncols=1,
                                      sharex=False, sharey=False,
                                      figsize = (
                                          plt.rcParams['figure.figsize'][0],
                                          plt.rcParams['figure.figsize'][1]*3
                                          ))
    fig.suptitle(title)
    colplt_funcs_ax(x=x, func=func_cohort['d0'],
                    params=params, step_range=step_range,
                    title='Displacement', 
                    xlabel=xlabel, ylabel='Displacement / mm',
                    Point_df=Point_df, ax=ax1,
                    cblabel=cblabel, cbtick=cbtick)
    colplt_funcs_ax(x=x, func=func_cohort['d1'],
                    params=params, step_range=step_range,
                    title='Slope', 
                    xlabel=xlabel, ylabel='Slope / (mm/mm)',
                    Point_df=None, ax=ax2,
                    cblabel=cblabel, cbtick=cbtick)
    colplt_funcs_ax(x=x, func=func_cohort['d2'],
                    params=params, step_range=step_range,
                    title='Curvature', 
                    xlabel=xlabel, ylabel='Curvature / (1/mm)',
                    Point_df=None, ax=ax3,
                    cblabel=cblabel, cbtick=cbtick)
    emec.plotting.plt_handle_suffix(fig, path=path, **plt_scopt)
```

---

© Copyright 2024, MarcGebhardt.

Built with Sphinx using a
theme
provided by Read the Docs.
