# Supplementary material for: Approach to Standardized Material Characterization of the Human Lumbopelvic System: Testing and Evaluation
Source: Bioengineering (Basel). 2025 Aug 11;12(8):862. doi: 10.3390/bioengineering12080862 (PMC12383908; doi:10.3390/bioengineering12080862)
Supplement: Supplementary file 1 [file bioengineering-12-00862-s001.zip › File S3 Evaluation code/ExMechEva-0.1.2/docs/_build/html/_modules/exmecheva/common/analyze.html]

exmecheva.common.analyze — ExMechEva 0.1.0 documentation


ExMechEva

Contents:

- ExMechEva

ExMechEva

- Module code
- exmecheva.common.analyze

---

# Source code for exmecheva.common.analyze

```
# -*- coding: utf-8 -*-
"""
Analyzing functionality.

@author: MarcGebhardt
"""
import warnings
import numpy as np
import pandas as pd

from .helper import (check_empty)

#%% core analysing

[docs]def sign_n_change(y):
    """
    Computes sign and sign change of an array.

    Parameters
    ----------
    y : pandas.Series of float
        Array which should analyzed.

    Returns
    -------
    y_sign : pandas.Series of integer
        Signs of y.
    y_signchange : pandas.Series of boolean
        Sign changes of y.

    """
    y_sign       = np.sign(y)
    y_signchange = ((np.roll(y_sign, True) - y_sign) != False).astype(bool)
    y_sign       = pd.Series(y_sign,index=y.index)
    y_signchange = pd.Series(y_signchange,index=y.index)
    return (y_sign,y_signchange)


[docs]def normalize(pdo, axis=0, norm='absmax', normadd=0.5, pdo_n=None,
              warn_excl=['x_sc','dx_sc']):
    """
    Normalize an array in respect to given option.

    Parameters
    ----------
    pdo : pandas.Series or pandas.DataFrame
        Input values.
    axis : {0 or ‘index’, 1 or ‘columns’},, optional
        Axis to apply normalizing. The default is 0.
    norm : string, optional
        Apllied option for normalizing.
        Possible are:
            - 'absmax': Normalize to maximum of absolut input values.
            - 'absmin': Normalize to minimum of absolut input values.
            - 'absqua': Normalize to Quantile of absolut input values (specified with normadd).
            - 'max': Normalize to maximum of input values.
            - 'min': Normalize to minimum of input values.
            - 'qua': Normalize to Quantile of input values (specified with normadd).
            - 'val': Normalize to given value (specified with normadd).
            - None: No normalization (return input values).
        The default is 'absmax'.
    normadd : int or float, optional
        Additional parameter for option 'qua'(to set quantile value), 
        or 'val'(to set norm parameter directly).
        The default is 0.5.
    pdo_n : pandas.Series or pandas.DataFrame or None, optional
        Additional values to get normalizing parameter.
        If None use input values. If type is Dataframe, same shape required.
        The default is None.
    warn_excl : list
        Defines exclusions of ZeroDivisionError-prevention-warnings.
        The default is ['x_sc','dx_sc'].
    
    Raises
    ------
    NotImplementedError
        Option not implemented.

    Returns
    -------
    o : pandas.Series or pandas.DataFrame
        Normalized output values.

    """
    if (axis==1) and (norm in ['absmeanwoso','meanwoso']):
        raise NotImplementedError("Axis %s and norm %s not implemented!"%(axis,norm))
        
    if pdo_n is None:
        pdo_n = pdo
        
    if   norm == 'absmax':
        npar = abs(pdo_n).max(axis=axis)
    elif norm == 'absmin':
        npar = abs(pdo_n).min(axis=axis)
    elif norm == 'absmean':
        npar = abs(pdo_n).mean(axis=axis)
    elif norm == 'absmeanwoso':
        npar = abs(pdo_n).meanwoso()
    elif norm == 'absmed':
        npar = abs(pdo_n).median(axis=axis)
    elif norm == 'absqua':
        if isinstance(pdo_n, pd.core.base.ABCSeries):
            npar = abs(pdo_n).quantile(normadd)
        else:
            npar = abs(pdo_n).quantile(normadd,axis=axis)
            
    elif norm == 'max':
        npar = pdo_n.max(axis=axis)
    elif norm == 'min':
        npar = pdo_n.min(axis=axis)
    elif norm == 'mean':
        npar = pdo_n.mean(axis=axis)
    elif norm == 'meanwoso':
        npar = pdo_n.meanwoso()
    elif norm == 'med':
        npar = pdo_n.median(axis=axis)
    elif norm == 'qua':
        if isinstance(pdo, pd.core.base.ABCSeries):
            npar = pdo_n.quantile(normadd)
        else:
            npar = pdo_n.quantile(normadd,axis=axis)
            
    elif norm == 'val':
        if isinstance(pdo_n, pd.core.base.ABCDataFrame):
            npar = pd.Series([normadd,]*len(pdo_n.columns),
                             index=pdo_n.columns)
        else:
            npar = normadd
    elif norm is None:
        if isinstance(pdo_n, pd.core.base.ABCDataFrame):
            npar = pd.Series([1,]*len(pdo_n.columns),
                             index=pdo_n.columns)
        else:
            npar = 1
    else:
        raise NotImplementedError("Norming %s not implemented!"%norm)
    if isinstance(pdo_n, pd.core.base.ABCSeries):
        if npar == 0:
            npar = 1
            npar_0 = pdo_n.name
            if not npar_0 in warn_excl:
                warnings.warn('ZeroDivisionError prevented by setting norming to 1 for variable: {}'.format(npar_0))
    elif (npar == 0).any():
        npar[npar == 0] = 1
        # npar_0 = npar[npar == 0].index.values
        test=npar[npar==0].index.to_series().apply(lambda x: x not in warn_excl)
        if test.any():
            npar_0=npar[npar==0][test].index.tolist()
            warnings.warn('ZeroDivisionError prevented by setting norming to 1 for variables: {}'.format(npar_0))
    o = pdo/npar
    return o


[docs]def threshhold_setter(pdo, th=None, option='abs', set_val='th'):
    if set_val == 'th':
        set_val = th
    if th is None:
        return pdo
    if option == 'abs':
        o = pdo.where(pdo.abs() > th, set_val)
    elif option == 'lower':
        o = pdo.where(pdo       > th, set_val)
    elif option == 'higher':
        o = pdo.where(pdo       < th, set_val)
    else:
        raise NotImplementedError("Option %s not implemented!"%option)
    return o


[docs]def normalize_th(pdo, axis=0, pdo_n=None, norm='absmax', normadd=0.5,
                 th=None, th_option='abs', th_set_val='th',
                 warn_excl=['x_sc','dx_sc']):
    pds=normalize(pdo, axis=axis, norm=norm, normadd=normadd, pdo_n=pdo_n,
                  warn_excl=warn_excl)
    pdt=threshhold_setter(pds, th=th, option=th_option, set_val=th_set_val)
    return pdt


[docs]def sign_n_changeth(y, axis=0, norm=None, normadd=0.5,
                    th=None, th_option='abs', th_set_val=0,
                    rename=True, opt_out='Tuple'):
    """
    Computes sign and sign change of an array.

    Parameters
    ----------
    y : pandas.Series of float
        Array which should analyzed.

    Returns
    -------
    y_sign : pandas.Series of integer
        Signs of y.
    y_signchange : pandas.Series of boolean
        Sign changes of y.

    """
    def sign_tmp(x):
        if not x:
            o=x
        else:
            o=x // abs(x)
        return o
    def sign_ch_detect(i, std_val=False):
        if i == 0:
            o = False
        elif check_empty(i):
            o = std_val
        else:
            o = True
        return o
    if isinstance(y,np.ndarray):
        y = pd.Series(y)

    y = normalize(pdo=y, axis=axis, norm=norm, normadd=normadd)
    y = threshhold_setter(pdo=y, th=th, option=th_option, set_val=th_set_val)
    
    if isinstance(y, pd.core.base.ABCSeries):
        y_sign = y.apply(sign_tmp)
        y_signchange = y_sign.diff().apply(sign_ch_detect)
    else:
        y_sign = y.applymap(sign_tmp)
        y_signchange = y_sign.diff(axis=axis).applymap(sign_ch_detect)
        
    if rename:
        if isinstance(y, pd.core.base.ABCSeries):
            y_sign.name = y_sign.name+'_si'
            y_signchange.name = y_signchange.name+'_sc'
        else:
            if axis==0:
                y_sign.columns = y_sign.columns+'_si'
                y_signchange.columns = y_signchange.columns+'_sc'
            else:
                y_sign.index = y_sign.index+'_si'
                y_signchange.index = y_signchange.index+'_sc'
    if opt_out in ['DF','DataFrame','Dataframe']:
        out = pd.concat([y_sign,y_signchange], 
                        axis=1 if axis==0 else 0)
        return out
    elif opt_out=='Tuple':
        return y_sign, y_signchange
    else:
        raise NotImplementedError("Output option %s not implemented!"%opt_out)
        return y_sign, y_signchange


[docs]def Inter_Lines(r1,c1, r2,c2, out='x'):
    """
    Determine intersection point of two lines.

    Parameters
    ----------
    r1 : float
        Rise of first line.
    c1 : float
        Constant part of first line.
    r2 : float
        Rise of second line.
    c2 : float
        Constant part of second line.
    out : string, optional
        Option for output ('x', 'y', or 'xy'). The default is 'x'.

    Returns
    -------
    float or tuple of float
        Output values.

    """
    # if r1 == r2:
    #     raise ValueError("Lines are parallel (no intersection)!")
    #     return None
    x = (c2-c1)/(r1-r2)
    if out == 'x':
        return x
    else: 
        y = r1 * x + c1
        if out == 'y':
            return x, y
        elif out == 'xy':
            return x, y


[docs]def Line_from2P(P1, P2):
    """Determine line from two points (1st argument is x)"""
    slope = (P2[1] - P1[1]) / (P2[0] - P1[0])
    constant = P1[1] - slope * P1[0]
    return slope, constant

#%% Circle and radius/curvature from 3 points

[docs]def TP_circle(p1, p2, p3):
    """
    Returns the center and radius of the circle passing the given 3 points.
    In case the 3 points form a line, returns (None, infinity).
    """
    temp = p2[0] * p2[0] + p2[1] * p2[1]
    bc = (p1[0] * p1[0] + p1[1] * p1[1] - temp) / 2
    cd = (temp - p3[0] * p3[0] - p3[1] * p3[1]) / 2
    det = (p1[0] - p2[0]) * (p2[1] - p3[1]) - (p2[0] - p3[0]) * (p1[1] - p2[1])
    if abs(det) < 1.0e-6:
        # return (None, np.inf)
        return ((np.nan,np.nan), np.inf)
    # Center of circle
    cx = (bc*(p2[1] - p3[1]) - cd*(p1[1] - p2[1])) / det
    cy = ((p1[0] - p2[0]) * cd - (p2[0] - p3[0]) * bc) / det
    radius = np.sqrt((cx - p1[0])**2 + (cy - p1[1])**2)
    return ((cx, cy), radius)


[docs]def TP_radius(pts, outopt='signed_curvature'):
    """
    Determines the radius or curvature of a circle through three points.
    The sign indicates the direction (positve=left turn/curvature downwards).

    Parameters
    ----------
    pts : np.array or pd.DataFrame of shape (2=[x,y],3=[left,mid,right])
        Array of point coordinates.
    outopt : string, optional
        Option for output. 
        Possible are: 
            - ['signed_radius','sr']: signed radius
            - ['signed_curvature','sc']: signed curvature
            - ...: all values (Center of circle, radius, sign, quadrant to mid)
        The default is 'signed_curvature'.

    Returns
    -------
    float or tuple
        Radius, curvature or all outputs.

    """
    c,r=TP_circle(pts[0],pts[1],pts[2])
    if r==np.inf:
        m=[0,0]
    else:
        m=c-pts[[0,2]].mean(axis=1).values
    if np.sign(m[1])==1: # quadrant to determine sign of radius
        q=1 if (np.sign(m[0]) in [0,1]) else 4
    elif np.sign(m[1])==-1:
        q=2 if (np.sign(m[0]) in [0,1]) else 3
    else:
        q=1 if (np.sign(m[0]) in [0,1]) else 2
    s=1 if q in [1,4] else -1 # sign is positive if center is above mid
    if outopt in ['signed_radius','sr']:
        return r*s
    elif outopt in ['signed_curvature','sc']:
        return 1/r*s
    else:
        return (c,r,s,q)


[docs]def Geo_curve_TBC(func, params, length, outopt='signed_curvature'):
    """
    Determines the radius or curvature of a circle through three points.
    The sign indicates the direction (positve=left turn/curvature downwards).    

    Parameters
    ----------
    func : TYPE
        DESCRIPTION.
    params : TYPE
        DESCRIPTION.
    length : TYPE
        DESCRIPTION.
    outopt : string, optional
        Option for output. 
        Possible are: 
            - ['signed_radius','sr']: signed radius
            - ['signed_curvature','sc']: signed curvature
            - ...: all values (Center of circle, radius, sign, quadrant to mid)
        The default is 'signed_curvature'.

    Returns
    -------
    r : float or tuple
        Radius, curvature or all outputs.

    """
    ptx=np.array([-length/2,0,length/2])
    pty=func(ptx,params)
    pts=pd.DataFrame([ptx,pty],index=['x','y'])
    r=TP_radius(pts, outopt=outopt)
    return r
```

---

© Copyright 2024, MarcGebhardt.

Built with Sphinx using a
theme
provided by Read the Docs.
