# Supplementary material for: Approach to Standardized Material Characterization of the Human Lumbopelvic System: Testing and Evaluation
Source: Bioengineering (Basel). 2025 Aug 11;12(8):862. doi: 10.3390/bioengineering12080862 (PMC12383908; doi:10.3390/bioengineering12080862)
Supplement: Supplementary file 1 [file bioengineering-12-00862-s001.zip › File S3 Evaluation code/ExMechEva-0.1.2/docs/_build/html/_modules/exmecheva/common/eva_opt_hand.html]

exmecheva.common.eva\_opt\_hand — ExMechEva 0.1.0 documentation


ExMechEva

Contents:

- ExMechEva

ExMechEva

- Module code
- exmecheva.common.eva\_opt\_hand

---

# Source code for exmecheva.common.eva\_opt\_hand

```
# -*- coding: utf-8 -*-
"""
Contains functionality for evaluation options.

@author: MarcGebhardt
"""
import numpy as np
import pandas as pd
import json

from .helper import check_empty

[docs]def com_option_file_read(file, check_OPT=True):
    """
    Reads common options file with json and return as pandas dataframe.

    Parameters
    ----------
    file : path object or string
        Common options file location.
    check_OPT : bool, optional
        Check if variable name starts with 'OPT_'. 
        If it is true, then use for options dataframe.
        The default is True.

    Returns
    -------
    co : pd.DataFrame
        Common options dataframe.

    """
    with open(file, 'r') as f:
        co_js = json.load(f)
    co = pd.DataFrame(co_js)
    if check_OPT:
        co_io = co.index.str.startswith('OPT_')
        co = co.loc[co_io]
    return co


[docs]def com_option_file_write(file, co, indent=1):
    """
    Writes coomon options dataframe to file with json.

    Parameters
    ----------
    file : path object or string
        Common options file location.
    co : pd.DataFrame
        Common options dataframe.
    indent : int, optional
        Indent for json builder. The default is 1.

    Returns
    -------
    None.

    """
    co_d = co.to_dict()
    with open(file, 'w') as f:
        json.dump(co_d, f, indent=indent)


[docs]def set_type_by_string(o,t):
    """
    Create new object by given determiner.

    Parameters
    ----------
    o : object
        Object to convert.
    t : string
        Determiner for conversion. Implemented are:
            - "String": String object
            - "Int": Integer object
            - "Bool": Boolean object
            - "Float": Float precision object
            - "Json": Json object (uses json.loads)
            - "Free": No conversion
            - Combination of formantioned, split by '_' in descending order

    Raises
    ------
    NotImplementedError
        Given determiner/type is not implemented.

    Returns
    -------
    o : object
        Converted object.

    """
    if t=="String":
        o=str(o)
    elif t=="Int":
        o=int(o)
    elif t=="Bool":
        o=bool(o)
    elif t=="Float":
        o=float(o)
    elif t=="Json":
        o=json.loads(o)
    elif t=="Free":
        o=o
    # elif t=="Float_String":
    #     try: 
    #         o=float(o)
    #     except ValueError:
    #         o=str(o)
    elif '_' in t: #Following code may be bad practice
        for d in t.split('_'):
            try: 
                b=set_type_by_string(o,d)
            except ValueError:
                pass
            else: 
                o=b
                break
    else:
        raise NotImplementedError("Type %s not implemented!"%t)
    return o


[docs]def option_presetter(opt,mtype,preset,stype=None):
    """
    Presets option by value and types.

    Parameters
    ----------
    opt : object
        Option value.
    mtype : string
        Main type (see set_type_by_string for available types and additional "Array" with stype).
    preset : object
        Preset value.
    stype : string or None, optional
        Sub types, used if mtype == "Array" (see set_type_by_string for available types). 
        The default is None.

    Returns
    -------
    opt : object
        Presetted option value.

    """
    if check_empty(opt):
       opt=preset
    else:
        if mtype == "Array":
            j=0
            opt=str(opt).replace('"','').split(',')
            for s in stype:
                opt[j]=set_type_by_string(opt[j],s)
                j+=1
        else:
            opt=set_type_by_string(opt,mtype)
    return opt


[docs]def option_reader(options, com_opt_df, com_opts=None):
    all_inds=options.index.join(com_opt_df.index, how='outer')
    if not com_opts is None: all_inds=all_inds.join(com_opts.index, how='outer')
    # for o in options.index:
    for o in all_inds:
        if not o in options.index:
            options[o]=np.nan
        o_pres=option_presetter(opt=options[o],
                                mtype=com_opt_df.loc[o,'mtype'],
                                preset=com_opt_df.loc[o,'preset'],
                                stype=com_opt_df.loc[o,'stype'])
        if not com_opts is None:
            if (o in com_opts.index) and check_empty(options[o]):
                 options[o]=com_opts[o]
            else:
                options[o]=o_pres
        else:
            options[o]=o_pres
    return options


[docs]def option_reader_sel(prot_ser, paths, 
                      search_inds=['Number','Designation','name'], variant='',
                      option='JFile+Prot',
                      sheet_name="Eva_Options",
                      re_rkws=dict(header=3, skiprows=range(4,5), index_col=0)):
    """
    Reads in evaluation options with different methods.

    Parameters
    ----------
    prot_ser : pd.Series
        Protocoll series for measurement.
    paths : pd.Series
        Series with paths (need to have indexes 'opts' for common options and
                           'prot' if option contains 'Sheet').
    search_inds : list, optional
        List of strings to build search strings in index of option sheet 
        in protocoll excel table (excecuted in given order).
        Search strings have to be in prot_ser as variable name or an
        attribute of prot_ser (only 'name' implemented').
        The default is [].
    variant : string or str() callable, optional
        Additional addendum on search strings. Search will be exceuted after 
        other search strings (see search_inds). 
        The default is ['Number','Designation','name'].
    option : string, optional
        Implemented options for option_reader_sel.
        Implemented are:
            - 'JFile+Prot': Json file (loaded from paths['opts'] location) and 
                            direct load of evaluation options from protocoll 
                            series of measurement
            - 'JFile+Sheet': Json file (loaded from paths['opts'] location) and 
                            load of evaluation options from protocoll 
                            option sheet (see sheet_name). Search order by 
                            search_inds and combination of search_inds and variant.
        The default is 'JFile+Prot'.
    sheet_name : string, optional
        Name of evalutaion options sheet in protocoll excel table.
        The default is "Eva_Options".
    re_rkws : dict, optional
        Keyword arguments for loading evalutaion options from sheet in 
        protocoll excel table (see paths and sheet_name).
        The default is dict(header=3, skiprows=range(4,5), index_col=0).

    Raises
    ------
    ValueError
        The search string is neither in the index nor an attribute.
    NotImplementedError
        Option for selecting options not implemented.
        
    Returns
    -------
    opts_out : pd.Series
        Evaluation options.

    """
    if option in ['JFile+Prot', 'JFile+Sheet']:
        com_opt_df=com_option_file_read(file=paths['opts'], check_OPT=True)
        
    if option == 'JFile+Prot':
            opts = prot_ser[prot_ser.index.str.startswith('OPT_')]
            opts_out = option_reader(options=opts, com_opt_df=com_opt_df)
            
    elif option == 'JFile+Sheet': 
        # pandas SettingWithCopyWarning
        prot_opts = pd.read_excel(paths['prot'], sheet_name=sheet_name,**re_rkws)
        if "COMMON" in prot_opts.index:
            com_opts=option_reader(prot_opts.loc["COMMON"], com_opt_df=com_opt_df)
        else:
            com_opts=None
        if search_inds == []: #Fallback to standard
            search_names=[str(prot_ser.Number),
                          str(prot_ser.Designation),
                          str(prot_ser.name),
                          str(prot_ser.Number)+variant,
                          str(prot_ser.Designation)+variant,
                          str(prot_ser.name)+variant]
        else:
            search_names=[]
            snv=[]
            for i in search_inds: #Listcomprehension not working for buil in attributes [str(s[x]) for x in a]
                if not i in ['name']:
                    iap=str(i)
                else:
                    if i == 'name':
                        iap=str(prot_ser.name)
                    else:
                        raise ValueError('The search string %s is neither in the index nor an attribute!'%i)
                search_names.append(iap)
                if variant != '': snv.append(iap+str(variant))
            if snv != []: search_names=search_names+snv
        for s in search_names:
            if s in prot_opts.index:
                opts=prot_opts.loc[s]
            else:
                opts=pd.Series(['',]*len(prot_opts.columns),
                                index=prot_opts.columns, dtype='O')
            com_opts=option_reader(options=opts, com_opt_df=com_opt_df,
                                   com_opts=com_opts)
        opts_out=com_opts
    else:
        raise NotImplementedError('Option for selcting options not implemented!'%option)
    return opts_out
```

---

© Copyright 2024, MarcGebhardt.

Built with Sphinx using a
theme
provided by Read the Docs.
