# Supplementary material for: Approach to Standardized Material Characterization of the Human Lumbopelvic System: Testing and Evaluation
Source: Bioengineering (Basel). 2025 Aug 11;12(8):862. doi: 10.3390/bioengineering12080862 (PMC12383908; doi:10.3390/bioengineering12080862)
Supplement: Supplementary file 1 [file bioengineering-12-00862-s001.zip › File S3 Evaluation code/ExMechEva-0.1.2/docs/_build/html/_modules/exmecheva/common/fitting.html]

exmecheva.common.fitting — ExMechEva v0.1.1 documentation


ExMechEva

Contents:

- ExMechEva

ExMechEva

- Module code
- exmecheva.common.fitting

---

# Source code for exmecheva.common.fitting

```
# -*- coding: utf-8 -*-
"""
Created on Mon Dec  4 17:43:14 2023

@author: mgebhard
"""
import warnings

import numpy as np
import pandas as pd
import lmfit

from .pd_ext import (pd_combine_index)
#%% General functions with string equations

[docs]def func_lin(x, a, b):
    """Return values from a general linear function."""
    return a + b * x


[docs]def func_lin_str(xl, yl, 
                 a, b,
                 t_form='{a:.3e},{b:.3e}'):
    """Return string from a general linear function."""
    txtc = t_form.format(**{'a':a,'b':b},).split(',')
    if a==0:
        txt = r'${y}\,=\,{0} \cdot {x}$'.format(
			txtc[1],**{'y':yl,'x':xl},
			)
    else:
        if b>=0:
            txt = r'${y}\,=\,{0}\,+\,{1} \cdot {x}$'.format(
                txtc[0],txtc[1],**{'y':yl,'x':xl},
                )
        else:
            txtc[1]=txtc[1][1:]
            txt = r'${y}\,=\,{0}\,-\,{1} \cdot {x}$'.format(
                txtc[0],txtc[1],**{'y':yl,'x':xl},
                )
    return txt


[docs]def func_exp(x, a, b, c):
    """Return values from a general exponential function."""
    return a + b * np.exp(c * x)


[docs]def func_exp_str(xl, yl, 
                 a, b, c, 
                 t_form='{a:.3e},{b:.3e},{c:.3e}'):
    """Return string from a general exponential function."""
    txtc = t_form.format(**{'a':a,'b':b,'c':c},).split(',')
    txtd = '{0}\,{1}'.format(txtc[2],xl,)
    if a==0:
        txt = r'${y}\,=\,{0} \cdot e^{1}{2}{3}$'.format(
			txtc[1],'{',txtd,'}',**{'y':yl},
			)
    elif a==(-b):
        txt = r'${y}\,=\,{0} \cdot (e^{1}{2}{3}-1)$'.format(
			txtc[1],'{',txtd,'}',**{'y':yl},
			)
    else:
        txt = r'${y}\,=\,{0}\,+\,{1} \cdot e^{2}{3}{4}$'.format(
			txtc[0],txtc[1],'{',txtd,'}',**{'y':yl},
			)
    return txt


[docs]def func_pow(x, a, b, c):
    """Return values from a general power function."""
    return a + b * x**(c)


[docs]def func_pow_str(xl, yl, 
                 a, b, c, 
                 t_form='{a:.3e},{b:.3e},{c:.3e}'):
    """Return string from a general power function."""
    txtc = t_form.format(**{'a':a,'b':b,'c':c},).split(',')
    if a==0:
        txt = r'${y}\,=\,{0} \cdot {x}^{1}{2}{3}$'.format(
                txtc[1],'{',txtc[2],'}',**{'x':xl,'y':yl},
                )
    else:
        if b>=0:
            txt = r'${y}\,=\,{0}\,+\,{1} \cdot {x}^{2}{3}{4}$'.format(
                txtc[0],txtc[1],'{',txtc[2],'}',**{'x':xl,'y':yl},
                )
        else:
            txtc[1]=txtc[1][1:]
            txt = r'${y}\,=\,{0}\,-\,{1} \cdot {x}^{2}{3}{4}$'.format(
                txtc[0],txtc[1],'{',txtc[2],'}',**{'x':xl,'y':yl},
                )
    return txt

#%% Fitting

[docs]def regfitret(pdo, x,y, name='linear', guess = dict(a=0.01, b=0.1),
              xl=r'$X$', yl=r'$Y$', t_form='{a:.3e},{b:.3e}',
              xt=None, yt=None,
			  max_nfev=1000, nan_policy='omit', outtype='Series'):
    """
    Performs a least square regression fit according given function type.

    Parameters
    ----------
    pdo : pd.DataFrame
        Input data.
    x : str
        Column name for abscissa data.
    y : str
        Column name for ordinate data.
    name : str, optional
        Type and name of function to adjust. 
        Implemented are:
            - 'linear': linear and constant function (see func_lin)
            - 'power': power and constant function (see func_pow)
            - 'exponential': exponantial and constant function (see func_exp)
            - additional endings:
                - '_nc': no constant value
                - '_x0': fixed to zero
        The default is 'linear'.
    guess : dictionary, optional
        First guess for fitting. The default is dict(a=0.01, b=0.1).
    xl : str, optional
        Variable name for abscissa. The default is r'$X$'.
    yl : str, optional
        Variable name for ordinate. The default is r'$Y$'.
    t_form : str, optional
        String of dictionary with variables and format strings. 
        The default is '{a:.3e},{b:.3e}'.
    xt : str, optional
        Variable string for description. The default is None.
    yt : str, optional
        Variable string for description. The default is None.
    max_nfev : int, optional
        Maximum number of evaluations. The default is 1000.
    nan_policy : string, optional
        NaN policy (omit, raise or propagate). The default is 'omit'.
    outtype : string, optional
        Type for output (dictionary or pd.Series). The default is 'Series'.

    Raises
    ------
    NotImplementedError
        Function type not implemented.

    Returns
    -------
    out : pd:series or dict
        Output data.

    """
    def des_t_name(inp):
        for i in inp:
            if not i is None: out=i
        return out
    xd = des_t_name(['x',x,xl,xt])
    yd = des_t_name(['y',y,yl,yt])
    if name in ['linear','linear_nc','linear_x0']:
        dtxt='Linear relation between {} and {}'.format(yd,xd)
        func=func_lin
        func_str=func_lin_str
    elif name in ['power','power_nc','power_x0']:
        dtxt='Power relation between {} and {}'.format(yd,xd)
        func=func_pow
        func_str=func_pow_str
    elif name in ['exponential','exponential_nc','exponential_x0']:
        dtxt='Exponential relation between {} and {}'.format(yd,xd)
        func=func_exp
        func_str=func_exp_str
    else:
        raise NotImplementedError('Type %s not implemented!'%name)
    if name.endswith('_nc'):
        dtxt+= ' (no constant value)'
    elif name.endswith('_x0'):
        dtxt+= ' (fixed to zero)'
    model=lmfit.Model(func)
    params=model.make_params()
    if name in ['linear_nc','power_nc','exponential_nc']:
        params.add('a', value=0, vary=False)
    elif name in ['linear_x0','power_x0']:
        params.add('a', value=guess['a'], vary=False)
    elif name in ['exponential_x0']:
        y0=guess.pop('a')
        params.add('a', value=None, expr="{:.8e}-b".format(y0), vary=False)
    fit = model.fit(pdo[y], x=pdo[x], params=params, nan_policy=nan_policy, **guess)
    Rquad = 1 - fit.residual.var() / np.nanvar(pdo[y])     
    fit.rsqur=Rquad
    # rtxt=lmfit.fit_report(fit.result)
    # rtxt=rtxt.replace("\n[[Variables]]",
    #                   "\n    R-square           = %1.8f\n[[Variables]]"%Rquad)
    rtxt=fit_report_adder(fit,Rquad, show_correl=True)
    etxt=func_str(xl=xl, yl=yl, t_form=t_form, **fit.result.params.valuesdict())
    out = {'Name': name, 'Model': model, 'Result': fit, 
           'PD': fit.result.params.valuesdict(),
		   'Description': dtxt,
           'Exp_txt': etxt, 'Rep_txt': rtxt, 'Rquad': Rquad}
    if outtype=='Series': out=pd.Series(out)
    return out


[docs]def regfitret_restring_func(reg_res, xl=r'$X$', yl=r'$Y$',
                            t_form='{a:.3e},{b:.3e}',
                            rquad_add=True, rq_form='{:.3f}'):
    """
    Rebuild equation string for regfit result.

    Parameters
    ----------
    reg_res : pd.Series
        Result of regfitret.
    xl : str, optional
        Variable name for abscissa. The default is r'$X$'.
    yl : str, optional
        Variable name for ordinate. The default is r'$Y$'.
    t_form : str, optional
        String of dictionary with variables and format strings. 
        The default is '{a:.3e},{b:.3e}'.
    rquad_add : bool, optional
        Switch for adding coefficent of determination. The default is True.
    rq_form : string, optional
        Format string for coefficent of determination. The default is '{:.3f}'.

    Raises
    ------
    NotImplementedError
        Function type not implemented.

    Returns
    -------
    out : string
        Equation string.

    """
    if reg_res['Name'] in ['linear','linear_nc','linear_x0']:
        func_str=func_lin_str
    elif reg_res['Name'] in ['power','power_nc','power_x0']:
        func_str=func_pow_str
    elif reg_res['Name'] in ['exponential','exponential_nc','exponential_x0']:
        func_str=func_exp_str
    else:
        raise NotImplementedError('Type %s not implemented!'%reg_res['Name'])
    if xl is None: xl=reg_res['varxsym']
    if yl is None: yl=reg_res['varysym']
    out=func_str(xl=xl, yl=yl, t_form=t_form, **reg_res['PD'])
    if rquad_add:
        out+=' ($R²$ = '
        out+=(rq_form+')').format(reg_res['Rquad'])
    return out


[docs]def YM_sigeps_lin(stress_ser, strain_ser, 
                  method='leastsq', nan_policy='omit',
                  ind_S=None, ind_E=None):
    """
    Calculates Youngs Modulus of a stress-strain-curve with linear approach 
    using non linear least squares curve fitting.

    Parameters
    ----------
    strain_col : pd.Series
        Strain curve.
    stress_col : pd.Series
        Stress_curve.
    ind_S : integer
        First used index in df.
    ind_E : integer
        Last used index in df.

    Returns
    -------
    E : float
        Youngs Modulus (corresponds to slope of linear fit).
    Eabs : float
        Stress value on strain origin (corresponds to interception of linear fit).
    Rquad : float
        Coefficient of determination.
    fit : lmfit.model.ModelResult
        Fitting result from lmfit (use with fit.fit_report() for report).

    """
    if not (ind_S is None):
        stress_ser = stress_ser.loc[ind_S:]
        strain_ser = strain_ser.loc[ind_S:]
    if not (ind_E is None):
        stress_ser = stress_ser.loc[:ind_E]
        strain_ser = strain_ser.loc[:ind_E]
        
    try:
        m       = lmfit.models.LinearModel()
        # guess doesn't work with NaN's
        # pg      = m.guess(stress_ser, x=strain_ser)
        # fit     = m.fit(stress_ser, pg, x=strain_ser,
        #                 method=method, nan_policy=nan_policy)
        fit     = m.fit(stress_ser, x=strain_ser,
                        method=method, nan_policy=nan_policy)
        E       = fit.params.valuesdict()['slope']
        Eabs    = fit.params.valuesdict()['intercept']
        # Rquad    = 1 - fit.residual.var() / np.var(stress_ser)
        Rquad    = 1 - fit.residual.var() / np.nanvar(stress_ser)
    except np.linalg.LinAlgError:
        warnings.warn("SVD did not converge in Linear Least Squares, all values set to NaN.")
        E, Eabs, Rquad, fit = np.nan,np.nan,np.nan,'SVD-Error'
    return E, Eabs, Rquad, fit


[docs]def fit_report_adder(fit, Var, Varname='R-square', show_correl=False):
    """
    Adds a statistical entry to the fit report of lmfit (p.e. coefficient of 
    determination). 

    Parameters
    ----------
    fit : string or lmfit.model.ModelResult
        Fit report.
    Var : float
        Value of addional statistics entry.
    Varname : string, optional
        Name of addional statistics entry. The default is 'R-square'.
    show_correl : bool, optional
        Show or hide correlation. The default is False.

    Raises
    ------
    TypeError
        Fit has wrong type.

    Returns
    -------
    txt : string
        Fit report with additional statistics value.

    """
    if isinstance(fit, str):
        fit = fit
    elif isinstance(fit, lmfit.model.ModelResult):
        fit=fit.fit_report(show_correl=show_correl)
    else:
        raise TypeError('Datatype %s is not implemented!'%(type(fit)))
    orgstr="\n[[Variables]]"
    repstr="\n    {0:18s} = {1:1.5f}\n[[Variables]]".format(*[Varname,Var])
    txt=fit.replace(orgstr,repstr)
    return txt


[docs]def YM_eva_com_sel(stress_ser,
                   strain_ser,
                   comp=True, name='A', 
                   det_opt='incremental',**kws):
    """
    Calculates Young's Modulus over defined range with definable method.

    Parameters
    ----------
    stress_ser : pd.Series
        Series with stress values corresponding strain_ser.
    strain_ser : pd.Series
        Series with strain values corresponding stress_ser.
    comp : boolean, optional
        Compression mode. The default is True.
    name : string, optional
        Name of operation. The default is 'A'.
    det_opt : TYPE, optional
        Definable method for determination.
        Ether incremental or leastsq. The default is 'incremental'.
    **kws : dict
        Keyword dict for least-square determination.

    Returns
    -------
    det_opt == "incremental":
        YM_ser : pd.Series
            Series of Young's Moduli.
    or
    det_opt == "leastsq":
        YM : float
            Youngs Modulus (corresponds to slope of linear fit).
        YM_abs : float
            Stress value on strain origin (corresponds to interception of linear fit).
        YM_Rquad : float
            Coefficient of determination.
        YM_fit : lmfit.model.ModelResult
            Fitting result from lmfit (use with fit.fit_report() for report).
    """
    step_range=pd_combine_index(stress_ser, strain_ser)
    stress_ser = stress_ser.loc[step_range]
    strain_ser = strain_ser.loc[step_range]
    if det_opt=="incremental":
        YM_ser = stress_ser / strain_ser
        YM_ser = pd.Series(YM_ser, name=name)
        return YM_ser
    elif det_opt=="leastsq":
        YM, YM_abs, YM_Rquad, YM_fit = YM_sigeps_lin(stress_ser,
                                                     strain_ser, **kws)
        return YM, YM_abs, YM_Rquad, YM_fit


[docs]def Refit_YM_vals(m_df, YM, VIP, n_strain='Strain', n_stress='Stress',
              n_loBo=['F3'], n_upBo=['F4'], option='range', outopt='Series'):
    """
    Refits line values for given rising (i.e. absolute value and R² vor given elastic modulus)

    Parameters
    ----------
    m_df : pd.DataFrame
        Measured data.
    YM : float
        Youngs Modulus (fixed rising of line).
    VIP : pd.Series
        Important points corresponding to measured data.
    n_strain : string
        Name of used strain (have to be in measured data).
    n_stress : string
        Name of used stress (have to be in measured data).
    n_loBo : [string]
        List of lower borders for determination (have to be in VIP). The default is ['F3'].
    n_upBo : [string]
        List of upper borders for determination (have to be in VIP). The default is ['F4'].
    option : string, optional
        Determination range. The default is 'range'.
    outopt : string, optional
        Switch for outupt (Series or list). The default is 'Series'.

    Returns
    -------
    out : list or pd.Series
        Line values and firt parameters for fixed rising.

    """
    m_lim = m_df.loc[min(VIP[n_loBo]):max(VIP[n_upBo])]
    if option == 'range':
        fitm = lmfit.models.LinearModel()
        fitpar = fitm.make_params()
        fitpar.add('slope', value=YM, vary = False)
        fit=fitm.fit(m_lim[n_stress], x=m_lim[n_strain], 
                     params=fitpar,method='leastsq', nan_policy='omit')
        E       = fit.params.valuesdict()['slope']
        Eabs    = fit.params.valuesdict()['intercept']
        Rquad    = 1 - fit.residual.var() / np.nanvar(m_lim['Stress'])
    out=E, Eabs, Rquad, fit
    if outopt=='Series':
        out= pd.Series(out, index=['E','E_abs','Rquad','Fit_result'])
    return out


[docs]def Rquad(y_true, y_predicted, nan_policy='omit'):
    """
    Returns coefficient of determination.

    Parameters
    ----------
    y_true : array of float
        True values.
    y_predicted : array of float
        Predicted values.
    nan_policy : bool, optional
        NaN policy (omit, raise or propagate). The default is 'omit'.

    Raises
    ------
    ValueError
        NaN values during Rquad detected (nan_policy is raise).
    NotImplementedError
        NaN policy type not implemented.

    Returns
    -------
    r2_score : TYPE
        Coefficient of determination.

    """
    if nan_policy == 'omit':
        sse = np.nansum((y_true - y_predicted)**2)
        y_t_l_woNan = len(y_true) - np.count_nonzero(np.isnan(y_true))
        tse = (y_t_l_woNan - 1) * np.nanvar(y_true, ddof=1)
        r2_score = 1 - (sse / tse)
        return r2_score
    elif nan_policy == 'raise':
        if np.isnan(y_true).any() or np.isnan(y_predicted):
            raise ValueError("NaN values during Rquad detected")
    else:
        if nan_policy != 'propagate':
            raise NotImplementedError("NaN policy %s not implemented"%nan_policy)
    sse = np.sum((y_true - y_predicted)**2)
    tse = (len(y_true) - 1) * np.var(y_true, ddof=1)
    r2_score = 1 - (sse / tse)
    return r2_score


[docs]def stress_linfit(strain_ser, YM, YM_abs, strain_offset=0.002):
    """Linearised stress fit corresponding Youngs Modulus and strain"""
    stress_fit_ser=(strain_ser - strain_offset) * YM + YM_abs
    return stress_fit_ser


[docs]def stress_linfit_plt(strain_ser, inds, YM, YM_abs, strain_offset=0, ext=0.1):
    """Linearised stress fit for plotting corresponding Youngs Modulus and strain"""
    strain_vals = strain_ser.loc[inds].values
    strain_vals = np.array([
        max(0,strain_vals[0]-ext*(strain_vals[-1]-strain_vals[0])),
        min(strain_ser.max(),strain_vals[-1]+ext*(strain_vals[-1]-strain_vals[0]))
        ])
    stress_vals = stress_linfit(strain_vals, YM, YM_abs, strain_offset)
    return strain_vals, stress_vals


[docs]def strain_linfit(stress_ser, YM, YM_abs, strain_offset=0.002):
    """Linearised strain fit corresponding Youngs Modulus and stress"""
    strain_fit_ser=(stress_ser - YM_abs) / YM + strain_offset
    return strain_fit_ser
```

---

© Copyright 2024, MarcGebhardt.

Built with Sphinx using a
theme
provided by Read the Docs.
