# Supplementary material for: Approach to Standardized Material Characterization of the Human Lumbopelvic System: Testing and Evaluation
Source: Bioengineering (Basel). 2025 Aug 11;12(8):862. doi: 10.3390/bioengineering12080862 (PMC12383908; doi:10.3390/bioengineering12080862)
Supplement: Supplementary file 1 [file bioengineering-12-00862-s001.zip › File S3 Evaluation code/ExMechEva-0.1.2/docs/_build/html/_modules/exmecheva/common/helper.html]

exmecheva.common.helper — ExMechEva 0.1.0 documentation


ExMechEva

Contents:

- ExMechEva

ExMechEva

- Module code
- exmecheva.common.helper

---

# Source code for exmecheva.common.helper

```
# -*- coding: utf-8 -*-
"""
Contains test and basic numeric functionality.

@author: MarcGebhardt
"""
import warnings
import numpy as np
import pandas as pd

#%% tests
def _test_overwrites(impmods, overwr):
    """
    Tests if function in module. Use while importing.

    Parameters
    ----------
    impmods : list, optional
        Modules to search for function overwrites. The default is [].
    overwr : list, optional
        Function overwrites to check in modules. The default is [].

    Raises
    ------
    ImportWarning
        Function in module and will be overwritten.

    """
    def funcinmod(func, mod):
        if str(func) in dir(mod):
            o = True
        else:
            o = False
        return o
    
    # _overrides=["indlim","meanwoso","stdwoso"]
    # _impmods=[pd.Series,pd.DataFrame]
    for ta in overwr:
        for tb in impmods:
            if funcinmod(func=ta, mod=tb):
                warnings.warn("Function %s in %s and will be overwritten!"%(ta,tb),
                              ImportWarning)

[docs]def type_str_return(obj):
    """
    Tests type of given object and return an identifying string.

    Parameters
    ----------
    obj : object
        Input object to check type.
        Implemented are:
            - Standard: None, bool, str, int and float
            - Numpy array of shape:
                - unknown shape -> npAu
                - single row -> npAr
                - single column -> npAc
                - frame -> npAF
            - Pandas:
                - Index -> pdIN
                - Series -> pdSe
                - DataFrame-> pdDF

    Raises
    ------
    NotImplementedError
        Type of object not implemented.
        
    Returns
    -------
    t : str
        String of derived type.

    """
    if obj is None:
        t='None'
    elif isinstance(obj, bool):
        t='bool'
    elif isinstance(obj, str):
        t='str'
    elif isinstance(obj, int):
        t='int'
    elif isinstance(obj, float):
        t='float'
    elif isinstance(obj, np.ndarray):
        t='npAu'
        npshape=obj.shape
        if np.size(npshape)==1:
            t='npAr'
        elif np.size(npshape)==2:
            if npshape[0]==1:
                t='npAc'
            else:
                t='npAF'
    elif isinstance(obj, pd.core.base.ABCIndex):
        t='pdIn'
    elif isinstance(obj, pd.core.base.ABCSeries):
        t='pdSe'
    elif isinstance(obj, pd.core.base.ABCDataFrame):
        t='pdDF'
    else:
        raise NotImplementedError("Type {} not implemented!".format(type(obj)))
    return t


[docs]def check_empty(x, empty_str=['',' ','  ','   ',
                              '#NV''NaN','nan','NA']):
    """
    Tests if given variable (string or float) can interpreted as empty.

    Parameters
    ----------
    x : None or string or float
        Given variable to test.
    empty_str : list of strings
        Strings determining if variable can interpreted as empty.

    Returns
    -------
    t : bool
        Test result.

    """
    t = (x is None or 
         x in empty_str or
         (isinstance(x, float) and  np.isnan(x)))
    return t


[docs]def str_to_bool(x, str_true=["True","true","Yes","1","On"]):
    """
    Interpretes a given string as boolean value

    Parameters
    ----------
    x : string or bool
        Value to be interpreted.
    str_true : list of strings
        List of strings interpreted as True.

    Returns
    -------
    t : bool
        Boolean value result.

    """
    
    if x is True:
        t = x
    elif x in str_true:
        t = True
    else:
        t = False
    return t

#%% Numeric

[docs]def sigdig(x, sd=3):
    """
    Returns the significant digits of a floating number.

    Parameters
    ----------
    x : float
        Number.
    sd : int, optional
        Significant digits. The default is 3.

    Returns
    -------
    xr : float
        Rounded number to significant digits.

    """
    # import math
    # ndig = int(math.floor(math.log10(abs(x))))
    if ((x==0) or (abs(x)==np.inf) or (np.isnan(x))):
        ndig = 0
    else:
        ndig = int(np.floor(np.log10(abs(x))))
    rdig = sd - ndig - 1
    return rdig


[docs]def round_to_sigdig(x, sd=3):
    """
    Round a floating number to a number of significant digits.

    Parameters
    ----------
    x : float
        Number.
    sd : int, optional
        Significant digits. The default is 3.

    Returns
    -------
    xr : float
        Rounded number to significant digits.

    """
    rdig = sigdig(x, sd)
    xr =  round(x, rdig)
    return xr
```

---

© Copyright 2024, MarcGebhardt.

Built with Sphinx using a
theme
provided by Read the Docs.
