# Supplementary material for: Approach to Standardized Material Characterization of the Human Lumbopelvic System: Testing and Evaluation
Source: Bioengineering (Basel). 2025 Aug 11;12(8):862. doi: 10.3390/bioengineering12080862 (PMC12383908; doi:10.3390/bioengineering12080862)
Supplement: Supplementary file 1 [file bioengineering-12-00862-s001.zip › File S3 Evaluation code/ExMechEva-0.1.2/docs/_build/html/_modules/exmecheva/common/list_ops.html]

exmecheva.common.list\_ops — ExMechEva 0.1.0 documentation


ExMechEva

Contents:

- ExMechEva

ExMechEva

- Module code
- exmecheva.common.list\_ops

---

# Source code for exmecheva.common.list\_ops

```
# -*- coding: utf-8 -*-
"""
List manipulation and interpretation functionality.

@author: MarcGebhardt
"""
import re
import numpy as np
import pandas as pd

#%% List Operations

[docs]def list_cell_compiler(ser,sep=',', replace_whitespaces=True, replace_nans=True):
    ser = ser.astype(str)
    if replace_whitespaces:
        ser = ser.agg(lambda x: x.replace(' ',''))
    ser = ser.agg(lambda x: x.split(sep))
    return ser


[docs]def list_interpreter(ser, inter_list, option = 'exclude'):
    if option == 'exclude':
        func = lambda x: False if len(set(x).intersection(set(inter_list)))>0 else True
    elif option == 'include':
        func = lambda x: True if len(set(x).intersection(set(inter_list)))>0 else False
    else:
        raise NotImplementedError('Option %s not implemented!'%option)
    bool_ser = ser.agg(func)
    return bool_ser


[docs]def list_ser_to_1D(series, drop_duplicates=True, 
                   sort_values=True, exclude=[]):
    new = pd.Series([x for _list in series for x in _list])
    if drop_duplicates: new = new.drop_duplicates()
    if sort_values: new = new.sort_values().values
    for e in exclude:
        # new = np.delete(new,e)
        new = np.delete(new,new==e)
    return new


[docs]def list_boolean_df(series, unique_items, as_int=False):
    # Create empty dict
    bool_dict = {}
    # Loop through all the tags
    for i, item in enumerate(unique_items):
        # Apply boolean mask
        bool_dict[item] = series.apply(lambda x: item in x)
    # Return the results as a dataframe
    df = pd.DataFrame(bool_dict)
    if as_int: df = df.astype(int)
    return df

#%% Failure Codes ~ Assessment codes

[docs]def Failure_code_format(fcstr, pattern="[A-Z][0-9][0-9][.][0-3]"):
    matched = re.match(pattern, fcstr)
    fc_b=bool(matched)
    return fc_b


[docs]def Failure_code_checker(fc_ser, exclude =['nan'], pattern="[A-Z][0-9][0-9][.][0-3]"):
    fcu=pd.Series(list_ser_to_1D(fc_ser,**{'drop_duplicates':True,
                                           'sort_values':True,
                                           'exclude':exclude}))
    fc_wrong   = fcu[fcu.apply(Failure_code_format)==False].values.tolist()
    fc_wrongid = list_interpreter(fc_ser, fc_wrong, option = 'include')
    fc_wrongid = fc_ser[fc_wrongid]
    return fc_wrongid, fc_wrong


[docs]def Failure_code_lister(_list, level=1, strength=[1,2,3],
                        drop_duplicates=True, replace_whitespaces=True):
    """ Shortens List of Failure-codes (Version 3.1) to levels and optional strength
    Level: 0=Procedure, 1=Procedure Failure_Type, 2=Porcedure.Failure_Type.Strength
           'P'=Procedure, 'F'=Failure_Type 
    """
    
    strength = [str(x) for x in strength]
    
    if replace_whitespaces:
        _list = [x.replace(' ','') for x in _list]
    if strength:
        _list = [i for i in _list if i.split('.')[-1] in strength]
        
    if level == 0 or level == 'P':
        new = [x[0] for x in _list]
    elif level == 1:
        new = [x.split('.')[0] for x in _list]
    elif level == 2:
        new = [x for x in _list]
    elif level == 'F':
        new = [x.split('.')[0][1:] for x in _list]
        
    if drop_duplicates:
        new = list(dict.fromkeys(new))
    return new


[docs]def Failure_code_bool_df(list_ser, sep=',', level=1, strength=[1,2,3],
                         drop_duplicates=True, sort_values=True,
                         exclude =['nan'],
                         replace_whitespaces=True, as_int=True):
    # list_ser = list_cell_compiler(series,sep,replace_whitespaces)
    list_ser = list_ser.apply(Failure_code_lister,**{'level':level,'strength':strength,
                                                     'drop_duplicates':drop_duplicates,
                                                     'replace_whitespaces':replace_whitespaces})
    uniques = list_ser_to_1D(list_ser,**{'drop_duplicates':drop_duplicates,
                                         'sort_values':sort_values,
                                         'exclude':exclude})
    df = list_boolean_df(list_ser,uniques,**{'as_int':as_int})
    return df, uniques

#%% ICD Codes

[docs]def ICD_lister(_list, level=1,
               drop_duplicates=True, replace_whitespaces=True):
    """ Shortens List of ICD-codes to levels """
    if replace_whitespaces:
        _list = [x.replace(' ','') for x in _list]
    if level == 0:
        new = [x[0] for x in _list]
    elif level == 1:
        new = [x.split('.')[0] for x in _list]
    elif level == 2:
        new = [x for x in _list]
    if drop_duplicates:
        new = list(dict.fromkeys(new))
    return new


[docs]def ICD_bool_df(series, sep=',', level=1,
                drop_duplicates=True, sort_values=True,
                exclude =[],
                replace_whitespaces=True, as_int=True):
    list_ser = list_cell_compiler(series,sep,replace_whitespaces)
    list_ser = list_ser.apply(ICD_lister,**{'level':level,
                                            'drop_duplicates':drop_duplicates,
                                            'replace_whitespaces':replace_whitespaces})
    uniques = list_ser_to_1D(list_ser,**{'drop_duplicates':drop_duplicates,
                                         'sort_values':sort_values,
                                         'exclude':exclude})
    df = list_boolean_df(list_ser,uniques,**{'as_int':as_int})
    return df
```

---

© Copyright 2024, MarcGebhardt.

Built with Sphinx using a
theme
provided by Read the Docs.
