# Supplementary material for: Approach to Standardized Material Characterization of the Human Lumbopelvic System: Testing and Evaluation
Source: Bioengineering (Basel). 2025 Aug 11;12(8):862. doi: 10.3390/bioengineering12080862 (PMC12383908; doi:10.3390/bioengineering12080862)
Supplement: Supplementary file 1 [file bioengineering-12-00862-s001.zip › File S3 Evaluation code/ExMechEva-0.1.2/docs/_build/html/_modules/exmecheva/common/loadnsave.html]

exmecheva.common.loadnsave — ExMechEva 0.1.0 documentation


ExMechEva

Contents:

- ExMechEva

ExMechEva

- Module code
- exmecheva.common.loadnsave

---

# Source code for exmecheva.common.loadnsave

```
# -*- coding: utf-8 -*-
"""
Contains functionality for loading and saving files.

@author: MarcGebhardt
"""
import os
import numpy as np
import pandas as pd

[docs]def file_namer(fstr, svars='>', svare='<', sform='#'):
    """
    Names a file according given string (sub module of File_namer_interpreter).

    Parameters
    ----------
    fstr : string
        String with replaceable tokens.
    svars : string, optional
        Start character for token. The default is '>'.
    svare : string, optional
        End character for token. The default is '<'.
    sform : string, optional
        Form determinator at end of token. The default is '#'.

    Returns
    -------
    vardf : TYPE
        DESCRIPTION.
        
    Examples
    --------
    - >Number#02<>Variant<.xlsx:
        with Number = 05 (from protocol) and Variant = C (from call) 
        -> 05C.xlsx
    - >Designation<_>Variant<_foo.csv:
        with (from protocol) Designation = Test_1 and Variant = B 
        -> Test_1_B_foo.csv

    """
    if '.' in fstr:
        fstr,fdend=fstr.split('.')
        fdend='.'+fdend
    else:
        fdend=''
    vardf=pd.DataFrame([],columns=['Value','IsVar','HasForm','Form','IsEnd'],dtype='O')
    j=0
    if svars in fstr:
        fstr = fstr.split(svars)
        for fsub in fstr:
            value=fsub
            isvar=False
            hasform=False
            form=''
            if fsub.endswith(svare):
                isvar=True
                if '#' in fsub:
                    hasform=True
                    value,form=fsub.split('#')
                    form=form.replace(svare,'')
                else:
                    value=fsub.replace(svare,'')
            vardf.loc[j]=pd.Series({'Value':value,'IsVar':isvar,
                                    'HasForm':hasform,'Form':form,'IsEnd':False})
            j+=1
    else:
        vardf.loc[j]=pd.Series({'Value':fstr,'IsVar':False,
                                'HasForm':False,'Form':'','IsEnd':False})
    if not fdend == '':
        vardf.loc[j+1]=pd.Series({'Value':fdend,'IsVar':False,
                                  'HasForm':False,'Form':'','IsEnd':True})
    return vardf


[docs]def file_namer_interpreter(fstr, prot_ser, path, variant='',
                           expext='.xlsx', svars='>', svare='<', sform='#'):
    """
    Builds a file location according given string (with repleacable tokens).

    Parameters
    ----------
    fstr : string
        String with replaceable tokens.
    prot_ser : pd.Series
        Series form protocoll dataframe with tokens as index.
    path : string
        Path addendum (will be added before adjusted filename).
    variant : string, optional
        Variant (special string for token 'Variant'). The default is ''.
    expext : string, optional
        Expected extension of file. The default is '.xlsx'.
    svars : string, optional
        Start character for token. The default is '>'.
    svare : string, optional
        End character for token. The default is '<'.
    sform : string, optional
        Form determinator at end of token. The default is '#'.

    Raises
    ------
    ValueError
        Token not found in protocoll.

    Returns
    -------
    fname : string
        Adjusted file name.
        
    Examples
    --------
    - >Number#02<>Variant<.xlsx:
        with Number = 05 (from protocol) and Variant = C (from call) 
        -> 05C.xlsx
    - >Designation<_>Variant<_foo.csv:
        with (from protocol) Designation = Test_1 and Variant = B 
        -> Test_1_B_foo.csv
    """
    var_df=file_namer(fstr=fstr,svars=svars,svare=svare,sform=sform)
    fname=path
    for i in var_df.index:
        if var_df.loc[i,'IsEnd']: 
            fdend=var_df.loc[i,'Value']
        else:
            fdend=expext
        if var_df.loc[i,'IsVar']:
            if var_df.loc[i,'Value'] in prot_ser.index:
                val=prot_ser[var_df.loc[i,'Value']]
                if var_df.loc[i,'HasForm']:
                    fname+=("{:%s}"%var_df.loc[i,'Form']).format(val)
                else:
                    fname+=str(val)
            elif var_df.loc[i,'Value'] == 'Variant':
                val=variant
                if var_df.loc[i,'HasForm']:
                    fname+=("{:%s}"%var_df.loc[i,'Form']).format(val)
                else:
                    fname+=str(val)                
            else:
                raise ValueError("%s not in protocol!"%var_df.loc[i,'Value'])
        elif not var_df.loc[i,'IsEnd']:
            fname+=var_df.loc[i,'Value']
    fname+=fdend
    return fname

#%% Log-files

[docs]def comb_logs(in_paths, out_path):
    """
    Generates a combined log-file of a series of log-files.

    Parameters
    ----------
    in_paths : pd.DataFrame with columns ['prot','out']
        Path for finding protocol and evaluation data.
    out_path : string
        Output path of combined log-file (without extension).

    Returns
    -------
    None.

    """
    lps = in_paths['out'] + in_paths['prot'].apply(
        lambda x: os.path.basename(x).replace('.xlsx','.log')
        )
    txt=''
    for i in lps.index:
        txt+='{}:\n({})'.format(i,lps.loc[i])
        l=open(lps.loc[i],"r")
        txt+=''.join(l.readlines())
        l.close()
        txt+='\n'+'-'*100+'\n'
    comblog = open(out_path+'.log','w')
    comblog.write(txt)
    comblog.close()

#%% HDF

[docs]def pack_hdf(in_paths, out_path, 
             hdf_naming = 'Designation', var_suffix = [""],
             h5_conc = 'Material_Parameters', h5_data = 'Measurement',
             prot_rkws=dict(header=11, skiprows=range(12,13), index_col=0),
             opt_pd_out = True, opt_hdf_save = True):
    """
    Packs specimenwise evaluated measurements (HDF-files, *.h5) to a 
    HDF-database containing material parameters (key is 'Summary') and 
    evaluated measurements (key is 'Test_Data').

    Parameters
    ----------
    in_paths : pd.DataFrame with columns ['prot','hdf']
        Path for finding protocol and evaluation data.
    out_path : string
        Output path of created database.
    hdf_naming : string, optional
        Naming rule of evaluated measurements (*.h5). 
        The default is 'Designation'.
    var_suffix : list of strings, optional
        Suffix of hdf naming rule. The default is [""].
    h5_conc : str, optional
        Identifier of material parameters conclusion in evaluated measurements 
        (*.h5). The default is 'Material_Parameters'.
    h5_data : str, optional
        Identifier of measured and evaluated curves in evaluated measurements 
        (*.h5). The default is 'Measurement'.
    prot_rkws : dict, optional
        Dictionary for reading protocol. Must be keyword in pandas.read_excel.
        The default is dict(header=11, skiprows=range(12,13), index_col=0).
    opt_pd_out : bool, optional
        Option for pandas object output. The default is True.
    opt_hdf_save : bool, optional
        Option for hdf saving. The default is True.

    Returns
    -------
    dfc : pd.DataFrame
        Summary of protocol and material data.
    dfd : pd.DataFrame
        Summary of test data.

    """
    
    dfc=pd.DataFrame([],dtype='float64')
    dfd=pd.Series([],dtype='object')
    for p in in_paths.index:
        prot=pd.read_excel(in_paths.loc[p,'prot'], **prot_rkws)
        for ms in var_suffix:
            for i in prot[hdf_naming].loc[np.invert(prot[hdf_naming].isna())]:
                if os.path.exists(in_paths.loc[p,'hdf']+i+ms+'.h5'):
                    data_read = pd.HDFStore(in_paths.loc[p,'hdf']+i+ms+'.h5','r')
                    df1 = data_read.select(h5_conc)
                    df2 = data_read.select(h5_data)
                    data_read.close()
                    dfp=prot.loc[[prot.loc[prot[hdf_naming]==i].index[0]]]
                    dfp.index=dfp.index+ms
                    df1=pd.concat([dfp,df1],axis=1)
                    dfc = dfc.append(df1)
                    # dfd = dfd.append(df2)
                    dfd[prot.loc[prot[hdf_naming]==i].index[0]+ms] = df2
                    del df1,df2
                else: # added 211028
                    dfp=prot.loc[[prot.loc[prot[hdf_naming]==i].index[0]]]
                    dfp.index=dfp.index+ms
                    dfc = dfc.append(dfp)
    if opt_hdf_save:
        HDFst = pd.HDFStore(out_path+'.h5')
        HDFst['Summary'] = dfc
        HDFst['Test_Data'] = dfd
        HDFst.close()
    if opt_pd_out:
        return dfc, dfd


[docs]def pack_hdf_mul(in_paths, out_path, 
                 hdf_naming = 'Designation', var_suffix = [""],
                 h5_conc = 'Material_Parameters', h5_data = 'all',
                 prot_rkws=dict(header=11, skiprows=range(12,13), index_col=0),
                 opt_pd_out = True, opt_hdf_save = True):
    """
    Packs specimenwise evaluated measurements (HDF-files, *.h5) to a 
    HDF-database containing material parameters key is 'Summary' and other 
    included data (key starting with 'Add_').
    Only all keys in single hdf-files are implemented. (High storage 
    requirements!)

    Parameters
    ----------
    in_paths : pd.DataFrame with columns ['prot','hdf']
        Path for finding protocol and evaluation data.
    out_path : string
        Output path of created database.
    hdf_naming : string, optional
        Naming rule of evaluated measurements (*.h5). 
        The default is 'Designation'.
    var_suffix : list of strings, optional
        Suffix of hdf naming rule. The default is [""].
    h5_conc : str, optional
        Identifier of material parameters conclusion in evaluated measurements 
        (*.h5). The default is 'Material_Parameters'.
    h5_data : str, optional
        Identifier for additional packaging. Only 'all' (packing all keys from 
        input hfd's)implemented. The default is 'all'.
    prot_rkws : dict, optional
        Dictionary for reading protocol. Must be keyword in pandas.read_excel.
        The default is dict(header=11, skiprows=range(12,13), index_col=0).
    opt_pd_out : bool, optional
        Option for pandas object output. The default is True.
    opt_hdf_save : bool, optional
        Option for hdf saving. The default is True.

    Returns
    -------
    dfc : pd.DataFrame
        Summary of protocol and material data.
    dfd : pd.DataFrame
        Summary of test data.

    """
    dfc=pd.DataFrame([],dtype='float64')
    dfd=pd.Series([],dtype='object')
    # dfd={}
    for p in in_paths.index:
        prot=pd.read_excel(in_paths.loc[p,'prot'], **prot_rkws)
        for ms in var_suffix:
            for i in prot[hdf_naming].loc[np.invert(prot[hdf_naming].isna())]:
                if os.path.exists(in_paths.loc[p,'hdf']+i+ms+'.h5'):
                    data_read = pd.HDFStore(in_paths.loc[p,'hdf']+i+ms+'.h5','r')
                    df1 = data_read.select(h5_conc)
                    didks=data_read.keys()
                    for k in didks:
                        df2 = data_read.select(k)
                        if k not in dfd.keys():
                            dfd[k]=pd.Series([],dtype='object')
                        dfd[k][prot.loc[prot[hdf_naming]==i].index[0]+ms]=df2
                        del df2
                    data_read.close()
                    dfp=prot.loc[[prot.loc[prot[hdf_naming]==i].index[0]]]
                    dfp.index=dfp.index+ms
                    df1=pd.concat([dfp,df1],axis=1)
                    dfc = dfc.append(df1)
                    # dfd = dfd.append(df2)
                    # dfd[prot.loc[prot[hdf_naming]==i].index[0]+ms] = df2
                    del df1
                else: # added 211028
                    dfp=prot.loc[[prot.loc[prot[hdf_naming]==i].index[0]]]
                    dfp.index=dfp.index+ms
                    dfc = dfc.append(dfp)
    if opt_hdf_save:
        HDFst = pd.HDFStore(out_path+'.h5','w')
        HDFst['Summary'] = dfc
        # HDFst['Data'] = dfd
        for k in dfd.keys():
            HDFst['Add_'+k] = dfd[k]
        HDFst.close()
    if opt_pd_out:
        return dfc, dfd
```

---

© Copyright 2024, MarcGebhardt.

Built with Sphinx using a
theme
provided by Read the Docs.
