# Supplementary material for: Approach to Standardized Material Characterization of the Human Lumbopelvic System: Testing and Evaluation
Source: Bioengineering (Basel). 2025 Aug 11;12(8):862. doi: 10.3390/bioengineering12080862 (PMC12383908; doi:10.3390/bioengineering12080862)
Supplement: Supplementary file 1 [file bioengineering-12-00862-s001.zip › File S3 Evaluation code/ExMechEva-0.1.2/docs/_build/html/_modules/exmecheva/common/mc_char.html]

exmecheva.common.mc\_char — ExMechEva 0.1.1 documentation


ExMechEva

Contents:

- ExMechEva

ExMechEva

- Module code
- exmecheva.common.mc\_char

---

# Source code for exmecheva.common.mc\_char

```
# -*- coding: utf-8 -*-
"""
Created on Tue Dec  5 12:25:34 2023

@author: mgebhard
"""
import warnings
import numpy as np
import pandas as pd
import scipy
import scipy.signal as scsig

from .analyze import (sign_n_change, sign_n_changeth, normalize, Inter_Lines)
from .pd_ext import (pd_valid_index, pd_combine_index, pd_slice_index,
                     Find_closest, Find_closestv, 
                     Find_closest_perc)
from .mc_man import (Extend_Series_Poly, Extend_Series_n_setter, Retrim_Series,
                     smooth, Smoothsel)
from .fitting import (YM_sigeps_lin)

#%% Standard functions

[docs]def rise_curve(meas_curve, smoothbool, smooth_lvl):
    """ 
    Computes partial integration (1st[rise] and 2nd-grade[curvature])
    to find points of inconstancy in measured curves
    """ 
#    if smoothbool==True:
#        meas_curve=pd.Series(smooth(meas_curve,smooth_lvl),index=meas_curve.index)
    driC=meas_curve.diff()
    if smoothbool==True:
        driC=pd.Series(smooth(driC,smooth_lvl),index=driC.index)
    dcuC=driC.diff()
    driC_sign = np.sign(driC)
    driC_signchange = ((np.roll(driC_sign, True) - driC_sign) != False).astype(bool)
    driC_signchange = pd.Series(driC_signchange,index=driC.index)
    return (driC,dcuC,driC_signchange)


[docs]def find_SandE(Val,Val2,drop_op,drop_val):
    """
    Computes first and last indices which fullfill the choosen condition.

    Parameters
    ----------
    Val : pd.Series([],dtype='float64')
        Input value.
    Val2 : pd.Series([],dtype='float64')
        Testing value.
    drop_op : string, case-sensitive
        Condition name (abV_self,pgm_self,pgm_other,qua_self),
    drop_val : float64
        Value for condition.

    Returns
    -------
    iS : TYPE
        First index which fullfill the choosen condition.
    iE : TYPE
        Last index which fullfill the choosen condition.

    """
    if drop_op=="abV_self":
        iS=Val[abs(Val)>=drop_val].index[0]
        iE=Val[abs(Val)>=drop_val].index[-1]
    elif drop_op=="pgm_self":
        iS=Val[abs(Val)>=(abs(Val).max()*drop_val)].index[0]
        iE=Val[abs(Val)>=(abs(Val).max()*drop_val)].index[-1]
    elif drop_op=="pgm_other":
        iS=Val[abs(Val)>=(abs(Val2)*drop_val)].index[0]
        iE=Val[abs(Val)>=(abs(Val2)*drop_val)].index[-1]
    elif drop_op=="qua_self":
        iS=Val[abs(Val)>=(abs(Val).quantile(drop_val))].index[0]
        iE=Val[abs(Val)>=(abs(Val).quantile(drop_val))].index[-1]
    elif drop_op=="qua_other":
        iS=Val[abs(Val)>=(abs(Val2).quantile(drop_val))].index[0]
        iE=Val[abs(Val)>=(abs(Val2).quantile(drop_val))].index[-1]
    else:
        print("Error in find_SandE (case)!")
    return (iS,iE)


[docs]def Diff_Quot(meas_curve_A, meas_curve_B, 
              smoothbool, smooth_lvl, opt_shift=False):
    """
    Computes difference quotient (1st[rise] and 2nd-grade[curvature])
    to find points of inconstancy in measured curves.

    Parameters
    ----------
    meas_curve_A : pd.Series of float
        Abscissa of the curve for which the difference quotient is to be determined.
    meas_curve_B : pd.Series of float
        Ordinate of the curve for which the difference quotient is to be determined.
    smoothbool : bool
        Controles determination of rolling mean.
    smooth_lvl : positv integer
        Width of rolling mean determination.
    opt_shift : bool
        Shifting returned values to pointed input values.
        If False it points to the index before.
        Leading to a difficult to handle shift, ingreasing with difference quotient grade.
        Optional. The defalut is False.
        
    Test
    -------
    t=pd.DataFrame([[0,0],[1,0],[1.5,1],[2.5,2],[3.5,3],[4,3.5],
                    [5,2.5],[6,2],[7,2]], columns=['x','y'])
    DQ_df = pd.concat(Diff_Quot(t.x, t.y, False, 2, True), axis=1)
        "   DQ1 DQ1_signchange  DQ2 DQ2_signchange  DQ3 DQ3_signchange
         0  NaN           True  NaN           True  NaN           True
         1  0.0           True  NaN           True  NaN           True
         2  2.0          False  4.0           True  NaN           True
         3  1.0          False -1.0           True -5.0           True
         4  1.0          False  0.0          False  1.0           True
         5  1.0           True  0.0           True  0.0           True
         6 -1.0          False -2.0           True -2.0           True
         7 -0.5           True  0.5          False  2.5           True
         8  0.0            NaN  0.5            NaN  0.0            NaN"
        
    Returns
    -------
    DQ1 : pd.Series of float
        Differential quotient 1st grade (rise) of meas_curve_B to meas_curve_A.
    DQ1_signchange : pd.Series of bool
        Indicates sign changes of DQ1.
    DQ2 : pd.Series of float
        Differential quotient 2nd grade (curvature) of meas_curve_B to meas_curve_A.
    DQ2_signchange : pd.Series of bool
        Indicates sign changes of DQ2.
    DQ3 : pd.Series of float
        Differential quotient 3rd grade of meas_curve_B to meas_curve_A.
    DQ3_signchange : pd.Series of bool
        Indicates sign changes of DQ3.

    """
    warnings.warn("Method will be replaced by DiffQuot2", DeprecationWarning)
    if smoothbool==True:
        meas_curve_A = pd.Series(smooth(meas_curve_A,smooth_lvl),
                                 index=meas_curve_A.index)
        meas_curve_B = pd.Series(smooth(meas_curve_B,smooth_lvl),
                                 index=meas_curve_B.index)
    DQ1=meas_curve_B.diff() / meas_curve_A.diff() # m=dSpannung/dDehnung
    # if opt_shift:
    #     DQ1 = DQ1.shift(-1)
    if smoothbool==True:
        DQ1s = pd.Series(smooth(DQ1,smooth_lvl), index=DQ1.index)
    else:
        DQ1s = DQ1
    DQ2 = DQ1s.diff() / meas_curve_A.diff()
    # if opt_shift:
    #     DQ2 = DQ2.shift(-1)
    if smoothbool==True:
        DQ2s = pd.Series(smooth(DQ2,smooth_lvl), index=DQ2.index)
    else:
        DQ2s = DQ2
    DQ3 = DQ2s.diff() / meas_curve_A.diff()
    # if opt_shift:
    #     DQ3 = DQ3.shift(-1)
    
    DQ1_sign, DQ1_signchange, = sign_n_change(DQ1)
    DQ2_sign, DQ2_signchange, = sign_n_change(DQ2)
    DQ3_sign, DQ3_signchange, = sign_n_change(DQ3)
    
    DQ1.name='DQ1'
    DQ2.name='DQ2'
    DQ3.name='DQ3'
    DQ1_signchange.name='DQ1_signchange'
    DQ2_signchange.name='DQ2_signchange'
    DQ3_signchange.name='DQ3_signchange'
    if opt_shift:
        DQ1_signchange = DQ1_signchange.shift(-1, fill_value=False)
        DQ2_signchange = DQ2_signchange.shift(-1, fill_value=False)
        DQ3_signchange = DQ3_signchange.shift(-1, fill_value=False)
    return DQ1,DQ1_signchange,DQ2,DQ2_signchange,DQ3,DQ3_signchange


[docs]def test_pdmon(df,cols,m,dist):
    """
    Test of monotonic de-/increasing in dataframe columns.

    Parameters
    ----------
    df : pd.DataFrame
        Dataframe which includes cols as Column-names.
    cols : string or array of strings
        Column-names for monotonic test.
    m : np.int
        Kind of monotoni (1=increasing, -1=decreasing).
    dist : np.int
        test distance of monotonic creasing.

    Returns
    -------
    d : pd.Series([],dtype='float64')
        Series with index 'cols' of last 'df.index' with monotonic de-/increasing of 'dist'-length.
    """
    
    b=df.loc[:,cols].transform(lambda x: np.sign(x.diff()))
    d=pd.Series([],dtype='float64')
    for i in b.iloc[dist+1:].index:
        c=(m==b.loc[i-dist+1:i]).all(axis=0)
        for j in c.index:
            if c[j]:  d[j]=i
    return (d)


[docs]def YM_eva_range_refine(m_df, VIP, n_strain, n_stress,
                         n_loBo='S', n_upBo='U',
                         d_loBo=0.05, d_max=0.75, 
                         rise_det=[True,4],
                         n_Outlo='F3',n_Outmi='FM',n_Outhi='F4'):
    """
    Refines the Youngs Modulus determinition range according to 
    "Keuerleber, M. (2006) - Bestimmung des Elastizitätsmoduls von Kunststoffen
    bei hohen Dehnraten am Beispiel von PP. Von der Fakultät Maschinenbau der 
    Universität Stuttgart zur Erlangung der Würde eines Doktor-Ingenieurs (Dr.-Ing.) 
    genehmigte Abhandlung. Doktorarbeit. Universität Stuttgart, Stuttgart"

    Parameters
    ----------
    m_df : pd.DataFrame
        Measured data.
    VIP : pd.Series
        Important points corresponding to measured data.
    n_strain : string
        Name of used strain (have to be in measured data).
    n_stress : string
        Name of used stress (have to be in measured data).
    n_loBo : string
        Lower border for determination (have to be in VIP). The default is 'S'.
    n_upBo : string
        Upper border for determination (have to be in VIP). The default is 'U'.
    d_loBo : float/str, optional
        When float: Percentage of range between n_upBo and n_loBo as start distance to n_loBo.
        When str starting with 'S', followed by integer: Distance in steps to n_loBo.
        The default is 0.05.
    d_max : float, optional
        Percentage of . The default is 0.75.
    rise_det : [bool, int], optional
        Determination options for stress rising ([smoothing, smoothing factor]).
    n_Outlo : string, optional
        Name of new lower border. The default is 'F3'.
    n_Outmi : string, optional
        Name of maximum differential quotient. The default is 'FM'.
    n_Outhi : string, optional
        Name of new upper border. The default is 'F4'.

    Yields
    ------
    VIP_new : pd.Series
        Important points corresponding to measured data.
    txt : string
        Documantation string.
    """
    if isinstance(d_loBo, str):
        if d_loBo.startswith('S'):
            i_loBo = int(d_loBo[1:])
        else:
            raise ValueError("Distance to lower border %s seems to be a string, but doesn't starts with 'S'"%d_loBo)
        Lbord = VIP[n_loBo]+i_loBo
        ttmp = m_df.loc[VIP[n_loBo]+i_loBo:VIP[n_upBo],n_stress]
    else:
        ttmp = m_df.loc[VIP[n_loBo]:VIP[n_upBo],n_stress]
        ftmp=float(ttmp.iloc[0]+(ttmp.iloc[-1]-ttmp.iloc[0])*d_loBo)
        Lbord=abs(ttmp-ftmp).idxmin()

    DQdf=pd.concat(Diff_Quot(m_df.loc[:,n_strain], m_df.loc[:,n_stress],
                              rise_det[0], rise_det[1]), axis=1)
    DQdf=m_df.loc(axis=1)[[n_strain,n_stress]].join(DQdf,how='outer')
    DQdfs=DQdf.loc[Lbord:VIP[n_upBo]]
    
    
    VIP_new = VIP
    txt =""
    VIP_new[n_Outmi]=DQdfs.DQ1.idxmax()
    try:
        VIP_new[n_Outlo]=DQdfs.loc[:VIP_new[n_Outmi]].iloc[::-1].loc[(DQdfs.DQ1/DQdfs.DQ1.max())<d_max].index[0]+1
    except IndexError:
        VIP_new[n_Outlo]=DQdfs.index[0]
        txt+="%s set to start of diff.-quot. determination "%n_Outlo
    try:
        VIP_new[n_Outhi]=DQdfs.loc[VIP_new[n_Outmi]:].loc[(DQdfs.DQ1/DQdfs.DQ1.max())<d_max].index[0]-1
    except IndexError:
        # VIP_new[n_Outhi]=VIP_new[n_Outmi]-1 #-1 könnte zu Problemen führen
        VIP_new[n_Outhi]=VIP_new[n_upBo] #-1 könnte zu Problemen führen
        txt+="%s set on maximum of diff.-quot. "%n_Outhi
    VIP_new=VIP_new.sort_values()
    return VIP_new, DQdfs, txt


[docs]def Diff_Quot2(x, y, 
               smooth_bool=False, smooth_type='SMA', 
               smooth_opts={'window_length':3}, 
               smooth_snip=False, 
               # sc_kwargs={'norm':None, 'normadd':0.5,
               #            'th':None, 'th_option':'abs', 'th_set_val':0},
               sc_kwargs={'norm':'absmax', 'normadd':0.5,
                          'th':0.05, 'th_option':'abs', 'th_set_val':0},
               opt_shift=False, opt_out='Tuple'):
    """
    Computes difference quotient of input arrays (1st[rise], 2nd[curvature] and 3rd-grade)
    to find points of inconstancy in measured curves.

    Parameters
    ----------
    x : pd.Series of float
        Abscissa of the curve for which the difference quotient is to be determined.
    y : pd.Series of float
        Ordinate of the curve for which the difference quotient is to be determined.
    smoothbool : bool or string
        Controles determination of rolling mean.
    smooth_type : string, case-sensitive, optional
        Choosen smoothing type.
        Optional. The defalut is 'SMA'.
        Possible are:
            - 'SMA': Moving average based on numpy.convolve.
            - 'SMA_f1d': Moving average based scipy.ndimage.filters.uniform_filter1d.
            - 'SavGol': Savitzky-Golay filter based on scipy.signal.savgol_filter.
    smooth_opts : dict, optional
        Keywords and values to pass to smoothing method. 
        For further informations see smooth_type and linked methods.
        Optional. The defalut is {'window_length':3, 'mode':'same'}.
    smooth_snip : bool or integer, optional
        Trimming of output. Either, if True with window_length in smooth_opts,
        none if False, or with inserted distance. 
        The default is False.
    opt_shift : bool
        Shifting returned values to pointed input values.
        If False it points to the index before.
        Leading to a difficult to handle shift.
        Optional. The defalut is False.
    opt_out : string
        Type of Output. Either Tuple or pandas dataframe.
        Optional. The defalut is 'Tuple'.
        
    Test
    -------
    t=pd.DataFrame([[0,0],[1,0],[1.5,1],[2.5,2],[3.5,3],[4,3.5],
                    [5,2.5],[6,2],[7,2]], columns=['x','y'])
    DQ_df = pd.concat(Diff_Quot(t.x, t.y, False, 2, True), axis=1)
        "   DQ1 DQ1_signchange  DQ2 DQ2_signchange  DQ3 DQ3_signchange
         0  NaN           True  NaN           True  NaN           True
         1  0.0           True  NaN           True  NaN           True
         2  2.0          False  4.0           True  NaN           True
         3  1.0          False -1.0           True -5.0           True
         4  1.0          False  0.0          False  1.0           True
         5  1.0           True  0.0           True  0.0           True
         6 -1.0          False -2.0           True -2.0           True
         7 -0.5           True  0.5          False  2.5           True
         8  0.0          False  0.5          False  0.0          False"
        
    Returns
    -------
    DQ1 : pd.Series of float
        Differential quotient 1st grade (rise) of y to x.
    DQ1_si : pd.Series of bool
       Signs of DQ1.
    DQ1_sc : pd.Series of bool
        Indicates sign changes of DQ1.
    DQ2 : pd.Series of float
        Differential quotient 2nd grade (curvature) of y to x.
    DQ2_si : pd.Series of bool
       Signs of DQ2.
    DQ2_sc : pd.Series of bool
        Indicates sign changes of DQ2.
    DQ3 : pd.Series of float
        Differential quotient 3rd grade (curvature change) of y to x.
    DQ3_si : pd.Series of bool
       Signs of DQ3.
    DQ3_sc : pd.Series of bool
        Indicates sign changes of DQ3.

    """
    
    if smooth_bool in [True,'Input','x-only','y-only']:
        if smooth_bool in [True,'Input','x-only']:
            x = Smoothsel(x=x, smooth_type=smooth_type,
                          smooth_opts=smooth_opts, snip=smooth_snip)
        if smooth_bool in [True,'Input','y-only']:
            y = Smoothsel(x=y, smooth_type=smooth_type,
                          smooth_opts=smooth_opts, snip=smooth_snip)
    DQ1=y.diff() / x.diff() # m=dOrdinate/dAbscissa
    # if opt_shift:
    #     DQ1 = DQ1.shift(-1)
    if smooth_bool==True:
        DQ1s = Smoothsel(x=DQ1, smooth_type=smooth_type,
                         smooth_opts=smooth_opts, snip=smooth_snip)
    else:
        DQ1s = DQ1
    DQ2 = DQ1s.diff() / x.diff()
    # if opt_shift:
    #     DQ2 = DQ2.shift(-1)
    if smooth_bool==True:
        DQ2s = Smoothsel(x=DQ2, smooth_type=smooth_type,
                         smooth_opts=smooth_opts, snip=smooth_snip)
    else:
        DQ2s = DQ2
    DQ3 = DQ2s.diff() / x.diff()
    # if opt_shift:
    #     DQ3 = DQ3.shift(-1)
    
    # DQ1_sign, DQ1_signchange, = sign_n_change(DQ1)
    # DQ2_sign, DQ2_signchange, = sign_n_change(DQ2)
    # DQ3_sign, DQ3_signchange, = sign_n_change(DQ3)
    
    # DQ1_signchange = DQ1_signchange.astype(bool)
    # DQ2_signchange = DQ2_signchange.astype(bool)
    # DQ3_signchange = DQ3_signchange.astype(bool)
    
    DQ1.name='DQ1'
    DQ2.name='DQ2'
    DQ3.name='DQ3'
    # DQ1_signchange.name='DQ1_signchange'
    # DQ2_signchange.name='DQ2_signchange'
    # DQ3_signchange.name='DQ3_signchange'
    
    DQ1_si, DQ1_sc, = sign_n_changeth(DQ1,**sc_kwargs)
    DQ2_si, DQ2_sc, = sign_n_changeth(DQ2,**sc_kwargs)
    DQ3_si, DQ3_sc, = sign_n_changeth(DQ3,**sc_kwargs)
    
    if opt_shift:
        DQ1 = DQ1.shift(-1, fill_value=np.nan)
        DQ2 = DQ2.shift(-1, fill_value=np.nan)
        DQ3 = DQ3.shift(-1, fill_value=np.nan)
        DQ1_si = DQ1_si.shift(-1, fill_value=np.nan)
        DQ2_si = DQ2_si.shift(-1, fill_value=np.nan)
        DQ3_si = DQ3_si.shift(-1, fill_value=np.nan)
        DQ1_sc = DQ1_sc.shift(-1, fill_value=False)
        DQ2_sc = DQ2_sc.shift(-1, fill_value=False)
        DQ3_sc = DQ3_sc.shift(-1, fill_value=False)
    if opt_out in ['DF','DataFrame','Dataframe']:
        out = pd.concat([DQ1,DQ1_si,DQ1_sc,
                         DQ2,DQ2_si,DQ2_sc,
                         DQ3,DQ3_si,DQ3_sc], axis=1)
        # temporäre Lösung
        out[['DQ1_sc','DQ2_sc','DQ3_sc']]=out[['DQ1_sc','DQ2_sc','DQ3_sc']].fillna(False)
        return out
    elif opt_out=='Tuple':
        return DQ1, DQ1_si, DQ1_sc, DQ2, DQ2_si, DQ2_sc, DQ3, DQ3_si, DQ3_sc
    else:
        raise NotImplementedError("Output option %s not implemented!"%opt_out)
        return DQ1, DQ1_si, DQ1_sc, DQ2, DQ2_si, DQ2_sc, DQ3, DQ3_si, DQ3_sc


[docs]def Diff_Quot3(y, x=None, deep=3, 
               ex_bool = True, ex_kwargs={'polydeg':1},
               smooth_bool=False, smooth_type='SMA', 
               smooth_opts={'window_length':3}, 
               sc_kwargs={'norm':'absmax', 'normadd':0.5,
                          'th':0.05, 'th_option':'abs', 'th_set_val':0},
               shift_value=False, opt_out='DataFrame'):
    """
    Computes difference quotient of input arrays (nth-grade) and their signs, 
    as well as sign changes to find points of inconstancy in measured curves.

    Parameters
    ----------
    y : pd.Series of float
        Ordinate of the curve for which the difference quotient is to be 
        determined.
    x : pd.Series of float or None, optional
        Abscissa of the curve for which the difference quotient is to be 
        determined. The default is None.
    deep : int, optional
        Number of differential quotients to determine. The default is 3.
    ex_bool : bool, optional
        Switch for extending data with Extend_Series_n_setter and afterwards 
        retrim them with Retrim_Series. The default is True.
    ex_kwargs : dict, optional
        Keyword arguments for Extend_Series_n_setter. 
        The default is {'polydeg':1}.
    smooth_bool : bool or string, optional
        Switch for smoothing of data. Must ether be True or False or in 
        ['Input','x-only','y-only','yandDQ','DQ']. The default is False.
    smooth_type : string, optional
        Type of smoothing methode (see Smoothsel for further information).
        The default is 'SMA'.
    smooth_opts : dict, optional
        Smoothing option for choosen method (see Smoothsel for further 
        information). The default is {'window_length':3}.
    sc_kwargs : dict, optional
        Keyword arguments for sign and sign change determination 
        (normalization and thresholding, for further information see 
        ./analyze.sign_n_changeth).
        The default is {'norm':'absmax', 'normadd':0.5, 
                        'th':0.05, 'th_option':'abs', 'th_set_val':0}.
    shift_value : bool or int, optional
        Shifting of values. The default is False.
    opt_out : string, optional
        Output option (only DataFrame or DF implemented yet). 
        The default is 'DataFrame'.

    Raises
    ------
    TypeError
        Determination length of smooth_opts seems to be wrong.
    NotImplementedError
        Output option not implemented.

    Returns
    -------
    out : pd.DataFrame
        Differnece quotients, signs and sign changes of given input data.

    """
    if x is None:
        x=y.index
    if ex_bool:
        if 'n' not in ex_kwargs.keys():
            ntmp1 = ntmp2 = 0
            if smooth_bool in [True,'Input','x-only','y-only','yandDQ','DQ']:
                if isinstance(smooth_opts, dict):
                    ntmp1 = Extend_Series_n_setter('Smooth',
                                                   ffunckwargs=smooth_opts)
                elif isinstance(smooth_opts, list):
                    ntmp1 = Extend_Series_n_setter('Smooth',
                                                   ffuncargs=smooth_opts)
                elif isinstance(smooth_opts, int or float):
                    ntmp1 = Extend_Series_n_setter('Smooth',
                                                   ffuncargs=[smooth_opts])
                else:
                    raise TypeError("Type %s of smoothopts not implemented! (Determination extension length)")
            if shift_value or (shift_value !=0):
                if shift_value: shift_value = -1 
                ntmp2 = Extend_Series_n_setter('Shift',
                                               ffuncargs=shift_value)
            ntmp3 = Extend_Series_n_setter('Diff',ffuncargs=deep)
            ex_kwargs['n'] = max(ntmp1,ntmp2,ntmp3)
        if 'kind' not in ex_kwargs.keys(): 
            ex_kwargs['kind'] = 'fb'
        x = Extend_Series_Poly(x, **ex_kwargs)
        y = Extend_Series_Poly(y, **ex_kwargs)
    
    if smooth_bool in [True,'Input','x-only','y-only','yandDQ']:
        if smooth_bool in [True,'Input','x-only']:
            x = Smoothsel(x=x, smooth_type=smooth_type,
                          smooth_opts=smooth_opts, snip=False)
        if smooth_bool in [True,'Input','y-only','yandDQ']:
            y = Smoothsel(x=y, smooth_type=smooth_type,
                          smooth_opts=smooth_opts, snip=False)
    df=pd.concat([x,y],axis=1)
    df.columns=['x','y']
    df['dx'] = x.diff()
    df['dy'] = y.diff()
    DQs_tmp = df['y']
    for d in range(1,deep+1):
        DQ_name = 'DQ{}'.format(d) 
        df[DQ_name] = DQs_tmp.diff() / df['dx'] # m=dOrdinate/dAbscissa
        if smooth_bool in [True,'yandDQ','DQ']:
            DQs_tmp = Smoothsel(x=df[DQ_name], smooth_type=smooth_type,
                                smooth_opts=smooth_opts, snip=False)
        else:
            DQs_tmp = df[DQ_name]
    t = sign_n_changeth(df,**sc_kwargs,opt_out = 'DF')
    df = pd.concat([df,t],axis=1)
    if shift_value: shift_value = -1
    if shift_value != 0: 
        # df = df.shift(periods=shift_value, fill_value=None)
        # exclude original values (x and y)
        dfs=df.iloc(axis=1)[2:].shift(-1, fill_value=None)
        df[dfs.columns] = dfs
    if ex_bool:
        df = Retrim_Series(y=df, axis=0, n=ex_kwargs['n'], 
                           kind=ex_kwargs['kind'])
    if opt_out in ['DF','DataFrame','Dataframe']:
        out = df
        return out
    else:
        raise NotImplementedError("Output option %s not implemented!"%opt_out)

#%% Automatic curve characterization and merging

[docs]def peaky_finder(df, cols='all', norm='absmax',
                 fp_kwargs={'prominence':0.1, 'height':0.1},
                 out_opt='valser-loc'):
    """
    Find peaks in passed input data.

    Parameters
    ----------
    df : pandas DataFrame
        Input data.
    cols : string or array of strings or boolean, optional
        Columns (axis=1) of data to use. The default is 'all'.
    norm : string, optional
        Normalization method. The default is 'absmax'.
    fp_kwargs : dict, optional
        Keyword arguments to pass to scipy.signal.find_peaks.
        The default is {'prominence':0.1, 'height':0.1}.
    out_opt : string, optional
        Output options. The default is 'valser-loc'.

    Raises
    ------
    NotImplementedError
        Option not implemented.

    Returns
    -------
    o : Series of arrays
        Series with input-columns as index and array of input index as values.

    """
    if cols=='all':
        df2 = df
    else:
        # df2 = df[cols]
        df2 = df.loc(axis=1)[cols]
        
    # if norm == 'absmax':
    #     df2 = df2/abs(df2).max(axis=0)
    # elif norm == 'max':
    #     df2 = df2/df2.max(axis=0)
    # elif norm == 'min':
    #     df2 = df2/df2.min(axis=0)
    # elif norm is None:
    #     df2 = df2
    # else:
    #     raise NotImplementedError("Norming %s not implemented!"%norm)
    df2 = normalize(pdo=df2, axis=0, norm=norm)
    
    # o=df2.agg(scipy.signal.find_peaks,**fp_kwargs)
    o=df2.agg(scsig.find_peaks,**fp_kwargs)
    if out_opt == 'complete-iloc':
        o = o
    elif out_opt == 'complete-loc':
        o.iloc[0] = o.iloc[0].apply(lambda x: df2.index[x].values)
    elif out_opt == 'valser-iloc':
        o = o.iloc[0]
    elif out_opt == 'valser-loc':
        o = o.iloc[0].apply(lambda x: df2.index[x].values)
    else:
        raise NotImplementedError("Output option %s not implemented!"%out_opt)
    
    return o


[docs]def peaky_finder_MM(df, cols='all', norm='absmax',
                    fp_kwargs={'prominence':0.1, 'height':0.1},
                    out_opt='valser-loc'):
    """Pacckage method of peaky_finder to find maxima and minima."""
    omax=peaky_finder(df= df, cols=cols, norm=norm,
                      fp_kwargs=fp_kwargs, out_opt=out_opt)
    omin=peaky_finder(df=-df, cols=cols, norm=norm,
                      fp_kwargs=fp_kwargs, out_opt=out_opt)
    return omax, omin


[docs]def curvecar_section(i_start, i_end, ref_ser, 
                     sm_w_length=0, threshold=0.01, norm='abs_max_ref',
                     out_vals=['','Const','Rise','Fall'], kind="median"):
    """
    Determine type of section by comparing a reference series to a given threshold.

    Parameters
    ----------
    i_start : corresponding type of index
        Start index of section.
    i_end : corresponding type of index
        End index of section.
    ref_ser : pands Series
        Reference series.
    sm_w_length : corresponding type of index, optional
        Determination distance to start and end. The default is 0.
    threshold : float, optional
        Determination threshold. The default is 0.01.
    norm : string, optional
        Nomralization method. The default is 'abs_max_ref'.
    out_vals : array of strings, optional
        Strings for determination type. The default is ['','Const','Rise','Fall'].

    Returns
    -------
    string
        Determined type of curve section.
    float
        Mean value over range of reference series.

    """
    i_sb = pd_valid_index(i_start+sm_w_length, ref_ser, opt='n')
    i_eb = pd_valid_index(i_end-sm_w_length, ref_ser, opt='n')
    if kind== "mean":
        mob_mean = ref_ser.loc[i_sb:i_eb].mean()
    elif kind== "median":
        mob_mean = ref_ser.loc[i_sb:i_eb].median()
    else:
        return NotImplementedError("Kind %s not implemented!"%kind)
    
    if norm == 'abs_max_ref':
        mob_mean_norm = mob_mean/abs(ref_ser).max()
    if norm == 'abs_med_ref':
        mob_mean_norm = mob_mean/abs(ref_ser).median()
    elif norm is None:
        mob_mean_norm = mob_mean 
    else:
        return NotImplementedError("Norm %s not implemented!"%norm)
    
    if  abs(mob_mean_norm) <= threshold:
        o = out_vals[1]
    elif mob_mean_norm > threshold:
        o = out_vals[2]
    elif mob_mean_norm < threshold:
        o = out_vals[3]
    else:
        o = out_vals[0]
    return o, mob_mean


[docs]def curvecar_refine(i_start,i_mid,i_end, ref_ser,
                    threshold=0.5, norm='abs_mid',
                    out_vals=['','Pos','Neg']):
    """
    Determine type, as well as new start and end points,
    of a refined section by comparing a reference series to a given threshold.

    Parameters
    ----------
    i_start : corresponding type of index
        Start index of first section.
    i_mid : corresponding type of index
        Index between sections.
    i_end : corresponding type of index
        End index of second section.
    ref_ser : pands Series
        Reference series.
    threshold : float, optional
        Determination threshold. The default is 0.5.
    norm : string, optional
        Nomralization method. The default is 'abs_mid'.
    out_vals : array of strings, optional
        Strings for determination type. The default is ['','Pos','Neg'].

    Returns
    -------
    string
        Determined type of curve section.
    corresponding type of index
        New start point of refined curve section.
    corresponding type of index
        New end point of refined curve section.
    float
        Mean value over range of reference series.

    """
    if norm == 'abs_mid':
        tn=abs(ref_ser/ref_ser.loc[i_mid])
    elif norm == 'abs_absmax_range':
        tn=abs(ref_ser/abs(ref_ser.loc[i_start:i_end]).max())
    elif norm == 'abs_absmax_complete':
        tn=abs(ref_ser/abs(ref_ser).max())
    elif norm == 'abs_absmed_complete':
        tn=abs(ref_ser/abs(ref_ser).median())
    elif norm == 'abs':
        tn=abs(ref_ser)
    elif norm is None:
        tn=ref_ser
    else:
        return NotImplementedError("Norm %s not implemented!"%norm)
        
    tnb=tn.loc[i_start:i_mid].iloc[::-1]
    tna=tn.loc[i_mid:i_end]
    try:
        i_snew=tnb.loc[tnb<=threshold].index[0]
    except IndexError:
        i_snew=None
    try:
        i_enew=tna.loc[tna<=threshold].index[0]
    except IndexError:
        i_enew=None
    if i_snew is None and i_enew is None:
        o = out_vals[0]
        return o, i_snew, i_enew, np.nan
    elif i_snew is None:
        i_snew=i_mid
    elif i_snew is None:
        i_enew=i_mid
    mean = ref_ser.loc[i_snew:i_enew].mean()
    if mean > 0:
        o = out_vals[1]
    elif mean < 0:
        o = out_vals[2]
    else:
        raise ValueError('mean is zero!')
    return o, i_snew, i_enew, mean


[docs]def curve_characterizer(x, y,
                        ex_bool=True, ex_kwargs={'polydeg':1},
                        smooth_bool=True, smooth_type='SMA',
                        smooth_opts={'window_length':3},
                        # smooth_snip=True,  opt_shift=True,
                        shift_value=-1,
                        sc_kwargs={'norm':'absmax', 'normadd':0.5,
                                   'th':0.05, 'th_option':'abs', 'th_set_val':0},
                        peak_norm='absmax', peak_kwargs={'prominence':0.1, 'height':0.1}, 
                        cc_snip=3, cc_threshold=0.01, cc_norm='abs_max_ref',
                        cc_refine=True, ccr_threshold=0.75, ccr_norm='abs_mid',
                        nan_policy='omit'):
    """
    Characterize a measured curve. To plot results please see 
    curve_char_plotter in ./plotting.py.

    Parameters
    ----------
    x : pandas Series of float, or pandas.DataFrame
        Abscissa or difference quotient dataframe of input.
    y : pandas Series of float
        Ordinate of input.
		
    smooth_bool : bool or string, optional
        Smoothing behavior. The default is True.
    smooth_type : string, case-sensitive, optional
        Choosen smoothing type.
        Optional. The defalut is 'SMA'.
        Possible are:
            - 'SMA': Moving average based on numpy.convolve.
            - 'SMA_f1d': Moving average based scipy.ndimage.filters.uniform_filter1d.
            - 'SavGol': Savitzky-Golay filter based on scipy.signal.savgol_filter.
    smooth_opts : dict, optional
        Keywords and values to pass to smoothing method. 
        For further informations see smooth_type and linked methods.
        Optional. The defalut is {'window_length':3, 'mode':'same'}.
    smooth_snip : bool or integer, optional
        Trimming of output. Either, if True with window_length in smooth_opts,
        none if False, or with inserted distance. 
        The default is False.
    opt_shift : bool
        Shifting returned values to pointed input values.
        If False it points to the index before.
        Leading to a difficult to handle shift.
        Optional. The defalut is False.
		
    peak_norm : string, optional
        Normalization method. The default is 'absmax'.
    peak_kwargs : dict, optional
        Keyword arguments to pass to scipy.signal.find_peaks.
        The default is {'prominence':0.1, 'height':0.1}.
		
    cc_snip : corresponding type of index, optional
        Determination distance to start and end for characterization of 
        section. The default is 3.
    cc_threshold : float, optional
        Determination threshold for characterization of section.
        The default is 0.01.
    cc_norm : string, optional
        Nomralization method for characterization of section.
        The default is 'abs_max_ref'.
		
    cc_refine : bool, optional
        Turn refinement by curvature of curve section on. The default is True.
    ccr_threshold : float, optional
        Determination threshold of refinement. The default is 0.75.
    ccr_norm : string, optional
        Nomralization method of refinement. The default is 'abs_mid'.

    Returns
    -------
    pandas Dataframe
        Characterization of input (for linear parts: linearisation and
                                   intersection to previous included).
    pandas Series
        Points of first characteriation(S=Start,E=End, I=Increase, D=Decrease).
    pandas Dataframe
        Maxima and minima of input, as well as differential quotients.
    pandas Dataframe
		Input, as well as differential quotients.
    """
    if nan_policy in ['i', 'interpolate']:
        x = x.interpolate(limit_direction='both') if x.isna().any() else x
        y = y.interpolate(limit_direction='both') if y.isna().any() else y
        
    if isinstance(x, pd.core.base.ABCDataFrame):
        df = x.copy(deep=True)
    else:
        df = Diff_Quot3(y=y, x=x, deep=3, ex_bool=ex_bool, ex_kwargs=ex_kwargs,
                        smooth_bool=smooth_bool, smooth_type=smooth_type,
                        smooth_opts=smooth_opts,
                        sc_kwargs=sc_kwargs,
                        shift_value=shift_value, opt_out='DataFrame')
    
    # Find peaks
    a,b=peaky_finder_MM(df=df, cols='all', norm=peak_norm,
    # # exclude sign and n
    # a,b=peaky_finder_MM(df=df, cols=~df.columns.str.contains('_s.*',regex=True),
                        fp_kwargs=peak_kwargs, out_opt='valser-loc')
    def max_or_min(col,ind,max_p_ser,min_p_ser):
        if ind in max_p_ser[col]:
            o='Max'
        elif ind in min_p_ser[col]:
            o='Min'
        else:
            o=''
        return o
    dfp = df.apply(lambda x: pd.DataFrame(x).apply(
        lambda y: max_or_min(x.name, y.name, a, b), axis=1)
        )
    dfp = dfp.loc[np.invert((dfp=='').all(axis=1))]
    
    #pre-identify linear sections
    cps = pd.DataFrame([], columns=['Type','Start','End','Special'], dtype='O')
    cip = pd.Series([], dtype=str)
    j = 0
    ib=df.index[0]
    cip[ib]='S'
    ind_DQ2MM = dfp.loc[(dfp.DQ2 == 'Max') | (dfp.DQ2 == 'Min')].index
    for i in ind_DQ2MM:
        cps.loc[j,'Start']=ib
        cps.loc[j,'End']=i
        if dfp.loc[i,'DQ2'] == 'Max':
            cip[i]='I'
        else:
            cip[i]='D'
        t1,t2=curvecar_section(i_start=ib, i_end=i, ref_ser=df.DQ1,
                               sm_w_length=cc_snip,
                               threshold=cc_threshold, norm=cc_norm)
        cps.loc[j,'Type']= t1
        cps.loc[j,'Special']= t2
        ib = i
        j += 1
        if i == ind_DQ2MM[-1]:
            ia=df.index[-1]
            cps.loc[j,'Start']=i
            cps.loc[j,'End']=ia
            t1,t2=curvecar_section(i_start=i, i_end=ia, ref_ser=df.DQ1,
                                   sm_w_length=cc_snip,
                                   threshold=cc_threshold, norm=cc_norm)
            cps.loc[j,'Type']= t1
            cps.loc[j,'Special']= t2
            cip[ia]='E'
            
    if cc_refine:
        cps_new = cps.copy(deep=True)
        cps_i_crf=cps.loc[cps.Type.apply(lambda x: x in ['Const','Rise','Fall'])].index
        for i in cps_i_crf[:-1]:
            t=curvecar_refine(cps_new.loc[i,'Start'],cps_new.loc[i,'End'],
                              cps_new.loc[i+1,'End'],
                              df['DQ2'], threshold=ccr_threshold,
                              norm=ccr_norm, out_vals=['','Pos','Neg'])
            if t[0] != '':
                cps_new.loc[i,'End']=t[1]
                cps_new.loc[i+1,'Start']=t[2]
                cps_new.loc[i+0.1,['Type','Start','End','Special']]=t
        cps=cps_new.sort_index()
        t=cps.loc[cps_i_crf].apply(lambda i: curvecar_section(i_start=i.Start,
                                                              i_end=i.End,
                                                              ref_ser=df.DQ1,
                                                              sm_w_length=cc_snip,
                                                              threshold=cc_threshold,
                                                              norm=cc_norm),
                                   axis=1, result_type='expand')
        t.columns=['Type','Special']
        cps.loc[t.index,t.columns]=t
        
    # leat-squares-fit on linear sections
    cps_i_crf=cps.loc[cps.Type.apply(lambda x: x in ['Const','Rise','Fall'])].index
    t = cps.loc[cps_i_crf].apply(lambda i: YM_sigeps_lin(df.y, df.x,
                                                         ind_S=i.Start,
                                                         ind_E=i.End,
                                                         nan_policy='propagate'),
                                     axis=1, result_type='expand')
    t.columns=['r','c','Rq','lmfR']
    cps[['r','c','Rq']]=t[['r','c','Rq']]
    
    #Find points of intersection for linear sections
    # for i in cps.index:
    for i in cps_i_crf:
        if i==cps.index[0]:
            # cps.loc[i,'IntP_x'] = inter_lines(np.inf, df.loc[0,'x'],
            #                                   cps.loc[i  ,'r'],cps.loc[i  ,'c'], out='x')
            cps.loc[i,'IntP_x'] = df.iloc[0]['x']
        else:
            cps.loc[i,'IntP_x']=Inter_Lines(cps.loc[i-1,'r'],cps.loc[i-1,'c'],
                                            cps.loc[i  ,'r'],cps.loc[i  ,'c'], out='x')
        if i==cps.index[-1]:
            cps.loc[i+1,'Type'] = 'Last'
            cps.loc[i+1,'Start'] = cps.loc[i+1,'End']  = cps.loc[i,'End']
            cps.loc[i+1,['r','c']] = cps.loc[i,['r','c']]
            cps.loc[i+1,'IntP_x'] = df.iloc[-1]['x']
    cps['IntP_y'] = cps['IntP_x']*cps['r']+cps['c']
    
    return cps, cip, dfp, df


[docs]def curve_merger(cps1,cps2, how='1stMax', option='nearest'):
    """
    Finds time offset between two measured curves.

    Parameters
    ----------
    cps1 : pandas Dataframe (result of curve_characterizer)
        First curve characterization.
    cps2 : pandas Dataframe (result of curve_characterizer)
        Second curve characterization.
    how : string, optional
        Switch for how to determine time offset. Possible are:
            - '1stMax': first maximum
            - 'wo_1st+last': without first and last relevant positions
            - 'complete': all relevant positions
            - 'list;[x,x,...]': list of relevant positions
            - 'int;x': one relevant position
            - 'fix;x': fixed time offset
        The default is '1stMax'.
    option : string, optional
        Switch for offset determination. The default is 'nearest'
        (merging nearest positions found in both frames).

    Raises
    ------
    NotImplementedError
        Method not implemented.

    Returns
    -------
    t_off : float
        Offset to apply on first to merge second.
    t1 : float or pd.Series
        Timestamp/-s of first curve characterization.
    t2 : float or pd.Series
        Timestamp/-s of second curve characterization.

    """
    if how == '1stMax':
        t1=cps1.loc[cps1.Type=='Fall','IntP_x'].iloc[0]
        t2=cps2.loc[cps2.Type=='Fall','IntP_x'].iloc[0]
    elif how == 'wo_1st+last':
        t1=cps1.IntP_x.iloc[1:-1]
        t2=cps2.IntP_x.iloc[1:-1]
    elif how == 'complete':
        t1=cps1.IntP_x
        t2=cps2.IntP_x
    else:
        howt=how.split(';')
        if howt[0] == 'list':
            j=0
            how1=howt[1].split(',')
            for how2 in how1:
                how1[j] = int(how2)
                j+=1
            t1=cps1.IntP_x.iloc[how1]
            t2=cps2.IntP_x.iloc[how1]
        elif howt[0] == 'int':
            how1=int(howt[1])
            t1=cps1.IntP_x.iloc[how1]
            t2=cps2.IntP_x.iloc[how1]
        elif howt[0] == 'fix':
            t1=float(howt[1])
            t2=0.0
        else:
            raise NotImplementedError("Method %s not implemented!"%how)

    if isinstance(t1, float) and isinstance(t2, float):
        t_off=t1-t2
    else:
        if option.split('_')[0]=='nearest':
            t=t2.apply(lambda x: Find_closest(t1,x))
            tt=t.duplicated(False)
            if tt.any():
                for i in tt[tt].index:
                    ti=Find_closest(t2,t1[t[i]])
                    if i!= ti and t[i] == t[ti]:
                        t.drop(i,inplace=True)
            t1=t1[t.values]
            t2=t2[t.index]
            t_off=np.mean(t1.values-t2.values)
        else:
            t_off=(t1-t2).mean()
    return t_off, t1, t2

#%% Points of interest

[docs]def poi_rel_finder(cip, cycles, xser, df, dx=None,
                   POI_start='P', POI_sec=['l','u'],
                   comp_dfx='x', rel_df='DQ2', ascending=True):
    """
    Finds the relevant point from several given points. For example, if there 
    are several rise changes (e.g. due to measurement deviation), an attempt 
    is made to find the actual one. 

    Parameters
    ----------
    cip : pd.Series ((interim-)result of poi_det_plh)
        Points of interest, with index by names and value by index of input.
        data. Labels are 'H'-high point, 'L'-low point, 'Pl'-rise change on 
        loading and 'Pu'-rise change on unloading.
    cycles : int
        Number of cyclic loadings.
    xser : pd.Series
        Input data on abcissa, p.e. time.
    df : pandas Dataframe (last result of curve_characterizer), optional
		Input, as well as differential quotients.
    dx : float or None, optional
        Test range. The default is None.
    POI_start : str, optional
        Start of point of interest name. The default is 'P'.
    POI_sec : list of strings, optional
        Second part of point of interest name. The default is ['l','u'].
    comp_dfx : string, optional
        Comparision data on df. The default is 'x'.
    rel_df : string, optional
        Relevant data on df for testing (DQ1=ris and DQ2=curve).
        The default is 'DQ2'.
    ascending : bool, optional
        Switch for ascending or descending. The default is True.

    Returns
    -------
    Vren : pd.Series
        Renamer for relevant points.
        
    Example
    -------
    Vren=poi_rel_finder(cip=cip, cycles=cycles, xser=x, 
                        df=dft, dx=x.diff().mean()*(det_dist),
                        POI_start='P', POI_sec=['l','u'],
                        comp_dfx='x', rel_df='DQ2', ascending=True)
    cip.index=cip.index.to_series().replace(Vren).values

    """
    Vren={}
    for cc in np.arange(1,cycles+1):
        for lu in POI_sec:
            dft=pd.DataFrame([])
            dft['V']=cip[cip.index.str.startswith(POI_start+lu+str(cc))]
            if len(dft['V'])<1:
                del dft
                pass
            else:
                dft['xTime'] = xser[dft['V']].values
                dft['tStep'] = dft['xTime'].apply(
                    lambda x: Find_closest(df[comp_dfx],x)
                    )
                if dx is None:
                    dft['test'] = df[rel_df].loc[dft['tStep']].values
                else:
                    if ascending:
                        dft['test']  = dft['xTime'].apply(
                            lambda x: df[rel_df].loc[
                                (df.x>=x-dx/2)&(df.x<=x+dx/2)
                                ].min()
                            )
                    else:
                        dft['test']  = dft['xTime'].apply(
                            lambda x: df[rel_df].loc[
                                (df.x>=x-dx/2)&(df.x<=x+dx/2)
                                ].max()
                            )
                dft=dft.sort_values('test', ascending=ascending)
                Vnew=dft.index.sort_values().values
                dft['Vnew']=Vnew
                Vren.update(dft['Vnew'].copy(deep=True).to_dict())
                del dft,Vnew
    return Vren


[docs]def poi_det_plh(x, y, cps, z=None, dft=None,
            det_dist=2, det_r='RF', refineP=True):
    """
    Automatically determines high and low points, as well of points with with 
    changing rise based on a given curve.

    Parameters
    ----------
    x : pd.Series
        Input data on abcissa, p.e. time.
    y : pd.Series
        Input data on ordinate, p.e. force.
    cps : pandas Dataframe (first result of curve_characterizer)
        Measurement curve characterization.
    z : pd.Series, optional
        Additional input data on ordinate, p.e. displacement. 
        The default is None.
    dft : pandas Dataframe (last result of curve_characterizer), optional
		Input, as well as differential quotients. Used if refineP is True.
        The default is None.
    det_dist : int, optional
        Determination distance. The default is 2.
    det_r : string, optional
        Determination range selection keyword to search in cps.
        The default is 'RF'.
    refineP : bool, optional
        Switch for refinement. The default is True.

    Returns
    -------
    cip : pd.Series
        Points of interest, with index by names and value by index of input.
        data. Labels are 'H'-high point, 'L'-low point, 'Pl'-rise change on 
        loading and 'Pu'-rise change on unloading.
    c : int
        Number of cyclic loadings.
        
    Example
    -------
    if (('preload' in _opts['OPT_Testtype']) 
        or ('cyclic' in _opts['OPT_Testtype'])):
    Vt, cycles = poi_det_plh(x=messu.Time, y=messu.Force, 
                         cps=cps_t, z=messu.Way, dft=df_t,
                         det_dist=_opts['OPT_Determination_Distance'], 
                         det_r='RF', refineP=True)
    VIP_messu = Vt.combine_first(VIP_messu).sort_values().astype(int)

    """
    if det_r == 'RF':
        dr = cps.loc[cps.Type.apply(lambda x: x in ['Rise','Fall'])]
    else:
        dr 
    ib = dr.index[0]
    hl=hu=1
    e=97
    cip = pd.Series([], dtype='int64')
    for i in dr.index[1:]:
        aT=dr.loc[i,  'Type']
        bT=dr.loc[ib, 'Type']
        if z is None:
            j = Find_closest(x, dr.loc[i, 'IntP_x'])
        else:
            j = Find_closestv(x,z, dr.loc[i, 'IntP_x'],dr.loc[i, 'IntP_y'])
        if aT == bT == 'Rise':
            if 'Pl'+str(hl) in cip.index:
                cip['Pl'+str(hl)+chr(e)]=j # wenn bereits Pl vorhanden, dann Plje
                e += 1
            else:
                cip['Pl'+str(hl)]=j
        elif aT == bT == 'Fall':
            if 'Pu'+str(hu) in cip.index:
                cip['Pu'+str(hu)+chr(e)]=j # wenn bereits Pu vorhanden, dann Puje
                e += 1
            else:
                cip['Pu'+str(hu)]=j
        elif bT ==  'Rise' and aT == 'Fall' :
            j = y.loc[j-det_dist//2:j+det_dist//2].idxmax()    
            cip['H'+str(hl)]=j
            hl += 1
            e = 97
        elif bT ==  'Fall' and aT == 'Rise' :
            j = y.loc[j-det_dist//2:j+det_dist//2].idxmin()
            cip['L'+str(hu)]=j
            hu += 1
            e = 97
        ib=i
    c=hu
    if refineP and (not dft is None):
        # Vren=poi_rel_finder(cip=cip, cycles=cycles, xser=x, # cycles may have to be given to function
        Vren=poi_rel_finder(cip=cip, cycles=c, xser=x, 
                           df=dft, dx=x.diff().mean()*(det_dist),
                           POI_start='P', POI_sec=['l','u'],
                           comp_dfx='x', rel_df='DQ2', ascending=True)
        cip.index=cip.index.to_series().replace(Vren).values
    return cip, c


[docs]def poi_vip_namer(VIP, ttype, key,
                  cc=1, mc=1, lu='l'):
    """
    Automatic naming of points of interest to use before poi_fixeva and 
    poi_refinement. Replaces given string by its equivalent for current cycle.
    For example if key is 'P' (may be lower edge of elastic modulus 
    determination range) and lu='l' it will find 'Pl1' for the first cycle and
    'L3' for the third cycle.

    Parameters
    ----------
    VIP : pd.Series
        Identified points of interest. Index by name and value by index of 
        measurement dataframe.
    ttype : string
        Test type, i.e. type of applied loading. Searches only for 'cyclic' 
        and 'destructive'.
    key : string
        Label for naming, possible are 'H'-high point, 'L'-low point, 
        'P'-rise change and 'S'-start.
    cc : int, optional
        Current cycle. The default is 1.
    mc : int, optional
        maximum applied cycles. The default is 1.
    lu : string, optional
        Loading ('l') or unloading ('u'). The default is 'l'.

    Returns
    -------
    string
        Found name of point of interest.
        
    Example
    -------
    for cc in np.arange(1,cycles+1):
        tmp_iS=poi_vip_namer(VIP=VIP_messu,ttype=_opts['OPT_Testtype'],
                              key=_opts['OPT_YM_Determination_range'][-2],
                              cc=cc,mc=cycles,lu='l')
        tmp_iE=poi_vip_namer(VIP=VIP_messu,ttype=_opts['OPT_Testtype'],
                              key=_opts['OPT_YM_Determination_range'][-1],
                              cc=cc,mc=cycles,lu='l')
        tmp,_,_ = poi_fixeva(pds=messu.Stress, 
                            p=_opts['OPT_YM_Determination_range'][0:2],
                            iS=VIP_messu[tmp_iS], iE=VIP_messu[tmp_iE],
                            range_sub='min', norm='max', option='abs',
                            check_irr=True, irr=messu[messu.driF_schg].index.values,
                            irr_opt='nearest')
        VIP_messu['FlA'+str(cc)],VIP_messu['FlB'+str(cc)]=tmp
        if not (('destructive' in _opts['OPT_Testtype']) and cc==cycles):
            tmp_iS=poi_vip_namer(VIP=VIP_messu,ttype=_opts['OPT_Testtype'],
                                 key=_opts['OPT_YM_Determination_range'][-1],
                                 cc=cc,mc=cycles,lu='u')
            tmp_iE=poi_vip_namer(VIP=VIP_messu,ttype=_opts['OPT_Testtype'],
                                  key=_opts['OPT_YM_Determination_range'][-2],
                                  cc=cc,mc=cycles,lu='u')
            tmp,_,_ = poi_fixeva(pds=messu.Stress, 
                                p=_opts['OPT_YM_Determination_range'][0:2],
                                iS=VIP_messu[tmp_iS], iE=VIP_messu[tmp_iE],
                                range_sub='min', norm='max', option='abs',
                                check_irr=True, irr=messu[messu.driF_schg].index.values,
                                irr_opt='nearest')
            VIP_messu['FuB'+str(cc)],VIP_messu['FuA'+str(cc)]=tmp

    """
    def indisin(t,i):
        if t in i.index:
            return True
        else:
            return False

    if key == 'P':
        t=key+lu+str(cc)
        sk = t if indisin(t,VIP) else poi_vip_namer(VIP, ttype, 'L',
                                                    cc, mc, lu)
    elif key == 'H':
        t=key+str(cc)
        sk = t if indisin(t,VIP) else poi_vip_namer(VIP, ttype, 'U',
                                                    cc, mc, lu)
    elif key == 'L':
        if lu == 'l':
            t=key+str(cc-1)
            sk = t if indisin(t,VIP) else poi_vip_namer(VIP, ttype, 'S',
                                                        cc, mc, lu)
        else:
            t=key+str(cc)
            sk = t if indisin(t,VIP) else poi_vip_namer(VIP, ttype, 'E',
                                                        cc, mc, lu)
    elif key == 'S' and cc==mc and lu=='u':
        sk = 'E'
    else:
        sk=key
    
    if ('cyclic' in ttype):
        if key == 'L' and cc==1 and lu=='l':
            sk='Lm' # have to be determined afterwards
        elif key == 'H' and cc==mc and lu=='l'and ('destructive' in ttype):
            sk='Hm' # have to be determined afterwards
    return sk


[docs]def poi_fixeva(pds, p, iS=None, iE=None, 
               range_sub='min', norm='max', option='abs',
               check_irr=False, irr=None, irr_opt='nearest'):
    """
    Determine points for start and end of fixed range determination 
    (by ratios to given value).

    Parameters
    ----------
    pds : pd.Series
        Input values, p.e. measured stress data.
    p : list of two floats
        Lower (first) and upper (second) border of determination range.
    iS : TYPE, optional
        DESCRIPTION. The default is None.
    iE : TYPE, optional
        DESCRIPTION. The default is None.
    range_sub : TYPE, optional
        DESCRIPTION. The default is 'min'.
    norm : TYPE, optional
        DESCRIPTION. The default is 'max'.
    option : TYPE, optional
        DESCRIPTION. The default is 'abs'.
    check_irr : TYPE, optional
        DESCRIPTION. The default is False.
    irr : TYPE, optional
        DESCRIPTION. The default is None.
    irr_opt : TYPE, optional
        DESCRIPTION. The default is 'nearest'.

    Raises
    ------
    NotImplementedError
        DESCRIPTION.

    Returns
    -------
    TYPE
        DESCRIPTION.
    TYPE
        DESCRIPTION.
    TYPE
        DESCRIPTION.
        
    Example
    -------
    for cc in np.arange(1,cycles+1):
        tmp_iS=poi_vip_namer(VIP=VIP_messu,ttype=_opts['OPT_Testtype'],
                              key=_opts['OPT_YM_Determination_range'][-2],
                              cc=cc,mc=cycles,lu='l')
        tmp_iE=poi_vip_namer(VIP=VIP_messu,ttype=_opts['OPT_Testtype'],
                              key=_opts['OPT_YM_Determination_range'][-1],
                              cc=cc,mc=cycles,lu='l')
        tmp,_,_ = poi_fixeva(pds=messu.Stress, 
                            p=_opts['OPT_YM_Determination_range'][0:2],
                            iS=VIP_messu[tmp_iS], iE=VIP_messu[tmp_iE],
                            range_sub='min', norm='max', option='abs',
                            check_irr=True, irr=messu[messu.driF_schg].index.values,
                            irr_opt='nearest')
        VIP_messu['FlA'+str(cc)],VIP_messu['FlB'+str(cc)]=tmp
        if not (('destructive' in _opts['OPT_Testtype']) and cc==cycles):
            tmp_iS=poi_vip_namer(VIP=VIP_messu,ttype=_opts['OPT_Testtype'],
                                 key=_opts['OPT_YM_Determination_range'][-1],
                                 cc=cc,mc=cycles,lu='u')
            tmp_iE=poi_vip_namer(VIP=VIP_messu,ttype=_opts['OPT_Testtype'],
                                  key=_opts['OPT_YM_Determination_range'][-2],
                                  cc=cc,mc=cycles,lu='u')
            tmp,_,_ = poi_fixeva(pds=messu.Stress, 
                                p=_opts['OPT_YM_Determination_range'][0:2],
                                iS=VIP_messu[tmp_iS], iE=VIP_messu[tmp_iE],
                                range_sub='min', norm='max', option='abs',
                                check_irr=True, irr=messu[messu.driF_schg].index.values,
                                irr_opt='nearest')
            VIP_messu['FuB'+str(cc)],VIP_messu['FuA'+str(cc)]=tmp

    """
    tmp=Find_closest_perc(
        pds=pds, p=p, iS=iS, iE=iE, range_sub=range_sub, 
        norm=norm, option=option
        )
    irrb = None
    irrpos = None
    if check_irr is True and irr is not None: 
        def c_nba(r,irr,irr_opt):
            i=''
            if irr < max(r) and irr > min(r):
                b = True
            else:
                b = False
            if b:
                if irr_opt in ['n','nearest']:
                    i=Find_closest(pds=pd.Series(r),val=irr,option='abs')
                elif irr_opt in ['b','lower','first']:
                    i=0
                elif irr_opt in ['a','higher','last']:
                    i=1
                else:
                    raise NotImplementedError('Option not implemented!')                    
                r[i]=irr
            return r, b, i
                    
        if   isinstance(irr, (float,int)):
            tmp, irrb, irrpos = c_nba(tmp,irr,irr_opt)
        elif isinstance(irr, (list,np.ndarray)) or isinstance(irr,pd.core.base.ABCSeries):
            irrb=[]
            irrpos=[]
            # if not isinstance(irr, pd.core.base.ABCSeries):
            #     irr = pd.Series(irr)
            for i in irr:
                tmp, irrbt, irrpost = c_nba(tmp,i,irr_opt)
                irrb.append(irrbt)
                irrpos.append(irrpost)
        else:
            raise NotImplementedError("Type %s for irregularity not implemented"%type(irr))
    return tmp, irrb, irrpos


[docs]def poi_refinement(DQ, B_ser, s_range, B_ind=None, names=['F3','M','F4'],
                   Blp=0.15, Bup=0.95, Maxp=0.75, trimsch=True):
    """
    Refine elastic modulus determination range according to a method described 
    by Keuerleber [1].
    ([1]: Keuerleber, M. (2006). Bestimmung des Elastizitätsmoduls von 
    Kunststoffen bei hohen Dehnraten am Beispiel von PP. Von der Fakultät 
    Maschinenbau der Universität Stuttgart zur Erlangung der Würde eines 
    Doktor-Ingenieurs (Dr.-Ing.) genehmigte Abhandlung. Doktorarbeit. 
    Universität Stuttgart, Stuttgart.)

    Parameters
    ----------
    DQ : pd.DataFrame
        Stress-strain-curve differential quotients and sign changes. 
        Generated by Diff_Quot.
    B_ser : pd.Series
        Input data, p.e. stress, to search for given lower limit ratio 
        (Blp) und upper limit ratio (Bup) for refined range.
    s_range : list
        Start and end of step range to search for refined range.
    B_ind : int, optional
        Index for B_ser. The default is None. Not implemented yet 
        (using absolute maximum of given B_ser values instead).
    names : list of string, optional
        Output names for lower limit, maximum of differental quotient 
        and upper limit. The default is ['F3','M','F4'].
    Blp : float, optional
        Lower limit ratio to identified B_ser value (p.e. maximum stress).
        The default is 0.15.
    Bup : float, optional
        Upper limit ratio to identified B_ser value (p.e. maximum stress).
        The default is 0.95.
    Maxp : float, optional
        Lowest value to include in refined range. The default is 0.75. 
        (i.e. 75 % of the maximum differntail quotient)
    trimsch : bool, optional
        Switch for trimming step range of differential quotients. 
        The default is True.

    Returns
    -------
    VIP : pd.Series
        Determined points of interest as series of steps indexed by given names.
    DQs : pd.DataFrame 
        Differential quotients limitet to range.
    
    Example
    -------
    DQcons=None
    DQopts=None
    for cc in np.arange(1,cycles+1):
        tmp_iS=poi_vip_namer(VIP=VIP_messu,ttype=_opts['OPT_Testtype'],
                              key=_opts['OPT_YM_Determination_refinement'][2],
                              cc=cc,mc=cycles,lu='l')
        tmp_iE=poi_vip_namer(VIP=VIP_messu,ttype=_opts['OPT_Testtype'],
                              key=_opts['OPT_YM_Determination_refinement'][3],
                              cc=cc,mc=cycles,lu='l')
        tmp,DQcont = poi_refinement(DQ=DQcon, B_ser=messu.loc(axis=1)['Stress'],
                            s_range=[VIP_messu[tmp_iS],VIP_messu[tmp_iE]],
                            names=['RlA'+str(cc),'RlM'+str(cc),'RlB'+str(cc)],
                            Blp=_opts['OPT_YM_Determination_refinement'][0],
                            Bup=1.0-_opts['OPT_YM_Determination_refinement'][0],
                            Maxp=_opts['OPT_YM_Determination_refinement'][1])
        VIP_messu=tmp.combine_first(VIP_messu).sort_values().astype(int)
        if DQcons is None:
            DQcons = DQcont
        else:
            DQcons=pd.concat([DQcons,DQcont],axis=0)
        tmp,DQoptt = poi_refinement(DQ=DQopt, B_ser=messu.loc(axis=1)['Stress'],
                            s_range=[VIP_messu[tmp_iS],VIP_messu[tmp_iE]],
                            names=['RlA'+str(cc),'RlM'+str(cc),'RlB'+str(cc)],
                            Blp=_opts['OPT_YM_Determination_refinement'][0],
                            Bup=1.0-_opts['OPT_YM_Determination_refinement'][0],
                            Maxp=_opts['OPT_YM_Determination_refinement'][1])
        VIP_dicu=tmp.combine_first(VIP_dicu).sort_values().astype(int)
        if DQopts is None:
            DQopts = DQoptt
        else:
            DQopts=pd.concat([DQopts,DQoptt],axis=0)
            
            
        if not (('destructive' in _opts['OPT_Testtype']) and cc==cycles):
            tmp_iS=poi_vip_namer(VIP=VIP_messu,ttype=_opts['OPT_Testtype'],
                                 key=_opts['OPT_YM_Determination_refinement'][3],
                                 cc=cc,mc=cycles,lu='u')
            tmp_iE=poi_vip_namer(VIP=VIP_messu,ttype=_opts['OPT_Testtype'],
                                  key=_opts['OPT_YM_Determination_refinement'][2],
                                  cc=cc,mc=cycles,lu='u')
            tmp,DQcont = poi_refinement(DQ=DQcon, B_ser=messu.loc(axis=1)['Stress'],
                                s_range=[VIP_messu[tmp_iS],VIP_messu[tmp_iE]],
                                names=['RuA'+str(cc),'RuM'+str(cc),'RuB'+str(cc)],
                                Blp=_opts['OPT_YM_Determination_refinement'][0],
                                Bup=1.0-_opts['OPT_YM_Determination_refinement'][0],
                                Maxp=_opts['OPT_YM_Determination_refinement'][1])
            VIP_messu=tmp.combine_first(VIP_messu).sort_values().astype(int)
            if DQcons is None:
                DQcons = DQcont
            else:
                DQcons=pd.concat([DQcons,DQcont],axis=0)
            tmp,DQoptt = poi_refinement(DQ=DQopt, B_ser=messu.loc(axis=1)['Stress'],
                                s_range=[VIP_messu[tmp_iS],VIP_messu[tmp_iE]],
                                names=['RuA'+str(cc),'RuM'+str(cc),'RuB'+str(cc)],
                                Blp=_opts['OPT_YM_Determination_refinement'][0],
                                Bup=1.0-_opts['OPT_YM_Determination_refinement'][0],
                                Maxp=_opts['OPT_YM_Determination_refinement'][1])
            VIP_dicu=tmp.combine_first(VIP_dicu).sort_values().astype(int)
            if DQopts is None:
                DQopts = DQoptt
            else:
                DQopts=pd.concat([DQopts,DQoptt],axis=0)

    """
    VIP=pd.Series([], dtype='int64')
    step_range=pd_combine_index(
        DQ.index, B_ser.loc[min(s_range):max(s_range)].index
        )
    
    B_range=Find_closest_perc(pds=B_ser, p=[Blp,Bup],
                                   iS=min(s_range), iE=max(s_range),
                                   range_sub='min', norm='max', option='abs')
    B_range=B_ser.indlim(*[min(B_range),max(B_range)]).index
    #B_range=B_ser[(B_ser>=Blp*B_ser.loc[B_ind])&(B_ser<=Bup*B_ser.loc[B_ind])].index
    DQs=DQ.loc[step_range]
    if trimsch:
        t=DQs[DQs['DQ1_signchange']]
        t2=pd.Series(t.index.values, index=t.index)
        t4=[DQs.iloc[0].name,DQs.iloc[-1].name]
        t2=t2.append(pd.Series(t4, index=t4))
        t2=t2.drop_duplicates().sort_index()
        t3=t2.diff()
        step_range=[t2[t2<t3.idxmax()].iloc[-1],t3.idxmax()-1]
        DQs=DQs.loc[pd_slice_index(DQs.index,step_range)]            
    step_range2=pd_combine_index(DQs.index, B_range)
    VIP[names[1]]=DQs.loc[step_range2].DQ1.idxmax()
    DQM=DQs.DQ1.loc[VIP[names[1]]]
    try:
        VIP[names[0]]=DQs.loc[:VIP[names[1]]].iloc[::-1].loc[(DQs.DQ1/DQM)<Maxp].index[0]+1
    except IndexError:
        VIP[names[0]]=DQs.index[0]
    try: # Hinzugefügt am 16.09.2021
        VIP[names[2]]=DQs.loc[VIP[names[1]]:].loc[(DQs.DQ1/DQM)<Maxp].index[0]-1
    except IndexError:
        VIP[names[2]]=VIP[names[1]]-1 #-1 could be problematic
    VIP=VIP.sort_values()
    return VIP, DQs
```

---

© Copyright 2024, MarcGebhardt.

Built with Sphinx using a
theme
provided by Read the Docs.
