# Supplementary material for: Approach to Standardized Material Characterization of the Human Lumbopelvic System: Testing and Evaluation
Source: Bioengineering (Basel). 2025 Aug 11;12(8):862. doi: 10.3390/bioengineering12080862 (PMC12383908; doi:10.3390/bioengineering12080862)
Supplement: Supplementary file 1 [file bioengineering-12-00862-s001.zip › File S3 Evaluation code/ExMechEva-0.1.2/docs/_build/html/_modules/exmecheva/common/mc_man.html]

exmecheva.common.mc\_man — ExMechEva 0.1.0 documentation


ExMechEva

Contents:

- ExMechEva

ExMechEva

- Module code
- exmecheva.common.mc\_man

---

# Source code for exmecheva.common.mc\_man

```
# -*- coding: utf-8 -*-
"""
Adds functionality to temporary manipulate measurement curves.

@author: MarcGebhardt
"""
import warnings
import numpy as np
import pandas as pd
import scipy

from .pd_ext import (pd_isSer)

#%% Extension and retrimming

[docs]def Extend_Series_Poly(y, n=3, polydeg=1, kind='fb'):
    """
    Extend an Array or series with polynomial values.

    Parameters
    ----------
    y : numpy.array or pandas.Series
        Input values.
    n : int or list of or dict of kind{'f':int,'b':int}
        Number of points to extend.
        Must be at least equal to the order of the polynomial.
        Could be 0 (skipping extension).
    polydeg : int or list of or dict of kind{'f':int,'b':int}, optional
        Order of polynom. The default is 1.
    kind : str, optional
        Kind of extension ('f'-forward, 'b'-backward and 'fb'-both).
        The default is 'fb'.

    Raises
    ------
    TypeError
        DESCRIPTION.

    Returns
    -------
    yout : numpy.array or pandas.Series
        Extended input values.

    """
    if isinstance(n, int):
        nf, nb = n, n
    elif isinstance(n, list):
        nf, nb = n
    elif isinstance(n, dict):
        nf, nb = n['f'],n['b']
    else:
        raise TypeError("Type %s for parameter n not implemented!"%type(n))
    if kind =='f': nb=0
    if kind =='b': nf=0
    if isinstance(polydeg, int):
        pdegf, pdegb = polydeg, polydeg
    elif isinstance(polydeg, list):
        pdegf, pdegb = polydeg
    elif isinstance(polydeg, dict):
        pdegf, pdegb = polydeg['f'], polydeg['b']
    else:
        raise TypeError("Type %s for parameter polydeg not implemented!"%type(polydeg))
        
    
    if isinstance(y, pd.core.base.ABCSeries):
        ya = y.values
        xa = y.index.values
        t = 'Series'
    elif isinstance(y, np.ndarray):
        ya = y
        ydt = ya.dtype
        xa = np.arange(len(y))
        t = 'Array'
    else:
        raise TypeError("Type %s not implemented!"%type(y))
        
    ydt = ya.dtype
    xdt = xa.dtype
        
    if (kind in ['f','fb']) and (nf > 0):
        yf_p = np.polyfit(xa[:nf+1],  ya[:nf+1],  pdegf)
        xf = xa[0] - np.cumsum(np.diff(xa[:nf+1]))[::-1]
        yf = np.polyval(yf_p, xf)
    else:
        xf = np.array([], dtype=xdt)
        yf = np.array([], dtype=ydt)
    if (kind in ['b','fb']) and (nb > 0):
        yb_p = np.polyfit(xa[-(nb+1):], ya[-(nb+1):], pdegb)
        xb = xa[-1] - np.cumsum(np.diff(xa[-1:-(nb+2):-1]))
        yb = np.polyval(yb_p, xb)
    else:
        xb = np.array([], dtype=xdt)
        yb = np.array([], dtype=ydt)

    yout = np.concatenate((yf, ya, yb), axis=0)
    xout = np.concatenate((xf, xa, xb), axis=0)
    if t == 'Series':
        yout = pd.Series(yout, index=xout, name=y.name)
    return yout


[docs]def Extend_Series_n_setter(ffunctype='Smooth', ffuncargs=(), ffunckwargs={}):
    """
    Sets window length for extending of series by given aspiired function.

    Parameters
    ----------
    ffunctype : function, optional
        Function to use after extension. The default is 'Smooth'.
    ffuncargs : list, optional
        Arguments for function. The default is ().
    ffunckwargs : dict, optional
        Keyword arguments for function. The default is {}.

    Raises
    ------
    ValueError
        No valid value found for extension length.

    Returns
    -------
    ntmp : integer
        Extension length.

    """
    ntmp=None
    if ffunctype=='Smooth':
        idks=['window_length','size','box','box_pts']
        for idk in idks:
            if idk in ffunckwargs.keys():
                ntmp=ffunckwargs[idk]
            else:
                no_idk=True
        if no_idk and isinstance(ffuncargs, list):
            for a in ffuncargs[::-1]:
                if type(a) is int:
                    ntmp = a
        elif no_idk and isinstance(ffuncargs,int):
            ntmp = ffuncargs
        else:
            ntmp = 1
        ntmp = (ntmp -1)//2
        
    elif ffunctype in ['Diff','diff','Shift','shift']:
        idks=['periods','shift','shift_value']
        for idk in idks:
            if idk in ffunckwargs.keys():
                ntmp=ffunckwargs[idk]
            else:
                no_idk=True
        if no_idk and isinstance(ffuncargs, list):
            for a in ffuncargs[::-1]:
                if type(a) is int:
                    ntmp = a
        elif no_idk and isinstance(ffuncargs,int):
            ntmp = ffuncargs
        ntmp = abs(ntmp)
    if ntmp is None:
        raise ValueError('Extension length not specified and no valid key found in smoothing options!')
    return ntmp


[docs]def Retrim_Series(y, axis=0, n=0, kind='fb'):
    """
    Retrim an Array or series after extension.

    Parameters
    ----------
    y : numpy.array or pandas.Series or pandas.DataFrame
        Input values.
    n : int or list of or dict of kind{'f':int,'b':int}
        Number of points which are extend.
        Could be 0 (skip retrimming).
    kind : str, optional
        Kind of extension ('f'-front, 'b'-back and 'fb'-both).
        The default is 'fb'.

    Raises
    ------
    TypeError
        DESCRIPTION.

    Returns
    -------
    yout : numpy.array or pandas.Series
        Extended input values.

    """
    if isinstance(n, int):
        nf, nb = n, n
    elif isinstance(n, list):
        nf, nb = n
    elif isinstance(n, dict):
        nf, nb = n['f'],n['b']
    else:
        raise TypeError("Type %s for parameter n not implemented!"%type(n))

    if kind =='f': nb=0
    if kind =='b': nf=0

    if isinstance(y, pd.core.base.ABCSeries):
        yt='Series'
    elif isinstance(y, pd.core.base.ABCDataFrame):
        yt='DataFrame'
    elif isinstance(y, np.ndarray):
        yt='Array'
        y=pd.Series(y)
    else:
        raise TypeError("Type %s not implemented!"%type(y))
    indf =  nf if nf > 0 else None
    indb = -nb if nb > 0 else None
    if yt == 'DataFrame':
        yout = y.iloc(axis=axis)[indf:indb]
    else:
        yout = y.iloc[indf:indb]
    if yt == 'Array':
        yout = yout.values
        
    return yout


[docs]def check_params(test_val, test_var='n', 
                 func=Extend_Series_Poly, 
                 args=(), kwargs={'polydeg':1}):
    """
    Checkes if tested values merge with given function.

    Parameters
    ----------
    test_val : TYPE
        Variable value to test.
    test_var : TYPE, optional
        Variable name. The default is 'n'.
    func : function, optional
        Function to test. Implemented are:
            - Extend_Series_Poly
        The default is Extend_Series_Poly.
    args : list, optional
        Arguments for function. The default is ().
    kwargs : dict, optional
        Keyword arguments for function. The default is {'polydeg':1}.

    Raises
    ------
    NotImplementedError
        Function not implemented.

    Returns
    -------
    leg_val : bool
        Indicates if the input value is legitimate.
    adj_val : TYPE
        Adjusted value.
    leg_str : TYPE
        Output string.

    """
    if func == Extend_Series_Poly:
        if test_var=='n' and test_val!=0:
            if 'polydeg' in kwargs.keys():
                ref_val = kwargs['polydeg']
            else:
                ref_val = 1 # min order of polynom
            test = (test_val/ref_val) >= 1
            adj = ref_val
            ref_var = 'polydeg'
            ref_cond = 'equal'
    else:
        raise NotImplementedError("Option %s not implemented!"%func)
    if test:
        leg_val = True
        adj_val = test_val
        leg_str = test_var+' is '+ref_cond+' then '+ref_var+' for '+str(func)
    else:
        leg_val = False
        adj_val = adj
        leg_str = test_var+' have to be '+ref_cond+' then '+ref_var+' for '+str(func)
        leg_str += '\n --> new value set to {}'.format(adj_val)
    return leg_val, adj_val, leg_str


[docs]def Predict_apply_retrim(x, afunc=None, pfunc=Extend_Series_Poly, 
                         aargs=[], akwargs={}, 
                         pargs=[], pkwargs={'n':3, 'polydeg':1, 'kind':'fb'},
                         shift_value=0, fill_value=None):
    """
    Predict input over their boundary values, apply a function and 
    retrim to original size.

    Parameters
    ----------
    x : numpy.array or pandas.Series
        Input values.
    afunc : function, optional
        Function for manipulation to apply after extending the input values. 
		The default is None.
    pfunc : function, optional
        Function for extending the input values. 
		The default is Extend_Series_Poly.
    aargs : list, optional
        Arguments passed to manipulation funtion (afunc). 
		The default is [].
    akwargs : dict, optional
        Keyword arguments passed to manipulation funtion (afunc). 
		The default is {}.
    pargs : list, optional
        Arguments passed to extening funtion (pfunc). 
		The default is [].
    pkwargs : dict, optional
        Keyword arguments passed to extening funtion (pfunc). 
		The default is {'n':1, 'polydeg':1, 'kind':'fb'}.
    shift_value : int, optional
        Value for shifting. The default is 0.
    fill_value : int or None, optional
        Value for filling free values after shifting. 
		The default is None, which apply self.dtype.na_value.

    Raises
    ------
    TypeError
        Not expected data type.

    Returns
    -------
    xout : numpy.array or pandas.Series
        Manipualted output values.

    """
       
    if isinstance(x, pd.core.base.ABCSeries):
        xt='Series'
    elif isinstance(x, np.ndarray):
        xt='Array'
    else:
        raise TypeError("Type %s not implemented!"%type(x))
    
    if isinstance(pkwargs['n'], int):
        nf, nb = pkwargs['n'], pkwargs['n']
    elif isinstance(pkwargs['n'], list):
        nf, nb = pkwargs['n']
    elif isinstance(pkwargs['n'], dict):
        nf, nb = pkwargs['n']['f'],pkwargs['n']['b']
    else:
        raise TypeError("Type %s for parameter n not implemented!"%type(pkwargs['n']))
        
    xlen = pfunc(x, *pargs, **pkwargs)
    if afunc is None:
        xman = xlen
    else:
        xman = afunc(xlen, *aargs, **akwargs)
        
    if xt == 'Array':
        xman = pd.Series(xman)
        
    if not shift_value == 0: xman = xman.shift(periods=shift_value, 
                                               fill_value=fill_value)
    indf =  nf if nf > 0 else None
    indb = -nb if nb > 0 else None
    xout = xman.iloc[indf:indb]
    if xt == 'Array':
        xout = xout.values
    
    return xout


[docs]def Diff_ext(x, periods=1, axis=0, pfunc=Extend_Series_Poly,
             pkwargs={}, shift_value=0, fill_value=None):
    """
    Calculates the difference of input values after extension and retrims.

    Parameters
    ----------
    x : pd.Series or pd. DataFrame
        Input values.
    periods : int, optional
        Periods to shift (see pandas diff). The default is 1.
    axis : integer, optional
        Axis to apply. The default is 0.
    pfunc : function, optional
        Extension function. The default is Extend_Series_Poly.
    pkwargs : dict, optional
        Keyword arguments passed to extening funtion (pfunc). 
		The default is {}.
    shift_value : int, optional
        Value for shifting. The default is 0.
    fill_value : int or None, optional
        Value for filling free values after shifting. 
		The default is None, which apply self.dtype.na_value.

    Raises
    ------
    TypeError
        DESCRIPTION.

    Returns
    -------
    xout : pd.Series or pd. DataFrame
        Output values.

    """
    
    if isinstance(x, pd.core.base.ABCSeries):
        xt='1D'
        afunc = pd.Series.diff
        akwargs = {'periods':periods}
    elif isinstance(x, pd.core.base.ABCDataFrame):
        xt='2D'
        afunc = pd.Series.diff
        akwargs = {'periods':periods}
    elif isinstance(x, np.ndarray):
        if len(x.shape) >1:
            raise TypeError("Array with shape %d not implemented (only 1D)!"%len(x.shape))
        else:
            xt='1D'
            afunc = np.diff
            akwargs = {'periods':periods, 'axis':axis, 
                       'prepend':[np.nan,]*periods}
    else:
        raise TypeError("Type %s not implemented!"%type(x))
        
    if not 'n' in pkwargs.keys():
        pkwargs['n']=abs(periods)
        
    n_check = check_params(test_val=pkwargs['n'], test_var='n',
                           # func=Extend_Series_Poly, kwargs=pkwargs)
                           func=pfunc, kwargs=pkwargs)
    if not n_check[0]:
        warnings.warn('\n Automatic set extension within Smoothsel_ext too small'+n_check[2])
        pkwargs['n'] = n_check[1]
        
    if xt == '2D':
        xout = x.apply(Predict_apply_retrim, axis=axis,
                       **{'afunc':afunc, 'pfunc':pfunc, 
                          'aargs':[], 'akwargs':akwargs, 
                          'pargs':[], 'pkwargs':pkwargs,
                          'shift_value':shift_value, 
                          'fill_value':fill_value})
    else:
        xout = Predict_apply_retrim(x, afunc=afunc,
                                    pfunc=pfunc, 
                                    aargs=[], akwargs=akwargs, 
                                    pargs=[], pkwargs=pkwargs,
                                    shift_value=shift_value, 
                                    fill_value=fill_value)
    return xout

#%% Smoothing

[docs]def smooth(y, box_pts): 
    """
    Computes moving average of y about distance of box_pts.

    Parameters
    ----------
    y : array of float
        Input data.
    box_pts : integer
        Window length.

    Returns
    -------
    y_smooth : array of float
        Output data.

    """
    warnings.warn("Method will be replaced by Smoothsel", DeprecationWarning)
    box = np.ones(box_pts)/box_pts 
    y_smooth = np.convolve(y, box, mode='same') 
    return y_smooth


[docs]def Smoothsel(x, smooth_type='SMA',
              # smooth_opts={'window_length':3, 'mode':'same'},
              smooth_opts={'window_length':3, 'mode':'nearest'},
              snip=False, conv_method='scipy'):
    """
    Computes a smoothed version of an input array, according to different 
    smoothing types.

    Parameters
    ----------
    x : array or pandas.Series of float
        Input values.
    smooth_type : string, case-sensitive, optional
        Choosen smoothing type.
        Optional. The defalut is 'SMA'.
        Possible are:
            - 'SMA': Simple moving average based on numpy.convolve.
            - 'BMA': Moving average with binomial coefficents based on numpy.convolve.
            - 'SMA_f1d': Simple moving average based scipy.ndimage.filters.uniform_filter1d.
            - 'SavGol': Savitzky-Golay filter based on scipy.signal.savgol_filter.
    smooth_opts : dict, optional
        Keywords and values to pass to smoothing method. 
        For further informations see smooth_type and linked methods.
        Optional. The defalut is {'window_length':3, 'mode':'nearest'}.
    snip : bool or integer, optional
        Trimming of output. Either, if True with (window_length-1)//2 in smooth_opts,
        none if False, or with inserted distance. 
        The default is False.
    conv_method : string, optional
        Convolve method to use ('numpy' for numpy.convolve,
                                'scipy' for scipy.ndimage.convolve1d)
        The default is 'scipy'.

    Raises
    ------
    TypeError
        Type not expected.
    NotImplementedError
        Not implemented.

    Returns
    -------
    out : array or pandas.Series of float
        Output values.

    """
    if conv_method == 'numpy':
        conmet = np.convolve
        box_mode_std = 'same'
    elif conv_method == 'scipy':
        conmet = scipy.ndimage.convolve1d
        box_mode_std = 'nearest'
    elif conv_method == '2D':
        conmet = scipy.signal.convolve2d
        box_mode_std = 'same'
        raise NotImplementedError("Convolving method %s not implemented!"%conv_method)
    else:        
        raise NotImplementedError("Convolving method %s not implemented!"%conv_method)
        
    if isinstance(x, pd.core.base.ABCSeries):
        #x = x.dropna() #???
        xa = x.values
        xt='Series'
        xi = x.index.values # nur für Kompatibilität USplineA
    elif isinstance(x, np.ndarray):
        xa = x
        xt='Array'
        xi = np.linspace(0,len(x),len(x)) # nur für Kompatibilität USplineA
    else:
        raise TypeError("Type %s not implemented!"%type(x))

    if smooth_type=='SMA':
        if isinstance(smooth_opts,int):
            box_pts  = smooth_opts
            box_mode = box_mode_std
        elif isinstance(smooth_opts,dict):
            box_pts  = smooth_opts['window_length']
            if not 'mode' in smooth_opts.keys():
                smooth_opts['mode'] = box_mode_std
            box_mode = smooth_opts['mode']
        else:
            raise TypeError("Unexpected type of smooth option (%s)."%type(smooth_opts))
        out=conmet(xa, np.ones(box_pts)/box_pts, mode=box_mode)
    
    elif smooth_type=='USplineA':
        box_pts  = 2 # Kompatibilität
        spline=scipy.interpolate.UnivariateSpline(xi, xa, **smooth_opts)
        out=spline(xi)
    
    elif smooth_type=='BMA':
        def binomcoeffs(n): 
            return (np.poly1d([0.5, 0.5])**n).coeffs
        if isinstance(smooth_opts,int):
            box_pts  = smooth_opts
            box_mode = box_mode_std
        elif isinstance(smooth_opts,dict):
            box_pts  = smooth_opts['window_length']
            if not 'mode' in smooth_opts.keys():
                smooth_opts['mode'] = box_mode_std
            box_mode = smooth_opts['mode']
        else:
            raise TypeError("Unexpected type of smooth option (%s)."%type(smooth_opts))
        out=conmet(xa, binomcoeffs(box_pts-1), mode=box_mode)
        
    elif smooth_type=='SMA_f1d':
        if isinstance(smooth_opts,int):
            box_pts  = smooth_opts
            orig = -(smooth_opts//2)
            box_mode = 'constant'
            smooth_opts  = {'size': smooth_opts, 'mode': box_mode,
                            'origin': orig}
        elif isinstance(smooth_opts,dict):
            box_pts  = smooth_opts['size']
            if not 'origin' in smooth_opts.keys():
                smooth_opts['origin'] = -(box_pts//2)
            if not 'mode' in smooth_opts.keys():
                smooth_opts['mode'] = 'constant'
        else:
            raise TypeError("Unexpected type of smooth option (%s)."%type(smooth_opts))
        out = scipy.ndimage.filters.uniform_filter1d(xa, **smooth_opts)
        
    elif smooth_type=='SavGol':
        box_pts  = smooth_opts['window_length']
        out = scipy.signal.savgol_filter(xa,**smooth_opts)
    else:
        raise NotImplementedError("Smoothing type %s not implemented!"%smooth_type)
        
    if xt=='Series':
        out = pd.Series(out, index=x.index, name=x.name)
    if not snip is False:
        if snip is True:
            # out = out[:-(box_pts-1)]
            out = out[:-(box_pts-1)//2]
        else:
            out = out[:-snip]
    return out


[docs]def Smoothsel_ext(x, axis=0, smooth_type='SMA',
                  smooth_opts={'window_length':3, 'mode':'nearest'},
                  snip=False, conv_method='scipy',
                  pfunc=Extend_Series_Poly,
                  pkwargs={}, shift_value=0, fill_value=None,
                  so_idks=['window_length','size','box','box_pts']):
    """
    Smoothes extended data and retrimes afterwards. 

    Parameters
    ----------
    x : pd.Series or pd.DataFrame
        Input data.
    axis : int, optional
        2D axis to apply. The default is 0.
    smooth_type : string, case-sensitive, optional
        Choosen smoothing type.
        Optional. The defalut is 'SMA'.
        Possible are:
            - 'SMA': Simple moving average based on numpy.convolve.
            - 'BMA': Moving average with binomial coefficents based on numpy.convolve.
            - 'SMA_f1d': Simple moving average based scipy.ndimage.filters.uniform_filter1d.
            - 'SavGol': Savitzky-Golay filter based on scipy.signal.savgol_filter.
    smooth_opts : dict, optional
        Keywords and values to pass to smoothing method. 
        For further informations see smooth_type and linked methods.
        Optional. The defalut is {'window_length':3, 'mode':'nearest'}.
    snip : bool or integer, optional
        Trimming of output. Either, if True with (window_length-1)//2 in smooth_opts,
        none if False, or with inserted distance. 
        The default is False.
    conv_method : string, optional
        Convolve method to use ('numpy' for numpy.convolve,
                                'scipy' for scipy.ndimage.convolve1d)
        The default is 'scipy'.
    pfunc : function, optional
        Function for extension. The default is Extend_Series_Poly.
    pkwargs : dict, optional
        Keywords for extension function (pfunc). The default is {}.
    shift_value : int, optional
        Value for shifting. The default is 0.
    fill_value : int or None, optional
        Value for filling free values after shifting. 
		The default is None, which apply self.dtype.na_value.
    so_idks : list of string, optional
        Smoothing options Keywords to search for extension length. 
        The default is ['window_length','size','box','box_pts'].

    Raises
    ------
    TypeError
        Input type error.
    ValueError
        No extension length found.

    Returns
    -------
    xout : pd.Series or pd.DataFrame
        Output data.

    """
    
    akwargs = {'smooth_type':smooth_type, 'smooth_opts':smooth_opts,
               'snip':snip, 'conv_method':conv_method}
    
    if isinstance(x, pd.core.base.ABCSeries):
        xt='1D'
    elif isinstance(x, pd.core.base.ABCDataFrame):
        xt='2D'
    elif isinstance(x, np.ndarray):
        if len(x.shape) >1:
            raise TypeError("Array with shape %d not implemented (only 1D)!"%len(x.shape))
        else:
            xt='1D'
    else:
        raise TypeError("Type %s not implemented!"%type(x))
        
    if not 'n' in pkwargs.keys():
        if isinstance(smooth_opts,int):
            ntmp=smooth_opts
        elif isinstance(smooth_opts,dict):
            for so_idk in so_idks:
                if so_idk in smooth_opts.keys():
                    ntmp=smooth_opts[so_idk]
                else:
                    raise ValueError('Extension length not specified and no valid key found in smoothing options!')
        else:
            raise TypeError("Type %s not implemented to calculate extension length!"%type(smooth_opts))
        ntmp = (ntmp-1)//2
        n_check = check_params(test_val=ntmp, test_var='n',
                               # func=Extend_Series_Poly, kwargs=pkwargs)
                               func=pfunc, kwargs=pkwargs)
        if not n_check[0]:
            warnings.warn('\n Automatic set extension within Smoothsel_ext too small'+n_check[2])
            pkwargs['n'] = n_check[1]
        else:
            pkwargs['n']=ntmp
        
    if xt == '2D':
        xout = x.apply(Predict_apply_retrim, axis=axis,
                       **{'afunc':Smoothsel, 'pfunc':pfunc, 
                          'aargs':[], 'akwargs':akwargs, 
                          'pargs':[], 'pkwargs':pkwargs,
                          'shift_value':shift_value, 
                          'fill_value':fill_value})
    else:
        xout = Predict_apply_retrim(x, afunc=Smoothsel,
                                    pfunc=pfunc, 
                                    aargs=[], akwargs=akwargs, 
                                    pargs=[], pkwargs=pkwargs,
                                    shift_value=shift_value, 
                                    fill_value=fill_value)
                        
    return xout

#%% Resampling

[docs]def mc_resampler(mdf, t_col='Time', 
                 resample=True, res_frequ=4, 
                 move_ave=True, ma_sampler='data_rf',
                 rel_time_digs=2):
    """
    Resample measured data to aspired frequancy, with or without moving 
    average.
    Only tested with downsampling and integer frequency;
    fixed time format 's' and 3 digits for resampling.
    TODO: Implement mc_smoothing.Smoothsel_ext 
          (extending, smoothing and retrimming).

    Parameters
    ----------
    mdf : pd.DataFrame
        Time depend measured data.
    t_col : string, optional
        Name of time column. The default is 'Time'.
    resample : bool, optional
        Resampling switch. The default is True.
    res_frequ : int, optional
        Aspired output frequency. The default is 4.
    move_ave : bool, optional
        Switch for moving average. The default is True.
    ma_sampler : string or int, optional
        Sampler for moving average. Can ether be option 'data_rf' 
        (sampler derived from quotient of present to aspired frequency) or 
        integer number. The default is 'data_rf'.
    rel_time_digs : int, optional
        Relevant digits for time variable. The default is 2.

    Returns
    -------
    mdf : pd.DataFrame
        Resampled measured data.

    """
    m_dt = mdf[t_col].diff().mean()
    m_f = round((1/m_dt),1)
    
    if resample:
        if move_ave: #TODO 
            if ma_sampler == 'data_rf':
                sampler = m_f/res_frequ
            elif isinstance(ma_sampler, int):
                sampler = ma_sampler
            else:
                raise ValueError("Moving average sampler must be ether 'data_rf' or integer!")
            m1 = mdf.transform(
                lambda x: np.convolve(
                    x, np.ones(int(sampler))/sampler, mode='same'
                    ))
            m1[t_col]=mdf[t_col]
            mdf=m1
            
        a = mdf[t_col].loc[
            (np.mod(mdf[t_col],1/res_frequ)==0)&(mdf[t_col]>=0)
            ].index[1]
        ti = pd.TimedeltaIndex(
            data=mdf[t_col][mdf[t_col]>=mdf[t_col].loc[a]],
            unit='s',name='dictimedelta'
            )
        m2 = mdf[mdf[t_col]>=mdf[t_col].loc[a]].set_index(ti,drop=True)
        m2 = m2.resample('%.3fS'%(1/res_frequ))
        mdf = m2.interpolate(method='linear',limit_area='inside',limit=8)
        mdf[t_col] = mdf[t_col].round(rel_time_digs)
        mdf.index = pd.RangeIndex(0,mdf[t_col].count(),1)
        mdf = mdf.iloc[:-1] # remove last step (wrong averaged)
    
    m_dt_a = mdf[t_col].diff().mean()
    m_f_a = round((1/m_dt_a),1)
    
    return mdf, m_f, m_f_a

#%% Shifting

[docs]def DetFinSSC(mdf, YM, iS, iLE=None, 
              StressN='Stress', StrainN='Strain', 
              addzero=True, izero=None, option='YM'):
    """
    Determine final stress-strain curve (moved to strain offset from elastic modulus).

    Parameters
    ----------
    mdf : pd.DataFrame
        Original stress-strain curve data.
    YM : dict or pd.Series or float
        Elastic modulus (float) or 
        elastic modulus (key=E) and intersection on strain=0 (key=Eabs) or
        direct input of strain offset (if option is in ['strain_offset','SO','so']).
    iS : index of mdf
        Start of linear behavior (all values befor will dropped).
    iLE : TYPE, optional
        End of linear behavior (if None, strain offset will determined only with iS). 
        The default is None.
    StressN : string, optional
        Column name for stress variable. The default is 'Stress'.
    StrainN : TYPE, optional
        Column name for strain variable. The default is 'Strain'.
    addzero : bool, optional
        Switch to adding Zero value (index,StressN,StrainN=0).
        The default is True.
    izero: index of mdf, optional
        Index for zero value line (p.e. to match old start index)
    option: string, optional
        Option for strain offset. Possible:
            - 'YM': Strain offset determined by elastic modulus.
            - ['strain_offset','SO','so']: Direct input of strain offset.
        The default is 'YM'.

    Returns
    -------
    out : pd.DataFrame
        Moved stress-strain curve data.
    so : float
        Strain offset.

    """
    out=mdf.loc[iS:,[StressN,StrainN]].copy(deep=True)
    if option in ['YM']:
        if isinstance(YM,dict) or pd_isSer(YM):
            so=-YM['E_abs']/YM['E']
        elif isinstance(YM,float):
            if iLE is None:
                lindet=out.iloc[0][[StressN,StrainN]]
            else:
                lindet=out.loc[[iS,iLE],[StressN,StrainN]].mean(axis=0)
            so=lindet[StrainN]-lindet[StressN]/YM
    elif option in ['strain_offset','SO','so']:
        so = YM
    else:
        raise NotImplementedError("Option %s not implemented!"%option)
    out[StrainN]=out[StrainN]-so
    if addzero:
        if izero is None:
            i=0
        else:
            i=izero
        tmp=pd.DataFrame([],columns=out.columns, index=[i])
        tmp[[StressN,StrainN]]=0
        out=pd.concat([tmp,out],axis=0)
    return out, so
```

---

© Copyright 2024, MarcGebhardt.

Built with Sphinx using a
theme
provided by Read the Docs.
