# Supplementary material for: Approach to Standardized Material Characterization of the Human Lumbopelvic System: Testing and Evaluation
Source: Bioengineering (Basel). 2025 Aug 11;12(8):862. doi: 10.3390/bioengineering12080862 (PMC12383908; doi:10.3390/bioengineering12080862)
Supplement: Supplementary file 1 [file bioengineering-12-00862-s001.zip › File S3 Evaluation code/ExMechEva-0.1.2/docs/_build/html/_modules/exmecheva/common/mc_yield.html]

exmecheva.common.mc\_yield — ExMechEva 0.1.0 documentation


ExMechEva

Contents:

- ExMechEva

ExMechEva

- Module code
- exmecheva.common.mc\_yield

---

# Source code for exmecheva.common.mc\_yield

```
# -*- coding: utf-8 -*-
"""
Created on Tue Dec  5 12:36:27 2023

@author: mgebhard
"""
import pandas as pd

from .pd_ext import (pd_limit, pd_valid_index, Find_closest)
from .analyze import (sign_n_change, Line_from2P, Inter_Lines)
from .fitting import (strain_linfit)
from .mc_char import (rise_curve, Diff_Quot)

[docs]def YM_eva_range_refine(m_df, VIP, n_strain, n_stress,
                         n_loBo='S', n_upBo='U',
                         d_loBo=0.05, d_max=0.75, 
                         rise_det=[True,4],
                         n_Outlo='F3',n_Outmi='FM',n_Outhi='F4'):
    """
    Refines the Youngs Modulus determinition range according to 
    "Keuerleber, M. (2006) - Bestimmung des Elastizitätsmoduls von Kunststoffen
    bei hohen Dehnraten am Beispiel von PP. Von der Fakultät Maschinenbau der 
    Universität Stuttgart zur Erlangung der Würde eines Doktor-Ingenieurs (Dr.-Ing.) 
    genehmigte Abhandlung. Doktorarbeit. Universität Stuttgart, Stuttgart"

    Parameters
    ----------
    m_df : pd.DataFrame
        Measured data.
    VIP : pd.Series
        Important points corresponding to measured data.
    n_strain : string
        Name of used strain (have to be in measured data).
    n_stress : string
        Name of used stress (have to be in measured data).
    n_loBo : string
        Lower border for determination (have to be in VIP). The default is 'S'.
    n_upBo : string
        Upper border for determination (have to be in VIP). The default is 'U'.
    d_loBo : float/str, optional
        When float: Percentage of range between n_upBo and n_loBo as start distance to n_loBo.
        When str starting with 'S', followed by integer: Distance in steps to n_loBo.
        The default is 0.05.
    d_max : float, optional
        Percentage of . The default is 0.75.
    rise_det : [bool, int], optional
        Determination options for stress rising ([smoothing, smoothing factor]).
    n_Outlo : string, optional
        Name of new lower border. The default is 'F3'.
    n_Outmi : string, optional
        Name of maximum differential quotient. The default is 'FM'.
    n_Outhi : string, optional
        Name of new upper border. The default is 'F4'.

    Yields
    ------
    VIP_new : pd.Series
        Important points corresponding to measured data.
    txt : string
        Documantation string.
    """
    if isinstance(d_loBo, str):
        if d_loBo.startswith('S'):
            i_loBo = int(d_loBo[1:])
        else:
            raise ValueError("Distance to lower border %s seems to be a string, but doesn't starts with 'S'"%d_loBo)
        Lbord = VIP[n_loBo]+i_loBo
        ttmp = m_df.loc[VIP[n_loBo]+i_loBo:VIP[n_upBo],n_stress]
    else:
        ttmp = m_df.loc[VIP[n_loBo]:VIP[n_upBo],n_stress]
        ftmp=float(ttmp.iloc[0]+(ttmp.iloc[-1]-ttmp.iloc[0])*d_loBo)
        Lbord=abs(ttmp-ftmp).idxmin()

    DQdf=pd.concat(Diff_Quot(m_df.loc[:,n_strain], m_df.loc[:,n_stress],
                              rise_det[0], rise_det[1]), axis=1)
    DQdf=m_df.loc(axis=1)[[n_strain,n_stress]].join(DQdf,how='outer')
    DQdfs=DQdf.loc[Lbord:VIP[n_upBo]]
    
    
    VIP_new = VIP
    txt =""
    VIP_new[n_Outmi]=DQdfs.DQ1.idxmax()
    try:
        VIP_new[n_Outlo]=DQdfs.loc[:VIP_new[n_Outmi]].iloc[::-1].loc[(DQdfs.DQ1/DQdfs.DQ1.max())<d_max].index[0]+1
    except IndexError:
        VIP_new[n_Outlo]=DQdfs.index[0]
        txt+="%s set to start of diff.-quot. determination "%n_Outlo
    try:
        VIP_new[n_Outhi]=DQdfs.loc[VIP_new[n_Outmi]:].loc[(DQdfs.DQ1/DQdfs.DQ1.max())<d_max].index[0]-1
    except IndexError:
        # VIP_new[n_Outhi]=VIP_new[n_Outmi]-1 #-1 könnte zu Problemen führen
        VIP_new[n_Outhi]=VIP_new[n_upBo] #-1 könnte zu Problemen führen
        txt+="%s set on maximum of diff.-quot. "%n_Outhi
    VIP_new=VIP_new.sort_values()
    return VIP_new, DQdfs, txt


[docs]def Yield_redet(m_df, VIP, n_strain, n_stress,
                n_loBo, n_upBo, n_loBo_int, 
                YM, YM_abs, strain_offset=0.002, 
                rise_det=[True,4], n_yield='Y'):
    """
    Redetermine yield point to different conditions 
    (intersection with linearised strain offset (ones after), zero rising, fixed endpoint).

    Parameters
    ----------
    m_df : pd.DataFrame
        Measured data.
    VIP : pd.Series
        Important points corresponding to measured data.
    n_strain : string
        Name of used strain (have to be in measured data).
    n_stress : string
        Name of used stress (have to be in measured data).
    n_loBo : [string]
        List of lower borders for determination (have to be in VIP).
    n_upBo : [string]
        List of upper borders for determination (have to be in VIP).
    n_loBo_int : [string]
        List of lower borders for interseption (have to be in VIP).
    YM : float
        Youngs Modulus.
    YM_abs : float
        Absolut value of Youngs Modulus.
    strain_offset : float, optional
        Strain offset (eq. plastic strain). The default is -0.002.
    rise_det : [bool, int], optional
        Determination options for stress rising ([smoothing, smoothing factor]).
        The default is [True,4].
    n_yield : string, optional
        Name of yield point (have to be in VIP). The default is 'Y'.

    Returns
    -------
    VIP_new : pd.Series
        Important points corresponding to measured data.
    txt : string
        Documantation string.

    """
    #     mit 0.2% Dehnversatz E(F+-F-) finden
    m_lim = m_df.loc[min(VIP[n_loBo]):max(VIP[n_upBo])]
    # stress_fit = stress_linfit(m_lim[n_strain],
    #                            YM, YM_abs, strain_offset)
    # i = m_lim[n_stress]-stress_fit
    # i_sign, i_sich = sign_n_change(i)
    strain_fit = strain_linfit(m_lim[n_stress],
                               YM, YM_abs, strain_offset)
    i = m_lim[n_strain]-strain_fit
    i_sign, i_sich = sign_n_change(i)
    i_sich.iloc[0]=False # First signchange always true
    i_sich = i_sich.loc[min(VIP[n_loBo_int]):] # Y erst ab F- suchen
    
    _,_,r_sich = rise_curve(m_df[n_stress],*rise_det)
    # r_sich = r_sich.loc[min(VIP[n_loBo])+2:max(VIP[n_upBo])+2]
    r_sich = r_sich.loc[min(VIP[n_loBo_int])+2:max(VIP[n_upBo])+2]
    
    VIP_new = VIP
    txt =""
    if i_sign.loc[min(VIP[n_loBo_int])] >= 0:
        VIP_new[n_yield] = min(VIP[n_loBo_int])
        txt+="Fy set on 1st point of lower border of intersection (lower than %.3f %% pl. strain)!"%(strain_offset*100)
    # if (i_sich.any()==True)and(r_sich.any()==True):
    elif (i_sich.any()==True)and(r_sich.any()==True):
        if(i_sich.loc[i_sich].index[0])<=(r_sich.loc[r_sich==True].index[0]):
            VIP_new[n_yield] = i_sich.loc[i_sich].index[0]
            txt+="Fy set on intersection with %.3f %% pl. strain!"%(strain_offset*100)
        else:
            VIP_new[n_yield]=r_sich.loc[r_sich==True].index[0]-2 #-2 wegen Anstiegsberechnung
            txt+="Fy on first point between F+ and Fu with rise of 0, instead intersection %.3f %% pl. strain! (earlier)"%(strain_offset*100)
    elif (i_sich.any()==True)and(r_sich.all()==False):
        VIP_new[n_yield]=i_sich.loc[i_sich].index[0]
        txt+="Fy set on intersection with %.3f %% pl. strain!"%(strain_offset*100)
    else:
        if r_sich.any():
            VIP_new[n_yield]=r_sich.loc[r_sich==True].index[0]-2
            txt+="Fy on first point between F+ and Fu with rise of 0, instead intersection %.3f %% pl. strain! (No intersection found!)"%(strain_offset*100)
        else:
            tmp = VIP[VIP==max(VIP[n_upBo])].index[0]
            VIP_new[n_yield] = VIP[tmp]
            txt+="Fy set to %s (max of %s), instead intersection %.3f %% pl. strain or rise of 0! (No intersection found!)"%(tmp,n_upBo,(strain_offset*100))
    VIP_new = VIP_new.sort_values()
    return VIP_new, txt


[docs]def Find_intg2p(gsl, gin, pdo, i1=None, i2=None, 
                    x='Strain', y='Stress', so=0, n_yield='Y'):
    """Determine intersection of line and point (usefull to find yield point)"""
    pdn = pd_limit(pdo, i1, i2)[[x,y]]
    psl, pin = Line_from2P(pdn.iloc[0].values,pdn.iloc[-1].values)
    gso=pd.DataFrame([0,1], columns=[y])
    gso[x]=gso[y].apply(lambda x: strain_linfit(x, gsl, gin, so))
    gso=gso[[x,y]]
    gslso, ginso = Line_from2P(gso.iloc[0].values,gso.iloc[-1].values)
    inter = Inter_Lines(r1=gslso, c1=ginso, r2=psl, c2=pin, out='xy')
    i = pdn.iloc[0].name + (pdn.iloc[-1].name-pdn.iloc[0].name)*(inter[1]-pdn.iloc[0][y])/(pdn.iloc[-1][y]-pdn.iloc[0][y])
    inter = pd.Series([i,*inter], index=['ind_ex',x,y], name=n_yield)
    return inter


[docs]def Yield_redet2(m_df, VIP, n_strain, n_stress,
                n_loBo, n_upBo, n_loBo_int,
                YM, YM_abs, strain_offset=0.002,
                use_rd =True, rise_det=[True,4],
                n_yield='Y', ywhere='n'):
    """
    Redetermine yield point to different conditions 
    (intersection with linearised strain offset (ones after), zero rising, fixed endpoint).

    Parameters
    ----------
    m_df : pd.DataFrame
        Measured data.
    VIP : pd.Series
        Important points corresponding to measured data.
    n_strain : string
        Name of used strain (have to be in measured data).
    n_stress : string
        Name of used stress (have to be in measured data).
    n_loBo : [string]
        List of lower borders for determination (have to be in VIP).
    n_upBo : [string]
        List of upper borders for determination (have to be in VIP).
    n_loBo_int : [string]
        List of lower borders for interseption (have to be in VIP).
    YM : float
        Youngs Modulus.
    YM_abs : float
        Absolut value of Youngs Modulus.
    strain_offset : float, optional
        Strain offset (eq. plastic strain). The default is -0.002.
    use_rd : bool, optional
        Switch for using change in stress rising. The default is True.
    rise_det : [bool, int], optional
        Determination options for stress rising ([smoothing, smoothing factor]).
        The default is [True,4].
    n_yield : string, optional
        Name of yield point (have to be in VIP). The default is 'Y'.
    ywhere : string, optional
        Which point should be choosen (n-next, a-after, b-before). The default is 'n'.

    Returns
    -------
    VIP_new : pd.Series
        Important points corresponding to measured data.
    txt : string
        Documantation string.

    """
    # Intersection with linearization and strain offset
    m_lim = m_df.loc[min(VIP[n_loBo]):max(VIP[n_upBo])]
    strain_fit = strain_linfit(m_lim[n_stress],
                               YM, YM_abs, strain_offset)
    i = m_lim[n_strain]-strain_fit
    i_sign, i_sich = sign_n_change(i)
    i_sich.iloc[0]=False # First signchange always true

    # find change in rise of stress
    if use_rd:
        _,_,r_sich = rise_curve(m_df[n_stress],*rise_det)
        r_sich = r_sich.loc[min(VIP[n_loBo_int])+2:max(VIP[n_upBo])+2]
        if r_sich.any()==True:
            i_r_sich_1st=r_sich.loc[r_sich==True].index[0]-2 # -2  due to determination
        else:
            i_r_sich_1st=max(VIP[n_upBo]) # no signchange, last point
    else:
        i_r_sich_1st=max(VIP[n_upBo]) # no signchange, last point

    # Intersection only from determination limit and to maximum/last sign change
    i_sign = i_sign.loc[min(VIP[n_loBo_int]):i_r_sich_1st]
    i_sich = i_sich.loc[min(VIP[n_loBo_int]):i_r_sich_1st]

    VIP_new = VIP
    plst_txt="%.3f %% plastic strain"%(strain_offset*100)
    txt ="%s "%(n_yield)
    if i_sich.any():
        iy=i_sich.loc[i_sich].index[-1] # Signchange allways after 'a'
        yexser=Find_intg2p(gsl=YM, gin=YM_abs, pdo=m_lim, 
                           i1=pd_valid_index(iy, i, opt='b'), i2=iy, 
                           x=n_strain, y=n_stress, so=strain_offset,n_yield=n_yield)
        if ywhere=='b':
            iy=pd_valid_index(iy, i, opt='b')
        elif ywhere=='n':
            iy=Find_closest(i.loc[[pd_valid_index(iy, i, opt='b'),iy]],0)
        txt+="set on intersection with %s (option=%s)"%(plst_txt,ywhere)
    else:
        if (i_sign <= 0).all():
            iy=i_r_sich_1st
            if i_r_sich_1st==max(VIP[n_upBo]):
                tmp = VIP[VIP==max(VIP[n_upBo])].index[0]
                txt+="set to %s (max of %s), instead intersection %s or rise of 0! (No intersection found!)"%(tmp,n_upBo,plst_txt)
            else:
                txt+="on first point between F+ and Fu with rise of 0, instead intersection %s! (No intersection found!)"%(plst_txt)
        else:
            iy=min(VIP[n_loBo_int])
            tmp = VIP[VIP==min(VIP[n_loBo_int])].index[0]
            txt+="set to %s (min of %s), instead intersection %s or rise of 0! (No intersection found!)"%(tmp,n_loBo_int,plst_txt)
        yexser = pd.Series([iy,*m_lim.loc[iy][[n_strain,n_stress]].values], 
                           index=['ind_ex',n_strain,n_stress], name=n_yield)
    VIP_new[n_yield] = iy
    VIP_new = VIP_new.sort_values()
    return VIP_new, txt, yexser


[docs]def Yield_redet2_Multi(m_df, VIP, YM, YM_abs,
                   strain_osd={'YK':0.0,'Y0':0.0,'Y':0.2/100,'Y1':0.007/100}, 
                   strain_osdf={'YK':'F4'},
                   n_strain='Strain', n_stress='Stress',
                   n_loBo=['F3'], n_upBo=['U'], n_loBo_int=['F3'],
                   use_rd =True, rise_det=[True,2], 
                   ywhere='n'):
    txt=''
    y_exact=pd.DataFrame(pd.Series(strain_osd), columns=['strain_os'])
    for i in strain_osd.keys():
        if i in strain_osdf.keys():
            VIP[i] = VIP[strain_osdf[i]]
            txt+="\n %s set on %s (fixed)"%(i, strain_osdf[i])
            tmp=VIP[i],VIP[i],*m_df.loc[VIP[i],[n_strain,n_stress]].values
            y_exact.loc[i, ['ind','ind_ex',n_strain,n_stress]] = tmp
        else:
            tmp = Yield_redet2(m_df=m_df, VIP=VIP,
                                n_strain=n_strain, n_stress=n_stress,
                                n_loBo=n_loBo, n_upBo=n_upBo, n_loBo_int=n_loBo_int,
                                YM     = YM, YM_abs = YM_abs,
                                strain_offset=strain_osd[i],
                                use_rd =use_rd, rise_det=rise_det, 
                                n_yield=i, ywhere=ywhere)
            VIP = tmp[0]
            txt += '\n '+ tmp[1]
            y_exact.loc[i, ['ind','ind_ex',n_strain,n_stress]] = VIP[i],*tmp[2].values
    return VIP, y_exact, txt
```

---

© Copyright 2024, MarcGebhardt.

Built with Sphinx using a
theme
provided by Read the Docs.
