# Supplementary material for: Approach to Standardized Material Characterization of the Human Lumbopelvic System: Testing and Evaluation
Source: Bioengineering (Basel). 2025 Aug 11;12(8):862. doi: 10.3390/bioengineering12080862 (PMC12383908; doi:10.3390/bioengineering12080862)
Supplement: Supplementary file 1 [file bioengineering-12-00862-s001.zip › File S3 Evaluation code/ExMechEva-0.1.2/docs/_build/html/_modules/exmecheva/common/output.html]

exmecheva.common.output — ExMechEva 0.1.0 documentation


ExMechEva

Contents:

- ExMechEva

ExMechEva

- Module code
- exmecheva.common.output

---

# Source code for exmecheva.common.output

```
# -*- coding: utf-8 -*-
"""
Functions for output data generation.

@author: MarcGebhardt
"""
import numpy as np
import pandas as pd

from .pd_ext import pd_trapz

#%% logging

[docs]def str_indent(po, indent=3):
    """
    Adds indentation to str at each new line.

    Parameters
    ----------
    po : string or str callable
        String to manipulate.
    indent : int, optional
        Number of spaces for indentation. The default is 3.

    Returns
    -------
    poo : string
        Output string with indentation.

    """
    # if isinstance('s',str):
    #     pos = po
    # elif isinstance(po, pd.core.base.ABCSeries):
    #     pos = po.to_string()
    # elif isinstance(po, pd.core.base.ABCDataFrame):
    #     pos = po.to_string()
    # else:
    #     raise NotImplementedError("Type %s not implemented!"%type(po))
    pos = str(po)
    if not pos.startswith("\n"):
        pos = indent*" " + pos
        # pos = "\n" + pos
    poo = ("\n"+pos.replace("\n","\n"+indent*" "))
    # poo = (pos.replace("\n","\n"+indent*" "))
    return poo


[docs]def str_log(s, logfp, output_lvl = 1, logopt=True, printopt=True):
    """
    Write lines to log and/or console.
    TODO: add functionality for different backends! (Console/window object)

    Parameters
    ----------
    s : string
        String to write or print.
    logfp : string or path
        Open log file.
    output_lvl : positiv integer
        Output level (0=none, 1=only text, 2=additional diagramms).
        The default is 1.
    logopt : bool
        Wriet in log file (logfp). The default is True.
    printopt : bool
        Display on terminal. The default is True.

    """
    if output_lvl>=1:
        if logopt and not(logfp is None):
            logfp.write(s)
        if printopt:
            print(s, end='')

#%% output values

[docs]def Outvalgetter(mdf, V, VIP, exacts=None, 
                 order=['f','e','U'], add_esuf='_con',
                 n_strain='Strain', n_stress='Stress',
                 use_exacts=True):
    """
    Get output values from given data and options.

    Parameters
    ----------
    mdf : pd.DataFrame
        DESCRIPTION.
    V : string
        Relevant VIP (have to be in VIP).
    VIP : list of string
        'Very important point'.
    exacts : pd.DataFrame, optional
        Exact values (p.e. for yield point). 
        Need to have special format (see common.analyze.Yield_redet2_Multi):
            - index: VIP
            - columns: ['strain_os','ind','ind_ex',n_strain,n_stress]
        The default is None.
    order : list of string, optional
        Output order. Implemented are:
            - 'f': stress (no suffix)
            - 'F': force (no suffix)
            - 'e': strain
            - 's': displacement
            - 'U': strain energy / -density
            - 'W': deformation energy
        The default is ['f','e','U'].
    add_esuf : string, optional
        Suffix for strain/displacement relation acc. to measurement method.
        The default is '_con'.
    n_strain : string, optional
        Column name in mdf for strain values. The default is 'Strain'.
    n_stress : string, optional
        Column name in mdf for stress values. The default is 'Stress'.
    use_exacts : bool, optional
        Switch to use exact values if applicable. The default is True.

    Returns
    -------
    out : pd.Series
        Evaluated values.

    """
    if exacts is None: use_exacts=False
    out=pd.Series([], dtype='float64')
    for i in order:
        if i in ['f','F']:
            name = i+V.lower()
            if use_exacts and (V in exacts.index):
                out[name]=exacts.loc[V,n_stress]
            elif (V in VIP.index):
                out[name]=mdf.loc[VIP[V],n_stress]
            else:
                out[name]=np.nan
        elif i in ['e','s']:
            name = i+V.lower()+add_esuf
            if use_exacts and (V in exacts.index):
                out[name]=exacts.loc[V,n_strain]
            elif (V in VIP.index):
                out[name]=mdf.loc[VIP[V],n_strain]
            else:
                out[name]=np.nan
        elif i in ['U','W']:
            name = i+V.lower()+add_esuf
            if use_exacts and (V in exacts.index):
                tmp = exacts.loc[V,[n_strain,n_stress]]
                tmp.name = exacts.loc[V,'ind_ex']
                tmp = mdf.append(tmp).sort_index()
                out[name]=pd_trapz(tmp.loc[:exacts.loc[V,'ind_ex']],
                                        y=n_stress, x=n_strain,
                                        axis=0, nan_policy='omit')
            elif (V in VIP.index):
                out[name]=pd_trapz(mdf.loc[:VIP[V]],
                                        y=n_stress, x=n_strain,
                                        axis=0, nan_policy='omit')
            else:
                out[name]=np.nan
    return out


[docs]def Otvalgetter_Multi(mdf, Vs=['Y','YK','Y0','Y1','U','B'],
                      datasep=['con','opt'], VIPs={'con':None,'opt':None}, 
                      exacts={'con':None,'opt':None}, 
                      orders={'con':['f','e','U'],'opt':['e','U']}, 
                      add_esufs={'con':'_con','opt':'_opt'},
                      n_strains={'con':'Strain','opt':'DStrain'}, 
                      n_stresss={'con':'Stress','opt':'Stress'},
                      use_exacts=True):
    """
    Wrapper for Outvalgetter.

    Parameters
    ----------
    mdf : TYPE
        DESCRIPTION.
    Vs : list of strings, optional
        Relevant VIPs (have to be in VIP). 
        The default is ['Y','YK','Y0','Y1','U','B'].
    datasep : list of strings, optional
        Data seperation by strain/displacement relation. 
        The default is ['con','opt'].
    VIPs : dict, optional
        'Very important points'. The default is {'con':None,'opt':None}.
    exacts : dict of pd.DataFrame, optional
        Exact values (p.e. for yield point) seperated by strain/displacement relation. 
        Need to have special format (see common.analyze.Yield_redet2_Multi):
            - index: VIP
            - columns: ['strain_os','ind','ind_ex',n_strain,n_stress]
        The default is None.
    orders : dict of lists of strings, optional
        Output order seperated to datasep. Implemented are:
            - 'f': stress
            - 'F': force
            - 'e': strain
            - 's': displacement
            - 'U': strain energy / -density
            - 'W': deformation energy
        The default is {'con':['f','e','U'],'opt':['e','U']}.
    add_esufs : dict of strings, optional
        Suffixes for strain/displacement relation acc. to measurement method.
        The default is {'con':'_con','opt':'_opt'}.
    n_strains : dict of strings, optional
        Column name in mdf for strain values.
        The default is {'con':'Strain','opt':'DStrain'}.
    n_stresss : TYPE, optional
        Column name in mdf for stress values.
        The default is {'con':'Stress','opt':'Stress'}.
    use_exacts : bool, optional
        Switch to use exact values if applicable. The default is True.

    Returns
    -------
    out : pd.Series
        Evaluated values.
        
    Examples
    --------
    Otvalgetter_Multi(messu_FP, Vs=relVS,
                      datasep=['con','opt'], 
                      VIPs={'con':VIP_messu,'opt':VIP_dicu}, 
                      exacts={'con':yield_df_con,'opt':yield_df_opt}, 
                      orders={'con':['f','e','U'],'opt':['e','U']}, 
                      add_esufs={'con':'_con','opt':'_opt'},
                      n_strains={'con':'Strain','opt':dic_used_Strain}, 
                      n_stresss={'con':'Stress','opt':'Stress'},
                      use_exacts=True)

    """
    out = pd.Series([],dtype='float64')
    for V in Vs:
        for ds in datasep:
            out=out.append(Outvalgetter(mdf=mdf, V=V, VIP=VIPs[ds], exacts=exacts[ds], 
                                    order=orders[ds], add_esuf=add_esufs[ds],
                                    n_strain=n_strains[ds], n_stress=n_stresss[ds],
                                    use_exacts=use_exacts))
    return out
```

---

© Copyright 2024, MarcGebhardt.

Built with Sphinx using a
theme
provided by Read the Docs.
