# Supplementary material for: Approach to Standardized Material Characterization of the Human Lumbopelvic System: Testing and Evaluation
Source: Bioengineering (Basel). 2025 Aug 11;12(8):862. doi: 10.3390/bioengineering12080862 (PMC12383908; doi:10.3390/bioengineering12080862)
Supplement: Supplementary file 1 [file bioengineering-12-00862-s001.zip › File S3 Evaluation code/ExMechEva-0.1.2/docs/_build/html/_modules/exmecheva/common/pd_ext.html]

exmecheva.common.pd\_ext — ExMechEva 0.1.0 documentation


ExMechEva

Contents:

- ExMechEva

ExMechEva

- Module code
- exmecheva.common.pd\_ext

---

# Source code for exmecheva.common.pd\_ext

```
# -*- coding: utf-8 -*-
"""
Contains extensions to pandas functionality (mostly indexing and NaN-handling).

@author: MarcGebhardt

ToDo:
    - Overwrite implementation needed?
"""
import numpy as np
import pandas as pd

from .analyze import (sign_n_change, normalize)

#%% tests

[docs]def pd_isDF(pdo):
    """Tests if object is instance of pandas dataframe"""
    return isinstance(pdo, pd.core.base.ABCDataFrame)


[docs]def pd_isSer(pdo):
    """Tests if  object is instance of pandas series"""
    return isinstance(pdo, pd.core.base.ABCSeries)

#%% indexing

[docs]def pd_limit(self, iS=None, iE=None):
    """
    Limit a pandas Series or Dataframe to given indexes

    Parameters
    ----------
    iS : matching type of self.index, optional
        Start index. The default is None.
    iE : matching type of self.index, optional
        End index. The default is None.

    Returns
    -------
    pandas.Series or pandas.DataFrame
        Limited pandas object.

    """
    if not (iS is None):
        self = self.loc[iS:]
    if not (iE is None):
        self = self.loc[:iE]
    return self

# Overwrite pandas
pd.Series.indlim = pd_limit
pd.DataFrame.indlim = pd_limit

[docs]def pd_slice_index(index, vals, option='range'):
    """
    Slice an index by given values and option.

    Parameters
    ----------
    index : pd.Index
        Index.
    vals : range or list
        Slicing values (list or, lower and upper limits).
    option : string, optional
        Option for slicing (range or list). The default is 'range'.

    Raises
    ------
    NotImplementedError
        Option not implemented.

    Returns
    -------
    ind_new : pd.Index
        New index.

    """
    if option=='range':
        ind=[]
        ind.append(index.searchsorted(vals[0], side='left'))
        ind.append(index.searchsorted(vals[-1], side='right'))
        ind_new=index[ind[0]:ind[-1]]
    elif option=='list':
        ind_new=index[index.searchsorted(vals)]
    else:
        raise NotImplementedError("option %s not implemented!"%option)
    return ind_new


[docs]def pd_combine_index(pd1, pd2, option='dropna'):
    """Returns a combined index of two pandas objects (series or dataframe) 
    with valide values in both."""
    if option=='dropna':
        if isinstance(pd1,pd.core.indexes.base.Index):
            index1=pd1
        else:
            index1=pd1.dropna().index
        if isinstance(pd2,pd.core.indexes.base.Index):
            index2=pd2
        else:
            index2=pd2.dropna().index
    elif option=='fl_valid':
        if isinstance(pd1,pd.core.indexes.base.Index):
            index1=pd1
        else:
            f1 = pd1.first_valid_index()
            l1 = pd1.last_valid_index()
            index1=pd1.loc[f1:l1].index
        if isinstance(pd2,pd.core.indexes.base.Index):
            index2=pd2
        else:
            f2 = pd2.first_valid_index()
            l2 = pd2.last_valid_index()
            index2=pd2.loc[f2:l2].index
    else:
        raise NotImplementedError("option %s not implemented!"%option)
    ind_new = index1.join(index2, how='inner')
    return ind_new


[docs]def pd_valid_index(val, pdo, opt='ba', na_hand='Value', na_out=None, add_ind=None):
    """Returning valid index of pandas object in respect to defined option.
    (b=before,a=after,n=nearest,ba=before and after,bna=before, nearest and after)
    Filling not available values according a handler (na_hand) with 
    standard value (na_out) or a part of an additional index (add_ind)."""
    if isinstance(pdo,pd.core.indexes.base.Index):
        index=pdo
    elif (type(pdo) is pd.core.frame.DataFrame) or (type(pdo) is pd.core.series.Series):
        index=pdo.index
    elif isinstance(pdo,list):
        index=pd.Index(pdo)
    else:
        raise TypeError('Unexpected type of input (%s)'%type(pdo))
    
    def pd_valid_index_na(na_hand, na_out, pos, add_ind):
        """Not available handler for function pd_valid_index."""
        if na_hand=='Value':
            na_ret=na_out
        elif na_hand=='fi_la_add':
            if pos=='b':
                na_ret=add_ind[0]
            elif pos=='a':
                na_ret=add_ind[-1]
            elif pos=='n':
                na_ret=na_out
            else:
                raise ValueError("Position (%s) not valid!"%pos)
        else:
            raise NotImplementedError("NA-Handler (%s) not implemented!"%na_hand)
        return na_ret            
    
    try:
        ob=index[index.get_indexer_for([val])[0]-1]
    except IndexError:
        # ob=nf_out
        ob=pd_valid_index_na(na_hand=na_hand, na_out=na_out, 
                             pos='b', add_ind=add_ind)
    if ob == index[-1]: #No jump from first to last position
        # ob=nf_out
        ob=pd_valid_index_na(na_hand=na_hand, na_out=na_out, 
                             pos='b', add_ind=add_ind)
    try:
        oa=index[index.get_indexer_for([val])[0]+1]
    except IndexError:
        # oa=nf_out
        oa=pd_valid_index_na(na_hand=na_hand, na_out=na_out, 
                             pos='a', add_ind=add_ind)
    try:
        on=index[index.get_indexer_for([val])[0]]
    except IndexError:
        # on=nf_out
        on=pd_valid_index_na(na_hand=na_hand, na_out=na_out, 
                             pos='n', add_ind=add_ind)
        
    # out=nf_out
    if opt=='b':
        out=ob
    elif opt=='a':
        out=oa
    elif opt=='n':
        out=on
    elif opt=='ba':
        out=[ob,oa]
    elif opt=='bna':
        out=[ob,on,oa]
    else:
        raise NotImplementedError("Option %s not implemented!"%opt)
    return out


[docs]def pd_find_index(ser, s):
    if not isinstance(s, list):
        s=list(s)
    o = ser.loc[ser.Type.apply(lambda x: x in s)].index
    return o


[docs]def deal_dupl_index(df,deal_dupl_ind='raise'):
    """
    Deal with dupliceted index values according option.

    Parameters
    ----------
    df : pandas.Series or pandas.DataFrame
        Data input.
    deal_dupl_ind : string, optional
        Option to deal with duplicated index entries. 
        Possible are:
            - 'raise': raises IndexError when finding duplicates.
            - 'keep': keep unchanged and give no error.
            - 'keep-first': drop all duplicated entries except first one.
            - 'keep-last': drop all duplicated entries except first one.
            - Aggratable function or string accapted by pd.agg: Aggregate duplicated index entries by function.
        The default is 'raise'.

    Raises
    ------
    IndexError
        Index has duplicates and option set to 'raise'.
    ValueError
        option (deal_dupl_ind) is not aggregatable.

    Returns
    -------
    dft : pandas.Series or pandas.DataFrame
        Data output.

    """
    dft = df.copy(deep=True)
    tmp=dft.index.duplicated(keep=False)
    if tmp.any() and deal_dupl_ind!='keep':
        if deal_dupl_ind=='raise':
                raise IndexError('Duplicated index found: \n%s'%dft.loc[tmp])
        elif deal_dupl_ind=='drop':
            tmp=dft.index.duplicated(keep=False)
            dft=dft.loc[~tmp]
        elif deal_dupl_ind=='keep-first':
            tmp=dft.index.duplicated(keep='first')
            dft=dft.loc[~tmp]
        elif deal_dupl_ind=='keep-last':
            tmp=dft.index.duplicated(keep='last')
            dft=dft.loc[~tmp]
        else:
            try:
                tmp=list(range(dft.index.nlevels))
                dft=dft.groupby(level=tmp).agg(deal_dupl_ind)
            except:
                raise ValueError('Operation %s not aggregatable!'%deal_dupl_ind)
    return dft


[docs]def Find_closest(pds, val, iS=None, iE=None, option='abs'):
    """Returns the index of the closest value of a Series to a value"""
    if not ((iS is None) and (iE is None)):
        pds = pds.indlim(iS=iS,iE=iE)
    if option == 'abs':
        pdt = abs(pds-val)
    elif option == 'lower':
        pdt = abs(pds.where(pds < val)-val)
    elif option == 'higher':
        pdt = abs(pds.where(pds > val)-val)
    i=pdt.idxmin()
    return i


[docs]def Find_closest_perc(pds, p, iS=None, iE=None, 
                      range_sub='min', norm='max', option='abs'):
    """Returns the index of the closest value of a Series 
    to a percentage value in a range of this series"""
    if not ((iS is None) and (iE is None)):
        pds = pds.indlim(iS=iS,iE=iE)
    if range_sub == 'min':
        pds = pds-pds.min() 
    elif range_sub == 'max':
        pds = pds-pds.max()
    elif range_sub == 'iS':
        pds = pds-pds.loc[iS]
    elif range_sub == 'iE':
        pds = pds-pds.loc[iE]
    pdn = normalize(pdo=pds, norm=norm)
    if isinstance(p,float):
        i=Find_closest(pds=pdn, val=p, option=option)
    elif isinstance(p,list) or isinstance(p,np.ndarray) or isinstance(p, pd.core.base.ABCSeries):
        i=[]
        for t in p:
            i.append(Find_closest(pds=pdn, val=t, option=option))
    else:
        raise TypeError("Type %s not implemented!"%type(p))
    return i


[docs]def Find_closestv(pds1, pds2, val1, val2, iS=None, iE=None, option='quad'):
    """Returns the index of the closest value of two corresponding Series
    to a pair of values"""
    if not ((iS is None) and (iE is None)):
        pds1 = pds1.indlim(iS=iS,iE=iE)
        pds2 = pds2.indlim(iS=iS,iE=iE)
    if option == 'abs':
        pdt = abs(pds1-val1)+abs(pds2-val2)
    # elif option == 'quad': # 221014 - option größer/kleiner val1
    elif option in ['quad','quad_hieq','quad_loeq',
                    'quad_1hieq','quad_1loeq','quad_2hieq','quad_2loeq']:
        pdt = ((pds1-val1)**2+abs(pds2-val2)**2)**0.5
        if option == 'quad_1hieq':
            pdt = pdt.where(pds1 >= val1)
        elif option == 'quad_1loeq':
            pdt = pdt.where(pds1 <= val1)
        elif option == 'quad_2hieq':
            pdt = pdt.where(pds2 >= val2)
        elif option == 'quad_2loeq':
            pdt = pdt.where(pds2 <= val2)
    i=pdt.idxmin()
    return i


[docs]def Find_first_sc(pds, val, iS=None, iE=None, 
                  direction='normal', 
                  option='after', exclude_1st=True,
                  nan_policy='omit'):
    """Returns the index of the first value of a Series with change in sign to a value"""
    if not ((iS is None) and (iE is None)):
        pdt = pds.indlim(iS=iS,iE=iE).copy(deep=True)
    else:
        pdt=pds.copy(deep=True)
    pdt=pd_nan_handler(pdt, nan_policy=nan_policy)
    if direction=='reverse':
        pdt=pdt.iloc[::-1]
        
    tmp=sign_n_change(pdt-val)[1]
    if exclude_1st:
        tmp=tmp.iloc[1:] #first index always==True?!
    if option in ['before','b','bf']:
        i=tmp[tmp].index[0]
        # if direction=='reverse':
        #     i=pd_valid_index(i+1, tmp, opt='b')
        # else:
        #     i=pd_valid_index(i-1, tmp, opt='b')
        i=pd_valid_index(i-1, tmp, opt='b')
    elif option in ['after','a','af']:
        i=tmp[tmp].index[0] 
    return i

#%% NaN handling

[docs]def pd_exclnan(pdo,axis=1):
    if pd_isDF(pdo):
        exm=pdo.isna().any(axis=axis)
    elif pd_isSer(pdo):
        exm=pdo.isna()
    else:
        NotImplementedError("Type %s not implemented!"%type(pdo))
    return pdo.loc[~exm]


[docs]def pd_nan_handler(pdo, ind=None, axis=0, nan_policy='omit'):
    """
    Handles NaN in pandas object according to NaN-policy.

    Parameters
    ----------
    pdo : pandas.Series or pandas.DataFrame
        Input Value.
    ind : (same as corresponding axis), optional
        Index limitation on not scanned axis. The default is None.
    axis : [0,1]/["index","columns"], optional
        Scanned axis. The default is 0.
    nan_policy : string, optional
        Policy to handle NaN. Implemented: omit, raise, interpolate. 
        The default is 'omit'.

    Raises
    ------
    ValueError
        Raise error if nan_policy is raise and NaN's detected.

    Returns
    -------
    pds : pandas.Series or pandas.DataFrame
        Output value with NaN handled acc. NaN-policy.

    """
    pds=pdo.copy(deep=True)
    if pd_isDF(pds):
        if not ind is None: pds=pds.loc(axis=pd_axischange(axis))[ind]
        if nan_policy=='omit':
            pds=pd_exclnan(pdo=pds, axis=pd_axischange(axis))
        elif nan_policy=='raise' and pds.isna().any(None):
            raise ValueError("NaN in objectiv and NaN-policy = %s!"%nan_policy)
        elif nan_policy=='interpolate':
            pds=pds.interpolate(axis=axis)
    elif pd_isSer(pds):
        if nan_policy=='omit':
            pds=pd_exclnan(pdo=pds)
        elif nan_policy=='raise' and pds.isna().any(None):
            raise ValueError("NaN in objectiv and NaN-policy = %s!"%nan_policy)
        elif nan_policy=='interpolate':
            pds=pds.interpolate()
    else:
        NotImplementedError("Type %s not implemented!"%type(pds))
    return pds

#%% misc

[docs]def pd_outsort(data, outsort='ascending'):
    """
    Sorts pandas object by given string.

    Parameters
    ----------
    data : pd.Series or pd.DataFrame
        Data to be sorted.
    outsort : str, optional
        Option for sorting. Implemented:
        - ascending: ['A','a','ascending',True,'r','rising','rise']
        - descending: ['D','d','descending','f','falling','fall']
        The default is 'ascending'.

    Returns
    -------
    dout : pd.Series or pd.DataFrame
        Sortted data.

    """
    if outsort in ['A','a','ascending',True,'r','rising','rise']:
        dout = data.sort_values(ascending=True)
    elif outsort in ['D','d','descending','f','falling','fall']:
        dout = data.sort_values(ascending=False)
    else:
        dout = data
    return dout


[docs]def pd_axischange(axis):
    """
    Change axis (use for pandas object). Index will return columns, 
    0 will return 1 and in visa versa.

    Parameters
    ----------
    axis : int in [0,1] or string in [index,columns]
        Axis determiner.

    Returns
    -------
    a : int or string
        Oposite axis of axis.

    """
    if axis==0:
        a=1
    elif axis==1:
        a=0
    if axis=="columns":
        a="index"
    elif axis=="index":
        a="columns"
    else:
        NotImplementedError("Axis %s not implemented!"%axis)
    return a


[docs]def pd_vec_length(pdo, norm=False, norm_kws={}, out='Series'):
    """Calculate (normalized) vector length and return ether whole series or index"""
    if norm:
        pds=normalize(pdo, **norm_kws)
    else:
        pds=pdo
    pds=((pds**2).sum(axis=1))**0.5
    if out=='Series':
        out=pds
    else:
        out=pds.idxmax()
    return out


[docs]def pd_trapz(pdo, y=None, x=None, axis=0, nan_policy='omit'):
    """
    Extends usage of np.trapz for pandas with nan policy.

    Parameters
    ----------
    pdo : pd.DataFrame or pd.Series
        Pandas object containing data.
    y : string, optional
        Determiner for y-values. Have to be in index or columns (see axis).
        The default is None.
    x : string, optional
        Determiner for x-values. Have to be in index or columns (see axis).
        The default is None.
    axis : int in [0,1] or string in [index,columns], optional
        Axis determiner to perform action. The default is 0.
    nan_policy : string, optional
        Option for handling NaN-values. The default is 'omit'.

    Raises
    ------
    ValueError
        NaN handling.
    NotImplementedError
        Option for nan_policy not implemented.

    Returns
    -------
    out : float or series of float
        DESCRIPTION.

    """
    if pd_isDF(pdo):
        pdo=pdo.loc(axis=pd_axischange(axis))[[x,y]]
        if nan_policy=='omit':
            pdo=pd_exclnan(pdo=pdo, axis=pd_axischange(axis))
        elif nan_policy=='raise' and pdo.isna().any(None):
            raise ValueError("NaN in objectiv!")
        elif nan_policy=='interpolate':
            pdo=pdo.interpolate(axis=axis)
        out=np.trapz(y=pdo.loc(axis=pd_axischange(axis))[y],
                     x=pdo.loc(axis=pd_axischange(axis))[x])
    elif pd_isSer(pdo):
        if nan_policy=='omit':
            pdo=pd_exclnan(pdo=pdo)
        elif nan_policy=='raise' and pdo.isna().any(None):
            raise ValueError("NaN in objectiv!")
        elif nan_policy=='interpolate':
            pdo=pdo.interpolate()
        out=np.trapz(y=pdo)
    else:
        NotImplementedError("Type %s not implemented!"%type(pdo))
    return out
```

---

© Copyright 2024, MarcGebhardt.

Built with Sphinx using a
theme
provided by Read the Docs.
