# Supplementary material for: Approach to Standardized Material Characterization of the Human Lumbopelvic System: Testing and Evaluation
Source: Bioengineering (Basel). 2025 Aug 11;12(8):862. doi: 10.3390/bioengineering12080862 (PMC12383908; doi:10.3390/bioengineering12080862)
Supplement: Supplementary file 1 [file bioengineering-12-00862-s001.zip › File S3 Evaluation code/ExMechEva-0.1.2/docs/_build/html/_modules/exmecheva/common/plotting.html]

exmecheva.common.plotting — ExMechEva v0.1.1 documentation


ExMechEva

Contents:

- ExMechEva

ExMechEva

- Module code
- exmecheva.common.plotting

---

# Source code for exmecheva.common.plotting

```
# -*- coding: utf-8 -*-
"""
Plotting functionality.

@author: MarcGebhardt
"""
import numpy as np
import pandas as pd
import matplotlib.pyplot as plt
import seaborn as sns

from .analyze import (normalize, threshhold_setter)
from .fitting import regfitret_restring_func

#%% core

[docs]def plt_handle_suffix(fig, path='foo', tight=True, show=True, 
                      save=True, s_types=["pdf","png"], 
                      clear=True, close=True):
    """
    Handler for end of plotting procedure.

    Parameters
    ----------
    fig : matplotlib.pyplot.figure
        Figure instance.
    path : str, optional
        Save path. The default is 'foo'.
    tight : bool, optional
        Tight the layout. The default is True.
    show : bool, optional
        Show figure. The default is True.
    save : bool, optional
        Save figure. The default is True.
    s_types : list of str, optional
        Types in which the figure is saved. The default is ["pdf","png"].
    clear : bool, optional
        Clear figure instance. The default is True.
    close : bool, optional
        Close figure window. The default is True.

    Returns
    -------
    None.

    """
    if tight: fig.tight_layout()  # otherwise the right y-label is slightly clipped
    if show:  plt.show()
    if save and not (path is None):
        for st in s_types:
            # plt.savefig(path+'.'+st)
            fig.savefig(path+'.'+st)
    if clear: fig.clf()
    if close: plt.close(fig)


[docs]def tick_label_renamer(ax, renamer={}, axis='both'):
    """Renames labels on axis (can be switched ['both','x','y'])."""
    def get_ren_label(ax, renamer, axis):
        if axis=='x':
            label = ax.get_xticklabels()
        elif axis=='y':
            label = ax.get_yticklabels()
        else:
            raise NotImplementedError('Axis %s not implemented!'%axis)
        label = pd.Series([item.get_text() for item in label])
        label = label.replace(renamer).values
        if axis=='x':
            label = ax.set_xticklabels(label)
        elif axis=='y':
            label = ax.set_yticklabels(label)
        else:
            raise NotImplementedError('Axis %s not implemented!'%axis)
    if axis=='both':
        get_ren_label(ax=ax, renamer=renamer, axis='x')
        get_ren_label(ax=ax, renamer=renamer, axis='y')
    elif axis=='x':
        get_ren_label(ax=ax, renamer=renamer, axis='x')
    elif axis=='y':
        get_ren_label(ax=ax, renamer=renamer, axis='y')
    else:
        raise NotImplementedError('Axis %s not implemented!'%axis)


[docs]def tick_legend_renamer(ax, renamer={}, title=''):
    """Renames legend entries on axis and optionally insert a title"""
    legend = ax.axes.get_legend()
    if title != '':
        legend.set_title(title)
    for i in legend.texts:
        i.set_text(renamer[i.get_text()])


[docs]def tick_label_inserter(ax, pos=0, ins='', axis='both'):
    """Inserts a string to tick labels on axis (can be switched ['both','x','y'])."""
    def get_ren_label(ax, pos, ins, axis):
        if axis=='x':
            label = ax.get_xticklabels()
        elif axis=='y':
            label = ax.get_yticklabels()
        else:
            raise NotImplementedError('Axis %s not implemented!'%axis)
        label = pd.Series([item.get_text() for item in label])
        if pos == 0:
            label = label.apply(lambda x: '{}{}'.format(ins,x))
        elif pos == -1:
            label = label.apply(lambda x: '{}{}'.format(x,ins))
        else:
            label = label.apply(lambda x: '{}{}{}'.format(x[:pos],ins,x[pos:]))
        label = label.values
        if axis=='x':
            label = ax.set_xticklabels(label)
        elif axis=='y':
            label = ax.set_yticklabels(label)
        else:
            raise NotImplementedError('Axis %s not implemented!'%axis)
    if axis=='both':
        get_ren_label(ax=ax, pos=pos, ins=ins, axis='x')
        get_ren_label(ax=ax, pos=pos, ins=ins, axis='y')
    elif axis=='x':
        get_ren_label(ax=ax, pos=pos, ins=ins, axis='x')
    elif axis=='y':
        get_ren_label(ax=ax, pos=pos, ins=ins, axis='y')
    else:
        raise NotImplementedError('Axis %s not implemented!'%axis)


[docs]def plt_add_DaAnno(mdf, x_n, y_n, VIP, ax=None, xy_standard=(-6,6),
                   xy_ded = {1:(1,1),2:(-1,1),3:(-1,-1),4:(1,-1)},
                   pkwargs={'marker':'x', 'linestyle':'','color':'red', 
                            'label':'Points of interest'},
                   akwargs={'xycoords':'data', 'textcoords':'offset points',
                            'ha':"center", 'va':"center"}):
    """
    Adds data annotations to curve.

    Parameters
    ----------
    mdf : pd.DataFrame
        DataFrame of measured data.
    x_n : string
        Column name of data to plot on x-axis.
    y_n : string
        Column name of data to plot on y-axis.
    VIP : pd.Series
        Points of interest (name as index and index of mdf as values).
    ax : plt.subplot axis, optional
        Matplotlib axis object to plot. The default is None.
    xy_standard : tuple of int, optional
        Standard position of text, coordinates related to data. 
        The default is (-6,6).
    xy_ded : dict, optional
        Dictionary with positiioning multipliers to apply to xy_standard. 
        The default is {1:(1,1),2:(-1,1),3:(-1,-1),4:(1,-1)}.
    pkwargs : dict, optional
        Keyword arguments for plt.plot. 
        The default is {'marker':'x', 'linestyle':'','color':'red', 
                        'label':'Points of interest'}.
    akwargs : TYPE, optional
        Keyword arguments for plt.annotate. 
        The default is {'xycoords':'data', 'textcoords':'offset points',
                        'ha':"center", 'va':"center"}.

    Returns
    -------
    pd.DataFrame
        Combined postion and text (for control purposes).

    """
    def recalc_xy(n, xy_ded):
        d,r = divmod(n,max(xy_ded.keys()))
        d+=1
        mult=np.multiply(xy_ded[r],d)
        return mult
    udf=mdf.loc[VIP,[x_n,y_n]]
    udf['VIP']=VIP.index
    udfg=udf.groupby([x_n,y_n])
    t2=pd.DataFrame([])
    for g in udfg:
        tg=g[1]
        tg['n']=range(1, 1 + len(tg))
        t2=pd.concat([t2,tg])
    t = t2['n'].apply(lambda x: np.multiply(recalc_xy(x, xy_ded),xy_standard))
    t = t.apply(pd.Series)
    udf[['xtext','ytext']]=t

    if ax is None: ax = plt.gca()
    ax.plot(udf[x_n], udf[y_n], **pkwargs)
    j=np.int64(-1)
    for x in udf['VIP']:
        j+=1
        ax.annotate('%s'%x, xy=(udf[x_n].iloc[j],udf[y_n].iloc[j]),
                     xytext=(udf['xtext'].iloc[j],udf['ytext'].iloc[j]), **akwargs)
    return udf

#%% plot analyse

[docs]def curve_char_plotter(cps, cip, dfp, df,
                       head=None, 
                       xlabel=None, ylabel_l=None, ylabel_r=None, 
                       cco={'y':'m'},
                       cco_sc={},
                       ccd={'DQ1':'b','DQ2':'g','DQ3':'y'},
                       ccd_sc={'DQ1_sc':'b','DQ2_sc':'g','DQ3_sc':'y'},
                       cc={'Const':'b','Rise':'g','Fall':'r','Pos':'m','Neg':'y'},
                       disp_opt_DQ='Normalized', 
                       do_kwargs={'norm':'absmax', 'normadd':0.5,
                                  'th':0.05, 'th_option':'abs', 'th_set_val':0},
                       limDQ=False, limDQvals=[-1.1,1.1]):
    """
    Plotting method for mc_char.curve_characterizer results.

    Parameters
    ----------
    cps : pd.Dataframe (mc_char.curve_characterizer output)
        Characterization of input (for linear parts: linearisation and
                                   intersection to previous included).
    cip : pd.Series (mc_char.curve_characterizer output)
        Points of first characteriation (S=Start, E=End, I=Increase, D=Decrease).
    dfp : pd.Dataframe (mc_char.curve_characterizer output)
        Maxima and minima of input, as well as differential quotients.
    df : pd.Dataframe (mc_char.curve_characterizer output)
		Input, as well as differential quotients.
    head : string or None, optional
        Figure title. The default is None.
    xlabel : string or None, optional
        Label on x-axis. The default is None.
    ylabel_l : string or None, optional
        Label on left y-axis. The default is None.
    ylabel_r : string or None, optional
        Label on right y-axis. The default is None.
    cco : dict, optional
        Color codes for Max and Min markers in accordance with dfp column name.
        The default is {'y':'m'}.
    cco_sc : dict, optional
        Color codes for changes in sign markers in accordance with dfp column 
        name. The default is {}.
    ccd : dict, optional
        Color codes for differential quotient plotting.
        The default is {'DQ1':'b','DQ2':'g','DQ3':'y'}.
    ccd_sc : dict, optional
        Color codes for plotting of differential quotient sign changes. 
        The default is {'DQ1_sc':'b','DQ2_sc':'g','DQ3_sc':'y'}.
    cc : ict, optional
        Color codes for plotting of curve description in cps.
        The default is {'Const':'b','Rise':'g','Fall':'r','Pos':'m','Neg':'y'}.
    disp_opt_DQ : string, optional
        Normalization of differential quotients. The default is 'Normalized'.
    do_kwargs : dict, optional
        Keyword arguments for normalization of differential quotients.
        The default is {'norm':'absmax', 'normadd':0.5,
                        'th':0.05, 'th_option':'abs', 'th_set_val':0}.
    limDQ : bool, optional
        Limit plot on y-axis to limDQvals. The default is False.
    limDQvals : list of float, optional
        Lower and upper limits on y-axis for limDQ. The default is [-1.1,1.1].

    Raises
    ------
    NotImplementedError
        DESCRIPTION.

    Returns
    -------
    None.

    """
    if disp_opt_DQ == 'True':
        df_u_DQ = df
    else:
        df_n = normalize(df.loc(axis=1)[~df.columns.str.contains('_s.*',regex=True)],
                         norm=do_kwargs['norm'], 
                         normadd=do_kwargs['normadd'])
        if disp_opt_DQ == 'Normalized':
            df_u_DQ = df_n
        elif disp_opt_DQ == 'Normalized_th':
            df_u_DQ = threshhold_setter(pdo=df_n, th=do_kwargs['th'],
                                        option=do_kwargs['th_option'],
                                        set_val=do_kwargs['th_set_val'])
        else:
            raise NotImplementedError("Display option %s not implemented!"%disp_opt_DQ)
    
    fig, (ax1,ax3) = plt.subplots(nrows=2, ncols=1, 
                                  sharex=True, sharey=False, 
                                  figsize = (6.3,2*3.54))
    fig.suptitle(head)
    ax1.set_title('Curve, difference quotients and extrema')
    ax1.plot(df.x, df.y,'r-', label='x-y')
    for i in cco:
        a = dfp[dfp[i]=='Max'].index
        b = dfp[dfp[i]=='Min'].index
        ax1.plot(df.loc[a,'x'],  df.loc[a,'y'], 
                 color=cco[i], marker='^', linestyle='')
        ax1.plot(df.loc[b,'x'],  df.loc[b,'y'], 
                 color=cco[i], marker='v', linestyle='')
    for i in cco_sc:
        ax1.plot(df.loc[df[i],'x'],  df.loc[df[i],i.replace('_sc','')], 
                 color=cco_sc[i], marker='x', linestyle='')
    ax1.grid()
    ax1.set_ylabel(ylabel_l)
    ax2=ax1.twinx()
    for i in ccd:
        a = dfp[dfp[i]=='Max'].index
        b = dfp[dfp[i]=='Min'].index
        ax2.plot(df.x, df_u_DQ[i],
                 ccd[i]+'--', label=i)
        ax2.plot(df.loc[a,'x'], df_u_DQ.loc[a,i],  
                 color=ccd[i], marker='^', linestyle='')
        ax2.plot(df.loc[b,'x'], df_u_DQ.loc[b,i],  
                 color=ccd[i], marker='v', linestyle='')
    for i in ccd_sc:
        ax2.plot(df.loc[df[i],'x'], df_u_DQ.loc[df[i],i.replace('_sc','')], 
                 color=ccd_sc[i], marker='x', linestyle='')
    ax2.axhline(0.0, color='gray', linestyle=':')
    ax2.axhline(1.0, color='gray', linestyle=':')
    ax2.axhline(-1.0, color='gray', linestyle=':') 
    if limDQ:
        ax2.set_ylim(limDQvals)
    ax2.set_ylabel(ylabel_r) 
    
    ax3.set_title('Curve characterisation')
    ax3.plot(df.x, df.y,'r-', label='x-y')
    for i in cps.index[:-1]:
        i_S=cps.loc[i,'Start']
        i_E=cps.loc[i,'End']
        ax3.plot(df.loc[i_S:i_E].x, df.loc[i_S:i_E].y,
                 cc[cps.loc[i,'Type']]+'-', linewidth=10,
                 solid_capstyle='butt', alpha=0.2,
                 label=cps.loc[i,'Type'])
    for i in cip.index:
        ax3.plot(df.loc[i].x, df.loc[i].y, color='k', marker='$%s$'%cip[i])
    cps_i_crf=cps.loc[cps.Type.apply(lambda x: x in ['Const','Rise','Fall','Last'])].index
    ax3.plot(cps.loc[cps_i_crf,'IntP_x'], cps.loc[cps_i_crf,'IntP_y'],
             'b:', label='lin')
    ax3.grid()
    ax3.set_ylabel(ylabel_l)
    ax3.set_xlabel(xlabel)
    # handles, labels = fig.gca().get_legend_handles_labels()
    # by_label = dict(zip(labels, handles))
    for a in [ax1,ax2,ax3]:
        handles, labels = a.get_legend_handles_labels()
        if a==ax1:
            by_label = dict(zip(labels, handles))
        else:
            by_label.update(dict(zip(labels, handles)))
    fig.legend(by_label.values(), by_label.keys(),
               loc='lower right', bbox_to_anchor=(1, 0.25))
    fig.tight_layout()  # otherwise the right y-label is slightly clipped
    plt.show(block=False)
    plt.close(fig)

#%% Seaborn extra

[docs]def plt_ax_regfit(pdo, x, y=None,  
                  fit={}, ax=None, plt_d=False,
    			  label_d='Data', label_f=False,
                  xlabel=None, ylabel=None, title=None, legend=True,
                  skws={}, lkws={}, t_form={}):
    """
    Plots a regression (done by fitting.regfitret) and data (optional).
    Optional legend entry with fitting function.

    Parameters
    ----------
    pdo : pd.DataFrame
        Input data.
    x : str
        Column name for abscissa data.
    y : str or None
        Column name for ordinate data or none, if plt_d is False. 
        The default is None.
    fit : pd.DataFrame (result of fitting.regfitret), optional
        Least square regression fit results. The default is {}.
    ax : plt.axis or None, optional
        Axis to plote result. The default is None.
    plt_d : bool, optional
        Switch for plotting data. The default is False.
    label_d : string, optional
        Label for data plot. The default is 'Data'.
    label_f : bool, optional
        Label for function plot. The default is False.
    xlabel : string, optional
        label for x-axis. The default is None.
    ylabel : string, optional
        Label for y-axis. The default is None.
    title : string, optional
        Axis title. The default is None.
    skws : dict, optional
        Dictionary for scatter plot (data). The default is {}.
    lkws : dict, optional
        Dictionary for lineplot (function). The default is {}.
    legend : bool, optional
        Switch for showing legend. The default is True.

    Returns
    -------
    None.

    """
    if ax is None: ax = plt.gca()
    if not title is None: ax.set_title(title)
    if not xlabel is None: ax.set_xlabel(xlabel)
    if not ylabel is None: ax.set_ylabel(ylabel)
    if plt_d: 
        sns.scatterplot(x=x, y=y, data=pdo, ax=ax, label=label_d, **skws)
    xtmp = np.linspace(pdo[x].min(), pdo[x].max(), 101)
    ytmp = fit['Model'].eval(x=xtmp, **fit['PD'])
    if label_f==True:
        ltxt=fit['Exp_txt']+' ($R²$={:.3f})'.format(fit['Rquad'])
    elif label_f=="restring_eq":
        ltxt=regfitret_restring_func(
            reg_res=fit, xl=fit['varxsym'], yl=fit['varysym'], 
            t_form=t_form, rquad_add=False, rq_form='')
    elif label_f=="restring_eq_rquad":
        ltxt=regfitret_restring_func(
            reg_res=fit, xl=fit['varxsym'], yl=fit['varysym'], 
            t_form=t_form['var'], rquad_add=True, rq_form=t_form['Rquad'])
    else:
        ltxt='Fit-%s'%fit['Name']
    sns.lineplot(x=xtmp, y=ytmp, ax=ax, label=ltxt, **lkws)
    if legend: ax.legend()


[docs]def sns_pointplot_MMeb(ax, data, x,y, hue=None,
                       dodge=0.2, join=False, palette=None,
                       markers=['o','P'], scale=1, barsabove=True, capsize=4,
                       controlout=False):
    """Generate Pointplot with errorbars marking minimum and maximum instead of CI."""
    axt = sns.pointplot(data=data, 
                         x=x, y=y, hue=hue,
                         ax=ax, join=join, dodge=dodge, legend=False,scale=scale,
                         markers=markers, palette=palette,
                         ci=None, errwidth=1, capsize=.1)
    if hue is None:
        # erragg = data.groupby(x).agg(['mean','min','max'])[y]
        erragg = data.groupby(x).agg(['mean','min','max'],sort=False)[y]
    else:
        # erragg = data.groupby([hue,x]).agg(['mean','min','max'])[y]
        erragg = data.groupby([hue,x]).agg(['mean','min','max'],sort=False)[y]
    # very qnd!!! (sonst falsch zugeordnet, prüfen!!!)
    if hue is not None:
        orgind=[data[hue].drop_duplicates().to_list(),data[x].drop_duplicates().to_list()]
        erragg=erragg.sort_index().loc(axis=0)[pd.MultiIndex.from_product(orgind)]
        
    errors=erragg.rename({'mean':'M','min':'I','max':'A'},axis=1)
    errors=errors.eval('''Min = M-I
                          Max = A-M''').loc(axis=1)[['Min','Max']].T
    i=0
    for point_pair in axt.collections:
        if hue is None:
            if i<1:
                i+=1
                colors=point_pair.get_facecolor()[0]
                x_coords = []
                y_coords = []
                for x, y in point_pair.get_offsets():
                    x_coords.append(x)
                    y_coords.append(y)
                ax.errorbar(x_coords, y_coords, yerr=errors.values,
                            c=colors, fmt=' ', zorder=-1, barsabove=barsabove, capsize=capsize)
        elif (i<=len(errors.columns.get_level_values(0).drop_duplicates())-1):
            errcol=errors.columns.get_level_values(0).drop_duplicates()[i]
            i+=1
            colors=point_pair.get_facecolor()[0]
            x_coords = []
            y_coords = []
            for x, y in point_pair.get_offsets():
                x_coords.append(x)
                y_coords.append(y)
            ax.errorbar(x_coords, y_coords, yerr=errors.loc(axis=1)[errcol].values,
                        c=colors, fmt=' ', zorder=-1, barsabove=barsabove, capsize=capsize)
    if controlout:
        return erragg
```

---

© Copyright 2024, MarcGebhardt.

Built with Sphinx using a
theme
provided by Read the Docs.
