# Supplementary material for: Approach to Standardized Material Characterization of the Human Lumbopelvic System: Testing and Evaluation
Source: Bioengineering (Basel). 2025 Aug 11;12(8):862. doi: 10.3390/bioengineering12080862 (PMC12383908; doi:10.3390/bioengineering12080862)
Supplement: Supplementary file 1 [file bioengineering-12-00862-s001.zip › File S3 Evaluation code/ExMechEva-0.1.2/docs/_build/html/_modules/exmecheva/common/stat_ext.html]

exmecheva.common.stat\_ext — ExMechEva v0.1.2 documentation


ExMechEva

Contents:

- ExMechEva

ExMechEva

- Module code
- exmecheva.common.stat\_ext

---

# Source code for exmecheva.common.stat\_ext

```
# -*- coding: utf-8 -*-
"""
Created on Mon Dec  4 17:54:05 2023

@author: mgebhard
"""
import string
import numpy as np
import pandas as pd
from scipy import stats
import statsmodels
from statsmodels.stats.multicomp import MultiComparison
import scikit_posthocs as sck_ph
import seaborn as sb #bootstrapping method

from .helper import type_str_return
from .pd_ext import (pd_outsort, deal_dupl_index)
from .fitting import regfitret
from .output import str_indent

#%% statistical outliers

[docs]def stat_outliers(data, option='IQR', span=1.5,
                  out='all', outsort = 'ascending'):
    """
    Determine statistical outliers.

    Parameters
    ----------
    data : pd.Series or pd.DataFrame
        Input data.
    option : str, optional
        Determination option. Only 'IQR': interquartile-range-rule implemented.
        The default is 'IQR'.
    span : float, optional
        Span for inclusion (p.e.: 'IQr' and 1.5 leads to standard 
        1.5-interquartile-range-rule). The default is 1.5.
    out : str, optional
        Output option. Implemented are:
            - 'all': all data points outside range (all statistical outliers)
            - 'lower': data points lower then range
            - 'higher': data points higher then range
            - 'inner': data points inside range (exclusion of statistical outliers)
        The default is 'all'.
    outsort : str, optional
        Sorting of output. Implemented are 'ascending' and 'descending'.
        All other values will return data unsorted. The default is 'ascending'.

    Raises
    ------
    NotImplementedError
        Option not implemented.

    Returns
    -------
    stol : pd.Series or pd.DataFrame (like input data)
        Statistical outlier data acc. to options.

    """
    # if isinstance(data, pd.core.base.ABCSeries):
    #     d = data
    # elif isinstance(data, np.ndarray):
    #     d = pd.Series(data)
    # else:
    #     raise NotImplementedError("Datatype %s not implemented!"%type(data))
        
    if option == 'IQR':
        dQ1 = data.quantile(q=0.25)
        dQ3 = data.quantile(q=0.75)
        IQR = dQ3 - dQ1
        lBo = dQ1 - span * IQR
        uBo = dQ3 + span * IQR
    else:
        raise NotImplementedError("Option %s not implemented!"%option)
    
    if outsort == 'ascending':
        data = data.sort_values(ascending=True)
    elif outsort == 'descending':
        data = data.sort_values(ascending=False)
    else:
        data = data
        
    if out == 'all':
        stol = data[(data < lBo) | (data > uBo)].index.to_list()
    elif out == 'lower':
        stol = data[(data < lBo)].index.to_list()
    elif out == 'higher':
        stol = data[(data > uBo)].index.to_list()
    elif out == 'inner':
        stol = data[(data >= lBo) & (data <= uBo)].index.to_list()
    else:
        raise NotImplementedError("Output %s not implemented!"%out)
        
    return stol


[docs]def stat_box_vals(data, option='IQR', span=1.5):
    """
    Determine values of typical boxplot values (1st quantile, 3rd qhuantile)

    data : pd.Series or pd.DataFrame
        Input data.
    option : str, optional
        Determination option. Only 'IQR': interquartile-range-rule implemented.
        The default is 'IQR'.
    span : float, optional
        Span for inclusion (p.e.: 'IQr' and 1.5 leads to standard 
        1.5-interquartile-range-rule). The default is 1.5.

    Raises
    ------
    NotImplementedError
        Option not implemented.

    Returns
    -------
    box_vals : dict
        DESCRIPTION.
        - 'lBo': lower boarder of 1.5-IQR
        - 'minin': minimal value inside range
        - 'dQ1': first quartile
        - 'med': Median
        - 'dQ3': third quartile
        - 'maxin': maximal value inside range
        - 'uBo': upper boarder of 1.5-IQR
    inner_vals : pd.Series or pd.DataFrame (like input data)
        Index of values inside range (not statistical outlier).
    outer_vals : pd.Series or pd.DataFrame (like input data)
        Index of values outside range (statistical outlier).

    """
    if option == 'IQR':
        dQ1 = data.quantile(q=0.25)
        med = data.quantile(q=0.50)
        dQ3 = data.quantile(q=0.75)
        IQR = dQ3 - dQ1
        lBo = dQ1 - span * IQR
        uBo = dQ3 + span * IQR
        # box_vals = {'lBo':lBo,'dQ1':dQ1,'med':med,'dQ3':dQ3,'uBo':uBo}
        inner_vals = stat_outliers(data=data, option=option, span=span,
                                   out='inner', outsort=None)
        outer_vals = stat_outliers(data=data, option=option, span=span,
                                   # out='outer', outsort=None)
                                   out='all', outsort=None)
        minin = data.loc[inner_vals].min()
        maxin = data.loc[inner_vals].max()
        box_vals = {'lBo':lBo,'minin':minin,'dQ1':dQ1,'med':med,
                    'dQ3':dQ3,'maxin':maxin,'uBo':uBo}
    else:
        raise NotImplementedError("Option %s not implemented!"%option)
    return box_vals, inner_vals, outer_vals


[docs]def NaN_stat_outliers(df, numeric_only=True, 
                      option='IQR', span=1.5,
                      out='all', outsort=None):
    """
    Determine statistical outliers with respect to NaN values. Optional selects
    numerical data only.

    Parameters
    ----------
    df : pd.DataFrame
        Input data.
    numeric_only : bool, optional
        Numerical columns only. The default is True.
    option : str, optional
        Determination option. Only 'IQR': interquartile-range-rule implemented.
        The default is 'IQR'.
    span : float, optional
        Span for inclusion (p.e.: 'IQr' and 1.5 leads to standard 
        1.5-interquartile-range-rule). The default is 1.5.
    out : str, optional
        Output option. Implemented are:
            - 'all': all data points outside range (all statistical outliers)
            - 'lower': data points lower then range
            - 'higher': data points higher then range
            - 'inner': data points inside range (exclusion of statistical outliers)
        The default is 'all'.
    outsort : str, optional
        Sorting of output. Implemented are 'ascending' and 'descending'.
        All other values will return data unsorted. The default is None.

    Returns
    -------
    dfout : pd.DataFrame
        Statistical outlier data acc. to options.

    """
    if numeric_only:
        cols = df.select_dtypes(include=['int','float']).columns
    else:
        cols = df.columns
    kws = {'option':option, 'span':span,
           'out':out, 'outsort' : outsort}
    t=df[cols].agg(stat_outliers,**kws)
    dfout = df.copy(deep=True)
    for i in cols:
        dfout.loc[t[i],i]=np.nan
    return dfout

#%% additional descriptive values

[docs]def meanwoso(data, option='IQR', span=1.5,
             out='inner', outsort = None):
    """
    Determine mean value of input data without statistical outliers. 
    (see stat_outliers for more information)

    Parameters
    ----------
    data : pd.Series or pd.DataFrame
        Input data.
    option : str, optional
        Determination option. Only 'IQR': interquartile-range-rule implemented.
        The default is 'IQR'.
    span : float, optional
        Span for inclusion (p.e.: 'IQr' and 1.5 leads to standard 
        1.5-interquartile-range-rule). The default is 1.5.
    out : str, optional
        Output option. Only option inner makes sense.
        Implemented are:
            - 'all': all data points outside range (all statistical outliers)
            - 'lower': data points lower then range
            - 'higher': data points higher then range
            - 'inner': data points inside range (exclusion of statistical outliers)
        The default is 'inner'.
    outsort : str, optional
        Sorting of output. Implemented are 'ascending' and 'descending'.
        All other values will return data unsorted. The default is None.

    Returns
    -------
    dout : float or pd.Series
        Mean value/-s of input data without statistical outliers.

    """
    used = stat_outliers(data=data, option=option, span=span, 
                         out=out, outsort=outsort)
    dout = data.loc[used].mean()
    return dout

# Overwrite pandas
pd.Series.meanwoso = meanwoso
pd.DataFrame.meanwoso = meanwoso

[docs]def stdwoso(data, option='IQR', span=1.5,
            out='inner', outsort = None):
    """
    Determine standard deviation of input data without statistical outliers. 
    (see stat_outliers for more information)

    Parameters
    ----------
    data : pd.Series or pd.DataFrame
        Input data.
    option : str, optional
        Determination option. Only 'IQR': interquartile-range-rule implemented.
        The default is 'IQR'.
    span : float, optional
        Span for inclusion (p.e.: 'IQr' and 1.5 leads to standard 
        1.5-interquartile-range-rule). The default is 1.5.
    out : str, optional
        Output option. Only option inner makes sense.
        Implemented are:
            - 'all': all data points outside range (all statistical outliers)
            - 'lower': data points lower then range
            - 'higher': data points higher then range
            - 'inner': data points inside range (exclusion of statistical outliers)
        The default is 'inner'.
    outsort : str, optional
        Sorting of output. Implemented are 'ascending' and 'descending'.
        All other values will return data unsorted. The default is None.

    Returns
    -------
    dout : float or pd.Series
        Standard deviation value/-s of input data without statistical outliers.

    """
    used = stat_outliers(data=data, option=option, span=span, 
                         out=out, outsort=outsort)
    dout = data.loc[used].std()
    return dout

# Overwrite pandas
pd.Series.stdwoso = stdwoso
pd.DataFrame.stdwoso = stdwoso

[docs]def coefficient_of_variation(data, outsort=None, optmeanabs=True):
    """
    Determine coefficient of determination (standard deviation to mean value).

    Parameters
    ----------
    data : pd.Series or pd.DataFrame
        Input data.
    outsort : str, optional
        Sorting of output. Implemented are None 'ascending' and 'descending'.
        The default is None.
    optmeanabs : bool, optional
        Option for using absolute mean value (if False and mean is negative, 
        coefficient of variation will be negative).
        The default is True.

    Returns
    -------
    dout : float or pd.Series
        Coefficient of variation value/-s of input data.

    """
    dstd = data.std()
    dmean = data.mean()
    if optmeanabs: dmean = abs(dmean) #absolute value to prevent negative CV
    if (isinstance(dmean, float) and dmean == 0):
        dout  = np.nan
    else:
        dout  = dstd/dmean
    # dout  = dstd/dmean
    if not outsort is None:
        dout = pd_outsort(data = dout, outsort = outsort)
    return dout

# short naming vor aggregation

[docs]def cv(data, outsort=None, optmeanabs=True):
    """
    Short naming of funciton coefficient_of_variation.
    Readability improved by usage of pandas aggregate function (pd.agg).
    
    """
    return coefficient_of_variation(data=data, outsort=outsort)


[docs]def coefficient_of_variation_woso(data, option='IQR', span=1.5,
                                  out='inner', outsort=None, 
                                  optmeanabs=True):
    """
    Determine coefficient of variation of input data without statistical 
    outliers.(see stat_outliers for more information)

    Parameters
    ----------
    data : pd.Series or pd.DataFrame
        Input data.
    option : str, optional
        Determination option. Only 'IQR': interquartile-range-rule implemented.
        The default is 'IQR'.
    span : float, optional
        Span for inclusion (p.e.: 'IQr' and 1.5 leads to standard 
        1.5-interquartile-range-rule). The default is 1.5.
    out : str, optional
        Output option. Only option inner makes sense.
        Implemented are:
            - 'all': all data points outside range (all statistical outliers)
            - 'lower': data points lower then range
            - 'higher': data points higher then range
            - 'inner': data points inside range (exclusion of statistical outliers)
        The default is 'inner'.
    outsort : str, optional
        Sorting of output. Implemented are 'ascending' and 'descending'.
        All other values will return data unsorted. The default is None.
    optmeanabs : bool, optional
        Option for using absolute mean value (if False and mean is negative, 
        coefficient of variation will be negative).
        The default is True.

    Returns
    -------
    dout : float or pd.Series
        Coefficient of variation value/-s of input data without statistical 
        outliers.

    """
    dstd = stdwoso(data=data, option=option, 
                   span=span, out=out, outsort=None)
    dmean = meanwoso(data=data, option=option, 
                     span=span, out=out, outsort=None)
    if optmeanabs: dmean = abs(dmean) #absolute value to prevent negative CV
    if (isinstance(dmean, float) and dmean == 0):
        dout  = np.nan
    else:
        dout  = dstd/dmean
    # dout  = dstd/dmean
    if not outsort is None:
        dout = pd_outsort(data = dout, outsort = outsort)
    return dout

# short naming vor aggregation

[docs]def cvwoso(data, option='IQR', span=1.5, out='inner', 
           outsort=None, optmeanabs=True):
    """
    Short naming of funciton coefficient_of_variation_woso.
    Readability improved by usage of pandas aggregate function (pd.agg).
    
    """
    return coefficient_of_variation_woso(
        data=data, option=option, span=span, out=out, outsort=outsort
        )


[docs]def confidence_interval(data, confidence=0.95, method="Seaborn_Bootstrap",
                        func = np.nanmean, n_boot = 1000, axis = None,
                        units = None, seed = 0, outtype="List"):
    """
    Calculates the confidence interval of given data.

    Parameters
    ----------
    data : pandas.Series of float
        Series of values to be analysed.
    confidence : float, optional
        Confidence value. The default is 0.95.
    method : string, optional
        Method to use.
        Implemented are:
            - Seaborn_Bootstrap: Seaborn Bootstrapping (Compare Seaborn barplot)
            - Wald: Wald-Confidence-Interval
        The default is "Seaborn_Bootstrap".
    func : string or callable, optional
        DESCRIPTION. The default is "nanmean".
    n_boot : int, optional
        Number of iterations. The default is 1000.
    axis : int, optional
        Applied axis. The default is None.
    units : array, optional
        Sampling units (see seaborn.algorithms). The default is None.
    seed : Generator | SeedSequence | RandomState | int | None, optional
        Seed for rondom number generator. The default is 0.
    outtype : str, optional
        Switch for output (List, Dict, Min, Max). The default is List.

    Raises
    ------
    NotImplementedError
        Error to raise if option not implemented.

    Returns
    -------
    CI : list or or dict or value
        Confidence interval values [lower, higher].

    """
    def CIoutsel(inp, outtype):
        if outtype=="List":
            out = inp
        elif outtype=="Dict":
            out = {'CImin':inp[0],'CImax':inp[1]}
        elif outtype=="Min":
            out = inp[0]
        elif outtype=="Max":
            out = inp[1]        
        return out    
    # if numeric_only:
    #     if not pd.api.types.is_numeric_dtype(data):
    #         try:
    #             data=data.apply(pd.to_numeric, errors='raise')
    #         except:
    #             return CIoutsel([np.nan, np.nan], outtype)
    # if skipna:
    #     data=pd_exclnan(data,axis=axis)
    if method == "Wald":
        if confidence == 0.95 and func in [np.nanmean,"mean"]:
            m = data.mean().values[0]
            f = 1.96 # only implemented for 95%-confidence 
            v = np.sqrt(data.std()/data.count()).values[0]
            CI = np.array([m - f*v , m + f*v])
        else:
            raise NotImplementedError("Wald-Confidence-Interval only implemented for 95 % confidence!")
    # elif method == "Scikits_Bootstrap":
    #     import scipy
    #     import scikits.bootstrap as bootstrap
    #     CI = bootstrap.ci(data=data, statfunction=scipy.mean, alpha = 1-confidence, n_samples=n_boot)
    elif method == "Seaborn_Bootstrap":
        CI = sb.utils.ci(a=sb.algorithms.bootstrap(data, func= func, n_boot=n_boot,
                                                   axis=axis, units=units, seed=seed),
                         which = confidence*100, axis=axis)
    else:
        CI = [np.nan, np.nan]
        raise NotImplementedError("Method %s not implemented!"%method)
    return CIoutsel(CI, outtype)


[docs]def CImin(data, confidence=0.95, method="Seaborn_Bootstrap",
          func = np.nanmean, n_boot = 1000, axis = None,
          units = None, seed = 0):
    """Return only minimum of confidence interval. (Use same seed and n_boot!)"""
    out = confidence_interval(data=data,confidence=confidence,method=method,
                              func=func, n_boot=n_boot, axis=axis,
                              units=units, seed=seed,
                              outtype="Min")
    return out


[docs]def CImax(data, confidence=0.95, method="Seaborn_Bootstrap",
          func = np.nanmean, n_boot = 1000, axis = None,
          units = None, seed = 0):
    """Return only maximum of confidence interval. (Use same seed and n_boot!)"""
    out = confidence_interval(data=data,confidence=confidence,method=method,
                              func=func, n_boot=n_boot, axis=axis,
                              units=units, seed=seed,
                              outtype="Max")
    return out


[docs]def relative_deviation(a, b, axis=0):
    """
    Calculates the relative deviation from second argument to first.
    
    .. math::
        RD_{b-a} = (b-a)/a

    Parameters
    ----------
    a : int or float or pd.Series or pd.DataFrame
        Base value.
    b : pd.Series or pd.DataFrame
        Calculation value.
    axis : int, optional
        Identifyier for performing calculation. The default is 0.

    Raises
    ------
    NotImplementedError
        Combination of types not implemented.

    Returns
    -------
    out : int or float or pd.Series or pd.DataFrame
        Relative deviation result (type depends on input types).

    """
    ta=type_str_return(a)
    tb=type_str_return(b)
    tab=ta+'_'+tb
    if tab=='pdDF_pdDF':
        out=(b.sub(a)).div(a)
    elif tab=='pdSe_pdDF':
        out=(b.sub(a, axis=axis)).div(a, axis=axis)
    elif tab=='pdSe_pdSe':
        out=(b.sub(a)).div(a)
    elif tab in ['float_float','float_int','int_float']:
        out=(b-a)/a
    elif tab in ['float_pdSe','int_pdSe',
                 'float_pdDF','int_pdDF']:
        out=(b-a)/a
    else:
        raise NotImplementedError(
        "Combination of types not implemented ({} and {})!".format(
        ta,tb
        ))
    return out

#%% extended pandas aggregation

[docs]def pd_agg(pd_o, agg_funcs=['mean','median','std','max','min'], 
           numeric_only=False):
    """Aggregate pandas object with defined functions."""
    if (type(pd_o) is pd.core.series.Series):
        out = pd_o.agg(agg_funcs)
    elif (type(pd_o) is pd.core.frame.DataFrame):
        if numeric_only:
            cols = pd_o.select_dtypes(include=['int','float']).columns
        else:
            cols = pd_o.columns
        out = pd_o[cols].agg(agg_funcs)
    elif (type(pd_o) is pd.core.groupby.generic.DataFrameGroupBy):
        if numeric_only:
            cols = pd_o.first().select_dtypes(include=['int','float']).columns
        else:
            # cols = pd_o.columns
            cols = pd_o.first().columns
        out = pd_o.agg(agg_funcs)[cols]
    else:
        raise NotImplementedError("Type %s not implemented!"%type(pd_o))
    return out


[docs]def agg_add_ci(pdo, agg_funcs=['mean','std','min','max']):
    """Adds confidence interval to pandas aggregate function"""
    a=pdo.agg(agg_funcs)
    a1=pdo.agg(confidence_interval)
    a1.index=['ci_min','ci_max']
    a=pd.concat([a,a1])
    return a


[docs]def pd_agg_custom(pdo, agg_funcs=['mean',meanwoso,'median',
                                  'std',coefficient_of_variation, 
                                  stdwoso, coefficient_of_variation_woso,
                                  'min','max',confidence_interval],
                  numeric_only=False, 
                  af_ren={'coefficient_of_variation_woso':'CVwoso',
                          'coefficient_of_variation':'CV'},
                  af_unp={'confidence_interval': ['CImin','CImax']}):
    """
    Aggregate pandas object with defined functions, including unpacked 
    multi-value functions. 

    Parameters
    ----------
    pdo : pd.DataFrame or pd.Series
        Pandas object (DataFrame or Series).
    agg_funcs : list of functions, optional
        List of aggregatable functions (pandas aggregate accepted). 
        The default is ['mean',meanwoso,'median',
                        'std',coefficient_of_variation,stdwoso,
                        coefficient_of_variation_woso,
                        'min','max',confidence_interval].
    numeric_only : bool, optional
        Switch for consideration of numerical values only (int and float). The default is False.
    af_ren : dict, optional
        Dictionary for renaming of aggregate function names.
        The default is {'coefficient_of_variation_woso':'CVwoso',
                        'coefficient_of_variation':'CV'}.
    af_unp : dict, optional
        Dictionary for unpacking of aggregate function. 
        The default is {'confidence_interval': ['CImin','CImax']}.

    Returns
    -------
    pd.DataFrame or pd.Series
        Aggregation values depending on given functions.

    """
    def unpack_aggval(o, n, rn):
        """Unpack arguments acc. to dictionary."""
        tmp=o.loc[n].apply(lambda x: pd.Series([*x], index=rn))
        i = o.index.get_indexer_for([n])[0]
        s=pd.concat([o.iloc[:i],tmp.T,o.iloc[i+1:]],axis=0)
        return s
    pda = pd_agg(pd_o=pdo, agg_funcs=agg_funcs, numeric_only=numeric_only)
    for i in af_unp.keys():
        pda = unpack_aggval(pda, i, af_unp[i])
    if len(af_ren.keys())>0:
        pda = pda.rename(af_ren)
    return pda

#%% tests (distribution, variance, hypothesis,...)

[docs]def Dist_test(pds, alpha=0.05, mcomp='Shapiro', mkws={},
              skipna=True, add_out = False):
    """
    Distribution test of data to Hypothesis sample looks Gaussian (reject, if 
    p<=alpha).

    Parameters
    ----------
    pds : pd.Series
        Series of data.
    alpha : float, optional
        Test criterion to reject zero hypothesis (normal distribution).
        The default is 0.05.
    mcomp : str, optional
        Test method. The default is 'Shapiro'.
    mkws : dict, optional
        Keyword arguments for used test. The default is {}.
    skipna : bool, optional
        Switch for skipping NaNs in data. The default is True.
    add_out : bool or str, optional
        Switch for output. The default is False.

    Raises
    ------
    NotImplementedError
        Method not implemented.

    Returns
    -------
    str, Dict, Series, [Series, Series, str]
        Output of test results, depending on add_out.

    """
    if skipna:
        data = pds.dropna()
    else:
        data = pds
    ano_n = data.count()
    # problem with wraps(partial(*))(*)
    if mcomp in ['Shapiro','S','shapiro']:
        # stats_test  = wraps(partial(stats.shapiro, **mkws))(stats.shapiro)
        t = stats.shapiro(data, **mkws)
    elif mcomp in ['Normaltest','normaltest','K2','Ksquare',
                   'DAgostino','dagostino','D’Agostino’s K^2']:
        # stats_test  = wraps(partial(stats.normaltest, **mkws))(stats.normaltest)
        t = stats.normaltest(data, **mkws)
    else:
        raise NotImplementedError('Method %s for distribution test not implemented!'%mcomp)
    # t = stats_test(data)
    F = t.statistic
    p = t.pvalue
    if p < alpha:
        rtxt = 'H0 rejected!'
        H0=False
    else:
        rtxt = 'Fail to reject H0!'
        H0=True
    txt=("- F(%d) = %.3e, p = %.3e (%s)"%(ano_n,F,p,rtxt)) # Gruppen sind normalverteielt p<0.05
    odict={'N':ano_n, 'Stat':F, 'p':p, 'H0':H0}
    if add_out is True:
       return txt, t
    elif add_out=='Dict':
       return odict
    elif add_out=='Series':
       return pd.Series(odict)
    elif add_out=='Test':
       return data, pd.Series(odict), txt
    else:
       return txt


[docs]def Dist_test_multi(pdo, axis=0,
                    alpha=0.05, mcomps=['Shapiro','DAgostino'], mkws={},
                    skipna=True, add_out = 'DF'):
    """Performs multiple distribution tests."""
    df_out = pd.DataFrame([],dtype='O')
    for mcomp in mcomps:
        tmp=pdo.apply(Dist_test, alpha=alpha, mcomp=mcomp, mkws=mkws, 
                      skipna=skipna, add_out='Dict')
        tmp=tmp.apply(pd.Series)
        tmp.columns = pd.MultiIndex.from_product([[mcomp],tmp.columns])
        df_out=pd.concat([df_out,tmp],axis=1)
    return df_out


[docs]def group_Anova(df, groupby, ano_Var,
                group_str=None, ano_str=None, alpha=0.05):
    """Performs an one way ANOVA -> depricated (use group_ANOVA_MComp)"""
    ano_data=pd.Series([],dtype='O')
    j=0
    for i in df[groupby].drop_duplicates().values:
        ano_data[j]=df.loc[df[groupby]==i] #Achtung: keine statistics-Abfrage!
        j+=1
    ano_df1=df[groupby].drop_duplicates().count()-1 #Freiheitsgrad 1 = Gruppen - 1
    # ano_df2=df.count()[0]-(ano_df1+1) #Freiheitsgrad 2 = Testpersonen - Gruppen
    ano_df2=df[ano_Var].count()-(ano_df1+1) #Freiheitsgrad 2 = Testpersonen - Gruppen #23-02-20: auf Var bezogen
    
    if ano_str is None:
        ano_str = ano_Var
    if group_str is None:
        group_str = groupby
    [F,p]=stats.f_oneway(*[ano_data[i][ano_Var] for i in ano_data.index])
    if p < alpha:
        rtxt = 'H0 rejected!'
    else:
        rtxt = 'Fail to reject H0!'
    txt=("F(%4d,%4d) = %7.3f, p = %.3e, for %s to %s (%s)"%(
        ano_df1,ano_df2, F,p, ano_str,group_str,rtxt
        )) # Gruppen sind signifikant verschieden bei p<0.05
    return txt


[docs]def group_ANOVA_MComp(df, groupby, ano_Var, 
                      group_str=None, ano_str=None,
                      mpop = "ANOVA", alpha=0.05, group_ren={},
                      do_mcomp_a=1, mcomp='TukeyHSD', mpadj='bonf', Ffwalpha=2,
                      mkws={}, nan_policy='omit', check_resnorm=False,
                      add_T_ind=3, add_out = False):
    """
    Performs an one way variance analysis and multi comparision test for given 
    variable, in respect to given groups.
    Returns an output string with summary and optional additional test outputs.

    Parameters
    ----------
    df : pd.DataFrame
        Dataframe with groups and values as columns for statistical test.
    groupby : str
        Name of groups for statistical test.
    ano_Var : str
        Name of variable for statistical test.
    group_str : str, optional
        String for output implementation of group names. The default is None.
    ano_str : str, optional
        String for output implementation of value names. The default is None.
    mpop : str, optional
        Method for population test.
        Implemented are ANOVA and Kruskal-Wallis H-test.
        The default is ANOVA.
    alpha : float, optional
        Test criterion to reject zero hypothesis (all means are equal). 
        The default is 0.05.
    group_ren : dict, optional
        Renaming of groups in df. The default is {}.
    do_mcomp_a : int, optional
        Performance level of tukey test 
        (0 - never, 1 - only if p of ANOVA lower alpha, 2 - allways).
        The default is 1.
    mcomp : str, optional
        Method for multi comparison.
        Implemented are TukeyHSD, ttest_ind, ttest_rel, mannwhitneyu.
        The default is TukeyHSD.
    mpadj : str, optional
        Method for testing and adjustment of pvalues.
        For further information see: statsmodels.stats.multitest.multipletests
        The default is bonf.
    Ffwalpha : float, optional
        Factor for family-wise error in comparision to alpha.
        The default is 2.
    mkws : dict, optional
        Keyword arguments for used multi comparision test.
        p.e.: {'equal_var': False, 'nan_policy': 'omit'}
        The default is {}. 
    nan_policy : str, optional
        Handling of NaN values. The default is 'omit'.
    add_T_ind : int, optional
        Additional indentation of lines in txt for Tukey HSD. The default is 3.
    add_out : bool, optional
        Additional output request. The default is False.
    
    Returns
    -------
    txt : str
        Text output with ANOVA and optional Tukey-HSD results.
    [F,p] : [float,float], optional
        ANOVA results
    t : statsmodels.iolib.table.SimpleTable, optional
        Results of multi comaprison test.
    """
    atxt=''
    df=df.copy(deep=True) #No Override
    if not groupby in df.columns:
        if groupby in df.index.names:
            df[groupby]=df.index.get_level_values(groupby)
        else:
            raise KeyError("Group ether in columns nor in index names!")
    if not len(group_ren.keys()) == 0:
        # df[groupby] = df[groupby].replace(group_ren) #SettingWithCopyWarning
        df[groupby].replace(group_ren, inplace=True)
    # direct implemented, else: RecursionError: maximum recursion depth exceeded while calling a Python object
    # Atxt = group_ANOVA_Tukey(df, groupby, ano_Var, group_str, ano_str, alpha)
    ano_data=pd.Series([],dtype='O')
    j=0
    # for i in df[groupby].drop_duplicates().values:
    #     ano_data[j]=df.loc[df[groupby]==i] #Achtung: keine statistics-Abfrage!
    #     j+=1
    # ano_df1=df[groupby].drop_duplicates().count()-1 #Freiheitsgrad 1 = Gruppen - 1
    for i in df[groupby].drop_duplicates().values:
        tmp=df.loc[df[groupby]==i][ano_Var]
        if nan_policy == 'raise':
            if tmp.isna().any():
                raise ValueError('NaN in group %s detected!'%i)
        elif nan_policy == 'omit':
            tmp=tmp.dropna()
        if tmp.count()==0:
            atxt += '(-%s)'%i
        else:
            ano_data[i]=tmp
            j+=1
    ano_df1=j-1 #Freiheitsgrad 1 = Gruppen - 1
    # ano_df2=df.count()[0]-(ano_df1+1) #Freiheitsgrad 2 = Testpersonen - Gruppen
    ano_df2=df[ano_Var].count()-(ano_df1+1) #Freiheitsgrad 2 = Testpersonen - Gruppen #23-02-20: auf Var bezogen
    if ano_str is None:
        ano_str = ano_Var
    if group_str is None:
        group_str = groupby
    if mpop in ["ANOVA", "f_oneway", "F-Test"]:
        [F,p]=stats.f_oneway(*[ano_data[i] for i in ano_data.index])
    elif mpop in ["Kruskal-Wallis H-test", "kruskal", "H-test", "Kruskal-Wallis"]:
        [F,p]=stats.kruskal(*[ano_data[i] for i in ano_data.index])
    elif mpop in ["Friedmann", "FriedmannChi²", "friedmann", "friedmanchisquare"]:
        [F,p]=stats.friedmanchisquare(*[ano_data[i] for i in ano_data.index])
    else:
        raise NotImplementedError('Method %s for population test not implemented!'%mpop)
    if p < alpha:
        rtxt = 'H0 rejected!'
        if do_mcomp_a > 0: do_mcomp_a+=1
        H0=False
    else:
        rtxt = 'Fail to reject H0!'
        H0=True
    Atxt=("- F(%3d,%4d) = %7.3f, p = %.3e, for %s to %s (%s)"%(
        ano_df1,ano_df2, F,p, ano_str,group_str, rtxt+atxt
        )) # Gruppen sind signifikant verschieden bei p<0.05
    if check_resnorm:
        tmp=df[[groupby,ano_Var]].copy()
        tmp.index=pd.MultiIndex.from_arrays(
            [tmp.index,tmp.loc(axis=1)[groupby]]
            )
        tmp1=df.groupby(groupby)[ano_Var].mean()
        dtest=Dist_test(
            tmp[ano_Var]-tmp1, alpha=alpha, mcomp='Shapiro', add_out='Series'
            )
        Atxt+='  -> Residuals are normally distributed = {H0} (F({N:d}) = {Stat:.3e}, p = {p:.3e})'.format(**dtest)
    if do_mcomp_a >= 2:
        # t = pairwise_tukeyhsd(endog=df[ano_Var], groups=df[groupby], alpha=alpha)
        mcp = MultiComparison(data=df[ano_Var], groups=df[groupby])
        if mcomp=='TukeyHSD':
            t = mcp.tukeyhsd(alpha=alpha*Ffwalpha)
            Ttxt = str_indent(t.summary(),add_T_ind)
        elif mcomp=='Dunn':
            def dunn_o2(a,b):
                data=[a,b]
                pvalue = sck_ph.posthoc_dunn(data)
                pvalue = pvalue[1][2]
                statistic = np.nan
                #return dict(statistic=statistic,pvalue=pvalue)
                return [statistic,pvalue]
            t = mcp.allpairtest(dunn_o2, alpha=alpha*Ffwalpha, method=mpadj)[0]
            Ttxt = str_indent(t,add_T_ind)
        elif mcomp=='ttest_ind':
            t = mcp.allpairtest(stats.ttest_ind, alpha=alpha*Ffwalpha, method=mpadj)[0]
            Ttxt = str_indent(t,add_T_ind)
        elif mcomp=='ttest_rel':
            t = mcp.allpairtest(stats.ttest_rel, alpha=alpha*Ffwalpha, method=mpadj)[0]
            Ttxt = str_indent(t,add_T_ind)
        elif mcomp=='mannwhitneyu':
            t = mcp.allpairtest(stats.mannwhitneyu, alpha=alpha*Ffwalpha, method=mpadj)[0]
            Ttxt = str_indent(t,add_T_ind)
        elif mcomp=='wilcoxon':
            t = mcp.allpairtest(stats.ttest_rel, alpha=alpha*Ffwalpha, method=mpadj)[0]
            Ttxt = str_indent(t,add_T_ind)
        else:
            # problem with wraps(partial(*))(*)
            # if mcomp=='ttest_ind':
            #     stats_test  = wraps(partial(stats.ttest_ind, **mkws))(stats.ttest_ind)
            # elif mcomp=='ttest_rel':
            #     stats_test  = wraps(partial(stats.ttest_rel, **mkws))(stats.ttest_rel)
            # elif mcomp=='mannwhitneyu':
            # #     stats_test  = wraps(partial(stats.mannwhitneyu, **mkws))(stats.mannwhitneyu)
            # # elif mcomp=='wilcoxon':
            # #     stats_test  = wraps(partial(stats.wilcoxon, **mkws))(stats.wilcoxon)
            # else:
                raise NotImplementedError('Method %s for multi comparison not implemented!'%mcomp)
            # t = mcp.allpairtest(stats_test, alpha=alpha*Ffwalpha, method=mpadj)[0]
            # Ttxt = str_indent(t,add_T_ind)
        txt = Atxt + Ttxt
    else:
        t='No multi comparision done, see do_mcomp_a.'
        txt = Atxt
    if check_resnorm:
        outser=pd.Series({"DF1": ano_df1, "DF2":ano_df2,
                          "Normp": dtest['p'], "NormH0": dtest['H0'],
                          "Stat":F, "p": p, "H0": H0,
                          "txt": txt, "MCP": t})
    else:
        outser=pd.Series({"DF1": ano_df1, "DF2":ano_df2,
                          "Stat":F, "p": p, "H0": H0,
                          "txt": txt, "MCP": t})
    if add_out==True:
        return txt, [F,p], t
    elif add_out=='Series':
        return outser
    elif add_out=='Test':
        return outser, ano_data
    else:
        return txt


[docs]def MComp_interpreter(T_Result):
    """
    Interprets a test result from statsmodels multicomparision (Zero hypothesis 
    reject). Returns identified higher order groups in relation to original 
    groups.

    Parameters
    ----------
    T_Result : statsmodels.iolib.table.SimpleTable
        Simple table result from statsmodels.

    Returns
    -------
    dict2 : dict
        Dictionary of results.
    txt : str
        Textual output.

    """
    if isinstance(T_Result, statsmodels.iolib.table.SimpleTable):
        t=pd.DataFrame(T_Result.data[1:], columns=T_Result.data[0])
    else:
        t=pd.DataFrame(T_Result.summary().data[1:], 
                       columns=T_Result.summary().data[0])
    df_True = t.loc[t.reject==True,:]
    # letters = list(string.ascii_lowercase)
    letters = list(string.ascii_lowercase)+list(string.ascii_uppercase) # List out of index
    n = 0
    txt=''
    group1_list = df_True.group1.tolist() #get the groups from the df with only True (True df) to a list
    group2_list = df_True.group2.tolist()
    group3 = group1_list+group2_list #concat both lists
    group4 = list(set(group3)) #get unique items from the list
    group5 = [str(i) for i in group4 ] #convert unicode to a str
    group5.sort() #sort the list
    gen = ((i, 0) for i in group5) #create dict with 0 so the dict won't be empty when starts
    dictionary = dict(gen)
    group6 = [(group5[i],group5[j]) for i in range(len(group5)) for j in range(i+1, len(group5))] #get all combination pairs
    for pairs in group6: #check for each combination if it is present in df_True
        txt += '\n' + str(n)
        txt += '\n' + str(dictionary)
        try:
            a = df_True.loc[(df_True.group1==pairs[0])&(df_True.group2==pairs[1]),:] #check if the pair exists in the df
        except:
            a.shape[0] == 0

        if a.shape[0] == 0: #it mean that the df is empty as it does not appear in df_True so this pair is equal
            txt += '\n' + str('equal')
            if dictionary[pairs[0]] != 0 and dictionary[pairs[1]] == 0: #if the 1st is populated but the 2nd in not populated
                txt += '\n' + str("1st is populated and 2nd is empty")
                dictionary[pairs[1]] = dictionary[pairs[0]]
            elif dictionary[pairs[0]] != 0 and dictionary[pairs[1]] != 0: #if both are populated, check matching labeles
                txt += '\n' + str("both are populated")
                if len(list(set([c for c in dictionary[pairs[0]] if c in dictionary[pairs[1]]]))) >0: #check if they have a common label
                        txt += '\n' + str("they have a shared character")
                else:
                    txt += '\n' + str("equal but have different labels")
                 #check if the 1st group label doesn't appear in anyother labels, if it is unique then the 2nd group can have the first group label
                    m = 0 #count the number of groups that have a shared char with 1st group
                    j = 0 #count the number of groups that have a shared char with 2nd group
                    for key, value in dictionary.items():
                        if key != pairs[0] and len(list(set([c for c in dictionary[pairs[0]] if c in value])))==0:
                            m+=1
                    for key, value in dictionary.items():
                        if key != pairs[1] and len(list(set([c for c in dictionary[pairs[1]] if c in value])))==0:
                            j+=1
                    if m == len(dictionary)-1 and j == len(dictionary)-1: #it means that this value is unique because it has no shared char with another group
                        txt += '\n' + str("unique")
                        dictionary[pairs[1]] = dictionary[pairs[0]][0]
                    else:
                        txt += '\n' + str("there is at least one group in the dict that shares a char with the 1st group")
                        dictionary[pairs[1]] = dictionary[pairs[1]] + dictionary[pairs[0]][0]
            else:  # if it equals 0, meaning if the 1st is empty (which means that the 2nd must be also empty)
                txt += '\n' + str("both are empty")
                dictionary[pairs[0]] = letters[n]
                dictionary[pairs[1]] = letters[n]
        else:
            txt += '\n' + str("not equal")
            if dictionary[pairs[0]] != 0: # if the first one is populated (has a value) then give a value only to the second 
                txt += '\n' + str('1st is populated')
                # if the 2nd is not empty and they don't share a charcter then no change is needed as they already have different labels
                if dictionary[pairs[1]] != 0 and len(list(set([c for c in dictionary[pairs[0]] if c in dictionary[pairs[1]]]))) == 0:
                    txt += '\n' + str("no change")
                elif dictionary[pairs[1]] == 0: #if the 2nd is not populated give it a new letter
                    dictionary[pairs[1]] = letters[n+1]
                #if the 2nd is populated and equal to the 1st, then change the letter of the 2nd to a new one and assign its original letter to all the others that had the same original letter       
                elif  dictionary[pairs[1]] != 0 and len(list(set([c for c in dictionary[pairs[0]] if c in dictionary[pairs[1]]]))) > 0:
                    #need to check that they don't share a charcter
                    txt += '\n' + str("need to add a letter")
                    original_value = dictionary[pairs[1]]
                    dictionary[pairs[1]] = letters[n]
                    for key, value in dictionary.items():
                        if key != pairs[0] and len(list(set([c for c in original_value if c in value])))>0: #for any given value, check if it had a character from the group that will get a new letter, if so, it means  that they are equal and thus the new letter should also appear in the value of the "old" group 
                            dictionary[key] = original_value + letters[n]  #add the original letter of the group to all the other groups it was similar to
            else:
                txt += '\n' + str('1st is empty')
                dictionary[pairs[0]] = letters[n]
                dictionary[pairs[1]] = letters[n+1]
                txt += '\n' + str(dictionary)
            n+=1
    
    # get the letter out the dictionary
    labels = list(dictionary.values())
    labels1 = list(set(labels))
    labels1.sort()
    final_label = ''.join(labels1)
    
    df2=pd.concat([t.group1,t.group2])
    group_names=df2.unique()
    for GroupName in group_names:
        if GroupName in dictionary:
            txt += '\n' + str("already exists")
        else:
            dictionary[str(GroupName)] = final_label
    
    for key, value in dictionary.items(): #this keeps only the unique char per group and sort it by group
        dictionary[key] =  ''.join(set(value))
    
    dict2 = dict(sorted(dictionary.items())) # the final output
    return dict2, txt


[docs]def group_ANOVA_MComp_multi(df, group_main='Series', group_sub=['A'],
                            ano_Var=['WC_vol'],
                            mpop = "ANOVA", alpha=0.05, group_ren={},
                            do_mcomp_a=0, mcomp='TukeyHSD', mpadj='bonf', Ffwalpha=1,
                            mkws={}, check_resnorm=False,
                            Transpose=True):
    """
    Performs an one way ANOVA and multi comparision test for given variable,
    in respect to given groups and sub-groups.
    Returns an output string with summary and optional additional test outputs.

    Parameters
    ----------
    df : pd.DataFrame
        Dataframe with groups and values as columns for statistical test.
    group_main : str
        Main group for statistical test.
    group_sub : list of str
        Sub-groups for statistical test (p.e. variants).
    ano_Var : list of str
        Names of variables for statistical test.
    group_str : str, optional
        String for output implementation of group names. The default is None.
    mpop : str, optional
        Method for population test.
        Implemented are ANOVA and Kruskal-Wallis H-test.
        The default is ANOVA.
    alpha : float, optional
        Test criterion to reject zero hypothesis (all means are equal). The default is 0.05.
    group_ren : dict, optional
        Renaming of groups in df. The default is {}.
    do_mcomp_a : int, optional
        Performance level of tukey test 
        (0 - never, 1 - only if p of ANOVA lower alpha, 2 - allways).
        The default is 1.
    mcomp : str, optional
        Method for multi comparison.
        Implemented are TukeyHSD, ttest_ind, ttest_rel, mannwhitneyu.
        The default is TukeyHSD.
    mpadj : str, optional
        Method for testing and adjustment of pvalues.
        For further information see: statsmodels.stats.multitest.multipletests
        The default is bonf.
    Ffwalpha : float, optional
        Factor for family-wise error in comparision to alpha.
        The default is 2.
    mkws : dict, optional
        Keyword arguments for used multi comparision test.
        p.e.: {'equal_var': False, 'nan_policy': 'omit'}
        The default is {}.
    Transpose : bool, optional
        Switch for transposing result. The default is False.
    
    Returns
    -------
    df_out : pd.DataFrame
        DataFrame output with variance analyses and additional results.
    """
    df_out=pd.DataFrame([],dtype='O')
    for av in ano_Var:
        if len(group_sub) == 0:
            tmp=group_ANOVA_MComp(df=df, groupby=group_main, 
                                       ano_Var=av,
                                       group_str=group_main,
                                       ano_str=av,
                                       mpop=mpop, alpha=alpha,
                                       do_mcomp_a=do_mcomp_a, 
                                       mcomp=mcomp, 
                                       mpadj=mpadj, Ffwalpha=Ffwalpha, mkws=mkws,
                                       check_resnorm=check_resnorm,
                                       add_out = 'Series')
            # name='_'.join([group_main,av,sg])
            name=av
            df_out[name]=tmp
        else:
            for sg in group_sub:
                tmp=group_ANOVA_MComp(df=df, groupby=(group_main,sg), 
                                           ano_Var=(av,sg),
                                           group_str=group_main,
                                           ano_str='_'.join([av,sg]),
                                           mpop=mpop, alpha=alpha,
                                           do_mcomp_a=do_mcomp_a, 
                                           mcomp=mcomp, 
                                           mpadj=mpadj, Ffwalpha=Ffwalpha, mkws=mkws,
                                           check_resnorm=check_resnorm,
                                           add_out = 'Series')
                # name='_'.join([group_main,av,sg])
                name='_'.join([av,sg])
                df_out[name]=tmp
    if Transpose:
        df_out=df_out.T
    return df_out


[docs]def Hypo_test(df, groupby, ano_Var,
              group_str=None, ano_str=None,
              alpha=0.05, group_ren={},
              mcomp='TukeyHSD', mkws={},
              rel=False, rel_keys=[],
              deal_dupl_ind='raise',
              group_ord=None,
              add_T_ind=3, add_out = False):
    """
    Performs hypothesis test of given data according to selected method. 
    Returns results acc. to selected output switch.

    Parameters
    ----------
    df : pd.DataFrame
        Dataframe with groups and values as columns for statistical test.
    groupby : str
        Name of groups for statistical test.
    ano_Var : str
        Name of variable for statistical test.
    group_str : str, optional
        String for output implementation of group names. The default is None.
    ano_str : str, optional
        String for output implementation of value names. The default is None.
    alpha : float, optional
        Test criterion to reject zero hypothesis (all means are equal). 
        The default is 0.05.
    group_ren : dict, optional
        Renaming of groups in df. The default is {}.
    mcomp : TYPE, optional
        Method for hypothesis test. The default is 'TukeyHSD'.
        Implemented are:
            - 'TukeyHSD': Tukey HSD test (see scipy.stats.tukey_hsd)
            - 'ttest_ind': Independend t-test (see scipy.stats.ttest_ind)
            - 'ttest_rel': Related t-test (see scipy.stats.ttest_rel)
            - 'mannwhitneyu': Mann-Whittney-U-test (see scipy.stats.mannwhitneyu)
            - 'wilcoxon': Wilcoxon signed rank test (see scipy.stats.wilcoxon)
    mkws : dict, optional
        Keyword arguments for used hypothesis test.
        p.e.: {'equal_var': False, 'nan_policy': 'omit'}
        The default is {}. 
    rel : bool, optional
        Switch for relation of samples (True= related). The default is False.
    rel_keys : list, optional
        Variabel/column names for determining relation. The default is [].
    deal_dupl_ind : string, optional
        Behavior with duplicated indices (see deal_dupl_index).
        The default is 'raise'.
    group_ord : None or list, optional
        Selected groups. None will use all columns. The default is None.
    add_T_ind : int, optional
        Additional indentation of level in textual output. The default is 3.
    add_out : TYPE, optional
        Switch for output. The default is False.
        Implemented are:
            - False: only text output
            - True: text output (str) and test result (statistic, pvalue as float)
            - 'Series': pd.Series of degrees of freedom, statistics, p-value 
            and zero hypothesis test result to alpha
            - 'Test': complete output

    Raises
    ------
    ValueError
        More than two groups identified.
    NotImplementedError
        Method not implemented.

    Returns
    -------
    str or (str, float) or pd.Series or (pd.DataFrame, pd.Series, string)
        Output of hypothesis test acc. to selected switch(see add_out).

    """
    if ano_str is None:
        ano_str = ano_Var
    if group_str is None:
        group_str = groupby
    dft = df.copy(deep=True)
    if rel and rel_keys!=[]:
        dft.index = pd.MultiIndex.from_frame(dft.loc(axis=1)[rel_keys+[groupby]])
    else:
        dft.index = pd.MultiIndex.from_arrays([dft.index,
                                               dft.loc(axis=1)[groupby]])
    dft = dft[ano_Var]
    dft =deal_dupl_index(dft,deal_dupl_ind=deal_dupl_ind) # new 231026
    dft = dft.unstack(level=-1)
    if rel: dft=dft.dropna(axis=0)
    
    if group_ord is None:
        dfgr=dft.columns.values
    else:
        dfgr=group_ord
    if not len(dfgr)==2:
        raise ValueError('More than two groups (%s)!'%dfgr)
    a = dft[dfgr[0]].dropna()
    b = dft[dfgr[1]].dropna()
    ano_df2=a.count()+b.count()-2 #Freiheitsgrad = Testpersonen pro Gruppe - 1
    
    # problem with wraps(partial(*))(*)
    if mcomp=='TukeyHSD':
        # stats_test  = wraps(partial(stats.tukey_hsd, **mkws))(stats.tukey_hsd)
        t = stats.tukey_hsd(a,b, **mkws)
    elif mcomp=='ttest_ind':
        # stats_test  = wraps(partial(stats.ttest_ind, **mkws))(stats.ttest_ind)
        t = stats.ttest_ind(a,b, **mkws)
    elif mcomp=='ttest_rel':
        # stats_test  = wraps(partial(stats.ttest_rel, **mkws))(stats.ttest_rel)
        t = stats.ttest_rel(a,b, **mkws)
    elif mcomp=='mannwhitneyu':
        # stats_test  = wraps(partial(stats.mannwhitneyu, **mkws))(stats.mannwhitneyu)
        t = stats.mannwhitneyu(a,b, **mkws)
    elif mcomp=='wilcoxon':
        # stats_test  = wraps(partial(stats.wilcoxon, **mkws))(stats.wilcoxon)
        t = stats.wilcoxon(a,b, **mkws)
    else:
        raise NotImplementedError('Method %s for hyphotesis test not implemented!'%mcomp)
    # t = stats_test(a,b) # problem with wraps(partial(*))(*)
    F = t.statistic
    p = t.pvalue
    if p < alpha:
        rtxt = 'H0 rejected!'
        H0=False
    else:
        rtxt = 'Fail to reject H0!'
        H0=True
    txt=("- F(%d) = %.3e, p = %.3e, for %s to %s (%s)"%(
        ano_df2, F,p, ano_str,group_str, rtxt
        )) # Gruppen sind signifikant verschieden bei p<0.05
    
    if add_out is True:
        return txt, t
    elif add_out=='Series':
        return pd.Series({'DF2':ano_df2, 'Stat':F, 'p':p, 'H0':H0})
    elif add_out=='Test':
        return dft, pd.Series({'DF2':ano_df2, 'Stat':F, 'p':p, 'H0':H0}), txt
    else:
        return txt


[docs]def Hypo_test_multi(df, group_main='Series', group_sub=['A'],
                    ano_Var=['WC_vol'],
                    mcomp='mannwhitneyu', alpha=0.05, mkws={},
                    rel=False, rel_keys=[],
                    deal_dupl_ind='raise',
                    group_ord=None,
                    Transpose=True):
    """
    Performs hypothesis tests for given variable, in respect to given groups
    according to selected method. 
    Returns results acc. to selected output switch.

    Parameters
    ----------
    df : pd.DataFrame
        Dataframe with groups and values as columns for statistical test.
    group_main : str
        Main group for statistical test. The default is 'Series'.
    group_sub : list of str
        Sub-groups for statistical test (p.e. variants). The default is ['A'].
    ano_Var : list of str
        Names of variables for statistical test. The default is ['WC_vol'].
    mcomp : TYPE, optional
        Method for hypothesis test. The default is 'TukeyHSD'.
        Implemented are:
            - 'TukeyHSD': Tukey HSD test (see scipy.stats.tukey_hsd)
            - 'ttest_ind': Independend t-test (see scipy.stats.ttest_ind)
            - 'ttest_rel': Related t-test (see scipy.stats.ttest_rel)
            - 'mannwhitneyu': Mann-Whittney-U-test (see scipy.stats.mannwhitneyu)
            - 'wilcoxon': Wilcoxon signed rank test (see scipy.stats.wilcoxon)
    alpha : float, optional
        Test criterion to reject zero hypothesis (all means are equal). 
        The default is 0.05.
    mkws : dict, optional
        Keyword arguments for used hypothesis test.
        p.e.: {'equal_var': False, 'nan_policy': 'omit'}
        The default is {}. 
    rel : bool, optional
        Switch for relation of samples (True= related). The default is False.
    rel_keys : list, optional
        Variabel/column names for determining relation. The default is [].
    deal_dupl_ind : string, optional
        Behavior with duplicated indices (see deal_dupl_index).
        The default is 'raise'.
    group_ord : None or list, optional
        Changing of order of groups not implemented. The default is None.
    Transpose : bool, optional
        Switch for transposing result. The default is False.

    Returns
    -------
    df_out : pd.DataFrame
        Dataframe of test results indexed variable name and group.

    """
    df_out=pd.DataFrame([],dtype='O')
    if group_sub is None:
        for av in ano_Var:
            tmp=Hypo_test(df=df, groupby=group_main, 
                          ano_Var=av, alpha=alpha,
                          mcomp=mcomp, mkws=mkws,
                          rel=rel, rel_keys= rel_keys,
                          deal_dupl_ind=deal_dupl_ind,
                          add_out = 'Series')
            name=av
            df_out[name]=tmp
    else:
        # for sg in group_sub:
        #     for av in ano_Var:
        for av in ano_Var:
            for sg in group_sub:
                tmp=Hypo_test(df=df, groupby=(group_main,sg), 
                              ano_Var=(av,sg), alpha=alpha,
                              mcomp=mcomp, mkws=mkws,
                              # rel=rel, rel_keys=rel_keys, add_out = 'Series')
                              rel=rel, rel_keys=[(x,sg) for x in rel_keys],
                              deal_dupl_ind=deal_dupl_ind,
                              add_out = 'Series')
                # name='_'.join([group_main,av,sg])
                name='_'.join([av,sg])
                df_out[name]=tmp
    if Transpose:
        df_out=df_out.T
    return df_out


[docs]def CD_rep(pdo, groupby='Series', var='DEFlutoB', 
           det_met='SM-RRT', outtype='txt', tnform='{:.3e}'):
    """
    Calculate critical differences and compare to given mean difference.
    Can provide information on the comparability and/or repeatability of two 
    test series.

    Parameters
    ----------
    pdo : pd.DataFrame
        Dataframe with groups and values as columns for test.
    groupby : str
        Name of groups for statistical test.
    var : str
        Name of variable for statistical test.
    det_met : string, optional
        Determination method for deriving critical differences (CD). 
        The default is 'SM-RRT'.
        Implemented are (all only implemented for 95 % confidence):
            - 'SM-RRT': CD by standard deviation and size
            (see https://www.methodensammlung-bvl.de/resource/blob/208066/e536126ed1723145e51fc90b12736f5e/planung-und-statistische-auswertung-data.pdf)
            - 'CV-DC': CD by coefficient of variation and maximum 
            (see https://flexikon.doccheck.com/de/Kritische_Differenz)
            - 'CV': CD by coefficient of variation 
            (see https://link.springer.com/chapter/10.1007/978-3-662-48986-4_887)
            - 'SD': CD by standard deviation
            (see https://edoc.hu-berlin.de/bitstream/handle/18452/11713/cclm.1982.20.11.817.pdf?sequence=1
    outtype : string, optional
        Switch for output. The default is 'txt'.
        Implemented are:
            - 'Ser_all': pd.Series with all derived results
            - 'Tuple_all': tuple of all derived results
            - 'txt': single line text output showing results acc. repeatability
            - 'Series': pd.Series with important resulzs
    tnform : formatcode, optional
        Format for float in sttestual output. The default is '{:.3e}'.

    Raises
    ------
    NotImplementedError
        Method not implemented.

    Returns
    -------
    out : pd.Series or tuple or string
        Output according to switch (see outtype).

    """
    # grsagg=pdo.groupby(groupby)[var].agg(['mean','std','count'])
    grsagg=pdo[[groupby,var]].groupby(groupby).agg(['mean','std','count'])
    if isinstance(var,tuple):
        grsagg=grsagg.droplevel([0,1],axis=1)
    grs_sumhrn=grsagg['count'].apply(lambda x: 1/(2*x)).sum()
    grs_MD=grsagg['mean'].max()-grsagg['mean'].min()
    
    CDF= 1.96 * 2**0.5 # 2.8 only implemented for 95%-probability level (only two groups?)
    if det_met=='SM-RRT': #https://www.methodensammlung-bvl.de/resource/blob/208066/e536126ed1723145e51fc90b12736f5e/planung-und-statistische-auswertung-data.pdf
        allagg=pdo[var].agg(['mean','std','count'])
        CD=CDF*allagg['std']*np.sqrt(grs_sumhrn)
    elif det_met=='CV-DC': #https://flexikon.doccheck.com/de/Kritische_Differenz
        allagg=pdo[var].agg(['max',coefficient_of_variation])
        CD=abs(allagg['coefficient_of_variation']*CDF*allagg['max'])
    elif det_met=='CV': #https://link.springer.com/chapter/10.1007/978-3-662-48986-4_887
        allagg=pdo[var].agg([coefficient_of_variation])
        CD=abs(allagg['coefficient_of_variation'])*CDF
    elif det_met=='SD': #https://edoc.hu-berlin.de/bitstream/handle/18452/11713/cclm.1982.20.11.817.pdf?sequence=1
        allagg=pdo[var].agg(['std'])
        CD=abs(allagg['std'])*CDF
    else:
        raise NotImplementedError("Method %s not implemented!"%det_met)
        
    eta = grs_MD/CD
    # MD_l_CD=True if abs(grs_MD) < CD else False
    MD_l_CD=False
    t_grs_MD=tnform.format(grs_MD)
    t_CD    =tnform.format(CD)
    txt="{} >  {} -> Repeatability not verified! (eta={})".format(t_grs_MD,t_CD,eta,)
    if abs(grs_MD) <= CD:
        MD_l_CD=True
        txt="{} <= {} -> Repeatability verified! (eta={})".format(t_grs_MD,t_CD,eta,)
    
    if outtype=='Ser_all':
        out= pd.Series({'df_groups_agg':grsagg,'df_all_agg':allagg,
                        'groups_MD':grs_MD, 'CD':CD, 'eta':eta, 'MDlCD':MD_l_CD})
    elif outtype=='Tuple_all':
        out=grsagg,allagg,grs_MD,CD,eta,MD_l_CD
    elif outtype=='txt':
        out= txt
    elif outtype=='Series':
        out= pd.Series({'MD':grs_MD,'CD':CD, 'eta':eta, 'H0': MD_l_CD})
    return out


[docs]def CD_test_multi(df, group_main='Series', group_sub=['A'],
                  ano_Var=['WC_vol'],
                  det_met='SM-RRT',
                  Transpose=True):
    """
    Calculate critical differences and compare to given mean difference for 
    groups and subgroups.
    Can provide information on the comparability and/or repeatability of two 
    test series.

    Parameters
    ----------
    df : pd.DataFrame
        Dataframe with groups, subgroups and values as columns for test.
    group_main : str, optional
        Main group for statistical test. The default is 'Series'.
    group_sub : list of str, optional
        Sub-groups for statistical test (p.e. variants). The default is ['A'].
    ano_Var : list of str, optional
        Names of variables for statistical test.The default is ['WC_vol'].
    det_met : string, optional
        Determination method for deriving critical differences (CD). 
        The default is 'SM-RRT'.
        Implemented are (all only implemented for 95 % confidence):
            - 'SM-RRT': CD by standard deviation and size
            (see https://www.methodensammlung-bvl.de/resource/blob/208066/e536126ed1723145e51fc90b12736f5e/planung-und-statistische-auswertung-data.pdf)
            - 'CV-DC': CD by coefficient of variation and maximum 
            (see https://flexikon.doccheck.com/de/Kritische_Differenz)
            - 'CV': CD by coefficient of variation 
            (see https://link.springer.com/chapter/10.1007/978-3-662-48986-4_887)
            - 'SD': CD by standard deviation
            (see https://edoc.hu-berlin.de/bitstream/handle/18452/11713/cclm.1982.20.11.817.pdf?sequence=1
    Transpose : bool, optional
        Switch for transposing result. The default is False.

    Returns
    -------
    df_out : pd.DataFrame
        Derived test results (pd.Series({'MD':grs_MD,'CD':CD, 'eta':eta, 'H0': MD_l_CD})).

    """
    df_out=pd.DataFrame([],dtype='O')
    for av in ano_Var:
        for sg in group_sub:
            tmp=CD_rep(pdo=df, groupby=(group_main,sg), 
                          var=(av,sg), det_met=det_met,
                          outtype = 'Series')
            # name='_'.join([group_main,av,sg])
            name='_'.join([av,sg])
            df_out[name]=tmp
    if Transpose:
        df_out=df_out.T
    return df_out


[docs]def Multi_conc(df, group_main='Donor', anat='VA', 
               met='Kruskal', alpha=0.05,
               stdict={'WC_vol':['A','B','L'],'WC_vol_rDA':['B','C','L'],
                       'lu_F_mean':['B'],'DEFlutoB':['C','G','L'],
                       'Hyst_An':['B'],'DHAntoB':['C','G','L']},
               rel=False, rel_keys=[], kws={}):
    """
    Multiple conclusion of statistical tests (variance analysis, hyphotesis test or critical differences)

    Parameters
    ----------
    df : pd.DataFrame
        Dataframe.
    group_main : str, optional
        Main group name. The default is 'Donor'.
    anat : str, optional
        Type of test.
        Implemented are:
            - VA: variance analysis
            - VAwoSg: variance analysis without subgrouping
            - HT: hyphotesis test
            - CD: critical differences
        The default is 'VA'.
    met : str, optional
        Methode of test. The default is 'Kruskal'.
    alpha : float, optional
        Test criterion to reject zero hypothesis.
        The default is 0.05.
    stdict : dict, optional
        Dictionary of value and list of variants to evaluate.
        The default is {'WC_vol':['A','B','L'],'WC_vol_rDA':['B','C','L'],
                        'lu_F_mean':['B'],'DEFlutoB':['C','G','L'], 
                        'Hyst_An':['B'],'DHAntoB':['C','G','L']}.
    rel : bool, optional
        Switch for relation of samples (True= related). The default is False.
    rel_keys : list, optional
        Variabel/column names for determining relation. The default is [].
    kws : dict, optional
        Dictionarie of additional Keyword arguments for selected test. 
        The default is {}.

    Returns
    -------
    out : pd.DataFrame
        Dataframe of results.

    """
    out=pd.DataFrame([],dtype='O')
    for i in stdict.keys():
        if anat=='VA':
            out2=group_ANOVA_MComp_multi(df=df, 
                                 group_main=group_main, 
                                 group_sub=stdict[i],
                                 ano_Var=[i],
                                 mpop=met, alpha=alpha, **kws)
        if anat=='VAwoSg':
            out2=group_ANOVA_MComp_multi(df=df, 
                                 group_main=group_main, 
                                 group_sub=[],
                                 ano_Var=[i],
                                 mpop=met, alpha=alpha, **kws)
        elif anat=='HT':
            out2=Hypo_test_multi(df=df, 
                                 group_main=group_main, 
                                 group_sub=stdict[i],
                                 ano_Var=[i], mcomp=met, alpha=alpha,
                                 rel=rel, rel_keys=rel_keys, **kws)
        elif anat=='CD':
            out2=CD_test_multi(df=df, 
                                 group_main=group_main, 
                                 group_sub=stdict[i],
                                 ano_Var=[i], det_met=met, **kws)
        out=pd.concat([out,out2])
    out.index=pd.MultiIndex.from_product([[group_main],out.index])
    return out


[docs]def Corr_ext(df, method='spearman', 
             sig_level={0.001:'$^a$',0.01:'$^b$',0.05:'$^c$',0.10:'$^d$'},
             corr_round=2):
    """
    Performs correlation according to method and significance levels.

    Parameters
    ----------
    df : pd.DataFrame
        Input data.
    method : string, optional
        Correlation method, implemented are:
            - Pearson: ['pearson','Pearson','P']
            - Spearman: ['spearman', 'Spearman','S']
            - Kendalltau: ['kendall', 'kendalltau', 'Kendall', 'Kendalltau', 'K']
        The default is 'spearman'.
    sig_level : list or dict, optional
        Significance level for anotation, if list: ascending number of *´s, 
        if dict: dictionary values.
        The default is {0.001:'$^a$',0.01:'$^b$',0.05:'$^c$',0.10:'$^d$'}.
    corr_round : int or None, optional
        Number of digits for rounding of annotation of correlation. 
        The default is 2.

    Raises
    ------
    NotImplementedError
        Method fpr correlation not implemented.

    Returns
    -------
    pd.DataFrame, pd.DataFrame
        Output dataframes (Correlation, Annotation strings with significance levels).

    """
    # quelle: https://stackoverflow.com/questions/52741236/how-to-calculate-p-values-for-pairwise-correlation-of-columns-in-pandas
    if method in ['pearson','Pearson','P']:
        df_c = df.corr(method='pearson')
        pval = df.corr(method=lambda x, y: stats.pearsonr(x, y)[1])
    elif  method in ['spearman', 'Spearman','S']:
        df_c = df.corr(method='spearman')
        pval = df.corr(method=lambda x, y: stats.spearmanr(x, y)[1])
    elif method in ['kendall', 'kendalltau', 'Kendall', 'Kendalltau', 'K']:
        df_c = df.corr(method='kendall')
        pval = df.corr(method=lambda x, y: stats.kendalltau(x, y)[1])
    else:
        raise NotImplementedError('Correlation method %s not implemented!'%method)
    if isinstance(corr_round,int):
        df_c=df_c.round(corr_round)
    if isinstance(sig_level, list):
        p = pval.applymap(lambda x: ''.join(['*' for t in sig_level if x<=t]))
    else:
        def p_extender(x, sld):
            if x > np.max(list(sld.keys())):
                return ''
            else:
                for i in np.sort(list(sld.keys())):
                    if x <= i:
                        return sld[i]
        p = pval.applymap(lambda x: p_extender(x, sld=sig_level))
    tmpf='{'+':.'+'%d'%(corr_round) +'f}'
    df_c2 = df_c.applymap(lambda x: tmpf.format(x)) + p
    return df_c, df_c2


[docs]def reg_stats_multi(df, lRd, var_ren={}, var_sym={}, ftype='linear',
                    guess = dict(a=0.01, b=0.1), t_form='{a:.3e},{b:.3e}', 
       			  max_nfev=1000, nan_policy='omit',
                    ind=3, addind=3):
    """
    Performs multiple regression fits (according fitting.regfitret) on given 
    data. Returns a DataFrame and textual output.

    Parameters
    ----------
    df : pd.DataFrame
        Input data.
    lRd : list
        List of compared variables (must be column names in df).
        Example [['Density_app','Age']]
    var_ren : dict, optional
        Renamer for given variables (description). 
        Example dict('Density_app'='apparent density','Age'='donor age')
        The default is {}.
    var_sym : dict, optional
        Renamer for given variables (symbols).
        Example dict('Density_app'='rho_{app}','Age'='age')
        The default is {}.
    ftype : str, optional
        Type and name of function to adjust. 
        Implemented are:
            - 'linear': linear and constant function (see func_lin)
            - 'power': power and constant function (see func_pow)
            - 'exponential': exponantial and constant function (see func_exp)
            - additional endings:
                - '_nc': no constant value
                - '_x0': fixed to zero
        The default is 'linear'.
    guess : dictionary, optional
        Dictionary for first guess. The default is dict(a=0.01, b=0.1).
    t_form : str, optional
        String of dictionary with variables and format strings. 
        The default is '{a:.3e},{b:.3e}'.
    max_nfev : int, optional
        Maximum number of evaluations. The default is 1000.
    nan_policy : string, optional
        NaN policy (omit, raise or propagate). The default is 'omit'.
    ind : TYPE, optional
        Indentation for text output. The default is 3.
    addind : int, optional
        Additional indentation for text output. The default is 3.

    Returns
    -------
    reg_df : pd.Dataframe
        Results of regressions.
    txt : str
        Textual output.

    """
    reg_df = pd.DataFrame(lRd, columns=['vary','varx'])
    reg_df.index = reg_df['vary']+'-'+reg_df['varx']
    reg_df[['varyren','varxren']]=reg_df[['vary','varx']].replace(var_ren)
    reg_df[['varysym','varxsym']]=reg_df[['vary','varx']].replace(var_sym)
    tmp=reg_df.apply(
        lambda x: regfitret(
            pdo=df, x=x['varx'], y=x['vary'], 
            xl=x['varxsym'], yl=x['varysym'], xt=x['varxren'], yt=x['varyren'],
            name=ftype, guess=guess, t_form=t_form, 
            max_nfev=max_nfev, nan_policy=nan_policy, outtype='Series'
            ), axis=1
        )
    reg_df=pd.concat([reg_df,tmp],axis=1)
    txt=reg_df.apply(
        lambda x: str_indent(x.loc['Description'], ind)
            + str_indent(x.loc['Exp_txt'], ind+addind) 
            + str_indent(x.loc['Rep_txt'], ind+addind), axis=1
        )
    return reg_df, txt
```

---

© Copyright 2024, MarcGebhardt.

Built with Sphinx using a
theme
provided by Read the Docs.
