# Supplementary material for: Approach to Standardized Material Characterization of the Human Lumbopelvic System: Testing and Evaluation
Source: Bioengineering (Basel). 2025 Aug 11;12(8):862. doi: 10.3390/bioengineering12080862 (PMC12383908; doi:10.3390/bioengineering12080862)
Supplement: Supplementary file 1 [file bioengineering-12-00862-s001.zip › File S3 Evaluation code/ExMechEva-0.1.2/docs/_build/html/_modules/exmecheva/eva.html]

exmecheva.eva — ExMechEva 0.1.0 documentation


ExMechEva

Contents:

- ExMechEva

ExMechEva

- Module code
- exmecheva.eva

---

# Source code for exmecheva.eva

```
# -*- coding: utf-8 -*-
"""
Functionality for selection of evaluation (single, series or complete) and 
packing of HDF-file.

@author: MarcGebhardt
ToDo:
    - series only work with output_lvl>=1
"""
import os
import traceback
import pandas as pd

import exmecheva.common as emec

[docs]def series(eva_single_func, paths, no_stats_fc, var_suffix, 
           prot_rkws, output_lvl=1,
           log_scopt={'logfp':None, 'output_lvl': 1,
                      'logopt':True, 'printopt':False},
           plt_scopt={'tight':True, 'show':True, 
                      'save':True, 's_types':["pdf"], 
                      'clear':True, 'close':True}):
    """
    Evaluates a series of single evaluations (one after an other).

    Parameters
    ----------
    eva_single_func : function
        Function to perform single evaluation.
    paths : pandas.Series
        Paths for evaluation:
            - 'prot': path to protocoll excel table
            - 'opts': path to common options as json file
            - 'meas': path to measurement (conventional) file location
            - 'dic': path to measurement (optical) file location
            - 'out': path for output files
    no_stats_fc : list of strings
        No evaluation if in protocoll.Failure_code. 
        Example: no_stats_fc = ['A01.1','A01.2','A01.3', 'A02.3',
                                'B01.1','B01.2','B01.3', 'B02.3',
                                'C01.1','C01.2','C01.3', 'C02.3',
                                'D01.1','D01.2','D01.3', 'D02.3',
                                'F01.1','F01.2','F01.3', 'F02.3']
    var_suffix : string
        Suffix for variant of measurement (p.E. different moistures ["A","B",...]).
    prot_rkws : dict
        Dictionary for reading protocol. Must be keyword ind pandas.read_excel.
    output_lvl : int, optional
        Output level (0: no output, 1: only necessary, 2: all).
        The default is 1.

    Returns
    -------
    None.

    """
    prot = pd.read_excel(paths['prot'], **prot_rkws)
    
    logfp = paths['out'] + os.path.basename(paths['prot']).replace('.xlsx','.log')
    if output_lvl>=1: log_mg=open(logfp,'w')
        
    prot.Failure_code  = emec.list_ops.list_cell_compiler(prot.Failure_code)
    eva_b = emec.list_ops.list_interpreter(prot.Failure_code, no_stats_fc)
    
    emec.output.str_log("\n paths:",log_mg,output_lvl,printopt=False)
    for path in paths.index:
        emec.output.str_log("\n  %s: %s"%(path,paths[path]),
                            log_mg,output_lvl,printopt=False)
    emec.output.str_log("\n evaluation: %d / %d"%(prot.loc[eva_b].count()[0],prot.count()[0]),
                            log_mg,output_lvl,printopt=False)
    emec.output.str_log("\n%s"%prot.loc[eva_b].Designation.values,
                            log_mg,output_lvl,printopt=False)
    emec.output.str_log("\n not evaluated: %d / %d"%(prot.loc[eva_b==False].count()[0],prot.count()[0]),
                            log_mg,output_lvl,printopt=False)
    emec.output.str_log("\n%s"%prot.loc[eva_b==False].Designation.values,
                            log_mg,output_lvl,printopt=False)

    for eva in prot[eva_b].index:
        for mfile_add in var_suffix:
            emec.output.str_log("\n %s"%prot.loc[eva].Designation+mfile_add,
                                log_mg,output_lvl,printopt=False)  
            try:
                cout = eva_single_func(prot_ser = prot.loc[eva],
                                       paths = paths, mfile_add=mfile_add)
                emec.output.str_log("\n   Eva_time: %.5f s (Control: %s)"%(cout[0].iloc[-1]-cout[0].iloc[0],cout[1]),
                                log_mg,output_lvl,printopt=False)
            except Exception:
                txt = '\n   Exception:'
                txt+=emec.output.str_indent('\n{}'.format(traceback.format_exc()),5)
                emec.output.str_log(txt, log_mg,output_lvl,printopt=False)  

    if output_lvl>=1: log_mg.close()


[docs]def selector(eva_single_func, option, combpaths, no_stats_fc,
             var_suffix=[""], ser='', des='', out_path='',
             prot_rkws=dict(header=11, skiprows=range(12,13), index_col=0),
             log_scopt={'logfp':None, 'output_lvl': 1,
                        'logopt':True, 'printopt':False},
             plt_scopt={'tight':True, 'show':True, 
                        'save':True, 's_types':["pdf"], 
                        'clear':True, 'close':True}):
    """
    Selects suitable evaluation method acc. to choosen option.

    Parameters
    ----------
    eva_single_func : function
        Function to perform single evaluation.
    option : str
        Evaluation option. Possible are:
            - 'single': Evaluate single measurement
            - 'series': Evaluate series of measurements (see protocol table)
            - 'complete': Evaluate series of series
            - 'pack': Pack all evaluations into single hdf-file (only results and evaluated measurement)
            - 'pack-all': Pack all evaluations into single hdf-file with (all results, Warning: high memory requirements!)
    combpaths : pandas.DataFrame
        Combined paths for in- and output of evaluations.
    no_stats_fc : list of str
        Assessment codes for excluding from evaluation (searched in protocol variable "Failure_code").
    var_suffix : list of str, optional
        Suffix of variants of measurements (p.E. different moistures ["A","B",...]). 
        The default is [""].
    ser : str, optional
        Accessor for series. Must be as index in combpaths. The default is ''.
    des : str, optional
        Accessor for/Designation of measurement/specimen. 
        Must be as index in combpaths. The default is ''.
    out_path : str or Path, optional
        Additional outputpath for packed evaluation (hdf file). The default is ''.
    prot_rkws : dict, optional
        Dictionary for reading protocol. Must be keyword ind pandas.read_excel.
        The default is dict(header=11, skiprows=range(12,13),index_col=0).

    Raises
    ------
    NotImplementedError
        Option not implemented.

    Returns
    -------
    None.

    """
    if option == 'single':
        mfile_add = var_suffix[0]
        prot=pd.read_excel(combpaths.loc[ser,'prot'],**prot_rkws)
        try:
            _ = eva_single_func(prot_ser=prot[prot.Designation==des].iloc[0], 
                                paths=combpaths.loc[ser],
                                mfile_add = mfile_add,
                                log_scopt=log_scopt,
                                plt_scopt=plt_scopt)
        except Exception:
            txt = '\n   Exception:'
            txt+=emec.output.str_indent('\n{}'.format(traceback.format_exc()),5)
            emec.output.str_log(txt, None , output_lvl=1, 
                                logopt=False, printopt=True)  
        
    elif option == 'series':
        series(eva_single_func=eva_single_func,
               paths = combpaths.loc[ser],
               no_stats_fc = no_stats_fc,
               var_suffix = var_suffix,
               prot_rkws=prot_rkws,
               log_scopt=log_scopt,
               plt_scopt=plt_scopt)
        
    elif option == 'complete':
        for ser in combpaths.index:
            series(eva_single_func,
                   paths = combpaths.loc[ser],
                   no_stats_fc = no_stats_fc,
                   var_suffix = var_suffix,
                   prot_rkws=prot_rkws,
                   log_scopt=log_scopt,
                   plt_scopt=plt_scopt)
            
    elif option == 'pack':
        emec.loadnsave.comb_logs(in_paths=combpaths, out_path=out_path)
        packpaths = combpaths[['prot','out']]
        packpaths.columns=packpaths.columns.str.replace('out','hdf')
        emec.loadnsave.pack_hdf(
            in_paths=packpaths, out_path = out_path,
            hdf_naming = 'Designation', var_suffix = var_suffix,
            h5_conc = 'Material_Parameters', h5_data = 'Measurement',
            prot_rkws=prot_rkws, opt_pd_out = False, opt_hdf_save = True
            )
        print("Successfully created %s"%out_path)
        
    elif option == 'pack-all':
        emec.loadnsave.comb_logs(in_paths=combpaths, out_path=out_path)
        packpaths = combpaths[['prot','out']]
        packpaths.columns=packpaths.columns.str.replace('out','hdf')
        emec.loadnsave.pack_hdf_mul(
            in_paths=packpaths, out_path = out_path,
            hdf_naming = 'Designation', var_suffix = var_suffix,
            h5_conc = 'Material_Parameters',
            prot_rkws=prot_rkws, opt_pd_out = False, opt_hdf_save = True
            )
        print("Successfully created %s"%out_path)
        
    else:
        raise NotImplementedError('Option %s not implemented!'%option)
```

---

© Copyright 2024, MarcGebhardt.

Built with Sphinx using a
theme
provided by Read the Docs.
