# Supplementary material for: Approach to Standardized Material Characterization of the Human Lumbopelvic System: Testing and Evaluation
Source: Bioengineering (Basel). 2025 Aug 11;12(8):862. doi: 10.3390/bioengineering12080862 (PMC12383908; doi:10.3390/bioengineering12080862)
Supplement: Supplementary file 1 [file bioengineering-12-00862-s001.zip › File S3 Evaluation code/ExMechEva-0.1.2/docs/_build/html/_modules/exmecheva/Eva_ACT.html]

exmecheva.Eva\_ACT — ExMechEva v0.1.1 documentation


ExMechEva

Contents:

- ExMechEva

ExMechEva

- Module code
- exmecheva.Eva\_ACT

---

# Source code for exmecheva.Eva\_ACT

```
# -*- coding: utf-8 -*-
"""
Axial compression test evaluation.

@author: MarcGebhardt

ToDo:
    - reimplement optical measurement usage ("OPT_DIC")
"""

#%% 0 Imports
import pandas as pd
import numpy as np
import matplotlib.pyplot as plt
from datetime import datetime
import time
import warnings

import exmecheva.common as emec

warnings.filterwarnings('ignore',category=pd.io.pytables.PerformanceWarning)
warnings.filterwarnings('ignore',category=FutureWarning)

log_custom = emec.output.str_log
log_cind = emec.output.str_indent

plt_hsuf =  emec.plotting.plt_handle_suffix
figsize = plt.rcParams['figure.figsize']


#%% 1.0 Evaluation

[docs]def ACT_single(prot_ser, paths, mfile_add='',
               log_scopt={'logfp':None, 'output_lvl': 1,
                          'logopt':True, 'printopt':False},
               plt_scopt={'tight':True, 'show':True, 
                          'save':True, 's_types':["pdf"], 
                          'clear':True, 'close':True}):
    """
    Evaluate single axial compression test measurement form protocol table and 
    path collection. Using common options wich are overwritten by protocol 
    variables starting with 'OPT_'. Produce evaluated measurements, 
    material parameters and plots and safe them as table (.csv), unstructured 
    database (.h5) and document (.pdf), using the designation of the specimen 
    for distinction.
    Procedure:
        - 1: Read in options and presetting
        - 2: Determining geometrical values
        - 3: Read in measurements (conventional and optical (optinal))
        - 4: Merging measurements (time offset of conventional to optical 
                                   (if available), downsampling and merging)
        - 5: Determine evaluation space (start and end)
        - 6: Evaluation (curves (stress/strain), importent points on curves,
                         elastic moduli (different types implemented))
        - 7: Generating output (tables, database and plots)

    Parameters
    ----------
    prot_ser : pd.Series
        Input data as pandas series with specimen information 
        (identifier, origin, geometrical data, 
         assessment codes, evaluation options).
    paths : pd.DataFrame
        Path collection for in- and output paths. 
        Needs indexes: 
            - "opts": Common evaluation options
            - "prot": Protocol
            - "meas": Conventional measured data
            - "dic": Optical measured data
            - "out": Output
    mfile_add : string, optional
        Suffix of variants of measurements 
        (p.E. diffferent moistures ["A","B",...]). 
        The default is ''.
    log_scopt : dict, optional
        Options for custom logging. Determining file path, 
        output level (0=none, 1=normal, 2=special), logging enabled and 
        printing enabled.
        The default is {'logfp':None, 'output_lvl': 1,
                        'logopt':True, 'printopt':False}.
    plt_scopt : dict, optional
        Options for plotting. 
        The default is {'tight':True, 'show':True, 
                        'save':True, 's_types':["pdf"],
                        'clear':True, 'close':True}.

    Raises
    ------
    ValueError
        Input value not correct.
    NotImplementedError
        Method not implemented.

    Yields
    ------
    timings : pd.Series
        Timings of procedure steps.
    cout : string
        Special text output for control purposes.

    """
    out_name = prot_ser['Designation']+mfile_add
    out_full = paths['out']+out_name
    if log_scopt['output_lvl']>=1: 
        if log_scopt['logfp'] is None or (not isinstance(log_scopt['logfp'],str)):
            log_scopt['logfp'] = out_full+'.log'
        log_scopt['logfp']=open(log_scopt['logfp'],'w')
    log_scoptf={'logfp':log_scopt['logfp'], 
                'output_lvl': log_scopt['output_lvl'], 
                'logopt':log_scopt['logopt'], 
                'printopt':True}
    
    _opts=emec.eva_opt_hand.option_reader_sel(
        prot_ser=prot_ser, paths=paths, 
        search_inds=['Number','Designation','name'], 
        variant='',
        option='JFile+Prot',
        sheet_name="",
        re_rkws=dict()
        )
    
    path_meas = paths['meas']+_opts['OPT_File_Meas']+mfile_add+".xlsx"
    path_dic = paths['dic']+_opts['OPT_File_DIC']+mfile_add+".csv"
    
    plt_name = prot_ser['Designation']+mfile_add
    timings=pd.Series([],dtype='float64')
    timings.loc[0.0]=time.perf_counter()
    
    
    # rel_time_digs = Evac.sigdig(_opts['OPT_Resampling_Frequency'], 4)
    rel_time_digs = 2
    
           
    dic_used_Strain="Strain_opt_"+_opts['OPT_YM_Determination_refinement'][3]
    tmp_in = _opts['OPT_YM_Determination_refinement'][3].split('_')[-1]
    dic_used_Disp="Disp_opt_"+tmp_in
    
    if _opts['OPT_YM_Determination_refinement'][3].split('_')[-2] == 'd':
        tmp_md = '2'
    elif _opts['OPT_YM_Determination_refinement'][3].split('_')[-2] == 'c':
        tmp_md = '4'
    else:
        raise ValueError('OPT_YM_Determination_refinement seems to be wrong')
    loc_Yd_tmp = 'E_lsq_R_A%s%sl'%(tmp_md,tmp_in)
        
    cout =''
    ftxt=(("  Parameters of evaluation:"),
          ("   Evaluation start time:     %s" %datetime.now().strftime('%Y-%m-%d %H:%M:%S')),
          ("   Evaluation options:        %s" %paths['opts']),
          ("   Path protocol:             %s" %paths['prot']),
          ("   Path measurement:          %s" %path_meas),
          ("   Path optical- measurement: %s" %path_dic),
          ("   Resampling = %s (Frequency = %d Hz, moving-average = %s)" %(
              _opts['OPT_Resampling'], _opts['OPT_Resampling_Frequency'],
              _opts['OPT_Resampling_moveave']
              )),
          ("   Compression = %s" %(_opts['OPT_Compression'])),
          ("   LVDT failure = %s" %(_opts['OPT_LVDT_failure'])),
          ("   LVDT-spring-force-reduction = %s (K_Fed = %f N/mm)" %(
              _opts['OPT_Springreduction'], _opts['OPT_Springreduction_K']
              )),
          ("   Rise and curvature smoothing = %s (window = %d)" %(
              _opts['OPT_Rise_Smoothing'][0],_opts['OPT_Rise_Smoothing'][1]
              )),
          ("   Youngs Modulus determination between %f and %f of point %s" %(
              *_opts['OPT_YM_Determination_range'],
              )),
          ("   Distance between points: %d / %d steps " %(
              *_opts['OPT_Determination_Distance'],
              )),
          ("   Improvment of Youngs-Modulus-determination between %f*Stress_max and point %s," %(
              _opts['OPT_YM_Determination_refinement'][0],
              _opts['OPT_YM_Determination_refinement'][2]
              )),
          ("    with smoothing on difference-quotient (%s, %d)" %(
              _opts['OPT_YM_Determination_refinement'][4],
              _opts['OPT_YM_Determination_refinement'][5]
              )),
          ("    with allowable deviation of %f * difference-quotient_max in determination range" %(
              _opts['OPT_YM_Determination_refinement'][1]
              )),
          ("   DIC-Measurement = %s" %(_opts['OPT_DIC'])),
          ("   DIC-Strain-suffix for range refinement and plots = %s" %(
              _opts['OPT_YM_Determination_refinement'][3]
              )))
          # ("   DIC-minimal points (special / specimen) = %d / %d" %(_opts['OPT_DIC_Tester'][-2],_opts['OPT_DIC_Tester'][-1])),
          # ("   DIC-names of special points (l,r,head), = %s, %s, %s" %(*_opts['OPT_DIC_Points_TBT_device'],)),
          # ("   DIC-names of meas. points for fork (l,m,r), = %s, %s, %s" %(*_opts['OPT_DIC_Points_meas_fork'],)),
          # ("   DIC-maximal SD = %.3f mm and maximal displacement between steps %.1f mm" %(_opts['OPT_DIC_Tester'][0],_opts['OPT_DIC_Tester'][1])))
    log_custom('\n'.join(ftxt), **log_scopt)
    # =============================================================================
    #%% 2 Geometry
    log_custom("\n "+"="*100, **log_scopt)
    log_custom("\n ### 2 Geometry ###", **log_scoptf)
    
    if prot_ser['Test_Shape'] == 'Cube':
        Length = prot_ser['Length_%s'%prot_ser['Direction_test']]
        Volume = prot_ser['Length_x']*prot_ser['Length_y']*prot_ser['Length_z']
        Area   = Volume / Length
    elif prot_ser['Test_Shape'] == 'Cylinder':
        Length = prot_ser['Height']
        Volume = prot_ser['Diameter']**2 * np.pi()/4 *Length
        Area   = Volume / Length
    else:
        raise NotImplementedError("Test shape %s not implemented"%prot_ser['Test_Shape'])
    
    log_custom("\n    Length det=IN: %s (%.3f-%.3f)"%(Length==prot_ser['Length_test'],
                                                          Length,prot_ser['Length_test']),
               **log_scoptf)    
    log_custom("\n    Area   det=IN: %s (%.3f-%.3f)"%(Area==prot_ser['Area_CS'],
                                                          Area,prot_ser['Area_CS']),
               **log_scoptf) 
    log_custom("\n    Volume det=IN: %s (%.3f-%.3f)"%(Volume/1000==prot_ser['Volume'],
                                                          Volume/1000,prot_ser['Volume']),
               **log_scoptf)
    # reset
    Length = prot_ser['Length_test']   
    Area   = prot_ser['Area_CS']
    Volume = prot_ser['Volume']
    
    # =============================================================================
    
    #%% 3 Read in measurements
    log_custom("\n "+"="*100, **log_scopt)
    log_custom("\n ### 3 Read in measurements ###", **log_scoptf)
    timings.loc[3.0]=time.perf_counter()
    log_custom("\n   Timing %f: %.5f s"%(timings.index[-1],
                                       timings.iloc[-1]-timings.iloc[0]),
               **log_scopt)
    # =============================================================================
    
    #%%% 3.1 Read in conventional measurement data
    timings.loc[3.1]=time.perf_counter()
    log_custom("\n   Timing %f: %.5f s"%(timings.index[-1],
                                       timings.iloc[-1]-timings.iloc[0]),
               **log_scopt)
    mess=pd.read_excel(
        path_meas, header=_opts['OPT_Measurement_file']['header'],
        names=_opts['OPT_Measurement_file']['head_names']
        )
    
    mess = mess-mess.iloc[0]
    
    if isinstance(_opts['OPT_Measurement_file']['used_names_dict']['Way'],type(list())):
        mess['L_IWA']=pd.Series(
            mess[_opts['OPT_Measurement_file']['used_names_dict']['Way']].mean(axis=1)
            )
        # automatic number of LVDTs by list elements in used way
        n_IWAs=len(_opts['OPT_Measurement_file']['used_names_dict']['Way'])
        _opts['OPT_Measurement_file']['used_names_dict']['Way'] ='L_IWA'
    #Applying spring correction factor (if true)
    if _opts['OPT_Springreduction']: 
        mess['F_IWA_red']=mess['L_IWA']*_opts['OPT_Springreduction_K']*n_IWAs
        
    # # =============================================================================
    # #%%% 3.2 Specify used conventional measured force and way
    timings.loc[3.2]=time.perf_counter()
    log_custom("\n   Timing %f: %.5f s"%(timings.index[-1],
                                       timings.iloc[-1]-timings.iloc[0]),
               **log_scopt)
    messu = pd.DataFrame({
        'Time': mess[_opts['OPT_Measurement_file']['used_names_dict']['Time']],
        'Force': mess[_opts['OPT_Measurement_file']['used_names_dict']['Force']],
        'Way': mess[_opts['OPT_Measurement_file']['used_names_dict']['Way']]
        })

    if _opts['OPT_Springreduction']:     
        messu['Force'] = messu['Force'] - mess['F_IWA_red']
    if _opts['OPT_LVDT_failure'][0]:     
        messu['Way'] = _opts['OPT_LVDT_failure'][1]*mess[_opts['OPT_LVDT_failure'][2]]
        
    if _opts['OPT_Compression']==True:
        messu.Force=messu.Force*(-1)
    if np.invert(np.isnan(_opts['OPT_End'])):
        messu=messu.loc[messu.Time.round(rel_time_digs) <= _opts['OPT_End']]
    
    # =============================================================================
    #%%% 3.3 Read in optical measurement data
    timings.loc[3.3]=time.perf_counter()
    log_custom("\n   Timing %f: %.5f s"%(timings.index[-1],
                                       timings.iloc[-1]-timings.iloc[0]),
               **log_scopt)
    if _opts['OPT_DIC']:
        dic=None
        dicu=None
        dic_dt=None
        step_range_dic=None
        raise NotImplementedError('DIC not implemented')
        
    # =============================================================================
    #%% 4 Merging measurements
    log_custom("\n "+"="*100, **log_scopt)
    log_custom("\n ### 4 Merging measurements ###", **log_scoptf)
    timings.loc[4.0]=time.perf_counter()
    log_custom("\n   Timing %f: %.5f s"%(timings.index[-1],
                                       timings.iloc[-1]-timings.iloc[0]),
               **log_scopt)
    # =============================================================================
    #%%% 4.1 Determine time offset between conventional and optical measurement
    timings.loc[4.1]=time.perf_counter()
    log_custom("\n   Timing %f: %.5f s"%(timings.index[-1],
                                       timings.iloc[-1]-timings.iloc[0]),
               **log_scopt)
    if _opts['OPT_DIC']:
        mun_tmp = messu.loc[abs(messu.Way.loc[:(messu.Way.idxmax())]-messu.Way.max()/8).idxmin():
                            abs(messu.Way.loc[:(messu.Way.idxmax())]-messu.Way.max()/4).idxmin()]
        linvm   = np.polyfit(mun_tmp.loc[:,'Time'],mun_tmp.loc[:,'Way'],1)
        tsm     = -linvm[1]/linvm[0]
        mun_tmp = dicu.loc[abs(dicu.Disp_opt_head.loc[:(dicu.Disp_opt_head.idxmax())]-dicu.Disp_opt_head.max()/8).idxmin():
                           abs(dicu.Disp_opt_head.loc[:(dicu.Disp_opt_head.idxmax())]-dicu.Disp_opt_head.max()/4).idxmin()]
        linvd   = np.polyfit(mun_tmp.loc[:,'Time'],mun_tmp.loc[:,'Disp_opt_head'],1)
        tsd     = -linvd[1]/linvd[0]
        toff    = tsm-tsd
        
        if True:
            maxt_tmp=max(messu.Time.loc[abs(messu.Way.loc[:(messu.Way.idxmax())]-messu.Way.max()/4).idxmin()],dicu.Time.loc[abs(dicu.Disp_opt_head.loc[:(dicu.Disp_opt_head.idxmax())]-dicu.Disp_opt_head.max()/4).idxmin()])
            xlin_tmp=np.linspace(min(tsm,tsd),maxt_tmp,11)
            
            fig, ax1 = plt.subplots()
            ax1.set_title('%s - Way-measuring time difference'%plt_name)
            ax1.set_xlabel('Time / s')
            ax1.set_ylabel('Way / mm')
            ax1.plot(messu.Time.loc[messu.Time<=maxt_tmp], messu.Way.loc[messu.Time<=maxt_tmp], 'r-', label='PM-way')
            ax1.plot(dicu.Time.loc[dicu.Time<=maxt_tmp],   dicu.Disp_opt_head.loc[dicu.Time<=maxt_tmp], 'b-', label='DIC-way')
            ax1.plot(xlin_tmp,np.polyval(linvm,xlin_tmp),'y:',label='PM-lin')
            ax1.plot(xlin_tmp,np.polyval(linvd,xlin_tmp),'g:',label='DIC-lin')
            ax1.axvline(x=tsm, color='red', linestyle=':', label='PM-way-start')
            ax1.axvline(x=tsd, color='blue', linestyle=':', label='DIC-way-start')
            ax1.legend()
            ftxt=('$t_{S,PM}$  = % 2.4f s '%(tsm),
                  '$t_{S,DIC}$ = % 2.4f s '%(tsd))
            fig.text(0.95,0.15,'\n'.join(ftxt),
                     ha='right',va='bottom', 
                     bbox=dict(boxstyle='round', edgecolor='0.8', 
                               facecolor='white', alpha=0.8))
            plt_hsuf(fig,path=out_full+"-toff",**plt_scopt)
            del xlin_tmp
        
        log_custom("\n "+"-"*100, **log_scopt)
        log_custom("\n   Time offset between PM and DIC: %.3f s" %(toff),
                   **log_scoptf)
    else:
        toff=0.0
        
    messu.Time=(messu.Time-toff).round(rel_time_digs)
    
    # =============================================================================
    #%%% 4.2 Downsampling of conventional data
    timings.loc[4.2]=time.perf_counter()
    log_custom("\n   Timing %f: %.5f s"%(timings.index[-1],
                                       timings.iloc[-1]-timings.iloc[0]),
               **log_scopt)
    
    messu, _, mess_f = emec.mc_man.mc_resampler(
        mdf=messu, 
        t_col=_opts['OPT_Measurement_file']['used_names_dict']['Time'], 
        resample=_opts['OPT_Resampling'], 
        res_frequ=_opts['OPT_Resampling_Frequency'], 
        move_ave=_opts['OPT_Resampling_moveave'], 
        ma_sampler='data_rf',
        rel_time_digs=rel_time_digs) 
    
    # =============================================================================
    #%%% 4.3 Merge optical to conventional data
    timings.loc[4.3]=time.perf_counter()
    log_custom("\n   Timing %f: %.5f s"%(timings.index[-1],
                                       timings.iloc[-1]-timings.iloc[0]),
               **log_scopt)
    if _opts['OPT_DIC']:
        dicu.Time=dicu.Time.round(rel_time_digs)
        ind=pd.RangeIndex(dicu.loc[dicu.Time>=messu.Time.min()].index[0],
                          messu.Time.count()+dicu.loc[dicu.Time>=messu.Time.min()].index[0],1)
        messu=messu.merge(dicu,how='left', on='Time').set_index(ind)
        
    else:
        # dic_f=round(1/dic_dt)
        dic_f=mess_f
        
    f_vdm=dic_f/mess_f
        
    # =============================================================================
    #%% 5 Start and End
    log_custom("\n "+"="*100, **log_scopt)
    log_custom("\n ### 5 Start and End ###", **log_scoptf)
    timings.loc[5.0]=time.perf_counter()
    log_custom("\n   Timing %f: %.5f s"%(timings.index[-1],
                                       timings.iloc[-1]-timings.iloc[0]),
               **log_scopt)                                                    
    # =============================================================================
    #%%% 5.1 Determine start and end of evaluation
    timings.loc[5.1]=time.perf_counter()
    log_custom("\n   Timing %f: %.5f s"%(timings.index[-1],
                                       timings.iloc[-1]-timings.iloc[0]),
               **log_scopt)
    if np.isnan(_opts['OPT_End']):
        dic_to_mess_End=messu.iloc[-1].name
    else:
        # dic_to_mess_End=messu.loc[messu.Time<=prot.loc[prot_lnr]['DIC_End']*1/f_vdm].index[-1]
        dic_to_mess_End=messu.loc[messu.Time<=_opts['OPT_End']].index[-1]
        
    mun_tmp=messu.loc[:min(dic_to_mess_End,messu.Force.idxmax()),'Force']
    messu['driF'],messu['dcuF'],messu['driF_schg']=emec.mc_char.rise_curve(
        messu.loc[:dic_to_mess_End]['Force'],
        _opts['OPT_Rise_Smoothing'][0], _opts['OPT_Rise_Smoothing'][1]
        )
    
    for i in messu.index: # Startpunkt über Vorzeichenwechsel im Anstieg
        if messu.loc[i,'driF_schg']:
            if not messu.loc[
                    i+1:i+max(int(_opts['OPT_Determination_Distance'][0]/2),1),
                    'driF_schg'
                    ].any():
                messu_iS=i
                break
    
    messu_iS,_=emec.mc_char.find_SandE(
        messu.loc[messu_iS:messu_iS+_opts['OPT_Determination_Distance'][0],
                                         'driF'],
        abs(messu['driF']).quantile(0.5),"pgm_other",0.1)
    _,messu_iE=emec.mc_char.find_SandE(messu['driF'],0,"qua_self",0.5)
    messu_iE=min(messu_iE,dic_to_mess_End)
    
    log_custom("\n "+"-"*100, **log_scopt)
    log_custom("\n   Start of evaluation after %.3f seconds, corresponds to %.5f %% of max. force."
               %(messu.Time[messu_iS],100*abs(messu.Force[messu_iS])/abs(messu.Force).max()),
               **log_scopt)
    
    messu=messu.loc[messu_iS:messu_iE]
    if _opts['OPT_DIC']:
        step_range_dic = emec.pd_ext.pd_combine_index(step_range_dic,messu.index)
        
    fig, ax1 = plt.subplots()
    ax1.set_title('%s - Measuring'%plt_name)
    color1 = 'tab:red'
    ax1.set_xlabel('Time / s')
    ax1.set_ylabel('Force / N', color=color1)  # we already handled the x-label with ax1
    ax1.tick_params(axis='y', labelcolor=color1)
    ax1.axvline(x=messu.Time.loc[messu_iS], color='gray', linestyle='-')
    ax1.axvline(x=messu.Time.loc[messu_iE], color='gray', linestyle='-')
    if np.invert(np.isnan(_opts['OPT_End'])):
        ax1.axvline(x=_opts['OPT_End'], color='gray', linestyle='-.')
    ax1.plot(mess.Time, -mess.F_WZ, 'r-', label='Force-WZ')
    ax1.plot(mess.Time, -mess.F_PM, 'm-', label='Force-PM')
    if _opts['OPT_Springreduction']: 
        ax1.plot(mess.Time, mess.F_IWA_red, 'b:', label='Force-red. (LVDT)')
    ax2 = ax1.twinx()  # instantiate a second axes that shares the same x-axis
    ax2.grid(False)
    color2 = 'tab:blue'
    ax2.set_ylabel('Way / mm', color=color2)  # we already handled the x-label with ax1
    ax2.tick_params(axis='y', labelcolor=color2)
    ax2.plot(mess.Time, -(mess.L_PM), 'y--', label='Way-PM')
    ax2.plot(mess.Time, mess.L_IWA1-mess.L_IWA1[0], 'b--', label='Way-IWA1')
    ax2.plot(mess.Time, mess.L_IWA2-mess.L_IWA2[0], 'b-.', label='Way-IWA2')
    if _opts['OPT_DIC']:
        ax2.plot(dic.Time, dic.Disp_opt_head, 'k:', label='Way-DIC')
        # ax2.plot(dic.Time, dic.DDisp_PM_c, 'm:', label='Way-DIC-P')
        # ax2.plot(dic.Time, dic.DDisp_PC_c, 'g:', label='Way-DIC-C')
    fig.legend(loc='upper left', bbox_to_anchor=(0.1, 0.9), ncol=1)
    plt_hsuf(fig,path=out_full+"-meas",**plt_scopt)
    
    # =============================================================================
    #%%% 5.2 Resetting way
    timings.loc[5.2]=time.perf_counter()
    log_custom("\n   Timing %f: %.5f s"%(timings.index[-1],
                                       timings.iloc[-1]-timings.iloc[0]),
               **log_scopt)
    messu.Way=messu.Way-messu.Way.loc[messu_iS]
    
    if _opts['OPT_DIC']:    
        messu.Disp_opt_head=messu.Disp_opt_head-messu.Disp_opt_head.loc[messu_iS]
        
    fig, ax1 = plt.subplots()
    ax1.set_title('%s - Measuring (used)'%plt_name)
    color1 = 'tab:red'
    ax1.set_xlabel('Time / s')
    ax1.set_ylabel('Force / N', color=color1)  # we already handled the x-label with ax1
    ax1.tick_params(axis='y', labelcolor=color1)
    ax1.axvline(x=messu.Time.loc[messu_iS], color='gray', linestyle='-')
    ax1.axvline(x=messu.Time.loc[messu_iE], color='gray', linestyle='-')
    ax1.plot(messu.Time, messu.Force, 'r-', label='Force')
    ax2 = ax1.twinx()  # instantiate a second axes that shares the same x-axis
    ax2.grid(False)
    color2 = 'tab:blue'
    ax2.set_ylabel('Way / mm', color=color2)  # we already handled the x-label with 
    ax2.tick_params(axis='y', labelcolor=color2)
    ax2.plot(messu.Time, messu.Way, 'b--', label='Way')
    if _opts['OPT_DIC']:
        ax2.plot(messu.Time, messu.Disp_opt_head, 'k:', label='Way-DIC')
        # ax2.plot(messu.Time, messu.DDisp_PM_c, 'm:', label='Way-DIC-P')
        # ax2.plot(messu.Time, messu.DDisp_PC_c, 'g:', label='Way-DIC-C')
    #fig.legend(loc='lower right', bbox_to_anchor=(0.9, 0.15), ncol=2)
    fig.legend(loc='upper left', bbox_to_anchor=(0.1, 0.9), ncol=1)
    plt_hsuf(fig,path=out_full+"-meas_u",**plt_scopt)
    
    # =============================================================================
    #%% 6 Evaluation
    log_custom("\n "+"="*100, **log_scopt)
    log_custom("\n ### 6 Evaluation ###", **log_scoptf)
    timings.loc[6.0]=time.perf_counter()
    log_custom("\n   Timing %f: %.5f s"%(timings.index[-1],
                                       timings.iloc[-1]-timings.iloc[0]),
               **log_scopt)
    
    #%%% 6.2 Determine evaluation curves
    log_custom("\n "+"-"*100, **log_scopt)
    log_custom("\n ### -6.2 Determine evaluation curves ###", **log_scopt)
    timings.loc[6.2]=time.perf_counter()
    log_custom("\n   Timing %f: %.5f s"%(timings.index[-1],
                                       timings.iloc[-1]-timings.iloc[0]),
               **log_scopt)
    
    messu['Strain']=messu.Way/Length
    messu['Stress']=messu.Force/Area

    if _opts['OPT_DIC']:
        tmon=emec.mc_char.test_pdmon(
            messu,
            ['Stress','Strain',
             'Strain_opt_d_A','Strain_opt_d_S','Strain_opt_d_M',
             'Strain_opt_c_A','Strain_opt_c_S','Strain_opt_c_M'],
            1,10
            )
    else:
        tmon=emec.mc_char.test_pdmon(messu,['Stress','Strain'],1,10)
    
    log_custom("\n   Last 10 monoton increasing periods:\n    %s"
                   %tmon.to_frame(name='Epoche').T.to_string().replace('\n','\n    '),
               **log_scoptf)
    
    # =============================================================================
    #%%% 6.3 Determine points of interest
    log_custom("\n "+"-"*100, **log_scopt)
    log_custom("\n ### -6.3 Determine points of interest ###", **log_scopt)
    timings.loc[6.31]=time.perf_counter()
    log_custom("\n   Timing %f: %.5f s"%(timings.index[-1],
                                       timings.iloc[-1]-timings.iloc[0]),
               **log_scopt)
    VIP_messu=pd.Series([],dtype='int64',name='VIP_messu')
    VIP_messu['S']=messu.driF.index[0]
    VIP_messu['E']=messu.driF.index[-1]
    VIP_messu['U']=messu.Force.idxmax()
    
    mun_tmp = messu.loc[VIP_messu['S']+_opts['OPT_Determination_Distance'][1]:VIP_messu['U']-1]
    if mun_tmp.driF_schg.any()==True: # 
        VIP_messu['Y']=mun_tmp.loc[mun_tmp.driF_schg==True].index[0]-1
    else:
        VIP_messu['Y']=VIP_messu['U']
        log_custom('\n    Fy set on datapoint of Fu!', **log_scoptf)
    
    if _opts['OPT_Determination_SecHard']:
        mun_tmp = messu.loc[VIP_messu['Y']+_opts['OPT_Determination_Distance'][1]:VIP_messu['E']-1]
        if mun_tmp.driF_schg.any()==True: # 
            # VIP_messu['E']=mun_tmp.loc[mun_tmp.driF_schg==True].index[0]
            # log_mg.write('\n    End set on first inflection after Y!')
            i=mun_tmp.loc[mun_tmp.driF_schg==True].index[0]
            VIP_messu['SE']=VIP_messu['E']
            VIP_messu['E']=abs(
                messu.Force.loc[i-_opts['OPT_Determination_Distance'][1]:
                                i+_opts['OPT_Determination_Distance'][1]*2]
                    ).idxmin()
            # VIP_messu['E']=abs(messu.Force.loc[i-step_change:i+step_change]).idxmin()
            log_custom('\n    End set on first minimum near inflection after Y!',
                       **log_scoptf)
            VIP_messu['U']=messu.loc[:VIP_messu['E'],'Force'].idxmax()
            log_custom('\n      -> Ultimate set before new Endpoint!',
                       **log_scoptf)    
        else:
            VIP_messu['E']=VIP_messu['E']
            VIP_messu['SE']=VIP_messu['E']
            log_custom('\n    End left on old Endpoint!', **log_scoptf)    
        
    # mun_tmp = messu.loc[VIP_messu['U']:VIP_messu['E']-1]
    mun_tmp = messu.loc[VIP_messu['U']-1:VIP_messu['E']-1]
    if mun_tmp.driF_schg.any():
        i=mun_tmp.loc[mun_tmp.driF_schg].index[0]
        # statt allgemeinem Minimum bei größtem Kraftabfall nahe Maximalkraft,
        #  -2 da differenz aussage über vorherigen punkt
        VIP_messu['B'] = mun_tmp.driF.loc[
            i:i+_opts['OPT_Determination_Distance'][1]
            ].idxmin()-2 
        if VIP_messu['B']<VIP_messu['U']: VIP_messu['B']=VIP_messu['U']
    else:
        log_custom('\n   Fb not reliably determinable!', **log_scoptf)
            
    
    ftmp=float(
        messu.Force.loc[VIP_messu[_opts['OPT_YM_Determination_range'][2]]]
        *_opts['OPT_YM_Determination_range'][0])
    VIP_messu['F1']=abs(
        messu.Force.loc[:VIP_messu[_opts['OPT_YM_Determination_range'][2]]]
        -ftmp).idxmin()
    ftmp=float(
        messu.Force.loc[VIP_messu[_opts['OPT_YM_Determination_range'][2]]]
        *_opts['OPT_YM_Determination_range'][1])
    VIP_messu['F2']=abs(
        messu.Force.loc[:VIP_messu[_opts['OPT_YM_Determination_range'][2]]]
        -ftmp).idxmin()
    # Test ob Streckgrenze zwischen F1 und F2 liegt
    if (VIP_messu['Y']>VIP_messu['F1']) and (VIP_messu['Y']<VIP_messu['F2']): 
        VIP_messu['F2']=VIP_messu['Y']
        log_custom("\n   F2 set on Y (Force-rise between F1 and old F2)",
                   **log_scoptf)
    
    if _opts['OPT_Determination_SecHard']:
        log_custom("\n   Second hardening is used:", **log_scoptf)
        VIP_messu['SU']=messu.loc[VIP_messu['E']:].Force.idxmax()
        
        mun_tmp = messu.loc[
            VIP_messu['E']+_opts['OPT_Determination_Distance'][1]:VIP_messu['SU']-1]
        if mun_tmp.driF_schg.any()==True: # 
            VIP_messu['SY']=mun_tmp.loc[mun_tmp.driF_schg==True].index[0]-1
        else:
            VIP_messu['SY']=VIP_messu['SU']
            log_custom("\n     Second Fy set on first point with rise of 0!",
                       **log_scoptf)

    
    VIP_messu=VIP_messu.sort_values()
    if _opts['OPT_DIC']:
        VIP_dicu=VIP_messu.copy(deep=True)
        VIP_dicu.name='VIP_dicu'
    
    #%%%% 6.3.2 Improvement of evaluation range
    timings.loc[6.32]=time.perf_counter()
    log_custom("\n   Timing %f: %.5f s"%(timings.index[-1],
                                       timings.iloc[-1]-timings.iloc[0]),
              **log_scopt)
    
    # (siehe Keuerleber, M. (2006). Bestimmung des Elastizitätsmoduls von Kunststoffen bei hohen Dehnraten am Beispiel von PP. Von der Fakultät Maschinenbau der Universität Stuttgart zur Erlangung der Würde eines Doktor-Ingenieurs (Dr.-Ing.) genehmigte Abhandlung. Doktorarbeit. Universität Stuttgart, Stuttgart.)
    ftmp=float(messu.Stress.loc[VIP_messu[_opts['OPT_YM_Determination_range'][2]]]*_opts['OPT_YM_Determination_refinement'][0])
    Lbord=abs(messu.Stress.loc[:VIP_messu[_opts['OPT_YM_Determination_range'][2]]]-ftmp).idxmin()
    
    DQcon=pd.concat(emec.mc_char.Diff_Quot(
        messu.loc[:,'Strain'],
        messu.loc[:,'Stress'],
        _opts['OPT_YM_Determination_refinement'][4],
        _opts['OPT_YM_Determination_refinement'][5]
        ), axis=1)
    DQcon=messu.loc(axis=1)[['Strain','Stress']].join(DQcon,how='outer')
    DQcons=DQcon.loc[Lbord:VIP_messu[_opts['OPT_YM_Determination_refinement'][2]]]
    VIP_messu['FM']=DQcons.DQ1.idxmax()
    try:
        VIP_messu['F3']=DQcons.loc[:VIP_messu['FM']].iloc[::-1].loc[(DQcons.DQ1/DQcons.DQ1.max())<_opts['OPT_YM_Determination_refinement'][1]].index[0]+1
        # VIP_messu['F3']=DQcons.loc[:VIP_messu['FM']].iloc[::-1].loc[(DQcons.DQ1/DQcons.DQ1.max())<_opts['OPT_YM_Determination_refinement'][1]].index[0]
    except IndexError:
        VIP_messu['F3']=DQcons.index[0]
    try: # Hinzugefügt am 16.09.2021
        VIP_messu['F4']=DQcons.loc[VIP_messu['FM']:].loc[(DQcons.DQ1/DQcons.DQ1.max())<_opts['OPT_YM_Determination_refinement'][1]].index[0]-1
        # VIP_messu['F4']=DQcons.loc[VIP_messu['FM']:].loc[(DQcons.DQ1/DQcons.DQ1.max())<_opts['OPT_YM_Determination_refinement'][1]].index[0]
    except IndexError:
        VIP_messu['F4']=VIP_messu['FM']-1 #-1 könnte zu Problemen führen
    VIP_messu=VIP_messu.sort_values()
    
    
    if _opts['OPT_Determination_SecHard']:
        ttmp = messu.Stress.loc[[VIP_messu['E'],VIP_messu[_opts['OPT_YM_Determination_refSecHard'][2]]]]
        ftmp=float(ttmp.iloc[0]+(ttmp.iloc[-1]-ttmp.iloc[0])*_opts['OPT_YM_Determination_refSecHard'][0])
        Lbord=abs(messu.Stress.loc[VIP_messu['E']:VIP_messu[_opts['OPT_YM_Determination_refSecHard'][2]]]-ftmp).idxmin()
        
        DQsec=pd.concat(emec.mc_char.Diff_Quot(
            messu.loc[:,'Strain'],
            messu.loc[:,'Stress'],
            _opts['OPT_YM_Determination_refSecHard'][4],
            _opts['OPT_YM_Determination_refSecHard'][5]
            ), axis=1)
        DQsec=messu.loc(axis=1)[['Strain','Stress']].join(DQsec,how='outer')
        DQsecs=DQsec.loc[Lbord:VIP_messu[_opts['OPT_YM_Determination_refSecHard'][2]]]
        VIP_messu['SM']=DQsecs.DQ1.idxmax()
        try:
            VIP_messu['S3']=DQsecs.loc[:VIP_messu['SM']].iloc[::-1].loc[(DQsecs.DQ1/DQsecs.DQ1.max())<_opts['OPT_YM_Determination_refSecHard'][1]].index[0]+1
            # VIP_messu['F3']=DQcons.loc[:VIP_messu['FM']].iloc[::-1].loc[(DQcons.DQ1/DQcons.DQ1.max())<_opts['OPT_YM_Determination_refinement'][1]].index[0]
        except IndexError:
            VIP_messu['S3']=DQsecs.index[0]
        try: # Hinzugefügt am 16.09.2021
            VIP_messu['S4']=DQsecs.loc[VIP_messu['SM']:].loc[(DQsecs.DQ1/DQsecs.DQ1.max())<_opts['OPT_YM_Determination_refSecHard'][1]].index[0]-1
            # VIP_messu['F4']=DQcons.loc[VIP_messu['FM']:].loc[(DQcons.DQ1/DQcons.DQ1.max())<_opts['OPT_YM_Determination_refinement'][1]].index[0]
        except IndexError:
            VIP_messu['S4']=VIP_messu['SM']-1 #-1 könnte zu Problemen führen
        VIP_messu=VIP_messu.sort_values()
    
    
    if True:
        fig, (ax1,ax3) = plt.subplots(nrows=2, ncols=1, sharex=False, sharey=False, 
                                      figsize = np.multiply(figsize,[1,2]))
        fig.suptitle('%s - Improvement of evaluation range for Youngs Modulus'%plt_name)
        ax1.set_title('Conventional measured strain')
        ax1.set_xlabel('Strain / -')
        ax1.set_ylabel('Stress / MPa')
        ax1.plot(messu.loc[:VIP_messu[_opts['OPT_YM_Determination_refinement'][2]],'Strain'], messu.loc[:VIP_messu[_opts['OPT_YM_Determination_refinement'][2]],'Stress'], 'r.',label='$\sigma$-$\epsilon$')
        a, b=messu.loc[VIP_messu[:_opts['OPT_YM_Determination_refinement'][2]],'Strain'],messu.loc[VIP_messu[:_opts['OPT_YM_Determination_refinement'][2]],'Stress']
        j=np.int64(-1)
        ax1.plot(a, b, 'bx')
        for x in VIP_messu[:_opts['OPT_YM_Determination_refinement'][2]].index:
            j+=1
            if j%2: c=(6,-6)
            else:   c=(-6,6)
            ax1.annotate('%s' % x, xy=(a.iloc[j],b.iloc[j]), xycoords='data',
                         xytext=c, ha="center", va="center", textcoords='offset points')
        ax2=ax1.twinx()
        ax2.set_ylabel('Normalized derivatives / -')
        # ax2.plot(DQcons['Strain'], 
        #          DQcons['DQ1']/abs(DQcons['DQ1']).max(), 'b:',label='DQ1')
        ax2.plot(DQcons['Strain'], 
                 DQcons['DQ1']/DQcons['DQ1'].max(), 'b:',label='DQ1')
        ax2.plot(DQcons['Strain'], 
                 DQcons['DQ2']/abs(DQcons['DQ2']).max(), 'g:',label='DQ2')
        ax2.plot(DQcons['Strain'], 
                 DQcons['DQ3']/abs(DQcons['DQ3']).max(), 'y:',label='DQ3')
        # ax2.axvline(x=DQcons.loc[VIP_messu['FM'],'Strain'],color='gray', linestyle='-')
        # ax2.axvline(x=DQcons.loc[VIP_messu['F3'],'Strain'],color='gray', linestyle=':')
        # ax2.axvline(x=DQcons.loc[VIP_messu['F4'],'Strain'],color='gray', linestyle=':')
        ax2.axvline(x=messu.loc[VIP_messu['FM'],'Strain'],color='gray', linestyle='-')
        ax2.axvline(x=messu.loc[VIP_messu['F3'],'Strain'],color='gray', linestyle=':')
        ax2.axvline(x=messu.loc[VIP_messu['F4'],'Strain'],color='gray', linestyle=':')
        ax2.axhline(y=_opts['OPT_YM_Determination_refinement'][1],color='gray', linestyle='--')
        ax2.set_yticks([-1,0,1])
        ax2.grid(which='major',axis='y',linestyle=':')
        fig.legend(loc='lower right', ncol=4)
        
        if _opts['OPT_Determination_SecHard']:
            ax3.set_title('Second hardening measured strain')
            ax3.set_xlabel('Strain / -')
            ax3.set_ylabel('Stress / MPa')
            ax3.plot(messu.loc[VIP_messu['E']:VIP_messu[_opts['OPT_YM_Determination_refSecHard'][2]],
                        'Strain'],
                      messu.loc[VIP_messu['E']:VIP_messu[_opts['OPT_YM_Determination_refSecHard'][2]],
                                'Stress'], 'r.',label='$\sigma$-$\epsilon$')
            a, b=messu.loc[VIP_messu['E':_opts['OPT_YM_Determination_refSecHard'][2]],
                            'Strain'], messu.loc[VIP_messu['E':_opts['OPT_YM_Determination_refSecHard'][2]],'Stress']
            j=np.int64(-1)
            ax3.plot(a, b, 'bx')
            for x in VIP_messu['E':_opts['OPT_YM_Determination_refSecHard'][2]].index:
                j+=1
                if j%2: c=(6,-6)
                else:   c=(-6,6)
                ax3.annotate('%s' % x, xy=(a.iloc[j],b.iloc[j]), xycoords='data',
                              xytext=c, ha="center", va="center", textcoords='offset points')
            ax4=ax3.twinx()
            ax4.set_ylabel('Normalized derivatives / -')
            ax4.plot(DQsecs['Strain'],
                      DQsecs['DQ1']/DQsecs['DQ1'].max(), 'b:',label='DQ1')
            ax4.plot(DQsecs['Strain'],
                      DQsecs['DQ2']/abs(DQsecs['DQ2']).max(), 'g:',label='DQ2')
            ax4.plot(DQsecs['Strain'],
                      DQsecs['DQ3']/abs(DQsecs['DQ3']).max(), 'y:',label='DQ3')
            ax4.axvline(x=messu.loc[VIP_messu['SM'],'Strain'],
                        color='gray', linestyle='-')
            ax4.axvline(x=messu.loc[VIP_messu['S3'],'Strain'],
                        color='gray', linestyle=':')
            ax4.axvline(x=messu.loc[VIP_messu['S4'],'Strain'],
                        color='gray', linestyle=':')
            ax4.axhline(y=_opts['OPT_YM_Determination_refSecHard'][1],
                        color='gray', linestyle='--')
            ax4.set_yticks([-1,0,1])
            ax4.grid(which='major',axis='y',linestyle=':')
        plt_hsuf(fig,path=out_full+"-YMRange_Imp",**plt_scopt)
    
    if _opts['OPT_DIC']:
        tmp={'con F1-F2':VIP_messu['F2']-VIP_messu['F1'],
             'opt F1-F2':VIP_dicu['F2']-VIP_dicu['F1'],
             'con F3-F4':VIP_messu['F4']-VIP_messu['F3'],
             'opt F3-F4':VIP_dicu['F4']-VIP_dicu['F3']}
        log_custom("\n   Datapoints (con/opt) between F1-F2: %d/%d and F3-F4: %d/%d."
                       %(*tmp.values(),),**log_scoptf)
        for i in tmp.keys(): 
            if tmp[i] < 3: cout+='%s:%d DPs, '%(i,tmp[i])
    else:
        tmp={'con F1-F2':VIP_messu['F2']-VIP_messu['F1'],
             'con F3-F4':VIP_messu['F4']-VIP_messu['F3']}
        log_custom("\n   Datapoints (con) between F1-F2: %d and F3-F4: %d."
                       %(*tmp.values(),),**log_scoptf)
        for i in tmp.keys(): 
            if tmp[i] < 3: cout+='%s:%d DPs, '%(i,tmp[i])
            
    # =====================================================================================
    #%%% 6.4 Determine Youngs-Moduli
    log_custom("\n "+"-"*100,**log_scopt)
    log_custom("\n ### -6.4 Determine Youngs-Moduli ###",**log_scopt)
    timings.loc[6.4]=time.perf_counter()
    log_custom("\n   Timing %f: %.5f s"%(timings.index[-1],
                                       timings.iloc[-1]-timings.iloc[0]),
              **log_scopt)
    
    d_stress_mid = messu.Stress.diff()
    d_strain_mid = messu.Strain.diff()
    # d_Force = messu.Force.diff()
    
    Ind_YM_f=['F1','F2']
    Ind_YM_r=['F3','F4']
    if _opts['OPT_Determination_SecHard']:
        Ind_YM_s=['S3','S4']
    sf_eva_con = messu.loc[VIP_messu[Ind_YM_f[0]]:VIP_messu[Ind_YM_f[1]]].index
    sr_eva_con = messu.loc[VIP_messu[Ind_YM_r[0]]:VIP_messu[Ind_YM_r[1]]].index    
    if _opts['OPT_Determination_SecHard']:
        ss_eva_con = messu.loc[VIP_messu[Ind_YM_s[0]]:VIP_messu[Ind_YM_s[1]]].index

    # -------------------------------------------------------------------------------------
    #%%%% 6.4.1 Method A
    timings.loc[6.41]=time.perf_counter()
    log_custom("\n   Timing %f: %.5f s"%(timings.index[-1],
                                       timings.iloc[-1]-timings.iloc[0]),
              **log_scopt)
    
    A0Al_ser = emec.fitting.YM_eva_com_sel(
        stress_ser=d_stress_mid,
        strain_ser=d_strain_mid,
        comp=_opts['OPT_Compression'],
        name='A0Al', 
        det_opt='incremental'
        )
    E_A_df = pd.concat([A0Al_ser],axis=1)
    E_A = emec.stat_ext.pd_agg(E_A_df.loc[sr_eva_con])

    if True:
        fig, (ax1,ax2) = plt.subplots(nrows=2, ncols=1, sharex=False, sharey=False, 
                                      figsize = np.multiply(figsize,[1,2]))
        fig.suptitle('%s - Compare method A'%(plt_name))
        ax1.set_title('All Steps')
        ax1.set_xlabel('Step / -')
        ax1.set_ylabel('E / MPa')
        cc={'A0Al':'m:'}
        for k in cc:
            ax1.plot(E_A_df.loc[:VIP_messu['U']].index,
                     E_A_df.loc[:VIP_messu['U']][k],
                     cc[k], label='%s - %.2f MPa'%(k,E_A.at['mean',k]))
        ax1.axvline(x=VIP_messu['F3'], color='brown', linestyle=':')
        ax1.axvline(x=VIP_messu['F4'], color='brown', linestyle='--')
        if _opts['OPT_DIC']:
            ax1.axvline(x=VIP_dicu['F3'], color='olive', linestyle=':')
            ax1.axvline(x=VIP_dicu['F4'], color='olive', linestyle='--')
        ax1.legend()
        ax2.set_title('Improved determination range')
        ax2.set_xlabel('Step / -')
        ax2.set_ylabel('E / MPa')
        cr={'A0Al':sr_eva_con}
        for k in cc:
            ax2.plot(cr[k], E_A_df[k].loc[cr[k]], cc[k])
        ax2.axvline(x=VIP_messu['F3'], color='brown', linestyle=':')
        ax2.axvline(x=VIP_messu['F4'], color='brown', linestyle='--')
        if _opts['OPT_DIC']:
            ax2.axvline(x=VIP_dicu['F3'], color='olive', linestyle=':')
            ax2.axvline(x=VIP_dicu['F4'], color='olive', linestyle='--')
        plt_hsuf(fig,path=out_full+"-YM-Me_A",**plt_scopt)
        
    #least-square fit
    E_lsq_F_A0Al = emec.fitting.YM_eva_com_sel(
        stress_ser=messu.Stress,
        strain_ser=messu.Strain,
        comp=_opts['OPT_Compression'],
        name='E_lsq_F_A0Al', 
        det_opt='leastsq',
        **{'ind_S':VIP_messu[Ind_YM_f[0]],
           'ind_E':VIP_messu[Ind_YM_f[1]]}
        )
    E_lsq_F_A0Al = pd.Series(
        E_lsq_F_A0Al, index=['E','E_abs','Rquad','Fit_result'],
        name='E_lsq_F_A0Al'
        )
    
    E_lsq_R_A0Al = emec.fitting.YM_eva_com_sel(
        stress_ser=messu.Stress,
        strain_ser=messu.Strain,
        comp=_opts['OPT_Compression'],
        name='E_lsq_R_A0Al', 
        det_opt='leastsq',
        **{'ind_S':VIP_messu[Ind_YM_r[0]],
           'ind_E':VIP_messu[Ind_YM_r[1]]}
        )
    E_lsq_R_A0Al = pd.Series(
        E_lsq_R_A0Al, 
        index=['E','E_abs','Rquad','Fit_result'],
        name='E_lsq_R_A0Al'
        )
    
    E_lsq_A = pd.concat([E_lsq_F_A0Al, E_lsq_R_A0Al],axis=1)
    if _opts['OPT_Determination_SecHard']:
        E_lsq_S_A0Al = emec.fitting.YM_eva_com_sel(
            stress_ser=messu.Stress,
            strain_ser=messu.Strain,
            comp=_opts['OPT_Compression'],
            name='E_lsq_S_A0Al', 
            det_opt='leastsq',
            **{'ind_S':VIP_messu[Ind_YM_s[0]],
               'ind_E':VIP_messu[Ind_YM_s[1]]}
            )
        E_lsq_S_A0Al = pd.Series(
            E_lsq_S_A0Al, 
            index=['E','E_abs','Rquad','Fit_result'],
            name='E_lsq_S_A0Al'
            )
        E_lsq_A = pd.concat([E_lsq_F_A0Al, E_lsq_R_A0Al, E_lsq_S_A0Al],axis=1)

    del A0Al_ser
    del E_lsq_F_A0Al, E_lsq_R_A0Al
    if _opts['OPT_Determination_SecHard']: del E_lsq_S_A0Al
        
    #%%%% 6.4.8 Method compare
    timings.loc[6.48]=time.perf_counter()
    log_custom("\n   Timing %f: %.5f s"%(timings.index[-1],
                                       timings.iloc[-1]-timings.iloc[0]),
              **log_scopt)    

    E_lsq=E_lsq_A

    E_Methods_df = E_A_df
    E_agg_funcs = ['mean',emec.stat_ext.meanwoso,'median',
                   'std',emec.stat_ext.stdwoso,
                   emec.stat_ext.cv, emec.stat_ext.cvwoso,
                   'min','max']
    
    E_inc_F_comp = E_Methods_df.loc[sf_eva_con].agg(E_agg_funcs)
    E_inc_R_comp = E_Methods_df.loc[sr_eva_con].agg(E_agg_funcs)
    if _opts['OPT_Determination_SecHard']:
        E_inc_S_comp = E_Methods_df.loc[ss_eva_con].agg(E_agg_funcs)
        
    log_custom("\n\n  Method comaparison:", **log_scoptf)
    log_custom("\n  - least square fit", **log_scoptf)
    log_custom(log_cind('\n'+E_lsq.loc[['E','Rquad']].T.to_string()),
               **log_scoptf)
    
    log_custom("\n\n  - incremental (F,R,S):", **log_scoptf)
    log_custom(log_cind('\n'+E_inc_F_comp.T.to_string()), **log_scoptf)
    log_custom(log_cind('\n'+E_inc_R_comp.T.to_string()), **log_scoptf)
    if _opts['OPT_Determination_SecHard']:
        log_custom(log_cind('\n'+E_inc_S_comp.T.to_string()), **log_scoptf)

    # set preffered Method
    YM_pref_con=E_lsq['E_lsq_R_A0Al']
    if _opts['OPT_Determination_SecHard']: YM_pref_conS=E_lsq['E_lsq_S_A0Al']
    
    # --------------------------------------------------------------------------
    #%%% 6.5 Determine yield point
    log_custom("\n "+"-"*100, **log_scopt)
    log_custom("\n ### -6.5 Determine yield point ###", **log_scopt)
    timings.loc[6.5]=time.perf_counter()
    log_custom("\n   Timing %f: %.5f s"%(timings.index[-1],
                                       timings.iloc[-1]-timings.iloc[0]),
               **log_scopt)
    
    strain_osd = {'YK':0.0,'Y0':0.0,'Y1':0.05/100,'Y2':0.1/100,'Y':0.2/100} #acc. Zhang et al. 2021, DOI: 10.1007/s10439-020-02719-2
    strain_osdf={'YK':'F4'}

    log_custom("\n  Determination of yield strain-conventional:", **log_scoptf)
    tmp=emec.mc_yield.Yield_redet2_Multi(
        m_df=messu, VIP=VIP_messu,
        strain_osd=strain_osd, strain_osdf=strain_osdf,
        n_strain='Strain', n_stress='Stress',
        n_loBo=['F3'], n_upBo=['U'], n_loBo_int=['F3'],
        YM     = YM_pref_con['E'],
        YM_abs = YM_pref_con['E_abs'],
        use_rd =True, rise_det=[
            _opts['OPT_Rise_Smoothing'][0], 
            _opts['OPT_Rise_Smoothing'][1]
            ], 
        ywhere='n'
        )
    VIP_messu, yield_df_con, txt = tmp
    log_custom(log_cind(txt,3),  **log_scoptf)
    if _opts['OPT_Determination_SecHard']:
        log_custom("\n  Determination of yield strain-second hardening:",
                   **log_scoptf)
        strain_osdS = {'S'+key:value for (key,value) in strain_osd.items()}
        strain_osdfS = {'SYK':'S4'}        
        tmp=emec.mc_yield.Yield_redet2_Multi(
            m_df=messu, VIP=VIP_messu,
            strain_osd=strain_osdS, strain_osdf=strain_osdfS,
            n_strain='Strain', n_stress='Stress',
            n_loBo=['S3'], n_upBo=['SU'], n_loBo_int=['S3'],
            YM     = YM_pref_conS['E'],
            YM_abs = YM_pref_conS['E_abs'],
            use_rd =True, rise_det=[
                _opts['OPT_Rise_Smoothing'][0],
                _opts['OPT_Rise_Smoothing'][1]
                ], 
            ywhere='n'
            )
        VIP_messu, yield_df_conS, txt = tmp
        log_custom(log_cind(txt,3), **log_scoptf)

    #%%% 6.6 Determine final curve
    log_custom("\n "+"-"*100, **log_scopt)
    log_custom("\n ### -6.6 Determine final stress-strain curve ###",
               **log_scopt)
    timings.loc[6.6]=time.perf_counter()
    log_custom("\n   Timing %f: %.5f s"%(timings.index[-1],
                                       timings.iloc[-1]-timings.iloc[0]),
               **log_scopt)

    FinSSC = True # finale kurve für werteermittlung auf Ym linearisieren an anfang
    if FinSSC:
        messu_FP,linstrainos_con=emec.mc_man.DetFinSSC(
            mdf=messu, YM=YM_pref_con, 
            iS=VIP_messu['F3'], iLE=None,
            StressN='Stress', StrainN='Strain', 
            addzero=True, izero=VIP_messu['S']
            )
        yield_df_con['Strain']=yield_df_con['Strain']-linstrainos_con
        yield_df_con['Force']=yield_df_con['Stress']*Area
        yield_df_con['Way']=yield_df_con['Strain']*Length
        log_custom("\n   Strain offset about %.5f"%(linstrainos_con),
                    **log_scoptf)
        messu_FP['Force']=messu_FP['Stress']*Area # recalc Force (should match messu)
        messu_FP['Way']=messu_FP['Strain']*Length # recalc Way
        if _opts['OPT_Determination_SecHard']:
            yield_df_conS['Strain']=yield_df_conS['Strain']-linstrainos_con
            yield_df_conS['Force']=yield_df_conS['Stress']*Area
            yield_df_conS['Way']=yield_df_conS['Strain']*Length       
        if linstrainos_con < 0:
            cout += "Lin. strain offset = %f (%f of eps_u), "%(
                linstrainos_con, 
                linstrainos_con/messu.loc[VIP_messu['U'],'Strain'])
    else:
        messu_FP =  messu
        linstrainos_con = 0
        log_custom("\n   No linear start of final stress-strain-curve", 
                   **log_scopt)
        
    # ============================================================================
    #%% 7 Outputs
    log_custom("\n "+"="*100, **log_scopt)
    log_custom("\n ### 7 Outputs ###", **log_scoptf)
    timings.loc[7.0]=time.perf_counter()
    log_custom("\n   Timing %f: %.5f s"%(timings.index[-1],
                                       timings.iloc[-1]-timings.iloc[0]),
               **log_scopt)    
    # ============================================================================
    #%%% 7.1 Prepare outputs
    timings.loc[7.1]=time.perf_counter()
    log_custom("\n   Timing %f: %.5f s"%(timings.index[-1],
                                       timings.iloc[-1]-timings.iloc[0]),
               **log_scopt)    

    out_tab               = pd.Series([],name=prot_ser.name,dtype='float64')
    out_tab['Date_eva']   = datetime.now().strftime('%d.%m.%Y')
    out_tab['leos_con']   = linstrainos_con      
    
    relVS = [*np.sort([*strain_osd.keys()]),'U','B','E']
    tmp=emec.output.Otvalgetter_Multi(
        messu_FP, Vs=relVS, datasep=['con'], VIPs={'con':VIP_messu}, 
        exacts={'con':yield_df_con}, orders={'con':['f','e','U']}, 
        add_esufs={'con':'_con'}, 
        n_strains={'con':'Strain'}, n_stresss={'con':'Stress'},
        use_exacts=True
        )
    tmp.name=prot_ser.name
    out_tab=out_tab.append(tmp)
    tmp=emec.output.Otvalgetter_Multi(
        messu_FP, Vs=relVS, datasep=['con'], VIPs={'con':VIP_messu}, 
        exacts={'con':yield_df_con}, orders={'con':['F','s','W']}, 
        add_esufs={'con':'_con'},
        n_strains={'con':'Way'}, n_stresss={'con':'Force'},
        use_exacts=True
        )
    tmp.name=prot_ser.name
    out_tab=out_tab.append(tmp)
    if _opts['OPT_Determination_SecHard']:
        relVS = [*np.sort([*strain_osdS.keys()]),'SU']
        tmp=emec.output.Otvalgetter_Multi(
            messu_FP, Vs=relVS, datasep=['con'], VIPs={'con':VIP_messu}, 
            exacts={'con':yield_df_conS}, orders={'con':['f','e','U']}, 
            add_esufs={'con':'_con'},
            n_strains={'con':'Strain'}, n_stresss={'con':'Stress'},
            use_exacts=True
            )
        tmp.name=prot_ser.name
        out_tab=out_tab.append(tmp)
        tmp=emec.output.Otvalgetter_Multi(
            messu_FP, Vs=relVS, datasep=['con'], VIPs={'con':VIP_messu}, 
            exacts={'con':yield_df_conS}, orders={'con':['F','s','W']}, 
            add_esufs={'con':'_con'},
            n_strains={'con':'Way'}, n_stresss={'con':'Force'},
            use_exacts=True
            )
        tmp.name=prot_ser.name
        out_tab=out_tab.append(tmp)
    # ============================================================================
    #%%% 7.2 Generate plots
    timings.loc[7.2]=time.perf_counter()
    log_custom("\n   Timing %f: %.5f s"%(timings.index[-1],
                                       timings.iloc[-1]-timings.iloc[0]),
               **log_scopt)    
        
    fig, ax1 = plt.subplots()
    ax1.set_title('%s - Analyzing meas. force'%plt_name)
    color1 = 'tab:red'
    ax1.set_xlabel('Time / s')
    ax1.set_ylabel('Force / N', color=color1)
    ax1.plot(messu.Time, messu.Force, 'r-', label='Force')
    a, b=messu.Time[VIP_messu],messu.Force[VIP_messu]
    j=np.int64(-1)
    ax1.plot(a, b, 'bx')
    for x in VIP_messu.index:
        j+=1
        if j%2: c=(6,-6)
        else:   c=(-6,6)
        ax1.annotate('%s' % x, xy=(a.iloc[j],b.iloc[j]), xycoords='data',
                     xytext=c, ha="center", va="center", textcoords='offset points')
    ax1.tick_params(axis='y', labelcolor=color1)
    ax2 = ax1.twinx()  # instantiate a second axes that shares the same x-axis
    color2 = 'tab:blue'
    ax2.set_ylabel('Rise / curve /  (N/s)', color=color2)
    ax2.plot(messu.Time, messu.driF, 'b:', label='Force-rise')
    ax2.plot(messu.Time, messu.dcuF, 'g:', label='Force-curve')
    ax2.tick_params(axis='y', labelcolor=color2)
    fig.legend(loc='lower right', bbox_to_anchor=(0.85, 0.15))
    plt_hsuf(fig,path=out_full+"-Fdricu",**plt_scopt)
    
    fig, ax1 = plt.subplots()
    ax1.set_title('%s - Stress vs. strain curve with labels'%plt_name)
    ax1.set_xlabel('Strain / -')
    ax1.set_ylabel('Stress / MPa')
    if _opts['OPT_Determination_SecHard']:
        ind_E='SE'
    else:
        ind_E='E'
    ax1.plot(messu.loc[:VIP_messu[ind_E]]['Strain'],
             messu.loc[:VIP_messu[ind_E]]['Stress'], 'r--',label='con')
    if _opts['OPT_DIC']:
        ax1.plot(messu.loc[:VIP_messu[ind_E]][dic_used_Strain],
                 messu.loc[:VIP_messu[ind_E]]['Stress'], 'm--',label='opt')
    a,b = emec.fitting.stress_linfit_plt(
        messu['Strain'], VIP_messu[['F3','F4']], *YM_pref_con[['E','E_abs']]
        )
    ax1.plot(a, b, 'g-',label='$E_{con}$')
    if _opts['OPT_Determination_SecHard']:
        a,b = emec.fitting.stress_linfit_plt(
            messu['Strain'], VIP_messu[['S3','S4']], *YM_pref_conS[['E','E_abs']]
            )
        ax1.plot(a, b, 'm-',label='$E_{sec}$')
    a, b=messu.Strain[VIP_messu[:ind_E]],messu.Stress[VIP_messu[:ind_E]]
    j=np.int64(-1)
    ax1.plot(a, b, 'bx')
    for x in VIP_messu[:ind_E].index:
        j+=1
        if j%2: c=(6,-6)
        else:   c=(-6,6)
        ax1.annotate('%s' % x, xy=(a.iloc[j],b.iloc[j]), xycoords='data',
                      xytext=c, ha="center", va="center", textcoords='offset points')
    if _opts['OPT_DIC']:
        a,b=messu.loc[VIP_dicu[:'SE'],dic_used_Strain],messu.Stress[VIP_dicu[:'SE']]
        j=np.int64(-1)
        ax1.plot(a, b, 'yx')
        a,b = emec.fitting.stress_linfit_plt(
            messu[dic_used_Strain], VIP_dicu[['F3','F4']],
            *E_lsq_A[loc_Yd_tmp][['E','E_abs']]
            )
        ax1.plot(a, b, 'b-',label='$E_{opt}$')
    ftxt=('$f_{y}$ = % 6.3f MPa ($\epsilon_{y}$ = %4.3f %%)'%(out_tab['fy'],out_tab['ey_con']*100),
          '$f_{u}$ = % 6.3f MPa ($\epsilon_{u}$ = %4.3f %%)'%(out_tab['fu'],out_tab['eu_con']*100),
          '$E_{con}$ = % 8.3f MPa ($R²$ = %4.3f)'%(*YM_pref_con[['E','Rquad']],))
    
    if _opts['OPT_Determination_SecHard']:
          ftxt+=('$E_{sec}$ = % 8.3f MPa ($R²$ = %4.3f)'%(*YM_pref_conS[['E','Rquad']],),)
    fig.text(0.95,0.15,'\n'.join(ftxt),
              ha='right',va='bottom', bbox=dict(boxstyle='round', edgecolor='0.8', facecolor='white', alpha=0.8))
    fig.legend(loc='upper left', bbox_to_anchor=(0.10, 0.91))
    plt_hsuf(fig,path=out_full+"-sigeps_wl",**plt_scopt)

    fig, ax1 = plt.subplots()
    ax1.set_title('%s - Stress vs. strain curve with labels (to 1st min.)'%plt_name)
    ax1.set_xlabel('Strain / -')
    ax1.set_ylabel('Stress / MPa')
    ax1.plot(messu.loc[:VIP_messu['E'],'Strain'],
             messu.loc[:VIP_messu['E'],'Stress'], 'r--',label='con')
    a,b = emec.fitting.stress_linfit_plt(
        messu['Strain'], VIP_messu[['F1','F2']],
        *E_lsq_A['E_lsq_F_A0Al'][['E','E_abs']]
        )
    ax1.plot(a, b, 'b-',label='$E_{con,F}$')
    a,b = emec.fitting.stress_linfit_plt(
        messu['Strain'], VIP_messu[['F3','F4']],
        *YM_pref_con[['E','E_abs']]
        )
    ax1.plot(a, b, 'g-',label='$E_{con,R}$')
    a, b=messu.Strain[VIP_messu[:'E']],messu.Stress[VIP_messu[:'E']]
    j=np.int64(-1)
    ax1.plot(a, b, 'bx')
    for x in VIP_messu[:'E'].index:
        j+=1
        if j%2: c=(6,-6)
        else:   c=(-6,6)
        ax1.annotate('%s' % x, xy=(a.iloc[j],b.iloc[j]), xycoords='data',
                     xytext=c, ha="center", va="center", textcoords='offset points')
    ftxt=('$f_{y}$ = %3.3f MPa ($\epsilon_{y}$ = %.3f %%)'%(out_tab['fy'],out_tab['ey_con']*100),
         '$f_{u}$ = %3.3f MPa ($\epsilon_{u}$ = %.3f %%)'%(out_tab['fu'],out_tab['eu_con']*100),
         '$E_{con}$ = % 8.3f MPa ($R²$ = %4.3f)'%(*YM_pref_con[['E','Rquad']],))
    fig.text(0.95,0.15,'\n'.join(ftxt),
             ha='right',va='bottom', bbox=dict(boxstyle='round', edgecolor='0.8', facecolor='white', alpha=0.8))
    fig.legend(loc='upper left', bbox_to_anchor=(0.10, 0.91))
    plt_hsuf(fig,path=out_full+"-sigeps_wl1m",**plt_scopt)
    
    fig, ax1 = plt.subplots()
    ax1.set_title('%s - Stress vs. strain curve, final part, with labels'%plt_name)
    ax1.set_xlabel('Strain [-]')
    ax1.set_ylabel('Stress [MPa]')
    ax1.plot(messu_FP['Strain'], messu_FP['Stress'], 'r--', label='meas. curve')
    a,b = emec.fitting.stress_linfit_plt(
        messu_FP['Strain'], VIP_messu[['F3','F4']],
        YM_pref_con['E'],0
        )
    ax1.plot(a, b, 'g-',label='$E_{con}$')
    tmp=VIP_messu[['S','F3','F4','Y','Y0','Y1','U']].sort_values()
    if 'B' in VIP_messu.index: tmp=tmp.append(VIP_messu[['B']])
    tmp=tmp.append(VIP_messu[['E']])
    if _opts['OPT_Determination_SecHard']: 
        tmp=tmp.append(VIP_messu[['S3','S4','SY','SY0','SY1','SU','SE']])
    tmp=tmp.sort_values()
    a, b=messu_FP.Strain[tmp],messu_FP.Stress[tmp]
    j=np.int64(-1)
    ax1.plot(a, b, 'bx')
    for x in tmp.index:
        j+=1
        if j%2: c=(6,-6)
        else:   c=(-6,6)
        ax1.annotate('%s' % x, xy=(a.iloc[j],b.iloc[j]), xycoords='data', 
                     xytext=c, ha="center", va="center", textcoords='offset points')
    ax1.legend()
    plt_hsuf(fig,path=out_full+"-sigeps_fin",**plt_scopt)
    
    # =============================================================================
    #%%% 7.3 Generate outputs
    timings.loc[7.3]=time.perf_counter()
    log_custom("\n   Timing %f: %.5f s"%(timings.index[-1],
                                       timings.iloc[-1]-timings.iloc[0]),
               **log_scopt)    
    
    
    t=E_lsq.loc[['E','Rquad']].T.stack()
    t.index=t.index.to_series().apply(lambda x: '{0}_{1}'.format(*x)).values
    t.name=prot_ser.name
    out_tab=pd.concat([out_tab,t])
    
    t=E_inc_F_comp.T.stack()
    t.index=t.index.to_series().apply(lambda x: '{0}_{1}'.format(*x)).values
    t.name=prot_ser.name
    t=t.add_prefix('E_inc_F_')
    out_tab=pd.concat([out_tab,t])

    t=E_inc_R_comp.T.stack()
    t.index=t.index.to_series().apply(lambda x: '{0}_{1}'.format(*x)).values
    t.name=prot_ser.name
    t=t.add_prefix('E_inc_R_')
    out_tab=pd.concat([out_tab,t])
    
    if _opts['OPT_Determination_SecHard']:
        t=E_inc_S_comp.T.stack()
        t.index=t.index.to_series().apply(lambda x: '{0}_{1}'.format(*x)).values
        t.name=prot_ser.name
        t=t.add_prefix('E_inc_S_')
        out_tab=pd.concat([out_tab,t])
    
    out_tab=pd.DataFrame([out_tab])

    vnew=pd.Series(VIP_messu.drop_duplicates().index.values,
                   index=VIP_messu.drop_duplicates(),dtype='O')
    VIP_messu=VIP_messu.sort_values()
    for i in VIP_messu.index:
        if VIP_messu.duplicated().loc[i]:
            vnew.loc[VIP_messu.loc[i]]=vnew.loc[VIP_messu.loc[i]]+','+i
    messu['VIP_m']=vnew
    messu.VIP_m.fillna('', inplace=True)
    
    if _opts['OPT_DIC']:
        vnew=pd.Series(VIP_dicu.drop_duplicates().index.values,
                       index=VIP_dicu.drop_duplicates(),dtype='O')
        VIP_dicu=VIP_dicu.sort_values()
        for i in VIP_dicu.index:
            if VIP_dicu.duplicated().loc[i]:
                vnew.loc[VIP_dicu.loc[i]]=vnew.loc[VIP_dicu.loc[i]]+','+i
        messu['VIP_d']=vnew
        messu.VIP_d.fillna('', inplace=True)
    
    messu.to_csv(out_full+'.csv',sep=';')   
    out_tab.to_csv(out_full+'-Mat.csv',sep=';')
    
    HDFst=pd.HDFStore(out_full+'.h5')
    HDFst['Measurement'] = messu
    HDFst['Measurement_FP'] = messu_FP
    HDFst['Material_Parameters'] = out_tab
    HDFst['Timings'] = timings
    HDFst['Options'] = _opts
    if _opts['OPT_DIC']:
        HDFst['VIP'] = pd.concat([VIP_messu,VIP_dicu],axis=1)
    else:
        HDFst['VIP'] = pd.concat([VIP_messu],axis=1)
    
    HDFst['DQcon'] = DQcon
    if _opts['OPT_Determination_SecHard']:
        HDFst['DQsec'] = DQsec
    
    HDFst['E_lsq'] = E_lsq
    HDFst['E_inc_df'] = E_Methods_df
    if _opts['OPT_Determination_SecHard']:
        HDFst['E_inc'] = pd.concat([E_inc_F_comp.add_prefix('E_inc_F_'),
                                    E_inc_R_comp.add_prefix('E_inc_R_'),
                                    E_inc_R_comp.add_prefix('E_inc_S_')],axis=1)
        HDFst['Exact_VIP_con']=pd.concat([yield_df_con,yield_df_conS])
    else:
        HDFst['E_inc'] = pd.concat([E_inc_F_comp.add_prefix('E_inc_F_'),
                                    E_inc_R_comp.add_prefix('E_inc_R_')],axis=1)
        HDFst['Exact_VIP_con']=yield_df_con
    
    HDFst.close()
    
    timings.loc[10.0]=time.perf_counter()
    if log_scopt['output_lvl']>=1: 
        log_scopt['logfp'].close()
        log_scopt['logfp'] = None # workaround (Error _io.TextIOWrapper while series run)
    
    return timings, cout
```

---

© Copyright 2024, MarcGebhardt.

Built with Sphinx using a
theme
provided by Read the Docs.
