# Supplementary material for: Approach to Standardized Material Characterization of the Human Lumbopelvic System: Testing and Evaluation
Source: Bioengineering (Basel). 2025 Aug 11;12(8):862. doi: 10.3390/bioengineering12080862 (PMC12383908; doi:10.3390/bioengineering12080862)
Supplement: Supplementary file 1 [file bioengineering-12-00862-s001.zip › File S3 Evaluation code/ExMechEva-0.1.2/docs/_build/html/_modules/exmecheva/Eva_ATT.html]

exmecheva.Eva\_ATT — ExMechEva v0.1.1 documentation


ExMechEva

Contents:

- ExMechEva

ExMechEva

- Module code
- exmecheva.Eva\_ATT

---

# Source code for exmecheva.Eva\_ATT

```
# -*- coding: utf-8 -*-
"""
Axial tensile test evaluation.

@author: MarcGebhardt

ToDo:
    - reimplement optical measurement usage ("OPT_DIC")
"""

#%% 0 Imports
import pandas as pd
import numpy as np
import matplotlib.pyplot as plt
from datetime import datetime
import time
import warnings

import exmecheva.common as emec

warnings.filterwarnings('ignore',category=pd.io.pytables.PerformanceWarning)
warnings.filterwarnings('ignore',category=FutureWarning)

log_custom = emec.output.str_log
log_cind = emec.output.str_indent

plt_hsuf =  emec.plotting.plt_handle_suffix
figsize = plt.rcParams['figure.figsize']

#%% 1.0 Evaluation

[docs]def ATT_single(prot_ser, paths, mfile_add='',
               log_scopt={'logfp':None, 'output_lvl': 1,
                          'logopt':True, 'printopt':False},
               plt_scopt={'tight':True, 'show':True, 
                          'save':True, 's_types':["pdf"], 
                          'clear':True, 'close':True}):
    """
    Evaluate single axial tensile test measurement form protocol table and 
    path collection. Using common options wich are overwritten by protocol 
    variables starting with 'OPT_'. Produce evaluated measurements, 
    material parameters and plots and safe them as table (.csv), unstructured 
    database (.h5) and document (.pdf), using the designation of the specimen 
    for distinction.
    Procedure:
        - 1: Read in options and presetting
        - 2: Determining geometrical values
        - 3: Read in measurements (conventional and optical (optinal))
        - 4: Merging measurements (time offset of conventional to optical 
                                   (if available), downsampling and merging)
        - 5: Determine evaluation space (start and end)
        - 6: Evaluation (curves (stress/strain), importent points on curves,
                         elastic moduli (different types implemented))
        - 7: Generating output (tables, database and plots)

    Parameters
    ----------
    prot_ser : pd.Series
        Input data as pandas series with specimen information 
        (identifier, origin, geometrical data, 
         assessment codes, evaluation options).
    paths : pd.DataFrame
        Path collection for in- and output paths. 
        Needs indexes: 
            - "opts": Common evaluation options
            - "prot": Protocol
            - "meas": Conventional measured data
            - "dic": Optical measured data
            - "out": Output
    mfile_add : string, optional
        Suffix of variants of measurements 
        (p.E. diffferent moistures ["A","B",...]). 
        The default is ''.
    log_scopt : dict, optional
        Options for custom logging. Determining file path, 
        output level (0=none, 1=normal, 2=special), logging enabled and 
        printing enabled.
        The default is {'logfp':None, 'output_lvl': 1,
                        'logopt':True, 'printopt':False}.
    plt_scopt : dict, optional
        Options for plotting. 
        The default is {'tight':True, 'show':True, 
                        'save':True, 's_types':["pdf"],
                        'clear':True, 'close':True}.

    Raises
    ------
    ValueError
        Input value not correct.
    NotImplementedError
        Method not implemented.

    Yields
    ------
    timings : pd.Series
        Timings of procedure steps.
    cout : string
        Special text output for control purposes.

    """
    out_name = prot_ser['Designation']+mfile_add
    out_full = paths['out']+out_name
    if log_scopt['output_lvl']>=1: 
        if log_scopt['logfp'] is None or (not isinstance(log_scopt['logfp'],str)):
            log_scopt['logfp'] = out_full+'.log'
        log_scopt['logfp']=open(log_scopt['logfp'],'w')
    log_scoptf={'logfp':log_scopt['logfp'], 
                'output_lvl': log_scopt['output_lvl'], 
                'logopt':log_scopt['logopt'], 
                'printopt':True}
        
    _opts=emec.eva_opt_hand.option_reader_sel(
        prot_ser=prot_ser, paths=paths, 
        search_inds=['Number','Designation','name'], 
        variant='',
        option='JFile+Prot',
        sheet_name="",
        re_rkws=dict()
        )
    
    path_meas = paths['meas']+_opts['OPT_File_Meas']+mfile_add+".xlsx"
    path_dic = paths['dic']+_opts['OPT_File_DIC']+mfile_add+".csv"
    
    plt_name = prot_ser['Designation']+mfile_add
    timings=pd.Series([],dtype='float64')
    timings.loc[0.0]=time.perf_counter()
    
    # rel_time_digs = Evac.sigdig(_opts['OPT_Resampling_Frequency'], 4)
    rel_time_digs = 2
    
           
    dic_used_Strain="Strain_"+_opts['OPT_YM_Determination_refinement'][3]
    tmp_in = _opts['OPT_YM_Determination_refinement'][3].split('_')[-1]
    dic_used_Disp="Disp_opt_"+tmp_in
    
    if _opts['OPT_YM_Determination_refinement'][3].split('_')[-2] == 'con':
        tmp_md = '0'
    elif _opts['OPT_YM_Determination_refinement'][3].split('_')[-2] == 'opt':
        tmp_md = '2'
    else:
        raise ValueError('OPT_YM_Determination_refinement seams to be wrong')
    loc_Yd_tmp = 'E_lsq_R_A%s%sl'%(tmp_md,tmp_in)
        
    cout =''
    ftxt=(("  Parameters of evaluation:"),
          ("   Evaluation start time:     %s" %datetime.now().strftime('%Y-%m-%d %H:%M:%S')),
          ("   Evaluation options:        %s" %paths['opts']),
          ("   Path protocol:             %s" %paths['prot']),
          ("   Path measurement:          %s" %path_meas),
          ("   Path optical- measurement: %s" %path_dic),
          ("   Resampling = %s (frequency = %d Hz, moving-average = %s)" %(
              _opts['OPT_Resampling'],_opts['OPT_Resampling_Frequency'],
              _opts['OPT_Resampling_moveave']
              )),
          ("   Compression = %s" %(_opts['OPT_Compression'])),
          ("   LVDT failure = %s" %(_opts['OPT_LVDT_failure'])),
          ("   LVDT-spring-force-reduction = %s (K_Fed = %f N/mm)" %(
              _opts['OPT_Springreduction'],_opts['OPT_Springreduction_K']
              )),
          ("   Rise and curvature smoothing = %s (window = %d)" %(
              _opts['OPT_Rise_Smoothing'][0],_opts['OPT_Rise_Smoothing'][1]
              )),
          ("   Youngs Modulus determination between %f and %f of point %s" %(
              *_opts['OPT_YM_Determination_range'],
              )),
          ("   Distance between points: %d / %d steps " %(
              *_opts['OPT_Determination_Distance'],
              )),
          ("   Improvment of Youngs-Modulus-determination between %s*Stress_max and point %s," %(
              _opts['OPT_YM_Determination_refinement'][0],
              _opts['OPT_YM_Determination_refinement'][2]
              )),
          ("    with smoothing on difference-quotient (%s, %d)" %(
              _opts['OPT_YM_Determination_refinement'][4],
              _opts['OPT_YM_Determination_refinement'][5]
              )),
          ("    with allowable deviation of %f * difference-quotient_max in determination range" %(
              _opts['OPT_YM_Determination_refinement'][1]
              )),
          ("   DIC-Measurement = %s" %(_opts['OPT_DIC'])),
          ("   DIC-Strain-suffix for range refinement and plots = %s" %(
              _opts['OPT_YM_Determination_refinement'][3]
              )))
          # ("   DIC-minimal points (special / specimen) = %d / %d" %(_opts['OPT_DIC_Tester'][-2],_opts['OPT_DIC_Tester'][-1])),
          # ("   DIC-names of special points (l,r,head), = %s, %s, %s" %(*_opts['OPT_DIC_Points_TBT_device'],)),
          # ("   DIC-names of meas. points for fork (l,m,r), = %s, %s, %s" %(*_opts['OPT_DIC_Points_meas_fork'],)),
          # ("   DIC-maximal SD = %.3f mm and maximal displacement between steps %.1f mm" %(_opts['OPT_DIC_Tester'][0],_opts['OPT_DIC_Tester'][1])))
    log_custom('\n'.join(ftxt), **log_scopt)
    # =============================================================================
    
    #%% 2 Geometry
    log_custom("\n "+"="*100, **log_scopt)
    log_custom("\n ### 2 Geometry ###", **log_scoptf)
    
    if prot_ser['Test_Shape'] == 'Belt':
        if prot_ser['CS_type'] == 'Rectangle':
            thickness_mean = prot_ser[['thickness_1','thickness_2']].mean()
            width_mean     = prot_ser[['width_1','width_2']].mean()
            Area   = thickness_mean * width_mean
            Volume = Area * prot_ser['Length']
        else:
            raise NotImplementedError("Cross section type %s not implemented"%prot_ser['CS_type'])
    elif prot_ser['Test_Shape'] == 'Dogbone':
        raise NotImplementedError("Test shape %s not implemented"%prot_ser['Test_Shape'])
    else:
        raise NotImplementedError("Test shape %s not implemented"%prot_ser['Test_Shape'])
    
    log_custom("\n    Area   det=IN: %s (%.3f-%.3f)"%(Area==prot_ser['Area_CS'],
                                                      Area,prot_ser['Area_CS']),
               **log_scoptf)    
    log_custom("\n    Volume det=IN: %s (%.3f-%.3f)"%(Volume/1000==prot_ser['Volume'],
                                                      Volume/1000,prot_ser['Volume']),
               **log_scoptf)
    # reset to protocoll values
    Length = prot_ser['Length_test']
    Area   = prot_ser['Area_CS']
    Volume = prot_ser['Volume']
    
    # =============================================================================
    
    #%% 3 Read in measurements
    log_custom("\n "+"="*100, **log_scopt)
    log_custom("\n ### 3 Read in measurements ###", **log_scoptf)
    timings.loc[3.0]=time.perf_counter()
    log_custom("\n   Timing %f: %.5f s"%(timings.index[-1],
                                       timings.iloc[-1]-timings.iloc[0]),
               **log_scopt)
    # =============================================================================
    
    #%%% 3.1 Read in conventional measurement data
    timings.loc[3.1]=time.perf_counter()
    log_custom("\n   Timing %f: %.5f s"%(timings.index[-1],
                                       timings.iloc[-1]-timings.iloc[0]),
               **log_scopt)
    mess=pd.read_excel(path_meas, header=_opts['OPT_Measurement_file']['header'],
                       names=_opts['OPT_Measurement_file']['head_names'])
    
    mess = mess-mess.iloc[0]
    
    if isinstance(_opts['OPT_Measurement_file']['used_names_dict']['Way'],type(list())):
        mess['L_IWA']=pd.Series(
            mess[_opts['OPT_Measurement_file']['used_names_dict']['Way']].mean(axis=1)
            )
        # automatic number of LVDTs by list elements in used way
        n_IWAs=len(_opts['OPT_Measurement_file']['used_names_dict']['Way'])
        _opts['OPT_Measurement_file']['used_names_dict']['Way'] ='L_IWA'
    # Additional option for easy switching of force measurement type 
    if ('OPT_Force_measure_type' in _opts.index):
        if not emec.helper.check_empty(_opts['OPT_Force_measure_type']):
            _opts['OPT_Measurement_file']['used_names_dict']['Force'] =(
                'F_'+_opts['OPT_Force_measure_type']
                )
    # Applying spring correction factor (if true), since displacement of LVDTs
    # is defined positive by sliding together, Springreduction_K have to be negative.
    # The resulting correction force is then positive.
    if _opts['OPT_Springreduction']:
        mess['F_IWA_red']=mess['L_IWA']*_opts['OPT_Springreduction_K']*n_IWAs
        
    # # =============================================================================
    # #%%% 3.2 Specify used conventional measured force and way
    timings.loc[3.2]=time.perf_counter()
    log_custom("\n   Timing %f: %.5f s"%(timings.index[-1],
                                       timings.iloc[-1]-timings.iloc[0]),
               **log_scopt)
    messu = pd.DataFrame({
        'Time': mess[_opts['OPT_Measurement_file']['used_names_dict']['Time']],
        'Force': mess[_opts['OPT_Measurement_file']['used_names_dict']['Force']],
        'Way': mess[_opts['OPT_Measurement_file']['used_names_dict']['Way']]
        })

    # The setup is can be seen as parallel connected springs
    # n of them are LVDTs, with a maximum from spring stiffness at the start of the test
    # if the sample is stretched, this force (from spring stiffness) is reduced
    # this force have to be substracted from the measured force
    if _opts['OPT_Springreduction']:     
        messu['Force'] = messu['Force'] - mess['F_IWA_red']
    if _opts['OPT_LVDT_failure'][0]:     
        messu['Way'] = _opts['OPT_LVDT_failure'][1]*mess[_opts['OPT_LVDT_failure'][2]]
        
    if _opts['OPT_Compression']==True:
        messu.Force=messu.Force*(-1)
    else:
        messu.Way=messu.Way*(-1) # normal definition of LVDT is compression positive
    if np.invert(np.isnan(_opts['OPT_End'])):
        messu=messu.loc[messu.Time.round(rel_time_digs) <= _opts['OPT_End']]
    
    # =============================================================================
    #%%% 3.3 Read in optical measurement data
    timings.loc[3.3]=time.perf_counter()
    log_custom("\n   Timing %f: %.5f s"%(timings.index[-1],
                                       timings.iloc[-1]-timings.iloc[0]),
               **log_scopt)
    if _opts['OPT_DIC']: #optical evaluation removed
        dic=None
        dicu=None
        dic_dt=None
        step_range_dic=None
        raise NotImplementedError('DIC not implemented')
        
    # =============================================================================
    #%% 4 Merging measurements
    log_custom("\n "+"="*100,**log_scopt)
    log_custom("\n ### 4 Merging measurements ###",**log_scoptf)
    timings.loc[4.0]=time.perf_counter()
    log_custom("\n   Timing %f: %.5f s"%(timings.index[-1],
                                       timings.iloc[-1]-timings.iloc[0]),
               **log_scopt)
    # =============================================================================
    #%%% 4.1 Determine time offset between conventional and optical measurement
    timings.loc[4.1]=time.perf_counter()
    log_custom("\n   Timing %f: %.5f s"%(timings.index[-1],
                                       timings.iloc[-1]-timings.iloc[0]),
               **log_scopt)
    if _opts['OPT_DIC']:
        if _opts['OPT_pre_load_cycles'] > 0:
            ri_Way,_,rich_Way=emec.mc_char.rise_curve(
                messu['Way'],
                _opts['OPT_Rise_Smoothing'][0], _opts['OPT_Rise_Smoothing'][1]
                )
            ws = _opts['OPT_Determination_Distance'][0]
            we = rich_Way[(rich_Way == True) and (rich_Way.index >= _opts['OPT_Determination_Distance'][0])].index[0]
            mun_tmp = messu.loc[
                abs(messu.Way.loc[ws:we]-messu.Way.loc[ws:we].max()/8).idxmin():
                abs(messu.Way.loc[ws:we]-messu.Way.loc[ws:we].max()/4).idxmin()
                ]
            del ri_Way, rich_Way, ws, we
            print('Check pre load cycles implementation in time offset!')
        else:
            mun_tmp = messu.loc[
                abs(messu.Way.loc[:(messu.Way.idxmax())]-messu.Way.max()/8).idxmin():
                abs(messu.Way.loc[:(messu.Way.idxmax())]-messu.Way.max()/4).idxmin()
                ]
        linvm   = np.polyfit(mun_tmp.loc[:,'Time'],mun_tmp.loc[:,'Way'],1)
        tsm     = -linvm[1]/linvm[0]
        mun_tmp = dicu.loc[
            abs(dicu.Disp_opt_head.loc[:(dicu.Disp_opt_head.idxmax())]-dicu.Disp_opt_head.max()/8).idxmin():
            abs(dicu.Disp_opt_head.loc[:(dicu.Disp_opt_head.idxmax())]-dicu.Disp_opt_head.max()/4).idxmin()
            ]
        linvd   = np.polyfit(mun_tmp.loc[:,'Time'],mun_tmp.loc[:,'Disp_opt_head'],1)
        tsd     = -linvd[1]/linvd[0]
        toff    = tsm-tsd
        
        if True:
            maxt_tmp=max(
                messu.Time.loc[abs(messu.Way.loc[:(messu.Way.idxmax())]-messu.Way.max()/4).idxmin()],
                dicu.Time.loc[abs(dicu.Disp_opt_head.loc[:(dicu.Disp_opt_head.idxmax())]-dicu.Disp_opt_head.max()/4).idxmin()]
                )
            xlin_tmp=np.linspace(min(tsm,tsd),maxt_tmp,11)
            
            fig, ax1 = plt.subplots()
            ax1.set_title('%s - Way-measuring time difference'%plt_name)
            ax1.set_xlabel('Time / s')
            ax1.set_ylabel('Way / mm')
            ax1.plot(messu.Time.loc[messu.Time<=maxt_tmp], 
                     messu.Way.loc[messu.Time<=maxt_tmp],
                     'r-', label='PM-way')
            ax1.plot(dicu.Time.loc[dicu.Time<=maxt_tmp],
                     dicu.Disp_opt_head.loc[dicu.Time<=maxt_tmp], 
                     'b-', label='DIC-way')
            ax1.plot(xlin_tmp, np.polyval(linvm,xlin_tmp),
                     'y:',label='PM-lin')
            ax1.plot(xlin_tmp, np.polyval(linvd,xlin_tmp),
                     'g:',label='DIC-lin')
            ax1.axvline(x=tsm, color='red', 
                        linestyle=':', label='PM-way-start')
            ax1.axvline(x=tsd, color='blue', 
                        linestyle=':', label='DIC-way-start')
            ax1.legend()
            ftxt=('$t_{S,PM}$  = % 2.4f s '%(tsm),
                  '$t_{S,DIC}$ = % 2.4f s '%(tsd))
            fig.text(0.95,0.15,'\n'.join(ftxt),
                     ha='right',va='bottom', 
                     bbox=dict(boxstyle='round', edgecolor='0.8', 
                               facecolor='white', alpha=0.8))
            plt_hsuf(fig,path=out_full+"-toff",**plt_scopt)
            del xlin_tmp
        
        log_custom("\n "+"-"*100,**log_scopt)
        log_custom("\n   Time offset between PM and DIC: %.3f s" %(toff),**log_scoptf)
    else:
        toff=0.0
        
    messu.Time=(messu.Time-toff).round(rel_time_digs)
    
    # =============================================================================
    #%%% 4.2 Downsampling of conventional data
    timings.loc[4.2]=time.perf_counter()
    log_custom("\n   Timing %f: %.5f s"%(timings.index[-1],
                                       timings.iloc[-1]-timings.iloc[0]),
               **log_scopt)
    
    messu, _, mess_f = emec.mc_man.mc_resampler(
        mdf=messu, 
        t_col=_opts['OPT_Measurement_file']['used_names_dict']['Time'], 
        resample=_opts['OPT_Resampling'], 
        res_frequ=_opts['OPT_Resampling_Frequency'], 
        move_ave=_opts['OPT_Resampling_moveave'], 
        ma_sampler='data_rf',
        rel_time_digs=rel_time_digs) 
    
    # =============================================================================
    #%%% 4.3 Merge optical to conventional data
    timings.loc[4.3]=time.perf_counter()
    log_custom("\n   Timing %f: %.5f s"%(timings.index[-1],
                                       timings.iloc[-1]-timings.iloc[0]),
               **log_scopt)
    if _opts['OPT_DIC']:
        dicu.Time=dicu.Time.round(rel_time_digs)
        ind=pd.RangeIndex(dicu.loc[dicu.Time>=messu.Time.min()].index[0],
                          messu.Time.count()+dicu.loc[dicu.Time>=messu.Time.min()].index[0],1)
        messu=messu.merge(dicu,how='left', on='Time').set_index(ind)
        
    else:
        # dic_f=round(1/dic_dt)
        dic_f=mess_f
        
    f_vdm=dic_f/mess_f
        
    # =============================================================================
    #%% 5 Start and End
    log_custom("\n "+"="*100,**log_scopt)
    log_custom("\n ### 5 Start and End ###",**log_scoptf)
    timings.loc[5.0]=time.perf_counter()
    log_custom("\n   Timing %f: %.5f s"%(timings.index[-1],
                                       timings.iloc[-1]-timings.iloc[0]),
               **log_scopt)                                                    
    # =============================================================================
    #%%% 5.1 Determine start and end of evaluation
    timings.loc[5.1]=time.perf_counter()
    log_custom("\n   Timing %f: %.5f s"%(timings.index[-1],
                                       timings.iloc[-1]-timings.iloc[0]),
               **log_scopt)
    if np.isnan(_opts['OPT_End']):
        dic_to_mess_End=messu.iloc[-1].name
    else:
        dic_to_mess_End=messu.loc[messu.Time<=_opts['OPT_End']].index[-1]
        
    mun_tmp=messu.loc[:min(dic_to_mess_End,messu.Force.idxmax()),'Force']
    messu['driF'],messu['dcuF'],messu['driF_schg']=emec.mc_char.rise_curve(
        messu.loc[:dic_to_mess_End]['Force'],
        _opts['OPT_Rise_Smoothing'][0], _opts['OPT_Rise_Smoothing'][1]
        )
    
    for i in messu.index: # Startpunkt über Vorzeichenwechsel im Anstieg
        if messu.loc[i,'driF_schg']:
            if not messu.loc[
                    i+1:i+max(int(_opts['OPT_Determination_Distance'][0]/2),1),
                    'driF_schg'
                    ].any():
                messu_iS=i
                break
    
    messu_iS,_=emec.mc_char.find_SandE(
        messu.loc[
            messu_iS:messu_iS+_opts['OPT_Determination_Distance'][0],
            'driF'
            ],
        abs(messu['driF']).quantile(0.5),
        "pgm_other", 0.1)

    # _,messu_iE=Evac.find_SandE(messu['driF'],0,"qua_self",0.5) # changed 211022 (B5-sr09)
    try: # search after maximum Force
        _,messu_iE=emec.mc_char.find_SandE(messu['driF'].loc[messu.Force.idxmax():],
                                    messu['driF'],"qua_other",0.5)
    except IndexError:
        messu_iE=dic_to_mess_End
        
    messu_iE=min(messu_iE,dic_to_mess_End)
    
    log_custom("\n "+"-"*100,**log_scopt)
    log_custom("\n   Start of evaluation after %.3f seconds, corresponds to %.5f %% of max. force."
               %(messu.Time[messu_iS],100*abs(messu.Force[messu_iS])/abs(messu.Force).max()),
               **log_scoptf)
    
    messu=messu.loc[messu_iS:messu_iE]
    if _opts['OPT_DIC']:
        step_range_dic = emec.pd_ext.pd_combine_index(step_range_dic,messu.index)
        
    fig, ax1 = plt.subplots()
    ax1.set_title('%s - Measuring'%plt_name)
    color1 = 'tab:red'
    ax1.set_xlabel('Time / s')
    ax1.set_ylabel('Force / N', color=color1)  # we already handled the x-label with ax1
    ax1.tick_params(axis='y', labelcolor=color1)
    ax1.axvline(x=messu.Time.loc[messu_iS], color='gray', linestyle='-')
    ax1.axvline(x=messu.Time.loc[messu_iE], color='gray', linestyle='-')
    if np.invert(np.isnan(_opts['OPT_End'])):
        ax1.axvline(x=_opts['OPT_End'], color='gray', linestyle='-.')
    ax1.plot(mess.Time, 
             mess[_opts['OPT_Measurement_file']['used_names_dict']['Force']],
             'r-', label='Force')
    if _opts['OPT_Springreduction']: 
        ax1.plot(mess.Time, mess.F_IWA_red, 'b:', label='Force-red. (LVDT)')
    ax2 = ax1.twinx()  # instantiate a second axes that shares the same x-axis
    color2 = 'tab:blue'
    ax2.grid(False)
    ax2.set_ylabel('Way / mm', color=color2)  # we already handled the x-label with ax1
    ax2.tick_params(axis='y', labelcolor=color2)
    ax2.plot(mess.Time, (mess.L_PM), 'y--', label='Way-PM')
    ax2.plot(mess.Time, -(mess.L_IWA1-mess.L_IWA1[0]), 'b--', label='Way-IWA1')
    ax2.plot(mess.Time, -(mess.L_IWA2-mess.L_IWA2[0]), 'b-.', label='Way-IWA2')
    if _opts['OPT_DIC']:
        ax2.plot(dic.Time, dic.Disp_opt_head, 'k:', label='Way-DIC')
        # ax2.plot(dic.Time, dic.DDisp_PM_c, 'm:', label='Way-DIC-P')
        # ax2.plot(dic.Time, dic.DDisp_PC_c, 'g:', label='Way-DIC-C')
    fig.legend(loc='upper left', bbox_to_anchor=(0.1, 0.9), ncol=1)
    plt_hsuf(fig,path=out_full+"-meas",**plt_scopt)
    
    # =============================================================================
    #%%% 5.2 Resetting way
    timings.loc[5.2]=time.perf_counter()
    log_custom("\n   Timing %f: %.5f s"%(timings.index[-1],
                                         timings.iloc[-1]-timings.iloc[0]),
               **log_scopt)
    # messu.Force=messu.Force-messu.Force.loc[messu_iS]
    messu.Way=messu.Way-messu.Way.loc[messu_iS]
    
    if _opts['OPT_DIC']:    
        messu.Disp_opt_head=messu.Disp_opt_head-messu.Disp_opt_head.loc[messu_iS]
        
    fig, ax1 = plt.subplots()
    ax1.set_title('%s - Measuring (used)'%plt_name)
    color1 = 'tab:red'
    ax1.set_xlabel('Time / s')
    ax1.set_ylabel('Force / N', color=color1)  # we already handled the x-label with ax1
    ax1.tick_params(axis='y', labelcolor=color1)
    ax1.axvline(x=messu.Time.loc[messu_iS], color='gray', linestyle='-')
    ax1.axvline(x=messu.Time.loc[messu_iE], color='gray', linestyle='-')
    ax1.plot(messu.Time, messu.Force, 'r-', label='Force')
    ax2 = ax1.twinx()  # instantiate a second axes that shares the same x-axis
    color2 = 'tab:blue'
    ax2.grid(False)
    ax2.set_ylabel('Way / mm', color=color2)  # we already handled the x-label with 
    ax2.tick_params(axis='y', labelcolor=color2)
    ax2.plot(messu.Time, messu.Way, 'b--', label='Way')
    if _opts['OPT_DIC']:
        ax2.plot(messu.Time, messu.Disp_opt_head, 'k:', label='Way-DIC')
    fig.legend(loc='upper left', bbox_to_anchor=(0.1, 0.9), ncol=1)
    plt_hsuf(fig,path=out_full+"-meas_u",**plt_scopt)
    
    # =============================================================================
    #%% 6 Evaluation
    log_custom("\n "+"="*100,**log_scopt)
    log_custom("\n ### 6 Evaluation ###",**log_scoptf)
    timings.loc[6.0]=time.perf_counter()
    log_custom("\n   Timing %f: %.5f s"%(timings.index[-1],
                                       timings.iloc[-1]-timings.iloc[0]),
               **log_scopt)
    
    #%%% 6.2 Determine evaluation curves
    log_custom("\n "+"-"*100,**log_scopt)
    log_custom("\n ### -6.2 Determine evaluation curves ###",
               **log_scopt)
    timings.loc[6.2]=time.perf_counter()
    log_custom("\n   Timing %f: %.5f s"%(timings.index[-1],
                                       timings.iloc[-1]-timings.iloc[0]),
               **log_scopt)
    
    messu['Strain']=messu.Way/Length
    messu['Stress']=messu.Force/Area

    if _opts['OPT_DIC']:
        tmon=emec.mc_char.test_pdmon(
            messu,
            ['Stress','Strain',
             'Strain_opt_d_A','Strain_opt_d_S','Strain_opt_d_M',
             'Strain_opt_c_A','Strain_opt_c_S','Strain_opt_c_M'],
            1,10
            )
    else:
        tmon=emec.mc_char.test_pdmon(messu,['Stress','Strain'],1,10)
    
    log_custom("\n   Last 10 monoton increasing periods:\n    %s"
               %tmon.to_frame(name='Epoche').T.to_string().replace('\n','\n    '),
               **log_scoptf)
    
    # =============================================================================
    #%%% 6.3 Determine points of interest
    log_custom("\n "+"-"*100,**log_scopt)
    log_custom("\n ### -6.3 Determine points of interest ###",
               **log_scopt)
    timings.loc[6.31]=time.perf_counter()
    log_custom("\n   Timing %f: %.5f s"%(timings.index[-1],
                                       timings.iloc[-1]-timings.iloc[0]),
               **log_scopt)
    
    tmp_odd1=_opts['OPT_Determination_Distance'][1]
    VIP_messu=pd.Series([],dtype='int64',name='VIP_messu')
    VIP_messu['S']=messu.driF.index[0]
    VIP_messu['E']=messu.driF.index[-1]
    VIP_messu['U']=messu.Force.idxmax()
    
    if _opts['OPT_pre_load_cycles']>0:
        # Zeitdifferenz zwischen Messverfahren und Zuordnung von Zyklen
        dt_drif_schg=1/_opts['OPT_Resampling_Frequency']
        # mun_tmp=messu.Time[messu.driF_schg].loc[
        #     (VIP_messu['S']+tmp_odd1):(VIP_messu['U']-tmp_odd1)
        #     ]
        _,_,driw_schg=emec.mc_char.rise_curve(
            messu['Way'],False,4
            ) # Using Way instead of force for pointing cycles
        mun_tmp=messu.Time[driw_schg].loc[
                    (VIP_messu['S']+tmp_odd1):(VIP_messu['U']-tmp_odd1)
                    ]
        dt=pd.DataFrame(data={'d1':mun_tmp.diff(),'d2':-mun_tmp.diff(-1)})
        dt['dmin']=dt.loc(axis=1)['d1':'d2'].min(axis=1).round(10)
        drif_schg_counter=dt.dmin.loc[
            abs((dt.dmin-dt.dmin.iloc[1:-1].max())
                /dt.dmin.iloc[1:-1].max())
            <=dt_drif_schg
            ]
        
        i=0
        while (drif_schg_counter.count()!=_opts['OPT_pre_load_cycles']*2):
            i+=1
            if (drif_schg_counter.count()>_opts['OPT_pre_load_cycles']*2):
                dt_drif_schg=dt_drif_schg/1.15
            else:
                dt_drif_schg=dt_drif_schg*1.1
            drif_schg_counter=dt.dmin.loc[
                abs((dt.dmin-dt.dmin.iloc[1:-1].max())
                    /dt.dmin.iloc[1:-1].max())
                <=dt_drif_schg
                ]
            if i==50:
                log_custom("\n  %d cycles, without result!\n    -> Using last %d as counter."
                           %(i,drif_schg_counter.count()),**log_scopt)
                break
        log_custom("\n  %d cycles, with result!\n    -> Using last %.3f s as delimiter."
                   %(i,dt_drif_schg),**log_scopt)
        
        zyklus=1
        VIP_PMf=pd.Series([],dtype='int64') #konventionell-Kraft
        VIP_PMw=pd.Series([],dtype='int64') #konventionell-Weg
        VIP_DICw=pd.Series([],dtype='int64') #Bildkorrelation-Weg
        
        for i in drif_schg_counter.index:
            #if period not named and cyclic loading applied
            if (np.invert(i in VIP_messu)) and (_opts['OPT_pre_load_cycles']!=0):
                if zyklus<=_opts['OPT_pre_load_cycles'] and (i>VIP_messu['S']+tmp_odd1):
                    if np.sign(messu.dcuF.loc[i]) == -1.0:
                        VIP_messu['C'+str(zyklus) + '-'] = abs(
                            messu.Force.loc[i-tmp_odd1/2:i+tmp_odd1/2+1]
                            ).idxmax()
                        VIP_PMf['C'+str(zyklus) + '-'] = abs(
                            messu.Force.loc[i-tmp_odd1/2:i+tmp_odd1/2+1]
                            ).idxmax()
                        VIP_PMw['C'+str(zyklus) + '-']  =abs(
                            messu.Way.loc[i-tmp_odd1/2:i+tmp_odd1/2+1]
                            ).idxmax()
                        if _opts['OPT_DIC']: 
                            VIP_DICw['C'+str(zyklus) + '-']=abs(
                                messu.DWay.loc[i-tmp_odd1/2:i+tmp_odd1/2+1]
                                ).idxmax()               
                        zyklus += 1
                    else:
                        VIP_messu['C'+str(zyklus) + '+'] = abs(
                            messu.Force.loc[i-tmp_odd1/2:i+tmp_odd1/2+1]
                            ).idxmin()
                        VIP_PMf['C'+str(zyklus) + '+']  =abs(
                            messu.Force.loc[i-tmp_odd1/2:i+tmp_odd1/2+1]
                            ).idxmin()
                        VIP_PMw['C'+str(zyklus) + '+'] = abs(
                            messu.Way.loc[i-tmp_odd1/2:i+tmp_odd1/2+1]
                            ).idxmin()   
                        if _opts['OPT_DIC']: 
                            VIP_DICw['C'+str(zyklus) + '+'] = abs(
                                messu.DWay.loc[i-tmp_odd1/2:i+tmp_odd1/2+1]
                                ).idxmin()              
                elif zyklus==(_opts['OPT_pre_load_cycles']+1):
                    VIP_messu['F1'] = abs(
                        messu.Force.loc[i-tmp_odd1/2:i+tmp_odd1/2+1]
                        ).idxmin()
                    VIP_PMf['F1'] = abs(
                        messu.Force.loc[i-tmp_odd1/2:i+tmp_odd1/2+1]
                        ).idxmin()
                    VIP_PMw['F1'] = abs(
                        messu.Way.loc[i-tmp_odd1/2:i+tmp_odd1/2+1]
                        ).idxmin() 
                    if _opts['OPT_DIC']: 
                        VIP_DICw['F1'] = abs(
                            messu.DWay.loc[i-tmp_odd1/2:i+tmp_odd1/2+1]
                            ).idxmin()
                    zyklus += 1            
        VIP_messu['C1+']=(abs(messu.Force.loc[VIP_messu['S']:VIP_messu['C1-']]-messu.Force.loc[VIP_messu[VIP_messu.index.str.endswith('+')]].mean())).idxmin()
        VIP_messu['F2']=(abs(messu.Force.loc[VIP_messu['F1']:VIP_messu['U']]-messu.Force.loc[VIP_messu[VIP_messu.index.str.endswith('-')]].mean())).idxmin()
        
        VIP_PMdiff=VIP_PMf-VIP_PMw
        VIP_PDdiff=VIP_PMf-VIP_DICw
        VIP_mess_diffs=pd.DataFrame(data={
            'PM_Force':VIP_PMf, 'PM_Way':VIP_PMw,
            'DIC_way':VIP_DICw,'Diff_PM':VIP_PMdiff,'Diff_PD':VIP_PDdiff
            })
        
        log_custom("\n "+"-"*100,**log_scopt)
        log_custom("\n  Differenz Extremwertvergleich: \n  %s"
                   %VIP_mess_diffs.to_string(),**log_scoptf)
        log_custom("\n  Differenz Kraft- zu Wegextreme-PM (MW):  %f"
                   %VIP_PMdiff.mean(),**log_scoptf)
        log_custom("\n  Differenz Kraft- zu Wegextreme-DIC (MW): %f"
                   %VIP_PDdiff.mean(),**log_scoptf)      

    else: #no cyclic preloading (fixed range set to determination range)
        VL_soll=_opts['OPT_YM_Determination_range'][0]*messu.Force.max() # VL_soll Krücke, da nan in Protokoll
        ZL_soll=_opts['OPT_YM_Determination_range'][1]*messu.Force.max() # ZL_soll Krücke, da nan in Protokoll
        tmp_Fr=messu.Force.loc[VIP_messu['S']:VIP_messu[_opts['OPT_YM_Determination_range'][2]]]
        VIP_messu['F1']=(abs(tmp_Fr-VL_soll)).idxmin()
        VIP_messu['F2']=(abs(tmp_Fr-ZL_soll)).idxmin()    

    # B    
    mun_tmp = messu.loc[VIP_messu['F1']+tmp_odd1:VIP_messu['U']-1]
    if mun_tmp.driF_schg.any()==True: # 
        VIP_messu['Y']=mun_tmp.loc[mun_tmp.driF_schg==True].index[0]-1
    else:
        VIP_messu['Y']=VIP_messu['U']
        log_custom('\n    Fy set on datapoint of Fu!',**log_scoptf) 
        
    # mun_tmp = messu.loc[VIP_messu['U']:VIP_messu['E']-1]
    mun_tmp = messu.loc[VIP_messu['U']-1:VIP_messu['E']-1]
    if mun_tmp.driF_schg.any():
        i=mun_tmp.loc[mun_tmp.driF_schg].index[0]
        VIP_messu['B'] = mun_tmp.driF.loc[i:i+tmp_odd1].idxmin()-2 # statt allgemeinem Minimum bei größtem Kraftabfall nahe Maximalkraft, -2 da differenz aussage über vorherigen punkt
        if VIP_messu['B']<VIP_messu['U']: VIP_messu['B']=VIP_messu['U']
    # # if (mun_tmp['driF'].min()/mun_tmp['driF'].quantile(0.25))>=2:
    # if (mun_tmp['driF'].min()/mun_tmp['driF'].quantile(0.25))>=1.0:
    #     VIP_messu['B']=mun_tmp['driF'].idxmin()-1
    else:
        log_custom('\n   Fb not reliably determinable!',**log_scoptf)
            
    # ftmp=float(messu.Force.loc[VIP_messu[_opts['OPT_YM_Determination_range'][2]]]*_opts['OPT_YM_Determination_range'][0])
    # VIP_messu['F1']=abs(messu.Force.loc[:VIP_messu[_opts['OPT_YM_Determination_range'][2]]]-ftmp).idxmin()
    # ftmp=float(messu.Force.loc[VIP_messu[_opts['OPT_YM_Determination_range'][2]]]*_opts['OPT_YM_Determination_range'][1])
    # VIP_messu['F2']=abs(messu.Force.loc[:VIP_messu[_opts['OPT_YM_Determination_range'][2]]]-ftmp).idxmin()
    
    if (VIP_messu['Y']>VIP_messu['F1']) and (VIP_messu['Y']<VIP_messu['F2']): # Test ob Streckgrenze zwischen F1 und F2 liegt
        VIP_messu['F2']=VIP_messu['Y']
        # VIP_messu['F4']=VIP_messu['Y']
        # VIP_dicu['F2']=VIP_dicu['Y']
        # VIP_dicu['F4']=VIP_dicu['Y']
        log_custom("\n   F2 set on Y (Force-rise between F1 and old F2)",
                   **log_scoptf)
    
    VIP_messu=VIP_messu.sort_values()
    if _opts['OPT_DIC']:
        VIP_dicu=VIP_messu.copy(deep=True)
        VIP_dicu.name='VIP_dicu'
    
    #%%%% 6.3.2 Improvement of evaluation range
    timings.loc[6.32]=time.perf_counter()
    log_custom("\n   Timing %f: %.5f s"%(timings.index[-1],
                                       timings.iloc[-1]-timings.iloc[0]),
               **log_scopt)
    
    # (siehe Keuerleber, M. (2006). Bestimmung des Elastizitätsmoduls von Kunststoffen bei hohen Dehnraten am Beispiel von PP. Von der Fakultät Maschinenbau der Universität Stuttgart zur Erlangung der Würde eines Doktor-Ingenieurs (Dr.-Ing.) genehmigte Abhandlung. Doktorarbeit. Universität Stuttgart, Stuttgart.)
    if _opts['OPT_pre_load_cycles'] == 0:
        iS='S'
    else:
        iS='F1'
    
    VIP_messu, DQcons, txt = emec.mc_char.YM_eva_range_refine(
        m_df=messu, VIP=VIP_messu,
        n_strain='Strain', n_stress='Stress',
        n_loBo=iS, n_upBo=_opts['OPT_YM_Determination_refinement'][2],
        d_loBo=_opts['OPT_YM_Determination_refinement'][0],
        d_max=_opts['OPT_YM_Determination_refinement'][1], 
        rise_det=_opts['OPT_YM_Determination_refinement'][-2:],
        n_Outlo='F3',n_Outmi='FM',n_Outhi='F4')
        
    log_custom(log_cind(txt),**log_scopt)
    if True:
        fig, (ax1) = plt.subplots(nrows=1, ncols=1, 
                                  sharex=False, sharey=False, 
                                  figsize = figsize)
        fig.suptitle('%s - Improvement of evaluation range for Youngs Modulus'%plt_name)
        ax1.set_title('Conventional measured strain')
        ax1.set_xlabel('Strain / -')
        ax1.set_ylabel('Stress / MPa')
        ax1.plot(messu.loc[VIP_messu[iS]:VIP_messu[_opts['OPT_YM_Determination_refinement'][2]],'Strain'], messu.loc[VIP_messu[iS]:VIP_messu[_opts['OPT_YM_Determination_refinement'][2]],'Stress'], 'r.',label='$\sigma$-$\epsilon$')
        a, b=messu.loc[VIP_messu[iS:_opts['OPT_YM_Determination_refinement'][2]],'Strain'], messu.loc[VIP_messu[iS:_opts['OPT_YM_Determination_refinement'][2]],'Stress']
        j=np.int64(-1)
        ax1.plot(a, b, 'bx')
        for x in VIP_messu[iS:_opts['OPT_YM_Determination_refinement'][2]].index:
            j+=1
            if j%2: c=(6,-6)
            else:   c=(-6,6)
            ax1.annotate('%s' % x, xy=(a.iloc[j],b.iloc[j]), xycoords='data',
                         xytext=c, ha="center", va="center", textcoords='offset points')
        ax2=ax1.twinx()
        ax2.set_ylabel('Normalized derivatives / -')
        ax2.plot(DQcons['Strain'], 
                 DQcons['DQ1']/DQcons['DQ1'].max(), 'b:',label='DQ1')
        ax2.plot(DQcons['Strain'], 
                 DQcons['DQ2']/abs(DQcons['DQ2']).max(), 'g:',label='DQ2')
        ax2.plot(DQcons['Strain'], 
                 DQcons['DQ3']/abs(DQcons['DQ3']).max(), 'y:',label='DQ3')
        ax2.axvline(x=messu.loc[VIP_messu['FM'],'Strain'],color='gray', linestyle='-')
        ax2.axvline(x=messu.loc[VIP_messu['F3'],'Strain'],color='gray', linestyle=':')
        ax2.axvline(x=messu.loc[VIP_messu['F4'],'Strain'],color='gray', linestyle=':')
        ax2.axhline(y=_opts['OPT_YM_Determination_refinement'][1],color='gray', linestyle='--')
        ax2.set_yticks([-1,0,1])
        ax2.grid(which='major',axis='y',linestyle=':')
        fig.legend(loc='lower right', ncol=4)
        plt_hsuf(fig,path=out_full+"-YMRange_Imp",**plt_scopt)
    
    if _opts['OPT_DIC']:
        tmp={'con F1-F2':VIP_messu['F2']-VIP_messu['F1'],
             'opt F1-F2':VIP_dicu['F2']-VIP_dicu['F1'],
             'con F3-F4':VIP_messu['F4']-VIP_messu['F3'],
             'opt F3-F4':VIP_dicu['F4']-VIP_dicu['F3']}
        log_custom("\n   Datapoints (con/opt) between F1-F2: %d/%d and F3-F4: %d/%d."
                       %(*tmp.values(),),**log_scopt)
        for i in tmp.keys(): 
            if tmp[i] < 3: cout+='%s:%d DPs, '%(i,tmp[i])
    else:
        tmp={'con F1-F2':VIP_messu['F2']-VIP_messu['F1'],
             'con F3-F4':VIP_messu['F4']-VIP_messu['F3']}
        log_custom("\n   Datapoints (con) between F1-F2: %d and F3-F4: %d."
                       %(*tmp.values(),),**log_scopt)
        for i in tmp.keys(): 
            if tmp[i] < 3: cout+='%s:%d DPs, '%(i,tmp[i])

    # =====================================================================================
    #%%% 6.4 Determine Youngs-Moduli
    log_custom("\n "+"-"*100,**log_scopt)
    log_custom("\n ### -6.4 Determine Youngs-Moduli ###",**log_scopt)
    timings.loc[6.4]=time.perf_counter()
    log_custom("\n   Timing %f: %.5f s"%(timings.index[-1],
                                       timings.iloc[-1]-timings.iloc[0]),
               **log_scopt)
    
    d_stress_mid = messu.Stress.diff()
    d_strain_mid = messu.Strain.diff()
    d_Force = messu.Force.diff()
    
    Ind_YM_f=['F1','F2']
    Ind_YM_r=['F3','F4']
    sf_eva_con = messu.loc[VIP_messu[Ind_YM_f[0]]:VIP_messu[Ind_YM_f[1]]].index
    sr_eva_con = messu.loc[VIP_messu[Ind_YM_r[0]]:VIP_messu[Ind_YM_r[1]]].index    

    # -------------------------------------------------------------------------------------
    #%%%% 6.4.1 Method A
    timings.loc[6.41]=time.perf_counter()
    log_custom("\n   Timing %f: %.5f s"%(timings.index[-1],
                                       timings.iloc[-1]-timings.iloc[0]),
               **log_scopt)
    
    A0Al_ser = emec.fitting.YM_eva_com_sel(
        stress_ser=d_stress_mid,
        strain_ser=d_strain_mid,
        comp=_opts['OPT_Compression'],
        name='A0Al', 
        det_opt='incremental'
        )
    E_A_df = pd.concat([A0Al_ser],axis=1)
    E_A = emec.stat_ext.pd_agg(E_A_df.loc[sr_eva_con])

    if True:
        fig, (ax1,ax2) = plt.subplots(nrows=2, ncols=1, 
                                      sharex=False, sharey=False, 
                                      figsize = np.multiply(figsize,[1,2]))
        fig.suptitle('%s - Compare method A'%(plt_name))
        ax1.set_title('All Steps')
        ax1.set_xlabel('Step / -')
        ax1.set_ylabel('E / MPa')
        cc={'A0Al':'m:'}
        for k in cc:
            ax1.plot(E_A_df.loc[:VIP_messu['U']].index,
                     E_A_df.loc[:VIP_messu['U']][k],
                     cc[k], label='%s - %.2f MPa'%(k,E_A.at['mean',k]))
        ax1.axvline(x=VIP_messu['F3'], color='brown', linestyle=':')
        ax1.axvline(x=VIP_messu['F4'], color='brown', linestyle='--')
        if _opts['OPT_DIC']:
            ax1.axvline(x=VIP_dicu['F3'], color='olive', linestyle=':')
            ax1.axvline(x=VIP_dicu['F4'], color='olive', linestyle='--')
        ax1.legend()
        ax2.set_title('Improved determination range')
        ax2.set_xlabel('Step / -')
        ax2.set_ylabel('E / MPa')
        cr={'A0Al':sr_eva_con}
        for k in cc:
            ax2.plot(cr[k], E_A_df[k].loc[cr[k]], cc[k])
        ax2.axvline(x=VIP_messu['F3'], color='brown', linestyle=':')
        ax2.axvline(x=VIP_messu['F4'], color='brown', linestyle='--')
        if _opts['OPT_DIC']:
            ax2.axvline(x=VIP_dicu['F3'], color='olive', linestyle=':')
            ax2.axvline(x=VIP_dicu['F4'], color='olive', linestyle='--')
        plt_hsuf(fig,path=out_full+"-YM-Me_A",**plt_scopt)
        
    #least-square fit
    E_lsq_F_A0Al = emec.fitting.YM_eva_com_sel(
        stress_ser=messu.Stress,
        strain_ser=messu.Strain,
        comp=_opts['OPT_Compression'],
        name='E_lsq_F_A0Al', 
        det_opt='leastsq',
        **{'ind_S':VIP_messu[Ind_YM_f[0]],
           'ind_E':VIP_messu[Ind_YM_f[1]]}
        )
    E_lsq_F_A0Al = pd.Series(E_lsq_F_A0Al, index=['E','E_abs','Rquad','Fit_result'],
                            name='E_lsq_F_A0Al')
    
    E_lsq_R_A0Al = emec.fitting.YM_eva_com_sel(
        stress_ser=messu.Stress,
        strain_ser=messu.Strain,
        comp=_opts['OPT_Compression'],
        name='E_lsq_R_A0Al', 
        det_opt='leastsq',
        **{'ind_S':VIP_messu[Ind_YM_r[0]],
           'ind_E':VIP_messu[Ind_YM_r[1]]}
        )
    E_lsq_R_A0Al = pd.Series(E_lsq_R_A0Al, index=['E','E_abs','Rquad','Fit_result'],
                            name='E_lsq_R_A0Al')
    
    E_lsq_A = pd.concat([E_lsq_F_A0Al, E_lsq_R_A0Al],axis=1)
    
    for i in VIP_messu[VIP_messu.index.str.endswith('+')].index:
        j = VIP_messu.index[VIP_messu.index.get_indexer_for([i])[0]+1]
        E_lsq_C_A0Al = emec.fitting.YM_eva_com_sel(
            stress_ser=messu.Stress,
            strain_ser=messu.Strain,
            comp=_opts['OPT_Compression'],
            name='E_lsq_%s_A0Al'%i, 
            det_opt='leastsq',
            **{'ind_S':VIP_messu[i],
               'ind_E':VIP_messu[j]}
            )
        E_lsq_C_A0Al = pd.Series(E_lsq_C_A0Al, 
                                 index=['E','E_abs','Rquad','Fit_result'],
                                 name='E_lsq_%s_A0Al'%i)
        E_lsq_A = pd.concat([E_lsq_A, E_lsq_C_A0Al],axis=1)
        
        k = VIP_messu.index[VIP_messu.index.get_indexer_for([j])[0]+1]
        E_lsq_C_A0Al = emec.fitting.YM_eva_com_sel(
            stress_ser=messu.Stress,
            strain_ser=messu.Strain,
            comp=_opts['OPT_Compression'],
            name='E_lsq_%s_A0Al'%j, 
            det_opt='leastsq',
            **{'ind_S':VIP_messu[j],
               'ind_E':VIP_messu[k]}
            )
        E_lsq_C_A0Al = pd.Series(E_lsq_C_A0Al, 
                                 index=['E','E_abs','Rquad','Fit_result'],
                                 name='E_lsq_%s_A0Al'%j)
        E_lsq_A = pd.concat([E_lsq_A, E_lsq_C_A0Al],axis=1)
    
    
    del A0Al_ser
    del E_lsq_F_A0Al, E_lsq_R_A0Al
        
    ind = VIP_messu[VIP_messu.index.str.contains('\+|F1')]
    strain_plastic = (messu.Strain.loc[ind]).diff()
    strain_plastic.index = ind.index.str.replace('F1','F').str.replace('+','')
    strain_plastic=strain_plastic.shift(-1)
    
    E_asc_ser = E_lsq_A.loc['E',E_lsq_A.columns.str.contains('\+')]
    E_asc_ser = pd.concat([E_asc_ser,E_lsq_A.loc['E',E_lsq_A.columns.str.contains('F')]])
    E_des_ser = E_lsq_A.loc['E',E_lsq_A.columns.str.contains('\-')]
    E_des_ser['F']=np.nan
    df_Ee = pd.DataFrame({'E_asc':E_asc_ser.values,'E_des':E_des_ser.values,
                          'e_pl':strain_plastic}, index = strain_plastic.index)
    
    #%%%% 6.4.8 Method compare
    timings.loc[6.48]=time.perf_counter()
    log_custom("\n   Timing %f: %.5f s"%(timings.index[-1],
                                       timings.iloc[-1]-timings.iloc[0]),
               **log_scopt)    

    E_lsq=E_lsq_A

    E_Methods_df = E_A_df
    E_agg_funcs = ['mean',emec.stat_ext.meanwoso,'median',
                   'std',emec.stat_ext.stdwoso,
                   emec.stat_ext.cv, emec.stat_ext.cvwoso,
                   'min','max']
    
    E_inc_F_comp = E_Methods_df.loc[sf_eva_con].agg(E_agg_funcs)
    E_inc_R_comp = E_Methods_df.loc[sr_eva_con].agg(E_agg_funcs)
    
    log_custom("\n\n  Method comaparison:",**log_scoptf)
    log_custom("\n  - least square fit",**log_scoptf)
    log_custom(log_cind('\n'+E_lsq.loc[['E','Rquad']].T.to_string()),
               **log_scoptf)
    
    log_custom("\n\n  - incremental (F,R):",**log_scoptf)
    log_custom(log_cind('\n'+E_inc_F_comp.T.to_string()),**log_scoptf)
    log_custom(log_cind('\n'+E_inc_R_comp.T.to_string()),**log_scoptf)
    
    # set preffered Method
    YM_pref_con=E_lsq_A['E_lsq_R_A0Al']
    
    if _opts['OPT_DIC']:
        if loc_Yd_tmp.startswith('E_lsq'):
            YM_pref_opt=E_lsq[loc_Yd_tmp]
        elif loc_Yd_tmp.startswith('E_inc_R'):
            YM_pref_opt=emec.fitting.Refit_YM_vals(
                m_df=messu, 
                YM = E_inc_R_comp[loc_Yd_tmp.split('_')[-1]]['meanwoso'], 
                VIP=VIP_dicu,
                n_strain=dic_used_Strain, n_stress='Stress',
                n_loBo=['F3'], n_upBo=['F4']
                )
        elif loc_Yd_tmp.startswith('E_inc_F'):
            YM_pref_opt=emec.fitting.Refit_YM_vals(
                m_df=messu, 
                YM = E_inc_F_comp[loc_Yd_tmp.split('_')[-1]]['meanwoso'], 
                VIP=VIP_dicu,
                n_strain=dic_used_Strain, n_stress='Stress',
                n_loBo=['F1'], n_upBo=['F2']
                )
        else:
            raise NotImplementedError('Prefered YM-method not selectable!')
    # --------------------------------------------------------------------------
    #%%% 6.5 Determine yield point
    log_custom("\n "+"-"*100,**log_scopt)
    log_custom("\n ### -6.5 Determine yield point ###",**log_scopt)
    timings.loc[6.5]=time.perf_counter()
    log_custom("\n   Timing %f: %.5f s"%(timings.index[-1],
                                       timings.iloc[-1]-timings.iloc[0]),
               **log_scopt)    
    
    strain_osd = {'YK':0.0,'Y0':0.0,'Y1':0.1/100,'Y':0.2/100,'Y2':0.5/100} #acc. Zhang et al. 2021, DOI: 10.1007/s10439-020-02719-2
    strain_osdf={'YK':'F4'}

    log_custom("\n  Determination of yield strain-conventional:",**log_scoptf)
    tmp=emec.mc_yield.Yield_redet2_Multi(
        m_df=messu, VIP=VIP_messu,
        strain_osd=strain_osd, strain_osdf=strain_osdf,
        n_strain='Strain', n_stress='Stress',
        n_loBo=['F3'], n_upBo=['U'], n_loBo_int=['F3'],
        YM     = YM_pref_con['E'],
        YM_abs = YM_pref_con['E_abs'],
        use_rd =True, 
        rise_det=[
            _opts['OPT_Rise_Smoothing'][0], _opts['OPT_Rise_Smoothing'][1]
            ], 
        ywhere='n'
        )
    VIP_messu, yield_df_con, txt = tmp
    log_custom(log_cind(txt,3), **log_scoptf)
    if _opts['OPT_DIC']:
        log_custom("\n  Determination of yield strain-optical:",**log_scoptf)
        tmp=emec.mc_yield.Yield_redet2_Multi(
            m_df=messu, VIP=VIP_dicu,
            strain_osd=strain_osd, strain_osdf=strain_osdf,
            n_strain=dic_used_Strain, n_stress='Stress',
            n_loBo=['F3'], n_upBo=['U'], n_loBo_int=['F3'],
            YM     = YM_pref_opt['E'],
            YM_abs = YM_pref_opt['E_abs'],
            use_rd =True,
            rise_det=[
                _opts['OPT_Rise_Smoothing'][0], _opts['OPT_Rise_Smoothing'][1]
                ],  
            ywhere='n'
            )
        VIP_dicu, yield_df_opt, txt = tmp
        log_custom(log_cind(txt,3),**log_scoptf)
    
    fig, (ax1,ax2) = plt.subplots(nrows=2, ncols=1, 
                                  sharex=False, sharey=False, 
                                  figsize = np.multiply(figsize,[1,2]))
    fig.suptitle('%s - Stress vs. strain curve - yield point determination'%plt_name)
    ax1.set_title('Conventional strain')
    ax1.set_xlabel('Strain / -')
    ax1.set_ylabel('Stress / MPa')
    ax1.plot(messu.loc[:VIP_messu['B']]['Strain'], 
             messu.loc[:VIP_messu['B']]['Stress'], 'r--',label='con')
    tmp=ax1.get_xlim(),ax1.get_ylim()
    for i in strain_osd.keys():
        ax1.plot(emec.fitting.strain_linfit(
            [0,1000],
            YM_pref_con['E'], 
            YM_pref_con['E_abs'], 
            strain_offset=strain_osd[i]
            ),
            [0,1000],
            '-',
            label='$E_{off-%.3fp}$'%(strain_osd[i]*100)
            )
    ax1.set_xlim(tmp[0])
    ax1.set_ylim(tmp[1])
    a, b=messu.Strain[VIP_messu[:'B']],messu.Stress[VIP_messu[:'B']]
    j=np.int64(-1)
    ax1.plot(a, b, 'bx')
    for x in VIP_messu[:'B'].index:
        j+=1
        if j%2: c=(6,-6)
        else:   c=(-6,6)
        ax1.annotate('%s' % x, xy=(a.iloc[j],b.iloc[j]), xycoords='data',
                      xytext=c, ha="center", va="center", textcoords='offset points')
    ax1.legend()
    ax2.set_title('Optical strain')
    if _opts['OPT_DIC']:
        ax2.set_xlabel('Strain / -')
        ax2.set_ylabel('Stress / MPa')
        ax2.plot(messu.loc[:VIP_dicu['B']][dic_used_Strain], 
                 messu.loc[:VIP_dicu['B']]['Stress'], 
                 'r--',label='opt')
        tmp=ax2.get_xlim(),ax1.get_ylim()
        for i in strain_osd.keys():
            ax2.plot(emec.fitting.strain_linfit(
                [0,1000],
                YM_pref_opt['E'], 
                YM_pref_opt['E_abs'], 
                strain_offset=strain_osd[i]
                ),
                [0,1000],
                '-',
                label='$E_{off-%.3fp}$'%(strain_osd[i]*100)
                )
        ax2.set_xlim(tmp[0])
        ax2.set_ylim(tmp[1])
        a, b=messu[dic_used_Strain][VIP_dicu[:'B']],messu.Stress[VIP_dicu[:'B']]
        j=np.int64(-1)
        ax2.plot(a, b, 'bx')
        for x in VIP_dicu[:'B'].index:
            j+=1
            if j%2: c=(6,-6)
            else:   c=(-6,6)
            ax2.annotate('%s' % x, xy=(a.iloc[j],b.iloc[j]), xycoords='data',
                          xytext=c, ha="center", va="center", textcoords='offset points')
        ax2.legend()
    plt_hsuf(fig,path=out_full+"-sigeps_yielddet",**plt_scopt)
    
    # =============================================================================
    #%%% 6.6 Determine final curve
    log_custom("\n "+"-"*100,**log_scopt)
    log_custom("\n ### -6.6 Determine final stress-strain curve ###",
               **log_scopt)
    timings.loc[6.6]=time.perf_counter()
    log_custom("\n   Timing %f: %.5f s"%(timings.index[-1],
                                       timings.iloc[-1]-timings.iloc[0]),
               **log_scopt)
    if ('B' in VIP_messu.index) and (VIP_messu['B']>=VIP_messu['U']):
        FP_end='B'
    else:
        FP_end='U'
    FP_sta='F3'
    
    FinSSC = True # finale kurve für werteermittlung auf Ym linearisieren an anfang
    if FinSSC:
        messu_FP, linstrainos_con = emec.mc_man.DetFinSSC(
            mdf=messu, YM=YM_pref_con, 
            iS=VIP_messu['F3'], iLE=None,
            StressN='Stress', StrainN='Strain', 
            addzero=True, izero=VIP_messu['S']
            )
        yield_df_con['Strain']=yield_df_con['Strain']-linstrainos_con
        yield_df_con['Force']=yield_df_con['Stress']*Area
        yield_df_con['Way']=yield_df_con['Strain']*Length
        log_custom("\n   Strain offset about %.5f"%(linstrainos_con),
                   **log_scopt)
        if _opts['OPT_DIC']:
            tmp, linstrainos_opt = emec.mc_man.DetFinSSC(
                mdf=messu, YM=YM_pref_opt, 
                iS=VIP_dicu['F3'], iLE=None,
                StressN='Stress', StrainN=dic_used_Strain, 
                addzero=True, izero=VIP_messu['S']
                )
            messu_FP=messu_FP.join(tmp, how='outer', lsuffix='', rsuffix='_opt')
            messu_FP['Stress'].fillna(messu_FP['Stress_opt'],inplace=True)
            yield_df_opt[dic_used_Strain]=yield_df_opt[dic_used_Strain]-linstrainos_opt
            del messu_FP['Stress_opt']
            yield_df_opt['Force']=yield_df_opt['Stress']*Area
            yield_df_opt[dic_used_Disp]=yield_df_opt[dic_used_Strain]*Length
            log_custom("\n   Strain offset (optical) about %.5f"%(linstrainos_opt),
                       **log_scopt)
    
        messu_FP['Force']=messu_FP['Stress']*Area # recalc Force (should match messu)
        messu_FP['Way']=messu_FP['Strain']*Length # recalc Way  
        if _opts['OPT_DIC']: messu_FP[dic_used_Disp]=messu_FP[dic_used_Strain]*Length
    else:
        messu_FP =  messu
        linstrainos_con = 0
        linstrainos_opt = 0
        log_custom("\n   No linear start of final stress-strain-curve",
                  **log_scopt)
        
    # ============================================================================
    #%% 7 Outputs
    log_custom("\n "+"="*100,**log_scopt)
    log_custom("\n ### 7 Outputs ###",**log_scoptf)
    timings.loc[7.0]=time.perf_counter()
    log_custom("\n   Timing %f: %.5f s"%(timings.index[-1],
                                       timings.iloc[-1]-timings.iloc[0]),
               **log_scopt)    
    # ============================================================================
    #%%% 7.1 Prepare outputs
    timings.loc[7.1]=time.perf_counter()
    log_custom("\n   Timing %f: %.5f s"%(timings.index[-1],
                                       timings.iloc[-1]-timings.iloc[0]),
               **log_scopt)    

    out_tab               = pd.Series([],name=prot_ser.name,dtype='float64')
    out_tab['Date_eva']   = datetime.now().strftime('%d.%m.%Y')
    out_tab['leos_con']   = linstrainos_con     
    if _opts['OPT_DIC']: out_tab['leos_opt']   = linstrainos_opt   
    
    if _opts['OPT_DIC']:
        relVS = [*np.sort([*strain_osd.keys()]),'U','B']
        tmp=emec.output.Otvalgetter_Multi(
            messu_FP, Vs=relVS,
            datasep=['con','opt'], 
            VIPs={'con':VIP_messu,'opt':VIP_dicu}, 
            exacts={'con':yield_df_con,'opt':yield_df_opt}, 
            orders={'con':['f','e','U'],'opt':['e','U']}, 
            add_esufs={'con':'_con','opt':'_opt'},
            n_strains={'con':'Strain','opt':dic_used_Strain}, 
            n_stresss={'con':'Stress','opt':'Stress'},
            use_exacts=True
            )
        tmp.name=prot_ser.name
        out_tab=out_tab.append(tmp)
        tmp=emec.output.Otvalgetter_Multi(
            messu_FP, Vs=relVS,
            datasep=['con','opt'], 
            VIPs={'con':VIP_messu,'opt':VIP_dicu}, 
            exacts={'con':yield_df_con,'opt':yield_df_opt}, 
            orders={'con':['F','s','W'],'opt':['s','W']}, 
            add_esufs={'con':'_con','opt':'_opt'},
            n_strains={'con':'Way','opt': dic_used_Disp}, 
            n_stresss={'con':'Force','opt':'Force'},
            use_exacts=True
            )
        tmp.name=prot_ser.name
        out_tab=out_tab.append(tmp)
    else:
        relVS = [*np.sort([*strain_osd.keys()]),'U','B']
        tmp=emec.output.Otvalgetter_Multi(
            messu_FP, Vs=relVS,
            datasep=['con'], 
            VIPs={'con':VIP_messu}, 
            exacts={'con':yield_df_con}, 
            orders={'con':['f','e','U']}, 
            add_esufs={'con':'_con'},
            n_strains={'con':'Strain'}, 
            n_stresss={'con':'Stress'},
            use_exacts=True
            )
        tmp.name=prot_ser.name
        out_tab=out_tab.append(tmp)
        tmp=emec.output.Otvalgetter_Multi(
            messu_FP, Vs=relVS,
            datasep=['con'], 
            VIPs={'con':VIP_messu}, 
            exacts={'con':yield_df_con}, 
            orders={'con':['F','s','W']}, 
            add_esufs={'con':'_con'},
            n_strains={'con':'Way'}, 
            n_stresss={'con':'Force'},
            use_exacts=True
            )
        tmp.name=prot_ser.name
        out_tab=out_tab.append(tmp)
        
    if _opts['OPT_pre_load_cycles'] > 0:
        ind = VIP_messu[VIP_messu.index.str.endswith('+')]
        out_tab['cyc_f_lo']   = pd.Series(messu.Stress.loc[ind]).reset_index(drop=True).mean()
        ind = VIP_messu[VIP_messu.index.str.endswith('-')]
        out_tab['cyc_f_hi']   = pd.Series(messu.Stress.loc[ind]).reset_index(drop=True).mean()
    
        out_tab['cyc_comp_E_con_first']   = df_Ee.loc['F','E_asc']/df_Ee.loc['C1','E_asc']
        out_tab['cyc_comp_E_con_rest']    = df_Ee.loc['F','E_asc']/df_Ee['E_asc'].iloc[1:-1].mean()
        out_tab['cyc_comp_epl_con_first'] = df_Ee.loc['C10','e_pl']/df_Ee.loc['C1','e_pl']
        out_tab['cyc_comp_epl_con_rest']  = df_Ee.loc['C10','e_pl']/df_Ee['e_pl'].iloc[1:-2].mean()
    # ============================================================================
    #%%% 7.2 Generate plots
    timings.loc[7.2]=time.perf_counter()
    log_custom("\n   Timing %f: %.5f s"%(timings.index[-1],
                                       timings.iloc[-1]-timings.iloc[0]),
               **log_scopt)    
        
    fig, ax1 = plt.subplots()
    ax1.set_title('%s - Analyzing meas. force'%plt_name)
    color1 = 'tab:red'
    ax1.set_xlabel('Time / s')
    ax1.set_ylabel('Force / N', color=color1)
    ax1.plot(messu.Time, messu.Force, 'r-', label='Force')
    a, b=messu.Time[VIP_messu],messu.Force[VIP_messu]
    j=np.int64(-1)
    ax1.plot(a, b, 'bx')
    for x in VIP_messu.index:
        j+=1
        if j%2: c=(6,-6)
        else:   c=(-6,6)
        ax1.annotate('%s' % x, xy=(a.iloc[j],b.iloc[j]), xycoords='data',
                     xytext=c, ha="center", va="center", textcoords='offset points')
    ax1.tick_params(axis='y', labelcolor=color1)
    ax2 = ax1.twinx()  # instantiate a second axes that shares the same x-axis
    color2 = 'tab:blue'
    ax2.grid(False)
    ax2.set_ylabel('Rise / curve /  (N/s)', color=color2)
    ax2.plot(messu.Time, messu.driF, 'b:', label='Force-rise')
    ax2.plot(messu.Time, messu.dcuF, 'g:', label='Force-curve')
    ax2.tick_params(axis='y', labelcolor=color2)
    fig.legend(loc='lower right', bbox_to_anchor=(0.85, 0.15))
    plt_hsuf(fig,path=out_full+"-Fdricu",**plt_scopt)
    
    fig, ax1 = plt.subplots()
    ax1.set_title('%s - Stress vs. strain curve with labels'%plt_name)
    ax1.set_xlabel('Strain / -')
    ax1.set_ylabel('Stress / MPa')
    ind_E='E'
    ax1.plot(messu.loc[:VIP_messu[ind_E]]['Strain'],
             messu.loc[:VIP_messu[ind_E]]['Stress'], 'r--',label='con')
    if _opts['OPT_DIC']:
        ax1.plot(messu.loc[:VIP_messu[ind_E]][dic_used_Strain],
                 messu.loc[:VIP_messu[ind_E]]['Stress'], 'm--',label='opt')
    a,b = emec.fitting.stress_linfit_plt(
        messu['Strain'], VIP_messu[['F3','F4']], *YM_pref_con[['E','E_abs']]
        )
    ax1.plot(a, b, 'g-',label='$E_{con}$')
    VIP_mwoC=VIP_messu[np.invert(VIP_messu.index.str.contains('C'))]
    a, b=messu.Strain[VIP_mwoC[:ind_E]],messu.Stress[VIP_mwoC[:ind_E]]
    j=np.int64(-1)
    ax1.plot(a, b, 'bx')
    for x in VIP_mwoC[:ind_E].index:
        j+=1
        if j%2: c=(6,-6)
        else:   c=(-6,6)
        ax1.annotate('%s' % x, xy=(a.iloc[j],b.iloc[j]), xycoords='data',
                      xytext=c, ha="center", va="center", textcoords='offset points')
    if _opts['OPT_DIC']:
        a,b=messu.loc[VIP_dicu[:'SE'],dic_used_Strain],messu.Stress[VIP_dicu[:'SE']]
        j=np.int64(-1)
        ax1.plot(a, b, 'yx')
        a,b = emec.fitting.stress_linfit_plt(
            messu[dic_used_Strain], VIP_dicu[['F3','F4']], *YM_pref_opt[['E','E_abs']]
            )
        ax1.plot(a, b, 'b-',label='$E_{opt}$')
    ftxt=('$f_{y}$ = % 6.3f MPa ($\epsilon_{y}$ = %4.3f %%)'%(
            out_tab['fy'],out_tab['ey_con']*100
            ),
          '$f_{u}$ = % 6.3f MPa ($\epsilon_{u}$ = %4.3f %%)'%(
            out_tab['fu'],out_tab['eu_con']*100
            ),
          '$E_{con}$ = % 8.3f MPa ($R²$ = %4.3f)'%(
            *YM_pref_con[['E','Rquad']],
            )
          )    
    fig.text(0.95,0.15,'\n'.join(ftxt),
              ha='right',va='bottom', 
              bbox=dict(boxstyle='round', edgecolor='0.8', 
                        facecolor='white', alpha=0.8))
    fig.legend(loc='upper left', bbox_to_anchor=(0.10, 0.91))
    plt_hsuf(fig,path=out_full+"-sigeps_wl",**plt_scopt)    

    fig, ax1 = plt.subplots()
    ax1.set_title('%s - Youngs-modulus and res. strain vs. cycles'%plt_name)
    color1 = 'tab:red'
    ax1.set_xlabel('Cycle [-]')
    ax1.set_ylabel('E [MPa]', color=color1)
    lns =ax1.plot(df_Ee.index, df_Ee.E_asc, 'rx--', label='$E_{con,load}$')
    lns+=ax1.plot(df_Ee.index, df_Ee.E_des, 'g+--', label='$E_{con,unload}$')
    # if _opts['OPT_DIC']:
        # lns+=ax1.plot(E_Dy.index, E_Dy, 'g+--', label='E-DIC-y')
        # lns+=ax1.plot(E_D1.index, E_D1, 'm+--', label='E-DIC-1')
    ax1.tick_params(axis='y', labelcolor=color1)
    ax2 = ax1.twinx()  # instantiate a second axes that shares the same x-axis
    color2 = 'tab:blue'
    ax2.set_ylabel('Plastic strain [-]', color=color2)
    lns+=ax2.plot(df_Ee.index, df_Ee.e_pl, 'bo:', label='$\epsilon_{con,pl}$')
    ax2.tick_params(axis='y', labelcolor=color2)
    ax2.grid(False)
    ax1.grid(axis='x')
    labs=[l.get_label() for l in lns]
    ax2.legend(lns,labs,loc='center right',ncol=2)
    plt_hsuf(fig,path=out_full+"-Eepszyk",**plt_scopt)
    
    fig, ax1 = plt.subplots()
    ax1.set_title('%s - Stress vs. strain curve, final part, with labels'%plt_name)
    ax1.set_xlabel('Strain [-]')
    ax1.set_ylabel('Stress [MPa]')
    ax1.plot(messu_FP['Strain'], messu_FP['Stress'], 'r--', label='meas. curve')
    a,b = emec.fitting.stress_linfit_plt(
        messu_FP['Strain'], VIP_messu[['F3','F4']], YM_pref_con['E'],0
        )
    ax1.plot(a, b, 'g-',label='$E_{con}$')
    # tmp=['g-','y-','m-']
    # j=0
    # for i in strain_osd.keys():
    #     a = Evac.strain_linfit(messu_FP['Stress'], YM=YM_pref_con['E'],
    #                            YM_abs=0, strain_offset=strain_osd[i])
    #     # ax1.plot(a, messu_FP['Stress'], tmp[j],label='$E_{con-%s}$'%i)   
    #     ax1.plot(a, messu_FP['Stress'], label='$E_{con-%s}$'%i)   
    #     j += 1
    a, b=messu_FP.Strain[VIP_messu[FP_sta:FP_end]],messu_FP.Stress[VIP_messu[FP_sta:FP_end]]
    j=np.int64(-1)
    ax1.plot(a, b, 'bx')
    for x in VIP_messu[FP_sta:FP_end].index:
        j+=1
        if j%2: c=(6,-6)
        else:   c=(-6,6)
        ax1.annotate('%s' % x, xy=(a.iloc[j],b.iloc[j]), xycoords='data', 
                     xytext=c, ha="center", va="center", textcoords='offset points')
    if _opts['OPT_DIC']:
        tmp=VIP_dicu[['S','F3',*np.sort([*strain_osd.keys()]),'U']].sort_values()
        if 'B' in VIP_dicu.index: tmp=tmp.append(VIP_dicu[['B']])
        tmp=tmp.append(VIP_dicu[['E']])
        tmp=tmp.sort_values()
        a,b=messu_FP.loc[tmp,dic_used_Strain],messu_FP.Stress[tmp]
        j=np.int64(-1)
        ax1.plot(a, b, 'yx')
        a,b = emec.fitting.stress_linfit_plt(
            messu_FP[dic_used_Strain], VIP_dicu[['F3','F4']], 
            YM_pref_opt['E'], 0
            )
        ax1.plot(a, b, 'b-',label='$E_{opt}$')
    ftxt=('$f_{y}$ = %3.3f MPa ($\epsilon_{y}$ = %.3f %%)'%(out_tab['fy'],out_tab['ey_con']*100),
          '$f_{u}$ = %3.3f MPa ($\epsilon_{u}$ = %.3f %%)'%(out_tab['fu'],out_tab['eu_con']*100),
          '$E_{con}$ = % 8.3f MPa ($R²$ = %4.3f)'%(*YM_pref_con[['E','Rquad']],))
    fig.text(0.95,0.15,'\n'.join(ftxt),
             ha='right',va='bottom', bbox=dict(boxstyle='round', edgecolor='0.8', facecolor='white', alpha=0.8))
    fig.legend(loc='upper left', bbox_to_anchor=(0.10, 0.91))
    plt_hsuf(fig,path=out_full+"-sigeps_fin",**plt_scopt)

    # =============================================================================
    #%%% 7.3 Generate outputs
    timings.loc[7.3]=time.perf_counter()
    log_custom("\n   Timing %f: %.5f s"%(timings.index[-1],
                                       timings.iloc[-1]-timings.iloc[0]),
               **log_scopt)    

    # Federsteifigkeit
    for i in E_lsq.loc(axis=1)[E_lsq.columns.str.contains('_F_|_R_')].columns:
        nvar=i.replace('E','D')
        out_tab[nvar]=E_lsq.loc['E',i]*prot_ser['Area_CS']/prot_ser['Length_test']
    
    t=E_lsq.loc[['E','Rquad']].T.stack()
    t.index=t.index.to_series().apply(lambda x: '{0}_{1}'.format(*x)).values
    t.name=prot_ser.name
    out_tab=pd.concat([out_tab,t])
    
    t=E_inc_F_comp.T.stack()
    t.index=t.index.to_series().apply(lambda x: '{0}_{1}'.format(*x)).values
    t.name=prot_ser.name
    t=t.add_prefix('E_inc_F_')
    out_tab=pd.concat([out_tab,t])

    t=E_inc_R_comp.T.stack()
    t.index=t.index.to_series().apply(lambda x: '{0}_{1}'.format(*x)).values
    t.name=prot_ser.name
    t=t.add_prefix('E_inc_R_')
    out_tab=pd.concat([out_tab,t])
    
    t=df_Ee['e_pl'][:-1]
    t.name=prot_ser.name
    t=t.add_prefix('epl_con_')
    out_tab=pd.concat([out_tab,t])
   
    out_tab=pd.DataFrame([out_tab])

    vnew=pd.Series(VIP_messu.drop_duplicates().index.values,
                   index=VIP_messu.drop_duplicates(),dtype='O')
    VIP_messu=VIP_messu.sort_values()
    for i in VIP_messu.index:
        if VIP_messu.duplicated().loc[i]:
            vnew.loc[VIP_messu.loc[i]]=vnew.loc[VIP_messu.loc[i]]+','+i
    messu['VIP_m']=vnew
    messu.VIP_m.fillna('', inplace=True)
    
    if _opts['OPT_DIC']:
        vnew=pd.Series(VIP_dicu.drop_duplicates().index.values,
                       index=VIP_dicu.drop_duplicates(),dtype='O')
        VIP_dicu=VIP_dicu.sort_values()
        for i in VIP_dicu.index:
            if VIP_dicu.duplicated().loc[i]:
                vnew.loc[VIP_dicu.loc[i]]=vnew.loc[VIP_dicu.loc[i]]+','+i
        messu['VIP_d']=vnew
        messu.VIP_d.fillna('', inplace=True)
    
    messu.to_csv(out_full+'.csv',sep=';')   
    out_tab.to_csv(out_full+'-Mat.csv',sep=';')
    
    HDFst=pd.HDFStore(out_full+'.h5')
    HDFst['Measurement'] = messu
    HDFst['Measurement_FP'] = messu_FP
    HDFst['Material_Parameters'] = out_tab
    HDFst['Timings'] = timings
    HDFst['Options'] = _opts
    if _opts['OPT_DIC']:
        HDFst['VIP'] = pd.concat([VIP_messu,VIP_dicu],axis=1)
    else:
        HDFst['VIP'] = pd.concat([VIP_messu],axis=1)
        
    HDFst['Exact_VIP_con']=yield_df_con
    if _opts['OPT_DIC']: HDFst['Exact_VIP_opt']=yield_df_opt
        
    HDFst['DQcon'] = DQcons
    HDFst['E_lsq'] = E_lsq
    HDFst['E_inc_df'] = E_Methods_df
    HDFst['E_inc'] = pd.concat([E_inc_F_comp.add_prefix('E_inc_F_'),
                                E_inc_R_comp.add_prefix('E_inc_R_')],axis=1)
    
    HDFst.close()
    
    timings.loc[10.0]=time.perf_counter()
    if log_scopt['output_lvl']>=1: 
        log_scopt['logfp'].close()
        log_scopt['logfp'] = None # workaround (Error _io.TextIOWrapper while series run)
        
    return timings,cout
```

---

© Copyright 2024, MarcGebhardt.

Built with Sphinx using a
theme
provided by Read the Docs.
