# Supplementary material for: Approach to Standardized Material Characterization of the Human Lumbopelvic System: Testing and Evaluation
Source: Bioengineering (Basel). 2025 Aug 11;12(8):862. doi: 10.3390/bioengineering12080862 (PMC12383908; doi:10.3390/bioengineering12080862)
Supplement: Supplementary file 1 [file bioengineering-12-00862-s001.zip › File S3 Evaluation code/ExMechEva-0.1.2/docs/_build/html/_modules/exmecheva/Eva_TBT.html]

exmecheva.Eva\_TBT — ExMechEva v0.1.1 documentation


ExMechEva

Contents:

- ExMechEva

ExMechEva

- Module code
- exmecheva.Eva\_TBT

---

# Source code for exmecheva.Eva\_TBT

```
# -*- coding: utf-8 -*-
"""
Three point bending test evaluation.

@author: MarcGebhardt
"""

#%% 0 Imports
import pandas as pd
import numpy as np
import lmfit
import matplotlib.pyplot as plt
from datetime import datetime
import time
import warnings

import exmecheva.common as emec
import exmecheva.bending as emeb

warnings.filterwarnings('ignore',category=pd.io.pytables.PerformanceWarning)
warnings.filterwarnings('ignore',category=FutureWarning)

log_custom = emec.output.str_log
log_cind = emec.output.str_indent

plt_hsuf =  emec.plotting.plt_handle_suffix
figsize = plt.rcParams['figure.figsize']

#%% 1.0 Evaluation

[docs]def TBT_single(prot_ser, paths, mfile_add='',
               log_scopt={'logfp':None, 'output_lvl': 1,
                          'logopt':True, 'printopt':False},
               plt_scopt={'tight':True, 'show':True, 
                          'save':True, 's_types':["pdf"], 
                          'clear':True, 'close':True}):
    """
    Evaluate single three point bending measurement form protocol table and 
    path collection. Using common options wich are overwritten by protocol 
    variables starting with 'OPT_'. Produce evaluated measurements, 
    material parameters and plots and safe them as table (.csv), unstructured 
    database (.h5) and document (.pdf), using the designation of the specimen 
    for distinction.
    Procedure:
        - 1: Read in options and presetting
        - 2: Determining geometrical values
        - 3: Read in measurements (conventional and optical (optinal))
        - 4: Merging measurements (time offset of conventional to optical 
                                   (if available), downsampling and merging)
        - 5: Determine evaluation space (start and end)
        - 6: Evaluation (curves (stress/strain), importent points on curves,
                         elastic moduli (different types implemented))
        - 7: Generating output (tables, database and plots)

    Parameters
    ----------
    prot_ser : pd.Series
        Input data as pandas series with specimen information 
        (identifier, origin, geometrical data, 
         assessment codes, evaluation options).
    paths : pd.DataFrame
        Path collection for in- and output paths. 
        Needs indexes: 
            - "opts": Common evaluation options
            - "prot": Protocol
            - "meas": Conventional measured data
            - "dic": Optical measured data
            - "out": Output
    mfile_add : string, optional
        Suffix of variants of measurements 
        (p.E. diffferent moistures ["A","B",...]). 
        The default is ''.
    log_scopt : dict, optional
        Options for custom logging. Determining file path, 
        output level (0=none, 1=normal, 2=special), logging enabled and 
        printing enabled.
        The default is {'logfp':None, 'output_lvl': 1,
                        'logopt':True, 'printopt':False}.
    plt_scopt : dict, optional
        Options for plotting. 
        The default is {'tight':True, 'show':True, 
                        'save':True, 's_types':["pdf"],
                        'clear':True, 'close':True}.

    Raises
    ------
    ValueError
        Input value not correct.
    NotImplementedError
        Method not implemented.

    Yields
    ------
    timings : pd.Series
        Timings of procedure steps.
    cout : string
        Special text output for control purposes.

    """
    out_name = prot_ser['Designation']+mfile_add
    out_full = paths['out']+out_name
    if log_scopt['output_lvl']>=1: 
        if log_scopt['logfp'] is None or (not isinstance(log_scopt['logfp'],str)):
            log_scopt['logfp'] = out_full+'.log'
        log_scopt['logfp']=open(log_scopt['logfp'],'w')
    log_scoptf={'logfp':log_scopt['logfp'], 
                'output_lvl': log_scopt['output_lvl'], 
                'logopt':log_scopt['logopt'], 
                'printopt':True}
    
    _opts=emec.eva_opt_hand.option_reader_sel(
        prot_ser=prot_ser, paths=paths, 
        search_inds=['Number','Designation','name'], 
        variant='',
        option='JFile+Prot',
        sheet_name="",
        re_rkws=dict()
        )
    
    path_meas = paths['meas']+_opts['OPT_File_Meas']+mfile_add+".xlsx"
    path_dic = paths['dic']+_opts['OPT_File_DIC']+mfile_add+".csv"
    
    plt_name = prot_ser['Designation']+mfile_add
    timings=pd.Series([],dtype='float64')
    timings.loc[0.0]=time.perf_counter()
    
    Length = prot_ser['Length_test']
    Bo_Le = -Length/2
    Bo_Ri =  Length/2
    xlin = np.linspace(-Length/2,Length/2,101)
    
    # rel_time_digs = emec.helper.sigdig(_opts['OPT_Resampling_Frequency'], 4)
    rel_time_digs = 2
    
    bl = emeb.bfunc_class.Bend_func_legion(
        name=_opts['OPT_DIC_Fitting']['Bending_Legion_Name']
        )
    bl.Builder(option=_opts['OPT_DIC_Fitting']['Bending_Legion_Builder'])
    
    pwargs=_opts['OPT_DIC_Fitting']['pwargs']
    # krücke
    pwargs['param_val']['xmin']=Bo_Le
    pwargs['param_val']['xmax']=Bo_Ri
        
    dic_used_Strain="Strain_opt_"+_opts['OPT_YM_Determination_refinement'][3]
    tmp_in = _opts['OPT_YM_Determination_refinement'][3].split('_')[-1]
    dic_used_Disp="Disp_opt_"+tmp_in
    
    if _opts['OPT_YM_Determination_refinement'][3].split('_')[-2] == 'd':
        tmp_md = '2'
    elif _opts['OPT_YM_Determination_refinement'][3].split('_')[-2] == 'c':
        tmp_md = '4'
    else:
        raise ValueError('OPT_YM_Determination_refinement seams to be wrong')
    # loc_Yd_tmp = 'E_lsq_R_A%s%sl'%(tmp_md,tmp_in)
    loc_Yd_tmp = 'E_inc_R_D%s%sgwt'%(tmp_md,tmp_in)
            
    cout =''
    ftxt=(("  Parameters of evaluation:"),
          ("   Evaluation start time:     %s" %datetime.now().strftime('%Y-%m-%d %H:%M:%S')),
          ("   Evaluation options:        %s" %paths['opts']),
          ("   Path protocol:             %s" %paths['prot']),
          ("   Path measurement:          %s" %path_meas),
          ("   Path optical- measurement: %s" %path_dic),
          ("   Resampling = %s (Frequency = %d Hz, moving-average = %s)" %(
              _opts['OPT_Resampling'],_opts['OPT_Resampling_Frequency'],
              _opts['OPT_Resampling_moveave']
              )),
          ("   Compression = %s" %(_opts['OPT_Compression'])),
          ("   LVDT failure = %s" %(_opts['OPT_LVDT_failure'])),
          ("   LVDT-spring-force-reduction = %s (K_Fed = %f N/mm)" %(
              _opts['OPT_Springreduction'],_opts['OPT_Springreduction_K']
              )),
          ("   Rise and curvature smoothing = %s (window = %d)" %(
              _opts['OPT_Rise_Smoothing'][0],_opts['OPT_Rise_Smoothing'][1]
              )),
          ("   Youngs Modulus determination between %f and %f of point %s" %(
              *_opts['OPT_YM_Determination_range'],
              )),
          ("   Distance between points: %d steps " %_opts['OPT_Determination_Distance']),
          ("   Improvment of Youngs-Modulus-determination between %f*Stress_max and point %s," %(
              _opts['OPT_YM_Determination_refinement'][0],
              _opts['OPT_YM_Determination_refinement'][2]
              )),
          ("    with smoothing on difference-quotient (%s, %d)" %(
              _opts['OPT_YM_Determination_refinement'][4],
              _opts['OPT_YM_Determination_refinement'][5]
              )),
          ("    with allowable deviation of %f * difference-quotient_max in determination range" %(
              _opts['OPT_YM_Determination_refinement'][1]
              )),
          ("   DIC-Measurement = %s" %(_opts['OPT_DIC'])),
          ("   DIC-Strain-suffix for range refinement and plots = %s" %(
              _opts['OPT_YM_Determination_refinement'][3]
              )),
          ("   DIC-minimal points (special / specimen) = %d / %d" %(
              _opts['OPT_DIC_Tester'][-2],_opts['OPT_DIC_Tester'][-1]
              )),
          ("   DIC-names of special points (l,r,head), = %s, %s, %s" %(
              *_opts['OPT_DIC_Points_TBT_device'],
              )),
          ("   DIC-names of meas. points for fork (l,m,r), = %s, %s, %s" %(
              *_opts['OPT_DIC_Points_meas_fork'],
              )),
          ("   DIC-maximal SD = %.3f mm and maximal displacement between steps %.1f mm" %(
              _opts['OPT_DIC_Tester'][0],_opts['OPT_DIC_Tester'][1]
              )))
    log_custom('\n'.join(ftxt),**log_scopt)
    
    #%% 2 Geometry
    log_custom("\n "+"="*100,**log_scopt)
    log_custom("\n ### 2 Geometry ###",**log_scoptf)
    timings.loc[2.0]=time.perf_counter()
    log_custom("\n   Timing %f: %.5f s"%(timings.index[-1],
                                       timings.iloc[-1]-timings.iloc[0]),
                   **log_scopt)
    
    geo = pd.DataFrame({'x': [Bo_Le, 0, Bo_Ri],
                        't': [prot_ser['thickness_1'], prot_ser['thickness_2'], prot_ser['thickness_3']],
                        'w': [prot_ser['width_1'], prot_ser['width_2'], prot_ser['width_3']]}) # Geometrie auf x-Koordinaten bezogen
    
    func_t = np.poly1d(np.polyfit(geo.loc[:,'x'], geo.loc[:,'t'],2), variable='x') # Polynom 2'ter Ordnung für Dicke über x-Koordinate
    func_w = np.poly1d(np.polyfit(geo.loc[:,'x'], geo.loc[:,'w'],2), variable='x') # Polynom 2'ter Ordnung für Breite über x-Koordinate
    func_A = func_t * func_w # Polynom für Querschnitt über x-Koordinate
    func_I = func_t**3*func_w/12 # Polynom für Flächenträgheitsmoment 2'ten Grades über x-Koordinate
    
    gamma_V=emeb.bfunc_com.gamma_V_det(
        _opts['OPT_Poisson_prediction'], geo['t'].mean(),
        Length, CS_type=prot_ser['CS_type']
        )
    
    if True:
        fig, (ax1,ax3) = plt.subplots(nrows=2, ncols=1, sharex=False, sharey=False, 
                                      figsize = np.multiply(figsize,[1,2]))
        ax1.set_title('%s - Width and Thickness'%plt_name)
        ax1.set_xlabel('x / mm')
        ax1.set_ylabel('Thickness / mm')
        lns=ax1.plot(xlin, func_t(xlin), 'b-', label = 'Thickness-fit')
        lns+=ax1.plot(geo.loc[:,'x'],geo.loc[:,'t'], 'bs', label = 'Thickness')
        ax2 = ax1.twinx() 
        ax2.grid(False)
        ax2.set_ylabel('Width / mm')
        lns+=ax2.plot(xlin, func_w(xlin), 'r-', label = 'Width-fit')
        lns+=ax2.plot(geo.loc[:,'x'],geo.loc[:,'w'], 'ro', label = 'Width')
        labs = [l.get_label() for l in lns]
        ax1.legend(lns, labs)
        ax3.set_title('%s - Moment of Inertia'%plt_name)
        ax3.set_xlabel('x / mm')
        ax3.set_ylabel('MoI / mm^4')
        ax3.plot(xlin, func_I(xlin), 'g-', label = 'MoI-fit')
        ax3.plot(geo.loc[:,'x'],geo.loc[:,'t']**3*geo.loc[:,'w']/12, 'gh', label = 'MoI')
        ax3.legend()
        plt_hsuf(fig,path=out_full+"-Geo",**plt_scopt)
    
    log_custom("\n  Measured Geometry:"+log_cind(geo),
               **log_scopt)
    log_custom("\n  Function of thickness:"+log_cind(func_t),
               **log_scopt)
    log_custom("\n  Function of width:"+log_cind(func_w),
               **log_scopt)
    log_custom("\n  Function of area:"+log_cind(func_A),
               **log_scopt)
    log_custom("\n  Function of moment of inertia:"+log_cind(func_I),
            **log_scopt)

    # =============================================================================
    #%% 3 Read in measurements
    log_custom("\n "+"="*100,**log_scopt)
    log_custom("\n ### 3 Read in measurements ###",**log_scoptf)
    timings.loc[3.0]=time.perf_counter()
    log_custom("\n   Timing %f: %.5f s"%(timings.index[-1],
                                       timings.iloc[-1]-timings.iloc[0]),
                   **log_scopt)
    # =============================================================================
    #%%% 3.1 Read in conventional measurement data
    timings.loc[3.1]=time.perf_counter()
    log_custom("\n   Timing %f: %.5f s"%(timings.index[-1],
                                       timings.iloc[-1]-timings.iloc[0]),
                   **log_scopt)
    mess=pd.read_excel(
        path_meas, header=_opts['OPT_Measurement_file']['header'],
        names=_opts['OPT_Measurement_file']['head_names']
        )
    
    mess = mess-mess.iloc[0]
    
    if isinstance(_opts['OPT_Measurement_file']['used_names_dict']['Way'],type(list())):
        mess['L_IWA']=pd.Series(
            mess[_opts['OPT_Measurement_file']['used_names_dict']['Way']].mean(axis=1)
            )
        # automatic number of LVDTs by list elements in used way
        n_IWAs=len(_opts['OPT_Measurement_file']['used_names_dict']['Way'])
        _opts['OPT_Measurement_file']['used_names_dict']['Way'] ='L_IWA'
    #Applying spring correction factor (if true)
    if _opts['OPT_Springreduction']: 
        mess['F_IWA_red']=mess['L_IWA']*_opts['OPT_Springreduction_K']*n_IWAs
        
    # # =============================================================================
    # #%%% 3.2 Specify used conventional measured force and way
    timings.loc[3.2]=time.perf_counter()
    log_custom("\n   Timing %f: %.5f s"%(timings.index[-1],
                                       timings.iloc[-1]-timings.iloc[0]),
                   **log_scopt)
    messu = pd.DataFrame({
        'Time': mess[_opts['OPT_Measurement_file']['used_names_dict']['Time']],
        'Force': mess[_opts['OPT_Measurement_file']['used_names_dict']['Force']],
        'Way': mess[_opts['OPT_Measurement_file']['used_names_dict']['Way']]
        })
    
    if _opts['OPT_Springreduction']:     
        messu['Force'] = messu['Force'] - mess['F_IWA_red']
        
    if _opts['OPT_Compression']==True:
        messu.Force=messu.Force*(-1)
    if np.invert(np.isnan(_opts['OPT_End'])):
        messu=messu.loc[messu.Time.round(rel_time_digs) <= _opts['OPT_End']]
    
    # =============================================================================
    #%%% 3.3 Read in optical measurement data
    timings.loc[3.3]=time.perf_counter()
    log_custom("\n   Timing %f: %.5f s"%(timings.index[-1],
                                       timings.iloc[-1]-timings.iloc[0]),
                   **log_scopt)
    if _opts['OPT_DIC']:
        dic = pd.read_csv(path_dic,
                          sep=";",index_col=0,skiprows=[0,1],usecols=[0,1],
                          names=['DStep','Time'], na_values={"Nan"," Nan","nan"})
        dic.index=pd.Int64Index(dic.index)
        if np.invert(np.isnan(_opts['OPT_End'])):
            dic = dic.loc[dic.Time <= _opts['OPT_End']]
    
        dic_dt = dic.Time.diff().mean()
        dic_f  = round(1/dic_dt)
        
        
        dic_tmp=pd.read_csv(path_dic,
                            sep=";",index_col=[0,1],header=[0,1],
                            na_values={"Nan"," Nan","nan"})
        dic_tmp.index.names=['Step','Time']
        dic_tmp.columns.names=['Points','Vars']
        dic_tmp=dic_tmp.reset_index()
        dic_tmp=dic_tmp.drop(columns=['Step','Time'])
        dic_tmp = dic_tmp.sort_index(axis=1)
        if np.invert(np.isnan(_opts['OPT_End'])):
            dic_tmp=dic_tmp.loc[:dic.index[-1]]
    
        
        testert={}
        testert[0]=(dic_tmp.loc(axis=1)[pd.IndexSlice[:, ['Sx','Sy','Sz']]].abs()>=_opts['OPT_DIC_Tester'][0]).any(axis=1,level=0) # Test, ob eine Standrardabweichung größer als die Grenzstd. ist
        testert[1]=(dic_tmp.loc(axis=1)[pd.IndexSlice[:, ['Sx','Sy','Sz']]].abs()==0                         ).any(axis=1,level=0) # Test, ob eine Standrardabweichung gleich 0 ist
        testert[2]=(dic_tmp.loc(axis=1)[pd.IndexSlice[:, ['Sx','Sy','Sz']]].isna()                           ).any(axis=1,level=0) # Test, ob eine Standrardabweichung NaN ist
        testert[3]=(dic_tmp.loc(axis=1)[pd.IndexSlice[:, ['Sx','Sy','Sz']]].std(axis=1,level=0)<=1e-06       ).any(axis=1,level=0) # Test, ob die Standrardabweichungen eines Punktes weniger als 1e-06 von einander abweichen (faktisch gleich sind)
        testert[4]=(dic_tmp.loc(axis=1)[pd.IndexSlice[:,    ['x','y','z']]].abs()>=_opts['OPT_DIC_Tester'][1]).any(axis=1,level=0) # Test, ob die erhaltenen Koordinaten im Bereich der erwarteten liegen
        vt=((dic_tmp.diff().loc(axis=1)[pd.IndexSlice[:,    ['x','y','z']]]**2).sum(axis=1,level=0))**0.5 # Verschiebung zwischen einzelnen Epochen
        testert[5]=(vt>=vt.median()*200) # Test ob die Verschiebung zwischen einzelnen Epochen größer als das 200-fache ihres Medians sind

        # testert[0]=(emeb.opt_mps.Point_df_idx(df=dic_tmp, coords= ['Sx','Sy','Sz']).abs()>=_opts['OPT_DIC_Tester'][0]).any(axis=1,level=0) # Test, ob eine Standrardabweichung größer als die Grenzstd. ist
        # testert[1]=(emeb.opt_mps.Point_df_idx(df=dic_tmp, coords= ['Sx','Sy','Sz']).abs()==0                         ).any(axis=1,level=0) # Test, ob eine Standrardabweichung gleich 0 ist
        # testert[2]=(emeb.opt_mps.Point_df_idx(df=dic_tmp, coords= ['Sx','Sy','Sz']).isna()                           ).any(axis=1,level=0) # Test, ob eine Standrardabweichung NaN ist
        # testert[3]=(emeb.opt_mps.Point_df_idx(df=dic_tmp, coords= ['Sx','Sy','Sz']).std(axis=1,level=0)<=1e-06       ).any(axis=1,level=0) # Test, ob die Standrardabweichungen eines Punktes weniger als 1e-06 von einander abweichen (faktisch gleich sind)
        # testert[4]=(emeb.opt_mps.Point_df_idx(df=dic_tmp, coords= ['x','y','z']   ).abs()>=_opts['OPT_DIC_Tester'][1]).any(axis=1,level=0) # Test, ob die erhaltenen Koordinaten im Bereich der erwarteten liegen
        # vt=((emeb.opt_mps.Point_df_idx(df=dic_tmp, coords= ['x','y','z']   ).diff()**2).sum(axis=1,level=0))**0.5 # Verschiebung zwischen einzelnen Epochen
        # testert[5]=(vt>=vt.median()*200) # Test ob die Verschiebung zwischen einzelnen Epochen größer als das 200-fache ihres Medians sind
        tester=testert[0]|testert[1]|testert[2]|testert[3]|testert[4]|testert[5]
        dic_tmp[pd.IndexSlice[tester]] = np.nan
        
        step_range_dic=tester.index
        anp=tester.loc[0,tester.loc[0].index.str.contains(pat='P',na=False,regex=True)].count()
        ans=tester.loc[0,tester.loc[0].index.str.contains(pat='S',na=False,regex=True)].count()
        dic_t_str=pd.Series([],dtype='O')
        log_custom("\n "+"-"*100,**log_scopt)
        log_custom("\n  DIC-Tester:",**log_scopt)
        for s in tester.index:
            dic_t_str[s]=""
            if tester.loc[s].any():
                fpnp=tester.loc[s,tester.loc[s].index.str.contains(pat='P',na=False,regex=True)].loc[tester.loc[s]==True].count()
                fpns=tester.loc[s,tester.loc[s].index.str.contains(pat='S',na=False,regex=True)].loc[tester.loc[s]==True].count()
                if ((anp-fpnp) < _opts['OPT_DIC_Tester'][3])|((ans-fpns) < _opts['OPT_DIC_Tester'][2]): 
                    step_range_dic = step_range_dic.drop(s)
                if log_scopt['output_lvl']>=1:
                    fps=[]
                    for l in tester.loc[s].index:
                        if tester.loc[s].loc[l]:
                            fps.append(l)
                    log_custom("\n    Failed points in series %d: %d of %d meas. | %d of %d special %s" %(s,fpnp,anp,fpns,ans,fps),
                                 **log_scoptf)
                    dic_t_str[s]=dic_t_str[s]+("\n    Failed points: %d of %d meas. | %d of %d special %s" %(fpnp,anp,fpns,ans,fps))
                    if ((anp-fpnp) < _opts['OPT_DIC_Tester'][3])|((ans-fpns) < _opts['OPT_DIC_Tester'][2]): dic_t_str[s]=dic_t_str[s]+"\n    -> DIC-curving of step dropped!!!"
    
        log_custom("\n "+"-"*100,**log_scopt)
        log_custom("\n  Coordinate transformation and curvature calculation:",**log_scopt)
        
        Points_T=pd.Series([],dtype='O')
        Points_L_T=pd.Series([],dtype='O')
        for step in step_range_dic:
            log_custom("\n  Step %d coordinate transformation:" %step, **log_scopt)
            log_custom(dic_t_str[step], **log_scopt)
            dic_c_tmp = pd.DataFrame([dic_tmp.loc[step].loc[:,'x'],
                                      dic_tmp.loc[step].loc[:,'y'],
                                      dic_tmp.loc[step].loc[:,'z']],
                                     index=['x','y','z'])
            dic_Sdev_tmp = pd.DataFrame([dic_tmp.loc[step].loc[:,'Sx'],
                                         dic_tmp.loc[step].loc[:,'Sy'],
                                         dic_tmp.loc[step].loc[:,'Sz']],
                                        index=['Sx','Sy','Sz'])
    
            # Krücke, muss besser gehen:
            fps=[]
            for l in tester.loc[step].index:
                if tester.loc[step].loc[l]:
                    fps.append(l)
            dic_c_tmp = dic_c_tmp.drop(fps,axis=1)
            dic_Sdev_tmp  = dic_Sdev_tmp.drop(fps,axis=1)
            
            Pmeas      = dic_c_tmp.iloc[:,dic_c_tmp.columns.str.contains(pat=_opts['OPT_DIC_Points_meas_prefix'],na=False,regex=True)]
            Pspec      = dic_c_tmp.iloc[:,dic_c_tmp.columns.str.contains(pat=_opts['OPT_DIC_Points_device_prefix'],na=False,regex=True)]
            Pmeas_Sdev = dic_Sdev_tmp.iloc[:,dic_Sdev_tmp.columns.str.contains(pat=_opts['OPT_DIC_Points_meas_prefix'],na=False,regex=True)]
            Pspec_Sdev = dic_Sdev_tmp.iloc[:,dic_Sdev_tmp.columns.str.contains(pat=_opts['OPT_DIC_Points_device_prefix'],na=False,regex=True)]
            
            # Start 3D point transformation
            Points_T[step], Points_L_T[step] = emeb.opt_mps.Point_df_transform(
                Pmeas = Pmeas,
                Pspec = Pspec,
                Pmeas_Sdev = Pmeas_Sdev,
                Pspec_Sdev = Pspec_Sdev,
                dic_P_name_org1 = _opts['OPT_DIC_Points_TBT_device'][0],
                dic_P_name_org2 = _opts['OPT_DIC_Points_TBT_device'][1],
                output_lvl = log_scopt['output_lvl'],
                log_mg = log_scopt['logfp'])
    
        Points_L_T_stacked=dic_tmp.copy(deep=True)
        Points_L_T_stacked.loc[:]=np.nan
        Points_T_stacked=dic_tmp.copy(deep=True)
        Points_T_stacked.loc[:]=np.nan
        
        for i in step_range_dic:
            Points_L_T_stacked.loc[i]=Points_L_T[i].T.stack()
            Points_T_stacked.loc[i]=Points_T[i].T.stack()
            # Pcoord_val_m[i]=np.polyval(Pcoord[i],0)
            # Pcoord_val_lr[i]=np.polyval(Pcoord[i],[-Pcoord_val_L/2,Pcoord_val_L/2]).mean()
        
        pt=emeb.opt_mps.Point_df_idx(Points_L_T_stacked, coords=['x','y'], deepcopy=True)
        pt_S=emeb.opt_mps.Point_df_idx(pt, points='S', deepcopy=True, option='Regex')
        pt_P=emeb.opt_mps.Point_df_idx(pt, points='P', deepcopy=True, option='Regex')
        geo_fit = emeb.fitting.Perform_Fit(
            BFL=bl, Fit_func_key='w_A', P_df=pt_P,
            lB=Bo_Le, rB=Bo_Ri, s_range=[0],
            Shear_func_key='w_S', gamma_V=gamma_V, 
            err_weights=[1,0,0,0],
            max_nfev=_opts['OPT_DIC_Fitting']['Bending_MCFit_opts']['fit_max_nfev_pre'],
            option='Pre', pb_b=False,**pwargs
            ).loc[0]
        
        geo_d2_max = bl['w_A']['d2'](xlin,geo_fit.loc['Fit_params_dict']).max()
        geo_d2_min = bl['w_A']['d2'](xlin,geo_fit.loc['Fit_params_dict']).min()
        geo_d2_mid = bl['w_A']['d2'](0.0,geo_fit.loc['Fit_params_dict'])
        
        fig, ax1 = plt.subplots()
        ax1.set_title('%s - Geometry'%(plt_name))
        ax1.set_xlabel('x / mm')
        ax1.set_ylabel('y / mm')
        ax1.plot(xlin, bl['w_A']['d0'](xlin,geo_fit.loc['Fit_params_dict']), 'r:', label='Org.')
        ax1.plot(pt_P.loc[0].loc[:,'x'],pt_P.loc[0].loc[:,'y'], 'ko', label='org. P')
        ax1.plot(pt_S.loc[0].loc[:,'x'],pt_S.loc[0].loc[:,'y'], 'k+', label='org. S')
        for x in pt.columns.droplevel(1):
            ax1.annotate('%s' % x, xy=(pt.loc[0].loc[x,'x'],pt.loc[0].loc[x,'y']),
                         xycoords='data', xytext=(7, -7), textcoords='offset points')
        ax1.legend()
        plt_hsuf(fig,path=out_full+"-DIC_fit_Geo",**plt_scopt)
        
        P_xcoord_ydisp = Points_L_T_stacked.loc(axis=1)[:,['x','y']].copy()
        P_xcoord_ydisp.loc(axis=1)[:,'y'] = P_xcoord_ydisp.loc(axis=1)[:,'y']-P_xcoord_ydisp.loc(axis=1)[:,'y'].loc[0]
        P_xcoord_ydisp_meas = P_xcoord_ydisp.loc(axis=1)[P_xcoord_ydisp.columns.droplevel(level=1).str.contains(pat='P',na=False,regex=True)]
        P_xcoord_ydisp_spec = P_xcoord_ydisp.loc(axis=1)[P_xcoord_ydisp.columns.droplevel(level=1).str.contains(pat='S',na=False,regex=True)]

        dic['Disp_opt_head'] = P_xcoord_ydisp_spec.loc(axis=1)[(_opts['OPT_DIC_Points_TBT_device'][2],'y')]
        if _opts['OPT_Compression']: dic['Disp_opt_head']=dic['Disp_opt_head']*(-1)
    
    
    dicu=dic.copy(deep=True)
    # dicu=pd.DataFrame({'Time': dic.Time, 'DWay': dic.DWay, 'DStrain': dic.DL1GS, 'DStrainq': dic.DL2GS})
    del dic_tmp,anp,ans
    del dic_c_tmp,dic_Sdev_tmp
    del Pmeas,Pspec,Pmeas_Sdev,Pspec_Sdev
    del Points_T,Points_L_T
    del pt,pt_S,pt_P
    
    # =============================================================================
    #%% 4 Merging measurements
    log_custom("\n "+"="*100,**log_scopt)
    log_custom("\n ### 4 Merging measurements ###",**log_scoptf)
    timings.loc[4.0]=time.perf_counter()
    log_custom("\n   Timing %f: %.5f s"%(timings.index[-1],
                                       timings.iloc[-1]-timings.iloc[0]),
                   **log_scopt)
    # =============================================================================
    #%%% 4.1 Determine time offset between conventional and optical measurement
    timings.loc[4.1]=time.perf_counter()
    log_custom("\n   Timing %f: %.5f s"%(timings.index[-1],
                                       timings.iloc[-1]-timings.iloc[0]),
                   **log_scopt)
    if _opts['OPT_DIC']:
        mun_tmp = messu.loc[
            abs(messu.Way.loc[:(messu.Way.idxmax())]-messu.Way.max()/8).idxmin():
            abs(messu.Way.loc[:(messu.Way.idxmax())]-messu.Way.max()/4).idxmin()
            ]
        linvm   = np.polyfit(mun_tmp.loc[:,'Time'],mun_tmp.loc[:,'Way'],1)
        tsm     = -linvm[1]/linvm[0]
        mun_tmp = dicu.loc[
            abs(dicu.Disp_opt_head.loc[:(dicu.Disp_opt_head.idxmax())]-dicu.Disp_opt_head.max()/8).idxmin():
            abs(dicu.Disp_opt_head.loc[:(dicu.Disp_opt_head.idxmax())]-dicu.Disp_opt_head.max()/4).idxmin()
            ]
        linvd   = np.polyfit(mun_tmp.loc[:,'Time'],mun_tmp.loc[:,'Disp_opt_head'],1)
        tsd     = -linvd[1]/linvd[0]
        toff    = tsm-tsd
        
        if True:
            maxt_tmp=max(
                messu.Time.loc[abs(messu.Way.loc[:(messu.Way.idxmax())]-messu.Way.max()/4).idxmin()],
                dicu.Time.loc[abs(dicu.Disp_opt_head.loc[:(dicu.Disp_opt_head.idxmax())]-dicu.Disp_opt_head.max()/4).idxmin()]
                )
            xlin_tmp=np.linspace(min(tsm,tsd),maxt_tmp,11)
            
            fig, ax1 = plt.subplots()
            ax1.set_title('%s - Way-measuring time difference'%plt_name)
            ax1.set_xlabel('Time / s')
            ax1.set_ylabel('Way / mm')
            ax1.plot(messu.Time.loc[messu.Time<=maxt_tmp], 
                     messu.Way.loc[messu.Time<=maxt_tmp], 
                     'r-', label='PM-way')
            ax1.plot(dicu.Time.loc[dicu.Time<=maxt_tmp],
                     dicu.Disp_opt_head.loc[dicu.Time<=maxt_tmp],
                     'b-', label='DIC-way')
            ax1.plot(xlin_tmp,np.polyval(linvm,xlin_tmp),
                     'y:',label='PM-lin')
            ax1.plot(xlin_tmp,np.polyval(linvd,xlin_tmp),
                     'g:',label='DIC-lin')
            ax1.axvline(x=tsm, color='red', 
                        linestyle=':', label='PM-way-start')
            ax1.axvline(x=tsd, color='blue', 
                        linestyle=':', label='DIC-way-start')
            ax1.legend()
            ftxt=('$t_{S,PM}$  = % 2.4f s '%(tsm),
                  '$t_{S,DIC}$ = % 2.4f s '%(tsd))
            fig.text(0.95,0.15,'\n'.join(ftxt),
                     ha='right',va='bottom', 
                     bbox=dict(boxstyle='round', edgecolor='0.8', 
                               facecolor='white', alpha=0.8))
            plt_hsuf(fig,path=out_full+"-toff",**plt_scopt)
            del xlin_tmp
        
        log_custom("\n "+"-"*100,**log_scopt)
        log_custom("\n   Time offset between PM and DIC: %.3f s" %(toff),
                   **log_scoptf)
    else:
        toff=0.0
        
    messu.Time=(messu.Time-toff).round(rel_time_digs)
    
    # =============================================================================
    #%%% 4.2 Downsampling of conventional data
    timings.loc[4.2]=time.perf_counter()
    log_custom("\n   Timing %f: %.5f s"%(timings.index[-1],
                                       timings.iloc[-1]-timings.iloc[0]),
                   **log_scopt)
    
    messu, _, mess_f = emec.mc_man.mc_resampler(
        mdf=messu, 
        t_col=_opts['OPT_Measurement_file']['used_names_dict']['Time'], 
        resample=_opts['OPT_Resampling'], 
        res_frequ=_opts['OPT_Resampling_Frequency'], 
        move_ave=_opts['OPT_Resampling_moveave'], 
        ma_sampler='data_rf',
        rel_time_digs=rel_time_digs) 
    
    # =============================================================================
    #%%% 4.3 Merge optical to conventional data
    timings.loc[4.3]=time.perf_counter()
    log_custom("\n   Timing %f: %.5f s"%(timings.index[-1],
                                       timings.iloc[-1]-timings.iloc[0]),
                   **log_scopt)
    if _opts['OPT_DIC']:
        dicu.Time=dicu.Time.round(rel_time_digs)
        ind=pd.RangeIndex(
            dicu.loc[dicu.Time>=messu.Time.min()].index[0],
            messu.Time.count()+dicu.loc[dicu.Time>=messu.Time.min()].index[0],1
            )
        messu=messu.merge(dicu,how='left', on='Time').set_index(ind)
    else:
        dic_f=round(1/dic_dt)
    f_vdm=dic_f/mess_f
        
    # =============================================================================
    #%% 5 Start and End
    log_custom("\n "+"="*100,**log_scopt)
    log_custom("\n ### 5 Start and End ###",**log_scoptf)
    timings.loc[5.0]=time.perf_counter()
    log_custom("\n   Timing %f: %.5f s"%(timings.index[-1],
                                       timings.iloc[-1]-timings.iloc[0]),
                   **log_scopt)                                                    
    # =============================================================================
    #%%% 5.1 Determine start and end of evaluation
    timings.loc[5.1]=time.perf_counter()
    log_custom("\n   Timing %f: %.5f s"%(timings.index[-1],
                                       timings.iloc[-1]-timings.iloc[0]),
                   **log_scopt)
    if np.isnan(_opts['OPT_End']):
        dic_to_mess_End=messu.iloc[-1].name
    else:
        # dic_to_mess_End=messu.loc[messu.Time<=prot.loc[prot_lnr]['DIC_End']*1/f_vdm].index[-1]
        dic_to_mess_End=messu.loc[messu.Time<=_opts['OPT_End']].index[-1]
        
    mun_tmp=messu.loc[:min(dic_to_mess_End,messu.Force.idxmax()),'Force']
    messu['driF'],messu['dcuF'],messu['driF_schg']=emec.mc_char.rise_curve(
        messu.loc[:dic_to_mess_End]['Force'],
        _opts['OPT_Rise_Smoothing'][0], _opts['OPT_Rise_Smoothing'][1]
        )
    
    for i in messu.index: # Startpunkt über Vorzeichenwechsel im Anstieg
        if messu.loc[i,'driF_schg']:
            if not messu.loc[i+1:i+max(int(_opts['OPT_Determination_Distance']/2),1),'driF_schg'].any():
                messu_iS=i
                break
    
    messu_iS,_=emec.mc_char.find_SandE(
        messu.loc[messu_iS:+messu_iS+_opts['OPT_Determination_Distance'],'driF'],
        abs(messu['driF']).quantile(0.5),
        "pgm_other",
        0.1
        )
    # _,messu_iE=emec.mc_char.find_SandE(messu['driF'],0,"qua_self",0.5) # not changed 251022 (B5-sr09)
    try: # search after maximum Force
        _,messu_iE=emec.mc_char.find_SandE(
            messu['driF'].loc[messu.Force.idxmax():],
            messu['driF'],
            "qua_other",
            0.5
            )
    except IndexError:
        messu_iE=dic_to_mess_End
    messu_iE=min(messu_iE,dic_to_mess_End)
    
    log_custom("\n "+"-"*100,**log_scopt)
    log_custom("\n   Start of evaluation after %.3f seconds, corresponds to %.5f %% of max. force."
               %(messu.Time[messu_iS],100*abs(messu.Force[messu_iS])/abs(messu.Force).max()),
               **log_scoptf)
    
    messu=messu.loc[messu_iS:messu_iE]
    if _opts['OPT_DIC']:
        P_xcoord_ydisp_meas=P_xcoord_ydisp_meas.loc[messu_iS:messu_iE]
        P_xcoord_ydisp_spec=P_xcoord_ydisp_spec.loc[messu_iS:messu_iE]
        step_range_dic = emec.pd_ext.pd_combine_index(step_range_dic,messu.index)
        # step_range_dic = emec.pd_ext.pd_combine_index(step_range_dic[1:],messu.index)
        
    fig, ax1 = plt.subplots()
    ax1.set_title('%s - Measuring'%plt_name)
    color1 = 'tab:red'
    ax1.set_xlabel('Time / s')
    ax1.set_ylabel('Force / N', color=color1)  # we already handled the x-label with ax1
    ax1.tick_params(axis='y', labelcolor=color1)
    ax1.axvline(x=messu.Time.loc[messu_iS], color='gray', linestyle='-')
    ax1.axvline(x=messu.Time.loc[messu_iE], color='gray', linestyle='-')
    if np.invert(np.isnan(_opts['OPT_End'])):
        ax1.axvline(x=_opts['OPT_End'], color='gray', linestyle='-.')
    ax1.plot(mess.Time, -mess.F_WZ, 'r-', label='Force-WZ')
    ax1.plot(mess.Time, -mess.F_PM, 'm-', label='Force-PM')
    if _opts['OPT_Springreduction']: 
        ax1.plot(mess.Time, mess.F_IWA_red, 'b:', label='Force-red. (LVDT)')
    ax2 = ax1.twinx()  # instantiate a second axes that shares the same x-axis
    ax2.grid(False)
    color2 = 'tab:blue'
    ax2.set_ylabel('Way / mm', color=color2)  # we already handled the x-label with ax1
    ax2.tick_params(axis='y', labelcolor=color2)
    ax2.plot(mess.Time, -(mess.L_PM), 'y--', label='Way-PM')
    ax2.plot(mess.Time, mess.L_IWA1-mess.L_IWA1[0], 'b--', label='Way-IWA1')
    ax2.plot(mess.Time, mess.L_IWA2-mess.L_IWA2[0], 'b-.', label='Way-IWA2')
    if _opts['OPT_DIC']:
        ax2.plot(dic.Time, dic.Disp_opt_head, 'k:', label='Way-DIC')
        # ax2.plot(dic.Time, dic.DDisp_PM_c, 'm:', label='Way-DIC-P')
        # ax2.plot(dic.Time, dic.DDisp_PC_c, 'g:', label='Way-DIC-C')
    fig.legend(loc='upper left', bbox_to_anchor=(0.1, 0.9), ncol=1)
    plt_hsuf(fig,path=out_full+"-meas",**plt_scopt)
    
    # =============================================================================
    #%%% 5.2 Resetting way
    timings.loc[5.2]=time.perf_counter()
    log_custom("\n   Timing %f: %.5f s"%(timings.index[-1],
                                       timings.iloc[-1]-timings.iloc[0]),
                   **log_scopt)
    # messu.Force=messu.Force-messu.Force.loc[messu_iS]
    messu.Way=messu.Way-messu.Way.loc[messu_iS]
    
    if _opts['OPT_DIC']:    
        messu.Disp_opt_head=messu.Disp_opt_head-messu.Disp_opt_head.loc[messu_iS]
        P_xcoord_ydisp_meas.loc(axis=1)[:,'y'] = P_xcoord_ydisp_meas.loc(axis=1)[:,'y']-P_xcoord_ydisp_meas.loc(axis=1)[:,'y'].loc[messu_iS]
        P_xcoord_ydisp_spec.loc(axis=1)[:,'y'] = P_xcoord_ydisp_spec.loc(axis=1)[:,'y']-P_xcoord_ydisp_spec.loc(axis=1)[:,'y'].loc[messu_iS]

    fig, ax1 = plt.subplots()
    ax1.set_title('%s - Measuring (used)'%plt_name)
    color1 = 'tab:red'
    ax1.set_xlabel('Time / s')
    ax1.set_ylabel('Force / N', color=color1)  # we already handled the x-label with ax1
    ax1.tick_params(axis='y', labelcolor=color1)
    ax1.axvline(x=messu.Time.loc[messu_iS], color='gray', linestyle='-')
    ax1.axvline(x=messu.Time.loc[messu_iE], color='gray', linestyle='-')
    ax1.plot(messu.Time, messu.Force, 'r-', label='Force')
    ax2 = ax1.twinx()
    ax2.grid(False)
    color2 = 'tab:blue'
    ax2.set_ylabel('Way / mm', color=color2)
    ax2.tick_params(axis='y', labelcolor=color2)
    ax2.plot(messu.Time, messu.Way, 'b--', label='Way')
    if _opts['OPT_DIC']:
        ax2.plot(messu.Time, messu.Disp_opt_head, 'k:', label='Way-DIC')
        # ax2.plot(messu.Time, messu.DDisp_PM_c, 'm:', label='Way-DIC-P')
        # ax2.plot(messu.Time, messu.DDisp_PC_c, 'g:', label='Way-DIC-C')
    fig.legend(loc='upper left', bbox_to_anchor=(0.1, 0.9), ncol=1)
    plt_hsuf(fig,path=out_full+"-meas_u",**plt_scopt)
    
    # =============================================================================
    #%% 6 Evaluation
    log_custom("\n "+"="*100,**log_scopt)
    log_custom("\n ### 6 Evaluation ###",**log_scoptf)
    timings.loc[6.0]=time.perf_counter()
    log_custom("\n   Timing %f: %.5f s"%(timings.index[-1],
                                       timings.iloc[-1]-timings.iloc[0]),
                   **log_scopt)
    # =============================================================================
    #%%% 6.0 Perform DIC-fittings
    #%%%% 6.11 Pre-Fit with all components (Indentaion, shear force deformation and bending deformation)
    log_custom("\n "+"-"*100,**log_scopt)
    log_custom('\n## Pre-fit on displacements:',**log_scopt)
    timings.loc[6.11]=time.perf_counter()
    log_custom("\n   Timing %f: %.5f s"%(timings.index[-1],
                                       timings.iloc[-1]-timings.iloc[0]),
                   **log_scopt)
    
    Pre_fit_df = emeb.fitting.Perform_Fit(
        BFL=bl, Fit_func_key='w_A', 
        P_df=P_xcoord_ydisp_meas,
        lB=Bo_Le, rB=Bo_Ri, 
        # s_range=step_range_dic, #Verschiebung an 0 is 0
        s_range=step_range_dic[1:],
        Shear_func_key='w_S', gamma_V=gamma_V, 
        err_weights=_opts['OPT_DIC_Fitting']['Bending_MCFit_opts']['error_weights_pre'], 
        max_nfev=_opts['OPT_DIC_Fitting']['Bending_MCFit_opts']['fit_max_nfev_pre'],
        nan_policy='omit',
        option='Pre', pb_b=True,**pwargs
        )
    for step in Pre_fit_df.index:
        ftxt=lmfit.fit_report(Pre_fit_df['Fit_Result'].loc[step])
        ftxt=ftxt.replace("\n[[Variables]]","\n    R-square-multi     = %1.5f\n[[Variables]]"%Pre_fit_df['Rquad_multi'].loc[step])
        ftxt=ftxt.replace("\n[[Variables]]","\n    R-square-disp      = %1.5f\n[[Variables]]"%Pre_fit_df['Rquad_disp'].loc[step])
        log_custom('\n  Step %d'%step+log_cind(ftxt),
                       **log_scopt)

    
    # =============================================================================
    #%%%% 6.12 Refit to adjusted bending deformation
    log_custom("\n "+"-"*100,**log_scopt)
    log_custom('\n## Bend-fit on displacements:',**log_scopt)
    timings.loc[6.12]=time.perf_counter()
    log_custom("\n   Timing %f: %.5f s"%(timings.index[-1],
                                       timings.iloc[-1]-timings.iloc[0]),
                   **log_scopt)
    
    P_xcoord_ydisp_meas_M=P_xcoord_ydisp_meas.loc[step_range_dic].copy(deep=True)
    P_xcoord_ydisp_meas_S=P_xcoord_ydisp_meas.loc[step_range_dic].copy(deep=True)
    iy=bl['w_I']['d0'](emeb.opt_mps.Point_df_idx(P_xcoord_ydisp_meas_M,coords='x'),
                       Pre_fit_df.loc[:,'Fit_params_dict'])
    vy=bl['w_V']['d0'](emeb.opt_mps.Point_df_idx(P_xcoord_ydisp_meas_M,coords='x'),
                       Pre_fit_df.loc[:,'Fit_params_dict'])
    P_xcoord_ydisp_meas_S.loc(axis=1)[:,'y']=P_xcoord_ydisp_meas_S.loc(axis=1)[:,'y']-iy
    P_xcoord_ydisp_meas_M.loc(axis=1)[:,'y']=P_xcoord_ydisp_meas_M.loc(axis=1)[:,'y']-iy-vy
    
    Bend_fit_df = emeb.fitting.Perform_Fit(
        BFL=bl, Fit_func_key='w_M',
        P_df=P_xcoord_ydisp_meas_M,
        lB=Bo_Le, rB=Bo_Ri, 
        s_range=step_range_dic[1:], 
        err_weights=_opts['OPT_DIC_Fitting']['Bending_MCFit_opts']['error_weights_bend'], 
        max_nfev=_opts['OPT_DIC_Fitting']['Bending_MCFit_opts']['fit_max_nfev_bend'],
        nan_policy='omit',
        option='Bend', pb_b=True,**pwargs
        )
    for step in Bend_fit_df.index:
        ftxt=lmfit.fit_report(Bend_fit_df['Fit_Result'].loc[step])
        ftxt=ftxt.replace("\n[[Variables]]","\n    R-square-multi     = %1.5f\n[[Variables]]"%Bend_fit_df['Rquad_multi'].loc[step])
        ftxt=ftxt.replace("\n[[Variables]]","\n    R-square-disp      = %1.5f\n[[Variables]]"%Bend_fit_df['Rquad_disp'].loc[step])
        log_custom('\n  Step %d'%step+log_cind(ftxt),
                       **log_scopt)
    
    # =============================================================================
    #%%%% Incremental:
    # P_xcoord_ydiff = emeb.opt_mps.Points_diff(emeb.opt_mps.Point_df_idx(Points_L_T_stacked, coords=['x','y'], deepcopy=True))
    P_xcoord_ydiff = emeb.opt_mps.Points_diff(P_xcoord_ydisp.loc[step_range_dic])

    P_xcoord_ydiff_meas = emeb.opt_mps.Point_df_idx(
        P_xcoord_ydiff, steps=step_range_dic, 
        points='P', deepcopy=False, option='Regex'
        )
    P_xcoord_ydiff_spec = emeb.opt_mps.Point_df_idx(
        P_xcoord_ydiff, steps=step_range_dic, 
        points='S', deepcopy=False, option='Regex'
        )
    step_range_dic_inc = step_range_dic[np.where(np.invert(
        emeb.opt_mps.Point_df_idx(
            P_xcoord_ydiff_meas, steps=step_range_dic, coords='y'
            ).isna()).sum(axis=1) >= (_opts['OPT_DIC_Tester'][3]))]
    # =============================================================================
    #%%%% 6.13 Pre-Fit with all components (Indentaion, shear force deformation and bending deformation)
    log_custom("\n "+"-"*100,**log_scopt)
    log_custom('\n## Pre-fit on increments:',**log_scopt)
    timings.loc[6.13]=time.perf_counter()
    log_custom("\n   Timing %f: %.5f s"%(timings.index[-1],
                                       timings.iloc[-1]-timings.iloc[0]),
                   **log_scopt)
    
    Pre_inc_fit_df = emeb.fitting.Perform_Fit(
        BFL=bl, Fit_func_key='w_A', 
        P_df=P_xcoord_ydiff_meas,
        lB=Bo_Le, rB=Bo_Ri, 
        s_range=step_range_dic_inc[1:],
        Shear_func_key='w_S', gamma_V=gamma_V, 
        err_weights=_opts['OPT_DIC_Fitting']['Bending_MCFit_opts']['error_weights_pre_inc'], 
        max_nfev=_opts['OPT_DIC_Fitting']['Bending_MCFit_opts']['fit_max_nfev_pre_inc'],
        nan_policy='omit',
        option='Pre', pb_b=True,**pwargs
        )
    for step in Pre_inc_fit_df.index:
        ftxt=lmfit.fit_report(Pre_inc_fit_df['Fit_Result'].loc[step])
        ftxt=ftxt.replace("\n[[Variables]]","\n    R-square-multi     = %1.5f\n[[Variables]]"%Pre_inc_fit_df['Rquad_multi'].loc[step])
        ftxt=ftxt.replace("\n[[Variables]]","\n    R-square-disp      = %1.5f\n[[Variables]]"%Pre_inc_fit_df['Rquad_disp'].loc[step])
        log_custom('\n  Step %d'%step+log_cind(ftxt),
                       **log_scopt)
    
    # =============================================================================
    #%%%% 6.14 Refit to adjusted bending deformation
    log_custom("\n "+"-"*100,**log_scopt)
    log_custom('\n## Bend-fit on increments:',**log_scopt)
    timings.loc[6.14]=time.perf_counter()
    log_custom("\n   Timing %f: %.5f s"%(timings.index[-1],
                                       timings.iloc[-1]-timings.iloc[0]),
                   **log_scopt)
    
    P_xcoord_ydiff_meas_M=P_xcoord_ydiff_meas.loc[step_range_dic].copy(deep=True)
    P_xcoord_ydiff_meas_S=P_xcoord_ydiff_meas.loc[step_range_dic].copy(deep=True)
    iy=bl['w_I']['d0'](
        emeb.opt_mps.Point_df_idx(P_xcoord_ydiff_meas_M, coords='x'),
        Pre_inc_fit_df.loc[:,'Fit_params_dict']
        )
    vy=bl['w_V']['d0'](
        emeb.opt_mps.Point_df_idx(P_xcoord_ydiff_meas_M, coords='x'),
        Pre_inc_fit_df.loc[:,'Fit_params_dict']
        )
    P_xcoord_ydiff_meas_S.loc(axis=1)[:,'y']=P_xcoord_ydiff_meas_S.loc(axis=1)[:,'y']-iy
    P_xcoord_ydiff_meas_M.loc(axis=1)[:,'y']=P_xcoord_ydiff_meas_M.loc(axis=1)[:,'y']-iy-vy
    
    Bend_inc_fit_df = emeb.fitting.Perform_Fit(
        BFL=bl, Fit_func_key='w_M',
        P_df=P_xcoord_ydiff_meas_M,
        lB=Bo_Le, rB=Bo_Ri, 
        s_range=step_range_dic_inc[1:], 
        err_weights=_opts['OPT_DIC_Fitting']['Bending_MCFit_opts']['error_weights_bend_inc'], 
        max_nfev=_opts['OPT_DIC_Fitting']['Bending_MCFit_opts']['fit_max_nfev_bend_inc'],
        nan_policy='omit',
        option='Bend', pb_b=True,**pwargs
        )
    for step in Bend_inc_fit_df.index:
        ftxt=lmfit.fit_report(Bend_inc_fit_df['Fit_Result'].loc[step])
        ftxt=ftxt.replace("\n[[Variables]]","\n    R-square-multi     = %1.5f\n[[Variables]]"%Bend_inc_fit_df['Rquad_multi'].loc[step])
        ftxt=ftxt.replace("\n[[Variables]]","\n    R-square-disp      = %1.5f\n[[Variables]]"%Bend_inc_fit_df['Rquad_disp'].loc[step])
        log_custom('\n  Step %d'%step+log_cind(ftxt),
                       **log_scopt)
    
    if True:
        # Fit comparison ----------------------------------------------------------
        # step=messu.Force.idxmax()
        step = emec.pd_ext.pd_combine_index(
            messu.loc[:messu.Force.idxmax()],
            step_range_dic_inc
            )[-1]
        
        fig, ax1 = plt.subplots()
        ax1.set_title('%s - Fit-compare - Displacement for step %i'%(plt_name,step))
        ax1.set_xlabel('x / mm')
        ax1.set_ylabel('y displacement / mm')
        ax1.plot(xlin, bl['w_A']['d0'](xlin,Pre_fit_df.loc[step,'Fit_params_dict']),
                 'r:', label='Org.')
        ax1.plot(xlin, bl['w_I']['d0'](xlin,Pre_fit_df.loc[step,'Fit_params_dict']),
                 'g:', label='Ind.')
        ax1.plot(xlin, bl['w_S']['d0'](xlin,Pre_fit_df.loc[step,'Fit_params_dict']),
                 'b:', label='Wo. ind.')
        ax1.plot(xlin, bl['w_V']['d0'](xlin,Pre_fit_df.loc[step,'Fit_params_dict']),
                 'm--', label='V')
        ax1.plot(xlin, bl['w_M_ima']['d0'](xlin,Pre_fit_df.loc[step,'Fit_params_dict']),
                 'g--', label='Imag. M')
        ax1.plot(xlin, bl['w_M']['d0'](xlin,Bend_fit_df.loc[step,'Fit_params_dict']),
                 'r--', label='M')
        ax1.plot(P_xcoord_ydisp_meas.loc[step].loc[:,'x'],
                 P_xcoord_ydisp_meas.loc[step].loc[:,'y'], 
                 'ko', label='org. P')
        ax1.plot(P_xcoord_ydisp_meas_M.loc[step].loc[:,'x'],
                 P_xcoord_ydisp_meas_M.loc[step].loc[:,'y'], 
                 'go', label='M P')
        ax1.legend()
        plt_hsuf(fig,path=out_full+"-DIC_fit-bl_U-d0",**plt_scopt)
        
        fig, ax1 = plt.subplots()
        ax1.set_title('%s - Fit-compare - Slope for step %i'%(plt_name,step))
        ax1.set_xlabel('x / mm')
        ax1.set_ylabel('slope / (mm/mm)')
        ax1.plot(xlin, bl['w_A']['d1'](xlin,Pre_fit_df.loc[step,'Fit_params_dict']),
                 'r:', label='Org.')
        ax1.plot(xlin, bl['w_S']['d1'](xlin,Pre_fit_df.loc[step,'Fit_params_dict']),
                 'b:', label='Wo. ind.')
        ax1.plot(xlin, bl['w_V']['d1'](xlin,Pre_fit_df.loc[step,'Fit_params_dict']),
                 'm--', label='V')
        ax1.plot(xlin, bl['w_M']['d1'](xlin,Bend_fit_df.loc[step,'Fit_params_dict']),
                 'r--', label='M')
        ax1.legend()
        plt_hsuf(fig,path=out_full+"-DIC_fit-bl_U-d1",**plt_scopt)
        
        fig, ax1 = plt.subplots()
        ax1.set_title('%s - Fit-compare - Curvature for step %i'%(plt_name,step))
        ax1.set_xlabel('x / mm')
        ax1.set_ylabel('curvature / (1/mm)')
        ax1.plot(xlin, bl['w_A']['d2'](xlin,Pre_fit_df.loc[step,'Fit_params_dict']),
                 'r:', label='Org.')
        ax1.plot(xlin, bl['w_S']['d2'](xlin,Pre_fit_df.loc[step,'Fit_params_dict']),
                 'b:', label='Wo. ind.')
        ax1.plot(xlin, bl['w_M']['d2'](xlin,Bend_fit_df.loc[step,'Fit_params_dict']),
                 'r--', label='M')
        ax1.legend()
        plt_hsuf(fig,path=out_full+"-DIC_fit-bl_U-d2",**plt_scopt)
        
        fig, ax1 = plt.subplots()
        ax1.set_title('%s - Fit-compare-inc - Displacement for step %i'%(plt_name,step))
        ax1.set_xlabel('x / mm')
        ax1.set_ylabel('y displacement / mm')
        ax1.plot(xlin, bl['w_A']['d0'](xlin,Pre_inc_fit_df.loc[step,'Fit_params_dict']), 
                 'r:', label='Org.')
        ax1.plot(xlin, bl['w_I']['d0'](xlin,Pre_inc_fit_df.loc[step,'Fit_params_dict']),
                 'g:', label='Ind.')
        ax1.plot(xlin, bl['w_S']['d0'](xlin,Pre_inc_fit_df.loc[step,'Fit_params_dict']),
                 'b:', label='Wo. ind.')
        ax1.plot(xlin, bl['w_V']['d0'](xlin,Pre_inc_fit_df.loc[step,'Fit_params_dict']),
                 'm--', label='V')
        ax1.plot(xlin, bl['w_M_ima']['d0'](xlin,Pre_inc_fit_df.loc[step,'Fit_params_dict']),
                 'g--', label='Imag. M')
        ax1.plot(xlin, bl['w_M']['d0'](xlin,Bend_inc_fit_df.loc[step,'Fit_params_dict']),
                 'r--', label='M')
        ax1.plot(P_xcoord_ydiff_meas.loc[step].loc[:,'x'],
                 P_xcoord_ydiff_meas.loc[step].loc[:,'y'], 
                 'ko', label='org. P')
        ax1.plot(P_xcoord_ydiff_meas_M.loc[step].loc[:,'x'],
                 P_xcoord_ydiff_meas_M.loc[step].loc[:,'y'], 
                 'go', label='M P')
        ax1.legend()
        plt_hsuf(fig,path=out_full+"-INC_fit-bl_U-d0",**plt_scopt)
        
        # Pre-Fit -----------------------------------------------------------------
        emeb.plotting.colplt_funcs_all(
            x=xlin, func_cohort=bl['w_A'],
            params=Pre_fit_df.loc(axis=1)['Fit_params_dict'],
            step_range=step_range_dic[1:], 
            title=('%s - Fit-full'%plt_name),
            xlabel='x  / mm',
            Point_df=P_xcoord_ydisp_meas,
            path=out_full+'-DIC_fit-A',
            plt_scopt=plt_scopt
            )
        # Bending-Fit -------------------------------------------------------------
        emeb.plotting.colplt_funcs_all(
            x=xlin, func_cohort=bl['w_M'],
            params=Bend_fit_df.loc(axis=1)['Fit_params_dict'],
            step_range=step_range_dic[1:], 
            title=('%s - Fit-Bending'%plt_name),
            xlabel='x / mm',
            Point_df=P_xcoord_ydisp_meas_M,
            path=out_full+'-DIC_fit-M',
            plt_scopt=plt_scopt
            )
        # Pre-Fit -----------------------------------------------------------------
        emeb.plotting.colplt_funcs_all(
            x=xlin, func_cohort=bl['w_A'],
            params=Pre_inc_fit_df.loc(axis=1)['Fit_params_dict'],
            step_range=step_range_dic_inc[1:], 
            title=('%s - Incremental Fit-full'%plt_name),
            xlabel='x / mm',
            Point_df=P_xcoord_ydiff_meas,
            path=out_full+'-INC_fit-A',
            plt_scopt=plt_scopt
            )
        # Bending-Fit -------------------------------------------------------------
        emeb.plotting.colplt_funcs_all(
            x=xlin, func_cohort=bl['w_M'],
            params=Bend_inc_fit_df.loc(axis=1)['Fit_params_dict'],
            step_range=step_range_dic_inc[1:], 
            title=('%s - Incremental Fit-Bending'%plt_name),
            xlabel='x / mm',
            Point_df=P_xcoord_ydiff_meas_M,
            path=out_full+'-INC_fit-M', 
            plt_scopt=plt_scopt
            )
    
    #%%% 6.2 Determine evaluation curves
    log_custom("\n "+"-"*100,**log_scopt)
    log_custom("\n ### -6.2 Determine evaluation curves ###",**log_scopt)
    timings.loc[6.2]=time.perf_counter()
    log_custom("\n   Timing %f: %.5f s"%(timings.index[-1],
                                       timings.iloc[-1]-timings.iloc[0]),
                   **log_scopt)
    
    messu['Moment']=emeb.evaluation.Moment_perF_func(0.0,Bo_Le,Bo_Ri)*messu.Force
    messu['Stress']=emeb.evaluation.stress_perF(0.0,func_I,func_t,Bo_Le,Bo_Ri)*messu.Force
    
    messu['Strain']=6*prot_ser['thickness_2']*messu.Way/Length**2

    if _opts['OPT_DIC']:
        messu['Disp_opt_A']=bl['w_A']['d0'](0.0,Pre_fit_df['Fit_params_dict'])
        if _opts['OPT_Compression']: messu['Disp_opt_A']=messu['Disp_opt_A']*(-1)
        messu['Strain_opt_d_A']=6*prot_ser['thickness_2']*messu['Disp_opt_A']/Length**2
        messu['Disp_opt_S']=bl['w_S']['d0'](0.0,Pre_fit_df['Fit_params_dict'])
        if _opts['OPT_Compression']: messu['Disp_opt_S']=messu['Disp_opt_S']*(-1)
        messu['Strain_opt_d_S']=6*prot_ser['thickness_2']*messu['Disp_opt_S']/Length**2
        messu['Disp_opt_M']=bl['w_M']['d0'](0.0,Bend_fit_df['Fit_params_dict'])
        if _opts['OPT_Compression']: messu['Disp_opt_M']=messu['Disp_opt_M']*(-1)
        messu['Strain_opt_d_M']=6*prot_ser['thickness_2']*messu['Disp_opt_M']/Length**2
    
        messu['Strain_opt_c_A']=emeb.evaluation.straindf_from_curve(
            0.0, bl['w_A']['d2'], Pre_fit_df['Fit_params_dict'], func_t
            )
        messu['Strain_opt_c_S']=emeb.evaluation.straindf_from_curve(
            0.0, bl['w_S']['d2'], Pre_fit_df['Fit_params_dict'], func_t
            )
        messu['Strain_opt_c_M']=emeb.evaluation.straindf_from_curve(
            0.0, bl['w_M']['d2'], Bend_fit_df['Fit_params_dict'], func_t
            )
        # Set first value of optical displacement and strain to 0.0 instead of NaN
        messu.loc[messu_iS,[    'Disp_opt_A',    'Disp_opt_S',    'Disp_opt_M',]]=0.0
        messu.loc[messu_iS,['Strain_opt_d_A','Strain_opt_d_S','Strain_opt_d_M',]]=0.0
        messu.loc[messu_iS,['Strain_opt_c_A','Strain_opt_c_S','Strain_opt_c_M',]]=0.0

    #Test Anstieg
        tmon=emec.mc_char.test_pdmon(
            messu,
            ['Stress','Strain',
             'Strain_opt_d_A','Strain_opt_d_S','Strain_opt_d_M',
             'Strain_opt_c_A','Strain_opt_c_S','Strain_opt_c_M'],
            1,10
            )
    else:
        tmon=emec.mc_char.test_pdmon(messu,['Stress','Strain'],1,10)
    
    log_custom("\n   Last 10 monoton increasing periods:\n    %s"
               %tmon.to_frame(name='Epoche').T.to_string().replace('\n','\n    '),
               **log_scoptf)
    
    # =============================================================================
    #%%% 6.3 Determine points of interest
    log_custom("\n "+"-"*100,**log_scopt)
    log_custom("\n ### -6.3 Determine points of interest ###",**log_scopt)
    timings.loc[6.31]=time.perf_counter()
    log_custom("\n   Timing %f: %.5f s"%(timings.index[-1],
                                       timings.iloc[-1]-timings.iloc[0]),
                   **log_scopt)
    VIP_messu=pd.Series([],dtype='int64',name='VIP_messu')
    VIP_messu['S']=messu.driF.index[0]
    VIP_messu['E']=messu.driF.index[-1]
    VIP_messu['U']=messu.Force.idxmax()
    
    mun_tmp = messu.loc[VIP_messu['S']+_opts['OPT_Determination_Distance']:VIP_messu['U']-1]
    if mun_tmp.driF_schg.any()==True: # 
        VIP_messu['Y']=mun_tmp.loc[mun_tmp.driF_schg==True].index[0]-1
    else:
        VIP_messu['Y']=VIP_messu['U']
        log_custom('\n    Fy set on datapoint of Fu!', **log_scoptf)
        
    # mun_tmp = messu.loc[VIP_messu['U']:VIP_messu['E']-1]
    mun_tmp = messu.loc[VIP_messu['U']-1:VIP_messu['E']-1]
    if mun_tmp.driF_schg.any():
        i=mun_tmp.loc[mun_tmp.driF_schg].index[0]
        # statt allgemeinem Minimum bei größtem Kraftabfall nahe Maximalkraft,
        #  -2 da differenz aussage über vorherigen punkt
        VIP_messu['B']  =mun_tmp.driF.loc[i:i+_opts['OPT_Determination_Distance']].idxmin()-2 
        if VIP_messu['B']<VIP_messu['U']: VIP_messu['B']=VIP_messu['U']
    else:
        log_custom('\n   Fb not reliably determinable!', **log_scoptf)
            
    ftmp=float(messu.Force.loc[VIP_messu[_opts['OPT_YM_Determination_range'][2]]]*_opts['OPT_YM_Determination_range'][0])
    VIP_messu['F1']=abs(messu.Force.loc[:VIP_messu[_opts['OPT_YM_Determination_range'][2]]]-ftmp).idxmin()
    ftmp=float(messu.Force.loc[VIP_messu[_opts['OPT_YM_Determination_range'][2]]]*_opts['OPT_YM_Determination_range'][1])
    VIP_messu['F2']=abs(messu.Force.loc[:VIP_messu[_opts['OPT_YM_Determination_range'][2]]]-ftmp).idxmin()
    
     # Test ob Streckgrenze zwischen F1 und F2 liegt
    if (VIP_messu['Y']>VIP_messu['F1']) and (VIP_messu['Y']<VIP_messu['F2']):
        VIP_messu['F2']=VIP_messu['Y']
        log_custom("\n   F2 set on Y (Force-rise between F1 and old F2)",
                   **log_scoptf)
    
    VIP_messu=VIP_messu.sort_values()
    if _opts['OPT_DIC']:
        VIP_dicu=VIP_messu.copy(deep=True)
        VIP_dicu.name='VIP_dicu'
    
    #%%%% 6.3.2 Improvement of evaluation range
    timings.loc[6.32]=time.perf_counter()
    log_custom("\n   Timing %f: %.5f s"%(timings.index[-1],
                                       timings.iloc[-1]-timings.iloc[0]),
                   **log_scopt)
    # (siehe Keuerleber, M. (2006). Bestimmung des Elastizitätsmoduls von Kunststoffen bei hohen Dehnraten am Beispiel von PP. Von der Fakultät Maschinenbau der Universität Stuttgart zur Erlangung der Würde eines Doktor-Ingenieurs (Dr.-Ing.) genehmigte Abhandlung. Doktorarbeit. Universität Stuttgart, Stuttgart.)
    ftmp=float(messu.Stress.loc[VIP_messu[_opts['OPT_YM_Determination_range'][2]]]*_opts['OPT_YM_Determination_refinement'][0])
    Lbord=abs(messu.Stress.loc[:VIP_messu[_opts['OPT_YM_Determination_range'][2]]]-ftmp).idxmin()
    
    DQcon=pd.concat(emec.mc_char.Diff_Quot(
        messu.loc[:,'Strain'],
        messu.loc[:,'Stress'],
        _opts['OPT_YM_Determination_refinement'][4],
        _opts['OPT_YM_Determination_refinement'][5]
        ), axis=1)
    DQcon=messu.loc(axis=1)[['Strain','Stress']].join(DQcon,how='outer')
    DQcons=DQcon.loc[Lbord:VIP_messu[_opts['OPT_YM_Determination_refinement'][2]]]
    VIP_messu['FM']=DQcons.DQ1.idxmax()
    try:
        VIP_messu['F3']=DQcons.loc[:VIP_messu['FM']].iloc[::-1].loc[(DQcons.DQ1/DQcons.DQ1.max())<_opts['OPT_YM_Determination_refinement'][1]].index[0]+1
        # VIP_messu['F3']=DQcons.loc[:VIP_messu['FM']].iloc[::-1].loc[(DQcons.DQ1/DQcons.DQ1.max())<_opts['OPT_YM_Determination_refinement'][1]].index[0]
    except IndexError:
        VIP_messu['F3']=DQcons.index[0]
    try: # Hinzugefügt am 16.09.2021
        VIP_messu['F4']=DQcons.loc[VIP_messu['FM']:].loc[(DQcons.DQ1/DQcons.DQ1.max())<_opts['OPT_YM_Determination_refinement'][1]].index[0]-1
        # VIP_messu['F4']=DQcons.loc[VIP_messu['FM']:].loc[(DQcons.DQ1/DQcons.DQ1.max())<_opts['OPT_YM_Determination_refinement'][1]].index[0]
    except IndexError:
        VIP_messu['F4']=VIP_messu['FM']-1 #-1 könnte zu Problemen führen
    VIP_messu=VIP_messu.sort_values()
    
    # optisch:    
    DQopt=pd.concat(emec.mc_char.Diff_Quot(
        messu.loc[:,dic_used_Strain],
        messu.loc[:,'Stress'],
        _opts['OPT_YM_Determination_refinement'][4],
        _opts['OPT_YM_Determination_refinement'][5]
        ), axis=1)
    DQopt=messu.loc(axis=1)[[dic_used_Strain,'Stress']].join(DQopt,how='outer')
    DQopts=DQopt.loc[Lbord:VIP_dicu[_opts['OPT_YM_Determination_refinement'][2]]]
    VIP_dicu['FM']=DQopts.DQ1.idxmax()
    try:
        VIP_dicu['F3']=DQopts.loc[:VIP_dicu['FM']].iloc[::-1].loc[(DQopts.DQ1/DQopts.DQ1.max())<_opts['OPT_YM_Determination_refinement'][1]].index[0]+1
        # VIP_dicu['F3']=DQopts.loc[:VIP_dicu['FM']].iloc[::-1].loc[(DQopts.DQ1/DQopts.DQ1.max())<_opts['OPT_YM_Determination_refinement'][1]].index[0]
    except IndexError:
        VIP_dicu['F3']=DQopts.index[0]
    try: # Hinzugefügt am 16.09.2021
        VIP_dicu['F4']=DQopts.loc[VIP_dicu['FM']:].loc[(DQopts.DQ1/DQopts.DQ1.max())<_opts['OPT_YM_Determination_refinement'][1]].index[0]-1
        # VIP_dicu['F4']=DQopts.loc[VIP_dicu['FM']:].loc[(DQopts.DQ1/DQopts.DQ1.max())<_opts['OPT_YM_Determination_refinement'][1]].index[0]
    except IndexError:
        VIP_dicu['F4']=VIP_dicu['FM']-1 #-1 könnte zu Problemen führen
    VIP_dicu=VIP_dicu.sort_values()
    
    if True:
        fig, (ax1,ax3) = plt.subplots(nrows=2, ncols=1, sharex=False, sharey=False, 
                                      figsize = np.multiply(figsize,[1,2]))
        fig.suptitle('%s - Improvement of evaluation range for Youngs Modulus'%plt_name)
        ax1.set_title('Conventional measured strain')
        ax1.set_xlabel('Strain / -')
        ax1.set_ylabel('Stress / MPa')
        ax1.plot(messu.loc[:VIP_messu[_opts['OPT_YM_Determination_refinement'][2]],'Strain'], messu.loc[:VIP_messu[_opts['OPT_YM_Determination_refinement'][2]],'Stress'], 'r.',label='$\sigma$-$\epsilon$')
        a, b=messu.loc[VIP_messu[:_opts['OPT_YM_Determination_refinement'][2]],'Strain'],messu.loc[VIP_messu[:_opts['OPT_YM_Determination_refinement'][2]],'Stress']
        j=np.int64(-1)
        ax1.plot(a, b, 'bx')
        for x in VIP_messu[:_opts['OPT_YM_Determination_refinement'][2]].index:
            j+=1
            if j%2: c=(6,-6)
            else:   c=(-6,6)
            ax1.annotate('%s' % x, xy=(a.iloc[j],b.iloc[j]), xycoords='data',
                         xytext=c, ha="center", va="center", textcoords='offset points')
        ax2=ax1.twinx()
        ax2.set_ylabel('Normalized derivatives / -')
        # ax2.plot(DQcons['Strain'], 
        #          DQcons['DQ1']/abs(DQcons['DQ1']).max(), 'b:',label='DQ1')
        ax2.plot(DQcons['Strain'], 
                 DQcons['DQ1']/DQcons['DQ1'].max(), 'b:',label='DQ1')
        ax2.plot(DQcons['Strain'], 
                 DQcons['DQ2']/abs(DQcons['DQ2']).max(), 'g:',label='DQ2')
        ax2.plot(DQcons['Strain'], 
                 DQcons['DQ3']/abs(DQcons['DQ3']).max(), 'y:',label='DQ3')
        ax2.axvline(x=messu.loc[VIP_messu['FM'],'Strain'],color='gray', linestyle='-')
        ax2.axvline(x=messu.loc[VIP_messu['F3'],'Strain'],color='gray', linestyle=':')
        ax2.axvline(x=messu.loc[VIP_messu['F4'],'Strain'],color='gray', linestyle=':')
        ax2.axhline(y=_opts['OPT_YM_Determination_refinement'][1],color='gray', linestyle='--')
        ax2.set_yticks([-1,0,1])
        ax2.grid(which='major',axis='y',linestyle=':')
        fig.legend(loc='lower right', ncol=4)
        
        ax3.set_title('Optical measured strain')
        ax3.set_xlabel('Strain / -')
        ax3.set_ylabel('Stress / MPa')
        ax3.plot(messu.loc[:VIP_dicu[_opts['OPT_YM_Determination_refinement'][2]],
                           dic_used_Strain],
                 messu.loc[:VIP_dicu[_opts['OPT_YM_Determination_refinement'][2]],
                           'Stress'], 'r.',label='$\sigma$-$\epsilon$')
        a, b=messu.loc[VIP_dicu[:_opts['OPT_YM_Determination_refinement'][2]],
                       dic_used_Strain], messu.loc[VIP_dicu[:_opts['OPT_YM_Determination_refinement'][2]],'Stress']
        j=np.int64(-1)
        ax3.plot(a, b, 'bx')
        for x in VIP_dicu[:_opts['OPT_YM_Determination_refinement'][2]].index:
            j+=1
            if j%2: c=(6,-6)
            else:   c=(-6,6)
            ax3.annotate('%s' % x, xy=(a.iloc[j],b.iloc[j]), xycoords='data',
                         xytext=c, ha="center", va="center", textcoords='offset points')
        ax4=ax3.twinx()
        ax4.set_ylabel('Normalized derivatives / -')
        # ax4.plot(DQopts[dic_used_Strain],
        #          DQopts['DQ1']/abs(DQopts['DQ1']).max(), 'b:',label='DQ1')
        ax4.plot(DQopts[dic_used_Strain],
                 DQopts['DQ1']/DQopts['DQ1'].max(), 'b:',label='DQ1')
        ax4.plot(DQopts[dic_used_Strain],
                 DQopts['DQ2']/abs(DQopts['DQ2']).max(), 'g:',label='DQ2')
        ax4.plot(DQopts[dic_used_Strain],
                 DQopts['DQ3']/abs(DQopts['DQ3']).max(), 'y:',label='DQ3')
        # ax4.axvline(x=DQopts.loc[VIP_dicu['FM'],dic_used_Strain],color='gray', linestyle='-')
        # ax4.axvline(x=DQopts.loc[VIP_dicu['F3'],dic_used_Strain],color='gray', linestyle=':')
        # ax4.axvline(x=DQopts.loc[VIP_dicu['F4'],dic_used_Strain],color='gray', linestyle=':')
        ax4.axvline(x=messu.loc[VIP_dicu['FM'],dic_used_Strain],
                    color='gray', linestyle='-')
        ax4.axvline(x=messu.loc[VIP_dicu['F3'],dic_used_Strain],
                    color='gray', linestyle=':')
        ax4.axvline(x=messu.loc[VIP_dicu['F4'],dic_used_Strain],
                    color='gray', linestyle=':')
        ax4.axhline(y=_opts['OPT_YM_Determination_refinement'][1],
                    color='gray', linestyle='--')
        ax4.set_yticks([-1,0,1])
        ax4.grid(which='major',axis='y',linestyle=':')
        plt_hsuf(fig,path=out_full+"-YMRange_Imp",**plt_scopt)
     
    if _opts['OPT_DIC']:
        tmp={'con F1-F2':VIP_messu['F2']-VIP_messu['F1'],
             'opt F1-F2':VIP_dicu['F2']-VIP_dicu['F1'],
             'con F3-F4':VIP_messu['F4']-VIP_messu['F3'],
             'opt F3-F4':VIP_dicu['F4']-VIP_dicu['F3']}
        log_custom("\n   Datapoints (con/opt) between F1-F2: %d/%d and F3-F4: %d/%d."
                       %(*tmp.values(),), **log_scoptf)
        for i in tmp.keys(): 
            if tmp[i] < 3: cout+='%s:%d DPs, '%(i,tmp[i])
    else:
        tmp={'con F1-F2':VIP_messu['F2']-VIP_messu['F1'],
             'con F3-F4':VIP_messu['F4']-VIP_messu['F3']}
        log_custom("\n   Datapoints (con) between F1-F2: %d and F3-F4: %d."
                       %(*tmp.values(),), **log_scoptf)
        for i in tmp.keys(): 
            if tmp[i] < 3: cout+='%s:%d DPs, '%(i,tmp[i])
            
    # =====================================================================================
    #%%% 6.4 Determine Youngs-Moduli
    log_custom("\n "+"-"*100,**log_scopt)
    log_custom("\n ### -6.4 Determine Youngs-Moduli ###",**log_scopt)
    timings.loc[6.4]=time.perf_counter()
    log_custom("\n   Timing %f: %.5f s"%(timings.index[-1],
                                       timings.iloc[-1]-timings.iloc[0]),
                   **log_scopt)
    
    d_stress_mid = messu.Stress.diff()
    d_strain_mid = messu.Strain.diff()
    d_Strain_opt_d_A_mid = messu.Strain_opt_d_A.diff()
    d_Strain_opt_d_S_mid = messu.Strain_opt_d_S.diff()
    d_Strain_opt_d_M_mid = messu.Strain_opt_d_M.diff()
    d_Strain_opt_c_A_mid = messu.Strain_opt_c_A.diff()
    d_Strain_opt_c_S_mid = messu.Strain_opt_c_S.diff()
    d_Strain_opt_c_M_mid = messu.Strain_opt_c_M.diff()
    d_Force = messu.Force.diff()
    
    # sr_eva_con = messu.loc[VIP_messu['F3']:VIP_messu['F4']].index
    # sr_eva_dic = Evac.pd_combine_index(step_range_dic, 
    #                                    messu.loc[VIP_dicu['F3']:VIP_dicu['F4']])
    Ind_YM_f=['F1','F2']
    Ind_YM_r=['F3','F4']
    sf_eva_con = messu.loc[VIP_messu[Ind_YM_f[0]]:VIP_messu[Ind_YM_f[1]]].index
    sf_eva_dic = emec.pd_ext.pd_combine_index(
        step_range_dic, 
        messu.loc[VIP_dicu[Ind_YM_f[0]]:VIP_dicu[Ind_YM_f[1]]]
        )
    sr_eva_con = messu.loc[VIP_messu[Ind_YM_r[0]]:VIP_messu[Ind_YM_r[1]]].index
    sr_eva_dic = emec.pd_ext.pd_combine_index(
        step_range_dic, 
        messu.loc[VIP_dicu[Ind_YM_r[0]]:VIP_dicu[Ind_YM_r[1]]]
        )
    # -------------------------------------------------------------------------------------
    #%%%% 6.4.1 Method A
    timings.loc[6.41]=time.perf_counter()
    log_custom("\n   Timing %f: %.5f s"%(timings.index[-1],
                                       timings.iloc[-1]-timings.iloc[0]),
                   **log_scopt)
    
    A0Al_ser = emeb.evaluation.YM_eva_method_A(
        stress_mid_ser=d_stress_mid,
        strain_mid_ser=d_strain_mid,
        comp=_opts['OPT_Compression'],
        name='A0Al', 
        det_opt='incremental'
        )
    A2Al_ser = emeb.evaluation.YM_eva_method_A(
        stress_mid_ser=d_stress_mid,
        strain_mid_ser=d_Strain_opt_d_A_mid,
        comp=_opts['OPT_Compression'],
        name='A2Al', 
        det_opt='incremental'
        )
    A2Sl_ser = emeb.evaluation.YM_eva_method_A(
        stress_mid_ser=d_stress_mid,
        strain_mid_ser=d_Strain_opt_d_S_mid,
        comp=_opts['OPT_Compression'],
        name='A2Sl', 
        det_opt='incremental'
        )
    A2Ml_ser = emeb.evaluation.YM_eva_method_A(
        stress_mid_ser=d_stress_mid,
        strain_mid_ser=d_Strain_opt_d_M_mid,
        comp=_opts['OPT_Compression'],
        name='A2Ml', 
        det_opt='incremental'
        )
    A4Al_ser = emeb.evaluation.YM_eva_method_A(
        stress_mid_ser=d_stress_mid,
        strain_mid_ser=d_Strain_opt_c_A_mid,
        comp=_opts['OPT_Compression'],
        name='A4Al', 
        det_opt='incremental'
        )
    A4Sl_ser = emeb.evaluation.YM_eva_method_A(
        stress_mid_ser=d_stress_mid,
        strain_mid_ser=d_Strain_opt_c_S_mid,
        comp=_opts['OPT_Compression'],
        name='A4Sl', 
        det_opt='incremental'
        )
    A4Ml_ser = emeb.evaluation.YM_eva_method_A(
        stress_mid_ser=d_stress_mid,
        strain_mid_ser=d_Strain_opt_c_M_mid,
        comp=_opts['OPT_Compression'],
        name='A4Ml', 
        det_opt='incremental'
        )
    E_A_df = pd.concat([A0Al_ser,
                        A2Al_ser, A2Sl_ser, A2Ml_ser,
                        A4Al_ser, A4Sl_ser, A4Ml_ser],axis=1)
    
    cols_con=E_A_df.columns.str.contains('0')
    E_A_con = emec.stat_ext.pd_agg(E_A_df.loc[sr_eva_con,cols_con])
    E_A_opt = emec.stat_ext.pd_agg(E_A_df.loc[sr_eva_dic,np.invert(cols_con)])
    E_A = pd.concat([E_A_con,E_A_opt],axis=1)

    if True:
        fig, (ax1,ax2) = plt.subplots(nrows=2, ncols=1, 
                                      sharex=False, sharey=False, 
                                      figsize = np.multiply(figsize,[1,2]))
        fig.suptitle('%s - Compare method A'%(plt_name))
        ax1.set_title('All Steps')
        ax1.set_xlabel('Step / -')
        ax1.set_ylabel('E / MPa')
        cc={'A0Al':'m:',
            'A2Al':'r:','A2Sl':'b:','A2Ml':'g:',
            'A4Al':'r--','A4Sl':'b--','A4Ml':'g--'}
        for k in cc:
            ax1.plot(E_A_df.loc[:VIP_messu['U']].index,
                     E_A_df.loc[:VIP_messu['U']][k],
                     cc[k], label='%s - %.2f MPa'%(k,E_A.at['mean',k]))
        ax1.axvline(x=VIP_messu['F3'], color='brown', linestyle=':')
        ax1.axvline(x=VIP_messu['F4'], color='brown', linestyle='--')
        ax1.axvline(x=VIP_dicu['F3'], color='olive', linestyle=':')
        ax1.axvline(x=VIP_dicu['F4'], color='olive', linestyle='--')
        ax1.legend()
        ax2.set_title('Improved determination range')
        ax2.set_xlabel('Step / -')
        ax2.set_ylabel('E / MPa')
        cr={'A0Al':sr_eva_con,
            'A2Al':sr_eva_dic,'A2Sl':sr_eva_dic,'A2Ml':sr_eva_dic,
            'A4Al':sr_eva_dic,'A4Sl':sr_eva_dic,'A4Ml':sr_eva_dic}
        for k in cc:
            ax2.plot(cr[k], E_A_df[k].loc[cr[k]], cc[k])
        ax2.axvline(x=VIP_messu['F3'], color='brown', linestyle=':')
        ax2.axvline(x=VIP_messu['F4'], color='brown', linestyle='--')
        ax2.axvline(x=VIP_dicu['F3'], color='olive', linestyle=':')
        ax2.axvline(x=VIP_dicu['F4'], color='olive', linestyle='--')
        plt_hsuf(fig,path=out_full+"-YM-Me_A",**plt_scopt)
        
    #least-square fit
    E_lsq_F_A0Al = emeb.evaluation.YM_eva_method_A(
        stress_mid_ser=messu.Stress,
        strain_mid_ser=messu.Strain,
        comp=_opts['OPT_Compression'],
        name='E_lsq_F_A0Al', 
        det_opt='leastsq',
        **{'ind_S':VIP_messu[Ind_YM_f[0]],
           'ind_E':VIP_messu[Ind_YM_f[1]]}
        )
    E_lsq_F_A0Al = pd.Series(
        E_lsq_F_A0Al, index=['E','E_abs','Rquad','Fit_result'],
        name='E_lsq_F_A0Al'
        )
    
    E_lsq_F_A2Al = emeb.evaluation.YM_eva_method_A(
        stress_mid_ser=messu.Stress,
        strain_mid_ser=messu.Strain_opt_d_A,
        comp=_opts['OPT_Compression'],
        name='E_lsq_F_A2Al', 
        det_opt='leastsq',
        **{'ind_S':VIP_dicu[Ind_YM_f[0]],
           'ind_E':VIP_dicu[Ind_YM_f[1]]}
        )
    E_lsq_F_A2Al = pd.Series(
        E_lsq_F_A2Al, index=['E','E_abs','Rquad','Fit_result'],
        name='E_lsq_F_A2Al'
        )
    E_lsq_F_A2Sl = emeb.evaluation.YM_eva_method_A(
        stress_mid_ser=messu.Stress,
        strain_mid_ser=messu.Strain_opt_d_S,
        comp=_opts['OPT_Compression'],
        name='E_lsq_F_A2Sl', 
        det_opt='leastsq',
        **{'ind_S':VIP_dicu[Ind_YM_f[0]],
           'ind_E':VIP_dicu[Ind_YM_f[1]]}
        )
    E_lsq_F_A2Sl = pd.Series(
        E_lsq_F_A2Sl, index=['E','E_abs','Rquad','Fit_result'],
        name='E_lsq_F_A2Sl'
        )
    E_lsq_F_A2Ml = emeb.evaluation.YM_eva_method_A(
        stress_mid_ser=messu.Stress,
        strain_mid_ser=messu.Strain_opt_d_M,
        comp=_opts['OPT_Compression'],
        name='E_lsq_F_A2Ml', 
        det_opt='leastsq',
        **{'ind_S':VIP_dicu[Ind_YM_f[0]],
           'ind_E':VIP_dicu[Ind_YM_f[1]]}
        )
    E_lsq_F_A2Ml = pd.Series(
        E_lsq_F_A2Ml, index=['E','E_abs','Rquad','Fit_result'],
         name='E_lsq_F_A2Ml'
         )
    
    E_lsq_F_A4Al = emeb.evaluation.YM_eva_method_A(
        stress_mid_ser=messu.Stress,
        strain_mid_ser=messu.Strain_opt_c_A,
        comp=_opts['OPT_Compression'],
        name='E_lsq_F_A4Al', 
        det_opt='leastsq',
        **{'ind_S':VIP_dicu[Ind_YM_f[0]],
           'ind_E':VIP_dicu[Ind_YM_f[1]]}
        )
    E_lsq_F_A4Al = pd.Series(
        E_lsq_F_A4Al, index=['E','E_abs','Rquad','Fit_result'],
        name='E_lsq_F_A4Al'
        )
    E_lsq_F_A4Sl = emeb.evaluation.YM_eva_method_A(
        stress_mid_ser=messu.Stress,
        strain_mid_ser=messu.Strain_opt_c_S,
        comp=_opts['OPT_Compression'],
        name='E_lsq_F_A4Sl', 
        det_opt='leastsq',
        **{'ind_S':VIP_dicu[Ind_YM_f[0]],
           'ind_E':VIP_dicu[Ind_YM_f[1]]}
        )
    E_lsq_F_A4Sl = pd.Series(
        E_lsq_F_A4Sl, index=['E','E_abs','Rquad','Fit_result'],
        name='E_lsq_F_A4Sl'
        )
    E_lsq_F_A4Ml = emeb.evaluation.YM_eva_method_A(
        stress_mid_ser=messu.Stress,
        strain_mid_ser=messu.Strain_opt_c_M,
        comp=_opts['OPT_Compression'],
        name='E_lsq_F_A4Ml', 
        det_opt='leastsq',
        **{'ind_S':VIP_dicu[Ind_YM_f[0]],
           'ind_E':VIP_dicu[Ind_YM_f[1]]}
        )
    E_lsq_F_A4Ml = pd.Series(
        E_lsq_F_A4Ml, index=['E','E_abs','Rquad','Fit_result'],
        name='E_lsq_F_A4Ml'
        )
        
    E_lsq_R_A0Al = emeb.evaluation.YM_eva_method_A(
        stress_mid_ser=messu.Stress,
        strain_mid_ser=messu.Strain,
        comp=_opts['OPT_Compression'],
        name='E_lsq_R_A0Al', 
        det_opt='leastsq',
        **{'ind_S':VIP_messu[Ind_YM_r[0]],
           'ind_E':VIP_messu[Ind_YM_r[1]]}
        )
    E_lsq_R_A0Al = pd.Series(
        E_lsq_R_A0Al, index=['E','E_abs','Rquad','Fit_result'],
        name='E_lsq_R_A0Al'
        )
    
    E_lsq_R_A2Al = emeb.evaluation.YM_eva_method_A(
        stress_mid_ser=messu.Stress,
        strain_mid_ser=messu.Strain_opt_d_A,
        comp=_opts['OPT_Compression'],
        name='E_lsq_R_A2Al', 
        det_opt='leastsq',
        **{'ind_S':VIP_dicu[Ind_YM_r[0]],
           'ind_E':VIP_dicu[Ind_YM_r[1]]}
        )
    E_lsq_R_A2Al = pd.Series(
        E_lsq_R_A2Al, index=['E','E_abs','Rquad','Fit_result'],
        name='E_lsq_R_A2Al'
        )
    E_lsq_R_A2Sl = emeb.evaluation.YM_eva_method_A(
        stress_mid_ser=messu.Stress,
        strain_mid_ser=messu.Strain_opt_d_S,
        comp=_opts['OPT_Compression'],
        name='E_lsq_R_A2Sl', 
        det_opt='leastsq',
        **{'ind_S':VIP_dicu[Ind_YM_r[0]],
           'ind_E':VIP_dicu[Ind_YM_r[1]]}
        )
    E_lsq_R_A2Sl = pd.Series(
        E_lsq_R_A2Sl, index=['E','E_abs','Rquad','Fit_result'],
        name='E_lsq_R_A2Sl'
        )
    E_lsq_R_A2Ml = emeb.evaluation.YM_eva_method_A(
        stress_mid_ser=messu.Stress,
        strain_mid_ser=messu.Strain_opt_d_M,
        comp=_opts['OPT_Compression'],
        name='E_lsq_R_A2Ml', 
        det_opt='leastsq',
        **{'ind_S':VIP_dicu[Ind_YM_r[0]],
           'ind_E':VIP_dicu[Ind_YM_r[1]]}
        )
    E_lsq_R_A2Ml = pd.Series(
        E_lsq_R_A2Ml, index=['E','E_abs','Rquad','Fit_result'],
        name='E_lsq_R_A2Ml'
        )
    
    E_lsq_R_A4Al = emeb.evaluation.YM_eva_method_A(
        stress_mid_ser=messu.Stress,
        strain_mid_ser=messu.Strain_opt_c_A,
        comp=_opts['OPT_Compression'],
        name='E_lsq_R_A4Al', 
        det_opt='leastsq',
        **{'ind_S':VIP_dicu[Ind_YM_r[0]],
           'ind_E':VIP_dicu[Ind_YM_r[1]]}
        )
    E_lsq_R_A4Al = pd.Series(
        E_lsq_R_A4Al, index=['E','E_abs','Rquad','Fit_result'],
        name='E_lsq_R_A4Al'
        )
    E_lsq_R_A4Sl = emeb.evaluation.YM_eva_method_A(
        stress_mid_ser=messu.Stress,
        strain_mid_ser=messu.Strain_opt_c_S,
        comp=_opts['OPT_Compression'],
        name='E_lsq_R_A4Sl', 
        det_opt='leastsq',
        **{'ind_S':VIP_dicu[Ind_YM_r[0]],
           'ind_E':VIP_dicu[Ind_YM_r[1]]}
        )
    E_lsq_R_A4Sl = pd.Series(
        E_lsq_R_A4Sl, index=['E','E_abs','Rquad','Fit_result'],
        name='E_lsq_R_A4Sl'
        )
    E_lsq_R_A4Ml = emeb.evaluation.YM_eva_method_A(
        stress_mid_ser=messu.Stress,
        strain_mid_ser=messu.Strain_opt_c_M,
        comp=_opts['OPT_Compression'],
        name='E_lsq_R_A4Ml', 
        det_opt='leastsq',
        **{'ind_S':VIP_dicu[Ind_YM_r[0]],
           'ind_E':VIP_dicu[Ind_YM_r[1]]}
        )
    E_lsq_R_A4Ml = pd.Series(
        E_lsq_R_A4Ml, index=['E','E_abs','Rquad','Fit_result'],
        name='E_lsq_R_A4Ml'
        )

    E_lsq_A = pd.concat([
        E_lsq_F_A0Al,
        E_lsq_F_A2Al, E_lsq_F_A2Sl, E_lsq_F_A2Ml,
        E_lsq_F_A4Al, E_lsq_F_A4Sl, E_lsq_F_A4Ml,
        E_lsq_R_A0Al,
        E_lsq_R_A2Al, E_lsq_R_A2Sl, E_lsq_R_A2Ml,
        E_lsq_R_A4Al, E_lsq_R_A4Sl, E_lsq_R_A4Ml
        ],axis=1)

    del A0Al_ser, A2Al_ser, A2Sl_ser, A2Ml_ser, A4Al_ser, A4Sl_ser, A4Ml_ser
    del E_lsq_F_A0Al, E_lsq_F_A2Al, E_lsq_F_A2Sl, E_lsq_F_A2Ml, E_lsq_F_A4Al, E_lsq_F_A4Sl, E_lsq_F_A4Ml
    del E_lsq_R_A0Al, E_lsq_R_A2Al, E_lsq_R_A2Sl, E_lsq_R_A2Ml, E_lsq_R_A4Al, E_lsq_R_A4Sl, E_lsq_R_A4Ml
    
    #%%%% 6.4.2 Method B
    timings.loc[6.42]=time.perf_counter()
    log_custom("\n   Timing %f: %.5f s"%(timings.index[-1],
                                       timings.iloc[-1]-timings.iloc[0]),
                   **log_scopt)
    B1Al_ser, B1Al_strain_ser = emeb.evaluation.YM_eva_method_B(
        option="Points", 
        stress_mid_ser=d_stress_mid,
        thickness = func_t(0.0), 
        Length = Length,
        P_df = P_xcoord_ydiff_meas, 
        P_fork_names = _opts['OPT_DIC_Points_meas_fork'],
        comp=_opts['OPT_Compression'],
        name='B1Al'
        )
    B1Sl_ser, B1Sl_strain_ser = emeb.evaluation.YM_eva_method_B(
        option="Points",
        stress_mid_ser=d_stress_mid,
        thickness = func_t(0.0), 
        Length = Length,
        P_df = P_xcoord_ydiff_meas_S, 
        P_fork_names = _opts['OPT_DIC_Points_meas_fork'],
        comp=_opts['OPT_Compression'],
        name='B1Sl'
        )
    B1Ml_ser, B1Ml_strain_ser = emeb.evaluation.YM_eva_method_B(
        option="Points",
        stress_mid_ser=d_stress_mid,
        thickness = func_t(0.0), 
        Length = Length,
        P_df = P_xcoord_ydiff_meas_M, 
        P_fork_names = _opts['OPT_DIC_Points_meas_fork'],
        comp=_opts['OPT_Compression'],
        name='B1Ml'
        )
    
    B2Al_ser, B2Al_strain_ser = emeb.evaluation.YM_eva_method_B(
        option="Fit",
        stress_mid_ser=d_stress_mid,
        thickness = func_t(0.0),
        Length=Length,
        w_func=bl['w_A']['d0'],
        w_params=Pre_inc_fit_df.loc(axis=1)['Fit_params_dict'],
        Length_det=10.0,
        comp=_opts['OPT_Compression'],
        name='B2Al'
        )
    B2Sl_ser, B2Sl_strain_ser = emeb.evaluation.YM_eva_method_B(
        option="Fit", 
        stress_mid_ser=d_stress_mid,
        thickness = func_t(0.0),
        Length=Length,
        w_func=bl['w_S']['d0'],
        w_params=Pre_inc_fit_df.loc(axis=1)['Fit_params_dict'],
        Length_det=10.0, 
        comp=_opts['OPT_Compression'],
        name='B2Sl'
        )
    B2Ml_ser, B2Ml_strain_ser = emeb.evaluation.YM_eva_method_B(
        option="Fit", 
        stress_mid_ser=d_stress_mid,
        thickness = func_t(0.0), 
        Length=Length,
        w_func=bl['w_M']['d0'],
        w_params=Bend_inc_fit_df.loc(axis=1)['Fit_params_dict'],
        Length_det=10.0, 
        comp = _opts['OPT_Compression'],
        name = 'B2Ml'
        )
    
    E_B_df = pd.concat([B1Al_ser,B1Sl_ser,B1Ml_ser,
                        B2Al_ser,B2Sl_ser,B2Ml_ser],axis=1)
    E_B = emec.stat_ext.pd_agg(E_B_df.loc[sr_eva_dic])
    
    if True:
        fig, (ax1,ax2) = plt.subplots(nrows=2, ncols=1, 
                                      sharex=False, sharey=False, 
                                      figsize = np.multiply(figsize,[1,2]))
        fig.suptitle('%s - Compare method B'%(plt_name))
        ax1.set_title('All Steps')
        ax1.set_xlabel('Step / -')
        ax1.set_ylabel('E / MPa')
        cc={'B1Al':'r:','B1Sl':'b:','B1Ml':'g:',
            'B2Al':'r--','B2Sl':'b--','B2Ml':'g--'}
        for k in cc:
            ax1.plot(E_B_df.loc[:VIP_messu['U']].index,
                     E_B_df.loc[:VIP_messu['U']][k],
                     cc[k], label='%s - %.2f MPa'%(k,E_B.at['mean',k]))
        ax1.axvline(x=VIP_dicu['F3'], color='olive', linestyle=':')
        ax1.axvline(x=VIP_dicu['F4'], color='olive', linestyle='--')
        ax1.legend()
        ax2.set_title('Improved determination range')
        ax2.set_xlabel('Step / -')
        ax2.set_ylabel('E / MPa')
        for k in cc:
            ax2.plot(sr_eva_dic, E_B_df[k].loc[sr_eva_dic], cc[k])
        plt_hsuf(fig,path=out_full+"-YM-Me_B",**plt_scopt)
        
    # least-square-fit
    E_lsq_F_B1Al = emeb.evaluation.YM_eva_method_B(
        option="Points",
        stress_mid_ser=messu.Stress,
        thickness = func_t(0.0),
        Length = Length,
        P_df = P_xcoord_ydisp_meas,
        P_fork_names = _opts['OPT_DIC_Points_meas_fork'],
        comp = _opts['OPT_Compression'],
        name='E_lsq_F_B1Al',
        det_opt='leastsq',
        **{'ind_S':VIP_dicu[Ind_YM_f[0]],
           'ind_E':VIP_dicu[Ind_YM_f[1]]}
        )
    E_lsq_F_B1Al = pd.Series(
        E_lsq_F_B1Al, index=['E','E_abs','Rquad','Fit_result','strain'],
        name='E_lsq_F_B1Al'
        )
    E_lsq_F_B1Sl = emeb.evaluation.YM_eva_method_B(
        option="Points",
        stress_mid_ser=messu.Stress,
        thickness = func_t(0.0),
        Length=Length,
        P_df = P_xcoord_ydisp_meas_S,
        P_fork_names = _opts['OPT_DIC_Points_meas_fork'],
        comp=_opts['OPT_Compression'],
        name='E_lsq_F_B1Sl',
        det_opt='leastsq',
        **{'ind_S':VIP_dicu[Ind_YM_f[0]],
           'ind_E':VIP_dicu[Ind_YM_f[1]]}
        )
    E_lsq_F_B1Sl = pd.Series(
        E_lsq_F_B1Sl, index=['E','E_abs','Rquad','Fit_result','strain'],
        name='E_lsq_F_B1Sl'
        )
    E_lsq_F_B1Ml = emeb.evaluation.YM_eva_method_B(
        option="Points",
        stress_mid_ser=messu.Stress,
        thickness = func_t(0.0),
        Length=Length,
        P_df = P_xcoord_ydisp_meas_M,
        P_fork_names = _opts['OPT_DIC_Points_meas_fork'],
        comp=_opts['OPT_Compression'],
        name='E_lsq_F_B1Ml',
        det_opt='leastsq',
        **{'ind_S':VIP_dicu[Ind_YM_f[0]],
           'ind_E':VIP_dicu[Ind_YM_f[1]]}
        )
    E_lsq_F_B1Ml = pd.Series(
        E_lsq_F_B1Ml, index=['E','E_abs','Rquad','Fit_result','strain'],
        name='E_lsq_F_B1Ml'
        )
    
    E_lsq_F_B2Al = emeb.evaluation.YM_eva_method_B(
        option="Fit", 
        stress_mid_ser=messu.Stress,
        thickness = func_t(0.0),
        Length=Length,
        w_func=bl['w_A']['d0'],
        w_params=Pre_fit_df.loc(axis=1)['Fit_params_dict'],
        Length_det=10.0,
        comp=_opts['OPT_Compression'],
        name='E_lsq_F_B2Al',
        det_opt='leastsq',
        **{'ind_S':VIP_dicu[Ind_YM_f[0]],
           'ind_E':VIP_dicu[Ind_YM_f[1]]}
        )
    E_lsq_F_B2Al = pd.Series(
        E_lsq_F_B2Al,index=['E','E_abs','Rquad','Fit_result','strain'],
        name='E_lsq_F_B2Al'
        )
    E_lsq_F_B2Sl = emeb.evaluation.YM_eva_method_B(
        option="Fit", 
        stress_mid_ser=messu.Stress,
        thickness = func_t(0.0),
        Length=Length,
        w_func=bl['w_S']['d0'],
        w_params=Pre_fit_df.loc(axis=1)['Fit_params_dict'],
        Length_det=10.0,
        comp=_opts['OPT_Compression'],
        name='E_lsq_F_B2Sl',
        det_opt='leastsq',
        **{'ind_S':VIP_dicu[Ind_YM_f[0]],
           'ind_E':VIP_dicu[Ind_YM_f[1]]}
        )
    E_lsq_F_B2Sl = pd.Series(
        E_lsq_F_B2Sl,index=['E','E_abs','Rquad','Fit_result','strain'],
        name='E_lsq_F_B2Sl'
        )
    E_lsq_F_B2Ml = emeb.evaluation.YM_eva_method_B(
        option="Fit", 
        stress_mid_ser=messu.Stress,
        thickness = func_t(0.0),
        Length=Length,
        w_func=bl['w_M']['d0'], 
        w_params=Bend_fit_df.loc(axis=1)['Fit_params_dict'],
        Length_det=10.0, 
        comp=_opts['OPT_Compression'],
        name='E_lsq_F_B2Ml',
        det_opt='leastsq',
        **{'ind_S':VIP_dicu[Ind_YM_f[0]],
           'ind_E':VIP_dicu[Ind_YM_f[1]]}
        )
    E_lsq_F_B2Ml = pd.Series(
        E_lsq_F_B2Ml,index=['E','E_abs','Rquad','Fit_result','strain'],
        name='E_lsq_F_B2Ml'
        )
    
    E_lsq_R_B1Al = emeb.evaluation.YM_eva_method_B(
        option="Points",
        stress_mid_ser=messu.Stress,
        thickness = func_t(0.0),
        Length = Length,
        P_df = P_xcoord_ydisp_meas,
        P_fork_names = _opts['OPT_DIC_Points_meas_fork'],
        comp = _opts['OPT_Compression'],
        name='E_lsq_R_B1Al',
        det_opt='leastsq',
        **{'ind_S':VIP_dicu[Ind_YM_r[0]],
           'ind_E':VIP_dicu[Ind_YM_r[1]]}
        )
    E_lsq_R_B1Al = pd.Series(
        E_lsq_R_B1Al, index=['E','E_abs','Rquad','Fit_result','strain'],
        name='E_lsq_R_B1Al'
        )
    E_lsq_R_B1Sl = emeb.evaluation.YM_eva_method_B(
        option="Points",
        stress_mid_ser=messu.Stress,
        thickness = func_t(0.0),
        Length=Length,
        P_df = P_xcoord_ydisp_meas_S,
        P_fork_names = _opts['OPT_DIC_Points_meas_fork'],
        comp=_opts['OPT_Compression'],
        name='E_lsq_R_B1Sl',
        det_opt='leastsq',
        **{'ind_S':VIP_dicu[Ind_YM_r[0]],
           'ind_E':VIP_dicu[Ind_YM_r[1]]}
        )
    E_lsq_R_B1Sl = pd.Series(
        E_lsq_R_B1Sl, index=['E','E_abs','Rquad','Fit_result','strain'],
        name='E_lsq_R_B1Sl'
        )
    E_lsq_R_B1Ml = emeb.evaluation.YM_eva_method_B(
        option="Points",
        stress_mid_ser=messu.Stress,
        thickness = func_t(0.0),
        Length=Length,
        P_df = P_xcoord_ydisp_meas_M,
        P_fork_names = _opts['OPT_DIC_Points_meas_fork'],
        comp=_opts['OPT_Compression'],
        name='E_lsq_R_B1Ml',
        det_opt='leastsq',
        **{'ind_S':VIP_dicu[Ind_YM_r[0]],
           'ind_E':VIP_dicu[Ind_YM_r[1]]}
        )
    E_lsq_R_B1Ml = pd.Series(
        E_lsq_R_B1Ml, index=['E','E_abs','Rquad','Fit_result','strain'],
        name='E_lsq_R_B1Ml'
        )
    
    E_lsq_R_B2Al = emeb.evaluation.YM_eva_method_B(
        option="Fit", 
        stress_mid_ser=messu.Stress,
        thickness = func_t(0.0),
        Length=Length,
        w_func=bl['w_A']['d0'],
        w_params=Pre_fit_df.loc(axis=1)['Fit_params_dict'],
        Length_det=10.0,
        comp=_opts['OPT_Compression'],
        name='E_lsq_R_B2Al',
        det_opt='leastsq',
        **{'ind_S':VIP_dicu[Ind_YM_r[0]],
           'ind_E':VIP_dicu[Ind_YM_r[1]]}
        )
    E_lsq_R_B2Al = pd.Series(
        E_lsq_R_B2Al,index=['E','E_abs','Rquad','Fit_result','strain'],
        name='E_lsq_R_B2Al'
        )
    E_lsq_R_B2Sl = emeb.evaluation.YM_eva_method_B(
        option="Fit", 
        stress_mid_ser=messu.Stress,
        thickness = func_t(0.0),
        Length=Length,
        w_func=bl['w_S']['d0'],
        w_params=Pre_fit_df.loc(axis=1)['Fit_params_dict'],
        Length_det=10.0,
        comp=_opts['OPT_Compression'],
        name='E_lsq_R_B2Sl',
        det_opt='leastsq',
        **{'ind_S':VIP_dicu[Ind_YM_r[0]],
           'ind_E':VIP_dicu[Ind_YM_r[1]]}
        )
    E_lsq_R_B2Sl = pd.Series(
        E_lsq_R_B2Sl,index=['E','E_abs','Rquad','Fit_result','strain'],
        name='E_lsq_R_B2Sl'
        )
    E_lsq_R_B2Ml = emeb.evaluation.YM_eva_method_B(
        option="Fit", 
        stress_mid_ser=messu.Stress,
        thickness = func_t(0.0),
        Length=Length,
        w_func=bl['w_M']['d0'], 
        w_params=Bend_fit_df.loc(axis=1)['Fit_params_dict'],
        Length_det=10.0, 
        comp=_opts['OPT_Compression'],
        name='E_lsq_R_B2Ml',
        det_opt='leastsq',
        **{'ind_S':VIP_dicu[Ind_YM_r[0]],
           'ind_E':VIP_dicu[Ind_YM_r[1]]}
        )
    E_lsq_R_B2Ml = pd.Series(
        E_lsq_R_B2Ml,index=['E','E_abs','Rquad','Fit_result','strain'],
        name='E_lsq_R_B2Ml'
        )
        
    E_lsq_B = pd.concat([
        E_lsq_F_B1Al,E_lsq_F_B1Sl,E_lsq_F_B1Ml,
        E_lsq_F_B2Al,E_lsq_F_B2Sl,E_lsq_F_B2Ml,
        E_lsq_R_B1Al,E_lsq_R_B1Sl,E_lsq_R_B1Ml,
        E_lsq_R_B2Al,E_lsq_R_B2Sl,E_lsq_R_B2Ml
        ],axis=1)
    
    del B1Al_strain_ser,B1Sl_strain_ser,B1Ml_strain_ser,B2Al_strain_ser,B2Sl_strain_ser,B2Ml_strain_ser
    del B1Al_ser,B1Sl_ser,B1Ml_ser,B2Al_ser,B2Sl_ser,B2Ml_ser
    del E_lsq_F_B1Al,E_lsq_F_B1Sl,E_lsq_F_B1Ml
    del E_lsq_F_B2Al,E_lsq_F_B2Sl,E_lsq_F_B2Ml
    del E_lsq_R_B1Al,E_lsq_R_B1Sl,E_lsq_R_B1Ml
    del E_lsq_R_B2Al,E_lsq_R_B2Sl,E_lsq_R_B2Ml

    #%%%% 6.4.3 Method C
    timings.loc[6.43]=time.perf_counter()
    log_custom("\n   Timing %f: %.5f s"%(timings.index[-1],
                                       timings.iloc[-1]-timings.iloc[0]),
                   **log_scopt)    
    
    E_C2Al_ser = emeb.evaluation.YM_eva_method_C(
        Force_ser=d_Force, w_func=bl['w_A']['d0'], 
        w_params=Pre_inc_fit_df.loc(axis=1)['Fit_params_dict'],
        length=Length, I_func=func_I, A_func=func_A,
        CS_type='Rectangle', kappa=None,
        poisson=_opts['OPT_Poisson_prediction'],
        comp=_opts['OPT_Compression'], option="M", name="C2Al"
        )
    E_C2Sl_ser = emeb.evaluation.YM_eva_method_C(
        Force_ser=d_Force, w_func=bl['w_S']['d0'], 
        w_params=Pre_inc_fit_df.loc(axis=1)['Fit_params_dict'],
        length=Length, I_func=func_I, A_func=func_A,
        CS_type='Rectangle', kappa=None,
        poisson=_opts['OPT_Poisson_prediction'],
        comp=_opts['OPT_Compression'], option="M", name="C2Sl"
        )
    E_C2Ml_ser = emeb.evaluation.YM_eva_method_C(
        Force_ser=d_Force, w_func=bl['w_M']['d0'], 
        w_params=Bend_inc_fit_df.loc(axis=1)['Fit_params_dict'],
        length=Length, I_func=func_I, A_func=func_A,
        CS_type='Rectangle', kappa=None,
        poisson=_opts['OPT_Poisson_prediction'],
        comp=_opts['OPT_Compression'], option="M", name="C2Ml"
        )
    E_C2Cl_ser = emeb.evaluation.YM_eva_method_C(
        Force_ser=d_Force, w_func=bl['w_S']['d0'], 
        w_params=Pre_inc_fit_df.loc(axis=1)['Fit_params_dict'],
        length=Length, I_func=func_I, A_func=func_A,
        CS_type='Rectangle', kappa=None,
        poisson=_opts['OPT_Poisson_prediction'],
        comp=_opts['OPT_Compression'], option="M+V", name="C2Cl"
        )
    
    E_C_df = pd.concat([E_C2Al_ser,E_C2Sl_ser,E_C2Ml_ser,E_C2Cl_ser],axis=1)
    E_C = emec.stat_ext.pd_agg(E_C_df.loc[sr_eva_dic])
    
    if True:    
        fig, (ax1,ax2) = plt.subplots(nrows=2, ncols=1, 
                                      sharex=False, sharey=False, 
                                      figsize = np.multiply(figsize,[1,2]))
        fig.suptitle('%s - Compare method C'%(plt_name))
        ax1.set_title('All Steps')
        ax1.set_xlabel('Step / -')
        ax1.set_ylabel('E / MPa')
        cc={'C2Al':'r:','C2Sl':'b:','C2Ml':'m--','C2Cl':'g--'}
        for k in cc:
            ax1.plot(E_C_df.loc[:VIP_messu['U']].index,
                     E_C_df.loc[:VIP_messu['U']][k],
                     cc[k], label='%s - %.2f MPa'%(k,E_C.at['mean',k]))
        ax1.axvline(x=VIP_dicu['F3'], color='olive', linestyle=':')
        ax1.axvline(x=VIP_dicu['F4'], color='olive', linestyle='--')
        ax1.legend()
        ax2.set_title('Improved determination range')
        ax2.set_xlabel('Step / -')
        ax2.set_ylabel('E / MPa')
        for k in cc:
            ax2.plot(sr_eva_dic,E_C_df[k].loc[sr_eva_dic], cc[k])
        plt_hsuf(fig,path=out_full+"-YM-Me_C",**plt_scopt)

    del E_C2Al_ser, E_C2Sl_ser, E_C2Ml_ser
    
    #%%%% 6.4.4 Method D
    timings.loc[6.44]=time.perf_counter()
    log_custom("\n   Timing %f: %.5f s"%(timings.index[-1],
                                       timings.iloc[-1]-timings.iloc[0]),
                   **log_scopt)    
    
    D1Agwt,D1Agwt_df=emeb.evaluation.YM_eva_method_D(
        P_df=P_xcoord_ydiff_meas, Force_ser=d_Force,
        rel_steps = step_range_dic_inc,
        Length=Length, func_I=func_I, n=100,
        weighted=True, weight_func=emeb.evaluation.Weight_func,
        wargs=['Custom_cut', bl['w_A']['d0']],
        wkwargs=Pre_inc_fit_df.loc(axis=1)['Fit_params_dict'],
        pb_b=True, name='D1Agwt'
        )  
    D1Aguw,D1Aguw_df=emeb.evaluation.YM_eva_method_D(
        P_df=P_xcoord_ydiff_meas, Force_ser=d_Force,
        rel_steps = step_range_dic_inc,
        Length=Length, func_I=func_I, n=100,
        weighted=True, weight_func=emeb.evaluation.Weight_func,
        wkwargs={'option':'Cut',
                 'xmin':Bo_Le,'xmax':Bo_Ri},
        pb_b=True, name='D1Aguw'
        )
    D1Sgwt,D1Sgwt_df=emeb.evaluation.YM_eva_method_D(
        P_df=P_xcoord_ydiff_meas_S, Force_ser=d_Force,
        rel_steps = step_range_dic_inc,
        Length=Length, func_I=func_I, n=100,
        weighted=True, weight_func=emeb.evaluation.Weight_func,
        wargs=['Custom_cut', bl['w_S']['d0']],
        wkwargs=Pre_inc_fit_df.loc(axis=1)['Fit_params_dict'],
        pb_b=True, name='D1Sgwt'
        )  
    D1Sguw,D1Sguw_df=emeb.evaluation.YM_eva_method_D(
        P_df=P_xcoord_ydiff_meas_S, Force_ser=d_Force,
        rel_steps = step_range_dic_inc,
        Length=Length, func_I=func_I, n=100,
        weighted=True, weight_func=emeb.evaluation.Weight_func,
        wkwargs={'option':'Cut',
                 'xmin':Bo_Le,'xmax':Bo_Ri},
        pb_b=True, name='D1Sguw'
        )
    D1Mgwt,D1Mgwt_df=emeb.evaluation.YM_eva_method_D(
        P_df=P_xcoord_ydiff_meas_M, Force_ser=d_Force,
        rel_steps = step_range_dic_inc,
        Length=Length, func_I=func_I, n=100,
        weighted=True, weight_func=emeb.evaluation.Weight_func,
        wargs=['Custom_cut', bl['w_M']['d0']],
        wkwargs=Bend_inc_fit_df.loc(axis=1)['Fit_params_dict'],
        pb_b=True, name='D1Mgwt'
        )  
    D1Mguw,D1Mguw_df=emeb.evaluation.YM_eva_method_D(
        P_df=P_xcoord_ydiff_meas_M, Force_ser=d_Force,
        rel_steps = step_range_dic_inc,
        Length=Length, func_I=func_I, n=100,
        weighted=True, weight_func=emeb.evaluation.Weight_func,
        wkwargs={'option':'Cut',
                 'xmin':Bo_Le,'xmax':Bo_Ri},
        pb_b=True, name='D1Mguw'
        )
    
    dftmp = emeb.opt_mps.Point_df_from_lin(xlin,step_range_dic)
    Diff_A_d0 = bl['w_A']['d0'](xlin,Pre_inc_fit_df.loc(axis=1)['Fit_params_dict'])
    A_d0_inc_df = emeb.opt_mps.Point_df_combine(dftmp,Diff_A_d0)
    Diff_S_d0 = bl['w_S']['d0'](xlin,Pre_inc_fit_df.loc(axis=1)['Fit_params_dict'])
    S_d0_inc_df = emeb.opt_mps.Point_df_combine(dftmp,Diff_S_d0)
    Diff_M_d0 = bl['w_M']['d0'](xlin,Bend_inc_fit_df.loc(axis=1)['Fit_params_dict'])
    M_d0_inc_df = emeb.opt_mps.Point_df_combine(dftmp,Diff_M_d0)
    D2Agwt,D2Agwt_df=emeb.evaluation.YM_eva_method_D(
        P_df=A_d0_inc_df,Force_ser=d_Force,
        rel_steps = step_range_dic_inc,
        Length=Length, func_I=func_I, n=100,
        weighted=True, weight_func=emeb.evaluation.Weight_func,
        wargs=['Custom_cut', bl['w_A']['d0']],
        wkwargs=Pre_inc_fit_df.loc(axis=1)['Fit_params_dict'],
        pb_b=True, name='D2Agwt'
        )
    D2Aguw,D2Aguw_df=emeb.evaluation.YM_eva_method_D(
        P_df=A_d0_inc_df,Force_ser=d_Force,
        rel_steps = step_range_dic_inc,
        Length=Length, func_I=func_I, n=100,
        weighted=True, weight_func=emeb.evaluation.Weight_func,
        wkwargs={'option':'Cut',
                 'xmin':Bo_Le,'xmax':Bo_Ri},
        pb_b=True, name='D2Aguw'
        )
    D2Sgwt,D2Sgwt_df=emeb.evaluation.YM_eva_method_D(
        P_df=S_d0_inc_df,Force_ser=d_Force,
        rel_steps = step_range_dic_inc,
        Length=Length, func_I=func_I, n=100,
        weighted=True, weight_func=emeb.evaluation.Weight_func,
        wargs=['Custom_cut', bl['w_S']['d0']],
        wkwargs=Pre_inc_fit_df.loc(axis=1)['Fit_params_dict'],
        pb_b=True, name='D2Sgwt'
        )
    D2Sguw,D2Sguw_df=emeb.evaluation.YM_eva_method_D(
        P_df=S_d0_inc_df,Force_ser=d_Force,
        rel_steps = step_range_dic_inc,
        Length=Length, func_I=func_I, n=100,
        weighted=True, weight_func=emeb.evaluation.Weight_func,
        wkwargs={'option':'Cut',
                 'xmin':Bo_Le,'xmax':Bo_Ri},
        pb_b=True, name='D2Sguw'
        )
    D2Mgwt,D2Mgwt_df=emeb.evaluation.YM_eva_method_D(
        P_df=M_d0_inc_df,Force_ser=d_Force,
        rel_steps = step_range_dic_inc,
        Length=Length, func_I=func_I, n=100,
        weighted=True, weight_func=emeb.evaluation.Weight_func,
        wargs=['Custom_cut', bl['w_M']['d0']],
        wkwargs=Bend_inc_fit_df.loc(axis=1)['Fit_params_dict'],
        pb_b=True, name='D2Mgwt'
        )
    D2Mguw,D2Mguw_df=emeb.evaluation.YM_eva_method_D(
        P_df=M_d0_inc_df,Force_ser=d_Force,
        rel_steps = step_range_dic_inc,
        Length=Length, func_I=func_I, n=100,
        weighted=True, weight_func=emeb.evaluation.Weight_func,
        wkwargs={'option':'Cut',
                 'xmin':Bo_Le,'xmax':Bo_Ri},
        pb_b=True, name='D2Mguw'
        )
    
    E_D_df = pd.concat([D1Agwt,D1Aguw,D1Sgwt,D1Sguw,D1Mgwt,D1Mguw,
                        D2Agwt,D2Aguw,D2Sgwt,D2Sguw,D2Mgwt,D2Mguw],axis=1)
    E_D = emec.stat_ext.pd_agg(E_D_df.loc[sr_eva_dic])
    
    if True:        
        fig, (ax1,ax2) = plt.subplots(nrows=2, ncols=1, 
                                      sharex=False, sharey=False, 
                                      figsize = np.multiply(figsize,[1,2]))
        fig.suptitle('%s - Compare method D'%(plt_name))
        ax1.set_title('All Steps')
        ax1.set_xlabel('Step / -')
        ax1.set_ylabel('E / MPa')
        cc={'D1Agwt':'r:','D1Sgwt':'b:','D1Mguw':'m:','D1Mgwt':'g:',
            'D2Agwt':'r--','D2Sgwt':'b--','D2Mguw':'m--','D2Mgwt':'g--'}
        for k in cc:
            ax1.plot(E_D_df.loc[:VIP_messu['U']].index,
                     E_D_df.loc[:VIP_messu['U']][k],
                     cc[k], label='%s - %.2f MPa'%(k,E_D.at['mean',k]))
        ax1.axvline(x=VIP_dicu['F3'], color='olive', linestyle=':')
        ax1.axvline(x=VIP_dicu['F4'], color='olive', linestyle='--')
        ax1.legend()
        ax2.set_title('Improved determination range')
        ax2.set_xlabel('Step / -')
        ax2.set_ylabel('E / MPa')
        for k in cc:
            ax2.plot(sr_eva_dic,E_D_df[k].loc[sr_eva_dic],
                     cc[k], label='%s - %.2f MPa'%(k,E_D.at['mean',k]))
        plt_hsuf(fig,path=out_full+"-YM-Me_D",**plt_scopt)
    
    del D1Agwt,D1Aguw,D1Sgwt,D1Sguw,D1Mgwt,D1Mguw
    del D2Agwt,D2Aguw,D2Sgwt,D2Sguw,D2Mgwt,D2Mguw
    del D1Agwt_df,D1Aguw_df,D1Sgwt_df,D1Sguw_df,D1Mgwt_df,D1Mguw_df
    del D2Agwt_df,D2Aguw_df,D2Sgwt_df,D2Sguw_df,D2Mgwt_df,D2Mguw_df
    
    #%%%% 6.4.5 Method E
    timings.loc[6.45]=time.perf_counter()
    log_custom("\n   Timing %f: %.5f s"%(timings.index[-1],
                                       timings.iloc[-1]-timings.iloc[0]),
                   **log_scopt)    
    
    E4A = emeb.evaluation.YM_eva_method_E(
        Force_ser=d_Force, length=Length,
        func_curve=bl['w_A']['d2'], 
        params_curve=Pre_inc_fit_df.loc(axis=1)['Fit_params_dict'],
        func_MoI=func_I,
        func_thick=func_t, evopt=1/2,
        opt_det='all', n=100,
        opt_g='strain', opt_g_lim=0.5, name='E4A'
        )
    E4A_E_df, E4A_sig_eps_df, E4A_E_to_x, E4A_stress_df, E4A_strain_df, E4A_E_to_x_g = E4A

    E4S = emeb.evaluation.YM_eva_method_E(
        Force_ser=d_Force, length=Length,
        func_curve=bl['w_S']['d2'], 
        params_curve=Pre_inc_fit_df.loc(axis=1)['Fit_params_dict'],
        func_MoI=func_I,
        func_thick=func_t, evopt=1/2,
        opt_det='all', n=100,
        opt_g='strain', opt_g_lim=0.5, name='E4S'
        )
    E4S_E_df, E4S_sig_eps_df, E4S_E_to_x, E4S_stress_df, E4S_strain_df, E4S_E_to_x_g = E4S

    E4M = emeb.evaluation.YM_eva_method_E(
        Force_ser=d_Force, length=Length,
        func_curve=bl['w_M']['d2'], 
        params_curve=Bend_inc_fit_df.loc(axis=1)['Fit_params_dict'],
        func_MoI=func_I, 
        func_thick=func_t, evopt=1/2,
        opt_det='all', n=100,
        opt_g='strain', opt_g_lim=0.5, name='E4M'
        )
    E4M_E_df, E4M_sig_eps_df, E4M_E_to_x, E4M_stress_df, E4M_strain_df, E4M_E_to_x_g = E4M

    E_E_df = pd.concat([E4A_E_df, E4S_E_df, E4M_E_df],axis=1)
    E_E = emec.stat_ext.pd_agg(E_E_df.loc[sr_eva_dic])
    
    if True:
        fig, (ax1,ax2,ax3) = plt.subplots(nrows=3, ncols=1, 
                                          sharex=False, sharey=False, 
                                          figsize = np.multiply(figsize,[1,3]))
        fig.suptitle("%s - M - Stress, Strain, Youngs-Modulus to x-coordinate for F3 to F4"%(plt_name))
        ax1 = emeb.plotting.colplt_common_ax(
            xdata=E4M_stress_df.columns,
            ydata=E4M_stress_df,
            step_range=sr_eva_dic, ax=ax1,
            title='Stress to x over Increment',
            xlabel='x / mm', 
            ylabel='$\sigma$ / MPa'
            )
        ax1.axvline(x=0, color='gray', linestyle='--')
        ax2 = emeb.plotting.colplt_common_ax(
            xdata=E4M_strain_df.columns,
            ydata=E4M_strain_df,
            step_range=sr_eva_dic, ax=ax2,
            title='Strain to x over Increment',
            xlabel='x / mm', 
            ylabel='$\sigma$ / MPa'
            )
        ax2.axvline(x=0, color='gray', linestyle='--')
        ax3 = emeb.plotting.colplt_common_ax(
            xdata=E4M_E_to_x_g.columns,
            ydata=E4M_E_to_x_g,
            step_range=sr_eva_dic, ax=ax3,
            title='Youngs Modulus to x over Increment',
            xlabel='x / mm',
            ylabel='E / MPa'
            )
        ax3.axvline(x=0, color='gray', linestyle='--')
        plt_hsuf(fig,path=None,**plt_scopt)
        
        fig, (ax1,ax2) = plt.subplots(nrows=2, ncols=1, 
                                      sharex=False, sharey=False, 
                                      figsize = np.multiply(figsize,[1,2]))
        fig.suptitle('%s - Compare method E'%(plt_name))
        ax1.set_title('All Steps')
        ax1.set_xlabel('Step / -')
        ax1.set_ylabel('E / MPa')
        cc={'E4Sl':'r:','E4Se':'b:','E4Sg':'g:',
            'E4Ml':'r--','E4Me':'b--','E4Mg':'g--'}
        for k in cc:
            ax1.plot(E_E_df.loc[:VIP_messu['U']].index,
                     E_E_df.loc[:VIP_messu['U']][k],
                     cc[k], label='%s - %.2f MPa'%(k,E_E.at['mean',k]))
        ax1.axvline(x=VIP_dicu['F3'], color='olive', linestyle=':')
        ax1.axvline(x=VIP_dicu['F4'], color='olive', linestyle='--')
        ax1.legend()
        ax2.set_title('Improved determination range')
        ax2.set_xlabel('Step / -')
        ax2.set_ylabel('E / MPa')
        for k in cc:
            ax2.plot(sr_eva_dic,E_E_df[k].loc[sr_eva_dic], cc[k])
        plt_hsuf(fig,path=out_full+"-YM-Me_E",**plt_scopt)
        
    del E4A, E4A_E_df, E4A_sig_eps_df, E4A_E_to_x, E4A_stress_df, E4A_strain_df, E4A_E_to_x_g
    del E4S, E4S_E_df, E4S_sig_eps_df, E4S_E_to_x, E4S_stress_df, E4S_strain_df, E4S_E_to_x_g
    del E4M, E4M_E_df, E4M_sig_eps_df, E4M_E_to_x, E4M_stress_df, E4M_strain_df, E4M_E_to_x_g

    #%%%% 6.4.6 Method F
    timings.loc[6.46]=time.perf_counter()
    log_custom("\n   Timing %f: %.5f s"%(timings.index[-1],
                                       timings.iloc[-1]-timings.iloc[0]),
                   **log_scopt)    
    
    F4A_df = emeb.evaluation.YM_eva_method_F(
        c_func=bl['w_A']['d2'],
        c_params=Pre_inc_fit_df.loc(axis=1)['Fit_params_dict'],
        Force_ser=d_Force, Length=Length, func_I=func_I,
        weighted=True, weight_func=emeb.evaluation.Weight_func,
        wargs=['Custom_cut', bl['w_A']['d0']],
        wkwargs=Pre_inc_fit_df.loc(axis=1)['Fit_params_dict'],
        xr_dict = {'fu':1/1, 'ha':1/2, 'th':1/3},
        pb_b=True, name='F4Ag', n=100
        )
    
    F4S_df = emeb.evaluation.YM_eva_method_F(
        c_func=bl['w_S']['d2'],
        c_params=Pre_inc_fit_df.loc(axis=1)['Fit_params_dict'],
        Force_ser=d_Force, Length=Length, func_I=func_I,
        weighted=True, weight_func=emeb.evaluation.Weight_func,
        wargs=['Custom_cut', bl['w_S']['d0']],
        wkwargs=Pre_inc_fit_df.loc(axis=1)['Fit_params_dict'],
        xr_dict = {'fu':1/1, 'ha':1/2, 'th':1/3},
        pb_b=True, name='F4Sg', n=100
        )
    
    F4M_df = emeb.evaluation.YM_eva_method_F(
        c_func=bl['w_M']['d2'],
        c_params=Bend_inc_fit_df.loc(axis=1)['Fit_params_dict'],
        Force_ser=d_Force, Length=Length, func_I=func_I,
        weighted=True, weight_func=emeb.evaluation.Weight_func,
        wargs=['Custom_cut', bl['w_M']['d0']],
        wkwargs=Bend_inc_fit_df.loc(axis=1)['Fit_params_dict'],
        xr_dict = {'fu':1/1, 'ha':1/2, 'th':1/3},
        pb_b=True, name='F4Mg', n=100
        )
    
    E_F_df = pd.concat([F4A_df, F4S_df, F4M_df],axis=1)
    E_F = emec.stat_ext.pd_agg(E_F_df.loc[sr_eva_dic])
    
    if True:    
        cc={'F4Agha':'r:', 'F4Agth':'r--',
            'F4Sgha':'b:', 'F4Sgth':'b--',
            'F4Mgha':'g:', 'F4Mgth':'g--'}
        fig, (ax1,ax2) = plt.subplots(nrows=2, ncols=1, 
                                      sharex=False, sharey=False, 
                                      figsize = np.multiply(figsize,[1,2]))
        fig.suptitle('%s - Compare method F'%(plt_name))
        ax1.set_title('All Steps')
        ax1.set_xlabel('Step / -')
        ax1.set_ylabel('E / MPa')
        for k in cc:
            ax1.plot(E_F_df.loc[:VIP_messu['U']].index,
                     E_F_df.loc[:VIP_messu['U']][k],
                     cc[k], label='%s - %.2f MPa'%(k,E_F.at['mean',k]))
        ax1.axvline(x=VIP_dicu['F3'], color='olive', linestyle=':')
        ax1.axvline(x=VIP_dicu['F4'], color='olive', linestyle='--')
        ax1.legend()
        ax2.set_title('Improved determination range')
        ax2.set_xlabel('Step / -')
        ax2.set_ylabel('E / MPa')
        for k in cc:
            ax2.plot(sr_eva_dic,E_F_df[k].loc[sr_eva_dic], cc[k])
        plt_hsuf(fig,path=out_full+"-YM-Me_F",**plt_scopt)
        
    del F4A_df, F4S_df, F4M_df
    
    #%%%% 6.4.7 Method G
    timings.loc[6.47]=time.perf_counter()
    log_custom("\n   Timing %f: %.5f s"%(timings.index[-1],
                                       timings.iloc[-1]-timings.iloc[0]),
                   **log_scopt)    
    
    E_G3Ag_ser = emeb.evaluation.YM_eva_method_G(
        Force_ser=d_Force,
        w_func_f_0=bl['w_A']['d0'], 
        w_params=Pre_inc_fit_df.loc(axis=1)['Fit_params_dict'],
        c_func=bl['w_A']['d2'],
        r_func=None,
        c_params=Pre_inc_fit_df.loc(axis=1)['Fit_params_dict'],
        r_params=None,
        length=Length, I_func=func_I, A_func=func_A,
        CS_type='Rectangle', kappa=None,
        poisson=_opts['OPT_Poisson_prediction'],
        comp=_opts['OPT_Compression'],
        option="ignore_V", name="G3Ag"
        )
    E_G3Sg_ser = emeb.evaluation.YM_eva_method_G(
        Force_ser=d_Force,
        w_func_f_0=bl['w_S']['d0'], 
        w_params=Pre_inc_fit_df.loc(axis=1)['Fit_params_dict'],
        c_func=bl['w_S']['d2'],
        r_func=None,
        c_params=Pre_inc_fit_df.loc(axis=1)['Fit_params_dict'],
        r_params=None,
        length=Length, I_func=func_I, A_func=func_A,
        CS_type='Rectangle', kappa=None,
        poisson=_opts['OPT_Poisson_prediction'],
        comp=_opts['OPT_Compression'],
        option="ignore_V", name="G3Sg"
        )
    E_G3Mg_ser = emeb.evaluation.YM_eva_method_G(
        Force_ser=d_Force,
        w_func_f_0=bl['w_M']['d0'], 
        w_params=Pre_inc_fit_df.loc(axis=1)['Fit_params_dict'],
        c_func=bl['w_M']['d2'],
        r_func=None,
        c_params=Bend_inc_fit_df.loc(axis=1)['Fit_params_dict'],
        r_params=None,
        length=Length, I_func=func_I, A_func=func_A,
        CS_type='Rectangle', kappa=None,
        poisson=_opts['OPT_Poisson_prediction'],
        comp=_opts['OPT_Compression'],
        option="ignore_V", name="G3Mg"
        )
    E_G3Cg_ser = emeb.evaluation.YM_eva_method_G(
        Force_ser=d_Force,
        w_func_f_0=bl['w_S']['d0'], 
        w_params=Pre_inc_fit_df.loc(axis=1)['Fit_params_dict'],
        c_func=bl['w_M']['d2'],
        r_func=bl['w_V']['d1'],
        c_params=Bend_inc_fit_df.loc(axis=1)['Fit_params_dict'],
        r_params=Pre_inc_fit_df.loc(axis=1)['Fit_params_dict'],
        length=Length, I_func=func_I, A_func=func_A,
        CS_type='Rectangle', kappa=None,
        poisson=_opts['OPT_Poisson_prediction'],
        comp=_opts['OPT_Compression'],
        option="M+V", name="G3Cg"
        )
    E_G_df = pd.concat([E_G3Ag_ser, E_G3Sg_ser, E_G3Mg_ser, E_G3Cg_ser],axis=1)
    E_G = emec.stat_ext.pd_agg(E_G_df.loc[sr_eva_dic])
    
    if True:    
        fig, (ax1,ax2) = plt.subplots(nrows=2, ncols=1, 
                                      sharex=False, sharey=False, 
                                      figsize = np.multiply(figsize,[1,2]))
        fig.suptitle('%s - Compare method G'%(plt_name))
        ax1.set_title('All Steps')
        ax1.set_xlabel('Step / -')
        ax1.set_ylabel('E / MPa')
        # cc={'G3Sg':'r:','G3Mg':'b--'}
        cc={'G3Ag':'r:', 'G3Sg':'b:', 'G3Mg':'m--', 'G3Cg':'g--'}
        for k in cc:
            ax1.plot(E_G_df.loc[:VIP_messu['U']].index,
                     E_G_df.loc[:VIP_messu['U']][k],
                     cc[k], label='%s - %.2f MPa'%(k,E_G.at['mean',k]))
        ax1.axvline(x=VIP_dicu['F3'], color='olive', linestyle=':')
        ax1.axvline(x=VIP_dicu['F4'], color='olive', linestyle='--')
        ax1.legend()
        ax2.set_title('Improved determination range')
        ax2.set_xlabel('Step / -')
        ax2.set_ylabel('E / MPa')
        for k in cc:
            ax2.plot(sr_eva_dic,E_G_df[k].loc[sr_eva_dic], cc[k])
        plt_hsuf(fig,path=out_full+"-YM-Me_G",**plt_scopt)
    
    del E_G3Ag_ser, E_G3Sg_ser, E_G3Mg_ser, E_G3Cg_ser

    #%%%% 6.4.8 Method compare
    timings.loc[6.48]=time.perf_counter()
    log_custom("\n   Timing %f: %.5f s"%(timings.index[-1],
                                       timings.iloc[-1]-timings.iloc[0]),
                   **log_scopt)    

    E_lsq=pd.concat([E_lsq_A, E_lsq_B],axis=1)

    E_Methods_df = pd.concat([
        E_A_df, E_B_df, E_C_df, E_D_df, E_E_df, E_F_df, E_G_df
        ],axis=1)
    E_agg_funcs = ['mean',emec.stat_ext.meanwoso,'median',
                   'std',emec.stat_ext.stdwoso,
                   emec.stat_ext.cv, emec.stat_ext.cvwoso,
                   'min','max']
    
    cols_con=E_Methods_df.columns.str.contains('0')
    E_inc_F_comp_con = E_Methods_df.loc[sf_eva_con,cols_con].agg(E_agg_funcs)
    E_inc_F_comp_opt = E_Methods_df.loc[sf_eva_dic,np.invert(cols_con)].agg(E_agg_funcs)
    E_inc_F_comp = pd.concat([E_inc_F_comp_con,E_inc_F_comp_opt],axis=1)

    cols_con=E_Methods_df.columns.str.contains('0')
    E_inc_R_comp_con = E_Methods_df.loc[sr_eva_con,cols_con].agg(E_agg_funcs)
    E_inc_R_comp_opt = E_Methods_df.loc[sr_eva_dic,np.invert(cols_con)].agg(E_agg_funcs)
    E_inc_R_comp = pd.concat([E_inc_R_comp_con,E_inc_R_comp_opt],axis=1)
    
    log_custom("\n\n  Method comaparison:",**log_scoptf)
    log_custom("\n  - least square fit",**log_scoptf)
    log_custom(log_cind('\n'+E_lsq.loc[['E','Rquad']].T.to_string()),
                   **log_scoptf)
    log_custom("\n\n  - incremental - fixed range:",
                   **log_scoptf)
    log_custom(log_cind('\n'+E_inc_F_comp.T.to_string()),
                   **log_scoptf)
    log_custom(log_cind('\n'+E_inc_F_comp.agg(['idxmax','idxmin'],axis=1).to_string()),
                   **log_scoptf)
    log_custom("\n\n  - incremental - refined range:",**log_scoptf)
    log_custom(log_cind('\n'+E_inc_R_comp.T.to_string()),
                   **log_scoptf)
    log_custom(log_cind('\n'+E_inc_R_comp.agg(['idxmax','idxmin'],axis=1).to_string()),
                   **log_scoptf)

    check_M_tmp=E_Methods_df.loc(axis=1)[E_Methods_df.columns.str.contains('M|Cl|Ce|Cg')]
    check_M_dict={i: check_M_tmp[i] for i in check_M_tmp.columns}
    Check_M = emeb.evaluation.YM_check_many_with_method_D(
        E_dict=check_M_dict, F=d_Force,
        Length=Length,I_func=func_I,
        w_func=bl['w_M']['d0'],
        w_params=Bend_inc_fit_df['Fit_params_dict'],
        rel_steps=sr_eva_dic, n=100, pb_b=False, name='M'
        )
    check_MtoD_g, check_MtoD_x, check_M, w_D_to_M = Check_M
    
    log_custom("\n\n  Check Methods with D (M-Displacement deviation):",
                   **log_scoptf)
    log_custom("\n  - mean over all (except first and last):",
                   **log_scoptf)
    log_custom(log_cind(check_MtoD_g.mean()),
                   **log_scoptf)
    log_custom("\n  - only in mid:",
                   **log_scoptf)
    log_custom(log_cind(check_MtoD_x.mean()),
                   **log_scoptf)
    
    check_S_tmp=E_Methods_df.loc(axis=1)[E_Methods_df.columns.str.contains('S')]
    check_S_dict={i: check_S_tmp[i] for i in check_S_tmp.columns}
    Check_S = emeb.evaluation.YM_check_many_with_method_D(
        E_dict=check_S_dict, F=d_Force,
        Length=Length,I_func=func_I,
        w_func=bl['w_S']['d0'],
        w_params=Pre_inc_fit_df['Fit_params_dict'],
        rel_steps=sr_eva_dic,
        n=100, pb_b=False, name='S'
        )
    check_StoD_g, check_StoD_x, check_S, w_D_to_S = Check_S
    
    log_custom("\n\n  Check Methods with D (S-Displacement deviation):",
                   **log_scoptf)
    log_custom("\n  - mean over all (except first and last):",
                   **log_scoptf)
    log_custom(log_cind(check_StoD_g.mean()),
                   **log_scoptf)
    log_custom("\n  - only in mid:",
                   **log_scoptf)
    log_custom(log_cind(check_StoD_x.mean()),
                   **log_scoptf)
    
    check_A_tmp=E_Methods_df.loc(axis=1)[E_Methods_df.columns.str.contains('Al|Ae|Ag')]
    check_A_dict={i: check_A_tmp[i] for i in check_A_tmp.columns}
    Check_A = emeb.evaluation.YM_check_many_with_method_D(
        E_dict=check_A_dict, F=d_Force,
        Length=Length,I_func=func_I,
        w_func=bl['w_A']['d0'],
        w_params=Pre_inc_fit_df['Fit_params_dict'],
        rel_steps=sr_eva_dic,
        n=100, pb_b=False, name='A'
        )
    check_AtoD_g, check_AtoD_x, check_A, w_D_to_A = Check_A
    
    log_custom("\n\n  Check Methods with D (A-Displacement deviation):",
                   **log_scoptf)
    log_custom("\n  - mean over all (except first and last):",
                   **log_scoptf)
    log_custom(log_cind(check_AtoD_g.mean()),
                   **log_scoptf)
    log_custom("\n  - only in mid:",
                   **log_scoptf)
    log_custom(log_cind(check_AtoD_x.mean()),
                   **log_scoptf)
    
    Check_to_D = pd.Series({'MtoD_g':check_MtoD_g, 'MtoD_x': check_MtoD_x,
                            'M':check_M, 'MwD': w_D_to_M,
                            'StoD_g':check_StoD_g, 'StoD_x': check_StoD_x,
                            'S':check_S, 'SwD': w_D_to_S,
                            'AtoD_g':check_AtoD_g, 'AtoD_x': check_AtoD_x,
                            'A':check_A, 'AwD': w_D_to_A},dtype='O')
    
    # set preffered Method
    YM_pref_con=E_lsq_A['E_lsq_R_A0Al']
    if loc_Yd_tmp.startswith('E_lsq'):
        YM_pref_opt=E_lsq[loc_Yd_tmp]
    elif loc_Yd_tmp.startswith('E_inc_R'):
        YM_pref_opt=emec.fitting.Refit_YM_vals(
            m_df=messu, 
            YM = E_inc_R_comp[loc_Yd_tmp.split('_')[-1]]['meanwoso'], 
            VIP=VIP_dicu,
            n_strain=dic_used_Strain, n_stress='Stress',
            n_loBo=['F3'], n_upBo=['F4']
            )
    elif loc_Yd_tmp.startswith('E_inc_F'):
        YM_pref_opt=emec.fitting.Refit_YM_vals(
            m_df=messu, 
            YM = E_inc_F_comp[loc_Yd_tmp.split('_')[-1]]['meanwoso'], 
            VIP=VIP_dicu,
            n_strain=dic_used_Strain, n_stress='Stress',
            n_loBo=['F1'], n_upBo=['F2']
            )
    else:
        raise NotImplementedError('Prefered YM-method not selectable!')
    # --------------------------------------------------------------------------
    #%%% 6.5 Determine yield point
    log_custom("\n "+"-"*100,**log_scopt)
    log_custom("\n ### -6.5 Determine yield point ###",**log_scopt)
    timings.loc[6.5]=time.perf_counter()
    log_custom("\n   Timing %f: %.5f s"%(timings.index[-1],
                                       timings.iloc[-1]-timings.iloc[0]),
                   **log_scopt)    
    

    strain_osd = {'YK':0.0,'Y0':0.0,'Y1':0.007/100,'Y2':0.1/100,'Y':0.2/100} #acc. Zhang et al. 2021, DOI: 10.1007/s10439-020-02719-2
    strain_osdf={'YK':'F4'}
        
    log_custom("\n  Determination of yield strain-conventional:",**log_scoptf)
    tmp=emec.mc_yield.Yield_redet2_Multi(
        m_df=messu, VIP=VIP_messu,
        strain_osd=strain_osd, strain_osdf=strain_osdf,
        n_strain='Strain', n_stress='Stress',
        n_loBo=['F3'], n_upBo=['U'], n_loBo_int=['F3'],
        YM     = YM_pref_con['E'],
        YM_abs = YM_pref_con['E_abs'],
        use_rd =True, 
        rise_det=[
            _opts['OPT_Rise_Smoothing'][0],
            _opts['OPT_Rise_Smoothing'][1]
            ], 
        ywhere='n'
        )
    VIP_messu, yield_df_con, txt = tmp
    log_custom(log_cind(txt,3), **log_scoptf)
    if _opts['OPT_DIC']:
        log_custom("\n  Determination of yield strain-optical:",**log_scoptf)
        tmp=emec.mc_yield.Yield_redet2_Multi(
            m_df=messu, VIP=VIP_dicu,
            strain_osd=strain_osd, strain_osdf=strain_osdf,
            n_strain=dic_used_Strain, n_stress='Stress',
            n_loBo=['F3'], n_upBo=['U'], n_loBo_int=['F3'],
            YM     = YM_pref_opt['E'],
            YM_abs = YM_pref_opt['E_abs'],
            use_rd =True, 
            rise_det=[
                _opts['OPT_Rise_Smoothing'][0],
                _opts['OPT_Rise_Smoothing'][1]
                ], 
            ywhere='n'
            )
        VIP_dicu, yield_df_opt, txt = tmp
        log_custom(log_cind(txt,3), **log_scoptf)
        
    fig, (ax1,ax2) = plt.subplots(nrows=2, ncols=1, 
                                  sharex=False, sharey=False, 
                                  figsize = np.multiply(figsize,[1,2]))
    fig.suptitle('%s - Stress vs. strain curve - yield point determination'%plt_name)
    ax1.set_title('Conventional strain')
    ax1.set_xlabel('Strain / -')
    ax1.set_ylabel('Stress / MPa')
    ax1.plot(messu.loc[:VIP_messu['B']]['Strain'], messu.loc[:VIP_messu['B']]['Stress'], 'r--',label='con')
    tmp=ax1.get_xlim(),ax1.get_ylim()
    for i in strain_osd.keys():
        ax1.plot(emec.fitting.strain_linfit(
            [0,1000],
            YM_pref_con['E'], 
            YM_pref_con['E_abs'], 
            strain_offset=strain_osd[i]
            ),[0,1000], '-',label='$E_{off-%.3fp}$'%(strain_osd[i]*100))
    ax1.set_xlim(tmp[0])
    ax1.set_ylim(tmp[1])
    a, b=messu.Strain[VIP_messu[:'B']],messu.Stress[VIP_messu[:'B']]
    j=np.int64(-1)
    ax1.plot(a, b, 'bx')
    for x in VIP_messu[:'B'].index:
        j+=1
        if j%2: c=(6,-6)
        else:   c=(-6,6)
        ax1.annotate('%s' % x, xy=(a.iloc[j],b.iloc[j]), xycoords='data',
                      xytext=c, ha="center", va="center", textcoords='offset points')
    ax1.legend()
    ax2.set_title('Optical strain')
    if _opts['OPT_DIC']:
        ax2.set_xlabel('Strain / -')
        ax2.set_ylabel('Stress / MPa')
        ax2.plot(messu.loc[:VIP_dicu['B']][dic_used_Strain], messu.loc[:VIP_dicu['B']]['Stress'], 
                 'r--',label='opt')
        tmp=ax2.get_xlim(),ax1.get_ylim()
        for i in strain_osd.keys():
            ax2.plot(emec.fitting.strain_linfit(
                [0,1000],
                YM_pref_opt['E'], 
                YM_pref_opt['E_abs'], 
                strain_offset=strain_osd[i]
                ),[0,1000], '-',label='$E_{off-%.3fp}$'%(strain_osd[i]*100))
        ax2.set_xlim(tmp[0])
        ax2.set_ylim(tmp[1])
        a, b=messu[dic_used_Strain][VIP_dicu[:'B']],messu.Stress[VIP_dicu[:'B']]
        j=np.int64(-1)
        ax2.plot(a, b, 'bx')
        for x in VIP_dicu[:'B'].index:
            j+=1
            if j%2: c=(6,-6)
            else:   c=(-6,6)
            ax2.annotate('%s' % x, xy=(a.iloc[j],b.iloc[j]), xycoords='data',
                          xytext=c, ha="center", va="center", textcoords='offset points')
        ax2.legend()
    plt_hsuf(fig,path=out_full+"-sigeps_yielddet",**plt_scopt)
    
    # ============================================================================
    #%%% 6.6 Determine final curve
    log_custom("\n "+"-"*100,**log_scopt)
    log_custom("\n ### -6.6 Determine final stress-strain curve ###",**log_scopt)
    timings.loc[6.6]=time.perf_counter()
    log_custom("\n   Timing %f: %.5f s"%(timings.index[-1],
                                       timings.iloc[-1]-timings.iloc[0]),
                   **log_scopt)
    FinSSC = True # finale kurve für werteermittlung auf Ym linearisieren an anfang
    if FinSSC:
        messu_FP,linstrainos_con=emec.mc_man.DetFinSSC(
            mdf=messu, YM=YM_pref_con, 
            iS=VIP_messu['F3'], iLE=None,
            StressN='Stress', StrainN='Strain', 
            addzero=True, izero=VIP_messu['S']
            )
        yield_df_con['Strain']=yield_df_con['Strain']-linstrainos_con
        yield_df_con['Force']=yield_df_con['Stress']/emeb.evaluation.stress_perF(
            0.0,func_I,func_t,Bo_Le,Bo_Ri
            )
        yield_df_con['Way']=(yield_df_con['Strain']*Length**2)/(6*prot_ser['thickness_2'])
        log_custom("\n   Strain offset (conventional) about %.5f"%(linstrainos_con),
                   **log_scopt)
        if _opts['OPT_DIC']:
            tmp, linstrainos_opt=emec.mc_man.DetFinSSC(
                mdf=messu, YM=YM_pref_opt, 
                iS=VIP_dicu['F3'], iLE=None,
                StressN='Stress', StrainN=dic_used_Strain, 
                addzero=True, izero=VIP_messu['S']
                )
            messu_FP=messu_FP.join(tmp, how='outer', lsuffix='', rsuffix='_opt')
            messu_FP['Stress'].fillna(messu_FP['Stress_opt'],inplace=True)
            yield_df_opt[dic_used_Strain]=yield_df_opt[dic_used_Strain]-linstrainos_opt
            del messu_FP['Stress_opt']
            yield_df_opt['Force']=yield_df_opt['Stress']/emeb.evaluation.stress_perF(
                0.0,func_I,func_t,Bo_Le,Bo_Ri
                )
            yield_df_opt[dic_used_Disp]=(yield_df_opt[dic_used_Strain]*Length**2)/(6*prot_ser['thickness_2'])
        log_custom("\n   Strain offset (optical) about %.5f"%(linstrainos_opt),**log_scopt)
    
        messu_FP['Force']=messu_FP['Stress']/emeb.evaluation.stress_perF(
            0.0,func_I,func_t,Bo_Le,Bo_Ri
            )
        messu_FP['Way']=(messu_FP['Strain']*Length**2)/(6*prot_ser['thickness_2'])
        if _opts['OPT_DIC']: 
            messu_FP[dic_used_Disp]=(messu_FP[dic_used_Strain]*Length**2)/(6*prot_ser['thickness_2'])
    else:
        messu_FP =  messu
        linstrainos_con = 0
        linstrainos_opt = 0
        log_custom("\n   No linear start of final stress-strain-curve",**log_scopt)
        
    # ============================================================================
    #%% 7 Outputs
    log_custom("\n "+"="*100,**log_scopt)
    log_custom("\n ### 7 Outputs ###",**log_scoptf)
    timings.loc[7.0]=time.perf_counter()
    log_custom("\n   Timing %f: %.5f s"%(timings.index[-1],
                                       timings.iloc[-1]-timings.iloc[0]),
                   **log_scopt)    
    # ============================================================================
    #%%% 7.1 Prepare outputs
    timings.loc[7.1]=time.perf_counter()
    log_custom("\n   Timing %f: %.5f s"%(timings.index[-1],
                                       timings.iloc[-1]-timings.iloc[0]),
                   **log_scopt)    

    out_tab               = pd.Series([],name=prot_ser.name,dtype='float64')
    out_tab['Date_eva']   = datetime.now().strftime('%d.%m.%Y')
    out_tab['geo_MoI_max'] = func_I(xlin).max()
    out_tab['geo_MoI_min'] = func_I(xlin).min()
    out_tab['geo_MoI_mid'] = func_I(0.0)
    if _opts['OPT_DIC']:
        out_tab['geo_curve_max'] = geo_d2_max
        out_tab['geo_curve_min'] = geo_d2_min
        out_tab['geo_curve_mid'] = geo_d2_mid
        
        t=bl['w_I']['d0'](np.array([Bo_Le,Bo_Ri]),
                          Pre_fit_df.loc(axis=1)['Fit_params_dict'])
        steps = emec.pd_ext.pd_combine_index(
            messu.loc[VIP_messu['S']:VIP_messu['U']],
            Pre_fit_df
            )[[0,-1]]
        
        # tl, tr = t.loc[[VIP_messu['S']+1,VIP_messu['U']]].diff().iloc[-1].values
        tl, tr = t.loc[steps].diff().iloc[-1].values
        out_tab['ind_U_l'],       out_tab['ind_U_r'] = tl, tr
        tl, tr = t.loc[sf_eva_con[[0,-1]]].diff().iloc[-1].values
        out_tab['ind_Fcon_l'], out_tab['ind_Fcon_r'] = tl, tr
        tl, tr = t.loc[sf_eva_dic[[0,-1]]].diff().iloc[-1].values
        out_tab['ind_Fopt_l'], out_tab['ind_Fopt_r'] = tl, tr
        tl, tr = t.loc[sr_eva_con[[0,-1]]].diff().iloc[-1].values
        out_tab['ind_Rcon_l'], out_tab['ind_Rcon_r'] = tl, tr
        tl, tr = t.loc[sr_eva_dic[[0,-1]]].diff().iloc[-1].values
        out_tab['ind_Ropt_l'], out_tab['ind_Ropt_r'] = tl, tr
    
    out_tab['leos_con']   = linstrainos_con
    if _opts['OPT_DIC']: out_tab['leos_opt']   = linstrainos_opt

    relVS = [*np.sort([*strain_osd.keys()]),'U','B']
    tmp=emec.output.Otvalgetter_Multi(
        messu_FP, Vs=relVS,
        datasep=['con','opt'], 
        VIPs={'con':VIP_messu,'opt':VIP_dicu}, 
        exacts={'con':yield_df_con,'opt':yield_df_opt}, 
        orders={'con':['f','e','U'],'opt':['e','U']}, 
        add_esufs={'con':'_con','opt':'_opt'},
        n_strains={'con':'Strain','opt':dic_used_Strain}, 
        n_stresss={'con':'Stress','opt':'Stress'},
        use_exacts=True
        )
    tmp.name=prot_ser.name
    out_tab=out_tab.append(tmp)
    tmp=emec.output.Otvalgetter_Multi(
        messu_FP, Vs=relVS,
        datasep=['con','opt'], 
        VIPs={'con':VIP_messu,'opt':VIP_dicu}, 
        exacts={'con':yield_df_con,'opt':yield_df_opt}, 
        orders={'con':['F','s','W'],'opt':['s','W']}, 
        add_esufs={'con':'_con','opt':'_opt'},
        n_strains={'con':'Way','opt': dic_used_Disp}, 
        n_stresss={'con':'Force','opt':'Force'},
        use_exacts=True
        )
    tmp.name=prot_ser.name
    out_tab=out_tab.append(tmp)
    # ============================================================================
    #%%% 7.2 Generate plots
    timings.loc[7.2]=time.perf_counter()
    log_custom("\n   Timing %f: %.5f s"%(timings.index[-1],
                                       timings.iloc[-1]-timings.iloc[0]),
                   **log_scopt)    
        
    fig, ax1 = plt.subplots()
    ax1.set_title('%s - Analyzing meas. force'%plt_name)
    color1 = 'tab:red'
    ax1.set_xlabel('Time / s')
    ax1.set_ylabel('Force / N', color=color1)
    ax1.plot(messu.Time, messu.Force, 'r-', label='Force')
    a, b=messu.Time[VIP_messu],messu.Force[VIP_messu]
    j=np.int64(-1)
    ax1.plot(a, b, 'bx')
    for x in VIP_messu.index:
        j+=1
        if j%2: c=(6,-6)
        else:   c=(-6,6)
        ax1.annotate('%s' % x, xy=(a.iloc[j],b.iloc[j]), xycoords='data',
                     xytext=c, ha="center", va="center", textcoords='offset points')
        # ax1.annotate('%s' % x, xy=(a.iloc[j],b.iloc[j]), xycoords='data',
        #              xytext=(5, -5), textcoords='offset points')
    ax1.tick_params(axis='y', labelcolor=color1)
    #ax1.xticks([x for x in range(100) if x%10==0])
    ax2 = ax1.twinx()  # instantiate a second axes that shares the same x-axis
    ax2.grid(False)
    color2 = 'tab:blue'
    ax2.set_ylabel('Rise / curve /  (N/s)', color=color2)
    ax2.plot(messu.Time, messu.driF, 'b:', label='Force-rise')
    ax2.plot(messu.Time, messu.dcuF, 'g:', label='Force-curve')
    ax2.tick_params(axis='y', labelcolor=color2)
    fig.legend(loc='lower right', bbox_to_anchor=(0.85, 0.15))
    plt_hsuf(fig,path=out_full+"-Fdricu",**plt_scopt)
    
    if _opts['OPT_DIC']:
        fig, ax1 = plt.subplots()
        ax1.set_title('%s - Stress vs. strain curve of different opt. strain calculations'%plt_name)
        ax1.set_xlabel('Strain / -')
        ax1.set_ylabel('Stress / MPa')
        ax1.plot(messu.loc[:VIP_messu['B']]['Strain_opt_d_A'], 
                 messu.loc[:VIP_messu['B']]['Stress'], 
                 'r:',label='displacement A')
        ax1.plot(messu.loc[:VIP_messu['B']]['Strain_opt_d_S'], 
                 messu.loc[:VIP_messu['B']]['Stress'], 
                 'b:',label='displacement S')
        ax1.plot(messu.loc[:VIP_messu['B']]['Strain_opt_d_M'],
                 messu.loc[:VIP_messu['B']]['Stress'],
                 'g:',label='displacement M')
        ax1.plot(messu.loc[:VIP_messu['B']]['Strain_opt_c_A'],
                 messu.loc[:VIP_messu['B']]['Stress'],
                 'r--',label='curvature A')
        ax1.plot(messu.loc[:VIP_messu['B']]['Strain_opt_c_S'],
                 messu.loc[:VIP_messu['B']]['Stress'],
                 'b--',label='curvature S')
        ax1.plot(messu.loc[:VIP_messu['B']]['Strain_opt_c_M'],
                 messu.loc[:VIP_messu['B']]['Stress'],
                 'g--',label='curvature M')
        fig.legend(loc=[0.45, 0.135])
        # ftxt=('$E_{curve}$ = % 8.3f MPa '%(ED2_vgl1),
        #       '$E_{fork-C}$ = % 8.3f MPa '%(ED2_vgl2),
        #       '$E_{fork-P}$ = % 8.3f MPa '%(ED2_vgl3),
        #       '$E_{curve-P4O}$ = % 8.3f MPa '%(ED2_vgl4))
        # fig.text(0.97,0.15,'\n'.join(ftxt),
        #          ha='right',va='bottom', bbox=dict(boxstyle='round', edgecolor='0.8', facecolor='white', alpha=0.8))
        plt_hsuf(fig,path=out_full+"-sigeps_dicvgl",**plt_scopt)
    
        # Pre-Fit -----------------------------------------------------------------
        plot_range_dic=messu.loc[
            VIP_dicu[(VIP_dicu[['F1','F3']]).idxmin()]:VIP_dicu[(VIP_dicu[['F2','F4']]).idxmax()]
            ].index
        cbtick=VIP_dicu.to_dict()
        emeb.plotting.colplt_funcs_all(
            x=xlin, func_cohort=bl['w_A'],
            params=Pre_fit_df.loc(axis=1)['Fit_params_dict'],
            step_range=plot_range_dic, 
            title=('%s - Fit-full - evaluation range'%plt_name),
            xlabel='x / mm',
            Point_df=P_xcoord_ydisp_meas,
            cblabel='VIP', cbtick=cbtick,
            path=out_full+'-DIC_fit-A-eva',
            plt_scopt=plt_scopt
            )
        # Bending-Fit -------------------------------------------------------------
        emeb.plotting.colplt_funcs_all(
            x=xlin, func_cohort=bl['w_M'],
            params=Bend_fit_df.loc(axis=1)['Fit_params_dict'],
            step_range=plot_range_dic, 
            title=('%s - Fit-Bending - evaluation range'%plt_name),
            xlabel='x / mm',
            Point_df=P_xcoord_ydisp_meas_M,
            cblabel='VIP', cbtick=cbtick,
            path=out_full+'-DIC_fit-M-eva',
            plt_scopt=plt_scopt
            )
        # Pre-Fit -----------------------------------------------------------------
        emeb.plotting.colplt_funcs_all(
            x=xlin, func_cohort=bl['w_A'],
            params=Pre_inc_fit_df.loc(axis=1)['Fit_params_dict'],
            step_range=plot_range_dic, 
            title=('%s - Incremental Fit-full - evaluation range'%plt_name),
            xlabel='x / mm',
            Point_df=P_xcoord_ydiff_meas,
            cblabel='VIP', cbtick=cbtick,
            path=out_full+'-INC_fit-A-eva',
            plt_scopt=plt_scopt
            )
        # Bending-Fit -------------------------------------------------------------
        emeb.plotting.colplt_funcs_all(
            x=xlin, func_cohort=bl['w_M'],
            params=Bend_inc_fit_df.loc(axis=1)['Fit_params_dict'],
            step_range=plot_range_dic, 
            title=('%s - Incremental Fit-Bending - evaluation range'%plt_name),
            xlabel='x / mm',
            Point_df=P_xcoord_ydiff_meas_M,
            cblabel='VIP', cbtick=cbtick,
            path=out_full+'-INC_fit-M-eva',
            plt_scopt=plt_scopt
            )

    fig, ax1 = plt.subplots()
    ax1.set_title('%s - Stress vs. strain curve with labels'%plt_name)
    ax1.set_xlabel('Strain / -')
    ax1.set_ylabel('Stress / MPa')
    ax1.plot(messu.loc[:VIP_messu['B']]['Strain'],
             messu.loc[:VIP_messu['B']]['Stress'], 'r--',label='con')
    if _opts['OPT_DIC']:
        ax1.plot(messu.loc[:VIP_messu['B']][dic_used_Strain], 
                 messu.loc[:VIP_messu['B']]['Stress'], 'm--',label='opt')
    t=messu.Strain.loc[[VIP_messu['F3'] , VIP_messu['F4']]].values
    t=np.array([max(0,t[0]-0.1*(t[1]-t[0])),
                min(messu.Strain.max(),t[1]+0.1*(t[1]-t[0]))])
    # ax1.plot(t, np.polyval(E2_pf_tmp[0][:],t), 'g-',label='$E_{con}$')
    # ax1.plot(t, E2_fit.eval(x=t), 'g-',label='$E_{con}$')
    ax1.plot(t, YM_pref_con['E']*t+YM_pref_con['E_abs'],
             'g-',label='$E_{con}$')
    a, b=messu.Strain[VIP_messu[:'B']],messu.Stress[VIP_messu[:'B']]
    j=np.int64(-1)
    ax1.plot(a, b, 'bx')
    for x in VIP_messu[:'B'].index:
        j+=1
        if j%2: c=(6,-6)
        else:   c=(-6,6)
        ax1.annotate('%s' % x, xy=(a.iloc[j],b.iloc[j]), xycoords='data',
                      xytext=c, ha="center", va="center", textcoords='offset points')
    if _opts['OPT_DIC']:
        a,b=messu.loc[VIP_dicu[:'B'],dic_used_Strain],messu.Stress[VIP_dicu[:'B']]
        j=np.int64(-1)
        ax1.plot(a, b, 'yx')
        t=messu.loc[[VIP_dicu['F3'] , VIP_dicu['F4']],dic_used_Strain].values
        t=np.array([
            max(0,t[0]-0.1*(t[1]-t[0])),
            min(messu.loc[:,dic_used_Strain].max(),t[1]+0.1*(t[1]-t[0]))
            ])
        # ax1.plot(t, np.polyval(ED2_pf_tmp[0][:],t), 'b-',label='$E_{opt}$')
        # ax1.plot(t, ED2_fit.eval(x=t), 'b-',label='$E_{opt}$')
        ax1.plot(t, YM_pref_opt['E']*t+YM_pref_opt['E_abs'],
             'b-',label='$E_{opt}$')
    ftxt=('$f_{y}$ = % 6.3f MPa ($\epsilon_{y}$ = %4.3f %%)'%(
            out_tab['fy'],
            out_tab['ey_opt']*100
            ),
          '$f_{u}$ = % 6.3f MPa ($\epsilon_{u}$ = %4.3f %%)'%(
            out_tab['fu'],
            out_tab['eu_opt']*100
            ),
          '$E_{con}$ = % 8.3f MPa ($R²$ = %4.3f)'%(*YM_pref_con[['E','Rquad']],),
          '$E_{opt}$ = % 8.3f MPa ($R²$ = %4.3f)'%(*YM_pref_opt[['E','Rquad']],))
    fig.text(0.95,0.15,'\n'.join(ftxt),
              ha='right',va='bottom', 
              bbox=dict(boxstyle='round', edgecolor='0.8', 
                        facecolor='white', alpha=0.8))
    fig.legend(loc='upper left', bbox_to_anchor=(0.10, 0.91))
    plt_hsuf(fig,path=out_full+"-sigeps_wl",**plt_scopt)
    
    fig, ax1 = plt.subplots()
    ax1.set_title('%s - Stress vs. strain curve, final part, with labels'%plt_name)
    ax1.set_xlabel('Strain / -')
    ax1.set_ylabel('Stress / MPa')
    ax1.plot(messu_FP['Strain'], messu_FP['Stress'], 'r--', label='con')
    if _opts['OPT_DIC']:
        ax1.plot(messu_FP[dic_used_Strain], messu_FP['Stress'], 'm--',label='opt')
    a,b = emec.fitting.stress_linfit_plt(
        messu_FP['Strain'], VIP_messu[['F3','F4']], YM_pref_con['E'], 0)
    ax1.plot(a, b, 'g-',label='$E_{con}$')
    tmp=VIP_messu[['S','F3','F4','Y','Y0','Y1','U']].sort_values()
    if 'B' in VIP_messu.index: tmp=tmp.append(VIP_messu[['B']])
    tmp=tmp.append(VIP_messu[['E']])
    tmp=tmp.sort_values()
    a, b=messu_FP.Strain[tmp],messu_FP.Stress[tmp]
    j=np.int64(-1)
    ax1.plot(a, b, 'bx')
    for x in tmp.index:
        j+=1
        if j%2: c=(6,-6)
        else:   c=(-6,6)
        ax1.annotate('%s' % x, xy=(a.iloc[j],b.iloc[j]), xycoords='data', 
                     xytext=c, ha="center", va="center", textcoords='offset points')
    if _opts['OPT_DIC']:
        tmp=VIP_dicu[['S','F3',*np.sort([*strain_osd.keys()]),'U']].sort_values()
        if 'B' in VIP_dicu.index: tmp=tmp.append(VIP_dicu[['B']])
        tmp=tmp.append(VIP_dicu[['E']])
        tmp=tmp.sort_values()
        a,b=messu_FP.loc[tmp,dic_used_Strain],messu_FP.Stress[tmp]
        j=np.int64(-1)
        ax1.plot(a, b, 'yx')
        for x in tmp.index:
            j+=1
            if j%2: c=(6,-6)
            else:   c=(-6,6)
            ax1.annotate('%s' % x, xy=(a.iloc[j],b.iloc[j]), xycoords='data', 
                         xytext=c, ha="center", va="center", textcoords='offset points')
        a,b = emec.fitting.stress_linfit_plt(
            messu_FP[dic_used_Strain], VIP_dicu[['F3','F4']], YM_pref_opt['E'], 0)
        ax1.plot(a, b, 'b-',label='$E_{opt}$')
    ax1.legend()
    plt_hsuf(fig,path=out_full+"-sigeps_fin",**plt_scopt)

    # =============================================================================
    #%%% 7.3 Generate outputs
    timings.loc[7.3]=time.perf_counter()
    log_custom("\n   Timing %f: %.5f s"%(timings.index[-1],
                                       timings.iloc[-1]-timings.iloc[0]),
                   **log_scopt)    
    
    
    t=E_lsq.loc[['E','Rquad']].T.stack()
    t.index=t.index.to_series().apply(lambda x: '{0}_{1}'.format(*x)).values
    t.name=prot_ser.name
    out_tab=pd.concat([out_tab,t])
    
    t=E_inc_F_comp.T.stack()
    t.index=t.index.to_series().apply(lambda x: '{0}_{1}'.format(*x)).values
    t.name=prot_ser.name
    t=t.add_prefix('E_inc_F_')
    out_tab=pd.concat([out_tab,t])

    t=E_inc_R_comp.T.stack()
    t.index=t.index.to_series().apply(lambda x: '{0}_{1}'.format(*x)).values
    t.name=prot_ser.name
    t=t.add_prefix('E_inc_R_')
    out_tab=pd.concat([out_tab,t])
    
    t = pd.concat([Check_to_D['AtoD_g'],Check_to_D['StoD_g'],
                   Check_to_D['MtoD_g']],axis=1).mean()
    t = t.add_prefix('Check_g_')
    t.name=prot_ser.name
    t2 = pd.concat([Check_to_D['AtoD_x'],Check_to_D['StoD_x'],
                    Check_to_D['MtoD_x']],axis=1).mean()
    t2 = t2.add_prefix('Check_l_')
    t2.name=prot_ser.name
    t = pd.concat([t,t2])
    out_tab=pd.concat([out_tab,t])
    
    out_tab=pd.DataFrame([out_tab])

    vnew=pd.Series(VIP_messu.drop_duplicates().index.values,
                   index=VIP_messu.drop_duplicates(),dtype='O')
    VIP_messu=VIP_messu.sort_values()
    for i in VIP_messu.index:
        if VIP_messu.duplicated().loc[i]:
            vnew.loc[VIP_messu.loc[i]]=vnew.loc[VIP_messu.loc[i]]+','+i
    messu['VIP_m']=vnew
    messu.VIP_m.fillna('', inplace=True)
    
    vnew=pd.Series(VIP_dicu.drop_duplicates().index.values,
                   index=VIP_dicu.drop_duplicates(),dtype='O')
    VIP_dicu=VIP_dicu.sort_values()
    for i in VIP_dicu.index:
        if VIP_dicu.duplicated().loc[i]:
            vnew.loc[VIP_dicu.loc[i]]=vnew.loc[VIP_dicu.loc[i]]+','+i
    messu['VIP_d']=vnew
    messu.VIP_d.fillna('', inplace=True)
    
    messu.to_csv(out_full+'.csv',sep=';')   
    out_tab.to_csv(out_full+'-Mat.csv',sep=';')
    
    HDFst=pd.HDFStore(out_full+'.h5')
    HDFst['Measurement'] = messu
    HDFst['Measurement_FP'] = messu_FP
    HDFst['Material_Parameters'] = out_tab
    HDFst['Geometrie_functions'] = pd.Series({
        'func_t': func_t,'func_w': func_w, 
        'func_A': func_A, 'func_I': func_I
        })
    HDFst['Timings'] = timings
    HDFst['Options'] = _opts
    
    # HDFst['Bending_Legion'] = bl
    HDFst['Opt_Geo_Fit'] = geo_fit
    HDFst['Opt_Pre_Fit'] = Pre_fit_df
    HDFst['Opt_Bend_Fit'] = Bend_fit_df
    HDFst['Inc_Pre_Fit'] = Pre_inc_fit_df
    HDFst['Inc_Bend_Fit'] = Bend_inc_fit_df
    
    HDFst['Points_transformed'] = Points_T_stacked
    HDFst['Points_lot_transformed'] = Points_L_T_stacked
    HDFst['Points_A_disp'] = P_xcoord_ydisp
    HDFst['Points_S_disp'] = P_xcoord_ydisp_meas_S
    HDFst['Points_M_disp'] = P_xcoord_ydisp_meas_M
    HDFst['Points_A_diff'] = P_xcoord_ydiff
    HDFst['Points_S_diff'] = P_xcoord_ydiff_meas_S
    HDFst['Points_M_diff'] = P_xcoord_ydiff_meas_M
    
    HDFst['VIP'] = pd.concat([VIP_messu,VIP_dicu],axis=1)
    
    HDFst['Exact_VIP_con']=yield_df_con
    HDFst['Exact_VIP_opt']=yield_df_opt
    
    HDFst['DQcon'] = DQcon
    HDFst['DQopt'] = DQopt
    
    HDFst['E_lsq'] = E_lsq
    HDFst['E_inc_df'] = E_Methods_df
    HDFst['E_inc'] = pd.concat([E_inc_F_comp.add_prefix('E_inc_F_'),
                                E_inc_R_comp.add_prefix('E_inc_R_')],axis=1)
    HDFst['Check_to_D'] = Check_to_D
    
    HDFst.close()
    
    timings.loc[10.0]=time.perf_counter()
    if log_scopt['output_lvl']>=1:
        log_scopt['logfp'].close()
        log_scopt['logfp'] = None # workaround (Error _io.TextIOWrapper while series run)
    
    return timings, cout
```

---

© Copyright 2024, MarcGebhardt.

Built with Sphinx using a
theme
provided by Read the Docs.
