# Supplementary material for: Approach to Standardized Material Characterization of the Human Lumbopelvic System: Testing and Evaluation
Source: Bioengineering (Basel). 2025 Aug 11;12(8):862. doi: 10.3390/bioengineering12080862 (PMC12383908; doi:10.3390/bioengineering12080862)
Supplement: Supplementary file 1 [file bioengineering-12-00862-s001.zip › File S3 Evaluation code/ExMechEva-0.1.2/docs/_build/html/_modules/index.html]

Overview: module code — ExMechEva v0.1.2 documentation


ExMechEva

Contents:

- ExMechEva

ExMechEva

- Overview: module code

---

# All modules for which code is available

- exmecheva.Eva\_ACT
- exmecheva.Eva\_ATT
- exmecheva.Eva\_TBT
- exmecheva.bending.bfunc\_class
- exmecheva.bending.bfunc\_com
- exmecheva.bending.bfunc\_fse
- exmecheva.bending.evaluation
- exmecheva.bending.fitting
- exmecheva.bending.opt\_mps
- exmecheva.bending.plotting
- exmecheva.common.analyze
- exmecheva.common.eva\_opt\_hand
- exmecheva.common.fitting
- exmecheva.common.helper
- exmecheva.common.list\_ops
- exmecheva.common.loadnsave
- exmecheva.common.mc\_char
- exmecheva.common.mc\_man
- exmecheva.common.mc\_yield
- exmecheva.common.output
- exmecheva.common.pd\_ext
- exmecheva.common.plotting
- exmecheva.common.stat\_ext
- exmecheva.eva

---

© Copyright 2024, MarcGebhardt.

Built with Sphinx using a
theme
provided by Read the Docs.
