# Supplementary figures and images for: Approach to Standardized Material Characterization of the Human Lumbopelvic System: Testing and Evaluation
Source: Bioengineering (Basel). 2025 Aug 11;12(8):862. doi: 10.3390/bioengineering12080862 (PMC12383908; doi:10.3390/bioengineering12080862)

tl21x - Analyzing meas. force

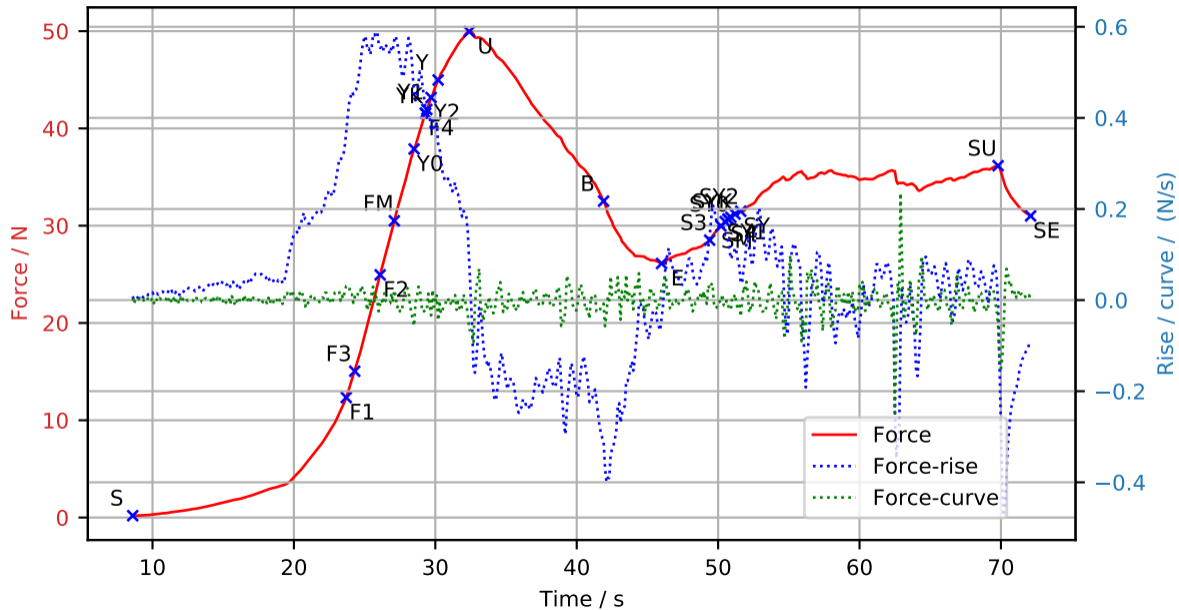

Supplement: Supplementary file 1 [file bioengineering-12-00862-s001.zip › File S3 Evaluation code/ExMechEva-0.1.2/data/Test/ACT/Series_Test/eva/tl21x-Fdricu.pdf]

tl21x - Measuring

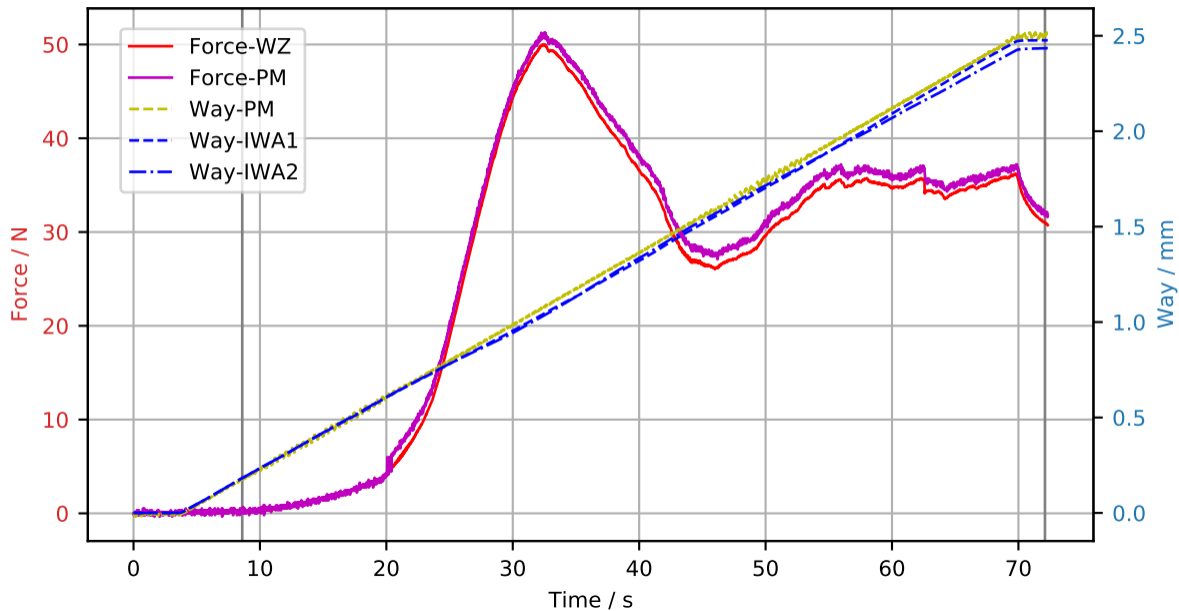

Supplement: Supplementary file 1 [file bioengineering-12-00862-s001.zip › File S3 Evaluation code/ExMechEva-0.1.2/data/Test/ACT/Series_Test/eva/tl21x-meas.pdf]

tl21x - Measuring (used)

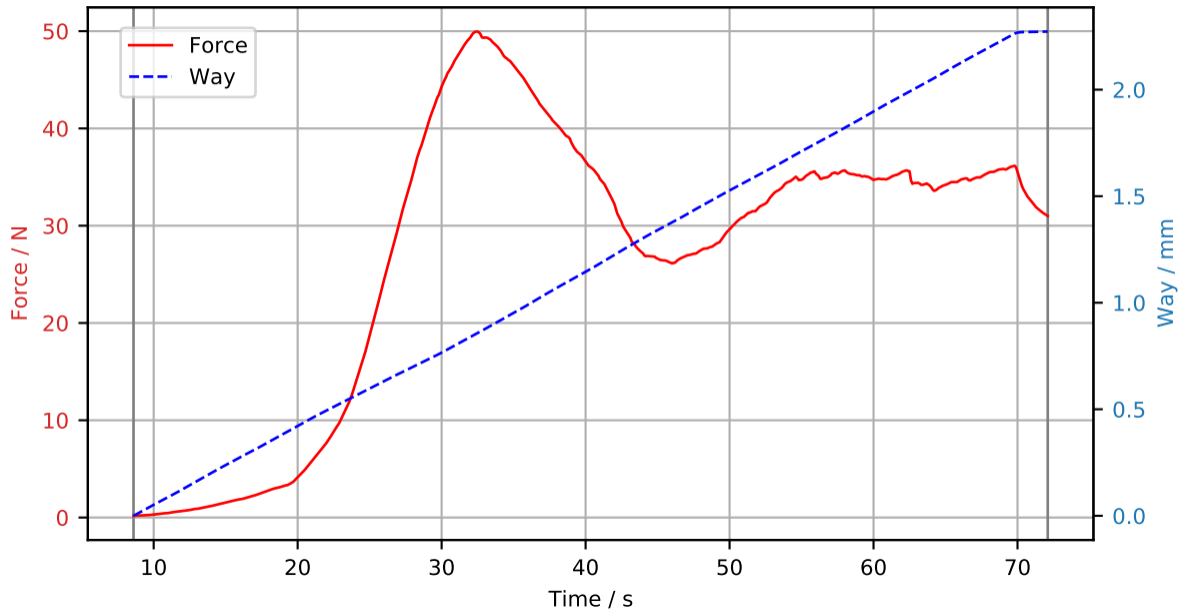

Supplement: Supplementary file 1 [file bioengineering-12-00862-s001.zip › File S3 Evaluation code/ExMechEva-0.1.2/data/Test/ACT/Series_Test/eva/tl21x-meas_u.pdf]

tl21x - Stress vs. strain curve, final part, with labels

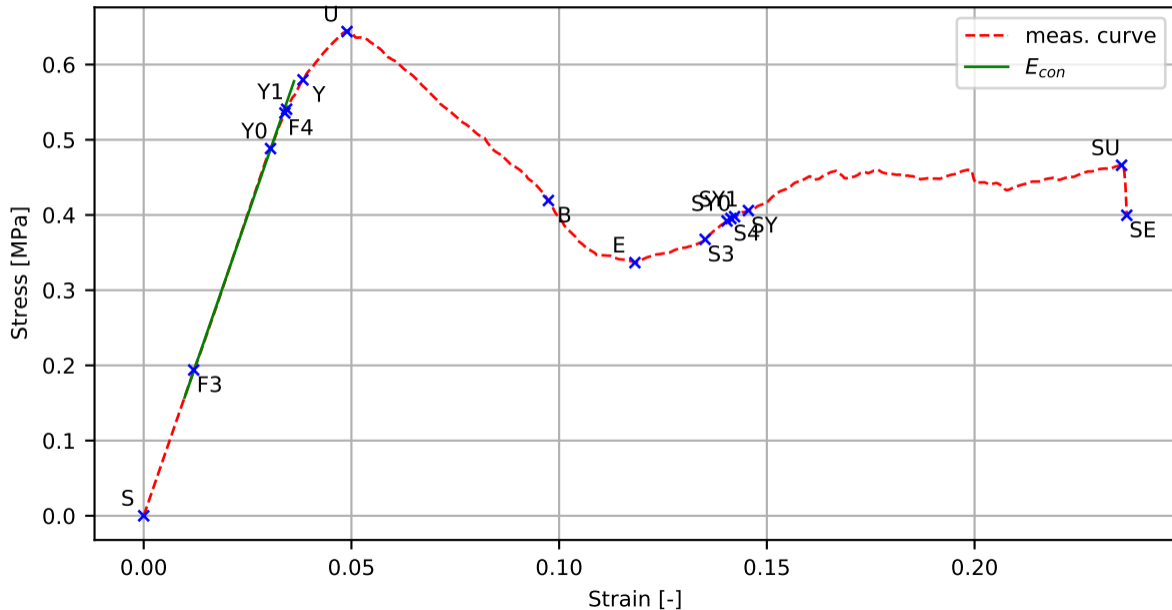

Supplement: Supplementary file 1 [file bioengineering-12-00862-s001.zip › File S3 Evaluation code/ExMechEva-0.1.2/data/Test/ACT/Series_Test/eva/tl21x-sigeps_fin.pdf]

tl21x - Stress vs. strain curve with labels

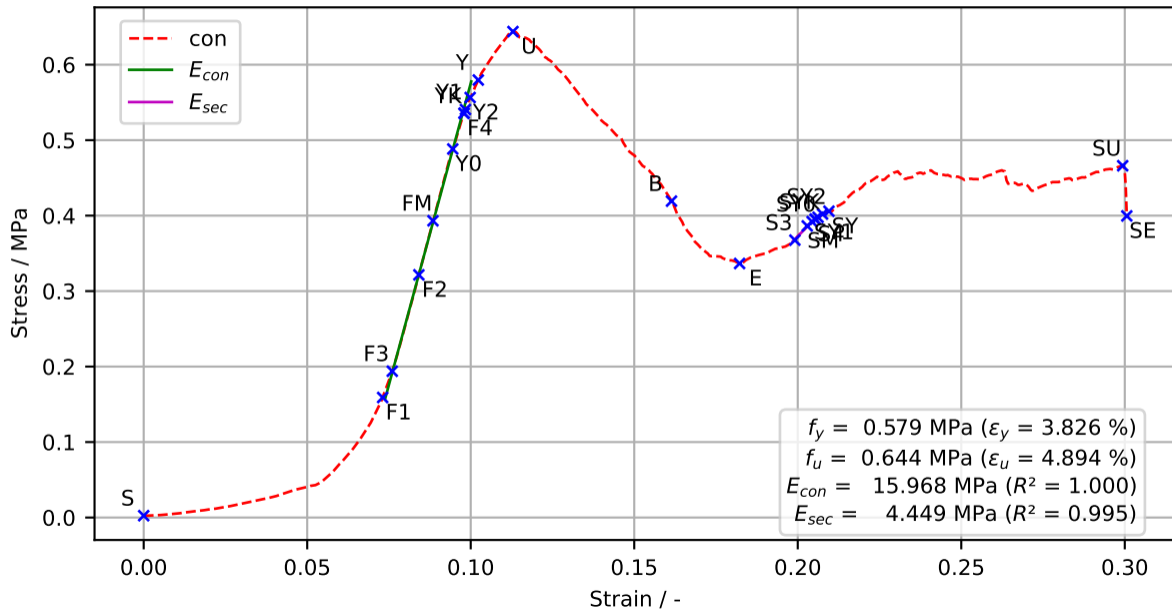

Supplement: Supplementary file 1 [file bioengineering-12-00862-s001.zip › File S3 Evaluation code/ExMechEva-0.1.2/data/Test/ACT/Series_Test/eva/tl21x-sigeps_wl.pdf]

tl21x - Stress vs. strain curve with labels (to 1st min.)

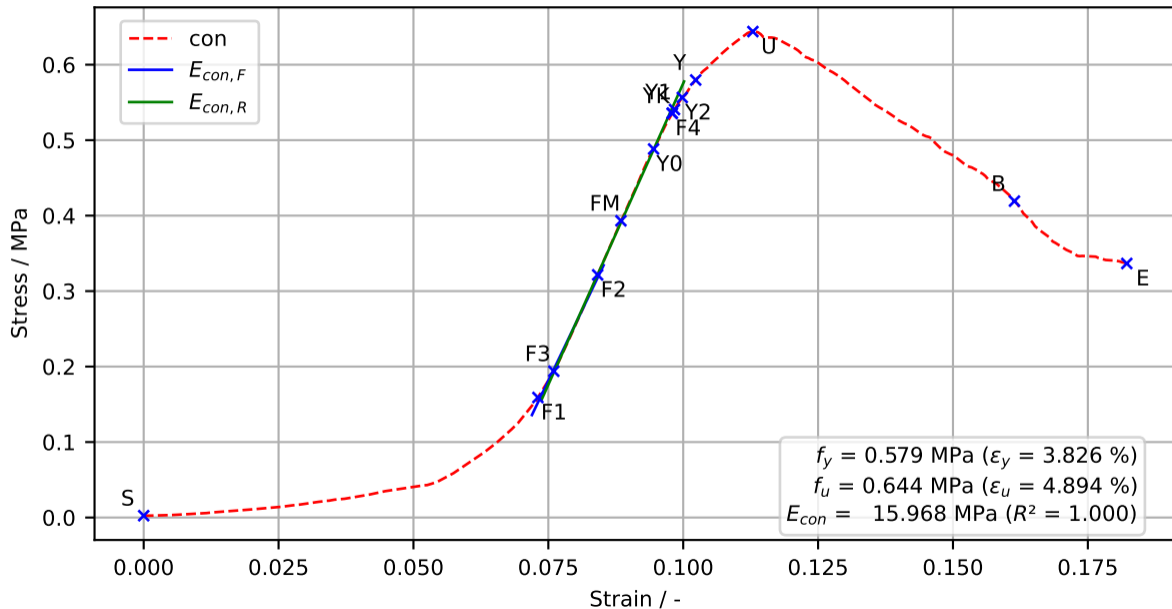

Supplement: Supplementary file 1 [file bioengineering-12-00862-s001.zip › File S3 Evaluation code/ExMechEva-0.1.2/data/Test/ACT/Series_Test/eva/tl21x-sigeps_wl1m.pdf]

# tl21x - Compare method A

All Steps

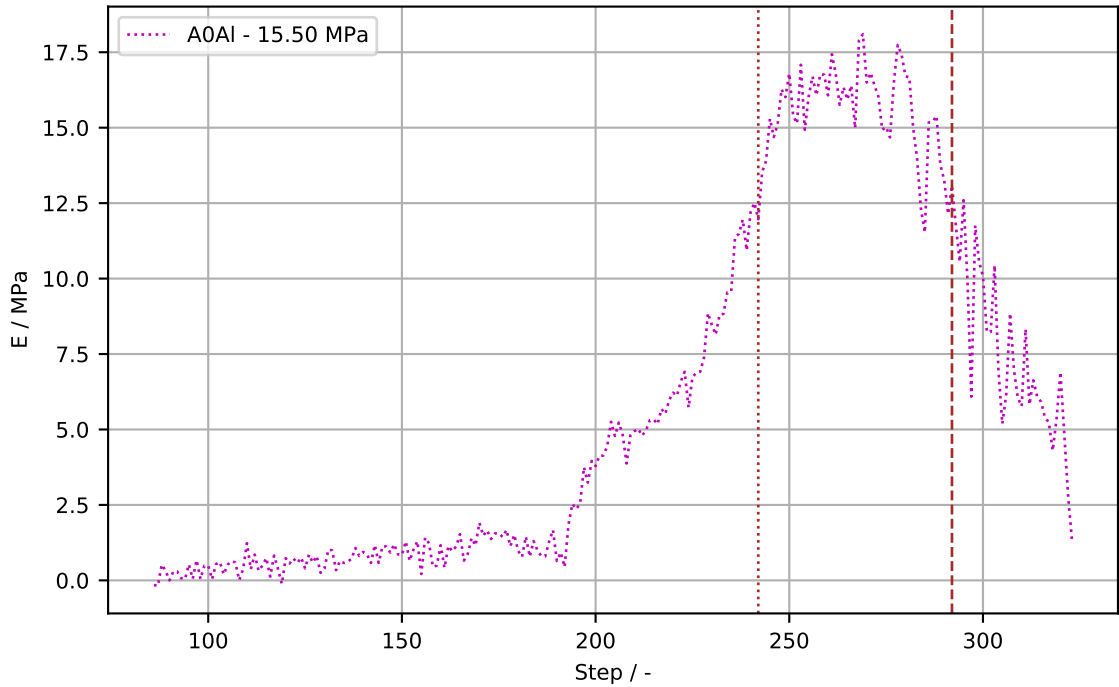

Improved determination range

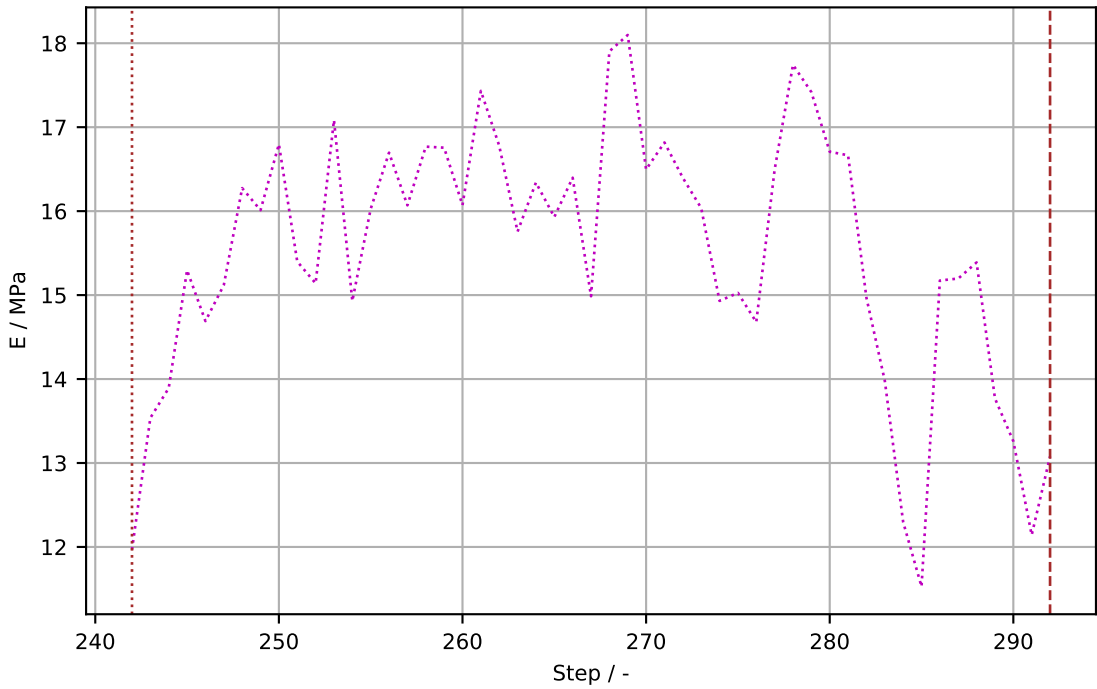

Supplement: Supplementary file 1 [file bioengineering-12-00862-s001.zip › File S3 Evaluation code/ExMechEva-0.1.2/data/Test/ACT/Series_Test/eva/tl21x-YM-Me_A.pdf]

sr03a - Youngs-modulus and res. strain vs. cycles

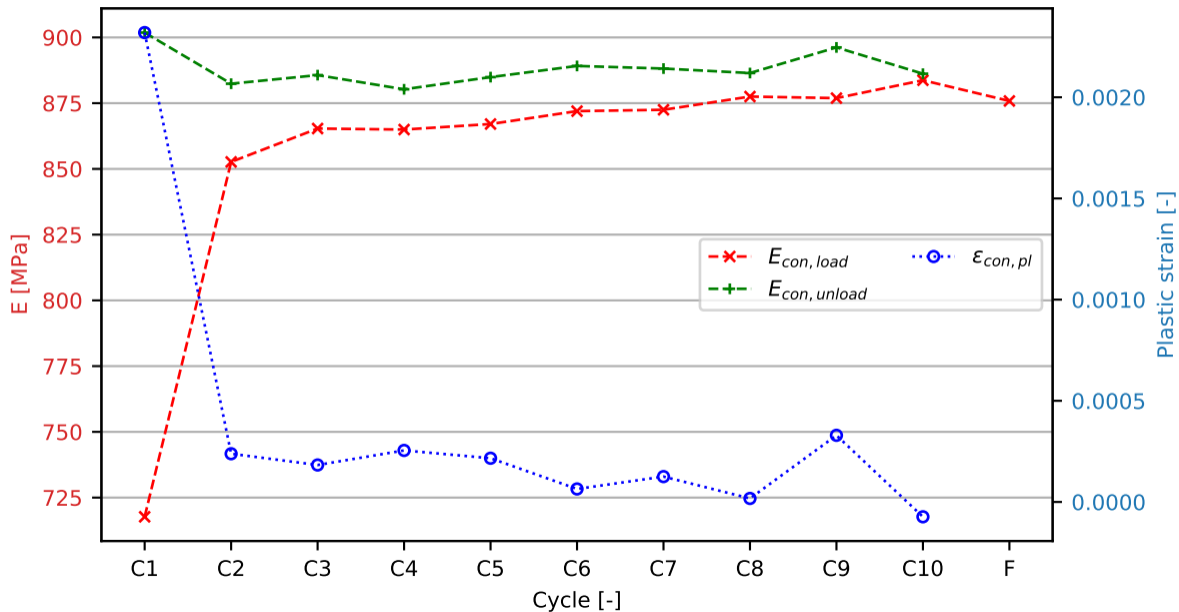

Supplement: Supplementary file 1 [file bioengineering-12-00862-s001.zip › File S3 Evaluation code/ExMechEva-0.1.2/data/Test/ATT/Series_Test/eva/sr03a-Eepszyk.pdf]

sr03a - Analyzing meas. force

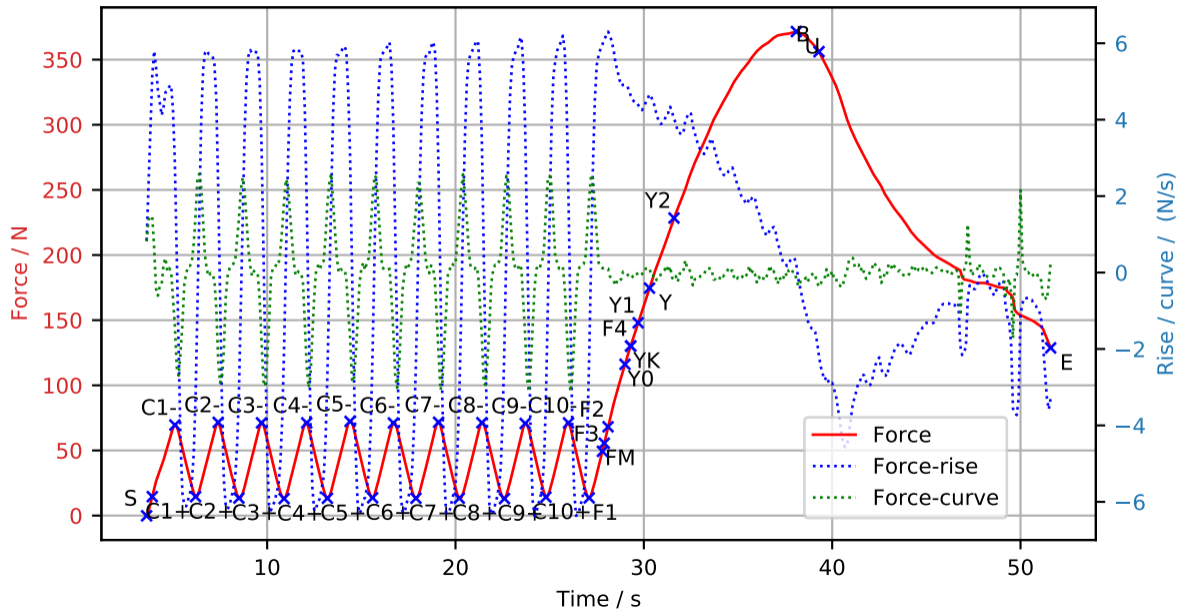

Supplement: Supplementary file 1 [file bioengineering-12-00862-s001.zip › File S3 Evaluation code/ExMechEva-0.1.2/data/Test/ATT/Series_Test/eva/sr03a-Fdricu.pdf]

sr03a - Measuring

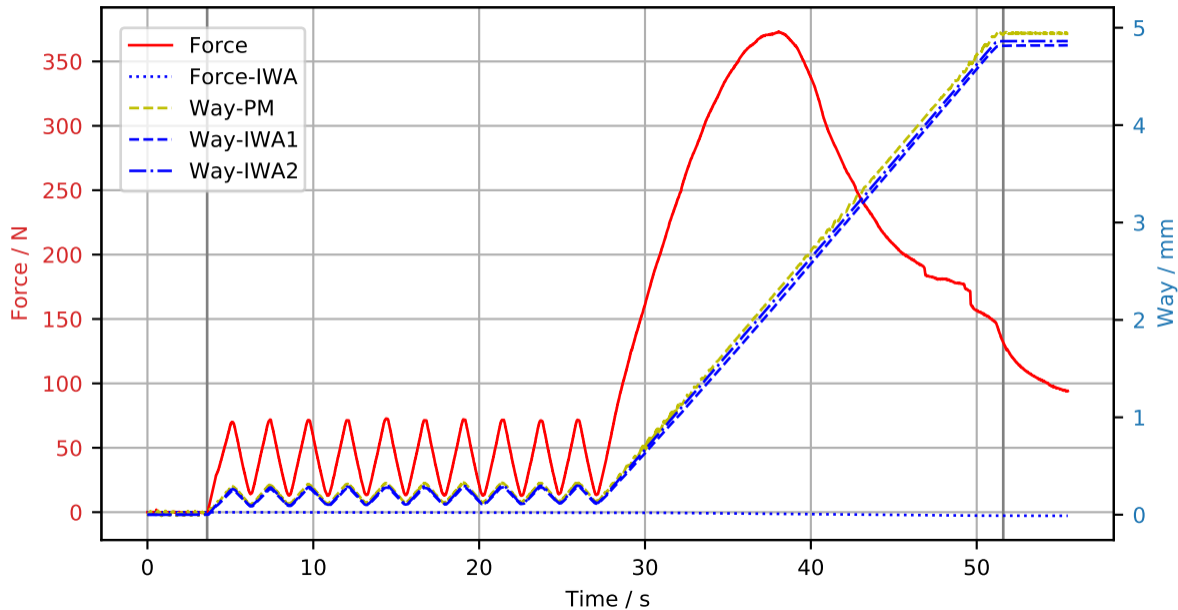

Supplement: Supplementary file 1 [file bioengineering-12-00862-s001.zip › File S3 Evaluation code/ExMechEva-0.1.2/data/Test/ATT/Series_Test/eva/sr03a-meas.pdf]

sr03a - Measuring (used)

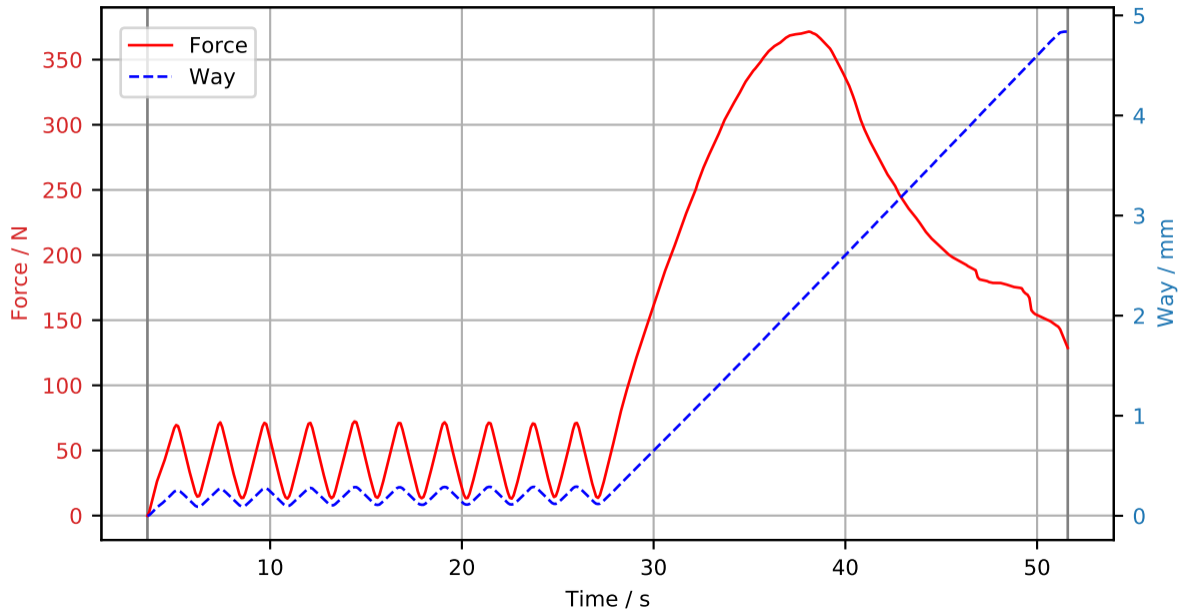

Supplement: Supplementary file 1 [file bioengineering-12-00862-s001.zip › File S3 Evaluation code/ExMechEva-0.1.2/data/Test/ATT/Series_Test/eva/sr03a-meas_u.pdf]

sr03a - Stress vs. strain curve, final part, with labels

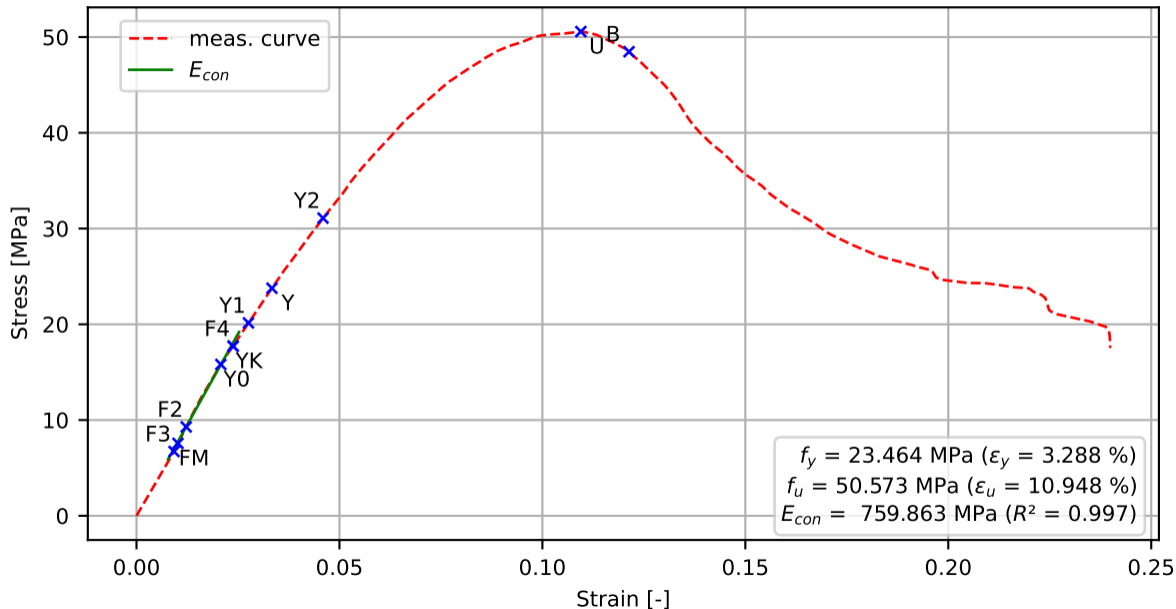

Supplement: Supplementary file 1 [file bioengineering-12-00862-s001.zip › File S3 Evaluation code/ExMechEva-0.1.2/data/Test/ATT/Series_Test/eva/sr03a-sigeps_fin.pdf]

sr03a - Stress vs. strain curve with labels

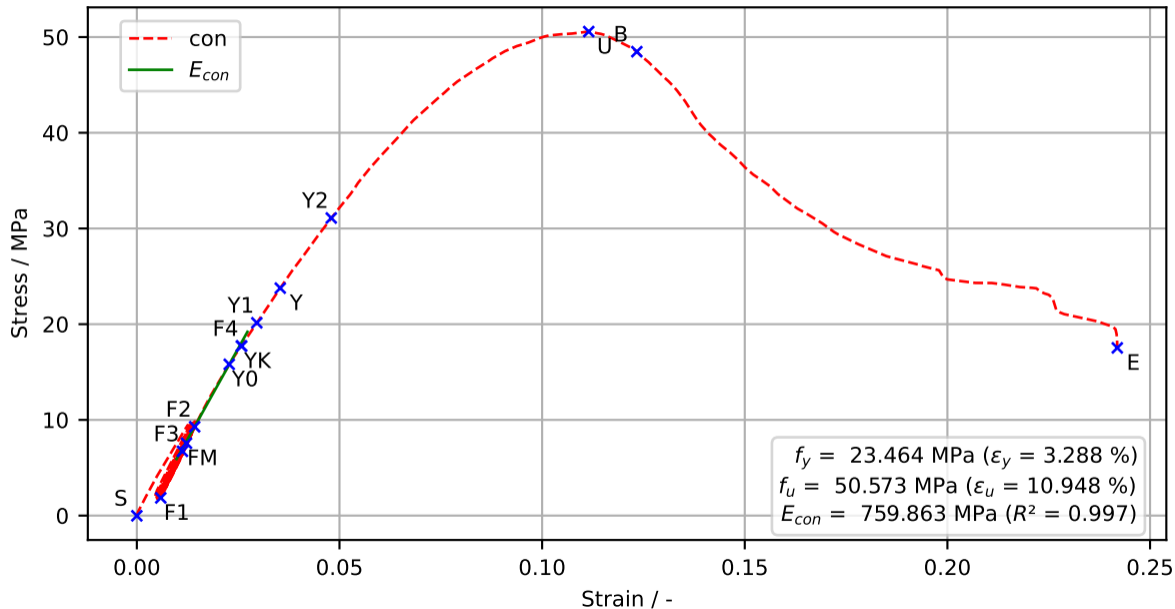

Supplement: Supplementary file 1 [file bioengineering-12-00862-s001.zip › File S3 Evaluation code/ExMechEva-0.1.2/data/Test/ATT/Series_Test/eva/sr03a-sigeps_wl.pdf]

sr03a - Stress vs. strain curve - yield point determination

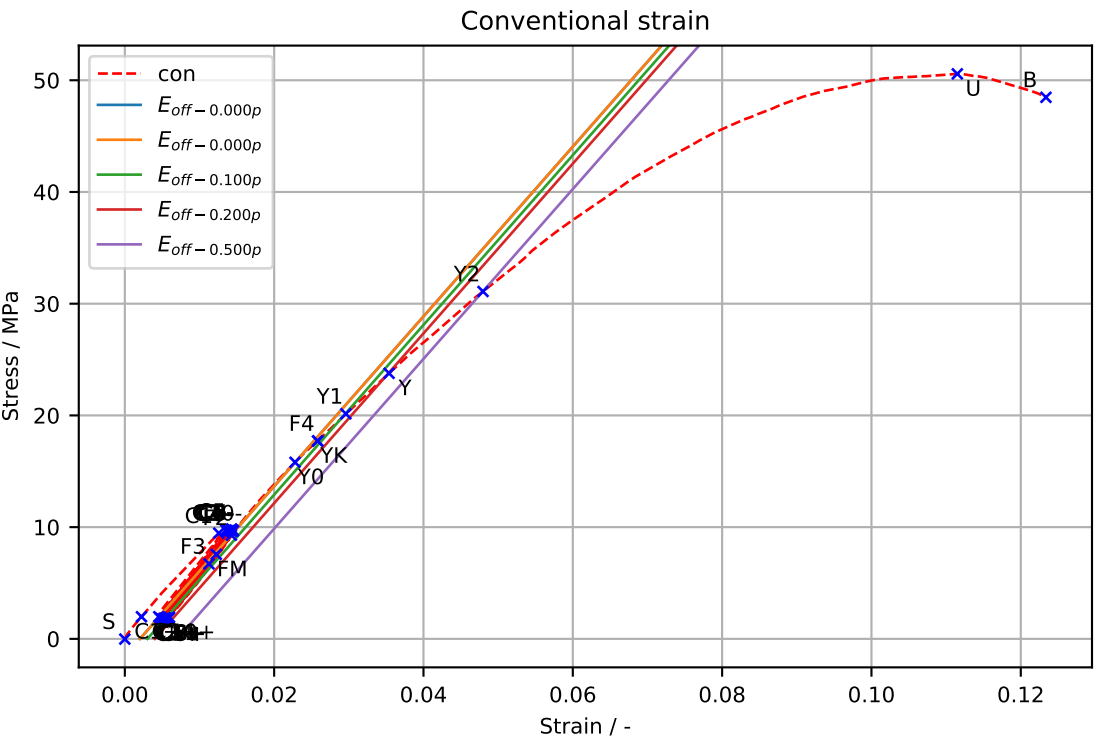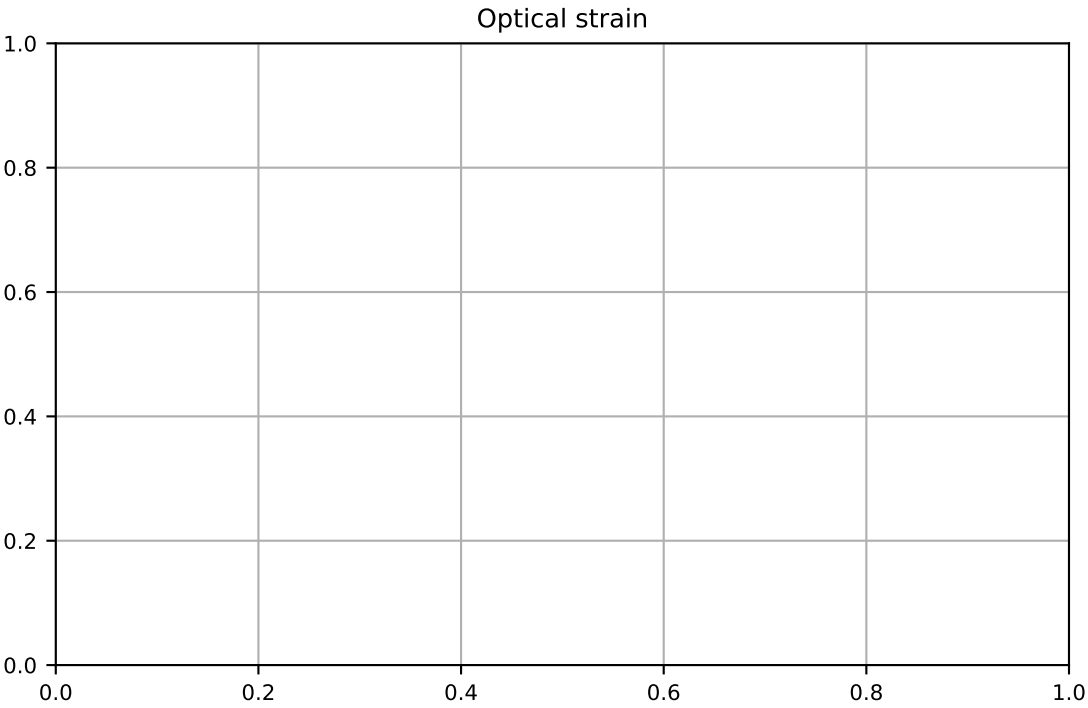

Supplement: Supplementary file 1 [file bioengineering-12-00862-s001.zip › File S3 Evaluation code/ExMechEva-0.1.2/data/Test/ATT/Series_Test/eva/sr03a-sigeps_yielddet.pdf]

# sr03a - Compare method A

All Steps

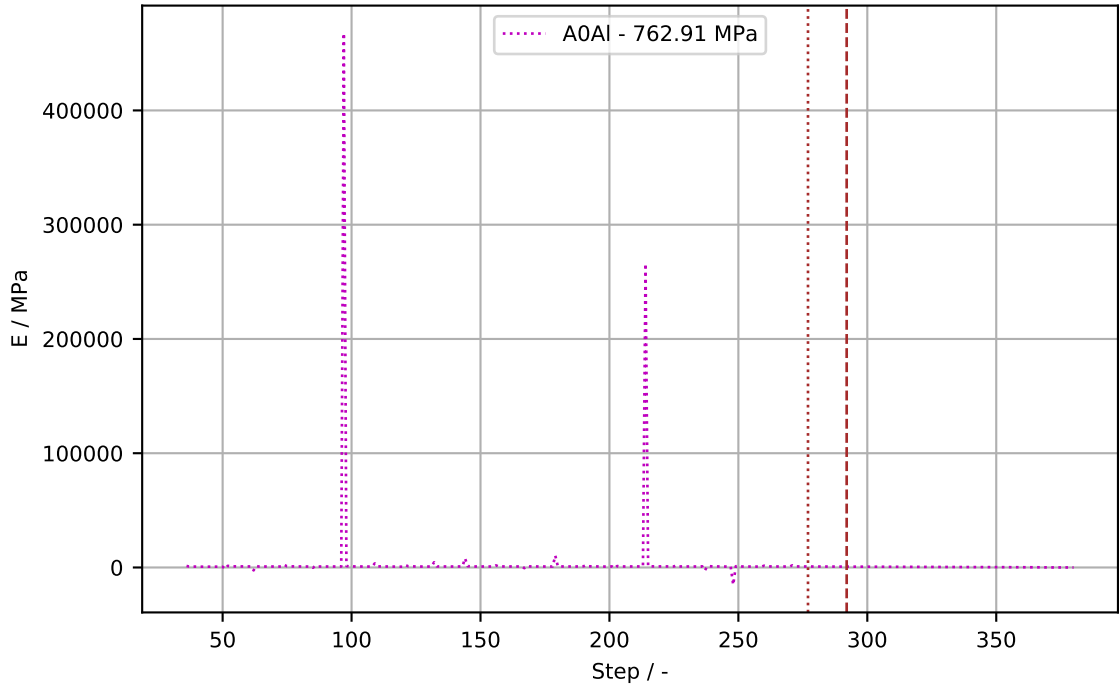

Improved determination range

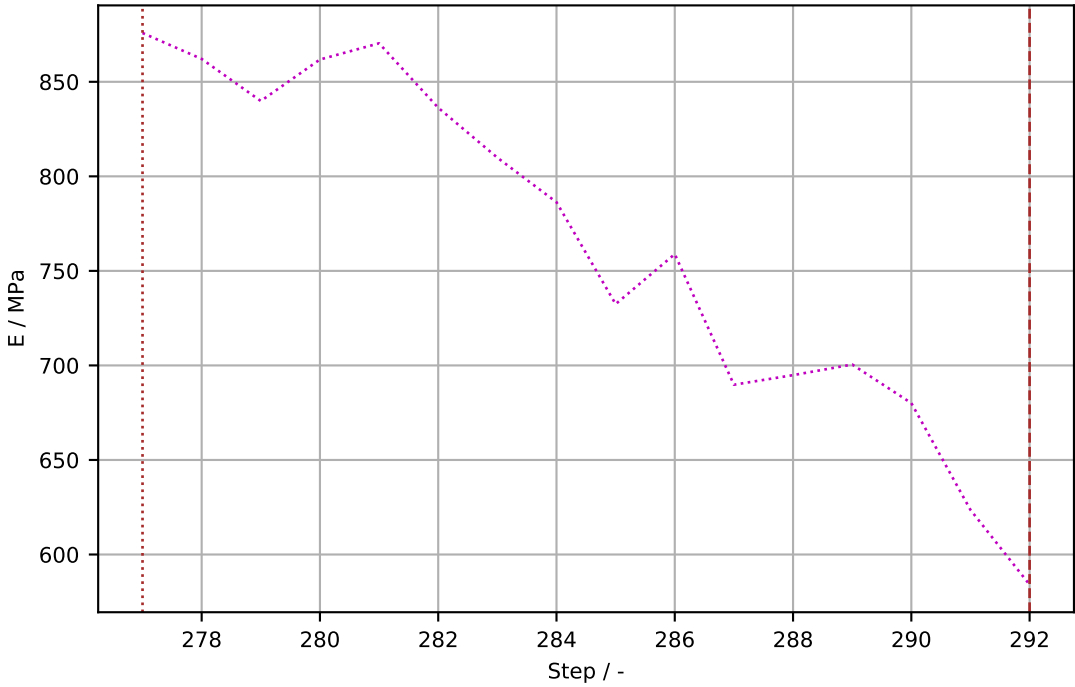

Supplement: Supplementary file 1 [file bioengineering-12-00862-s001.zip › File S3 Evaluation code/ExMechEva-0.1.2/data/Test/ATT/Series_Test/eva/sr03a-YM-Me_A.pdf]

# sr03a - Improvement of evaluation range for Youngs Modulus

Conventional measured strain

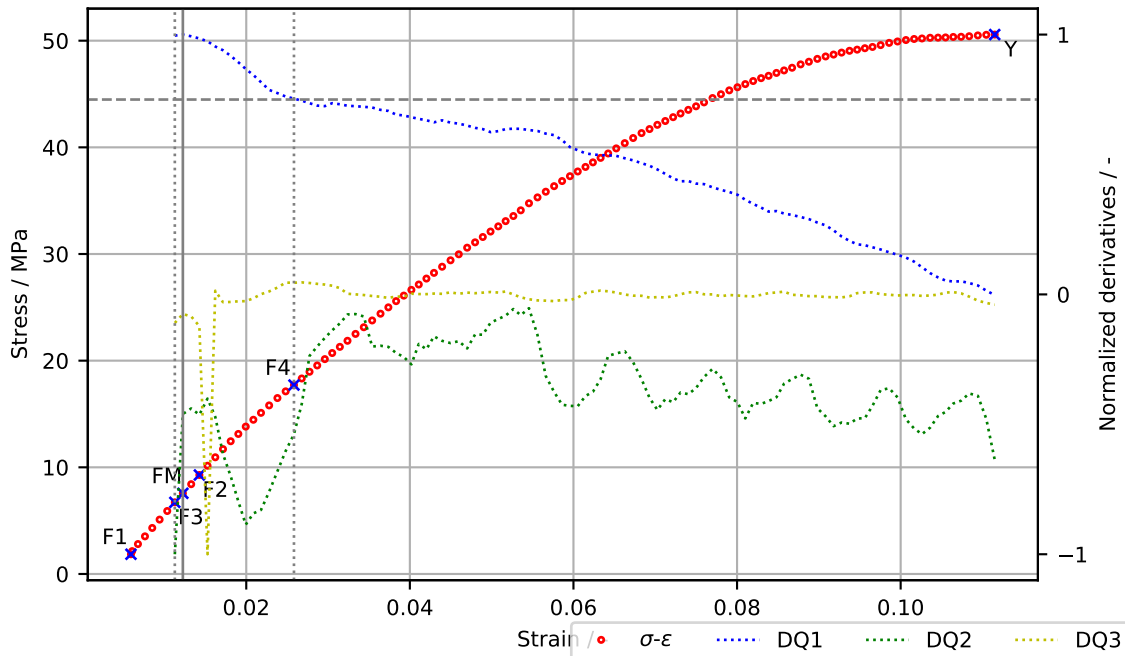

Supplement: Supplementary file 1 [file bioengineering-12-00862-s001.zip › File S3 Evaluation code/ExMechEva-0.1.2/data/Test/ATT/Series_Test/eva/sr03a-YMRange_Imp.pdf]

cl12a - Fit-full - evaluation range  
Displacement

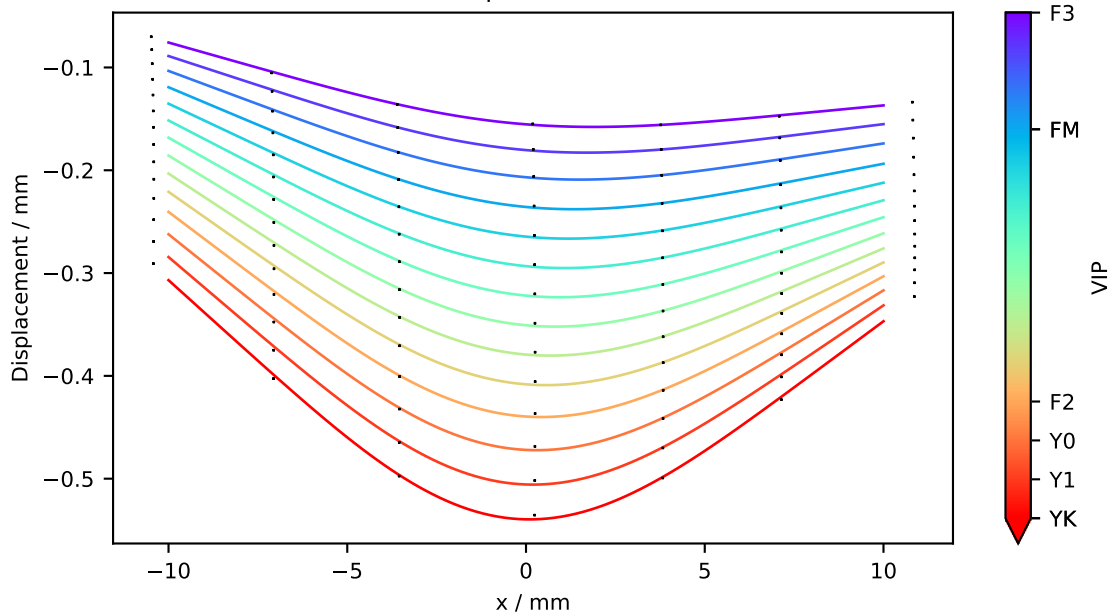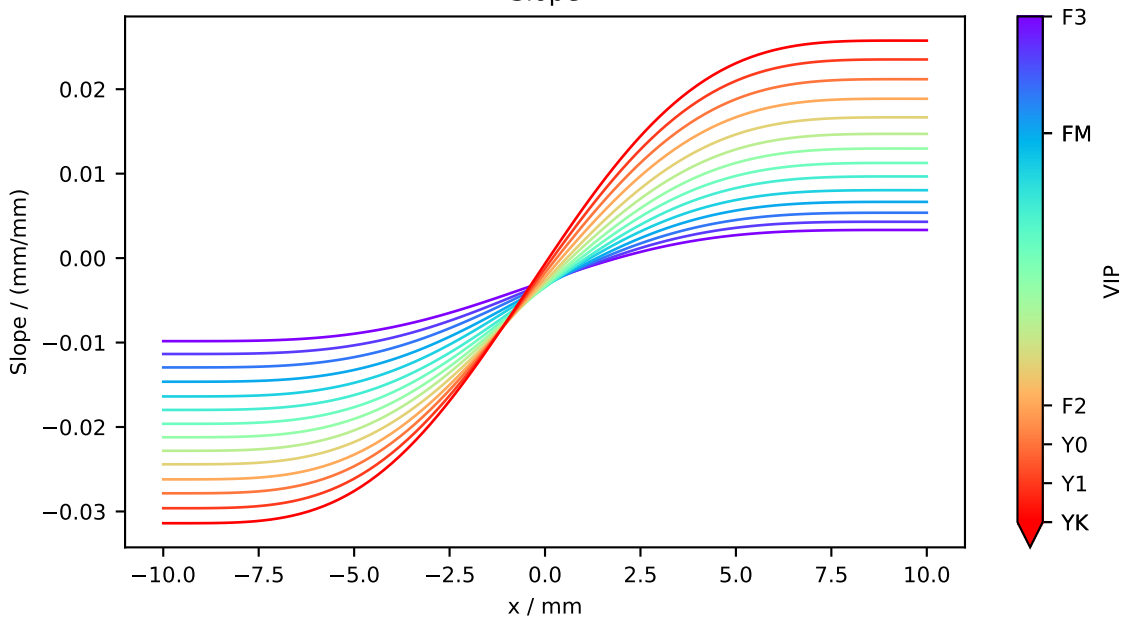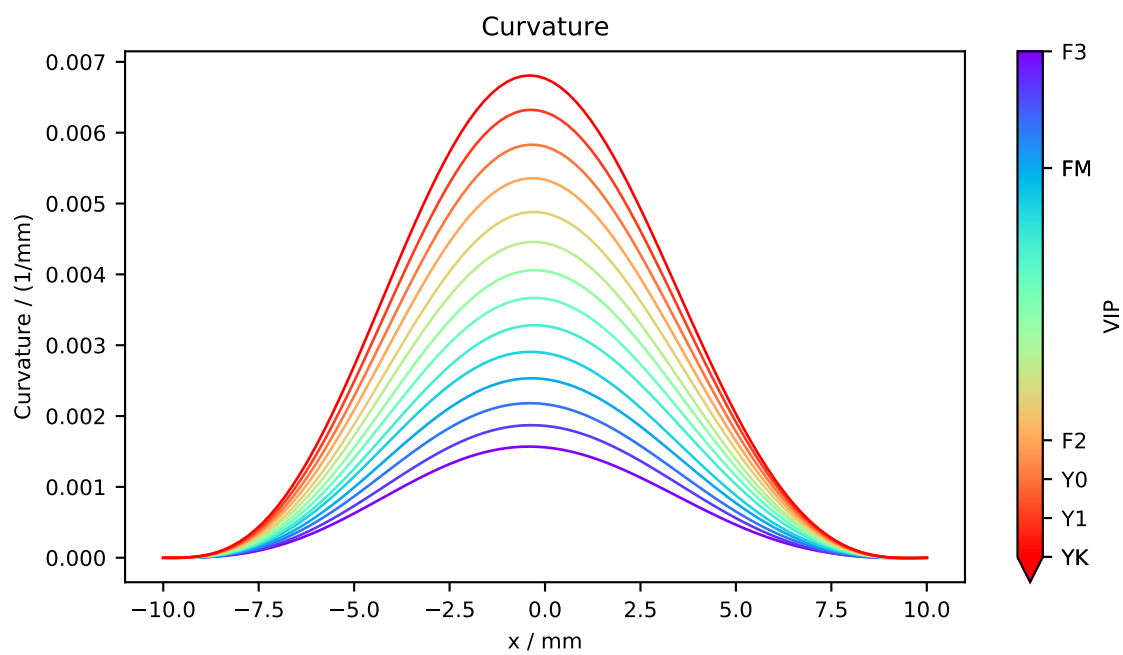

Supplement: Supplementary file 1 [file bioengineering-12-00862-s001.zip › File S3 Evaluation code/ExMechEva-0.1.2/data/Test/TBT/Series_Test/eva/cl12a-DIC_fit-A-eva.pdf]

cl12a - Fit-full  
Displacement

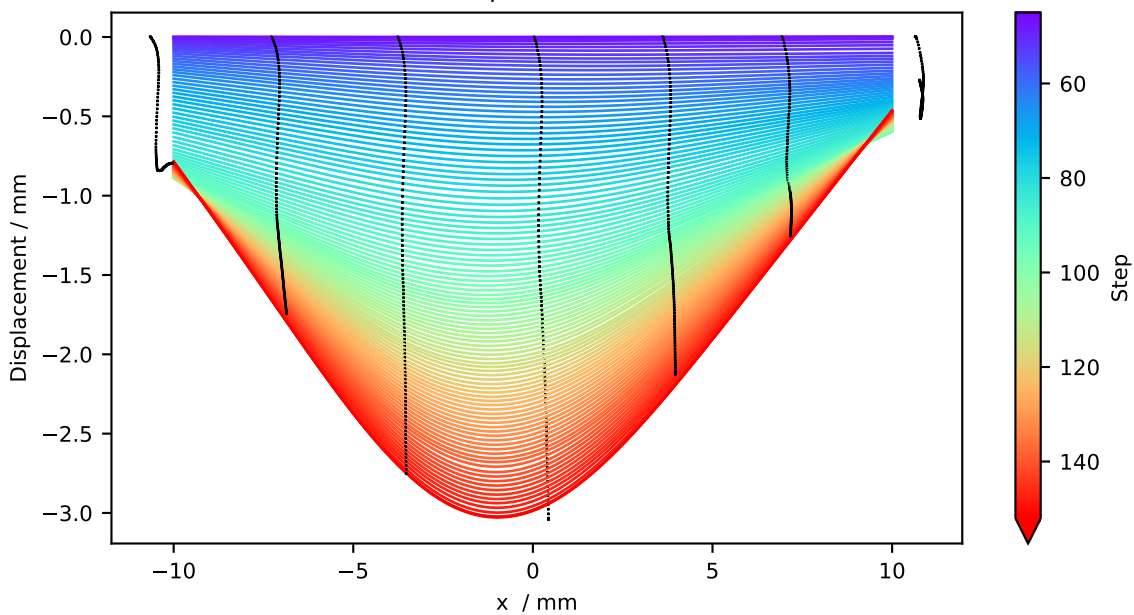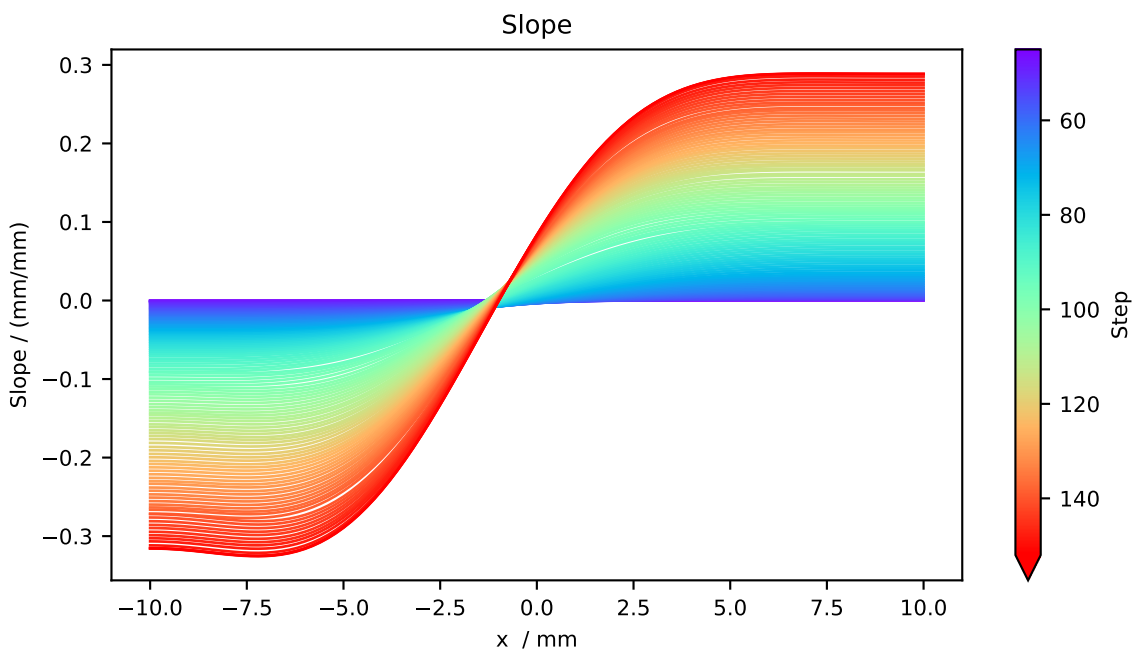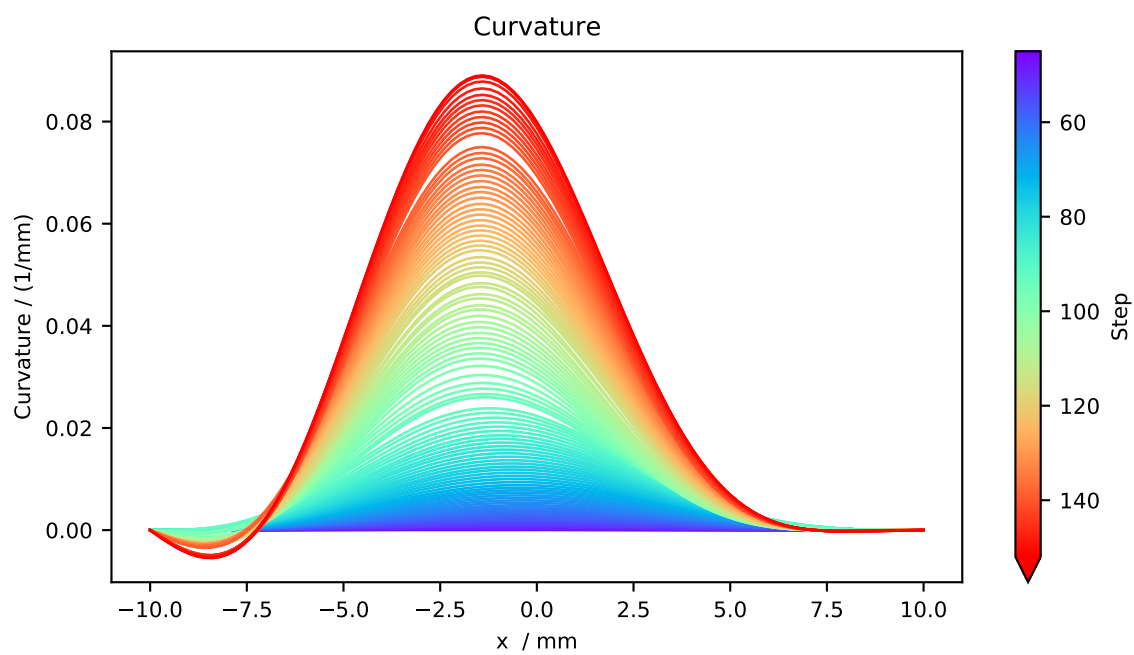

Supplement: Supplementary file 1 [file bioengineering-12-00862-s001.zip › File S3 Evaluation code/ExMechEva-0.1.2/data/Test/TBT/Series_Test/eva/cl12a-DIC_fit-A.pdf]

cl12a - Fit-compare - Displacement for step 92

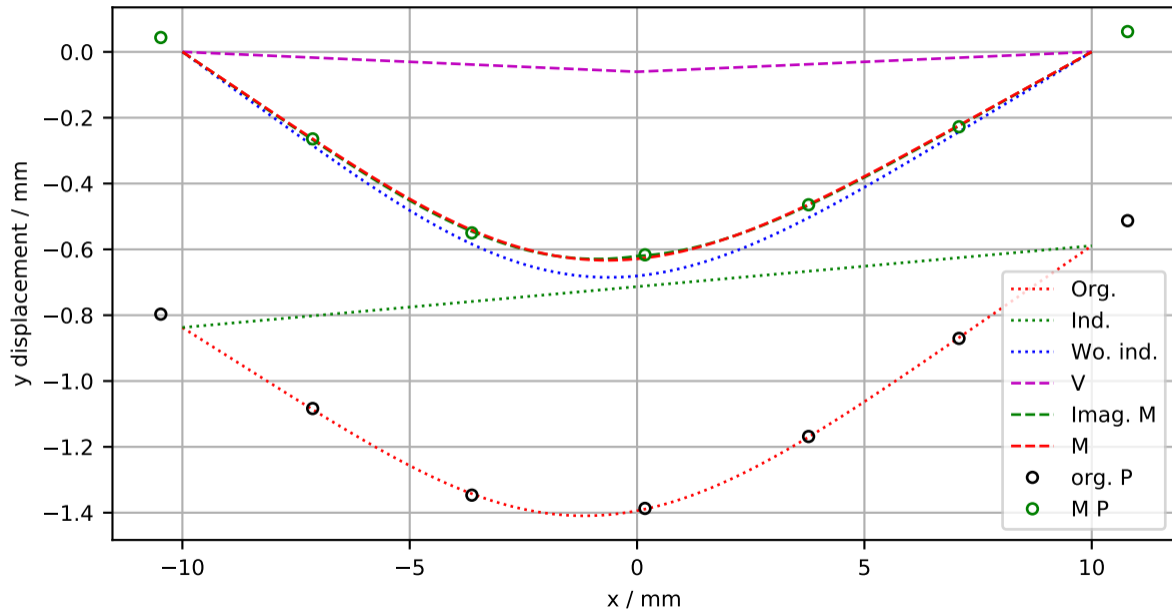

Supplement: Supplementary file 1 [file bioengineering-12-00862-s001.zip › File S3 Evaluation code/ExMechEva-0.1.2/data/Test/TBT/Series_Test/eva/cl12a-DIC_fit-bl_U-d0.pdf]

cl12a - Fit-compare - Slope for step 92

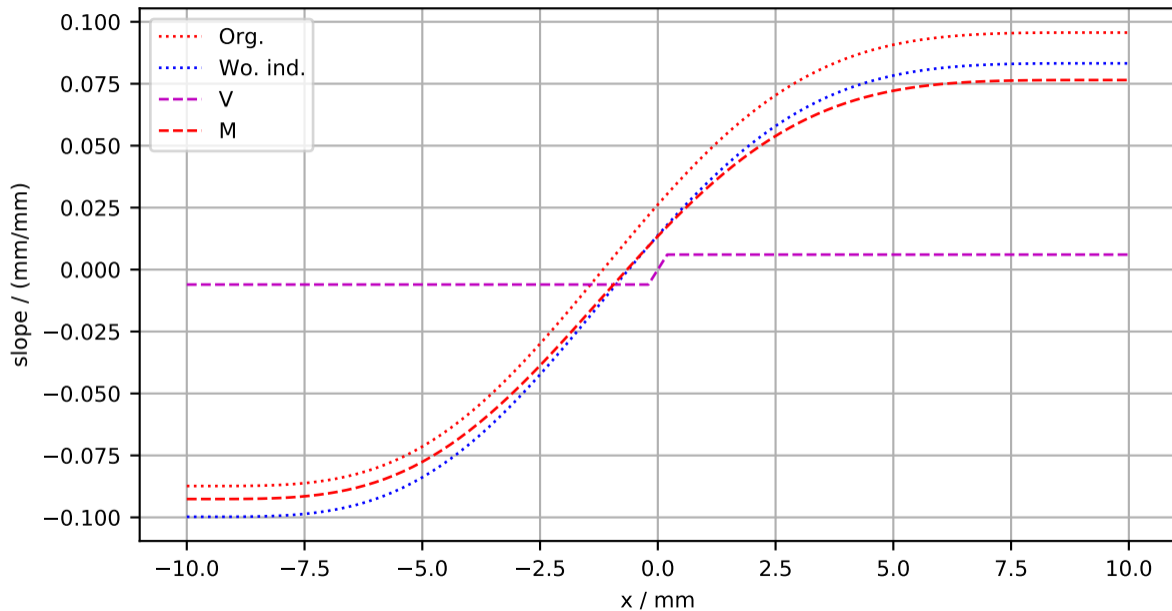

Supplement: Supplementary file 1 [file bioengineering-12-00862-s001.zip › File S3 Evaluation code/ExMechEva-0.1.2/data/Test/TBT/Series_Test/eva/cl12a-DIC_fit-bl_U-d1.pdf]

cl12a - Fit-compare - Curvature for step 92

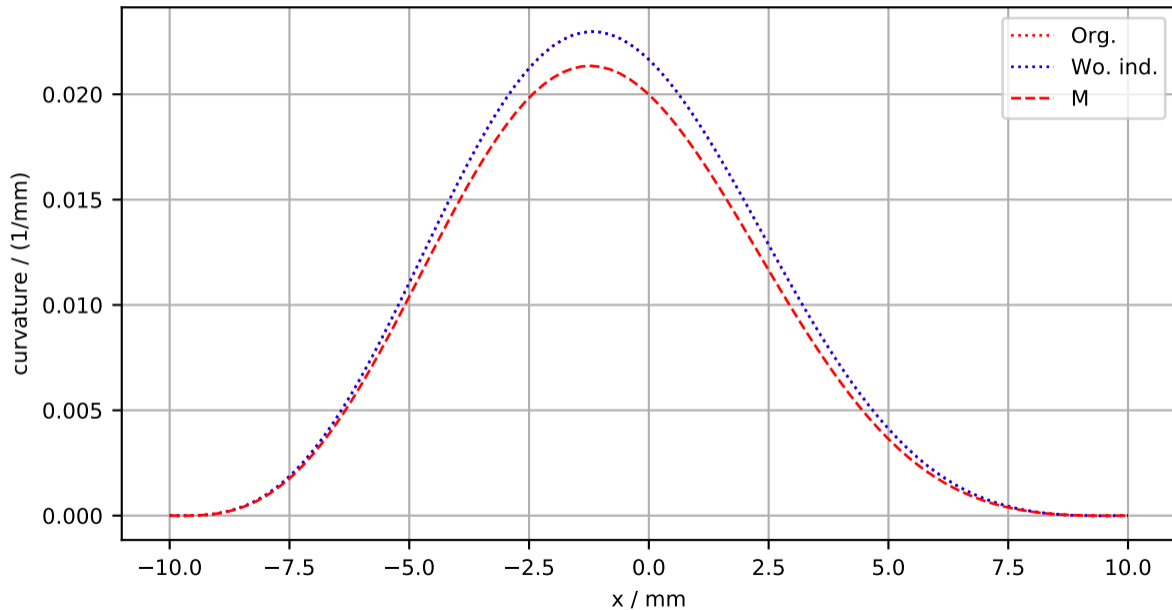

Supplement: Supplementary file 1 [file bioengineering-12-00862-s001.zip › File S3 Evaluation code/ExMechEva-0.1.2/data/Test/TBT/Series_Test/eva/cl12a-DIC_fit-bl_U-d2.pdf]

cl12a - Fit-Bending - evaluation range  
Displacement

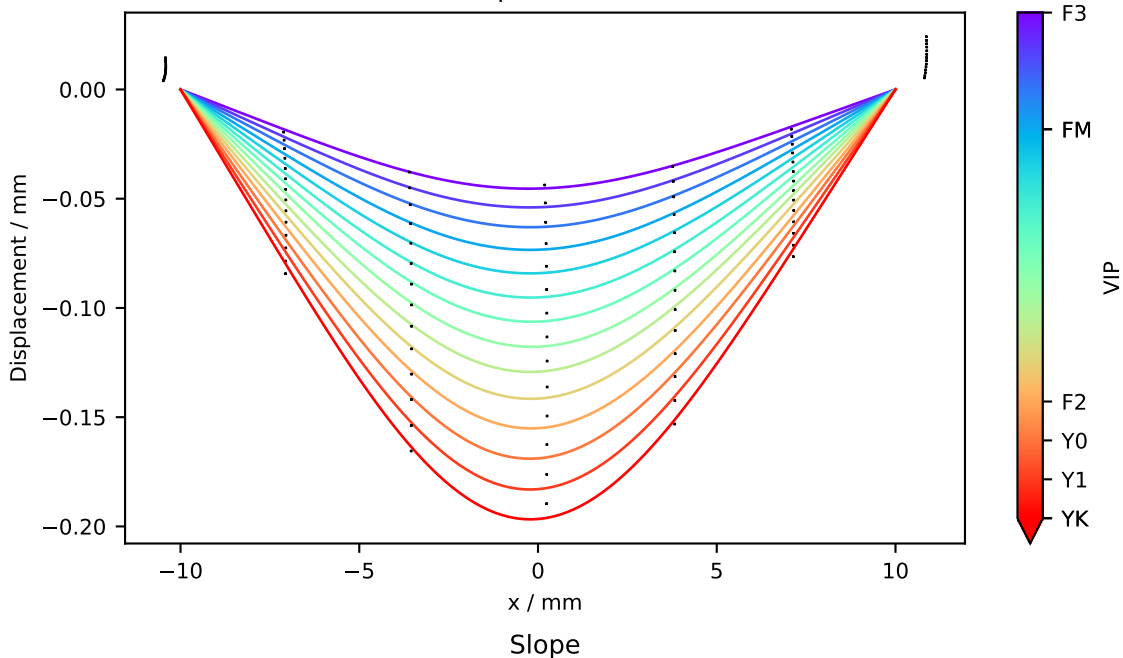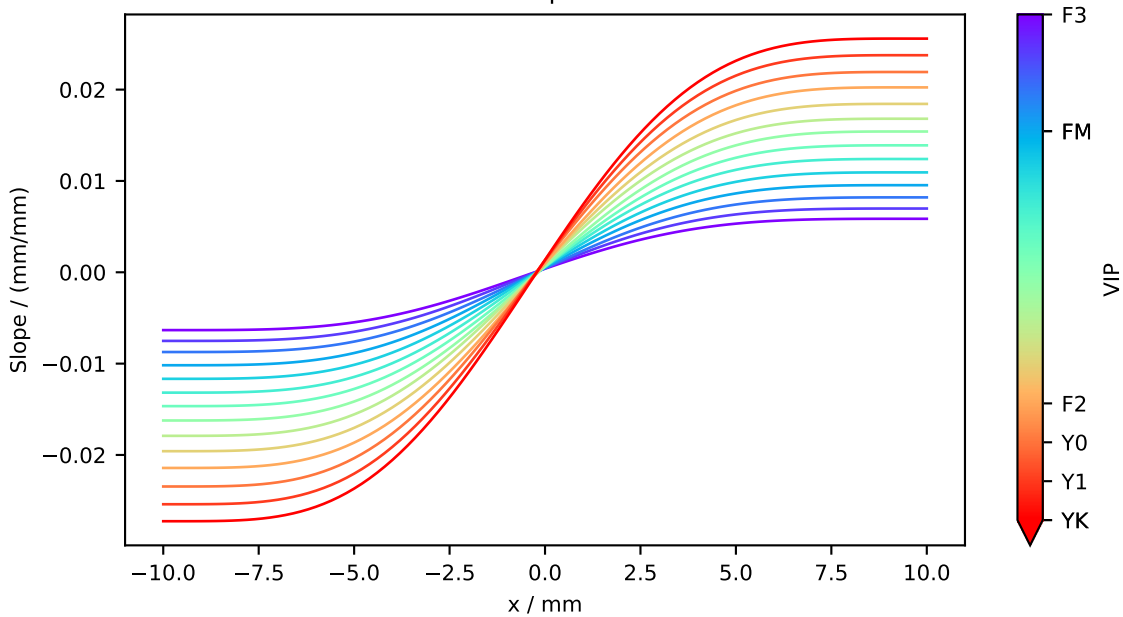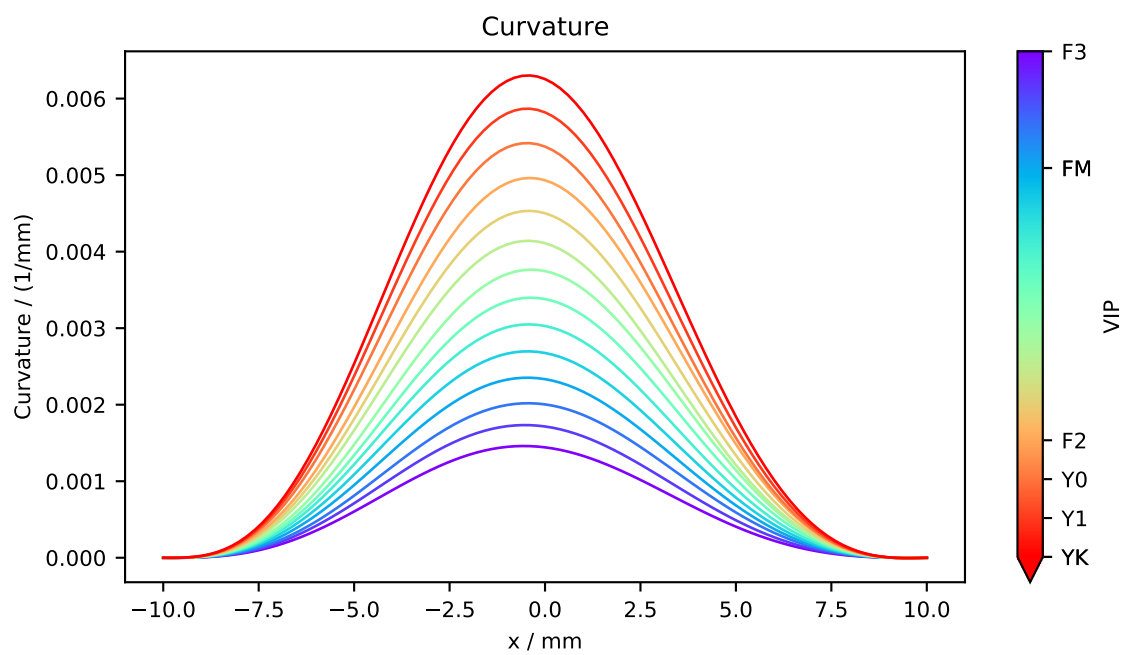

Supplement: Supplementary file 1 [file bioengineering-12-00862-s001.zip › File S3 Evaluation code/ExMechEva-0.1.2/data/Test/TBT/Series_Test/eva/cl12a-DIC_fit-M-eva.pdf]

cl12a - Fit-Bending  
Displacement

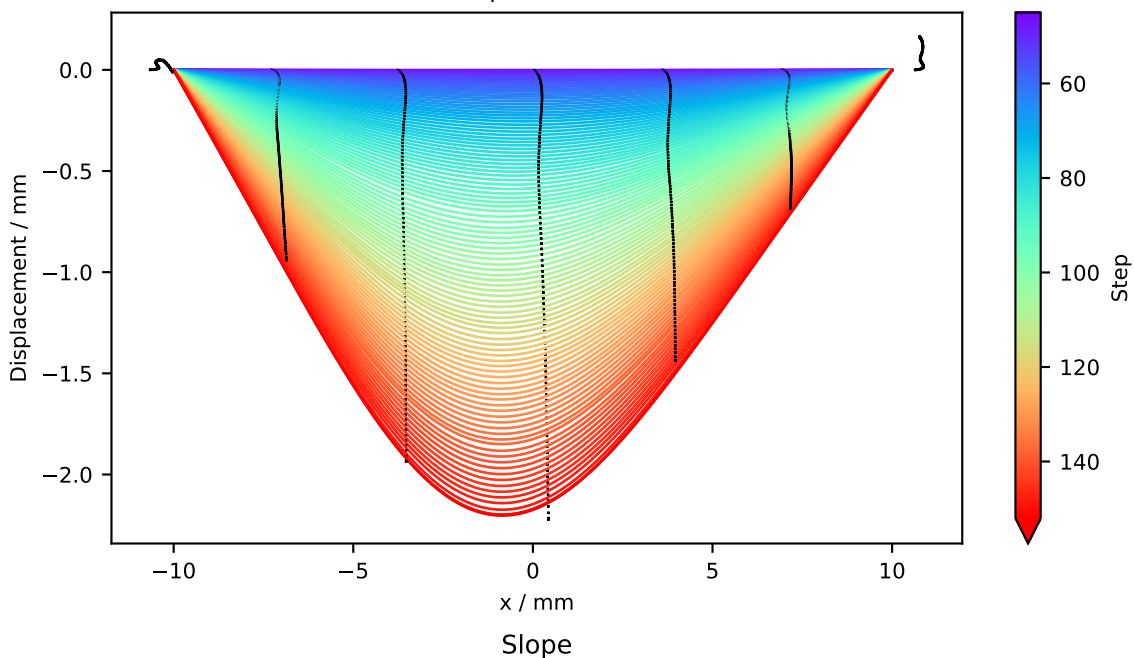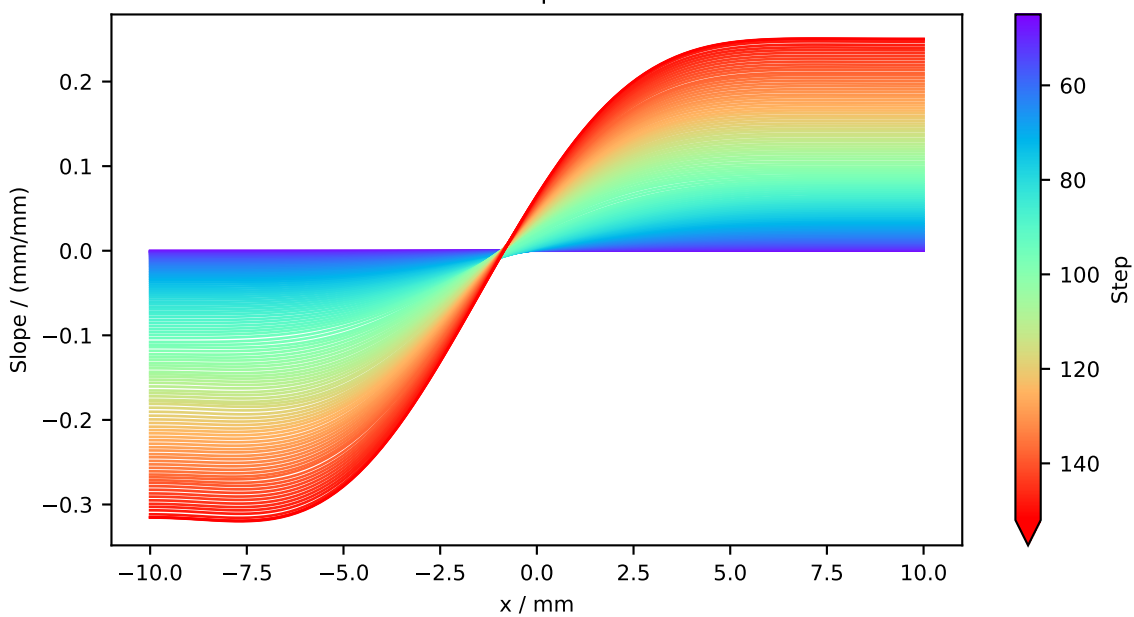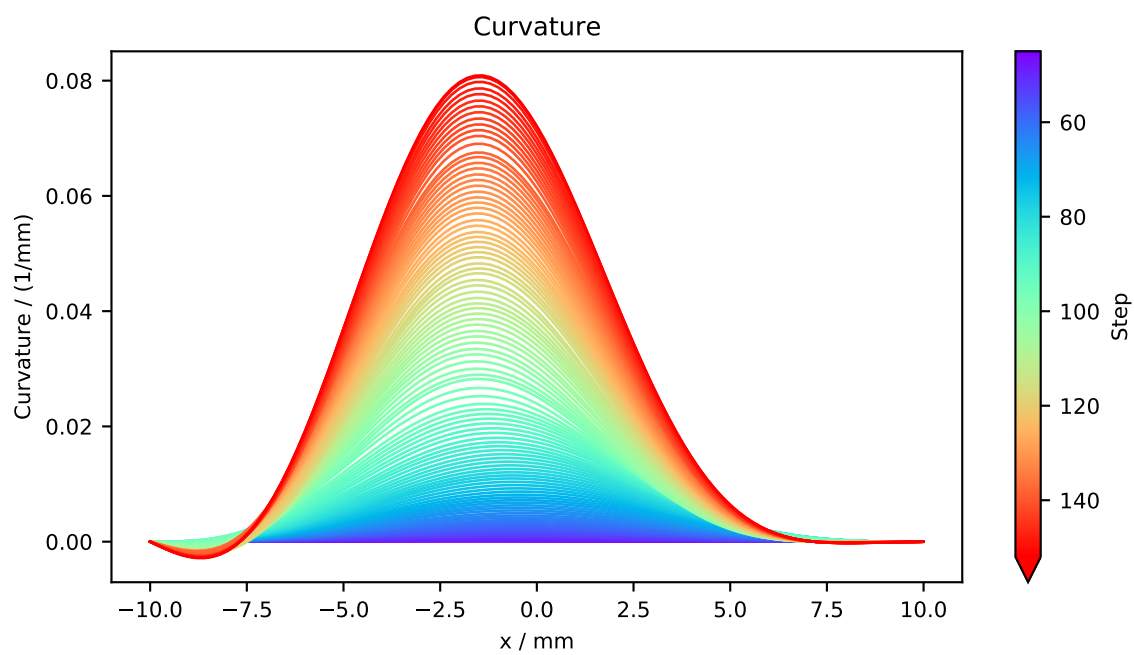

Supplement: Supplementary file 1 [file bioengineering-12-00862-s001.zip › File S3 Evaluation code/ExMechEva-0.1.2/data/Test/TBT/Series_Test/eva/cl12a-DIC_fit-M.pdf]

cl12a - Geometry

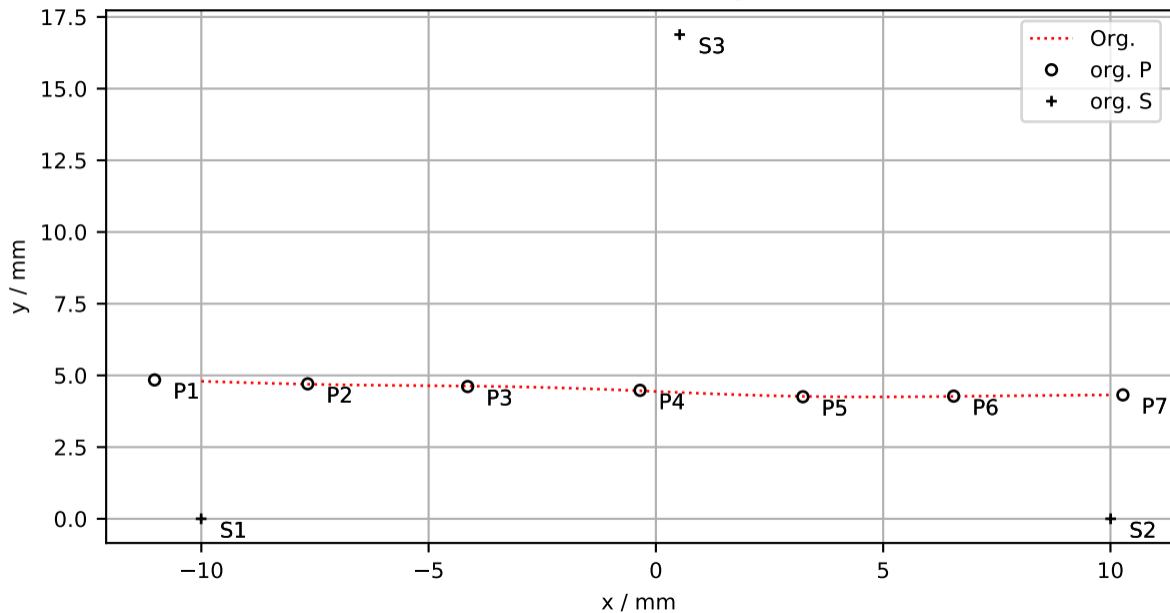

Supplement: Supplementary file 1 [file bioengineering-12-00862-s001.zip › File S3 Evaluation code/ExMechEva-0.1.2/data/Test/TBT/Series_Test/eva/cl12a-DIC_fit_Geo.pdf]

cl12a - Analyzing meas. force

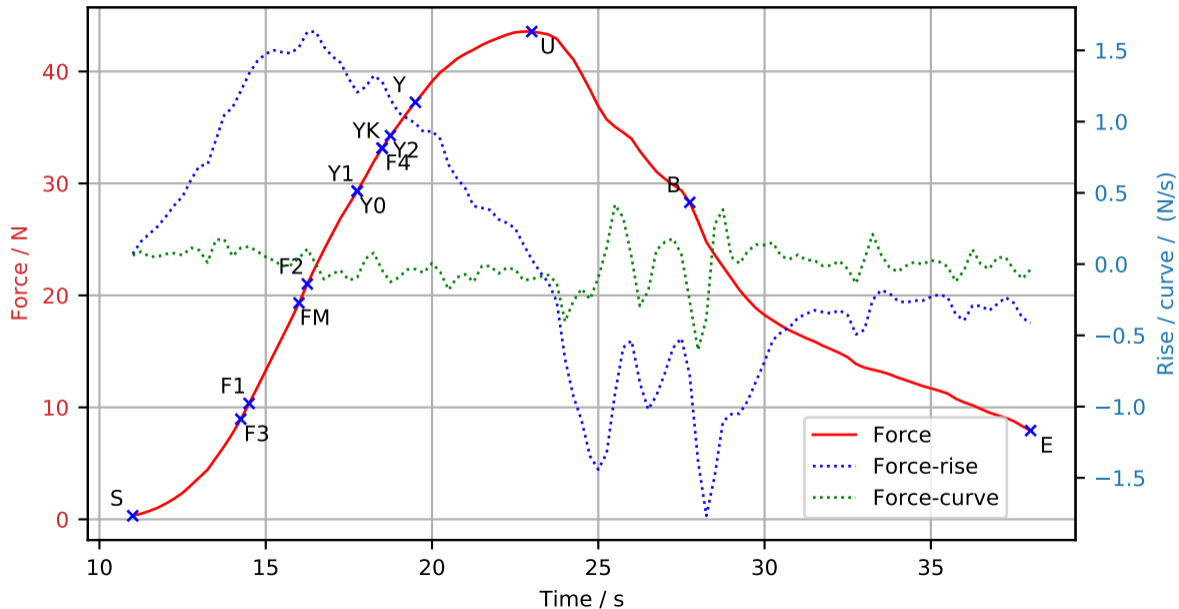

Supplement: Supplementary file 1 [file bioengineering-12-00862-s001.zip › File S3 Evaluation code/ExMechEva-0.1.2/data/Test/TBT/Series_Test/eva/cl12a-Fdricu.pdf]

cl12a - Width and Thickness

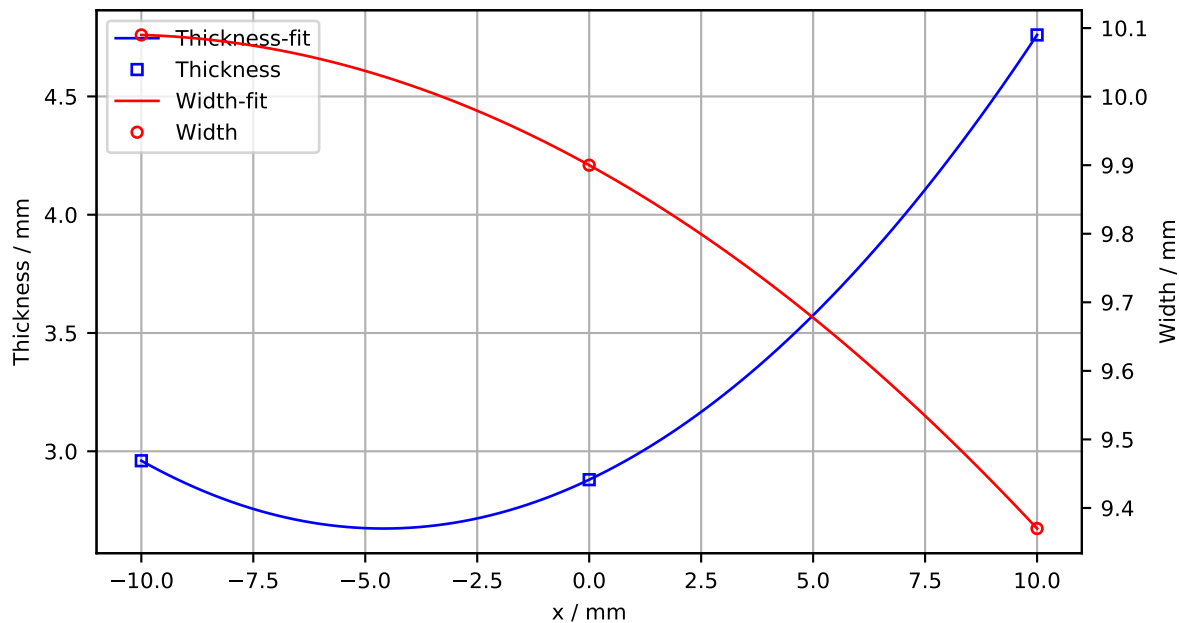

cl12a - Moment of Inertia

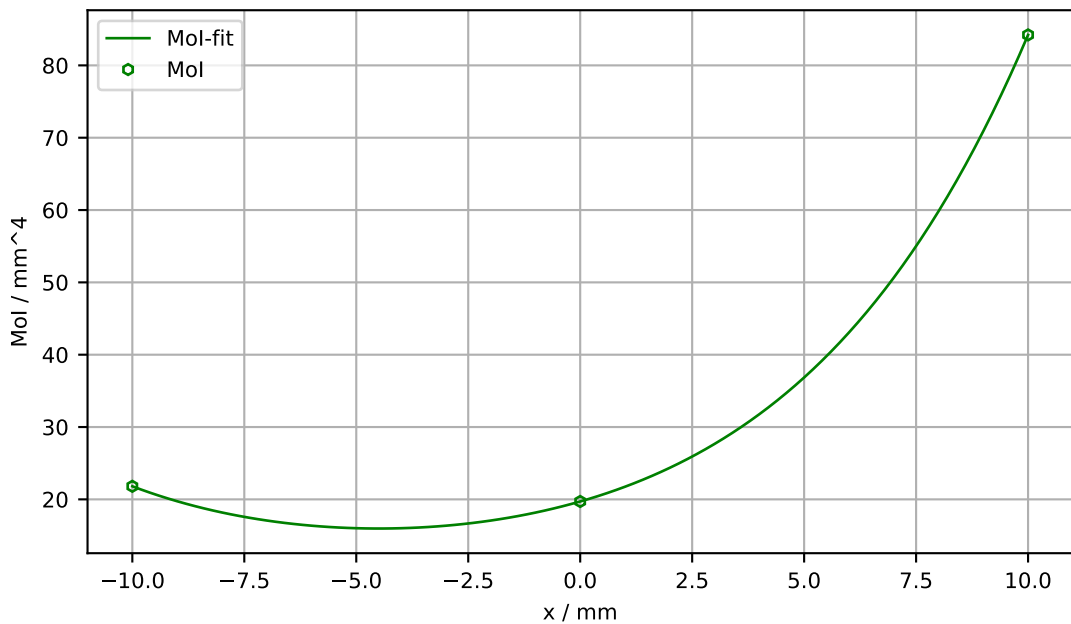

Supplement: Supplementary file 1 [file bioengineering-12-00862-s001.zip › File S3 Evaluation code/ExMechEva-0.1.2/data/Test/TBT/Series_Test/eva/cl12a-Geo.pdf]

cl12a - Incremental Fit-full - evaluation range  
Displacement

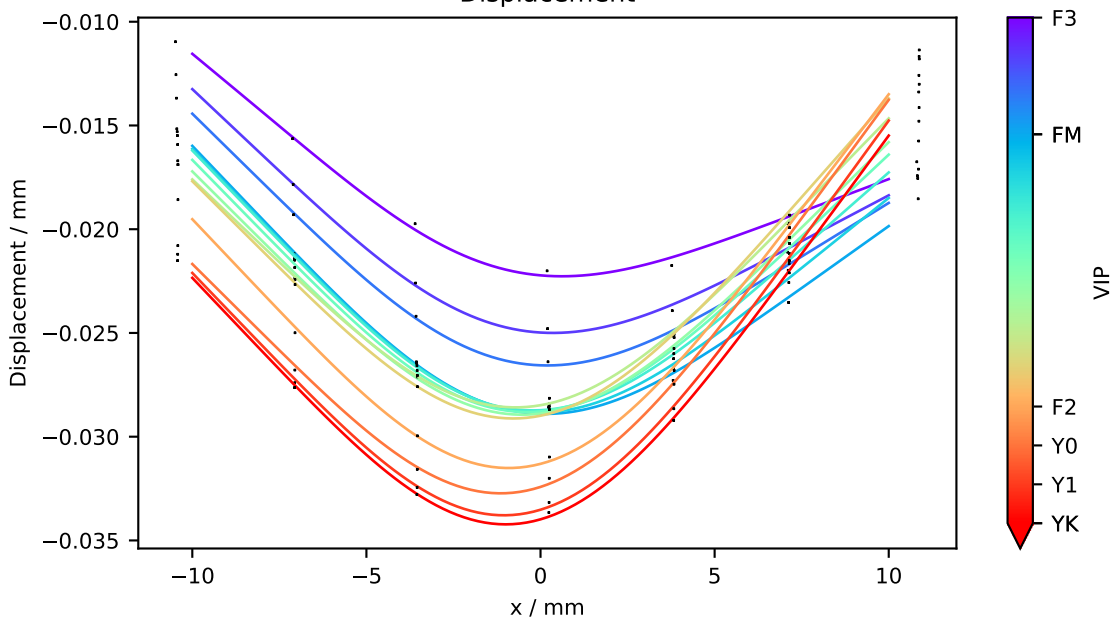

Slope

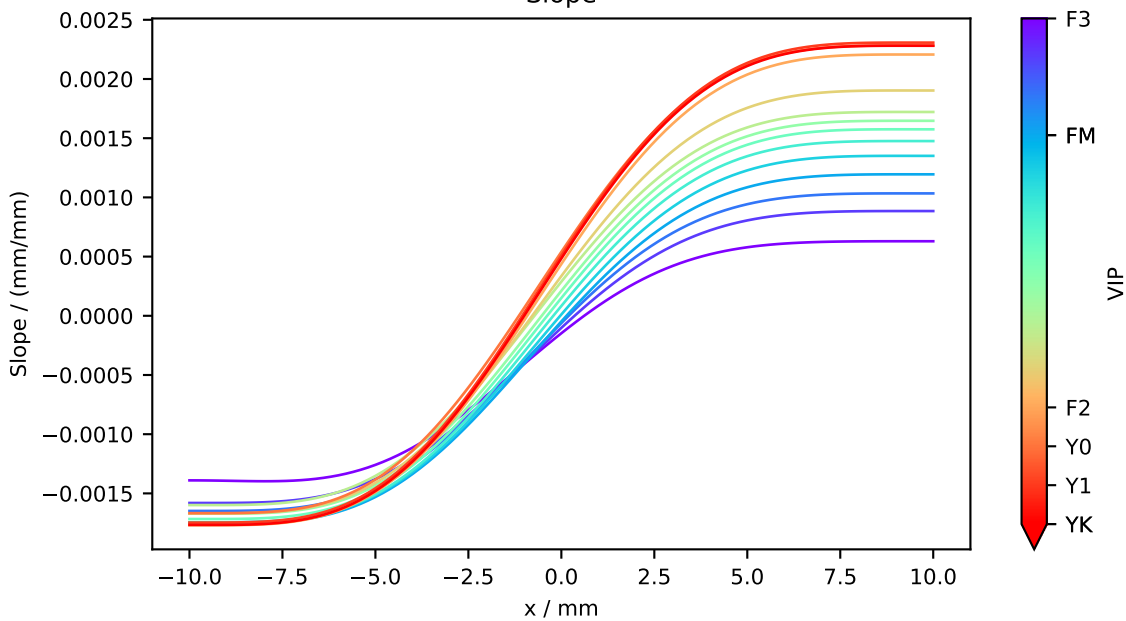

Curvature

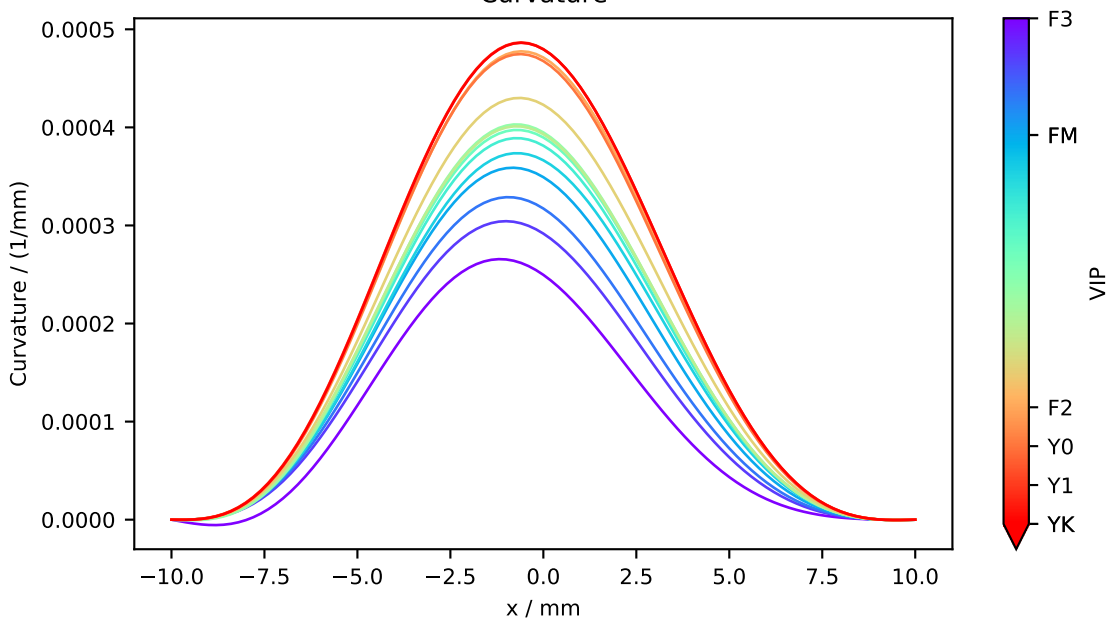

Supplement: Supplementary file 1 [file bioengineering-12-00862-s001.zip › File S3 Evaluation code/ExMechEva-0.1.2/data/Test/TBT/Series_Test/eva/cl12a-INC_fit-A-eva.pdf]

cl12a - Incremental Fit-full  
Displacement

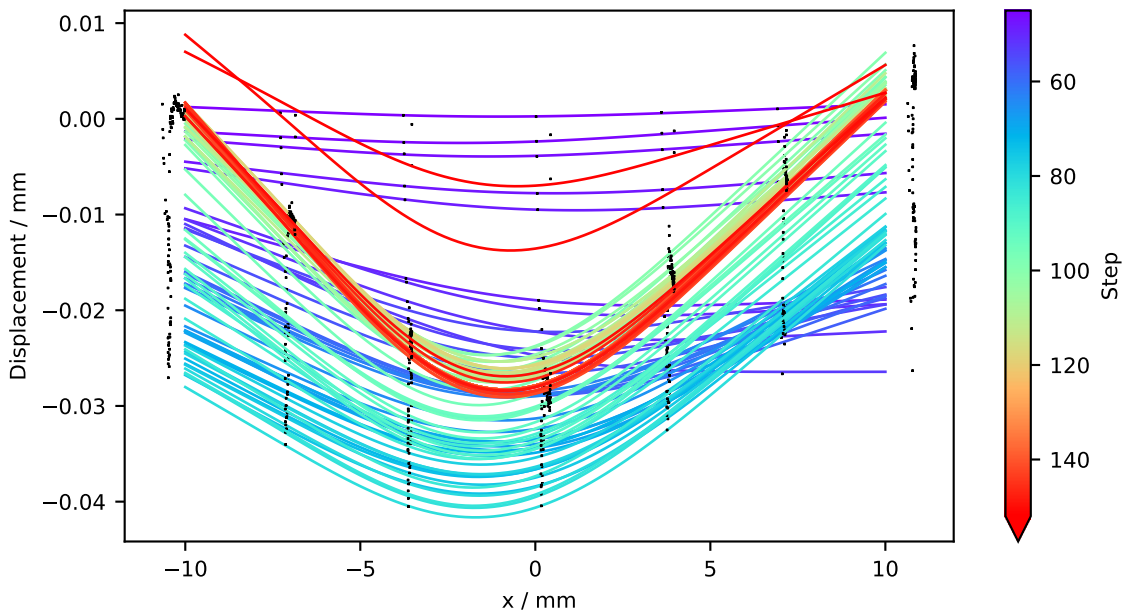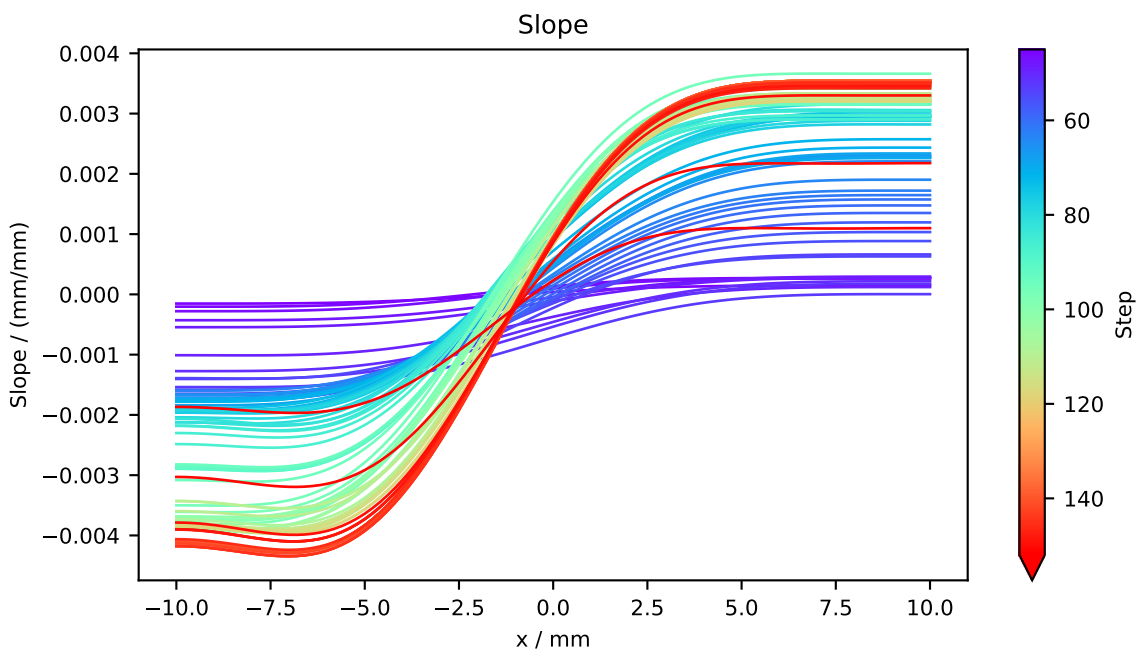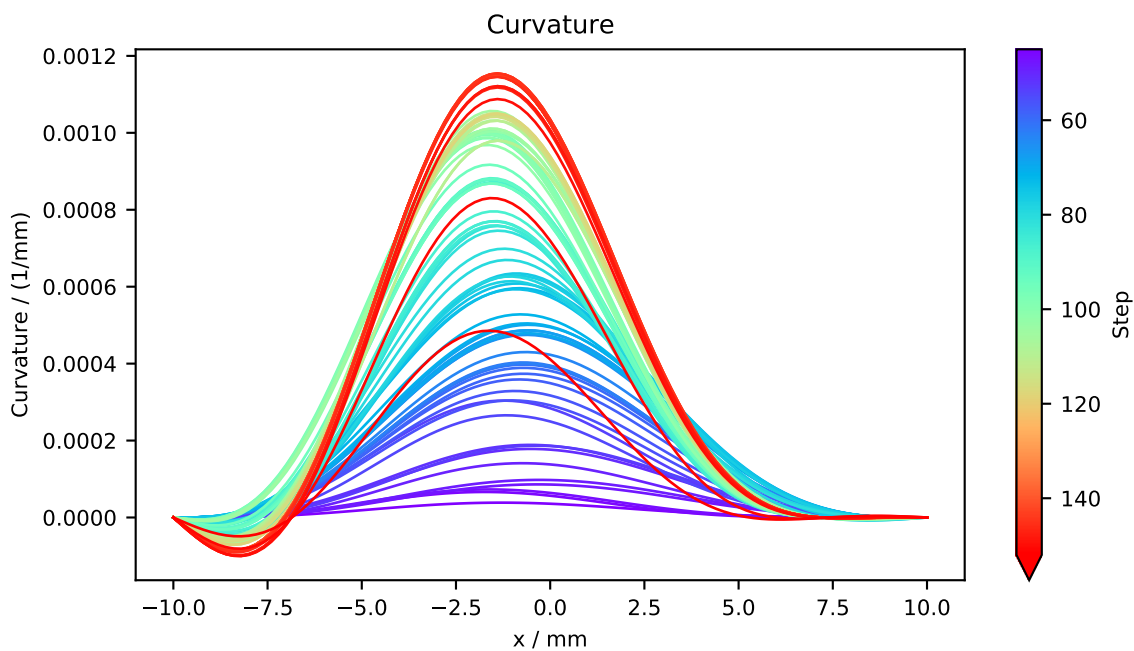

Supplement: Supplementary file 1 [file bioengineering-12-00862-s001.zip › File S3 Evaluation code/ExMechEva-0.1.2/data/Test/TBT/Series_Test/eva/cl12a-INC_fit-A.pdf]

cl12a - Fit-compare-inc - Displacement for step 92

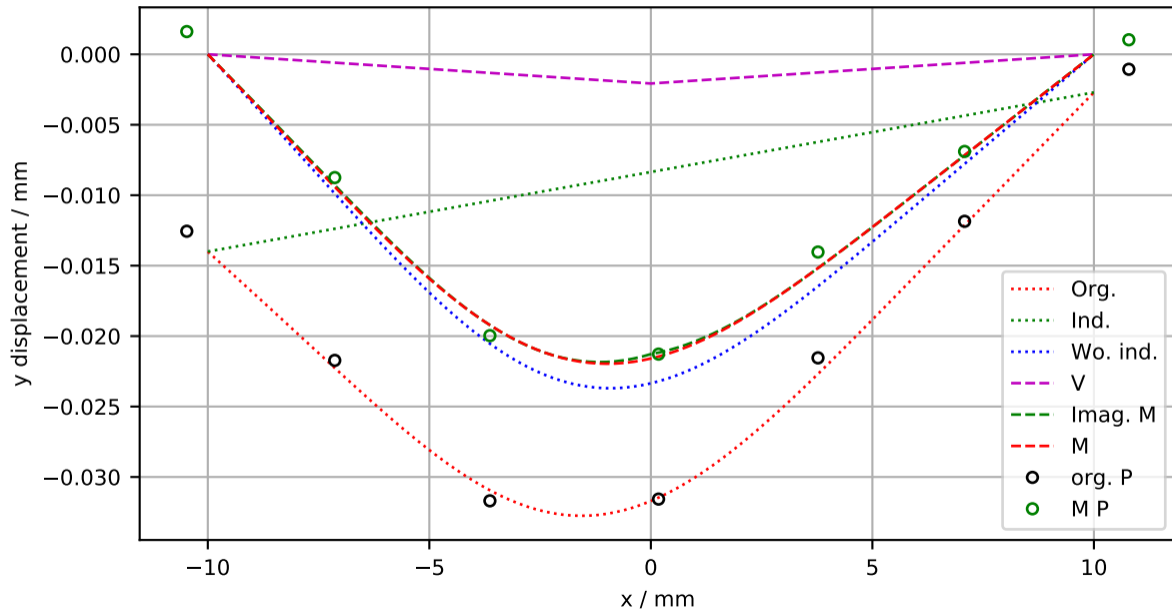

Supplement: Supplementary file 1 [file bioengineering-12-00862-s001.zip › File S3 Evaluation code/ExMechEva-0.1.2/data/Test/TBT/Series_Test/eva/cl12a-INC_fit-bl_U-d0.pdf]

cl12a - Incremental Fit-Bending - evaluation range  
Displacement

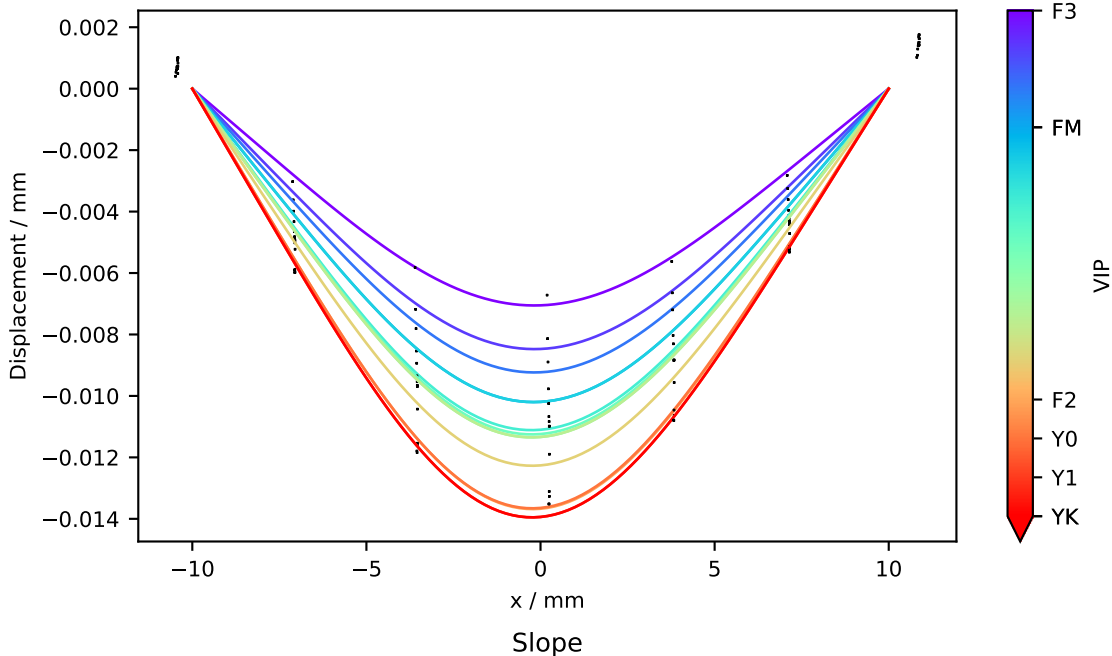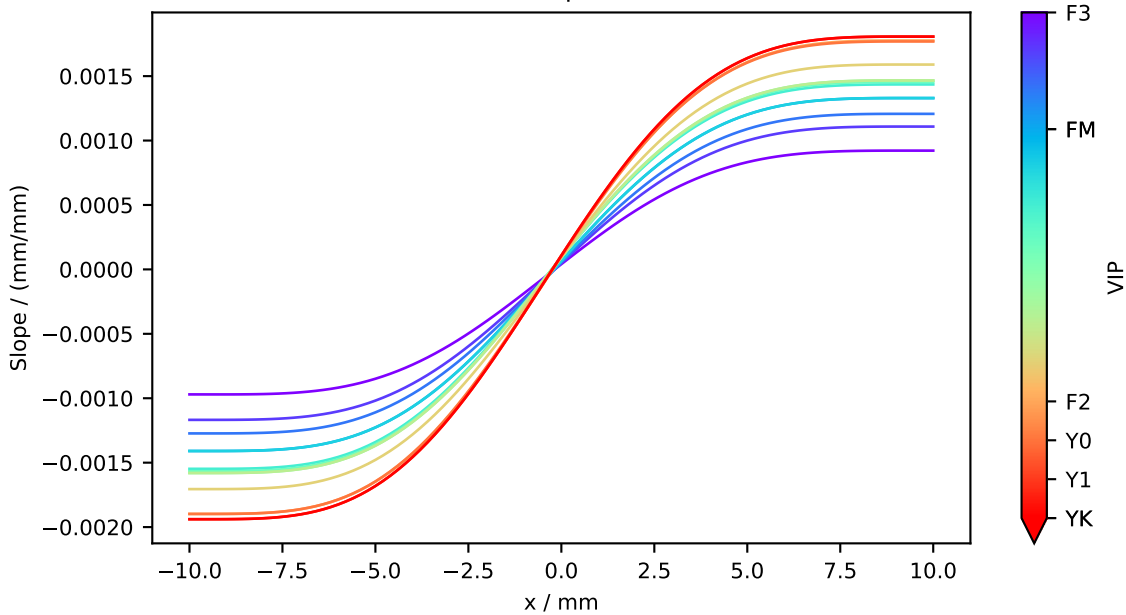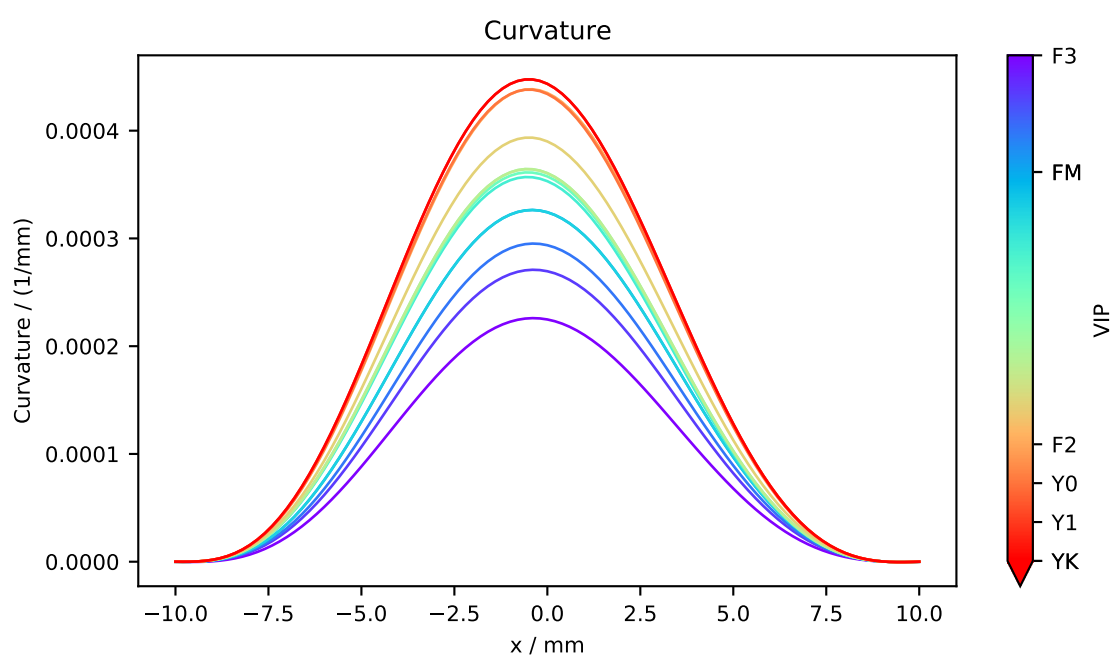

Supplement: Supplementary file 1 [file bioengineering-12-00862-s001.zip › File S3 Evaluation code/ExMechEva-0.1.2/data/Test/TBT/Series_Test/eva/cl12a-INC_fit-M-eva.pdf]

cl12a - Incremental Fit-Bending  
Displacement

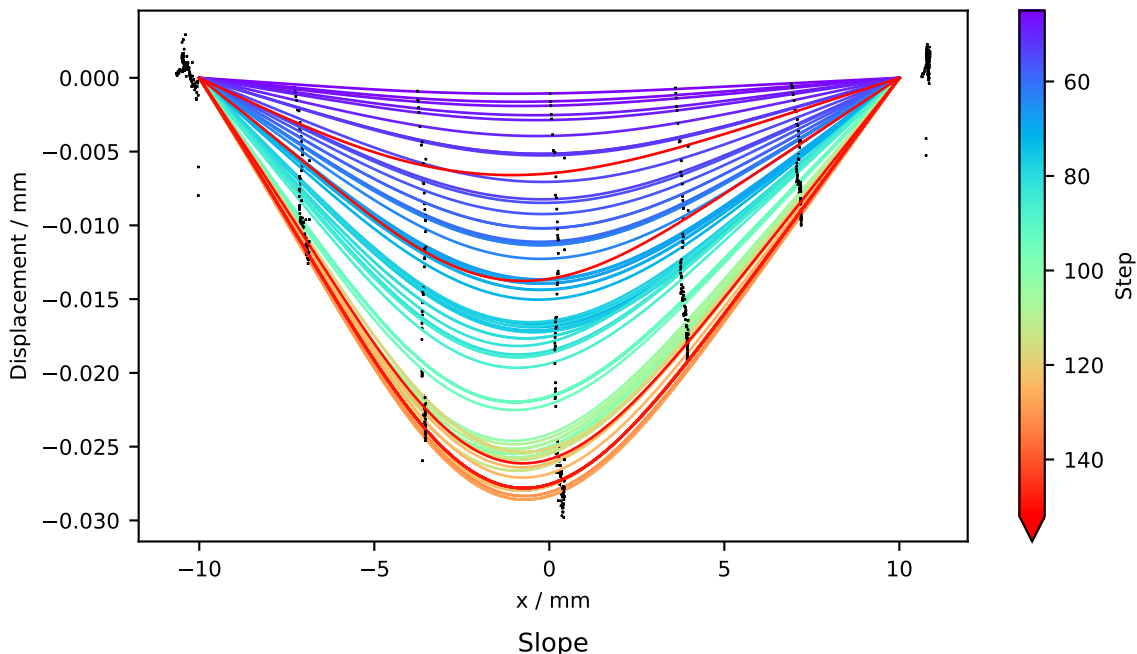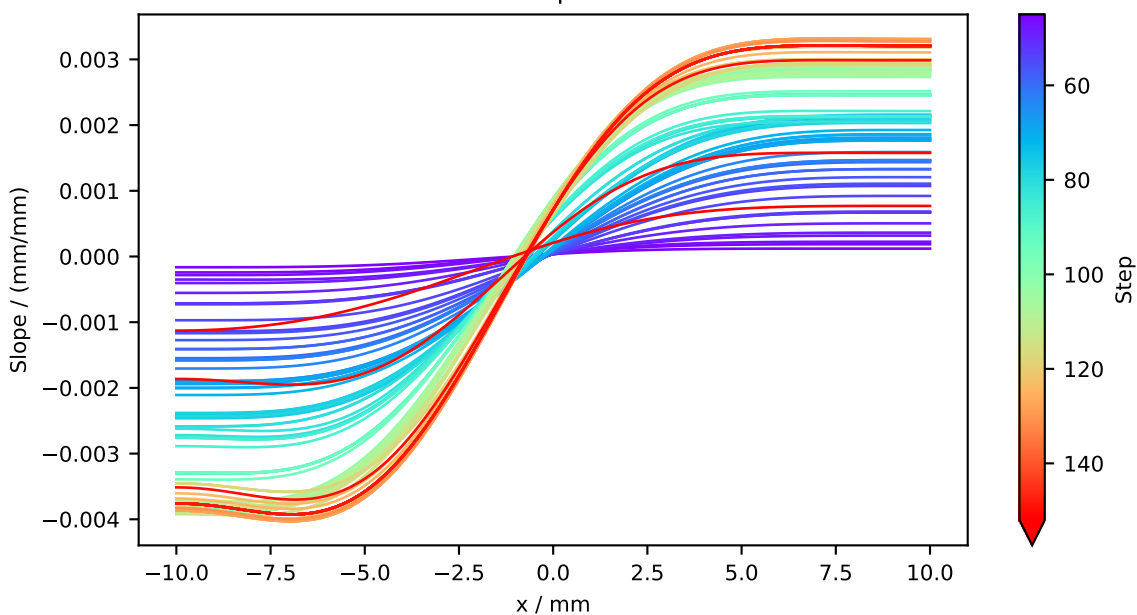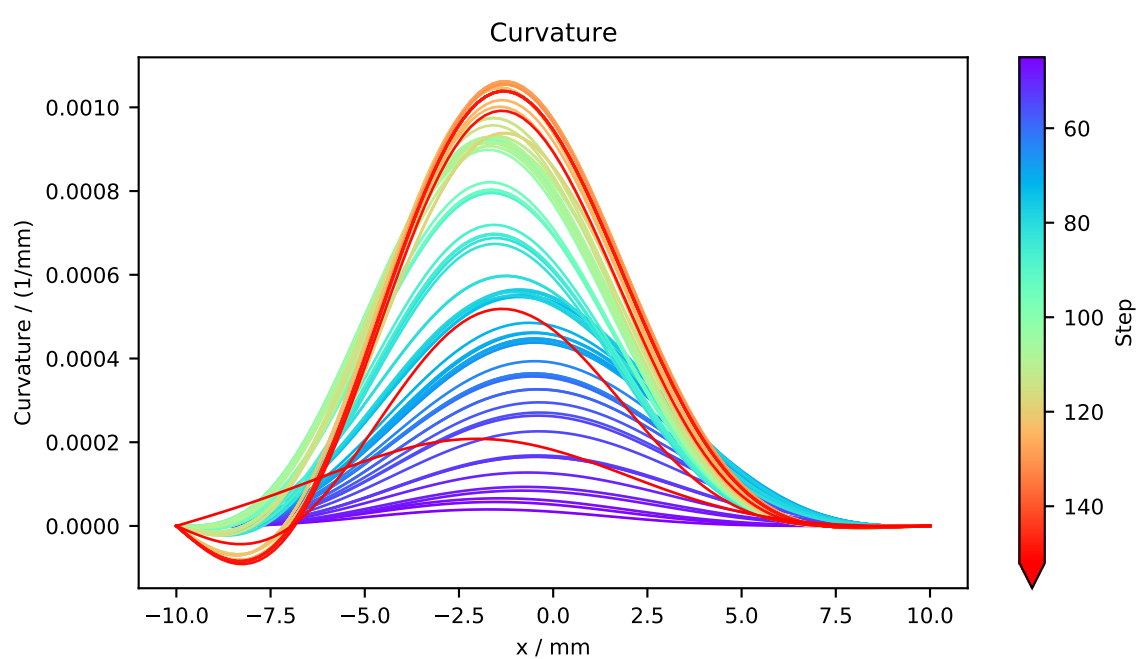

Supplement: Supplementary file 1 [file bioengineering-12-00862-s001.zip › File S3 Evaluation code/ExMechEva-0.1.2/data/Test/TBT/Series_Test/eva/cl12a-INC_fit-M.pdf]

cl12a - Measuring

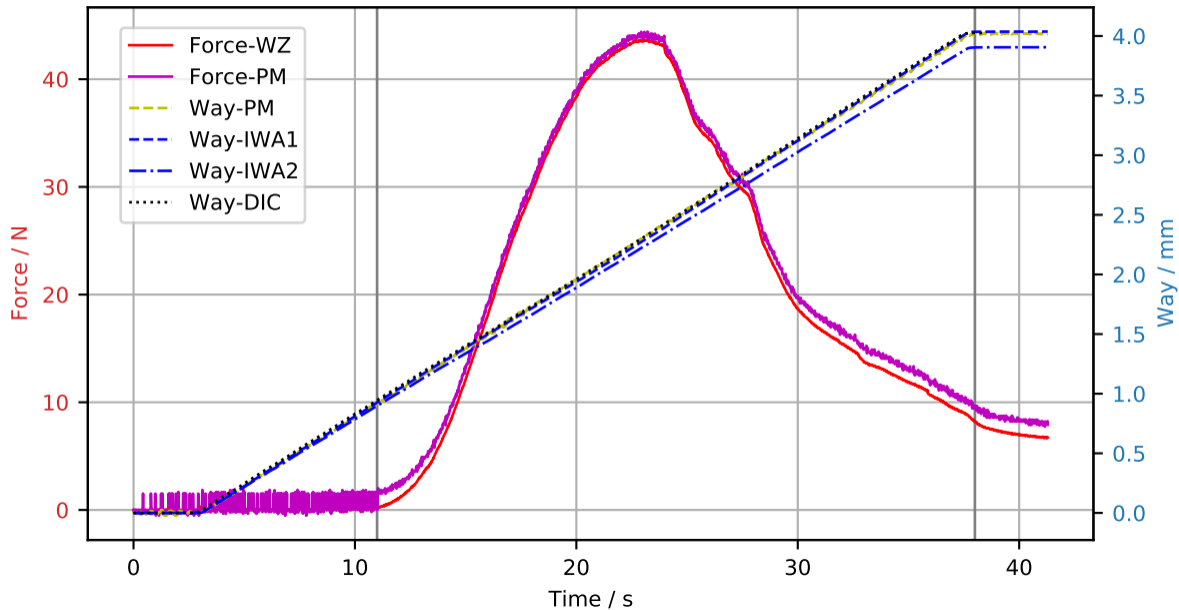

Supplement: Supplementary file 1 [file bioengineering-12-00862-s001.zip › File S3 Evaluation code/ExMechEva-0.1.2/data/Test/TBT/Series_Test/eva/cl12a-meas.pdf]

cl12a - Measuring (used)

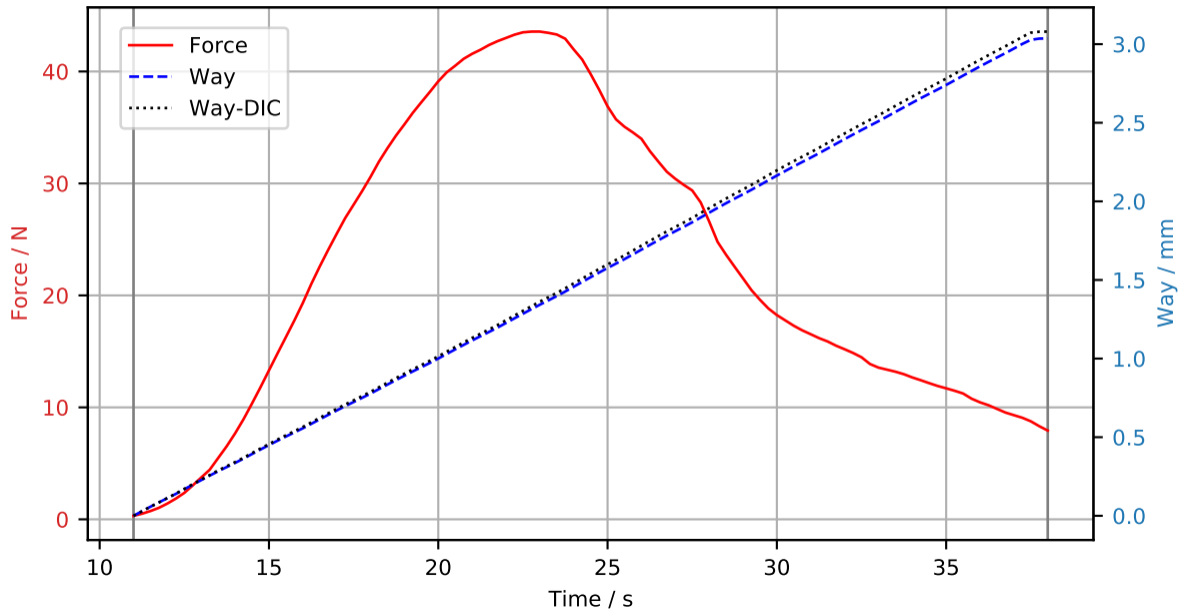

Supplement: Supplementary file 1 [file bioengineering-12-00862-s001.zip › File S3 Evaluation code/ExMechEva-0.1.2/data/Test/TBT/Series_Test/eva/cl12a-meas_u.pdf]

cl12a - Stress vs. strain curve of different opt. strain calculations

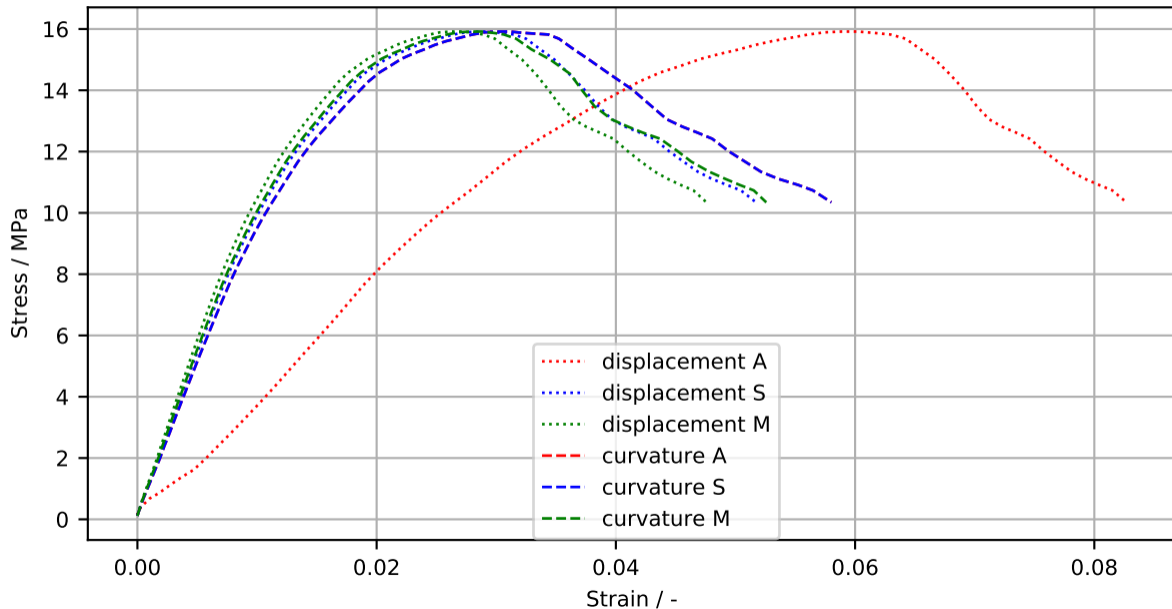

Supplement: Supplementary file 1 [file bioengineering-12-00862-s001.zip › File S3 Evaluation code/ExMechEva-0.1.2/data/Test/TBT/Series_Test/eva/cl12a-sigeps_dicvgl.pdf]

cl12a - Stress vs. strain curve, final part, with labels

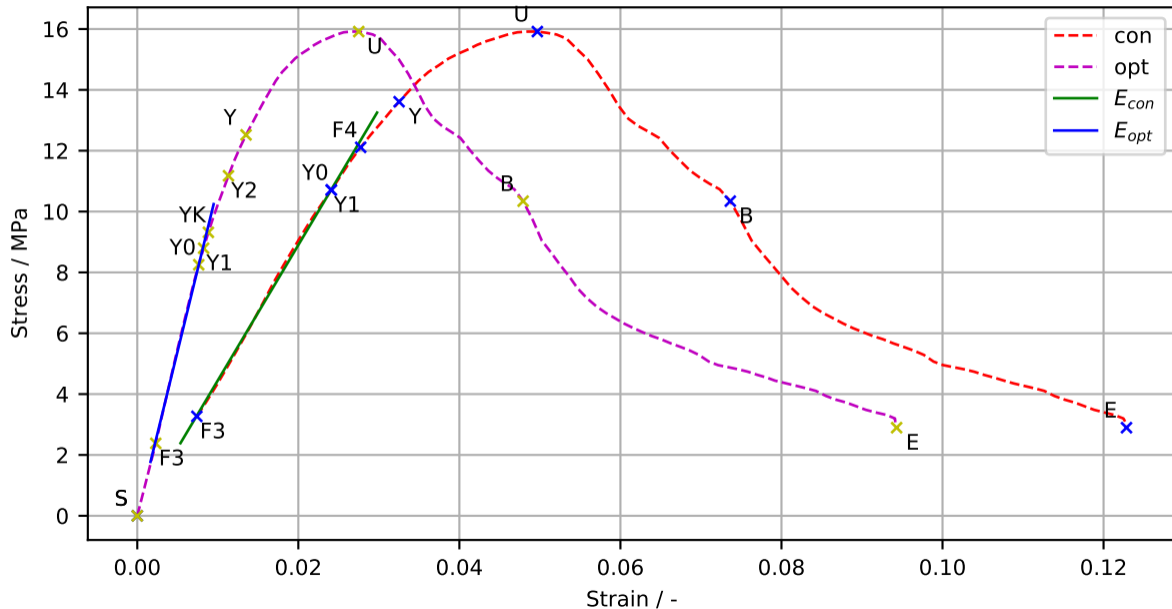

Supplement: Supplementary file 1 [file bioengineering-12-00862-s001.zip › File S3 Evaluation code/ExMechEva-0.1.2/data/Test/TBT/Series_Test/eva/cl12a-sigeps_fin.pdf]

cl12a - Stress vs. strain curve with labels

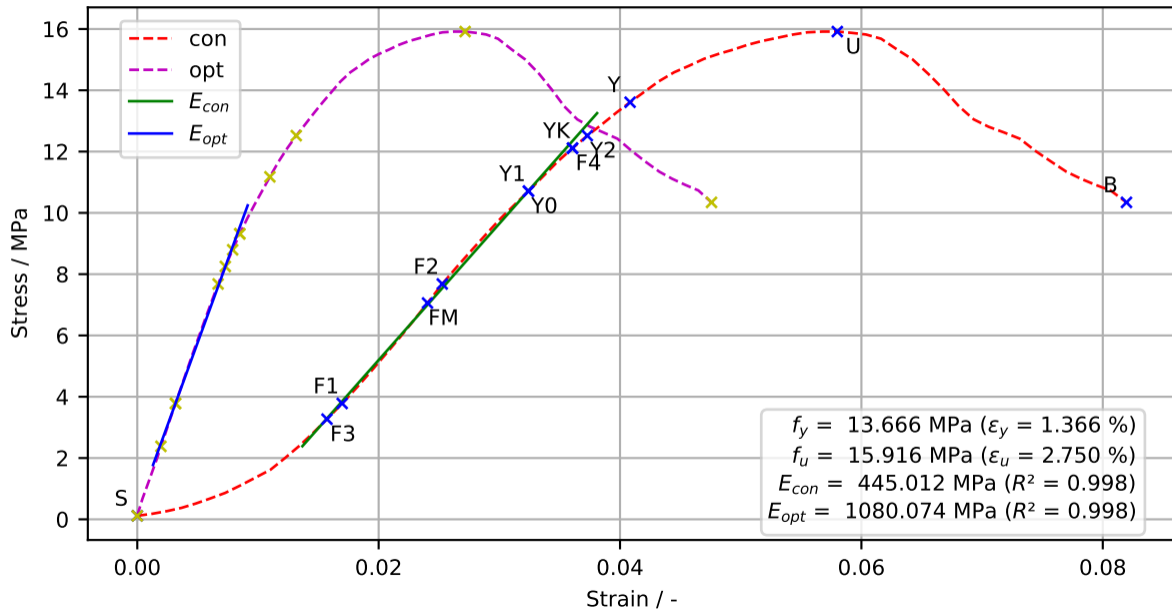

Supplement: Supplementary file 1 [file bioengineering-12-00862-s001.zip › File S3 Evaluation code/ExMechEva-0.1.2/data/Test/TBT/Series_Test/eva/cl12a-sigeps_wl.pdf]

# cl12a - Stress vs. strain curve - yield point determination

Conventional strain

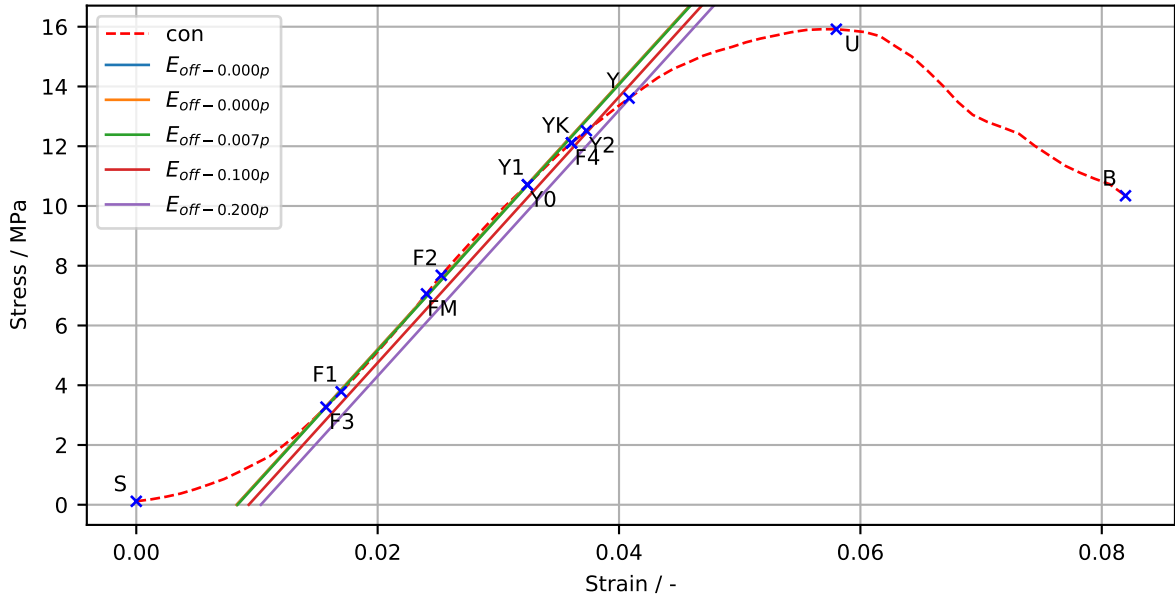

Optical strain

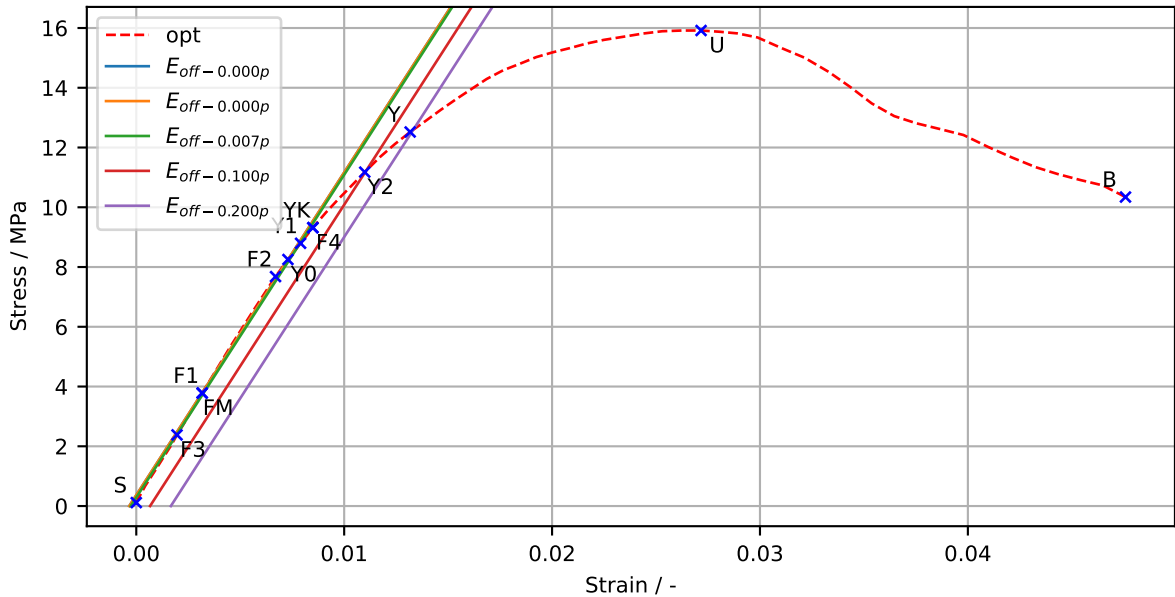

Supplement: Supplementary file 1 [file bioengineering-12-00862-s001.zip › File S3 Evaluation code/ExMechEva-0.1.2/data/Test/TBT/Series_Test/eva/cl12a-sigeps_yielddet.pdf]

cl12a - Way-measuring time difference

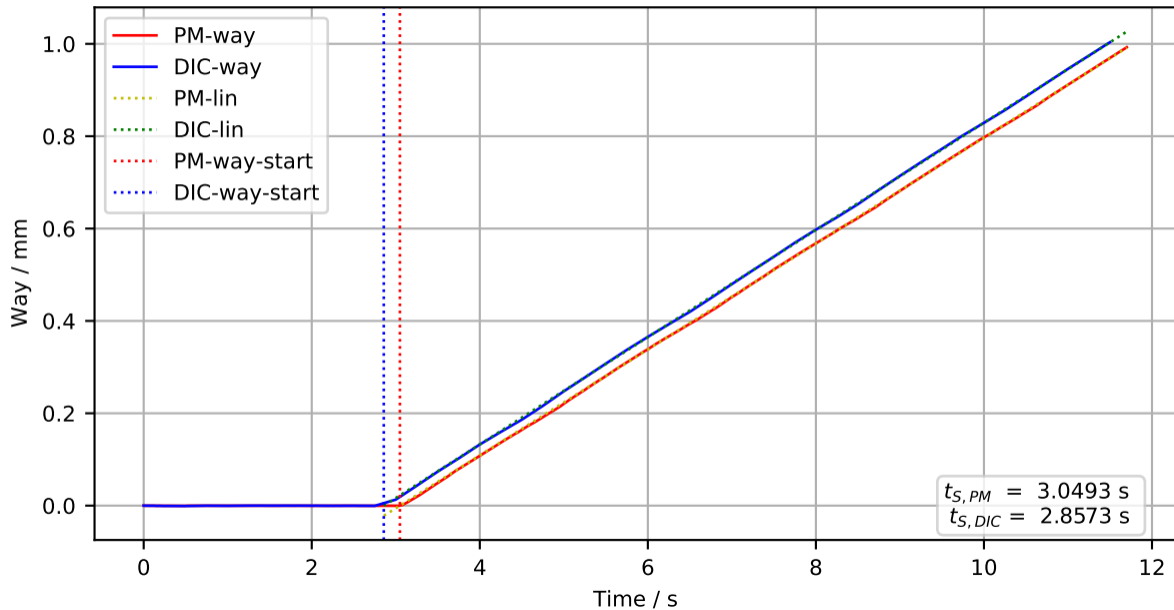

Supplement: Supplementary file 1 [file bioengineering-12-00862-s001.zip › File S3 Evaluation code/ExMechEva-0.1.2/data/Test/TBT/Series_Test/eva/cl12a-toff.pdf]

# cl12a - Compare method A

All Steps

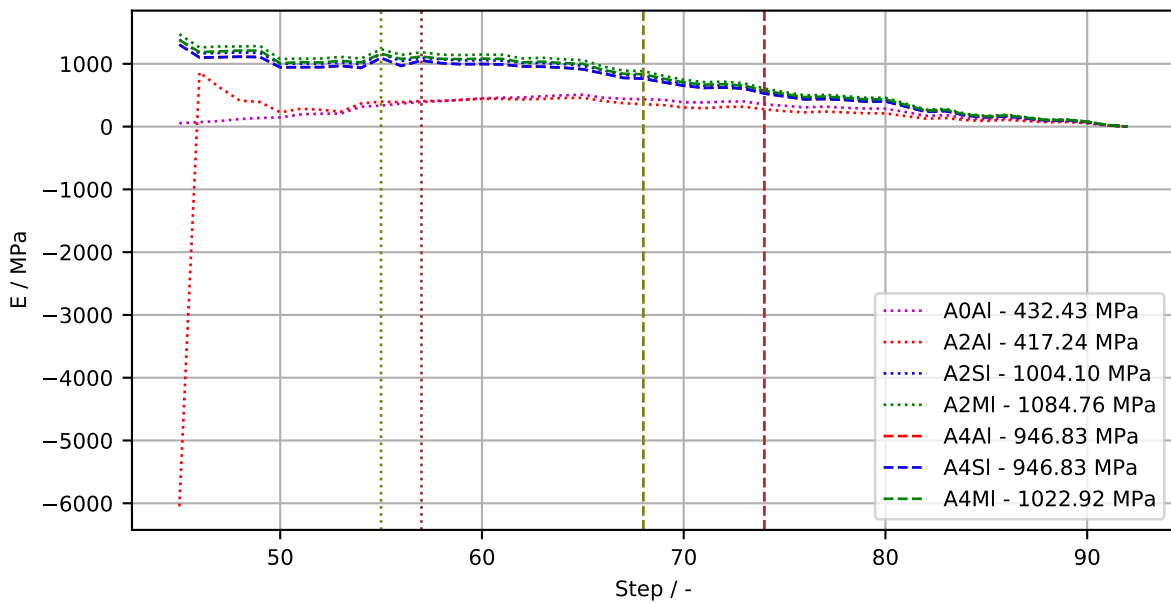

Improved determination range

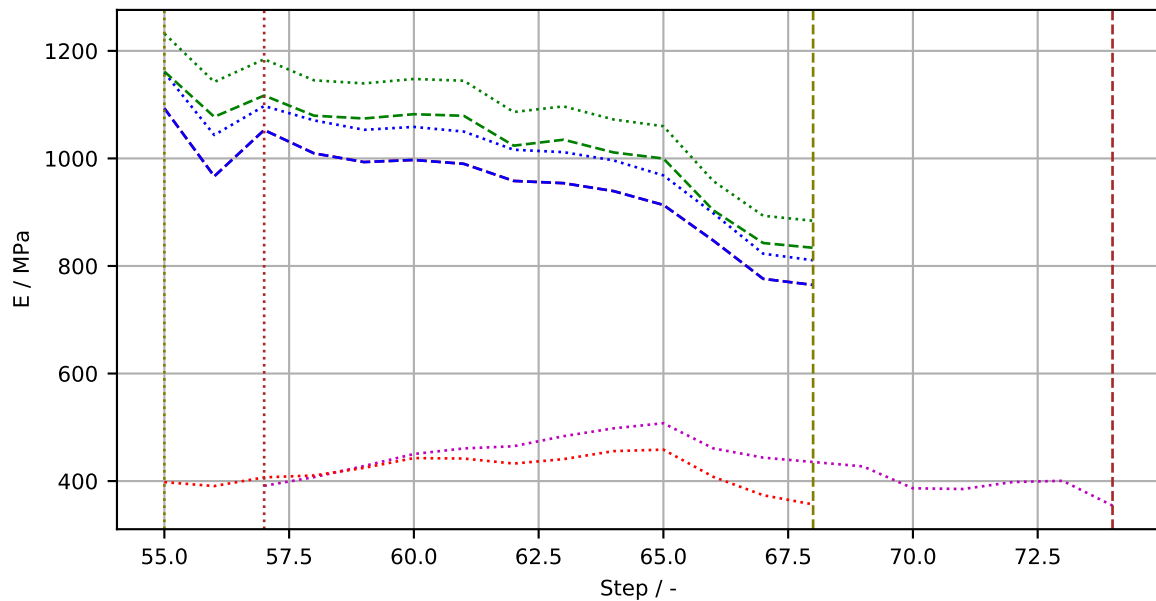

Supplement: Supplementary file 1 [file bioengineering-12-00862-s001.zip › File S3 Evaluation code/ExMechEva-0.1.2/data/Test/TBT/Series_Test/eva/cl12a-YM-Me_A.pdf]

# cl12a - Compare method B

## All Steps

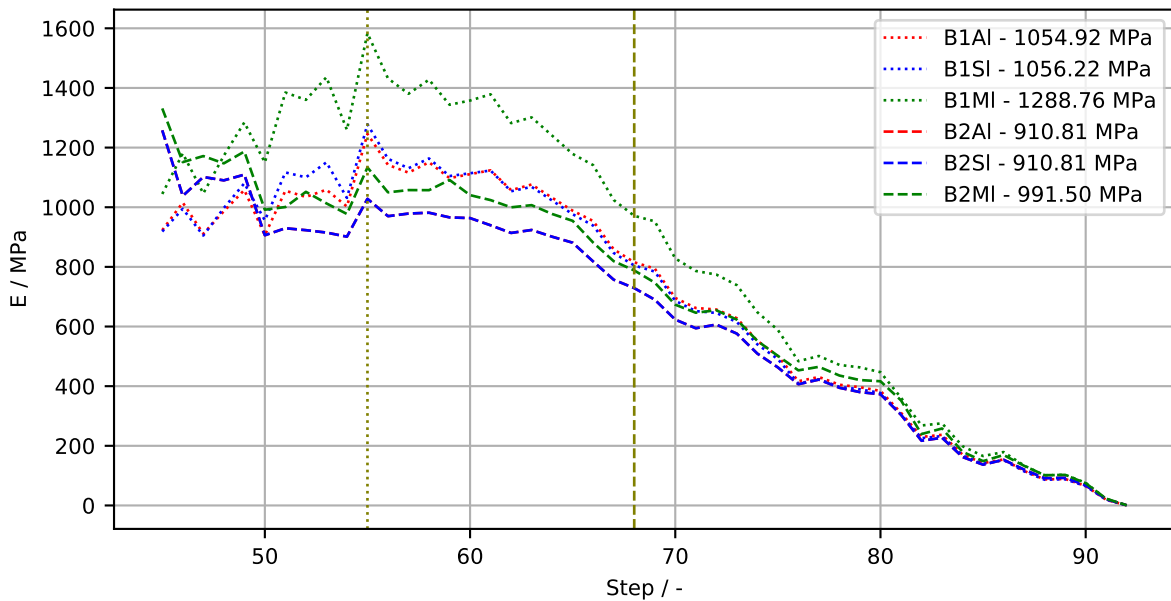

## Improved determination range

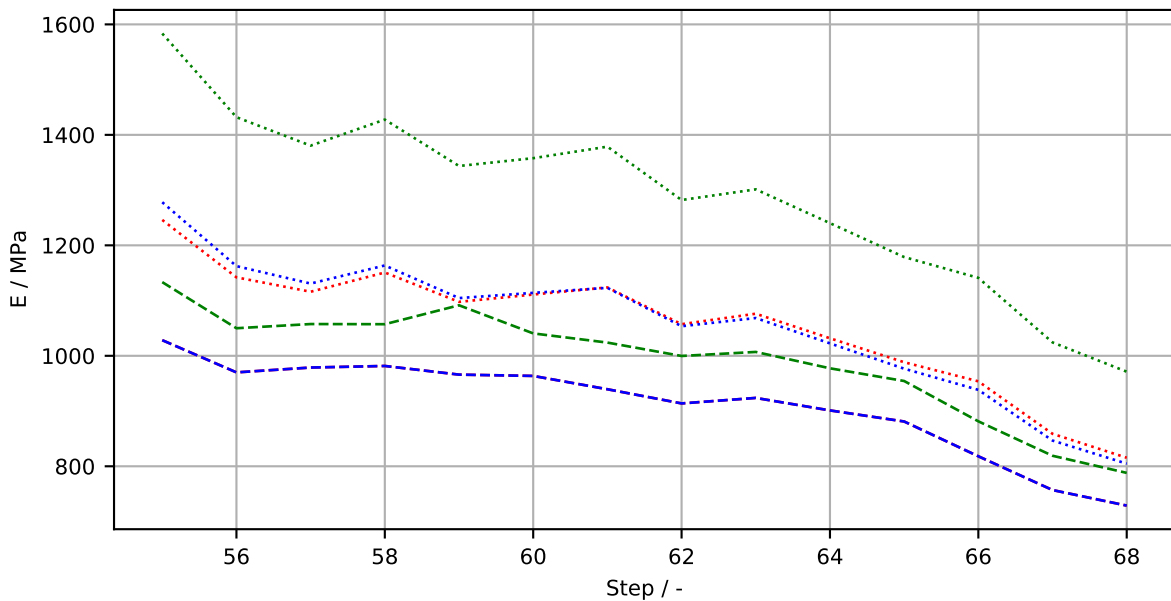

Supplement: Supplementary file 1 [file bioengineering-12-00862-s001.zip › File S3 Evaluation code/ExMechEva-0.1.2/data/Test/TBT/Series_Test/eva/cl12a-YM-Me_B.pdf]

# cl12a - Compare method C

## All Steps

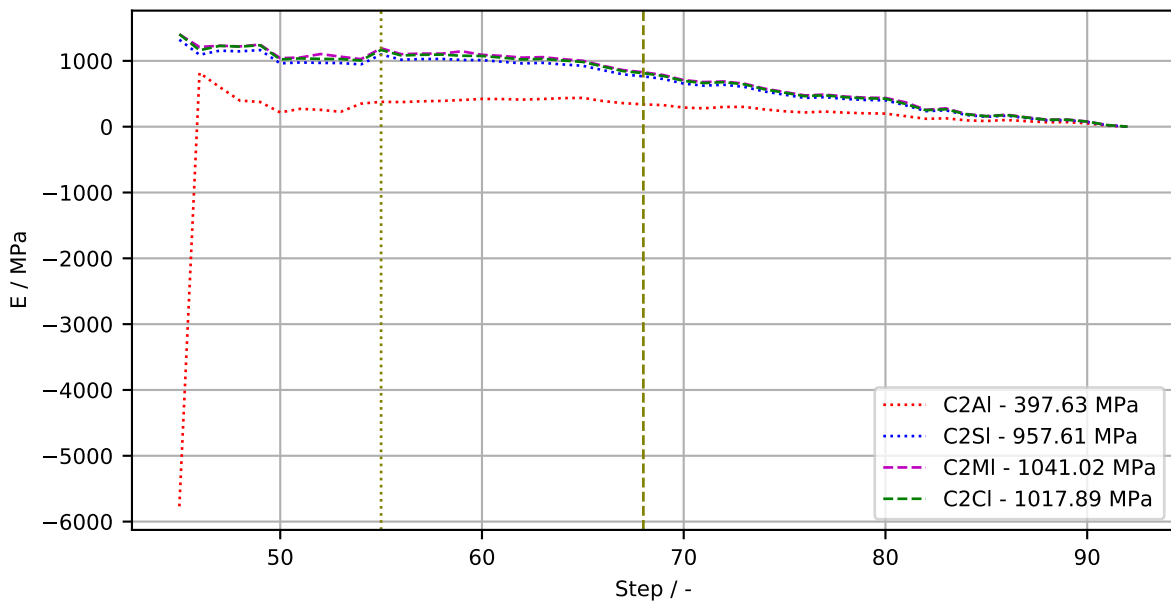

## Improved determination range

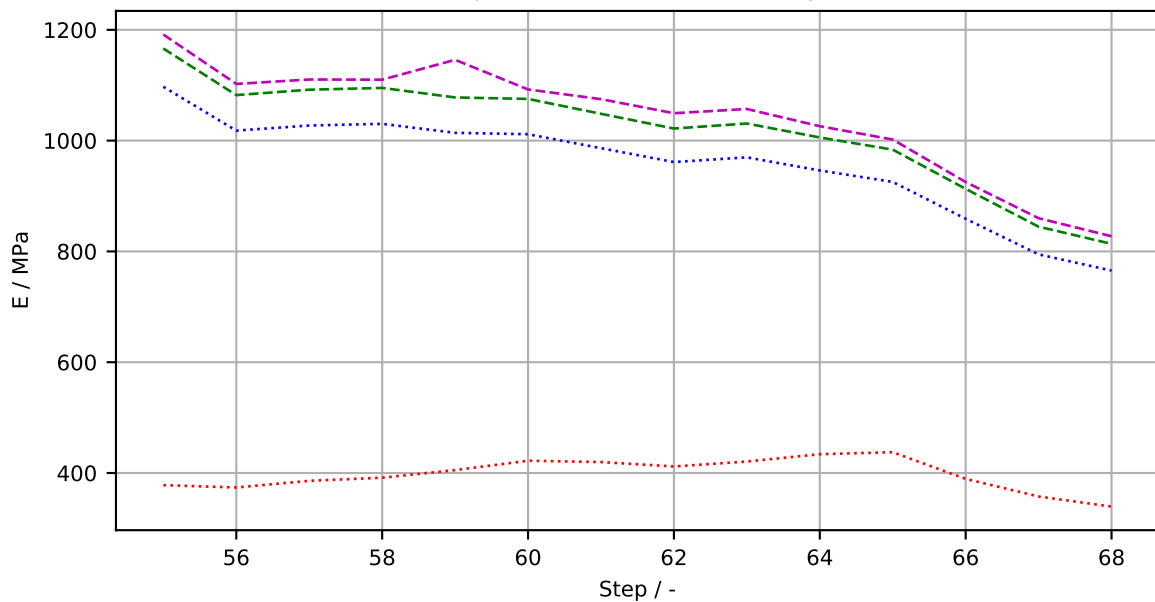

Supplement: Supplementary file 1 [file bioengineering-12-00862-s001.zip › File S3 Evaluation code/ExMechEva-0.1.2/data/Test/TBT/Series_Test/eva/cl12a-YM-Me_C.pdf]

# cl12a - Compare method D

## All Steps

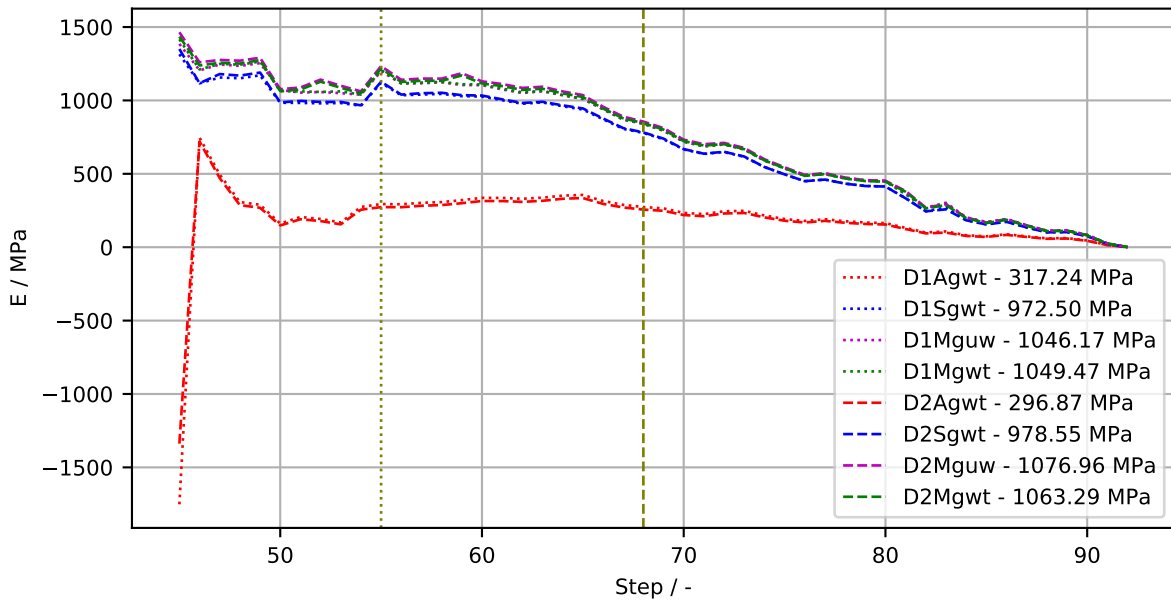

## Improved determination range

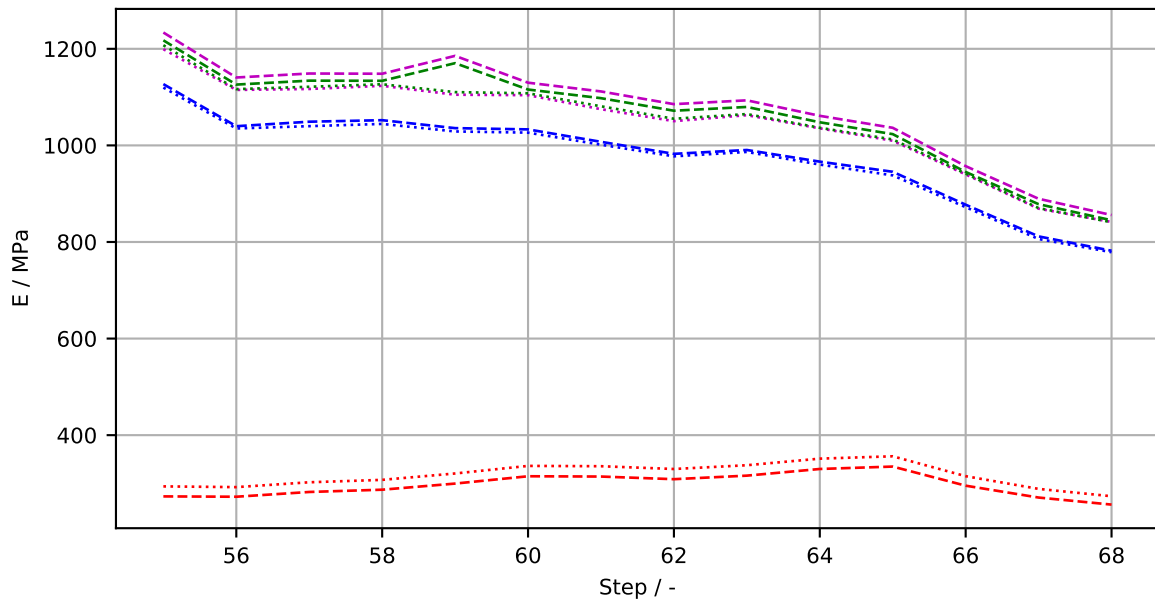

Supplement: Supplementary file 1 [file bioengineering-12-00862-s001.zip › File S3 Evaluation code/ExMechEva-0.1.2/data/Test/TBT/Series_Test/eva/cl12a-YM-Me_D.pdf]

# cl12a - Compare method E

## All Steps

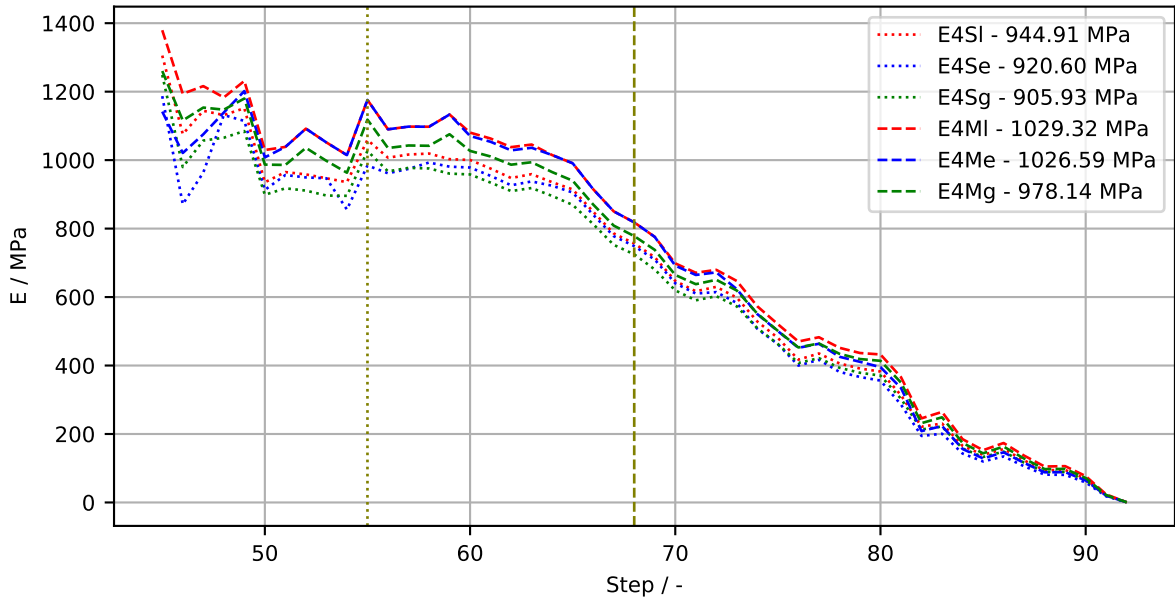

## Improved determination range

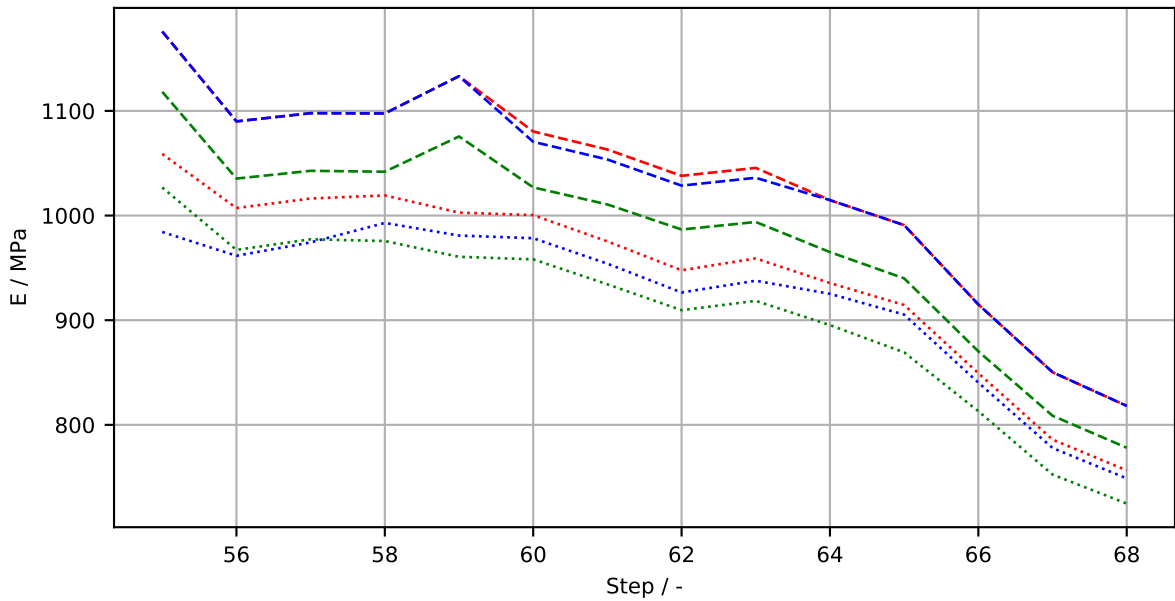

Supplement: Supplementary file 1 [file bioengineering-12-00862-s001.zip › File S3 Evaluation code/ExMechEva-0.1.2/data/Test/TBT/Series_Test/eva/cl12a-YM-Me_E.pdf]

# cl12a - Compare method F

## All Steps

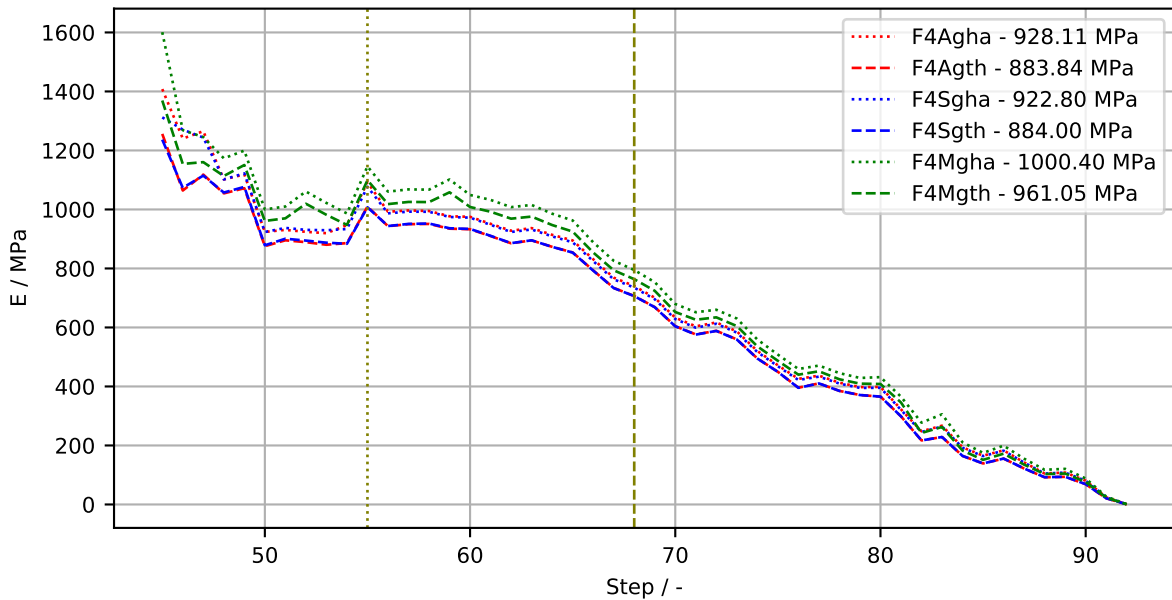

## Improved determination range

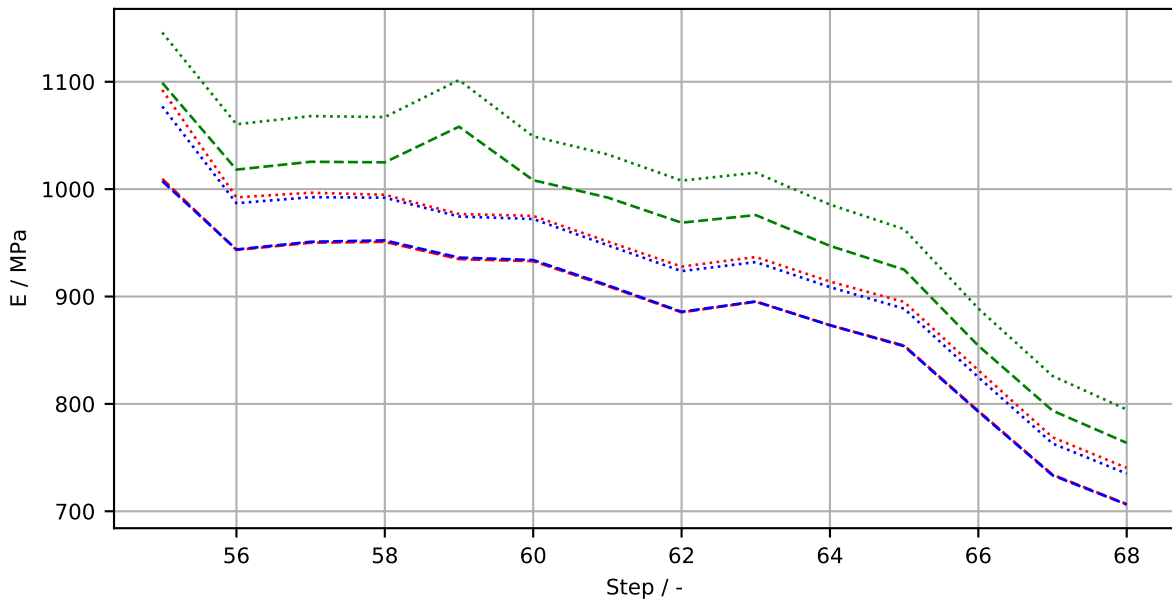

Supplement: Supplementary file 1 [file bioengineering-12-00862-s001.zip › File S3 Evaluation code/ExMechEva-0.1.2/data/Test/TBT/Series_Test/eva/cl12a-YM-Me_F.pdf]

# cl12a - Compare method G

## All Steps

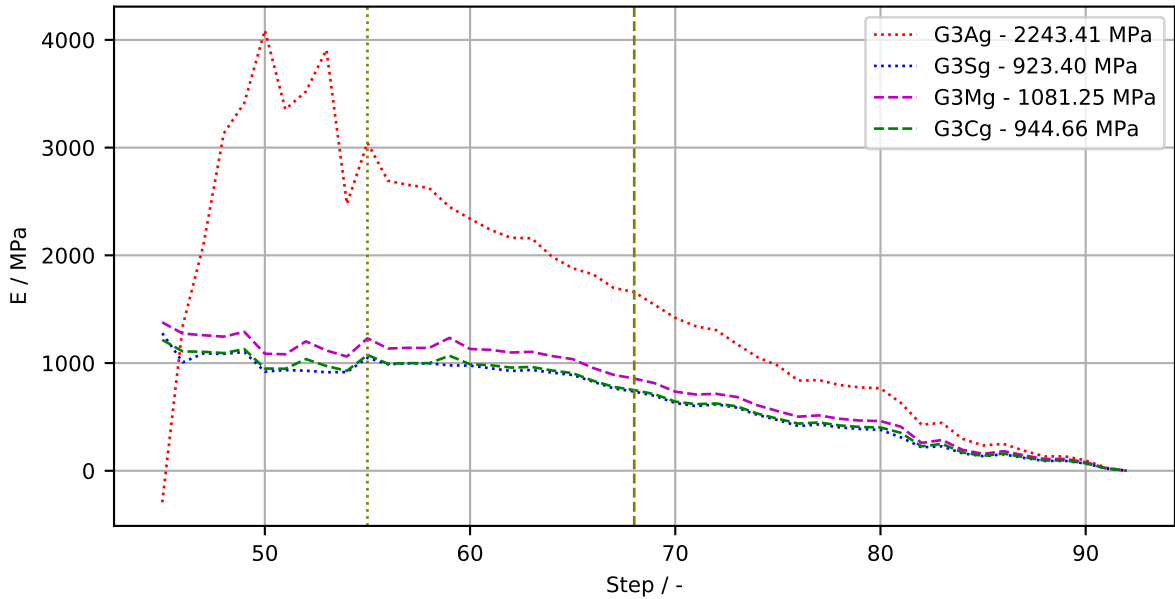

## Improved determination range

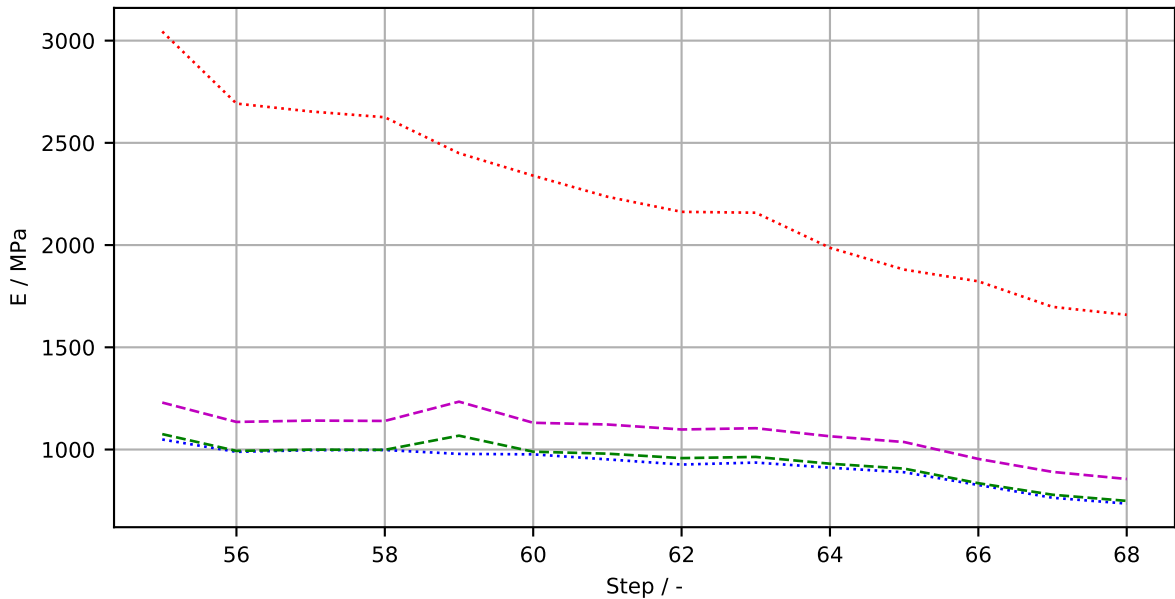

Supplement: Supplementary file 1 [file bioengineering-12-00862-s001.zip › File S3 Evaluation code/ExMechEva-0.1.2/data/Test/TBT/Series_Test/eva/cl12a-YM-Me_G.pdf]

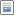

Supplement: Supplementary file 1 [file bioengineering-12-00862-s001.zip › File S3 Evaluation code/ExMechEva-0.1.2/docs/_build/html/_static/file.png]

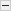

Supplement: Supplementary file 1 [file bioengineering-12-00862-s001.zip › File S3 Evaluation code/ExMechEva-0.1.2/docs/_build/html/_static/minus.png]

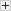

Supplement: Supplementary file 1 [file bioengineering-12-00862-s001.zip › File S3 Evaluation code/ExMechEva-0.1.2/docs/_build/html/_static/plus.png]
