# Supplementary material for: Approach to Standardized Material Characterization of the Human Lumbopelvic System: Testing and Evaluation
Source: Bioengineering (Basel). 2025 Aug 11;12(8):862. doi: 10.3390/bioengineering12080862 (PMC12383908; doi:10.3390/bioengineering12080862)
Supplement: Supplementary file 1 [file bioengineering-12-00862-s001.zip › File S1 Standard operation procedure–Testing.pdf]

Standard Operating Procedure – Testing

Approach to standardized material characterization of the human lumbopelvic system – testing and evaluation

Marc Gebhardt [1,2,†], **Sascha Kurz** [3,*,†], Fanny Grundmann [4], Thomas Klink [1], Volker Slowik [1], Christoph-Eckhard Heyde [3,5], Hanno Steinke [2]

1 Institute of Experimental Mechanics, Faculty of Civil Engineering, Leipzig University of Applied Sciences, Leipzig, Germany
2 Institute of Anatomy, Faculty of Medicine, Leipzig University, Leipzig, Germany
3 ZESBO - Center for Research on Musculoskeletal Systems, Faculty of Medicine, Leipzig University, Leipzig, Germany
4 Hospital St. Georg gGmbH, Clinic of Trauma, Orthopedic and Septic Surgery, Leipzig, Germany
5 Department of Orthopaedic Surgery, Traumatology and Plastic Surgery, University of Leipzig Medical Center, Leipzig, Germany
* Corresponding author: Sascha Kurz, sascha.kurz@medizin.uni-leipzig.de
† ***These authors contributed equally to this work and are co-first authors: Marc Gebhardt, Sascha Kurz***

Table of contents

Objective ... 2
Schedule ... 2
Materials ... 2
Preparation, storage and hygiene ... 2
Geometry and mass determination ... 2
Mechanical test equipment ... 3
Time dependent measurement ... 3
Methods ... 4
Test procedure ... 4
Test regime ... 6
Evaluation scheme ... 7
Biomechanical material database scheme ... 8
Structure ... 8
Key recommendation ... 8
References ... 10
Annex 1: Assessment code scheme ... 11
Annex 2: Testing protocols ... 13
Testing protocol soft tissue (s) ... 13
Testing protocol trabecular bone (t): ... 14
Testing protocol cortical bone (c): ... 15

Objective

The aim is the standardized determination of material parameters (elastic and plastic) of human tissue samples (soft tissue, cortical and trabecular bone) from the lumbopelvic complex by means of special test rigs (Axial Tension Test (ATT), Axial Compression Test (ACT) and Three Point Bending Test (TBT)).

Schedule

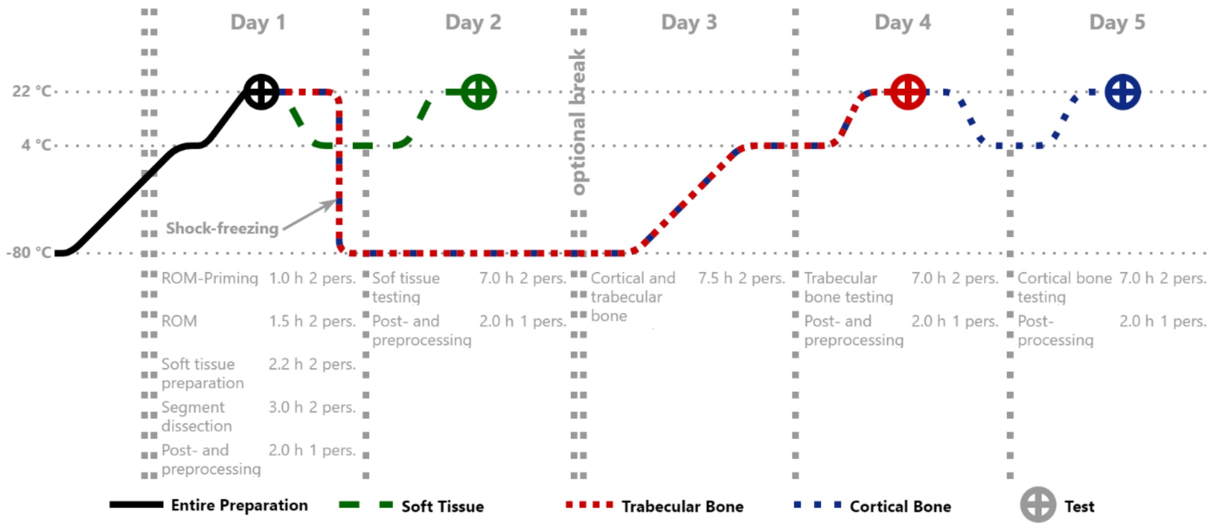

Figure 1: Flow chart with corresponding temperature curve as well as estimated time and personnel (from Kurz, S. and Gebhardt, M. et al. [1])

Materials

Preparation, storage and hygiene

- Specimen preparation and storage according Kurz, S. and Gebhardt, M. et al. [1]
- Refrigerator (approx. 4 °C)
- Preparation tray, scalpel and tweezers
- Washing bowl, paper towels, disinfectant dispenser, tarpaulins, gloves, degreaser and surface disinfection

Geometry and mass determination

- Digital caliper 150 mm (Preciva, Shenzhen, China or eq.)
- Precision scale (AT400, Mettler-Toledo, Gießen, Germany or eq.) with direction marked lightweight specimen pan

Mechanical test equipment

- Testing machine: 10 kN frame of universal testing machine (LFEM 600/100/10, walter+bai, Löhnringen Switzerland or eq.)
- Modular test setups (further information in Supplementary Material S2):
  - Axial tension test setup (ATT, drawing number PF002-004)
  - Axial compression test setup (ACT, drawing number PF003-002) with orthogonal spacer (drawing number PF010-001)
  - Three Point Flexure Test setup (TBT with 20 mm span, drawing number PF004-004) with orthogonal spacer (drawing number PF010-001)
- Additional tools:
  - Spanners and Allen keys
  - Dynamometric key (for ATT; MicroClick MC5, Proxxon Industrial, Föhren, Deutschland or eq.)

Time dependent measurement

- Conventional:
  - Measuring amplifier: QuantumX (100 Hz sampling rate, no filters; MX840B, Hottinger Brüel & Kjaer, Darmstadt, Germany or eq.)
  - Displacement: LVDT (ATT: WA20 [probe tip] / ACT and TBT: W10K [loose plunger], Hottinger Brüel & Kjaer, Darmstadt, Germany or eq.) with applicators (FDM)
  - Force: internal load cell of testing machine and additional 20 kg-load cell (S40S-G3-0020, Bosche, Damme, Germany or eq.) with load cell plates (drawing number PF010-001)
- Optical:
  - Measuring: Digital Image Correlation (DIC; 4 Hz sampling rate; Q400 DCM 12.0, Limess, Krefeld, Germany; Istra4D, Dantec Dynamics, Skovlunde, Denmark) with 50 mm lens (VS-5018H1, VS Technology, Tokyo, Japan or eq.) and spacer rings (ATT: 5 mm, ACT and TBT: 5 + 10 mm)
  - Lighting: Two area LED lights (T120, Shenzhen Neewer Technology, Shenzhen, China) at 5600 K
  - Calibration: Istra4D calibration target (ATT: 4 mm marked black plastic [4BMB BD-4.00mm-09x09-AC3], ACT and TBT: 2 mm marked white glass [GL-02-WMB_9x9] or eq.)
  - Marking:
    - Indirect: Speckle pattern with measurement marks laser printed on weather-resistant labels (AST-Speckle-Pattern_Labels_V03-2.pdf on L4775, Avery Zweckform, Oberlaindhaim, Germany or eq.)
    - Direct:
      - Opaque white priming (Eberhard Faber, Stein, Germany or eq.) with brush (GH30414, da Vinci Künstlerpinselfabrik DEFET, Nuremberg, Germany or eq.)
      - Black toner dust (2220D, Ricoh, Tokyo, Japan or eq.)
      - Pollinator can with flat filter (one layer of URMA, 1-2-3 Filter, Reinbek, Germany or eq.)
      - Draught-proof chamber
  - Evaluation: Istra4D (version 4.4.7.527, Dantec Dynamics, Skovlunde, Denmark) on 35 px grid spacing and 39 px facet size, or eq.
- Synchronization: Analog trigger (5 V batterie with switch)

Methods

Test procedure

Repetition per type of experiment:

- Assemble test setup

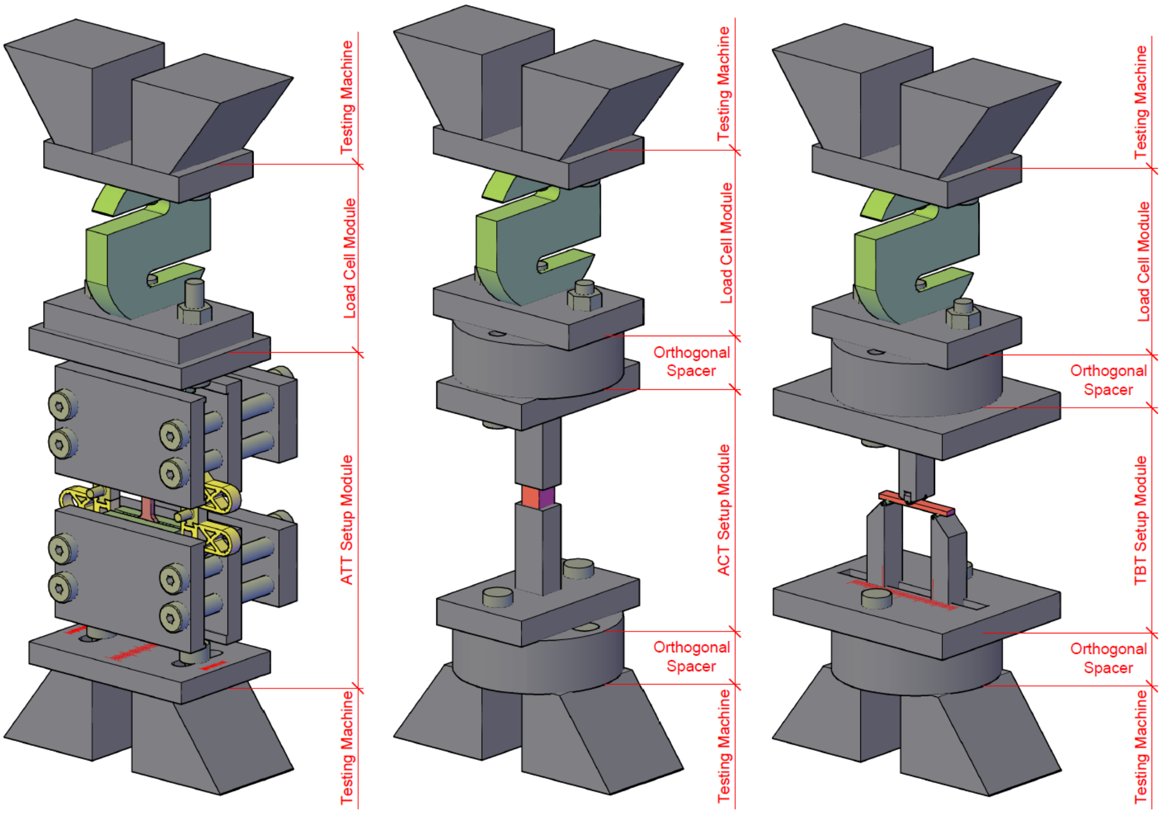

Figure 2: Assembly acc. Test setup (left: Axial tensile test (ATT), middle: Axial compression test (ACT), right: Three-point-bending test (TBT))

- Assemble DIC

Test setup	Objective distance/mm	Sensor distance/mm	Focal length/mm
**ATT**	~440	268	50+5
**ACT/TBT**	~250	164	50+10+5

- Focusing and calibration DIC
- Test recording and evaluation

- Repetition per specimen:
    - Geometry determination

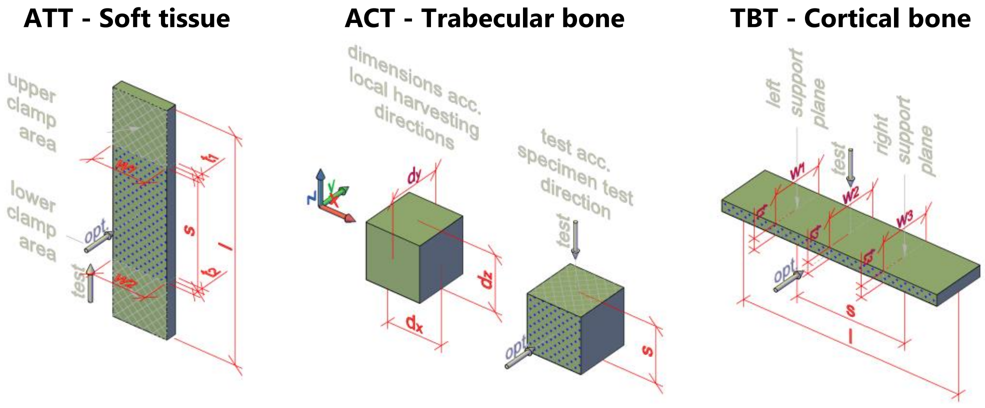

Figure 3: Geometry determination acc. test setup

    - Mass determination
    - Marking specimen:
        - Priming using brush and opaque white
        - Application of the speckle pattern by dusting with toner

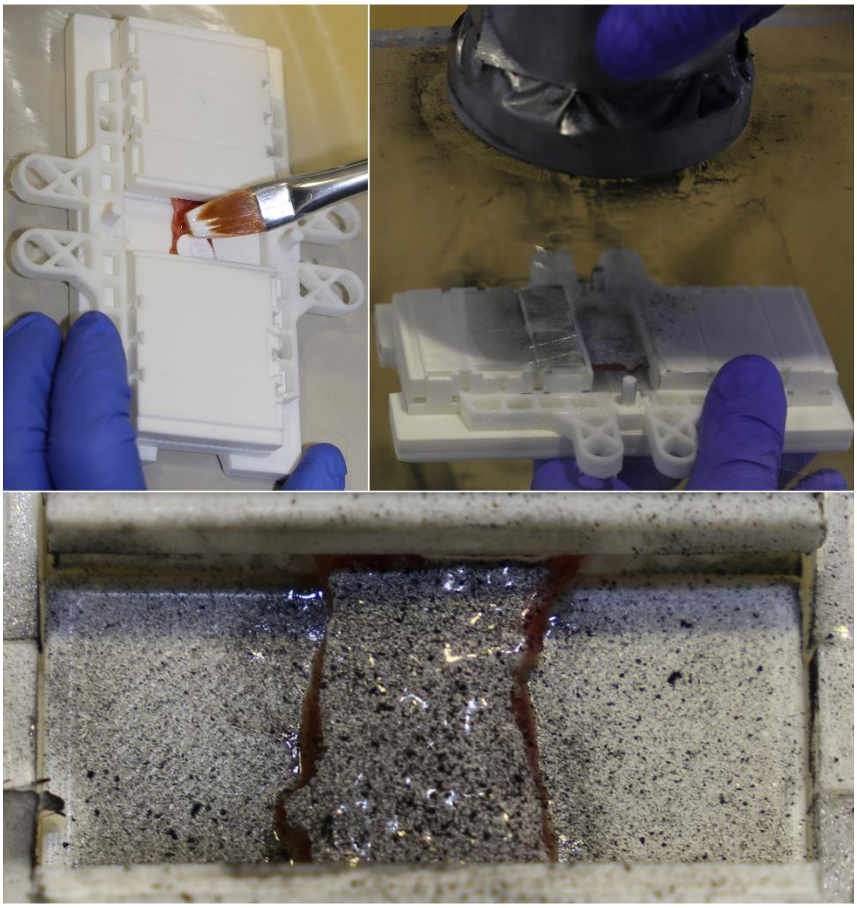

Figure 4: Exemplary speckle pattern application on soft tissue specimen for DIC

    - Installation in test environment
    - Conducting test regime
- DIC calibration for safety purpose
- Data backup

Test regime

- Axial tension test on soft tissue specimens:
    - Assembly of clamp mount with specimen
    - Removing spacer
    - Clamping with 2 Nm torque
    - Estimate Maximum load $F_{max,est} \sim \bar{A}_{CS} \cdot f_{u,est}$
    - Displacement controlled testing with $\dot{w} = \dot{\varepsilon} \cdot s = 0.010\ \mathrm{s}^{-1} \cdot 20\ \mathrm{mm} = 0.2\ \mathrm{mm/s}$
    - Strong soft tissue ($F_{max,est} \geq 100\ \mathrm{N}$):
        - Preload to $0.1 \cdot F_{max,est}$
        - 10 load cycles between 0.1 and $0.3 \cdot F_{max,est}$
    - Very strong soft tissue ($F_{max,est} \geq 200\ \mathrm{N}$): use other or build in machine load cell
    - Load until failure, or decrease of force by $0.3 \cdot F_{max}$
- Axial compression test on trabecular bone cubes:
    - Assembly of specimen
    - Displacement controlled testing with:
      $\dot{w} = \dot{\varepsilon} \cdot s$, $\dot{\varepsilon} = 0.005\ \mathrm{s}^{-1}$, $s = d_{test}$ = dimension in test direction
    - Maximum displacement (setup protection) $w_{max} = d_{test} \cdot 0.75$
    - Load until failure, or decrease of force by $0.3 \cdot F_{max}$
- Three-point bending test on cortical bone beams:
    - Assembly of specimen
    - Displacement controlled testing with:
      $\dot{w} = (\dot{\varepsilon} \cdot s^2)/(6 \cdot t)$, $\dot{\varepsilon} = 0.005\ \mathrm{s}^{-1}$, $s = 20\ \mathrm{mm}$, $t = t_2$ = thickness in midspan
    - Maximum force (setup protection) $F_{max,sec} = 250\ \mathrm{N}$
    - Load until failure, or decrease of force by $0.3 \cdot F_{max}$

Evaluation scheme

The evaluation should be performed with a high degree of automation. This makes it possible to analyze all specimens using the same evaluation scheme, which leads to better comparability and reproducibility. For this purpose, self-written routines were used, utilizing the language Python (3.7.10), as well as the modules Numpy (1.20.3), Scipy (1.6.2) and Pandas (1.3.0). Matplotlib (3.3.4) and Seaborn (0.11.1) were used to plot diagrams.

The evaluation scheme applied here is displayed in Figure 5. It starts with importing the protocol data and the paths of the measurements, as well as options. The protocol data contains all relevant information, such as key, geometry and assessment codes. In the following, the relevant cross-section values were determined depending on the type of test and the measurement data were read in. If optical measurement data were reliably determined, they were synchronized with the conventional data via the displacement of the load application point. The 100 Hz conventionally measured data was then downsampled to a frequency of 4 Hz with smoothing via the moving average. Without optical data, downsampling to 10 Hz was applied.

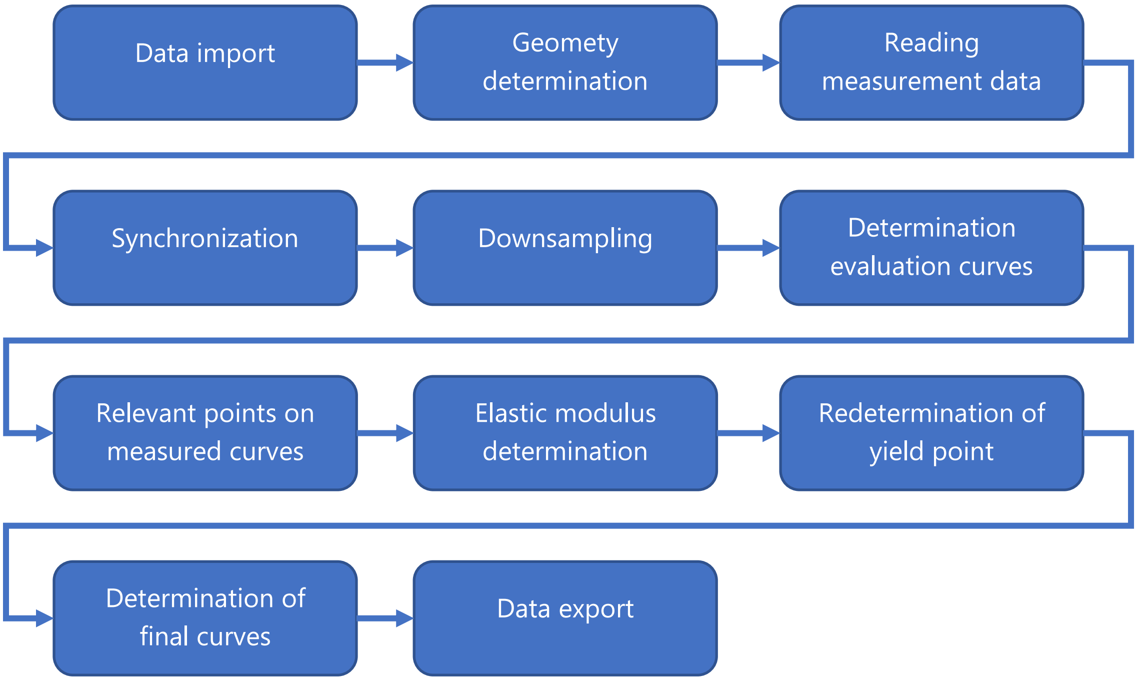

Figure 5: Evaluation scheme

The force values remain untouched since a possible preload cannot be excluded in principle. One exception is the compensation of the spring force, if LVDTs were used with probe tip. Then, the evaluation curves (stress/strain) are determined from the measured curves and specimens' geometry. The relevant evaluation points are now determined on the basis of these curves. For the determination of the curve section, that can be accepted as linear for the determination of the modulus of elasticity, a modification of the approach according to Keuerleber [2], due to the high variance, proved to be more meaningful than selecting a fixed area in the stress or strain space as usual. Here, the maximum of the difference quotient of the stress-strain function is determined. The limit of the linear range was then set at 75 % of this value.

In the default case, the modulus of elasticity was determined from conventional measurements using least squares fitting. A special case here is the evaluation of three-point bending tests, since here the influence of a cross-section that varies over the test length cannot be neglected. A more detailed consideration of this problem was given in an additional publication [3]. The method used here to determine the elastic modulus is the "D2Mgwt" method proposed there as the preferred variant. This is a global adjustment of the measured elastic curve with the theoretical one, eliminating falsifying influences such as support indentation and shear force deformation.

Before exporting the data, the strain offset was determined up to the point of intersection with the elastic modulus. The start of the stress-strain curve was then linearized to the lower limit of the above-mentioned Keuerleber range. In this way, it was also possible to exclude initial effects for the non-optically determined values.

Biomechanical material database scheme

Structure

The concept envisages a database with several sub-databases, which can be linked via unique keys. A draft of this approach is shown in Figure 6. The core of this is the biomechanical material database itself, which is partly fed by background data. On the one hand, this should be a donor database that contains all relevant donor data. Particular attention must be paid here to data protection. This means that only data that does not allow any conclusions to be drawn about the identity of the donor should be directly accessible. For this purpose, special user accounts must be introduced that restrict access to and processing of the original data. Furthermore, a database appears to be useful, which allows conclusions to be drawn about the harvesting and storage procedure. This should be nested to allow complete documentation from the level of the anatomical region under consideration to the specimen itself, linked to the associated preparation procedure. Finally, the test procedure should also be linked in a database in order to enable the traceability of the procedure used. Furthermore, it would be beneficial if the original measurement data could be linked and made available to facilitate review, reproduction and reuse. In order to save memory and transfer resources, a shortened version should be available in which these data are omitted.

Key recommendation

{ORCID}_{Preparation Location Key}_{Donor ID}_{Anatomical Region Key}_{Specimen Key}

Example:

- Executer: Marc Gebhardt – ORCID=0000-0001-8378-3108
- Preparation location: Leipzig-University Leipzig-Anatomy,
- local donor ID: 67-17
- Anatomical region Lumbopelvic complex
- Specimen type: cortical bone specimen
- Origin: Ramus superior ossis pubis, right, proximal

- **Resulting key**: 0000-0001-8378-3108_LEIULANA_67-17_LuPeCo_cr41a

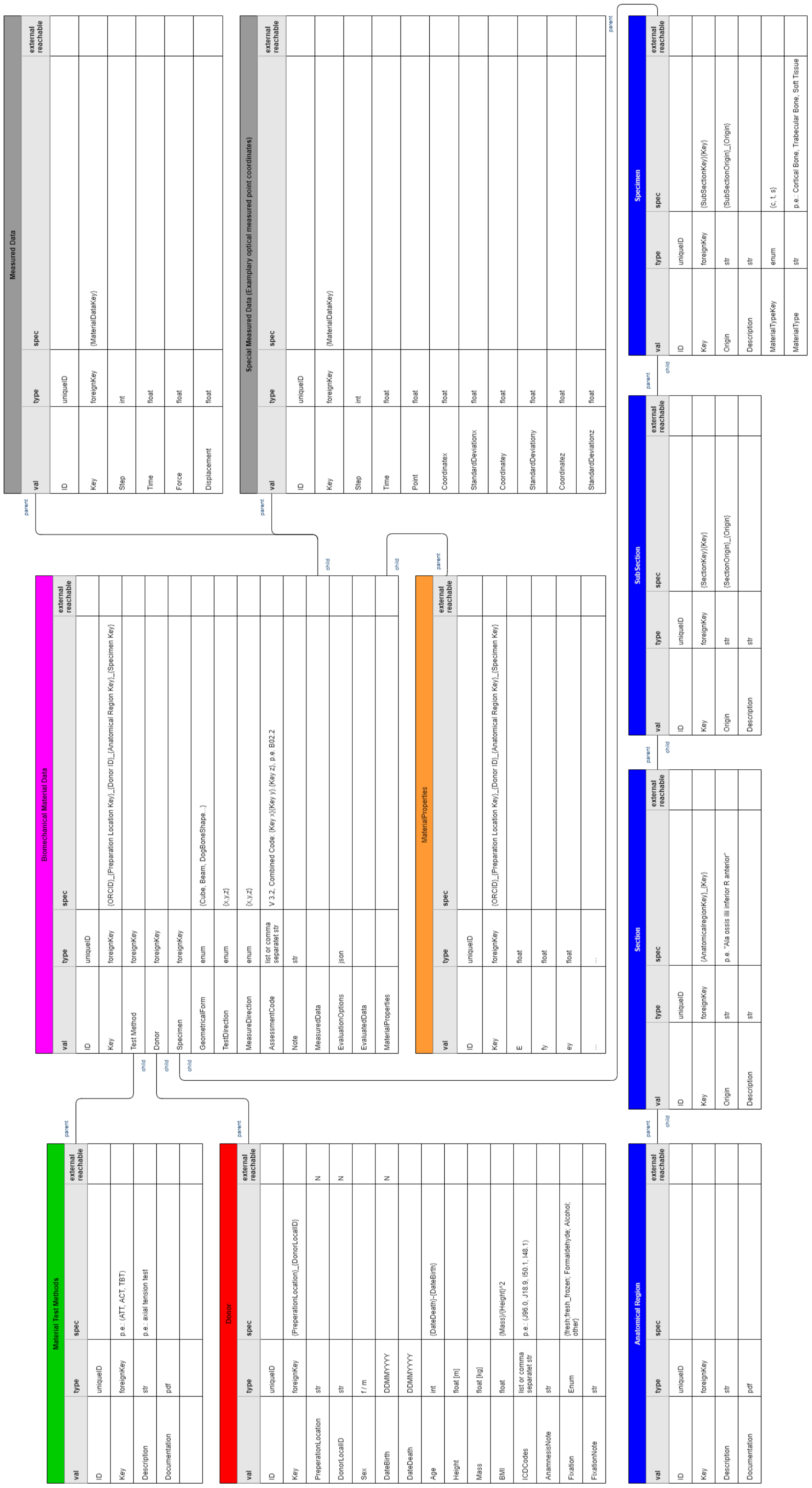

Figure 6: Biomechanical material database scheme

References

1. Kurz S, Gebhardt M, Grundmann F, Heyde C-E, Steinke H. Approach to standardized material characterization of the human lumbopelvic system-Specification, preparation and storage. PLOS ONE. 2023; 18:e0289482. Epub 2023/08/03. doi: 10.1371/journal.pone.0289482 PMID: 37535581.
2. Keuerleber M. Bestimmung des Elastizitätsmoduls von Kunststoffen bei hohen Dehnraten am Beispiel von PP. Von der Fakultät Maschinenbau der Universität Stuttgart zur Erlangung der Würde eines Doktor-Ingenieurs (Dr.-Ing.) genehmigte Abhandlung. Doktorarbeit, Universität Stuttgart. 25.08.2006. Available from: https://elib.uni-stuttgart.de/bitstream/11682/1717/1/Dissertation_Endversion.pdf.
3. Gebhardt M, Steinke H, Slowik V. Determination of the Modulus of Elasticity by Bending Tests of Specimens with Nonuniform Cross Section. Exp Mech. 2023; 63:743–58. doi: 10.1007/s11340-023-00945-y.

Annex 1: Assessment code scheme

This biomechanical assessment scheme, first published within the supporting information of Kurz, S. and Gebhardt, M. et al. [1], was developed to document any deviations or peculiarities within the entire procedure. It allows the evaluation of individual specimens concerning different procedure steps, assessment types and the strength of their occurrence. Thus, errors in the procedure in general as well as the reliability of the results can be quantified based on defined criteria and evaluated retrospectively. Table 1 shows this very assessment scheme.

Table 1: Biomechanical-assessment-code-scheme (Version 3.2, acc. to [1])

<table>
<tr><th colspan="3">Procedure Step</th><th colspan="4">Assessment Type</th><th colspan="2">Strength</th></tr>
<tr><th></th><th>Description</th><th>Key x</th><th>Description</th><th>Key y</th><th>Description</th><th>Key y</th><th>Description</th><th>Key z</th></tr>
<tr><td rowspan="3">Acqui-sition</td><td>Pre-dissection</td><td>A</td><td>Abnormal</td><td>00</td><td>Mass</td><td>11</td><td>Not</td><td>0</td></tr>
<tr><td>Dissection</td><td>B</td><td>Absence</td><td>01</td><td>Moisture</td><td>12</td><td>Slight</td><td>1</td></tr>
<tr><td>Storage</td><td>C</td><td>Damaged</td><td>02</td><td>Designation</td><td>13</td><td>Medium</td><td>2</td></tr>
<tr><td rowspan="2">Test</td><td>Testing</td><td>D</td><td>Deflection</td><td>03</td><td>Optical</td><td>14</td><td>Complete</td><td>3</td></tr>
<tr><td>Measurement</td><td>E</td><td>Density</td><td>04</td><td>Orientation</td><td>15</td><td colspan="2" rowspan="7"></td></tr>
<tr><td rowspan="2">Evalu-ation</td><td>Input</td><td>F</td><td>Displacement</td><td>05</td><td>Scheme</td><td>16</td></tr>
<tr><td>Statistics</td><td>G</td><td>Force</td><td>06</td><td>Temperature</td><td>17</td></tr>
<tr><td colspan="3" rowspan="4">Combined Code:
{Key x}{Key y}.{Key z}</td><td>Geometrical</td><td>07</td><td>Timing</td><td>18</td></tr>
<tr><td>Histological</td><td>08</td><td>Strain</td><td>19</td></tr>
<tr><td>Impure</td><td>09</td><td rowspan="2">…</td><td rowspan="2">…</td></tr>
<tr><td>Localization</td><td>10</td></tr>
</table>

The above-shown scheme leads to a combined code composed out of three partial keys. The first key describes the procedural step in which the assessment takes place; e.g. if the assessment is performed during the specimen dissection, *Key x = B*. The second key indicates the type of assessment, e.g. a damaged specimen is characterized by *Key y = 02*. Key three indicates the strength of the occurrence. For Example, a medium damaged specimen will lead to *Key y.Key z = 02.2*. Thus, the combined assessment code for this example would be *B02.2*. Other examples of coding an assessment are shown in Table 2.

Table 2: Application examples of the biomechanical-assessment-code (Version 3.2) generation

**Assessment**	**Biomechanical-assessment-code**
Specimen severely damaged during preliminary preparation and therefore not suitable for harvesting	A02.3, B01.3
Melanoma within trabecular bone specimen location, completely damaged during dissection	A00.3, B02.3
Specimen perhaps rotated	B15.2
Specimen stored outside the box, probably dried out	C12.2
Touching of test device after maximum loading	D03.1
Touching of test device before maximum loading	D03.2
Slippage of the specimen during the test	D03.3
Aborting the test by reaching the force limit	D06.3
Medium LVDT (linear variable displacement transformer) error	E05.2
Optical displacement measuring inaccurate due to low lighting	E14.2
Corrupted measurement data	F02.3
Remarkable deviation of the material data in contrast to comparable samples	G00.2

Annex 2: Testing protocols

Testing protocol soft tissue (s)

Donor:

Date:

T/°C:

RH/%:

No.	ID	Geometry/mm					Mass/g	$F_{max,det}$/N	Notes
		t_1	t_2	w_1	w_2	l			
01	sl01								
02	sr01								
03	sl02								
04	sr02								
05	sl03								
06	sr03								
07	sl04								
08	sr04								
09	sl05								
10	sr05								
11	sl06								
12	sr06								
13	sl07								
14	sr07								
15	sl08								
16	sr08								
17	sl09								
18	sr09								
19	sl10								
20	sr10								
21	sl11								
22	sr11								
23	sl12								
24	sr12								
25	sl13								
26	sr13								
27									
28									
29									
30									

Testing protocol trabecular bone (t):

No.	ID	Geometry/mm			Mass/g	test-dir.	opt.-dir.	Donor:	Date:
								T/°C:	RH/%:
		d_x	d_y	d_z				Notes	
01	tr21x					x	y		
02	tr21y					y	z		
03	tr21z					z	x		
04	tr22x					x	y		
05	tr22y					y	z		
06	tr22z					z	x		
07	tl21x					x	y		
08	tl21y					y	z		
09	tl21z					z	x		
10	tl22x					x	y		
11	tl22y					y	z		
12	tl22z					z	x		
13	tr31x					x	y		
14	tr31y					y	z		
15	tr31z					z	x		
16	tr32x					x	y		
17	tr32y					y	z		
18	tr32z					z	x		
19	tl31x					x	y		
20	tl31y					y	z		
21	tl31z					z	x		
22	tl32x					x	y		
23	tl32y					y	z		
24	tl32z					z	x		
25	tm21x					x	y		
26	tm21y					y	z		
27	tm21z					z	x		
28	tm31x					x	y		
29	tm31y					y	z		
30	tm31z					z	x		
31	tm41x					x	y		
32	tm41y					y	z		
33	tm41z					z	x		

Testing protocol cortical bone (c):

No.	ID	Geometry/mm							Mass/g	Donor:	Date:
										T/°C:	RH/%:
		t_1	t_2	t_3	w_1	w_2	w_3	l		Notes	
01	cr11a										
02	cr11b										
03	cr12a										
04	cr12b										
05	cr13a										
06	cr13b										
07	cl11a										
08	cl11b										
09	cl12a										
10	cl12b										
11	cl13a										
12	cl13b										
13	cr21a										
14	cr21b										
15	cr22b										
16	cl21a										
17	cl21b										
18	cl22b										
19	cr31a										
20	cr32b										
21	cl31a										
22	cl32b										
23	cr41a										
24	cr41b										
25	cl41a										
26	cl41b										
27	cr51a										
28	cr51b										
29	cl51a										
30	cl51b										
31	cm21a										
32	cm31a										
