# Supplementary material for: Approach to Standardized Material Characterization of the Human Lumbopelvic System: Testing and Evaluation
Source: Bioengineering (Basel). 2025 Aug 11;12(8):862. doi: 10.3390/bioengineering12080862 (PMC12383908; doi:10.3390/bioengineering12080862)
Supplement: Supplementary file 1 [file bioengineering-12-00862-s001.zip › File S4 Supporting results.pdf]

Supporting information S4 - Supporting results

Approach to standardized material characterization of the human lumbopelvic system – testing and evaluation

Marc Gebhardt [1,2,†], **Sascha Kurz** [3,*,†], Fanny Grundmann [4], Thomas Klink [1], Volker Slowik [1], Christoph-Eckhard Heyde [3,5], Hanno Steinke [2]

[1] Institute of Experimental Mechanics, Faculty of Civil Engineering, Leipzig University of Applied Sciences, Leipzig, Germany
[2] Institute of Anatomy, Faculty of Medicine, Leipzig University, Leipzig, Germany
[3] ZESBO - Center for Research on Musculoskeletal Systems, Faculty of Medicine, Leipzig University, Leipzig, Germany
[4] Hospital St. Georg gGmbH, Clinic of Trauma, Orthopedic and Septic Surgery, Leipzig, Germany
[5] Department of Orthopaedic Surgery, Traumatology and Plastic Surgery, University of Leipzig Medical Center, Leipzig, Germany
[*] Corresponding author: Sascha Kurz, sascha.kurz@medizin.uni-leipzig.de
[†] ***These authors contributed equally to this work and are co-first authors: Marc Gebhardt, Sascha Kurz***

Table of contents

1 Cortical bone 2
1.1 Assessment code representations 2
1.2 Exemplary test data visualization 3
1.3 Additional evaluation results 4
1.4 Correlations 11
1.5 Regressions 13
2 Trabecular bone 14
2.1 Assessment code representations 14
2.2 Exemplary test data visualization 15
2.3 Additional evaluation results 17
2.4 Correlations 24
2.5 Regressions 26
3 Soft tissue 27
3.1 Assessment code representations 27
3.2 Exemplary test data visualization 28
3.3 Additional evaluation results 31
3.4 Correlations 37
3.5 Regressions 39

1 Cortical bone

1.1 Assessment code representations

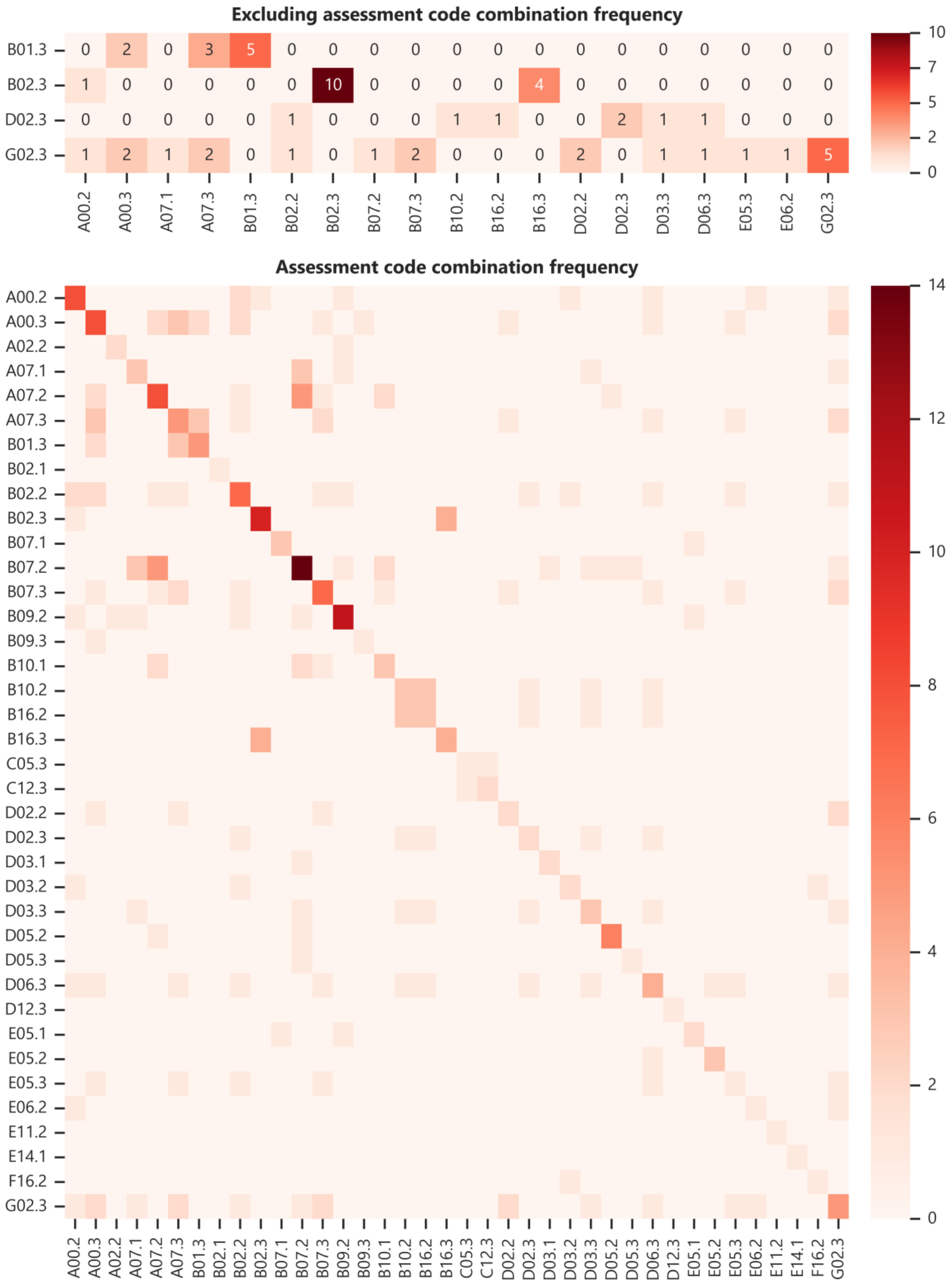

Fig. 1: Assessment code combination frequency for cortical bone specimens (upper: excluding code combinations, lower: all combinations)

1.2 Exemplary test data visualization

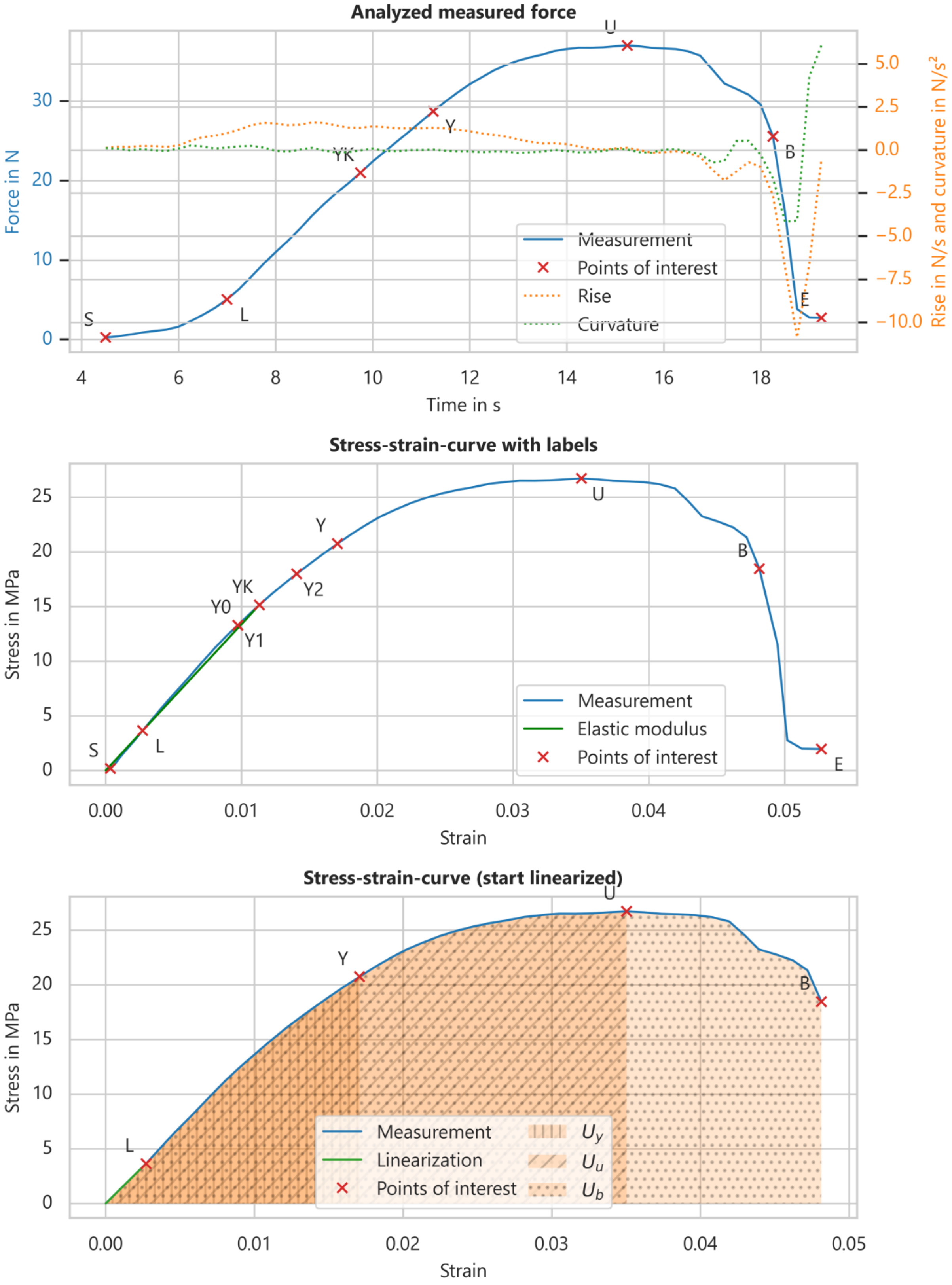

Fig. 2: Exemplary test data visualization of cortical bone specimen 60-17 cl32b (from top to bottom: measured force with curve analyzation, stress-strain-curve and stress-strain curve with representation of strain energy)

1.3 Additional evaluation results

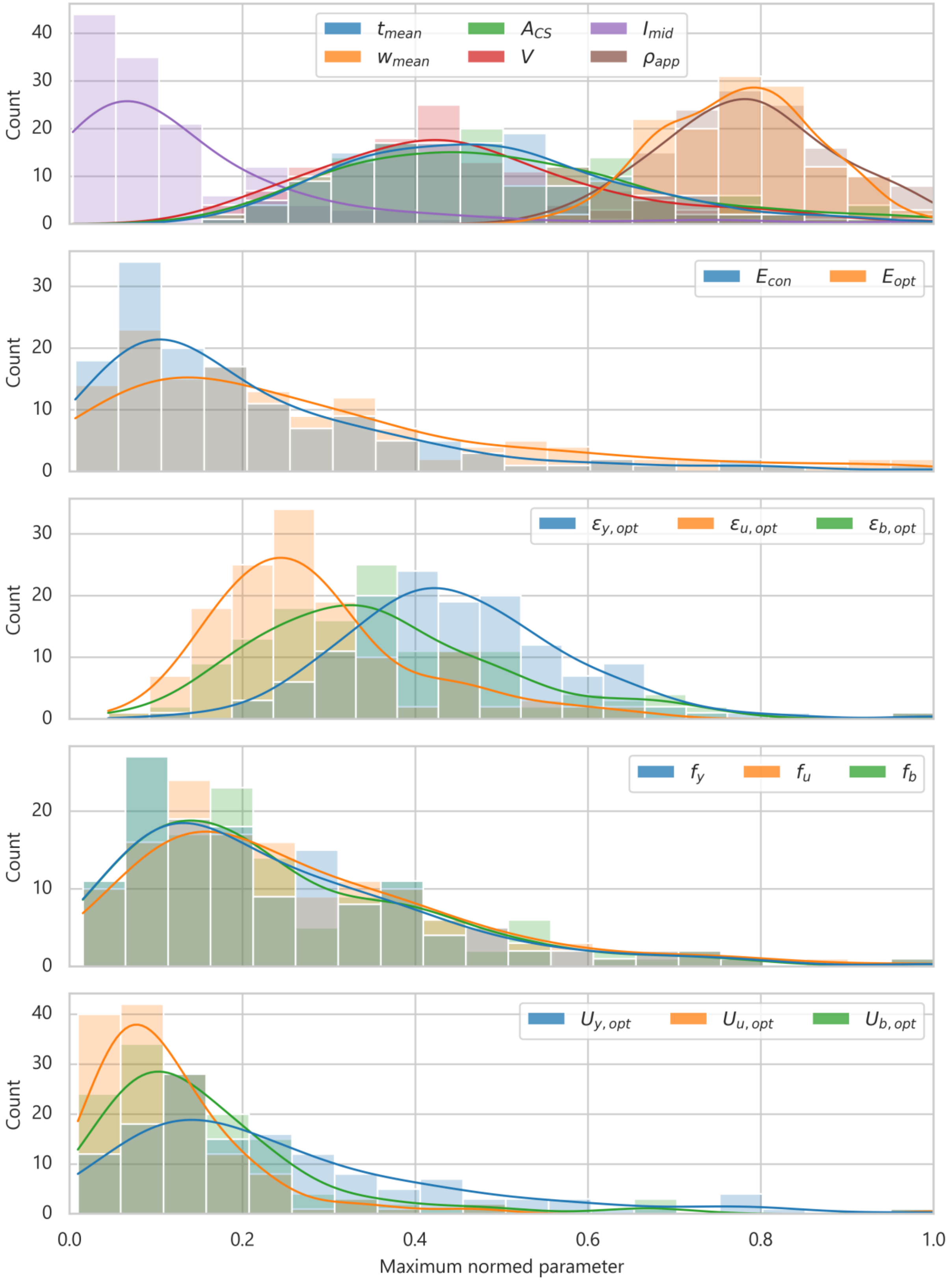

Fig. 3: Distributions of selected material parameters for cortical bone specimens

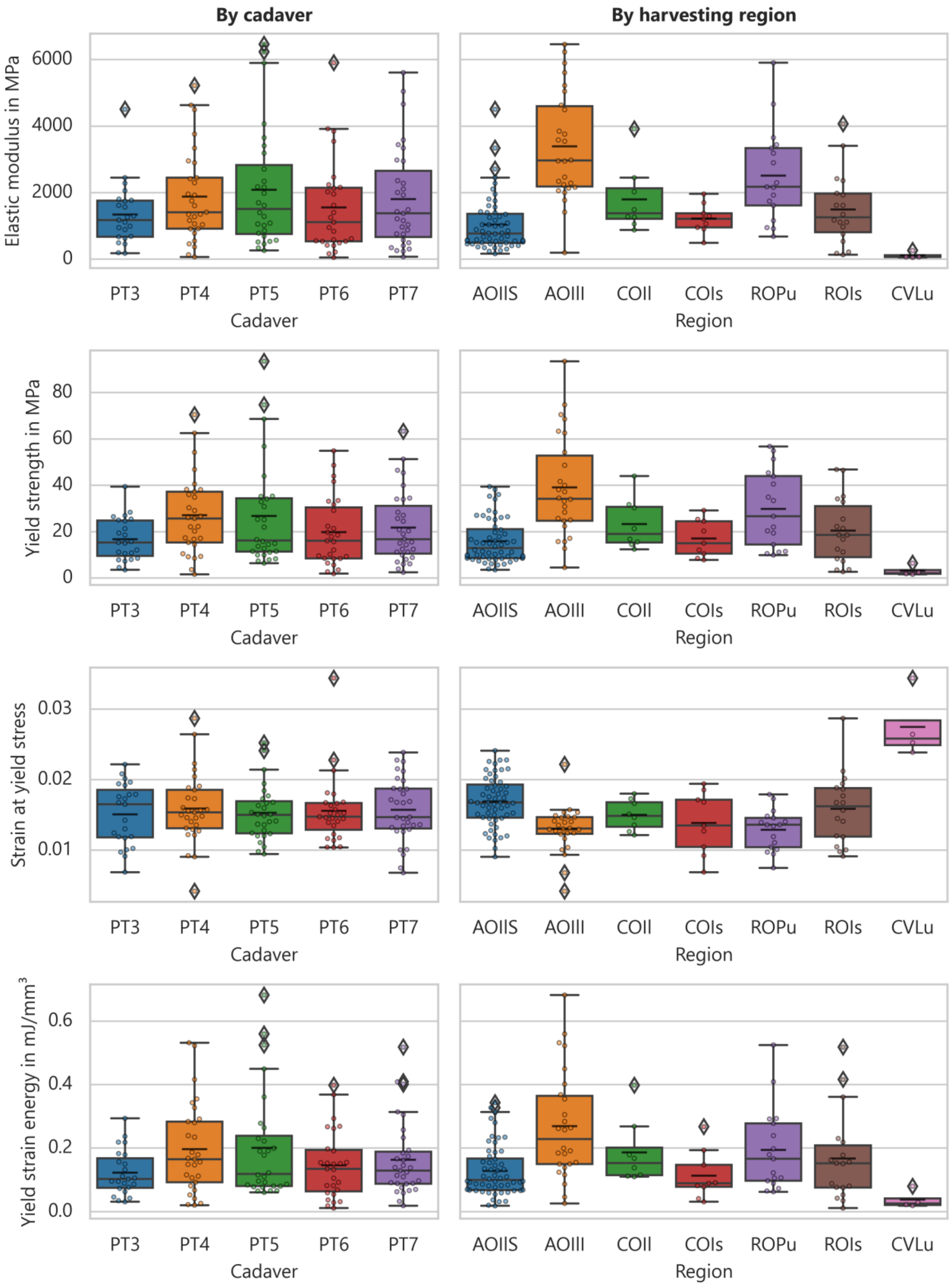

Fig. 4: Overview of selected elastic limit related material parameters for cortical bone specimens related to cadaver (left) and harvesting region (right)

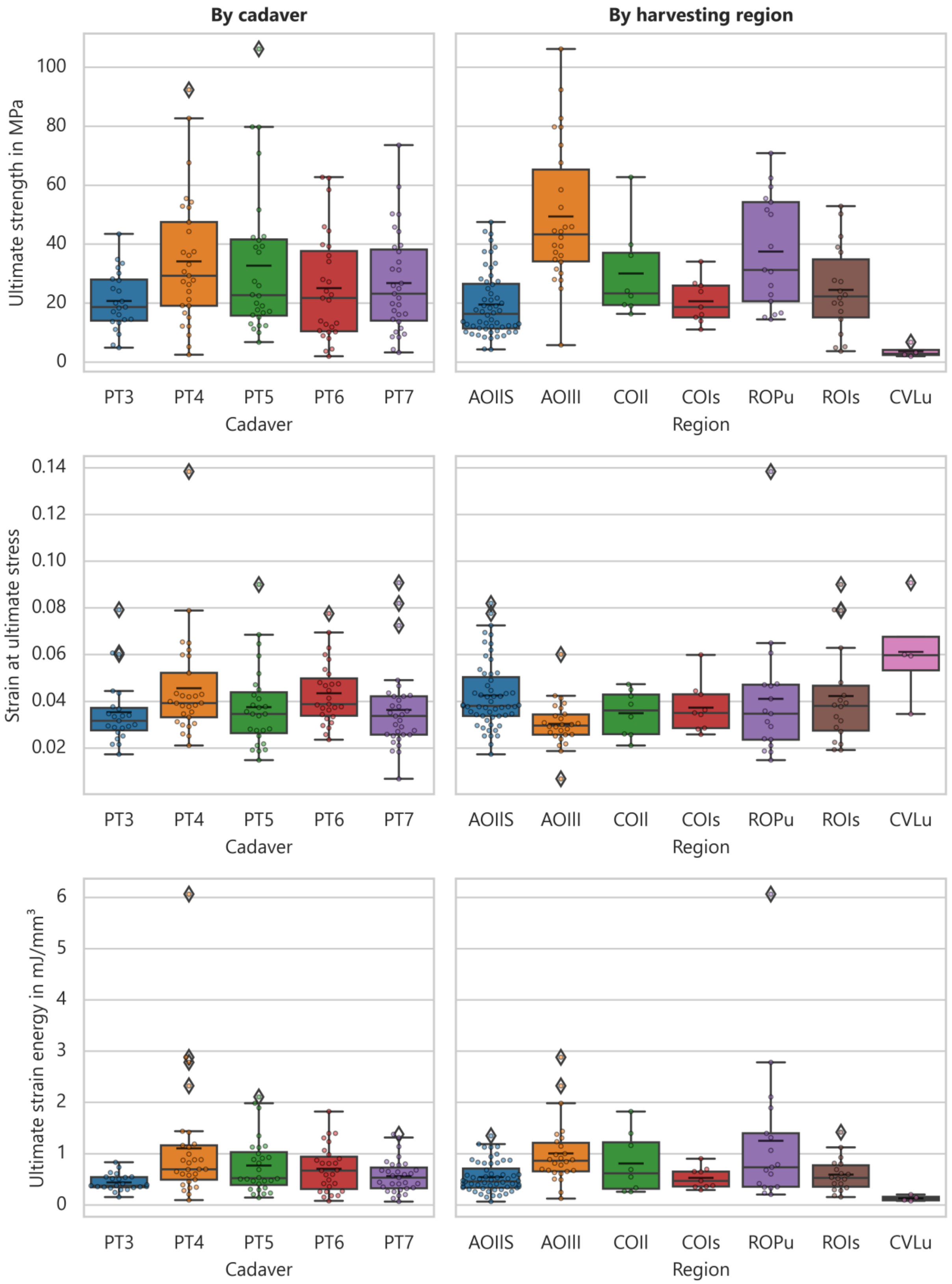

Fig. 5: Overview of selected ultimate limit related material parameters for cortical bone specimens related to cadaver (left) and harvesting region (right)

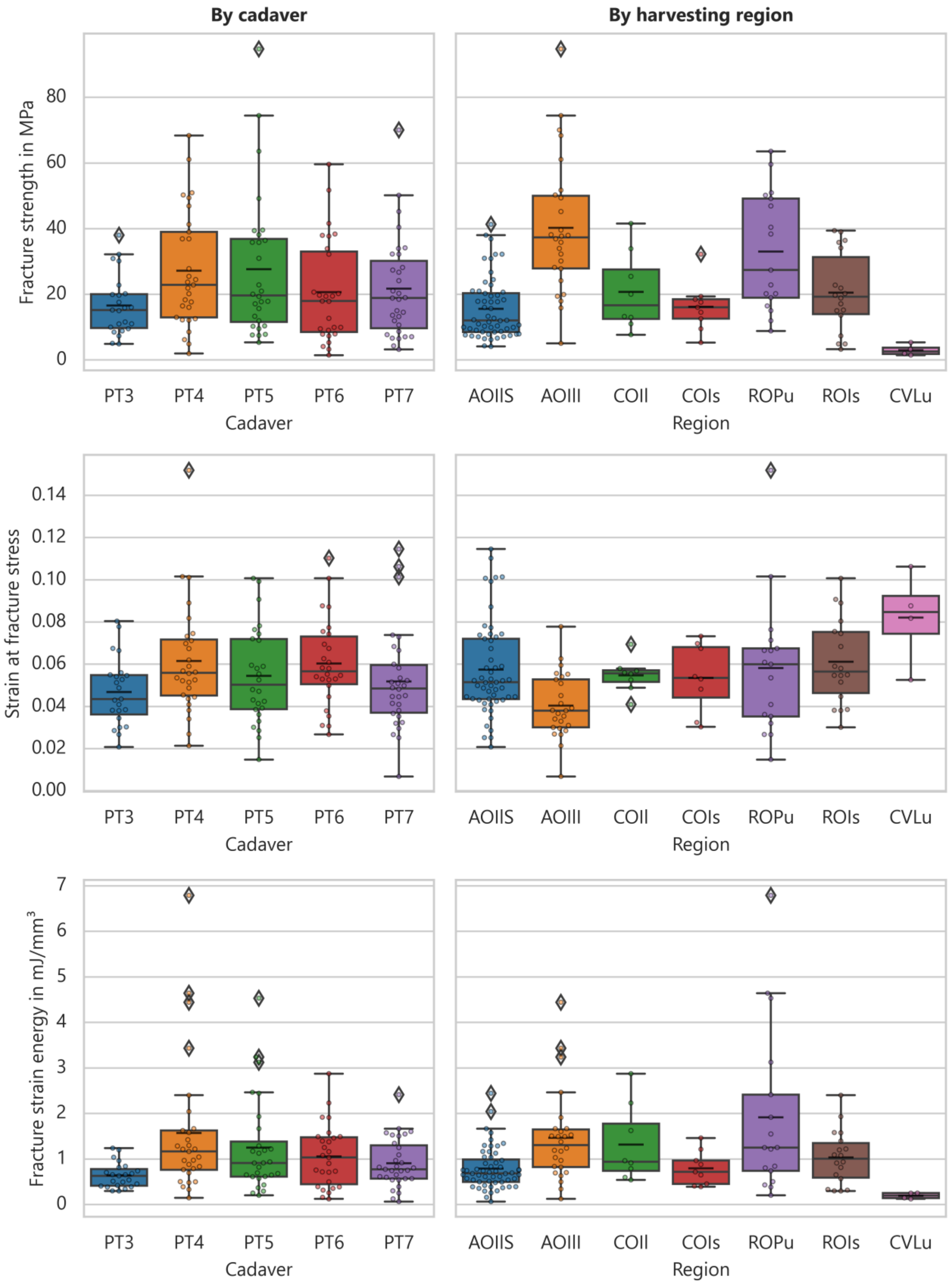

Fig. 6: Overview of selected fracture limit related material parameters for cortical bone specimens related to cadaver (left) and harvesting region (right)

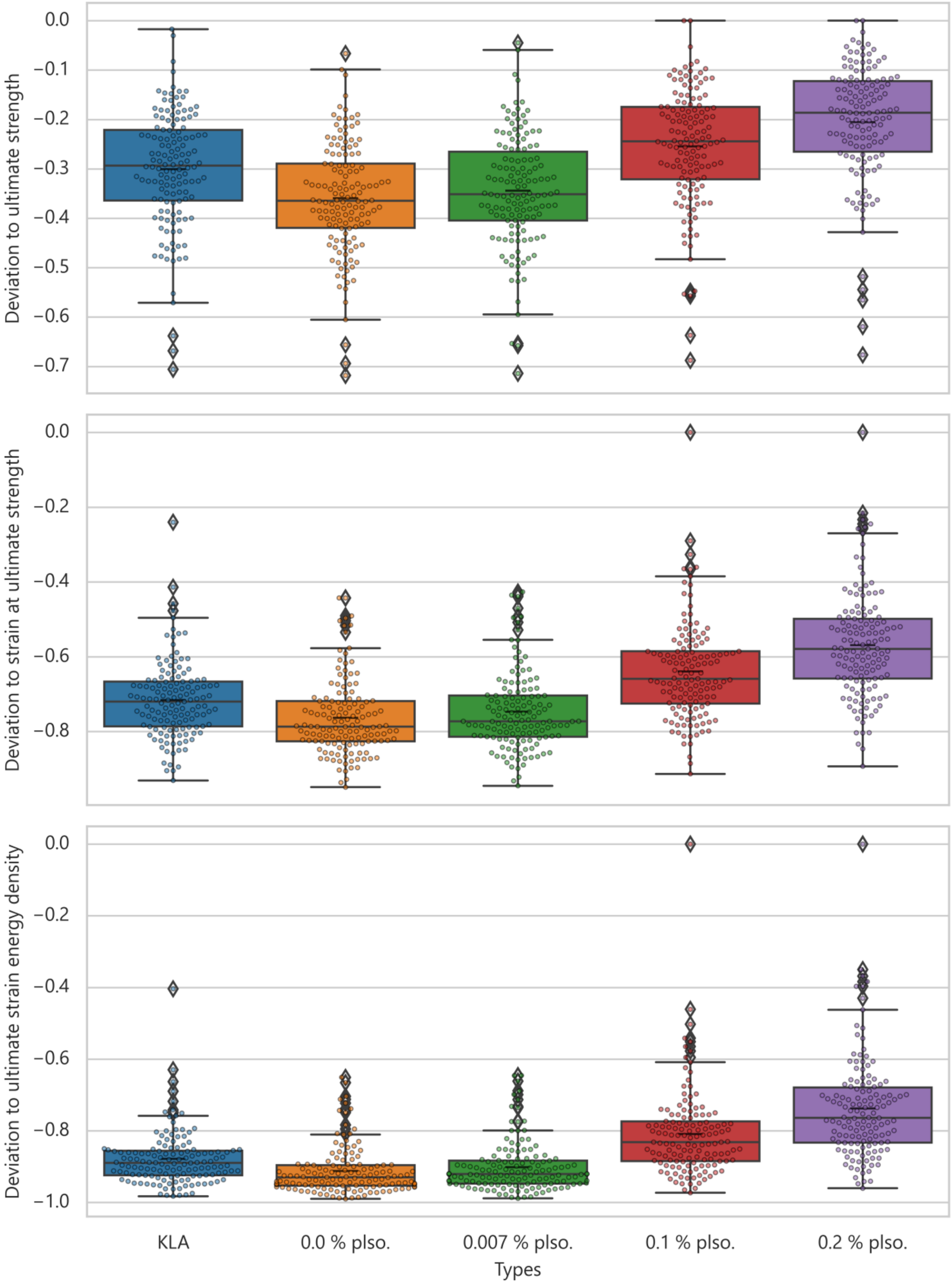

Fig. 7: Overview of selected elastic limit related material parameters for cortical bone specimens related to different yield point determinations (KLA: Keuerleber approach, plso.: plastic strain offset)

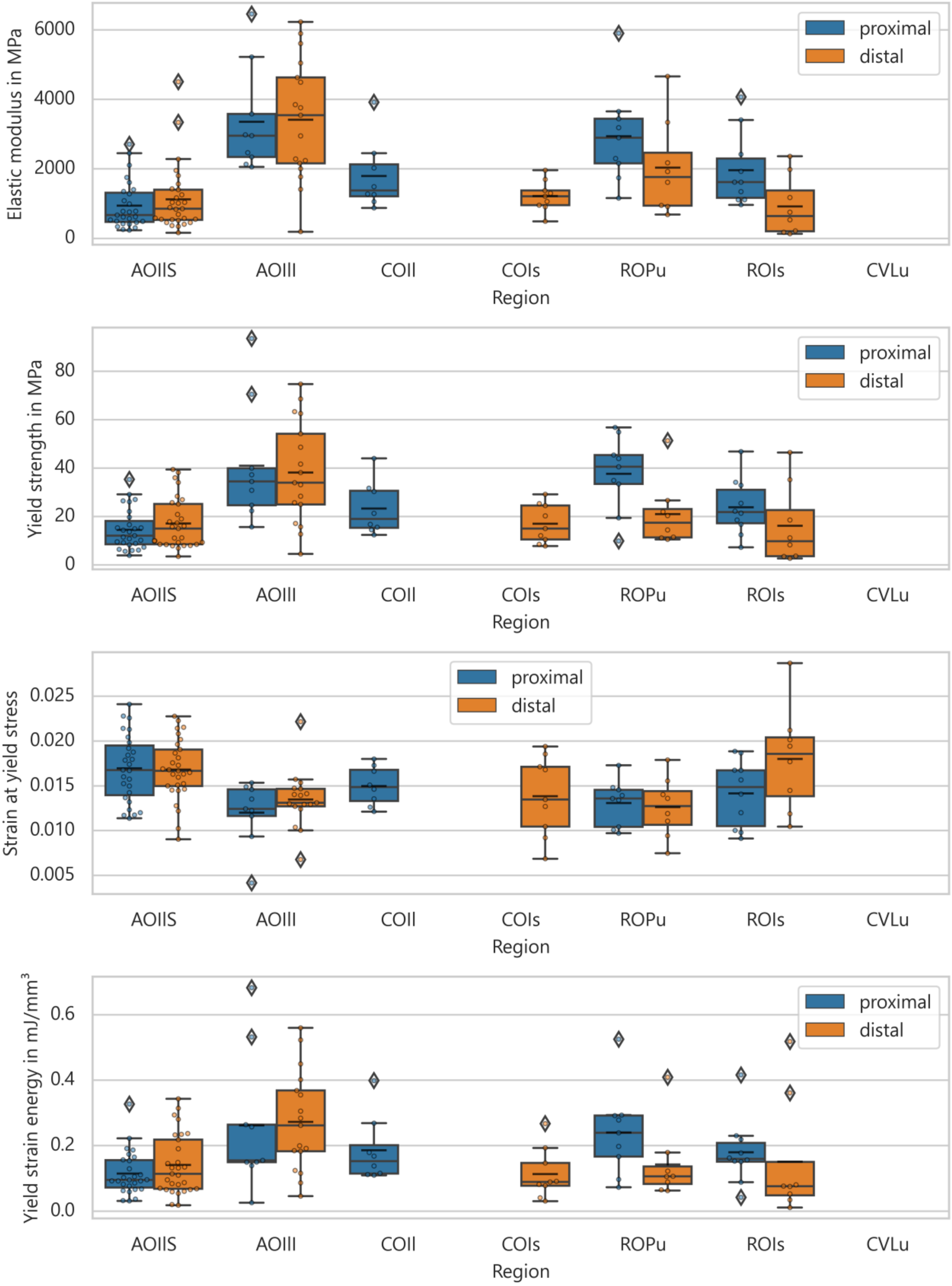

Fig. 8: Overview of selected elastic limit related material parameters for cortical bone specimens related to harvesting region and side

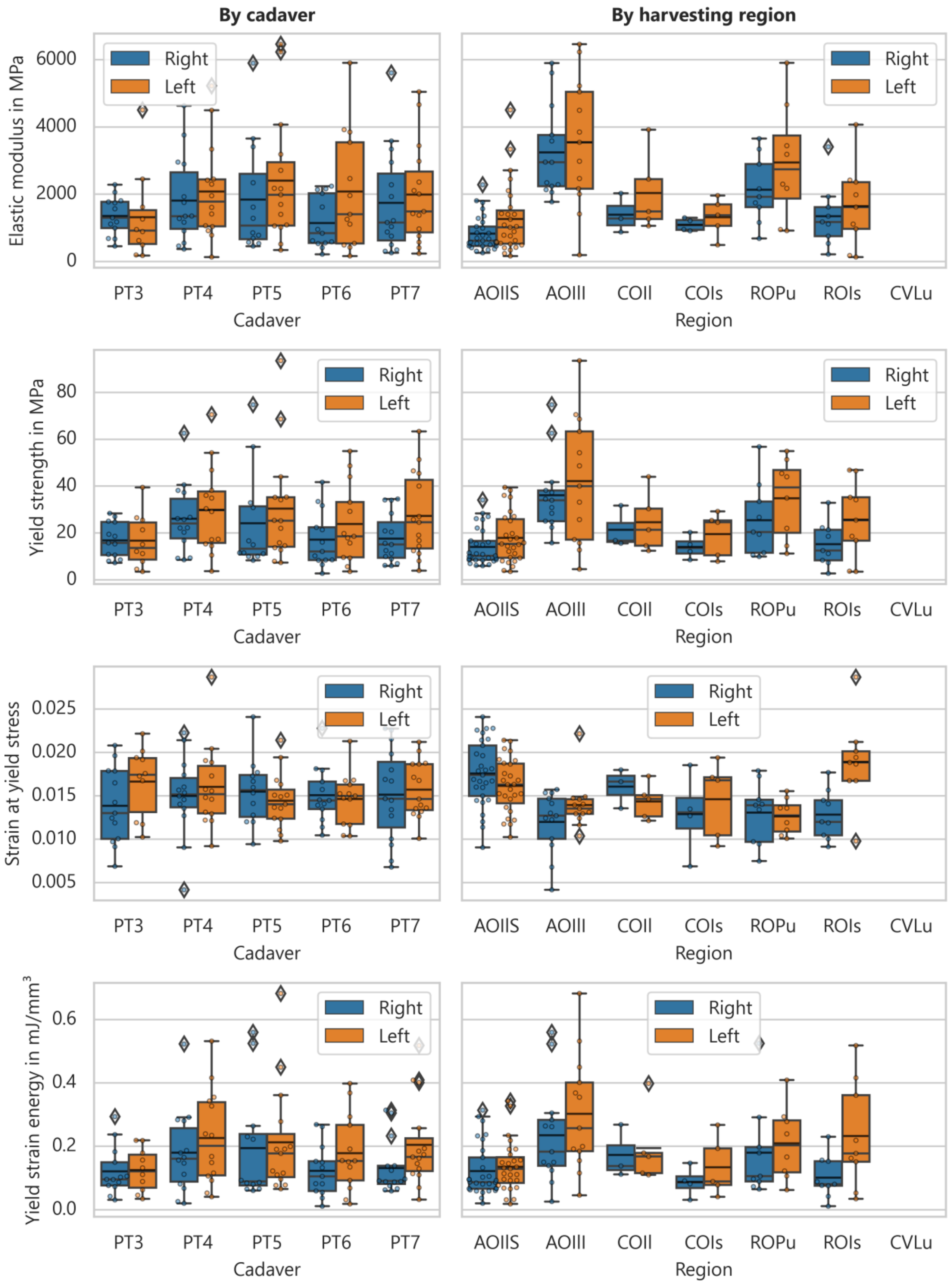

Fig. 9: Overview of selected elastic limit related material parameters for cortical bone specimens related to cadaver (left) and harvesting region (right) as well as body side

1.4 Correlations

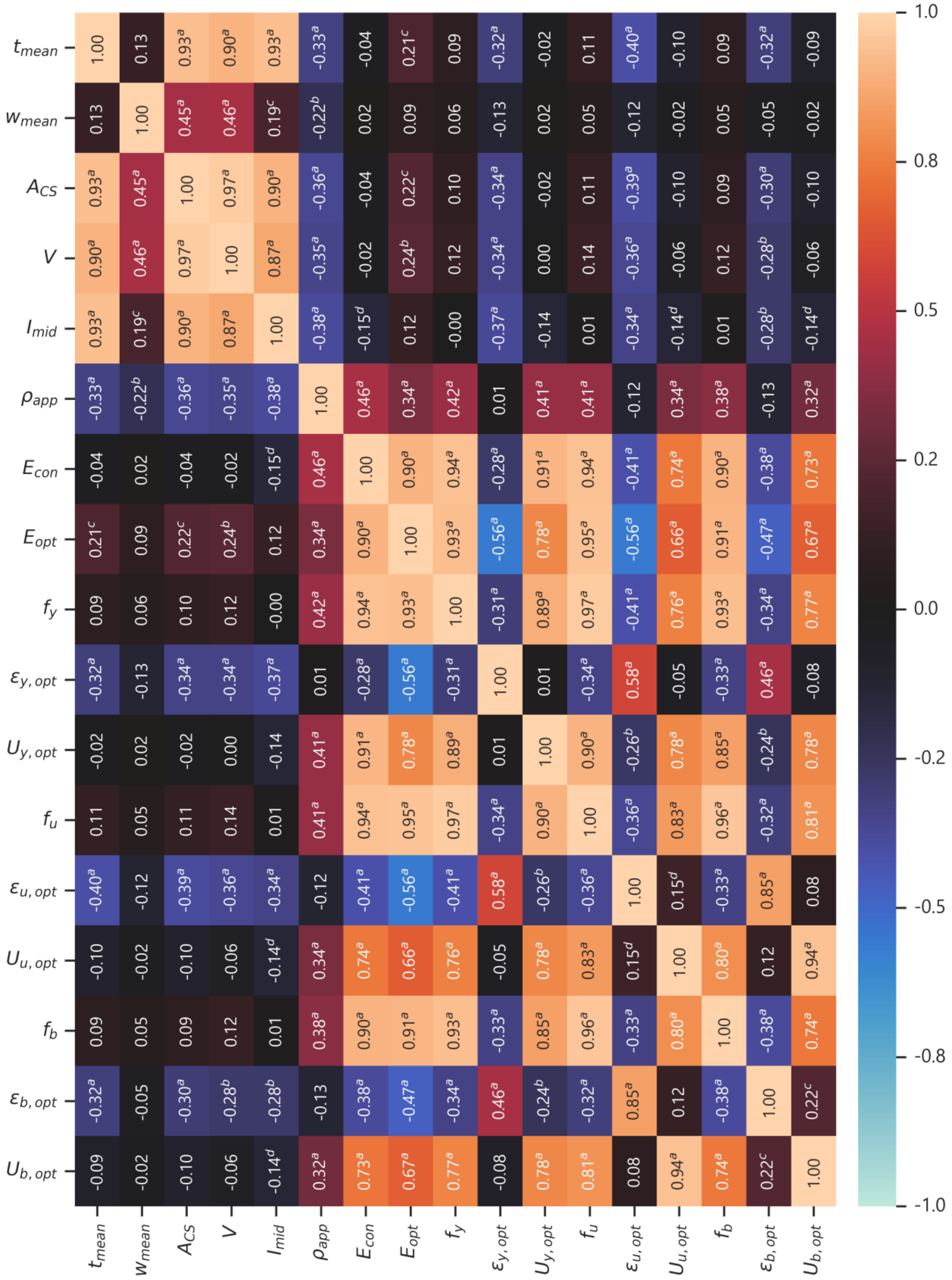

Fig. 10: Spearman correlation coefficients for selected parameters of cortical bone specimens (associated significance level labeled: a ≤0.001, b ≤0.01, c ≤0.05 and d ≤0.1)

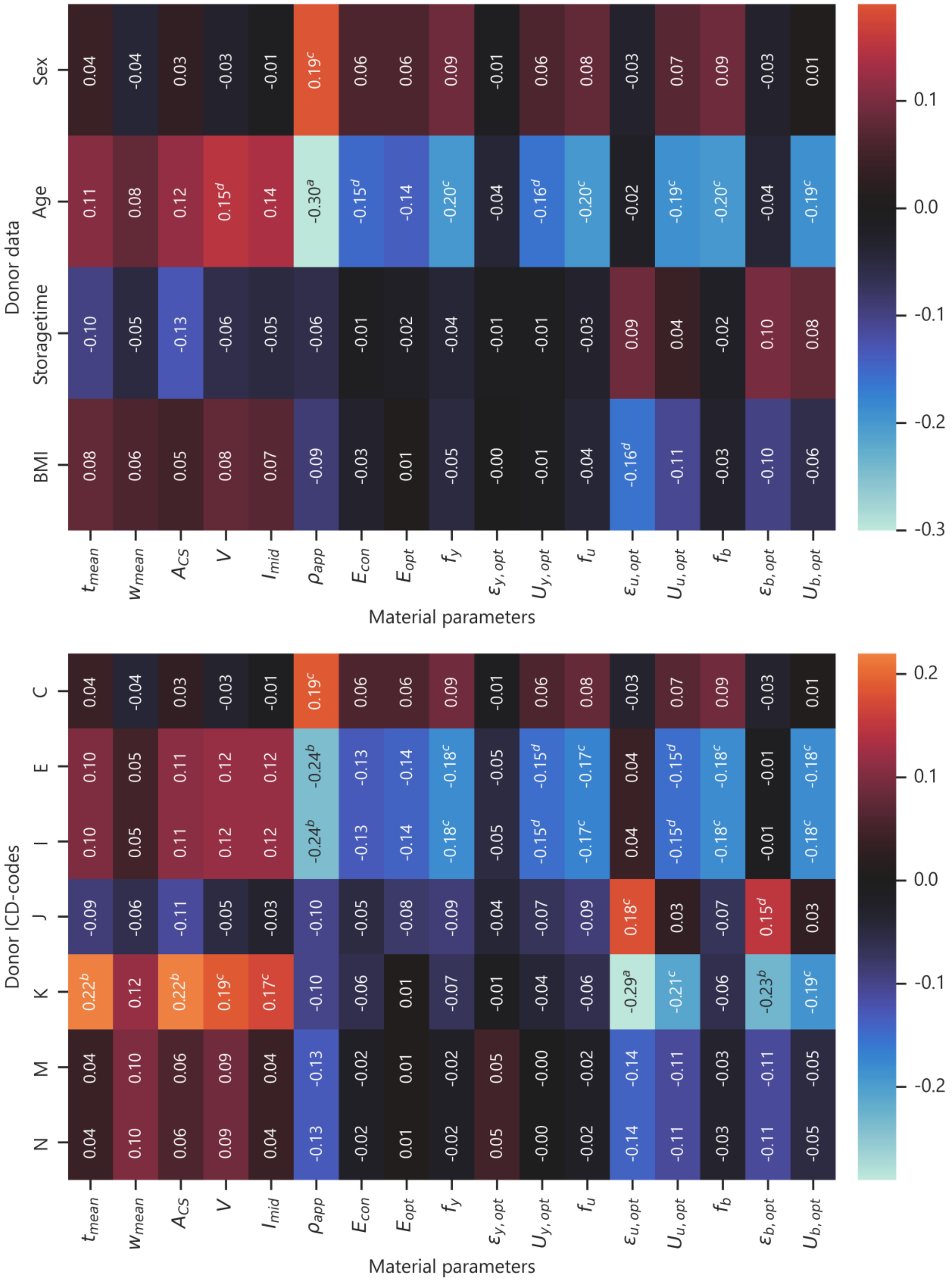

Fig. 11: Spearman correlation coefficients for selected parameters to donor-related data of cortical bone specimens (associated significance level labeled: a ≤0.001, b ≤0.01, c ≤0.05 and d ≤0.1)

1.5 Regressions

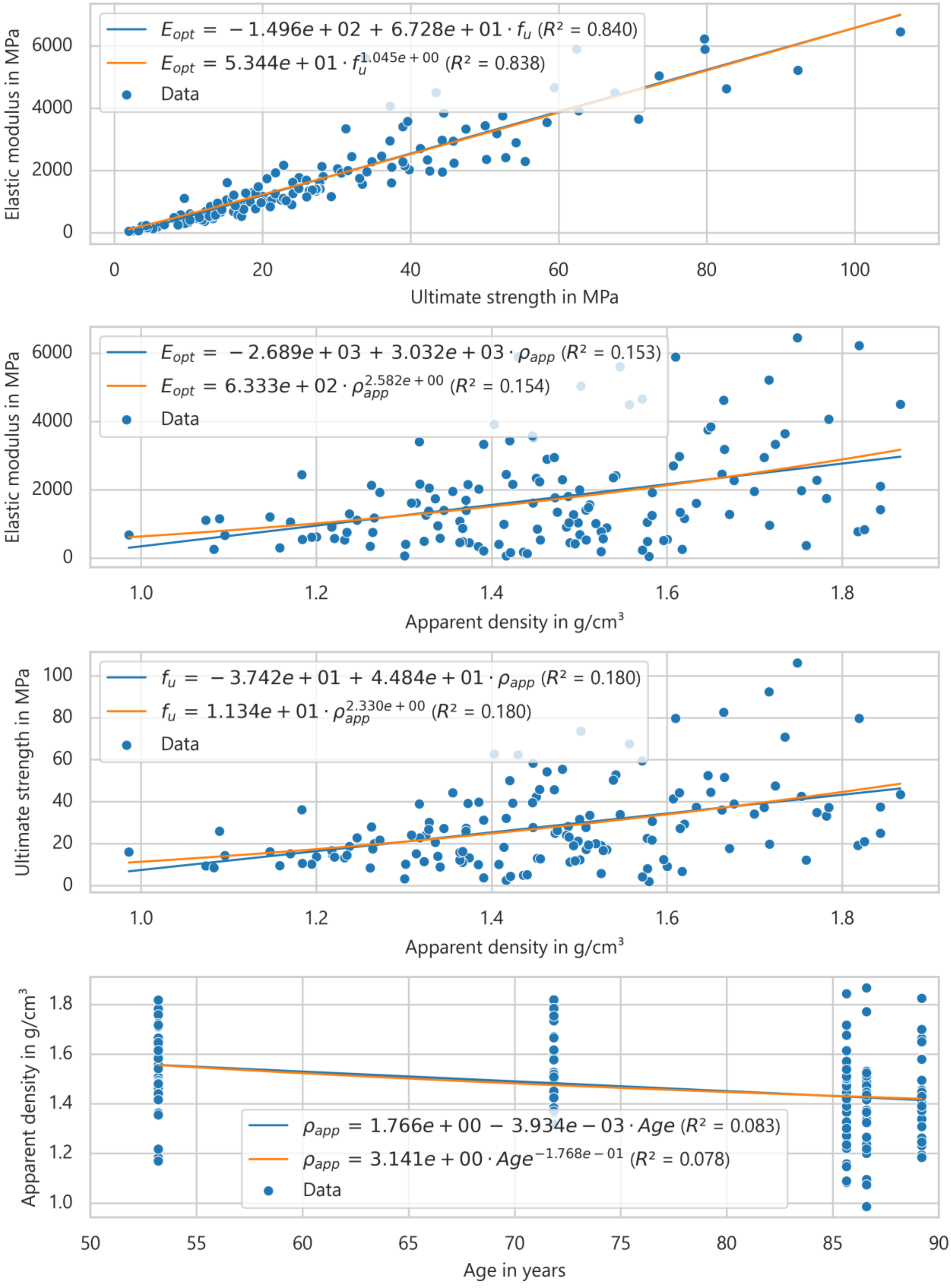

Fig. 12: Linear regressions of selected parameters of cortical bone specimens

2 Trabecular bone

2.1 Assessment code representations

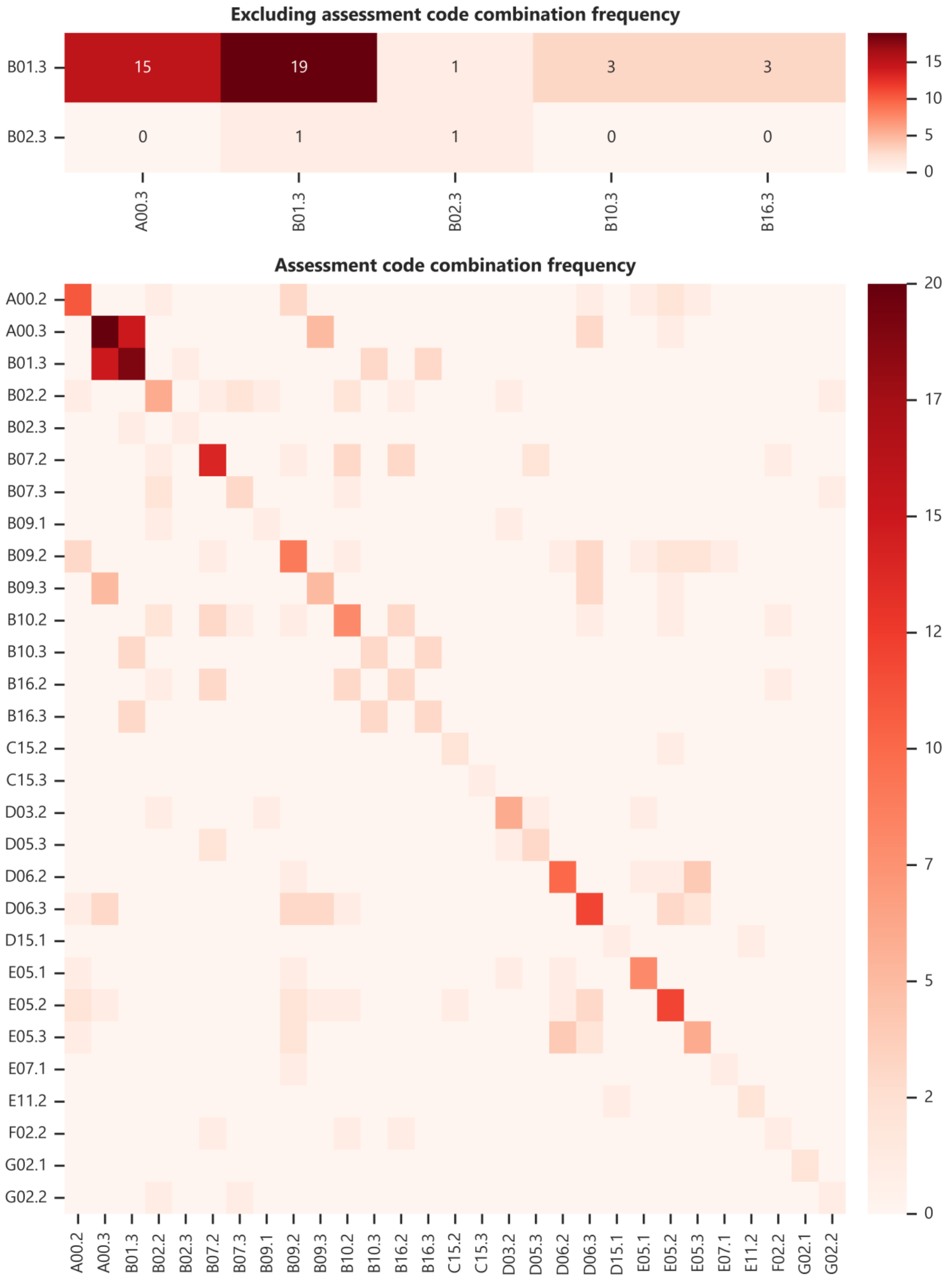

Fig. 13: Assessment code combination frequency for trabecular bone specimens (upper: excluding code combinations, lower: all combinations)

2.2 Exemplary test data visualization

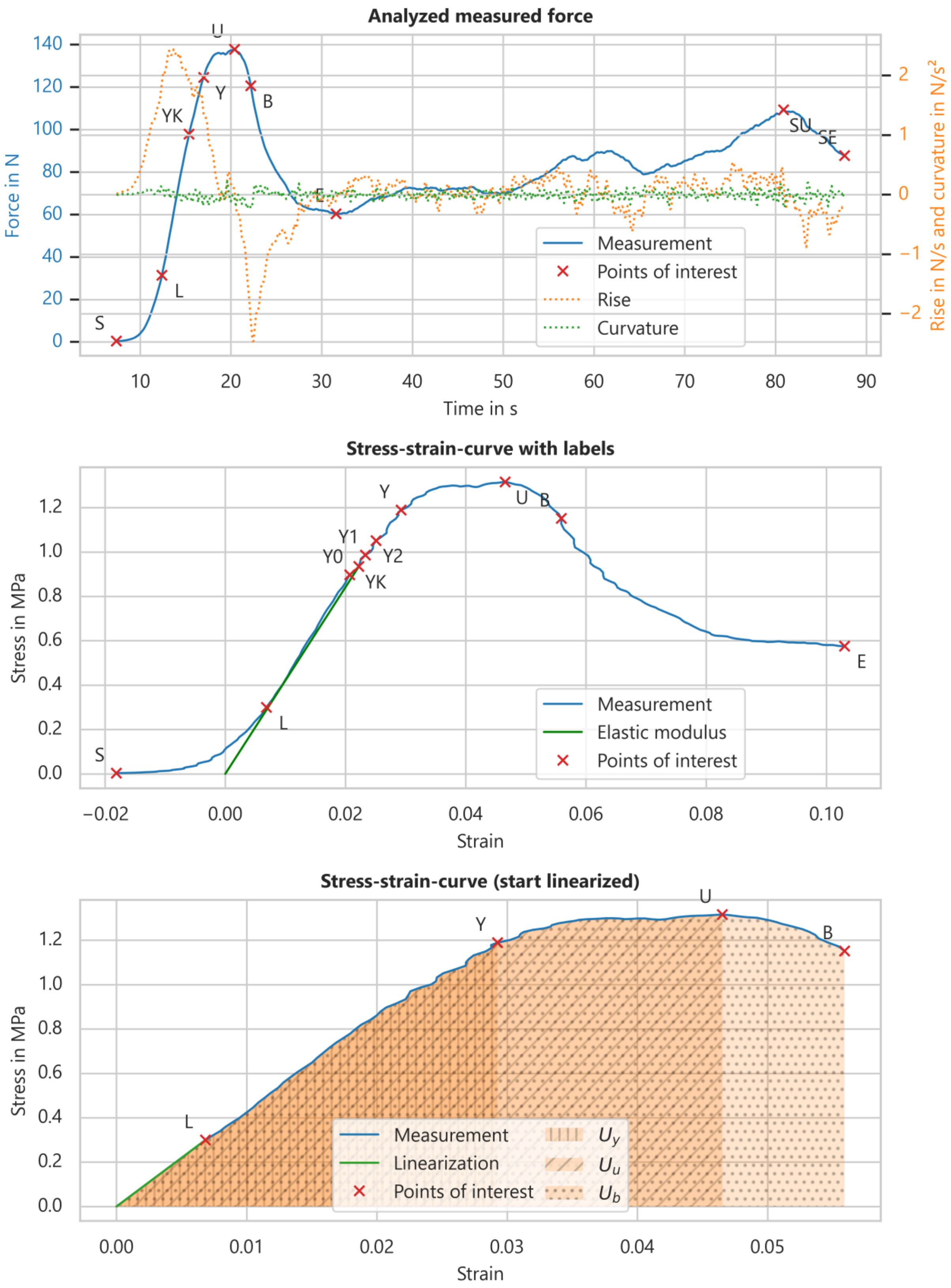

Fig. 14: Exemplary test data visualization of trabecular bone specimen 60-17 tm21y (from top to bottom: measured force with curve analyzation, stress-strain-curve and stress-strain curve with representation of strain energy)

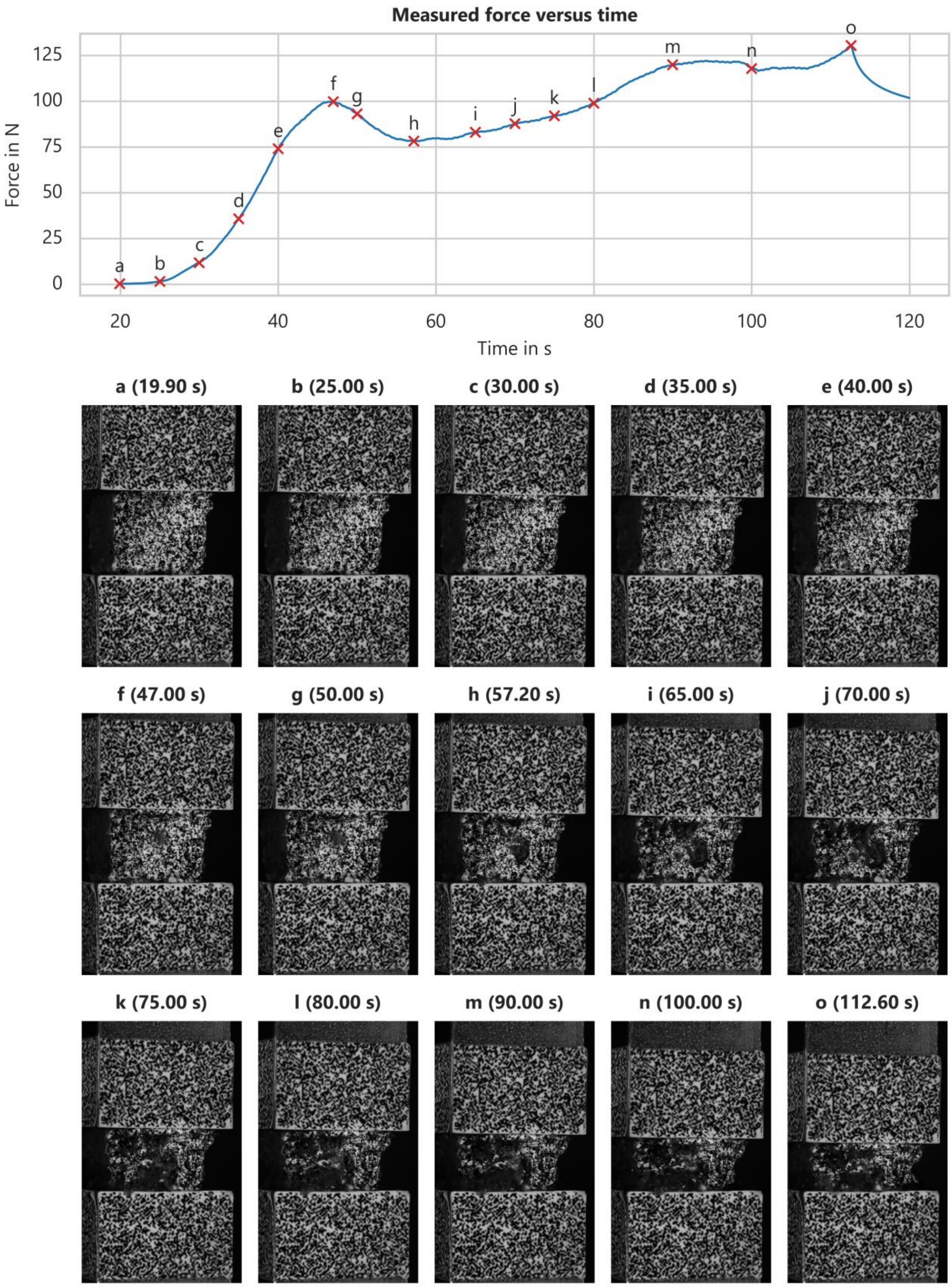

Fig. 15: Exemplary representation of the images from the left camera during mechanical testing of trabecular bone specimen 48-17 tl22y (top: measured force versus time with image marking, bottom: images for marked time)

2.3 Additional evaluation results

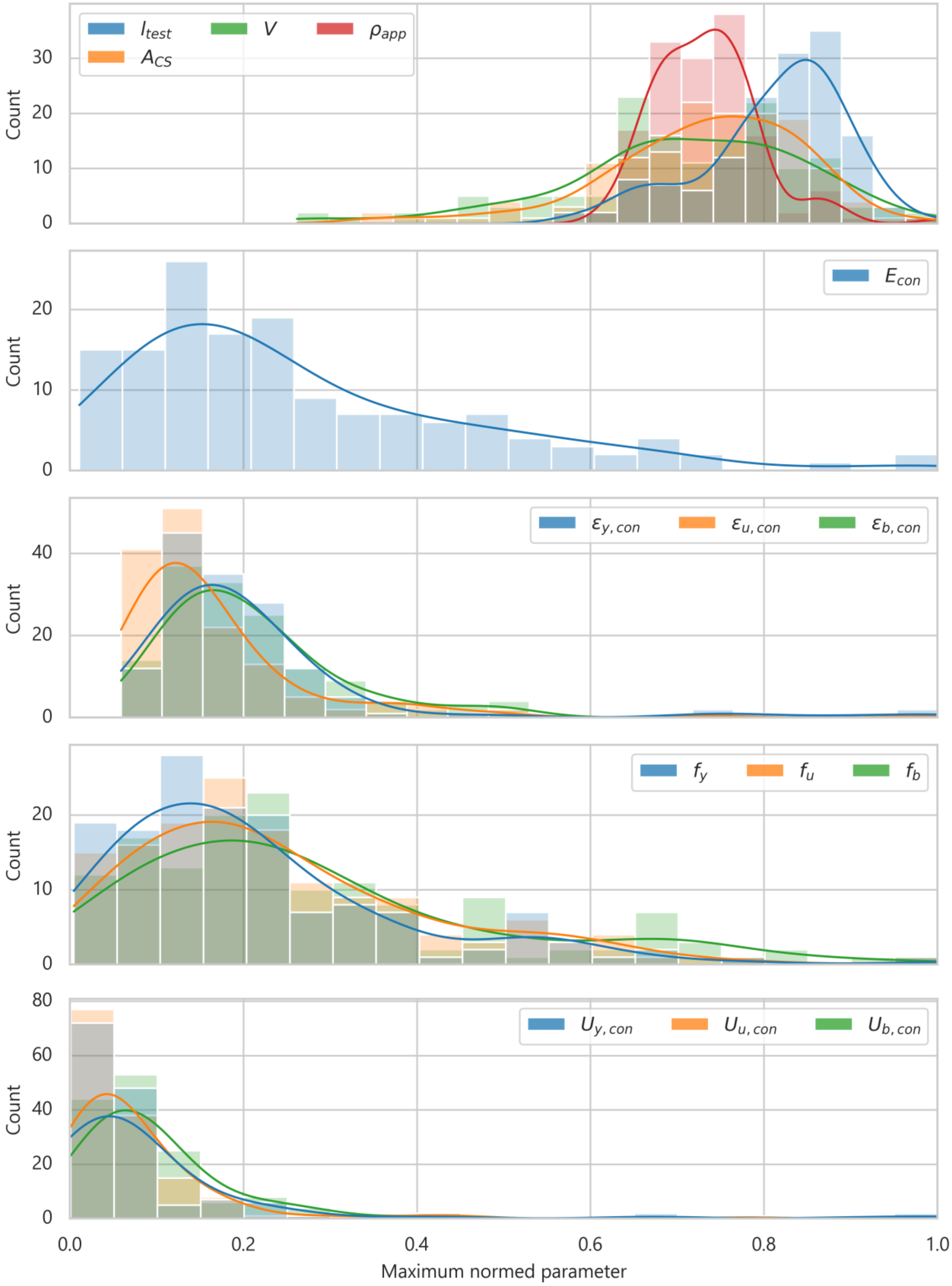

Fig. 16: Distributions of selected material parameters for trabecular bone specimens

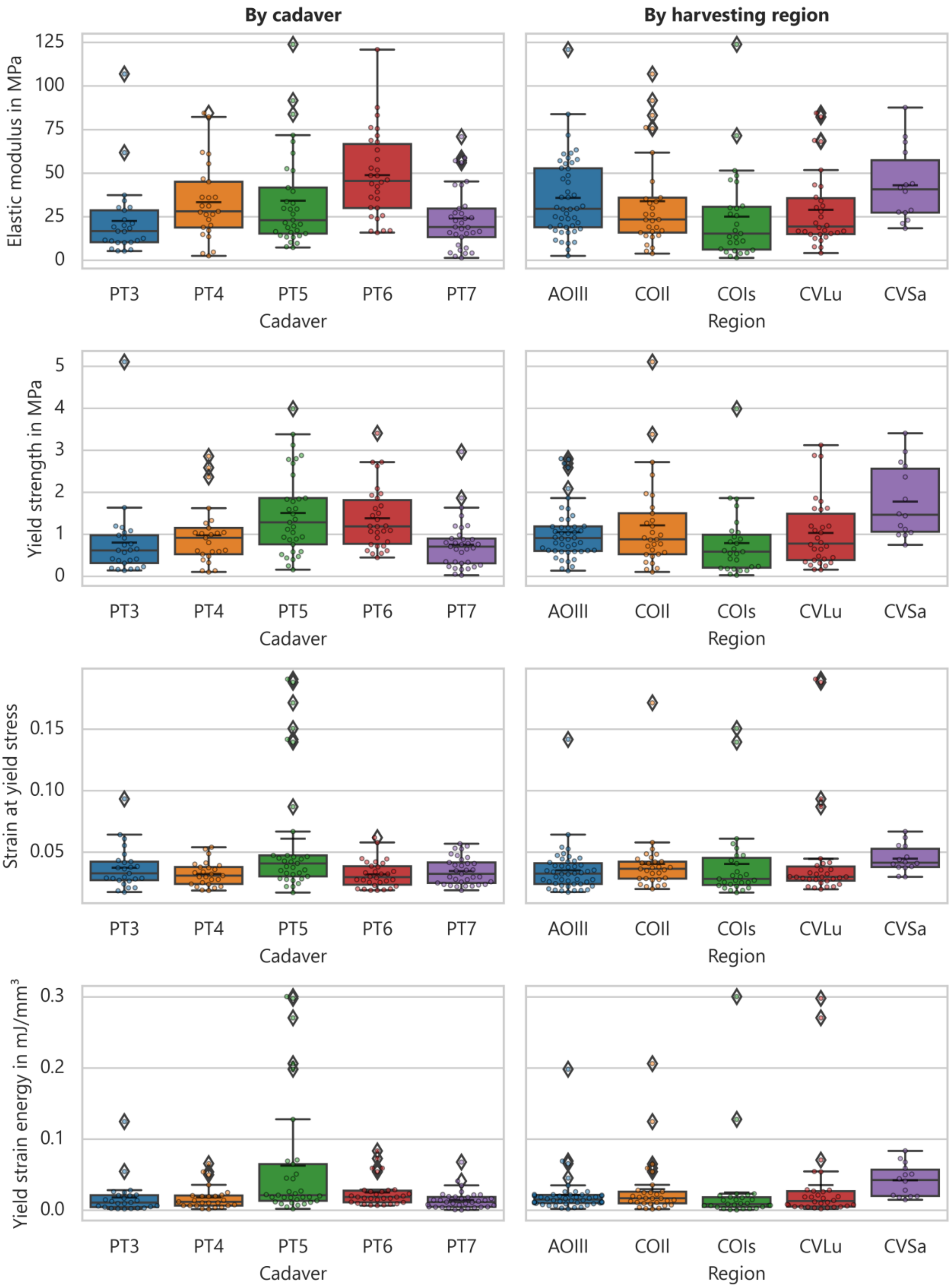

Fig. 17: Overview of selected elastic limit related material parameters for trabecular bone specimens related to cadaver (left) and harvesting region (right)

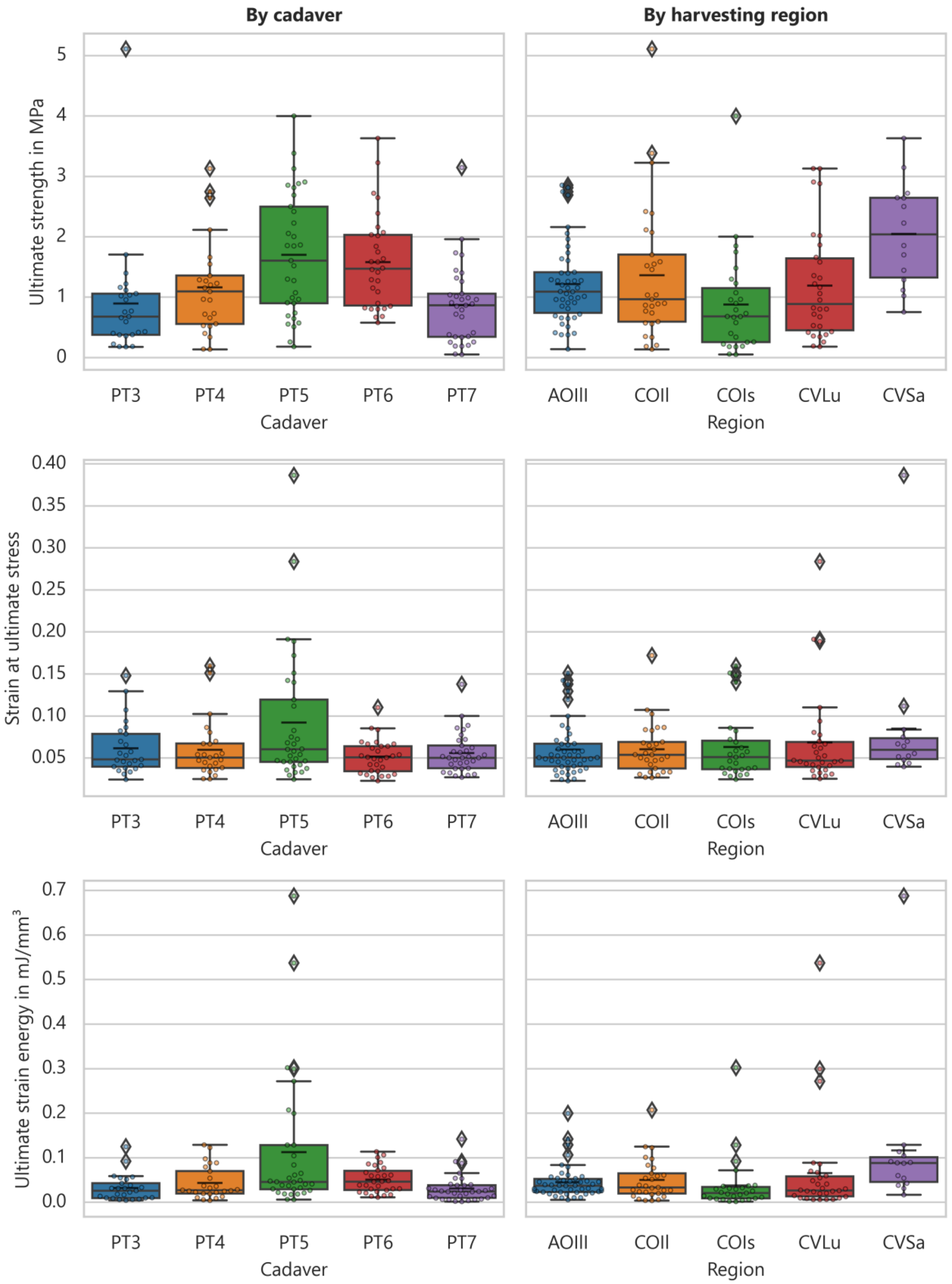

Fig. 18: Overview of selected ultimate limit related material parameters for trabecular bone specimens related to cadaver (left) and harvesting region (right)

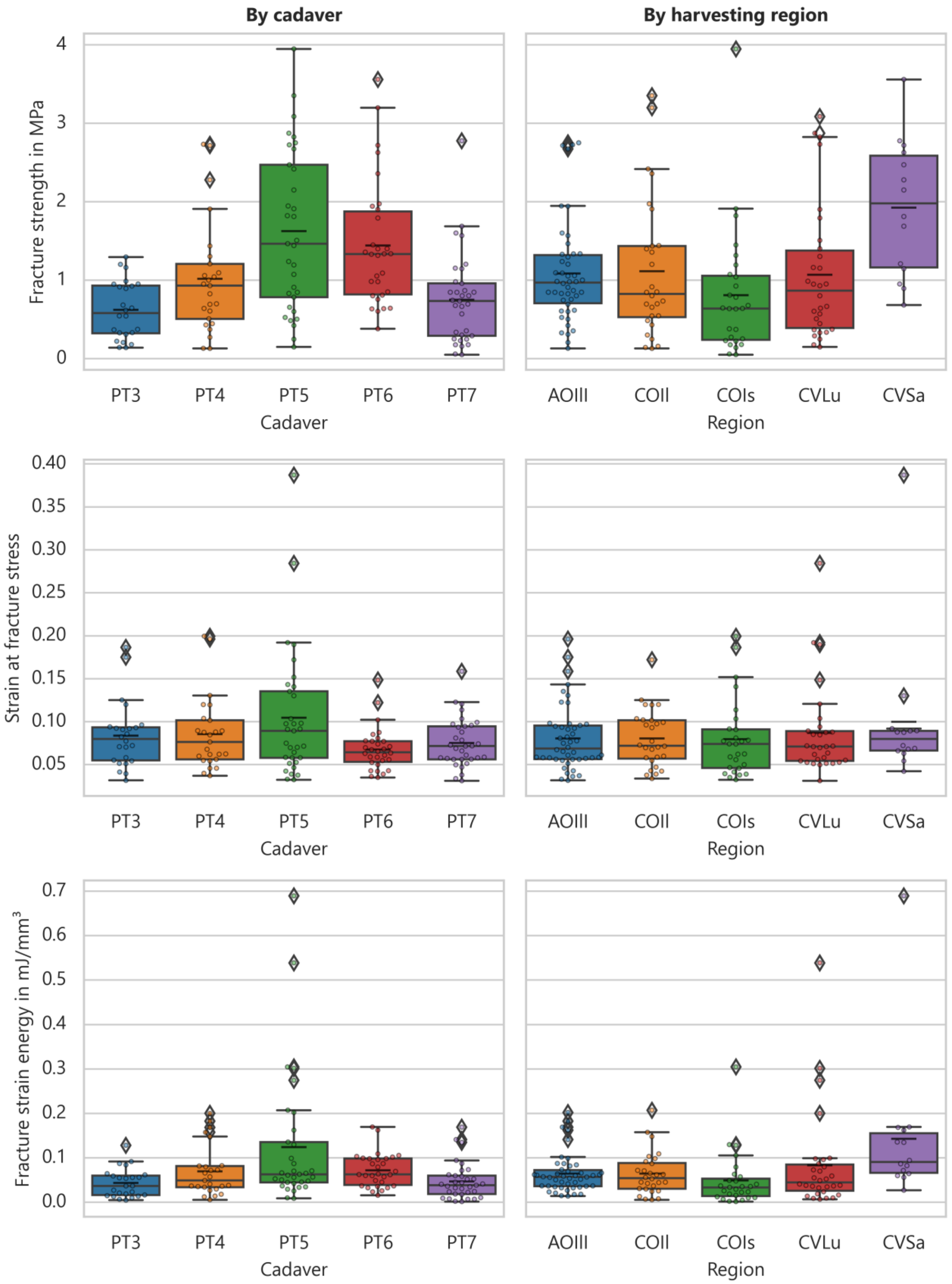

Fig. 19: Overview of selected fracture limit related material parameters for trabecular bone specimens related to cadaver (left) and harvesting region (right)

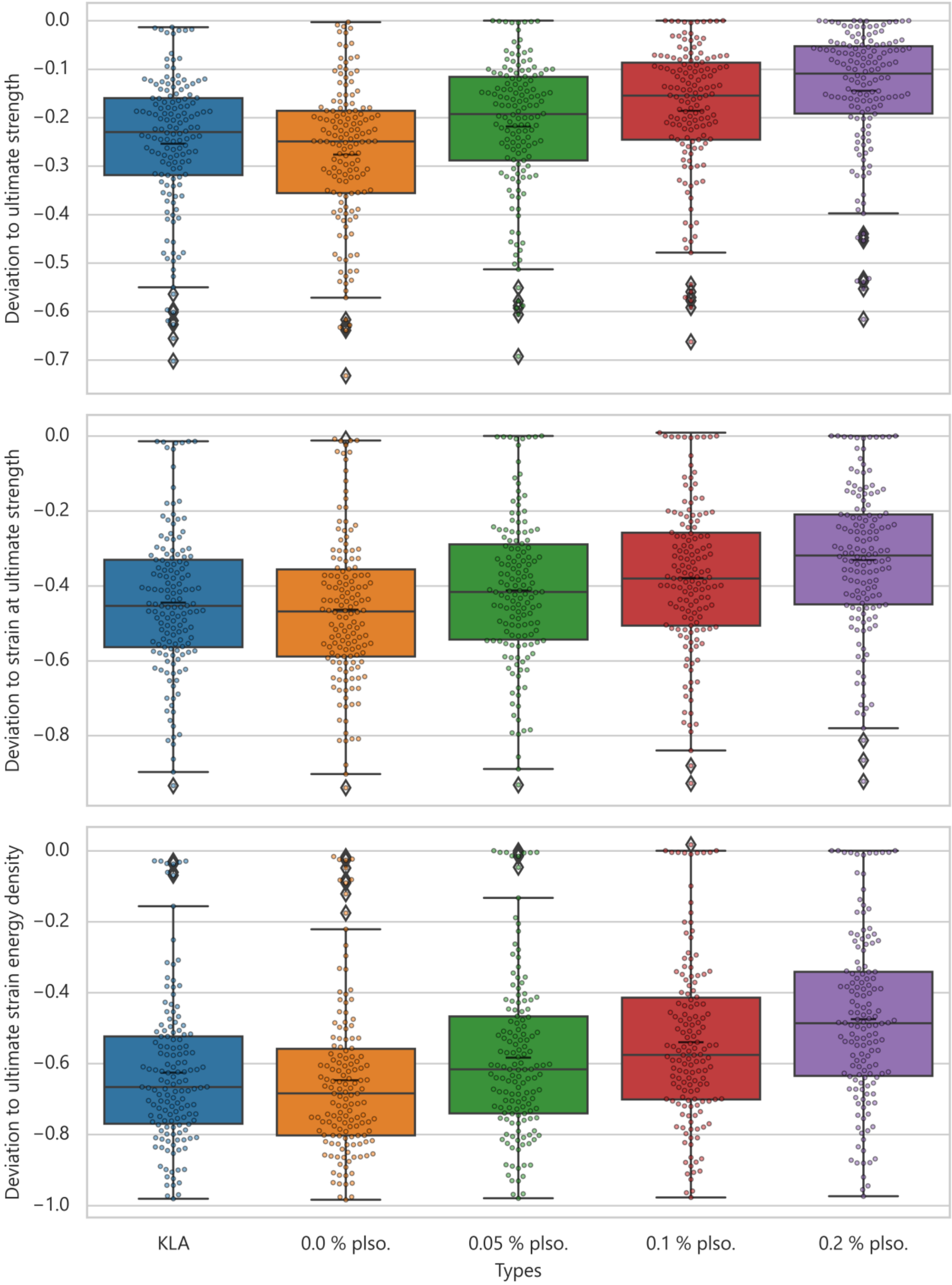

Fig. 20: Overview of selected elastic limit related material parameters for trabecular bone specimens related to different yield point determinations (KLA: Keuerleber approach, plso.: plastic strain offset)

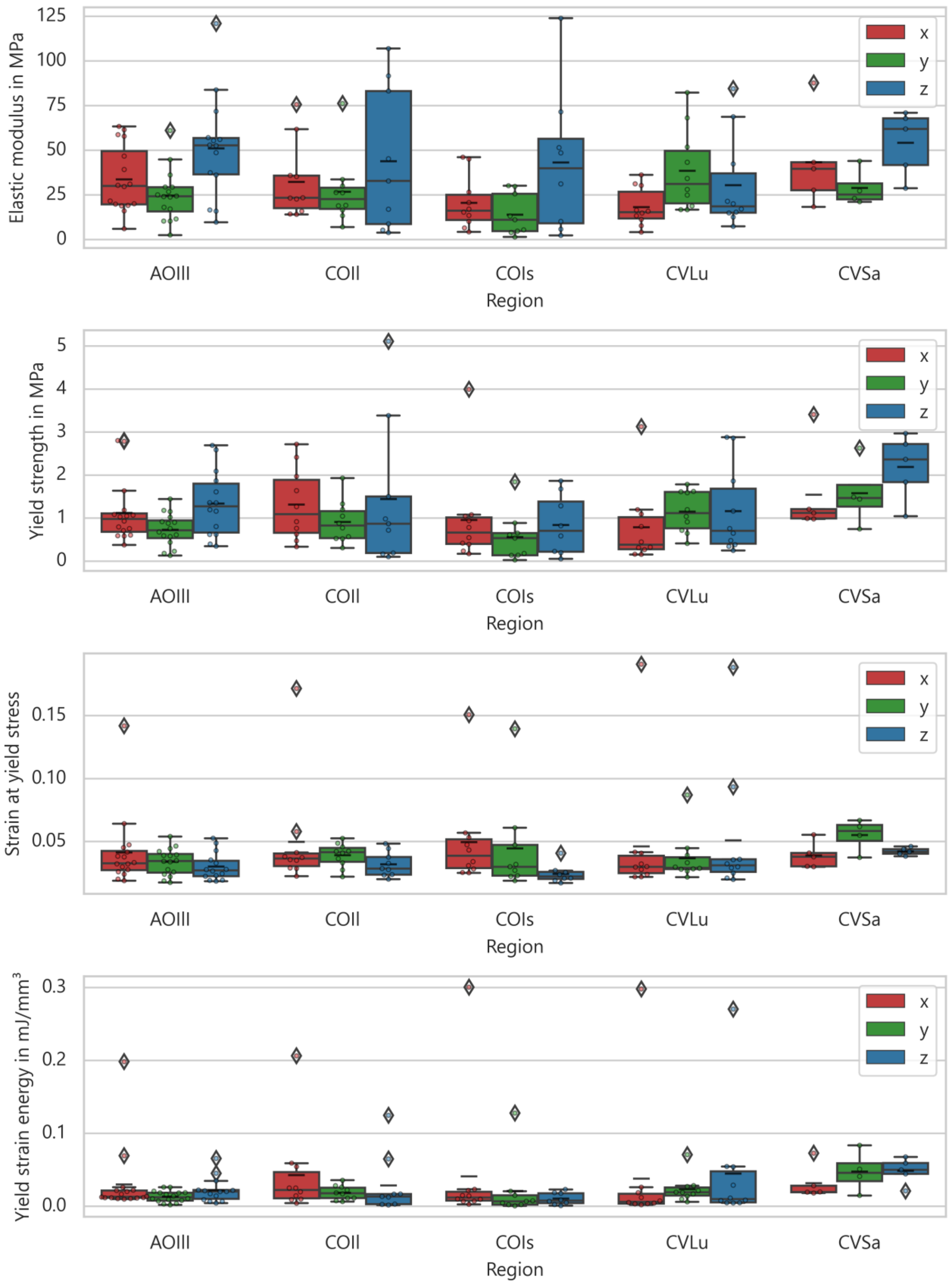

Fig. 21: Overview of selected elastic limit related material parameters for trabecular bone specimens related to harvesting region and local testing direction

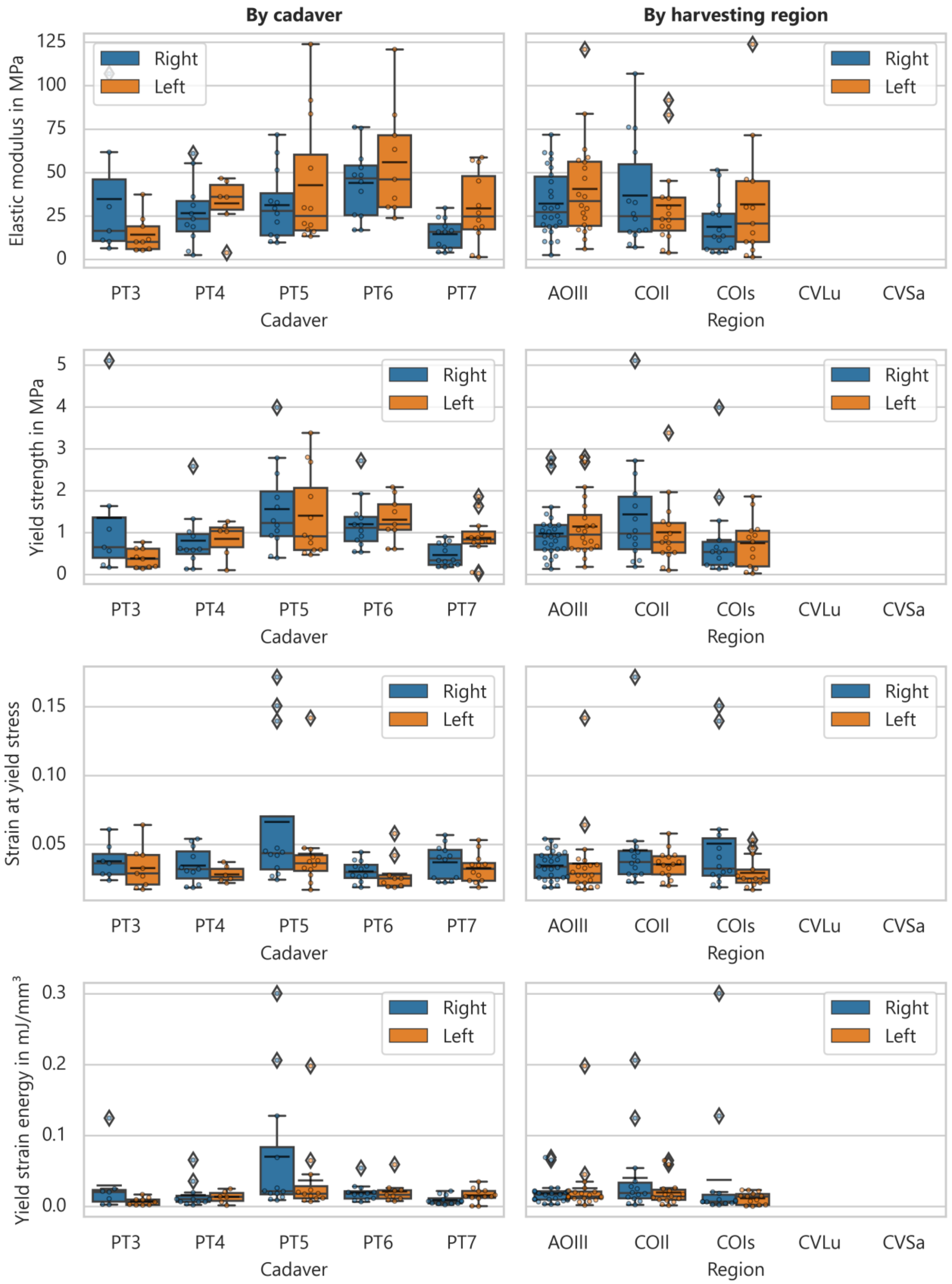

Fig. 22: Overview of selected elastic limit related material parameters for trabecular bone specimens related to cadaver (left) and harvesting region (right) as well as body side

2.4 Correlations

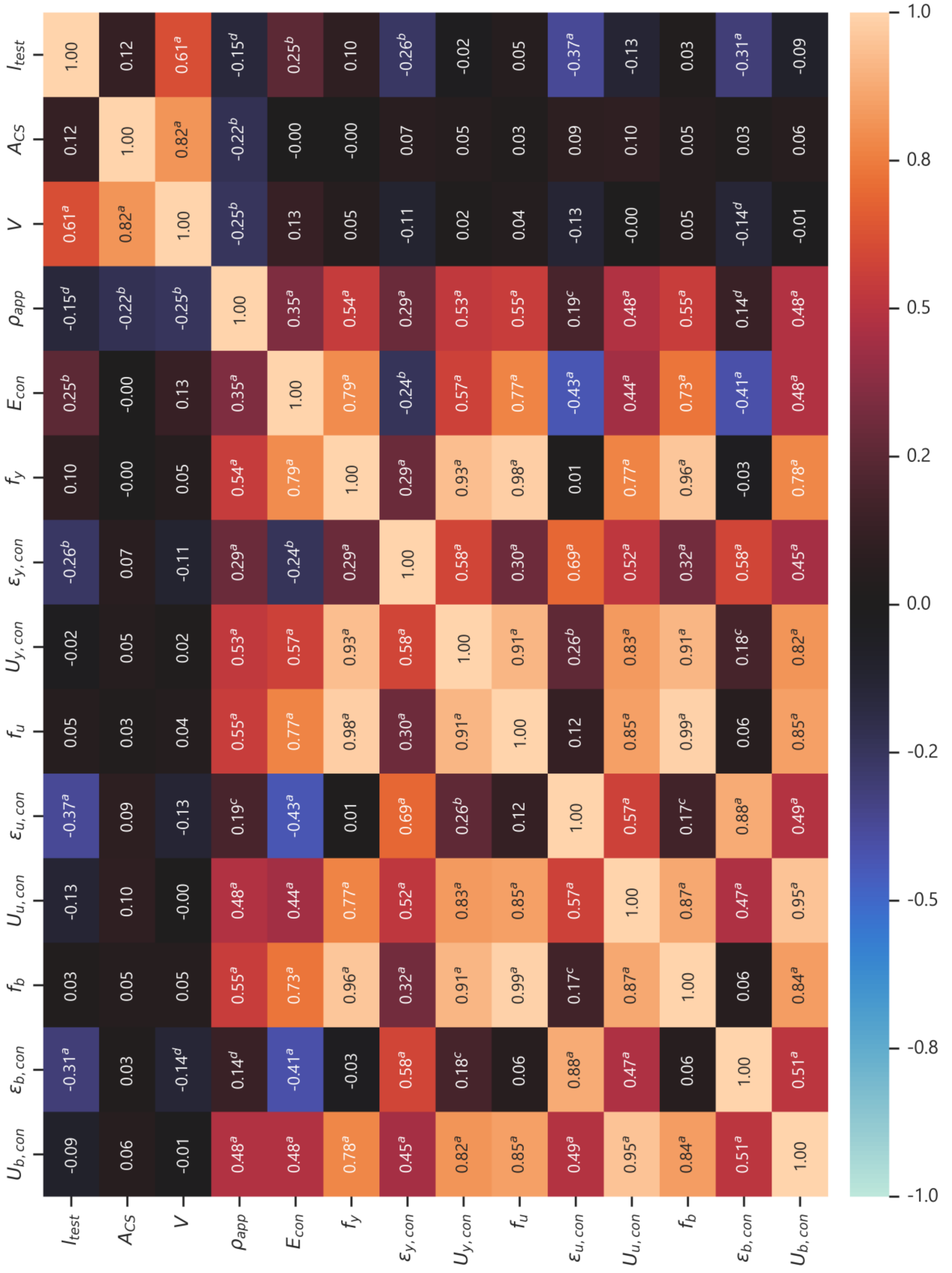

Fig. 23: Spearman correlation coefficients for selected parameters of trabecular bone specimens (associated significance level labeled: a ≤0.001, b ≤0.01, c ≤0.05 and d ≤0.1)

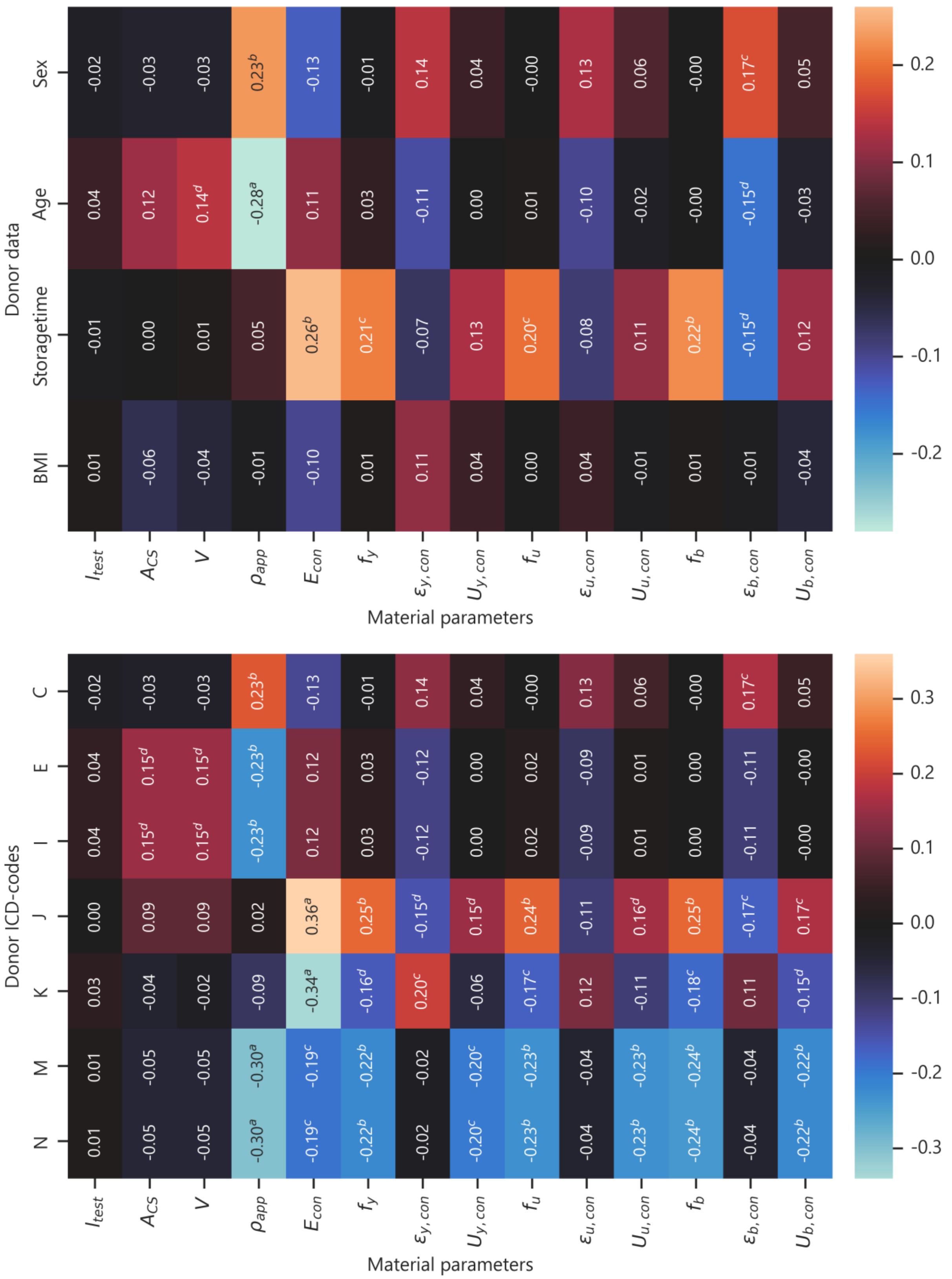

Fig. 24: Spearman correlation coefficients for selected parameters to donor-related data of trabecular bone specimens (associated significance level labeled: a ≤0.001, b ≤0.01, c ≤0.05 and d ≤0.1)

2.5 Regressions

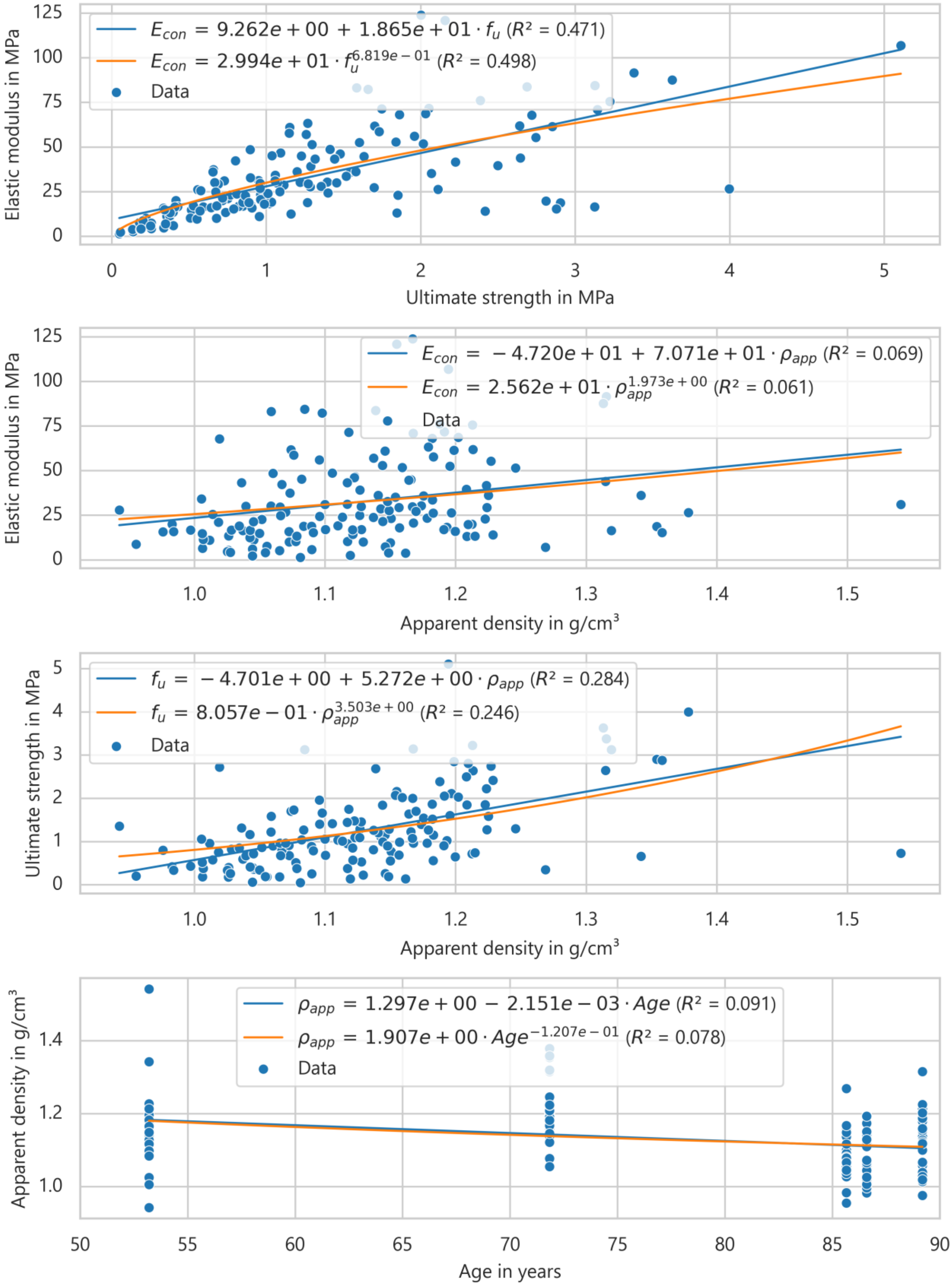

Fig. 25: Linear regressions of selected parameters of trabecular bone specimens

3 Soft tissue

3.1 Assessment code representations

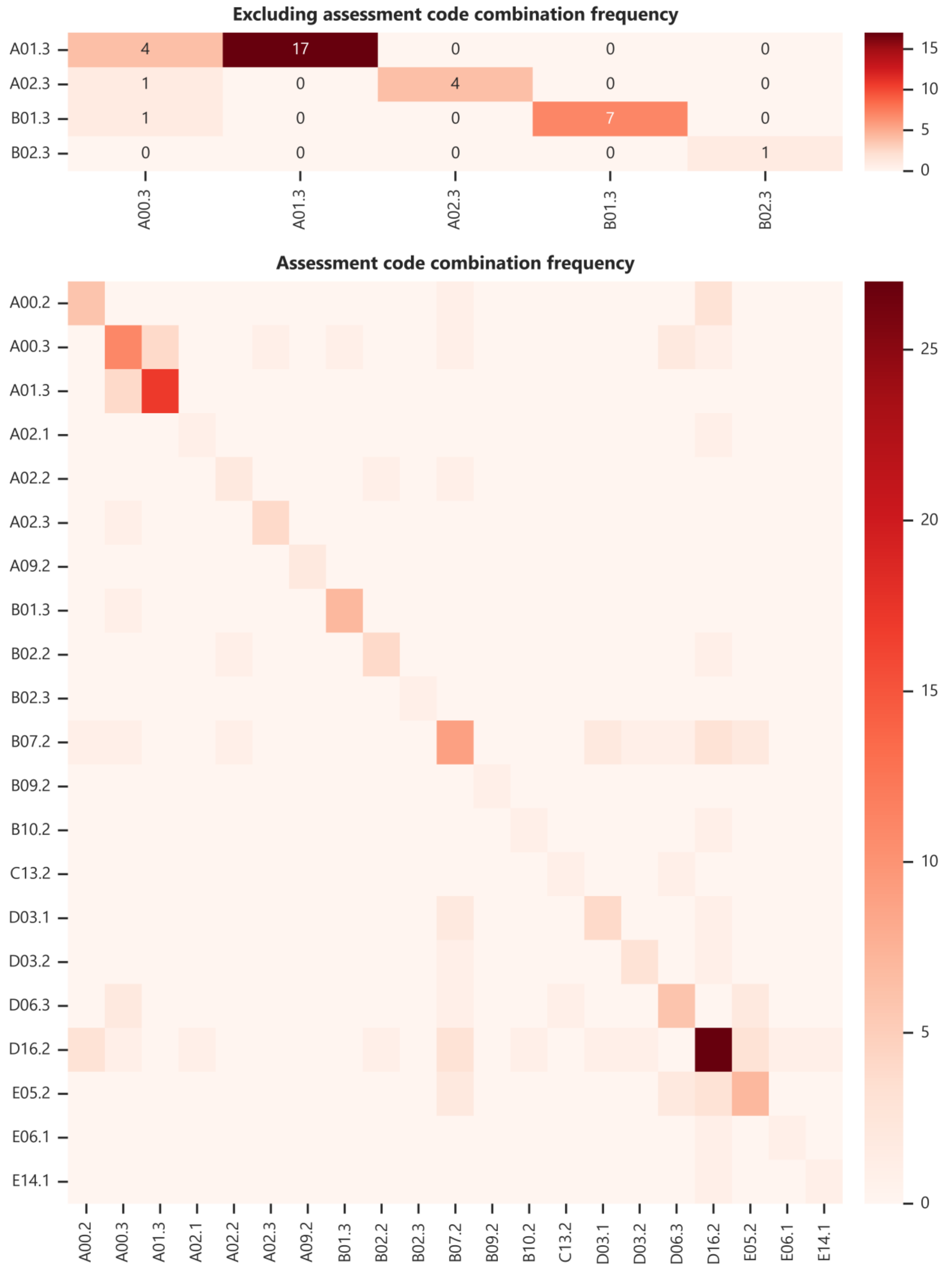

Fig. 26: Assessment code combination frequency for soft tissue specimens (upper: excluding code combinations, lower: all combinations)

3.2 Exemplary test data visualization

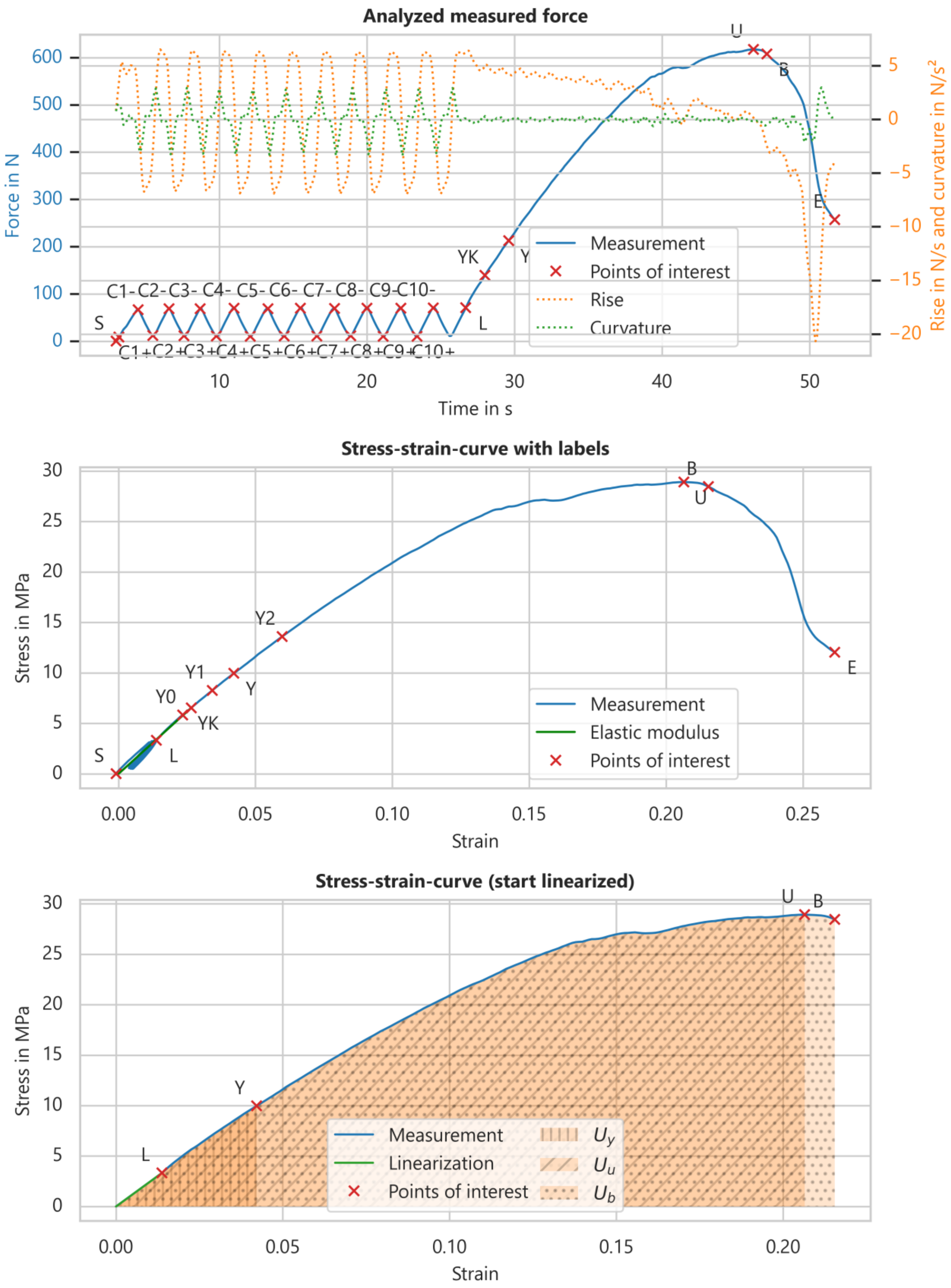

Fig. 27: Exemplary test data visualization of soft tissue specimen 60-17 sl03a (from top to bottom: measured force with curve analyzation, stress-strain-curve and stress-strain curve with representation of strain energy)

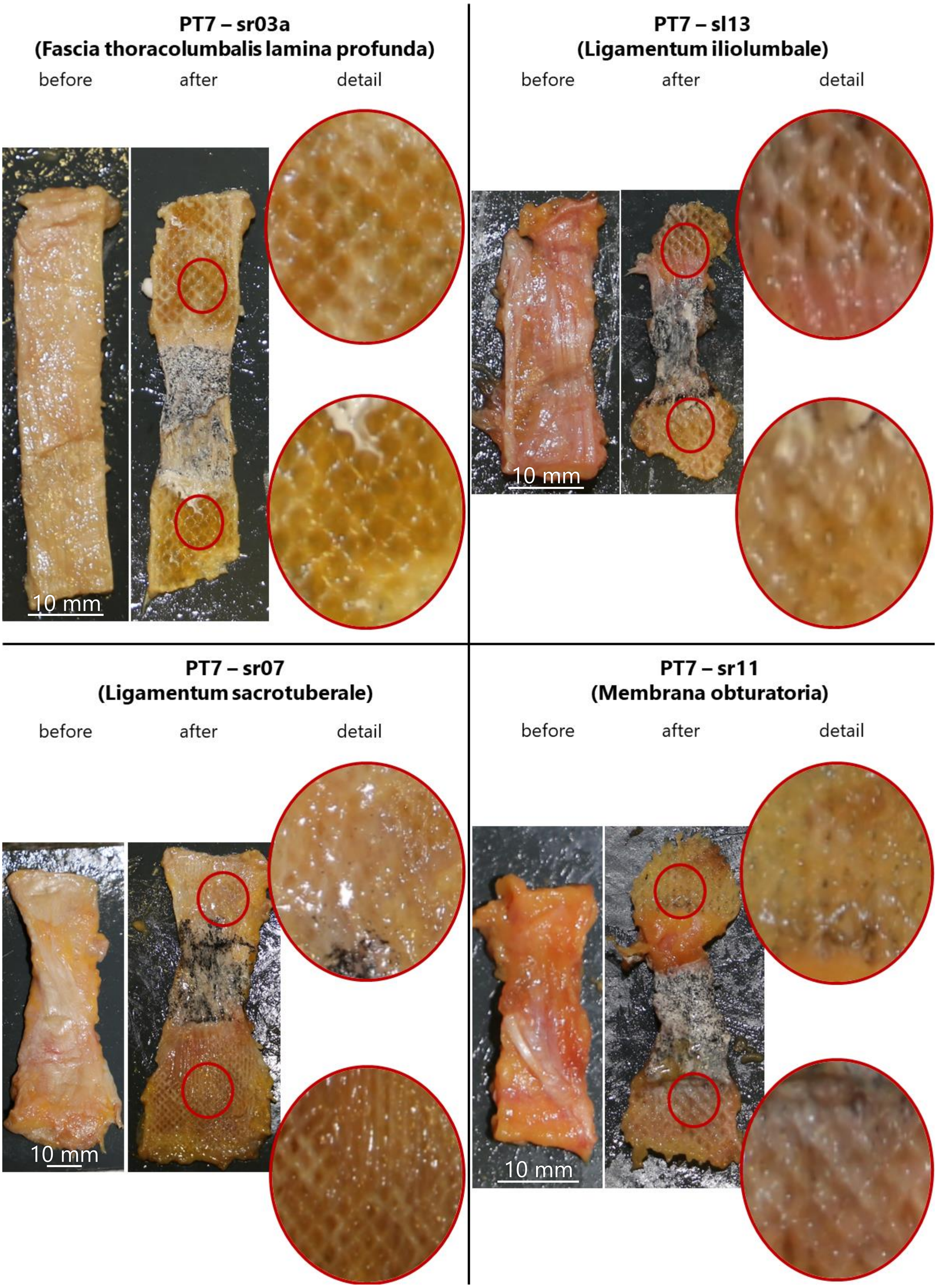

Fig. 28: Exemplary presentation of soft tissue specimens before and after mechanical testing, with additional detail of the clamping surfaces

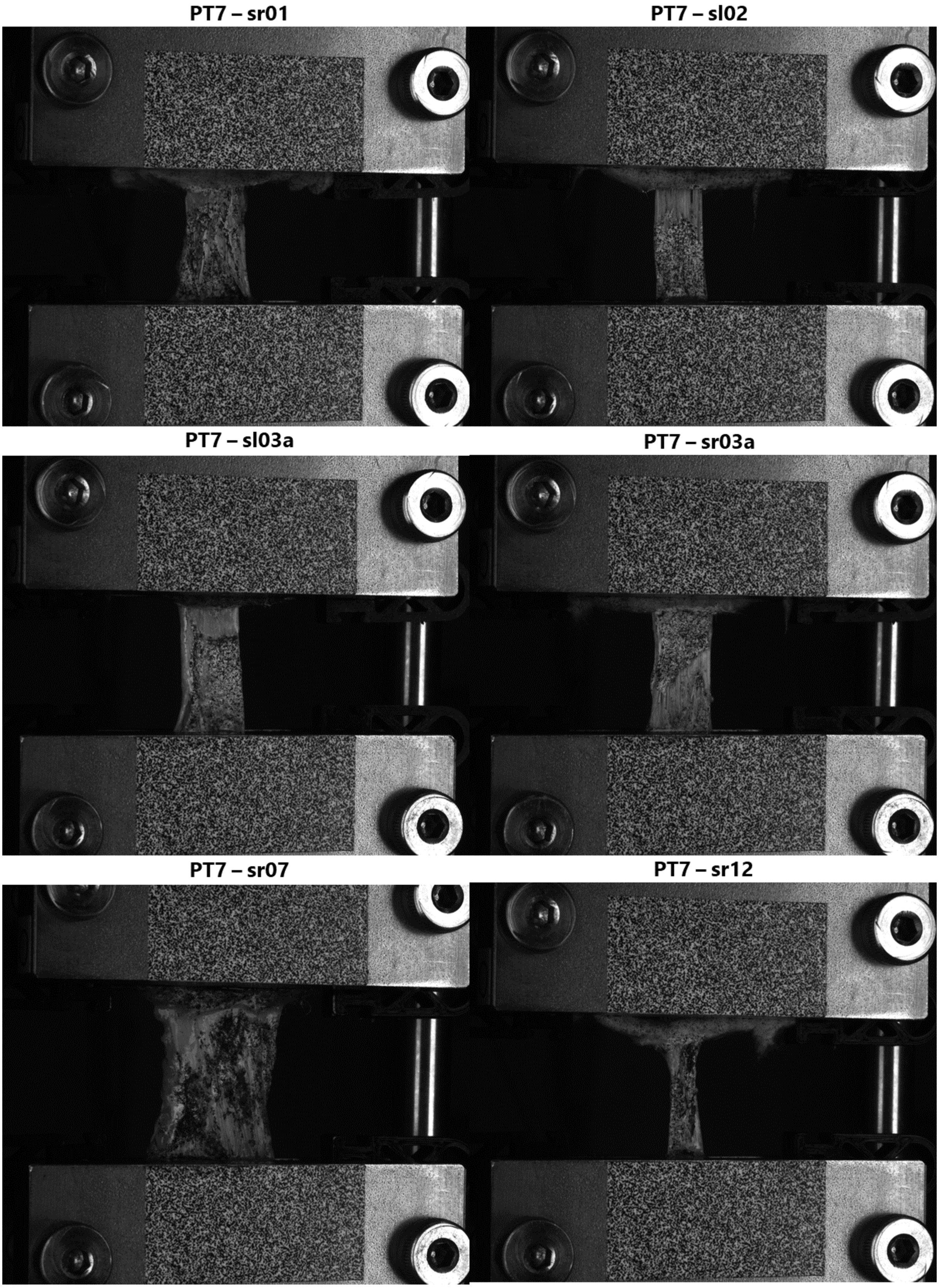

Fig. 29: Exemplary presentation of soft tissue specimens at end of mechanical testing

3.3 Additional evaluation results

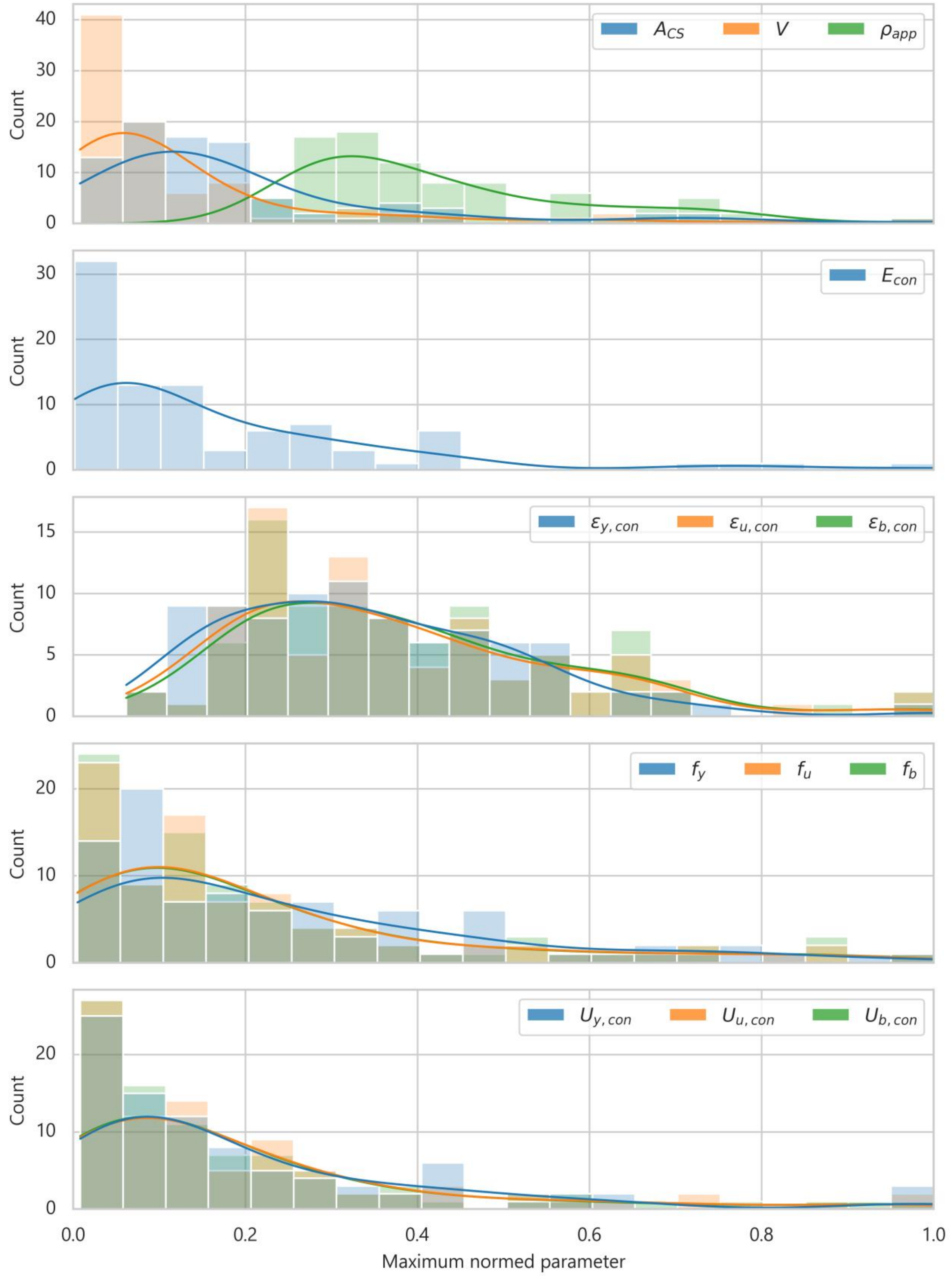

Fig. 30: Distributions of selected material parameters for soft tissue specimens

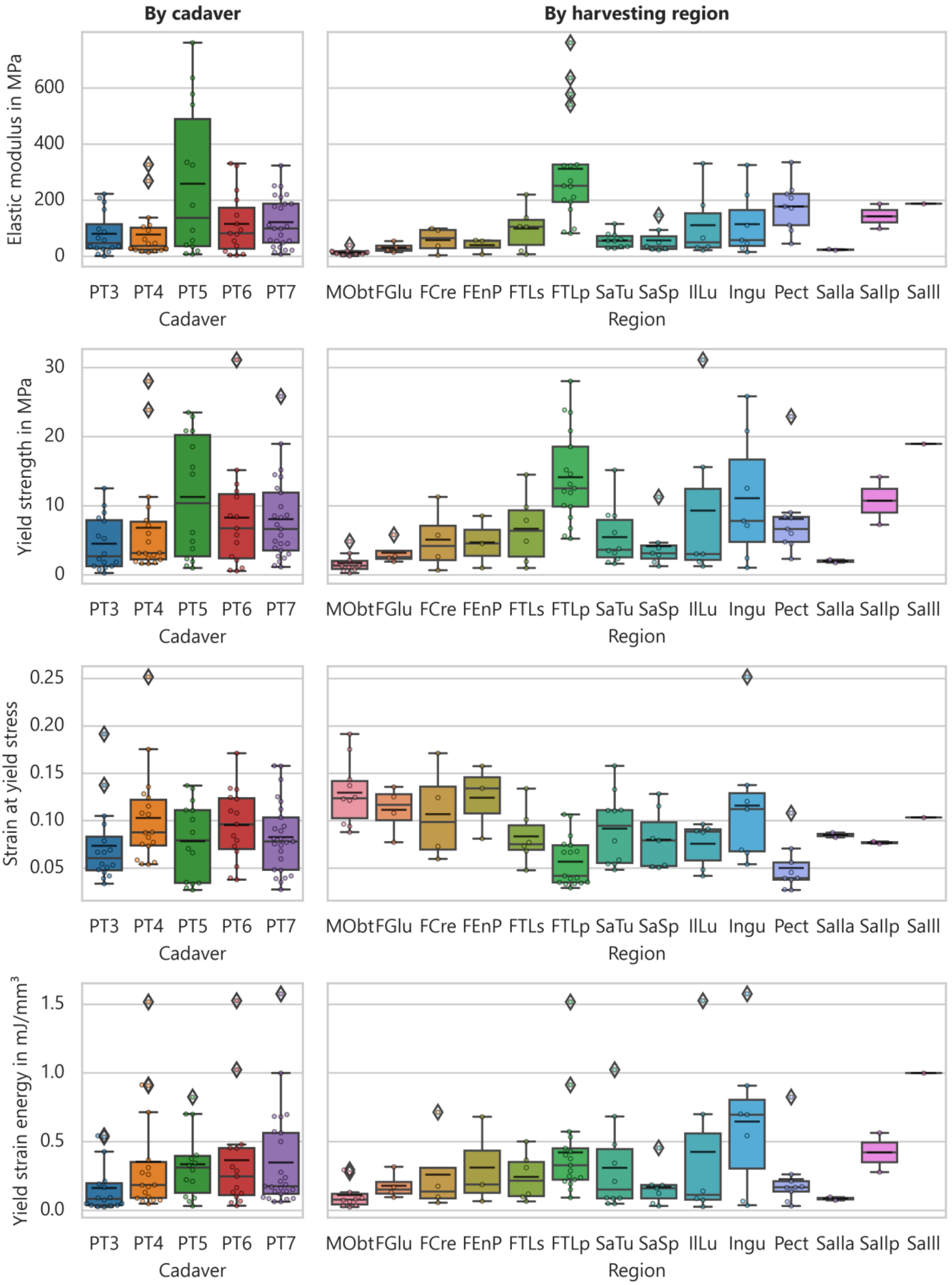

Fig. 31: Overview of selected elastic limit related material parameters for soft tissue specimens related to cadaver (left) and harvesting region (right)

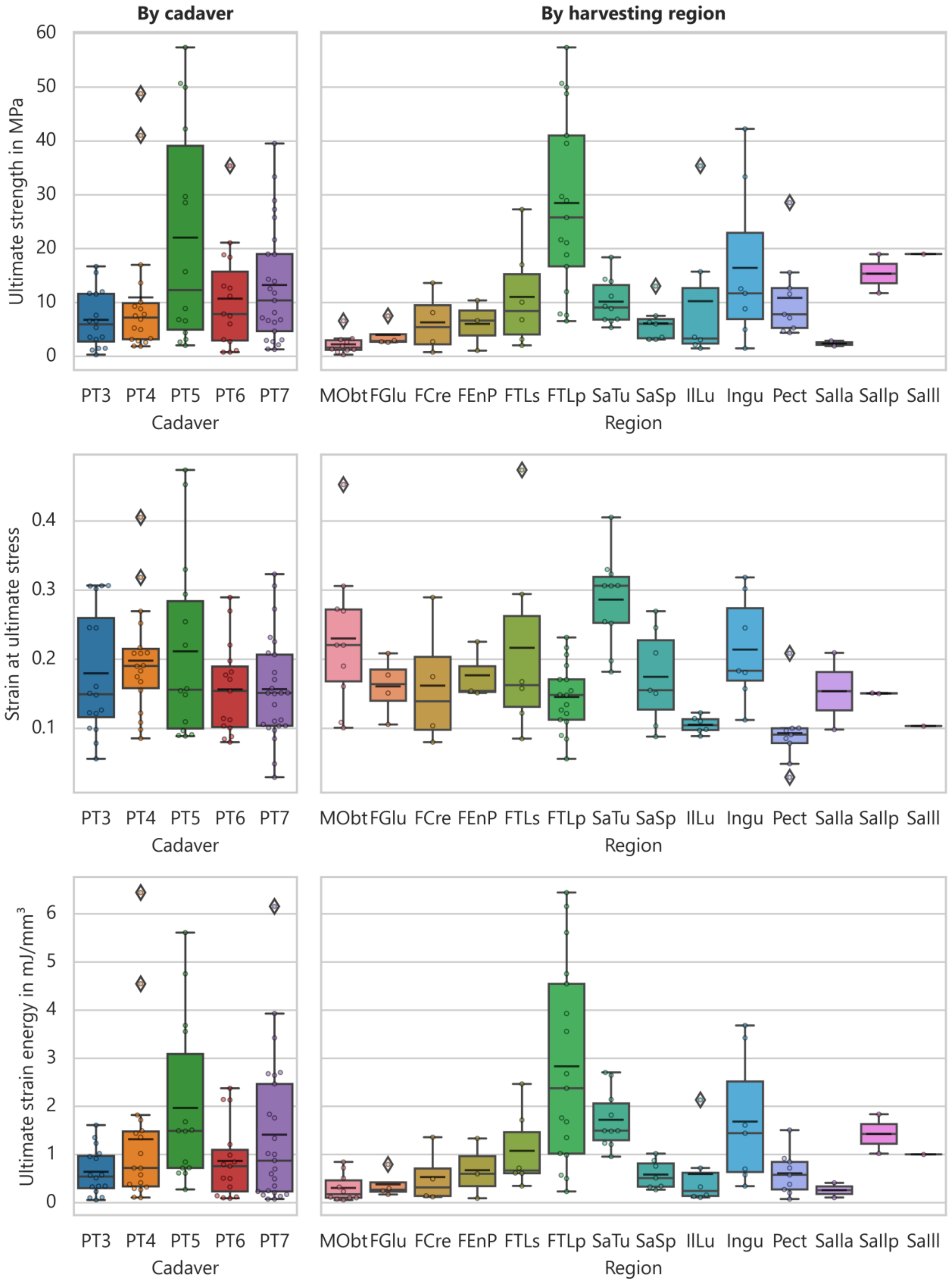

Fig. 32: Overview of selected ultimate limit related material parameters for soft tissue specimens related to cadaver (left) and harvesting region (right)

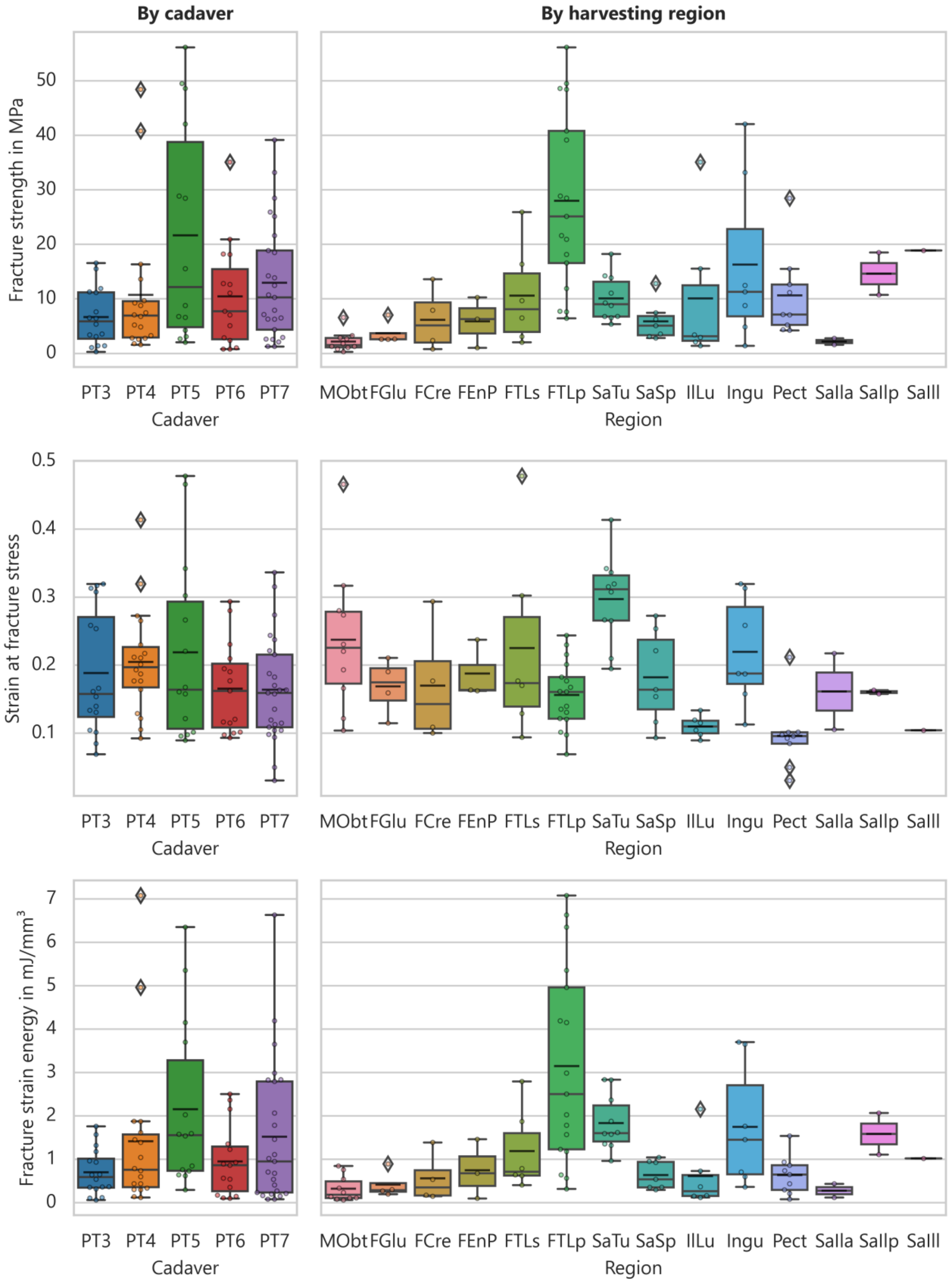

Fig. 33: Overview of selected fracture limit related material parameters for soft tissue specimens related to cadaver (left) and harvesting region (right)

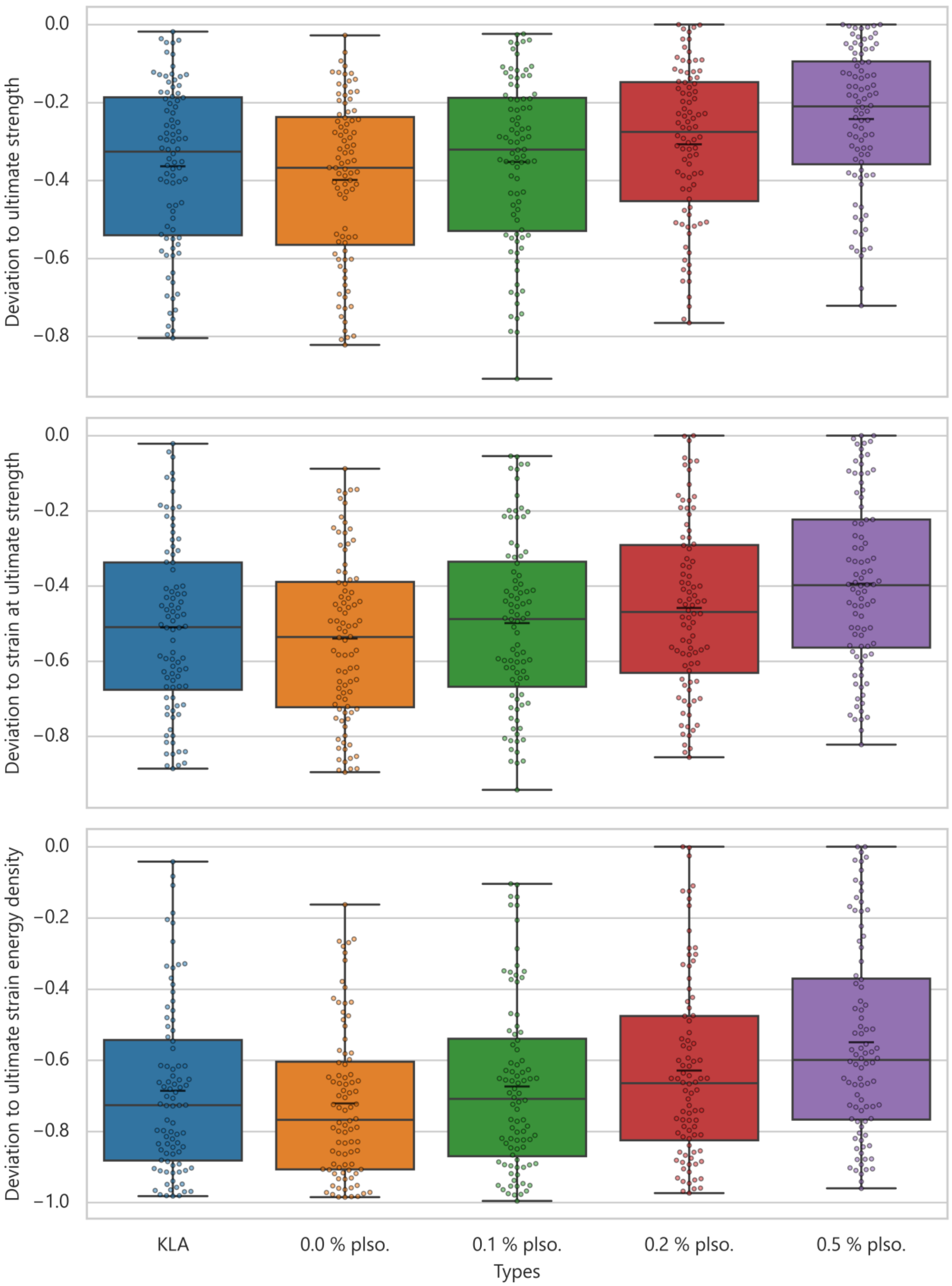

Fig. 34: Overview of selected elastic limit related material parameters for soft tissue specimens related to different yield point determinations (KLA: Keuerleber approach, plso.: plastic strain offset)

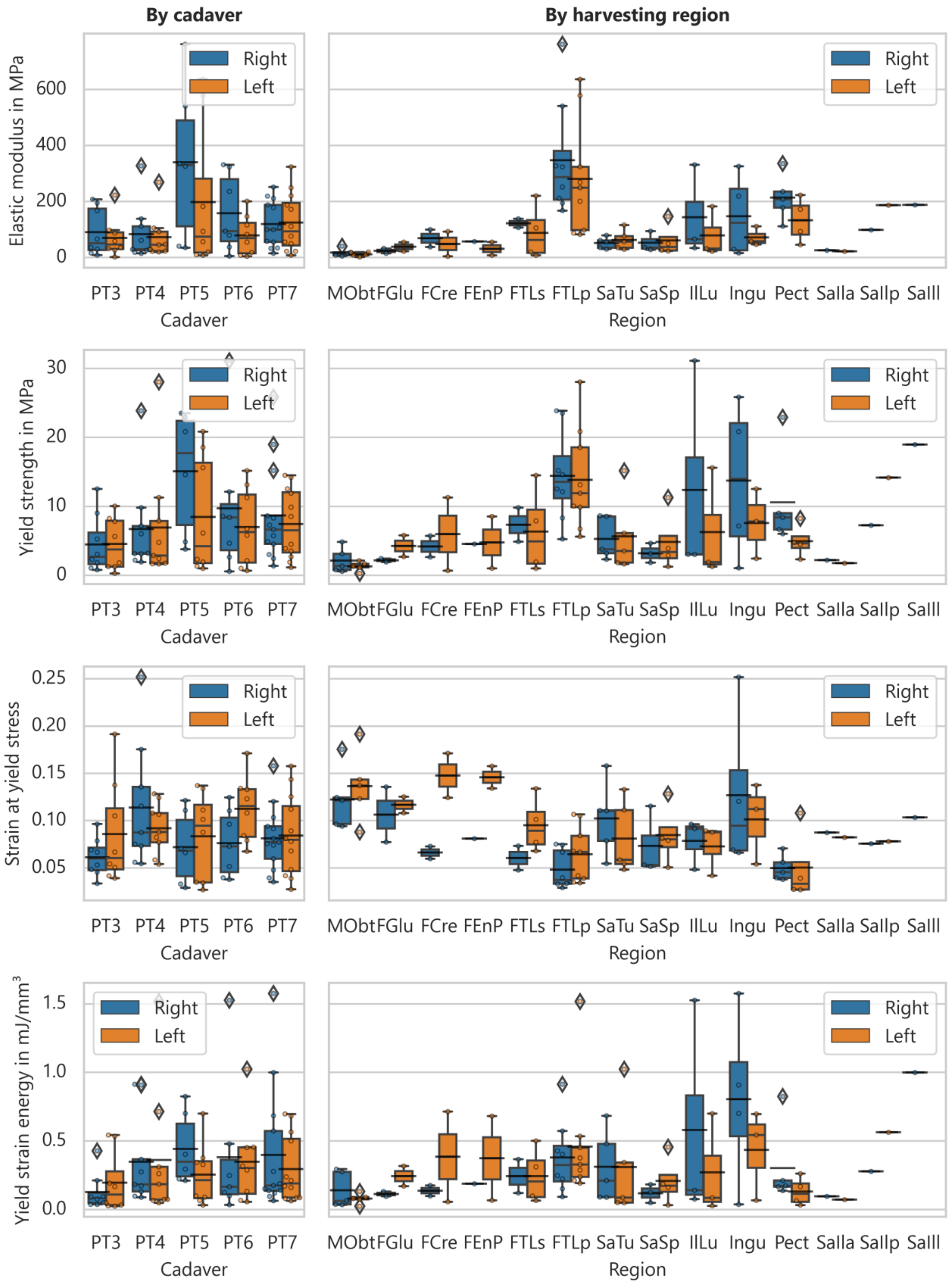

Fig. 35: Overview of selected elastic limit related material parameters for soft tissue specimens related to cadaver (left) and harvesting region (right) as well as body side

3.4 Correlations

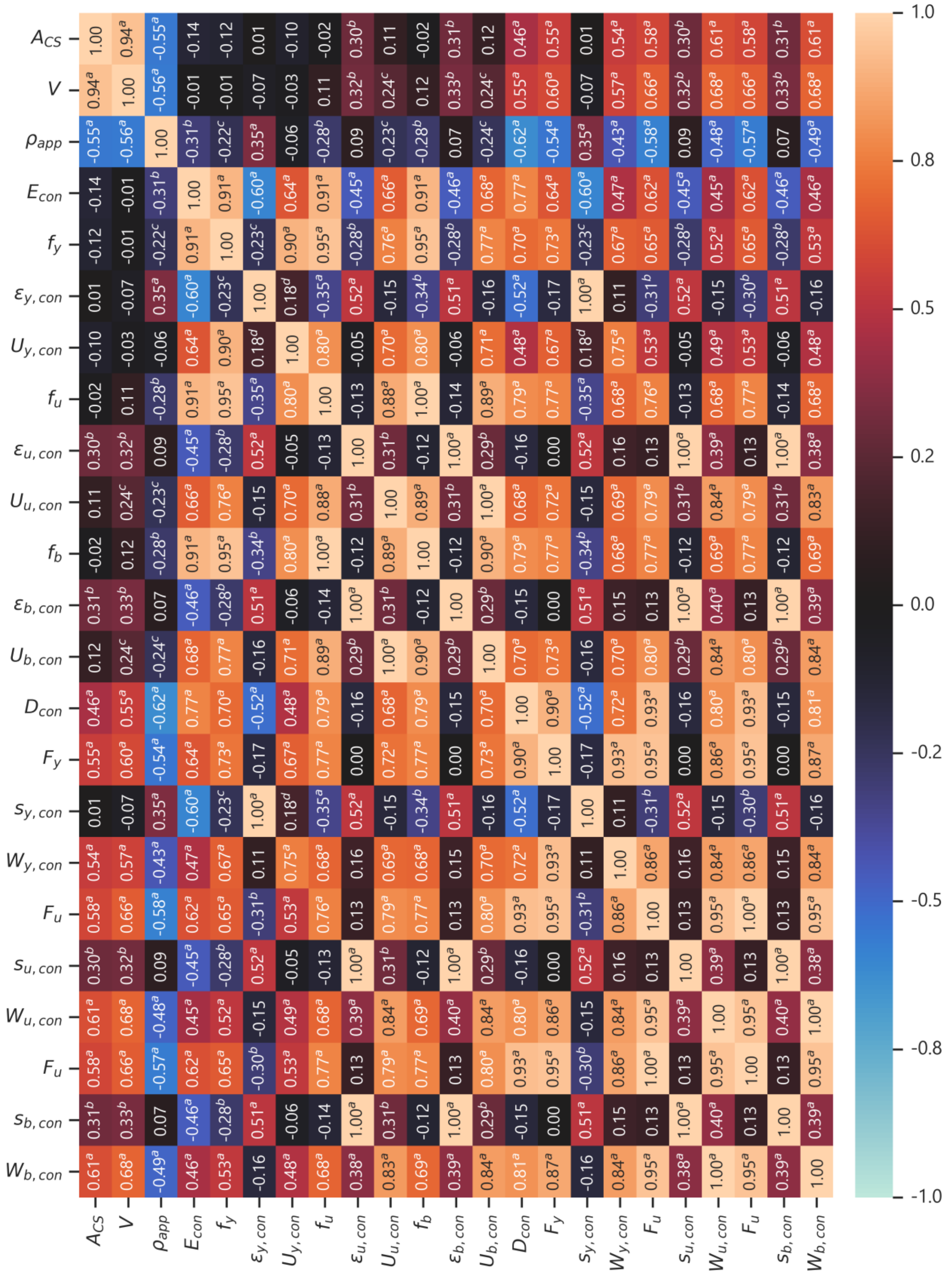

Fig. 36: Spearman correlation coefficients for selected parameters soft tissue specimens (associated significance level labeled: a ≤0.001, b ≤0.01, c ≤0.05 and d ≤0.1)

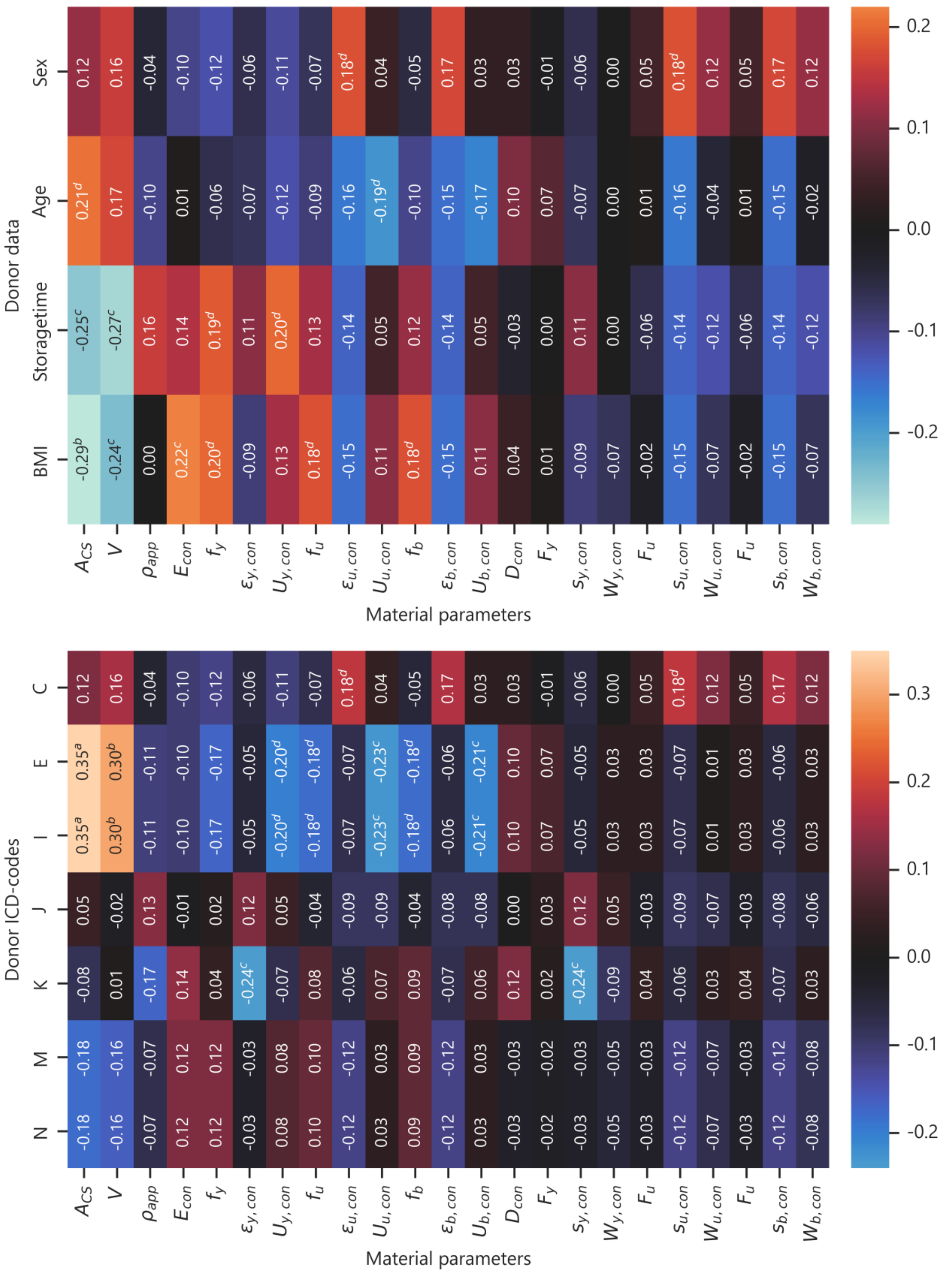

Fig. 37: Spearman correlation coefficients for selected parameters to donor-related data of soft tissue specimens (associated significance level labeled: a ≤0.001, b ≤0.01, c ≤0.05 and d ≤0.1)

3.5 Regressions

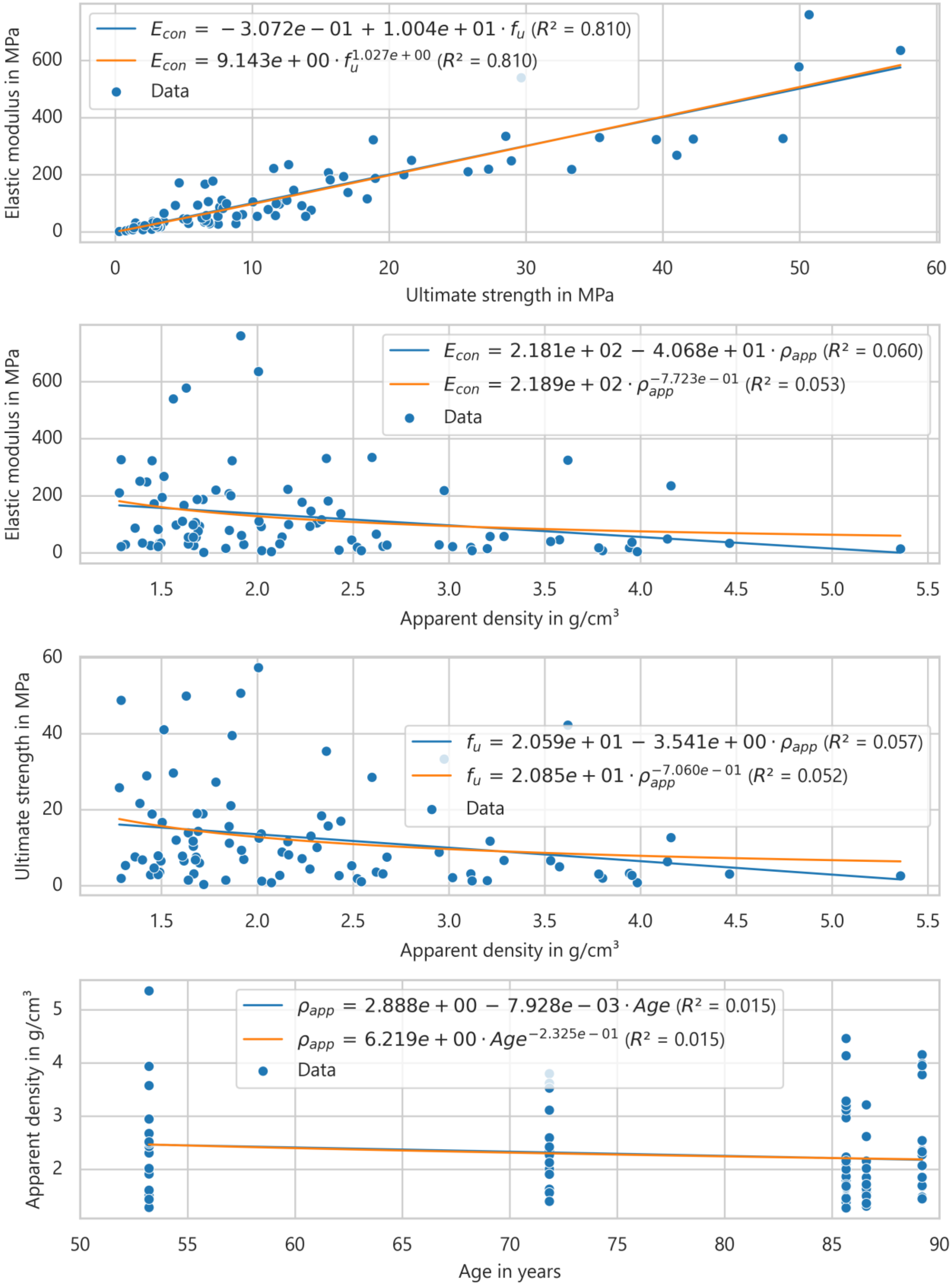

Fig. 38: Linear regressions of selected parameters of soft tissue specimens
